# Alpha-2-Macroglobulin as a New Promising Biomarker Improving the Diagnostic Sensitivity of Bovine Paratuberculosis

**DOI:** 10.3389/fvets.2021.637716

**Published:** 2021-03-05

**Authors:** Hyun-Eui Park, Jin-Sik Park, Hong-Tae Park, Jeong-Gyu Choi, Jeong-Ih Shin, Myunghwan Jung, Hyung-Lyun Kang, Seung-Chul Baik, Woo-Kon Lee, Donghyuk Kim, Han Sang Yoo, Min-Kyoung Shin

**Affiliations:** ^1^Department of Microbiology, Institute of Health Sciences, Gyeongsang National University School of Medicine, Jinju, South Korea; ^2^Department of Infectious Diseases, College of Veterinary Medicine, BK21Four and Bio-Max/N-Bio Institute, Seoul National University, Seoul, South Korea; ^3^Department of Convergence Medical Sciences, Gyeongsang National University, Jinju, South Korea; ^4^Schools of Energy & Chemical Engineering and Life Sciences, Ulsan National Institute of Science and Technology, Ulsan, South Korea

**Keywords:** Johne's disease, biomarkers, alpha-2-macroglobulin, cattle, serum

## Abstract

Johne's disease (JD) is a chronic granulomatous enteritis of ruminants caused by *Mycobacterium avium* subsp. *paratuberculosis* (MAP), which induces persistent diarrhea and cachexia. JD causes huge economic losses to the dairy industry due to reduced milk production and premature culling. Infected animals excrete MAP via feces during the prolonged subclinical stage without exhibiting any clinical signs. Therefore, accurate detection of subclinical stage animals is crucial for successful eradication of JD in the herd. In the current study, we analyzed serum samples of MAP-infected and non-infected cattle to identify potential biomarker candidates. First, we identified 12 differentially expressed serum proteins in subclinical and clinical shedder groups compared to the healthy control group. Second, we conducted ELISA for three selected biomarkers (alpha-2-macroglobulin (A2M), alpha-1-beta glycoprotein, and transthyretin) and compared their diagnostic performance with that of two commercial ELISA diagnostic kits. Serum A2M levels were significantly higher in the MAP-exposed, subclinical shedder, subclinical non-shedder, and clinical shedder groups than in the healthy control group, suggesting its possible use as a diagnostic biomarker for MAP infection. Furthermore, A2M demonstrated a sensitivity of 90.4%, and a specificity of 100% while the two commercial ELISA kits demonstrated a sensitivity of 67.83 and 73.04% and a specificity of 100%, respectively. In conclusion, our results suggest that measuring A2M by ELISA can be used as a diagnostic tool to detect MAP infection, considerably improving the detection rate of subclinical shedders and MAP-exposed animals that are undetectable using current diagnostic tools.

## Introduction

Johne's disease (JD) is a chronic granulomatous enteritis of ruminants caused by *Mycobacterium avium* subsp. *paratuberculosis* (MAP) that causes significant economic damage to dairy industries worldwide (1). The primary hosts of MAP are farmed and free-range ruminants, including cattle, lamb, goat, and deer (1). In addition, several researchers have also isolated MAP from various non-ruminant wildlife and captive animals such as rabbit, mice, rat, fox, raccoon, opossum, skunk, coyote, Cuban hutia, parrot, wallaby, and baboon which suggests the possibility of inter-species transmission (2–4). Furthermore, with the possibility of an association between MAP and human autoimmune diseases such as Crohn's disease, multiple sclerosis, type 1 diabetes, and rheumatoid arthritis, JD has become an important public health disease (5–7).

The prominent clinical features of JD are persistent diarrhea, progressive weight loss, decreased milk production, and debilitation after a prolonged incubation period (1). During disease progression, infected cattle shed MAP with their feces, and bacterial shedding subsequently contaminates water, feed, milk, and the surrounding environment (1, 8). Due to the waxy mycolic acid layer, MAP endure harsh environmental conditions, including dryness, low pH, and high or low temperatures; MAP viability in pastures can be maintained for over 1 year and much longer periods in water (9, 10). Ingestion of MAP-contaminated materials is the primary route of infection. Therefore, rapid detection and culling of subclinical fecal shedder from herd are needed for the control of the JD (1, 8).

The diagnosis of JD is mainly divided into two categories. The presence of MAP in clinical specimens such as feces, milk, colostrum, and intestinal tissues can be detected by PCR and bacterial culture (11). Bacterial culture from a fecal sample is the gold standard for diagnosis of JD and the only available method to detect viable MAP. However, observation of visible colonies takes at least 4 weeks after inoculation due to a slow growth rate (11). Furthermore, different disease progression rates, especially in subclinical stages, induce intermittent bacterial shedding and subsequently result in false-negative results for infected animals (11). Detection of MAP by PCR is a fast and high-throughput method that detects MAP-specific genes such as *IS*900, *ISM*ap02, and *f* 57 (12–14). Nevertheless, PCR-based detection has several limitations (15). First, PCR inhibitors in feces may interfere with the reaction and subsequently lead to false-negative results. Second, non-specific amplification from the host and other bacterial DNA can result in false-positive results (15). Third, most subclinically infected animals typically shed bacteria intermittently, which leads to false-negative results (15). Enzyme-linked immunosorbent assay (ELISA) is generally easy to conduct with a simple protocol and high-throughput method for herd-level diagnosis (16). However, diagnosis of MAP-infected cattle at subclinical stage is hampered by the low sensitivity of currently developed commercial ELISA. Furthermore, co-infection with other pathogens such as *Mycobacterium bovis, Fasciola hepatica*, and non-tuberculous mycobacteria may reduce diagnostic specificity by altering the immune response (17, 18). Taken together, alternative diagnostic methods that can detect subclinically infected animals are urgently needed for the control and eradication of JD.

Biomarkers are measurable indicators that represent physiological alterations during disease progression. A number of studies have previously identified potential biomarkers in MAP-infected animals (16, 19, 20). Acute phase proteins such as haptoglobin and serum amyloid A were significantly increased in various stages of paratuberculosis infection with different types of lesions (20). Shaughnessy *et al*. identified three fecal biomarkers (hsa-miR-658, hsa-miR-92a-3p, and hsa-miR-501-5p) that were significantly upregulated in fecal samples of cattle with JD (19). Several proteins, including ABCA13, FAM84A, DES, ABCA13, MMP8, and SPARC, were abundant in serum of MAP-infected animals with different pathological lesions compared to non-infected animals, suggesting their potential for diagnosis of subclinical stages of JD (16). In previous studies, efforts have been made to identify biomarker candidates by analyzing host transcripts in MAP-infected cells, mice and bovine models (21–23), and also by discovering several genes that are upregulated in cattle infected with MAP (24–26). However, mRNA and protein expression of these genes demonstrated poor correlation (27) and previous studies have been conducted with relatively small sample sizes. To overcome these limitations, we analyzed the proteomic profiles of serum samples from MAP-infected cattle in different stages of disease progression for biomarker discovery and evaluated the diagnostic potential of the novel biomarker candidates in naturally MAP-infected cattle.

## Materials and Methods

### Animal Subjects and Sample Collection

Cattle were selected from one farm in Chungcheongnam province and two farms in Gangwon province of South Korea. For proteomic analysis for the serum proteins, 28 cattle were selected and classified into three groups according to the results of fecal qPCR analysis and the level of serum MAP antibodies detected by ELISA as follows: (1) Healthy control group (*n* = 10): selected from a JD-free farm, negative for fecal PCR and serum ELISA. (2) Subclinical shedder group (*n* = 8): fecal PCR positive and ELISA negative. (3) Clinical shedder group (*n* = 10): exhibiting typical JD clinical signs, positive for fecal PCR and ELISA. For the evaluation of biomarker candidates, 126 cattle were selected according to ELISA results for serum samples detected using two commercial ELISA diagnostic kits (IDEXX Laboratories, Inc., Westbrook, ME, USA; ID Screen Paratuberculosis Indirect, ID Vet, Montpellier, France) and detection of MAP in fecal samples by qPCR targeting IS*900* and ISMap*02* (12, 28). In detail, 126 cattle were divided into four groups as follows: (1) Healthy control group (*n* = 11): selected from a JD-free farm, negative for fecal PCR and ELISA negative for both commercial kits. (2) MAP-exposed group (*n* = 20): selected from a JD-positive farm, negative for fecal PCR and ELISA negative for both commercial kits. (3) Subclinical shedder group (*n* = 27): fecal PCR positive and ELISA negative for both commercial kits. (4) Subclinical non-shedder group (*n* = 50): fecal PCR negative and ELISA positive for at least one commercial kit. (5) Clinical shedder group (*n* = 18): exhibiting typical JD clinical signs, fecal PCR positive, and ELISA positive for at least one commercial kit. Blood samples were collected by caudal vein venipuncture with Vacutainer Plus Plastic Serum Tubes (BD Biosciences, San Jose, CA, USA). Serum was separated by centrifugation at 2,500 *g* for 10 min. Separated serum was transferred to a 1.5 mL tube and stored at −80°C until use. The animal study was reviewed and approved by the Animal Ethics Committee of Seoul National University (SNU-200525-4).

### Two-Dimensional Gel Electrophoresis (2-DE) Analysis

For 2-DE analysis for the serum proteins, the pooled serum samples were prepared from three groups. Calbiochem ProteoExtract™ Removal Kits (Merck Millipore, Darmstadt, Germany) was used for the removal of albumin and IgG from pooled serum samples according to the manufacturer's instructions. 2-DE and image analysis with 2-DE samples were conducted as previously described (29). Serum protein samples were washed with 40 mmol/L Tris-hydrochloride (HCl) (pH 7.2) and 1 mmol/L ethylenediaminetetraacetic acid (EDTA) and lysed using a buffer containing 9.5 mol/L urea, 4% 3-((3-cholamidopropyl)dimethylammonium)-1-propanesulfonate (CHAPS), and 35 mmol/L Tris-HCl (pH 7.2). The rehydration solution containing 8 mol/L urea, 4% CHAPS, 10 mmol/L dithiothreitol (DTT), and 0.2% carrier ampholytes (pH 3.0–10.0) was mixed with the solubilized protein samples (30 μg) and applied to immobilized pH gradient (IPG) strips (7 cm; Bio-Rad Laboratories, Hercules, CA, USA) at pH 3.0–10.0 in a re-swelling tray (Bio-Rad). Isoelectric focusing (IEF) was performed using a Protein IEF Cell (Bio-Rad), and 3 preset programs consisting of the first conditioning step (15 min, 250 Vh), the linear voltage ramping step (3 h, 4,000 Vh), and the maximum voltage ramping step (up to 30,000 Vh). After IEF, the strips were equilibrated with 0.375 mol/L Tris buffer (pH 8.8) containing 6 mol/L urea, 2% sodium dodecyl sulfate (SDS), 20% glycerol, 2% DTT, and 0.01% bromophenol blue. The equilibrated strips were equilibrated again with the same buffer supplemented with 2.5% iodoacetamide. 2D SDS-PAGE was conducted overnight with 12.5% separating polyacrylamide gel (8–10 cm) without a stacking gel at 20 mA per gel.

### Image Analysis and In-gel Protein Digestion

Visualization of resolved protein spots on the gels was carried out by silver staining and scanned using a Fluor-S MultiImager (Bio-Rad). PDQUEST 2D Gel Analysis Software version 6 (Bio-Rad) was used to analyze spot intensities of each sample. After silver staining, individual spots were excised from the 2-DE gels and transferred into 1.5 mL tubes. Then, 30 mmol/L potassium ferricyanide and 100 mmol/L sodium thiosulfate were mixed (1:1 ratio) and 100 μL of the mixture was added to the sample and vortexed until the brownish color disappeared. Distilled water was added to the samples 3x to cease the reaction. Then, 500 μL of 200 mmol/L ammonium bicarbonate was added to cover the gel for 20 min. The solution was discarded, and the gel piece was dehydrated with 100 μL acetonitrile, followed by drying using vacuum centrifugation. In-gel digestion was conducted as previously described (30). Briefly, digestion buffer with 12.5 ng/mL trypsin was added to the gel pieces containing protein spots and incubated for 45 min on ice. Next, the enzyme solution was removed and 20 μL of buffer without enzyme was added to maintain hydration during the enzymatic reaction overnight at 37°C. The gel pieces were then subjected to vigorous vortexing for 30 min. The digested solution was transferred into a new 1.5 mL tube and dried using vacuum centrifugation. Finally, the samples were dissolved in 2 μL 0.1% trifluoroacetic acid (TFA).

### Peptide Mass Fingerprinting

For peptide mass fingerprinting, a matrix solution containing α-cyano-4-hydroxycinamic acid (40 mg/mL) in 50% acetonitrile and 0.1% TFA was prepared. An equal volume of matrix solution was added to the sample solution and 2 μL was transferred to the matrix-assisted laser desorption/ionization time of flight (MALDI-TOF)/TOF target plate, quickly dried, and washed with deionized water. After drying for 10 min at room temperature, the mixture solution was subjected to MALDI-TOF-mass spectrometry (MS) and MS/MS analysis using an ABI 4800 Plus TOF-TOF Mass Spectrometer (Applied Biosystems, Framingham, MA, USA). The apparatus was set at 200-Hz Nd:355-nm YAG laser operation for analysis. Peaks with signal/noise ratios >25 were selected, and the 10 most intense ions were used for MS/MS analysis in 1 kV mode and 1,000–1,250 consecutive laser exposures. *Bos taurus* proteins from the National Center for Biotechnology Information (NCBI) protein database (version 20140415; 2,845 sequences, 921,323 residues) were used for analysis of all MALDI-TOF-MS spectra with a molecular mass range of ±15% of that estimated from 2-DE, allowing a peptide mass accuracy of 50 ppm. Protein Pilot V.3.0 database software (with the MASCOT V.2. 3.02 database search engine) was used for analysis of MS/MS spectral data at a mass tolerance of 50 ppm. Individual peptide ion scores were detected using a statistically significant threshold value of *P* = 0.05. The MS proteomic data have been presented in Supplementary Data 1.

### ELISA

For quantitative detection of the three selected biomarkers (alpha-2-macroglobulin [A2M], transthyretin [TTR], and alpha-1-beta glycoprotein [A1BG]) in the serum of each animal, commercially available ELISA kits were used according to the manufacturer's instructions (MyBioSource, San Diego, CA. USA). The detection ranges of A2M, TTR, and A1BG were 0.156–10 μg/mL, 312.5–5,000 ng/mL, and 2.5–50 ng/mL, respectively. The intra and inter assay CV of ELISA kits were <8% and <10%, respectively. A standard curve was generated to determine the concentration of each biomarker in the serum samples.

### Statistical Analysis

ANOVA with Tukey's *post-hoc* test between different infection groups was conducted using GraphPad Prism software version 7.00 (GraphPad Software, Inc., La Jolla, CA, USA). A *P*-value < 0.05 was considered statistically significant. Receiver operating characteristic (ROC) curve analysis was carried out for the determination of the area under the curve (AUC) and optimal cut-off values for each biomarker candidate. ROC curve analysis was performed using MedCalc software version 19.4 (MedCalc Software, Ostend, Belgium). The optimal cut-off values were determined as the value showing the maximum Youden Index (J = Se+Sp-1). The ability to differentiate between different infection groups and the healthy control group was determined with the following meaning. In detail, biomarkers with AUC values ≥0.9 were considered to possess excellent discriminatory power. AUC values ≥0.8 and <0.9 were considered to possess good discriminatory power. AUC values ≥0.7 and <0.8 were considered to possess fair discriminatory power. AUC values <0.7 were considered to have poor discriminatory power (31).

## Results

### Animal Subjects

The characteristics of the 28 animals selected for serum profiling are summarized in Table 1A. The mean ELISA sample/positive (S/P) ratios of the healthy control, subclinical shedder, and clinical shedder groups were 4.16, 7.31, and 235.93, respectively. The mean ages of the cattle between the groups were not significantly different. The characteristics of the 126 animals selected for evaluation of the biomarker candidates are also summarized in Table 1B. The mean IDEXX ELISA S/P ratios for the healthy control, MAP-exposed, subclinical shedder, subclinical non-shedder, and clinical shedder groups were 3.73, 8.58, 5.84, 114.14, and 207.67, respectively. Similarly, the mean IDVET ELISA S/P ratios for these groups were 2.49, 8.01, 2.69, 90.25, and 183.35, respectively. Collectively, the mean ages of the cattle between the groups were not significantly different.

Table 1Clinical characteristics of the animal subjects in this study.

**Table:** 

**(A) Animals for the serum profiling for biomarker discovery**				
		**Healthy control** **(*n* = 10)**	**Subclinical shedder** **(*n* = 8)**	**Clinical shedder** **(*n* = 10)**
Age, years, mean ± SD		2.80 ± 1.31	3.50 ± 1.51	5.42 ± 1.61
Sex, female		10 (100)	8 (100)	10 (100)
MAP isolation		0 (0)	1 (12.5)	8 (80)
Fecal PCR positive		0 (0)	8 (100)	10 (100)
Serum ELISA S/P ratio, mean ± SD	IDEXX	4.06 ± 1.85	7.31 ± 4.15	235.93 ± 27.67

**Table:** 

**(B) Animals for the evaluation of the biomarker candidates**						
		**Healthy control** **(*n* = 11)**	**Exposed** **(*n* = 20)**	**Subclinical shedder** **(*n* = 28)**	**Subclinical non-shedder** **(*n* = 50)**	**Clinical shedder** **(*n* = 18)**
Age, years, mean ± SD		5.69 ± 1.6	4.33 ± 1.6	3.83 ± 2.33	4.73 ± 1.43	5 ± 1.71
Sex, female		11 (100)	20 (100)	28 (100)	50 (100)	18 (100)
MAP isolation		0 (0)	0 (0)	1 (3.6)	0 (0)	14 (77.8)
Fecal PCR positive		0 (0)	0 (0)	28 (100)	0 (0)	18 (100)
Serum ELISA S/P ratio, mean ± SD	IDEXX	3.73 ± 1.82	8.58 ± 11.74	5.84 ± 7.79	114.21 ± 54.14	207.67 ± 102.30
	IDVet	2.49 ± 1.24	8.01 ± 5.59	2.69 ± 2.16	90.25 ± 77.14	183.35 ± 60.99

*Data are presented as numbers (percentages) unless otherwise stated*.

### Protein Identification

**Figure 1:**
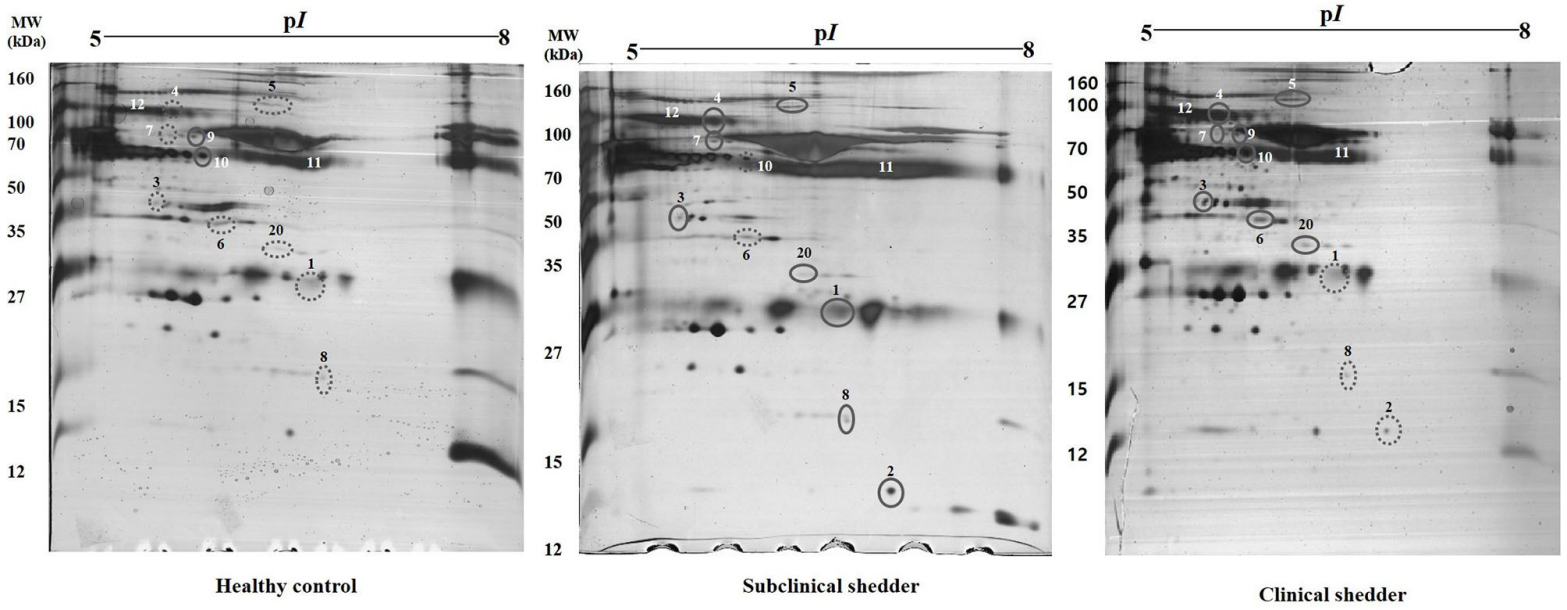
Representative two-dimensional gel electrophoresis of serum proteins from cattle according to MAP infection stages. Results from the healthy control group (left), subclinical shedder (middle) and clinical shedder (right) groups are presented. The pH range is indicated at the top. Circled spots were cut and analyzed by MALDI TOF MS/MS.

In total, 12 significant regions were identified through MALDI-TOF/MS analysis, and a meaningful protein list was prepared (Table 2 and Figure 1). Two proteins (complement C3 and TTR) were abundant only in the subclinical shedder group compared to the healthy control group, while four proteins (complement component 3d, A1BG precursor, complement component C9 precursor, and uncharacterized protein) were abundant only in the clinical shedder group. Furthermore, four proteins (apolipoprotein A-IV, A2M, IgM heavy chain constant region, and Kallikrein G) were abundant in both the subclinical and clinical shedder groups. Identification of the hIgG1 heavy chain constant region or bovine serum albumin suggested the possibility of incomplete removal of immunoglobin and albumin. A2M, A1BG, and TTR were selected for further evaluation of diagnostic performance.

**Table 2:** Identification of differentially expressed serum biomarkers in MAP-infected animals.

**Spot No**.	**Protein Name**	**ID(NCBI)**	**MW** **(Da)**	***pI***	**Expectation** **value**	**Upregulation in group**
1	Complement C3	A0A4W2D411	190190	8.36	3.20E−04	Subclinical shedder
2	Transthyretin	A0A4W2BU20	15831	5.91	2.40E−04	Subclinical shedder
3	Apolipoprotein A-IV	V6F7X3	42963	5.3	6.70E−24	Subclinical and clinical shedder
4	Alpha-2-macroglobulin	R9QSM8	134613	5.75	1.40E−04	Subclinical and clinical shedder
5	IgM heavy chain constant region	2232299	48512	5.68	3.10E+02	Subclinical and clinical shedder
6	Complement component 3d	Q693V9	34593	6.68	1.10E−20	Clinical shedder
7	alpha-1B-glycoprotein precursor	Q2KJF1	54091	5.29	2.40E−05	Clinical shedder
8	Kallikrein G	A0A1R3UGP4	28249	9.07	3.40E−02	Subclinical and clinical shedder
9	complement component C9 precursor	A0A3Q1MU98	58618	5.66	1.10E-08	Clinical shedder
10	Uncharacterized protein	A0A3Q1M3L6	41077	5.16	7.90E−04	Clinical shedder
11	hIgG1 heavy chain constant region	7547266	36510	6.09	2.70E−08	–
12	Inter-alpha-trypsin inhibitor heavy chain	F1MMD7	101620	6.22	5.00E−03	–

### Biomarker ELISA

**Figure 2:**
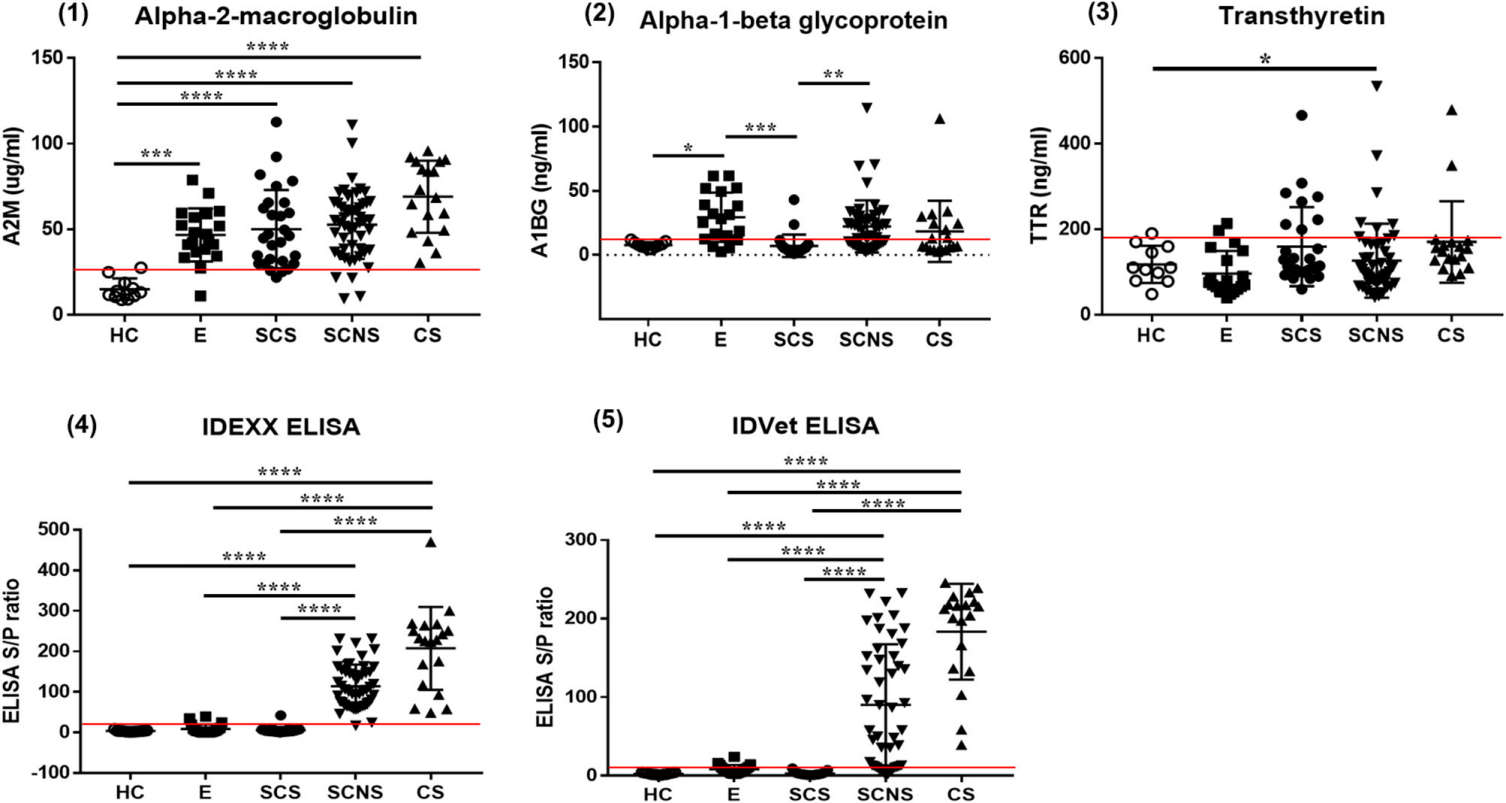
Biomarker expression levels in serum samples of MAP-infected cattle showing different infection status. Serum samples of total 126 cattle were used and grouped in to four as follows: (1) Healthy control group (HC, *n* = 11): selected from a JD-free farm, negative for fecal PCR and ELISA negative for both commercial kits. (2) MAP-exposed group (E, *n* = 20): selected from a JD-positive farm, negative for fecal PCR and ELISA negative for both commercial kits. (3) Subclinical shedder group (SCS, *n* = 27): fecal PCR positive and ELISA negative for both commercial kits. (4) Subclinical non-shedder group (SCNS, *n* = 50): fecal PCR negative and ELISA positive for at least one commercial kit. (5) Clinical shedder group (CS, *n* = 18): fecal PCR positive, and ELISA positive for at least one commercial kit (*p < 0.05; **p < 0.01; ***p < 0.001; ****p < 0.0001). The optimal cut-off values of A2M, A1BG, TTR, IDEXX, and IDVET ELISA are presented as red lines in each graph.

The serum levels of each biomarker candidate within different infection groups based on the results of serum ELISA and fecal PCR are shown in Figure 2. Serum A2M levels were significantly higher in the MAP-exposed (*p* < 0.01), subclinical shedder (*p* < 0.0001), subclinical non-shedder (*p* < 0.0001), and clinical shedder (*p* < 0.0001) groups than in the healthy control group. The serum A1BG level was significantly higher in the MAP-exposed group than in the healthy control group (*p* < 0.05) and significantly lower in the subclinical shedder group than in the MAP-exposed group (*p* < 0.01). Additionally, the subclinical non-shedder group demonstrated a higher serum A1BG level than the subclinical shedder group (*p* < 0.01). The serum TTR level was significantly higher in the subclinical non-shedder group than in the healthy control group (*p* < 0.05), but did not differ significantly among the other infection groups.

### Diagnostic Utility of Selected Biomarkers

**Figure 3:**
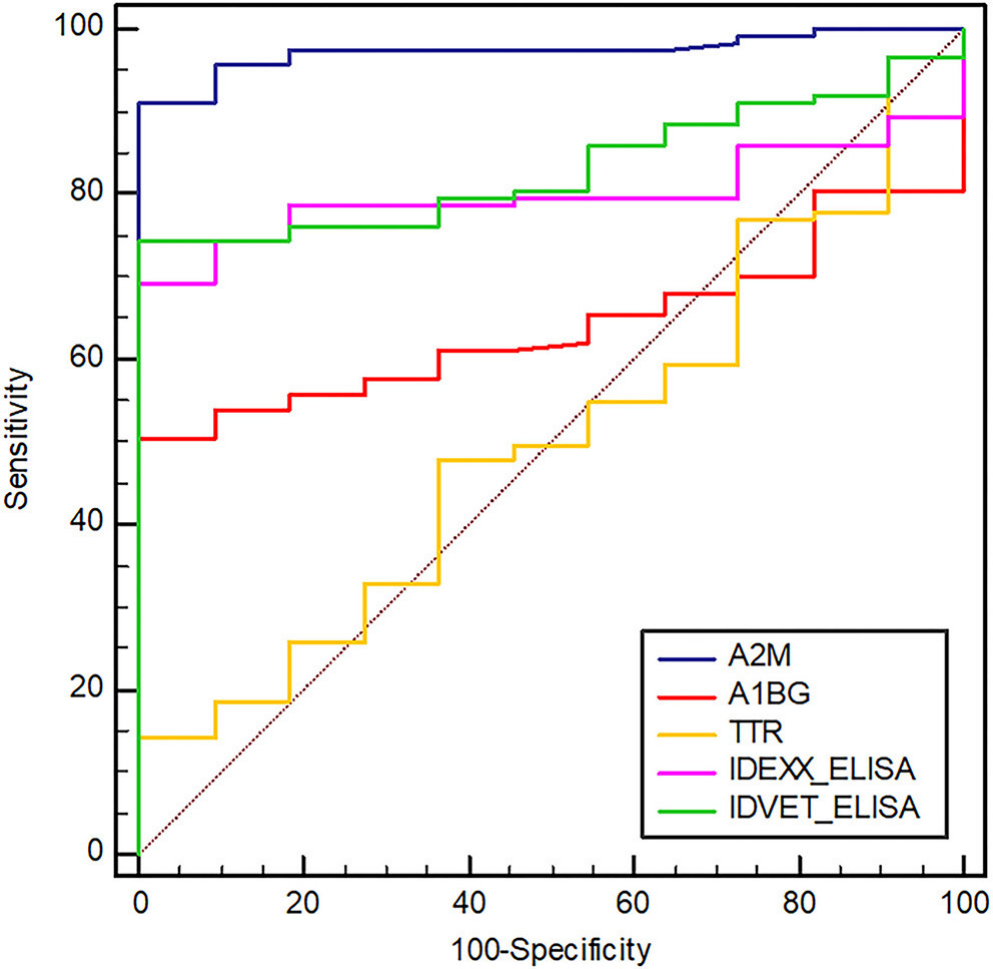
Receiver operating characteristic (ROC) curves of selected biomarkers and two commercial ELISAs (IDEXX and IDVET). A2M, alpha-2-macroglobulin; A1BG, alpha-1-beta glycoprotein; TTR, transthyretin. The area under the ROC of A2M, A1BG, TTR, IDEXX, and IDVET ELISAs is 0.973, 0.641, 0.512, 0.796, and 0.828, respectively. The optimal cut-off values of A2M, A1BG, TTR, IDEXX, and IDVET ELISA are 27.56, 11.93, 190.13, 8.92, and 4.37, respectively.

Compared with the commercial ELISA kits, the diagnostic performance of candidate biomarker proteins was presented according to groups (Table 3). All subjects in groups 1, 2 and 3 corresponding to the clinical shedder were positive in A2M-ELISA. In particular, 25 out of 26 subjects in groups 5 and 6, which were subclinical non-shedder and showed positive only in one of the commercial kits, were diagnosed as positive by A2M-ELISA. Above all, in group 7 belonging to the subclinical shedder and group 8 belonging to the exposed group, 23 out of 27 subjects and 18 out of 20 subjects were diagnosed as positive in the diagnosis using A2M-ELISA, respectively (Table 3). ROC curve analysis suggested the possibility of discrimination between the different infection groups using biomarker-based ELISA. AUCs and their optimal cut-off values were calculated (Figure 3). Comparing the healthy control group (*n* = 11) and the infected cattle group altogether (*n* = 115), A2M ELISA possessed excellent discriminatory power with an AUC = 0.973 (95% confidence interval [CI]: 0.946–1.000, *p* < 0.0001), a sensitivity of 90.4%, and a specificity of 100%. Conversely, A1BG ELISA possessed poor discriminatory power with an AUC = 0.641 (95% CI: 0.543–0.739, *p* = 0.0048), a sensitivity of 50.4%, and a specificity of 100%. Similarly, TTR ELISA demonstrated poor discriminatory ability with an AUC value of 0.512 (95% CI: 0.348–0.677, *p* = 0.8840), a sensitivity of 15.7%, and a specificity of 100%. The IDEXX commercial ELISA kit demonstrated fair discriminatory ability with an AUC value of 0.796 (95% CI: 0.720–0.872, *p* < 0.0001), a sensitivity of 67.83%, and a specificity of 100%. In addition, the IDVET commercial ELISA kit demonstrated good discriminatory ability with an AUC value of 0.828 (95% CI: 0.753–0.904, *p* < 0.0001), a sensitivity of 73.04%, and a specificity of 100%. Comparison of ROC curves obtained using the three biomarkers to those obtained using the two commercial kits revealed that A2M demonstrated superior diagnostic performance (Figure 3).

**Table 3:** Comparison of diagnostic performance using candidate biomarker proteins and commercial ELISA kits.

Group^*^				**A2M**		**A1BG**		**TTR**		**IDEXX ELISA**		**IDVET ELISA**	
	**PCR**	**IDEXX ELISA**	**IDVET ELISA**	**P**	**N**	**P**	**N**	**P**	**N**	**P**	**N**	**P**	**N**
1	P	P	P	15	0	7	8	1	14	15	0	15	0
2	P	P	N	2	0	1	1	0	2	2	0	0	2
3	P	N	P	1	0	0	1	1	0	0	1	1	0
4	N	P	P	21	3	17	7	1	23	24	0	24	0
5	N	P	N	23	1	13	11	3	21	24	0	0	24
6	N	N	P	2	0	1	1	2	0	0	2	2	0
7	P	N	N	23	4	2	25	8	19	0	27	0	27
8	N	N	N	18	2	16	4	2	18	0	20	0	20
9	N	N	N	0	11	0	11	0	11	0	11	0	11

* *Groups 1, 2, and 3 belong to the clinical shedder, groups 4, 5, and 6 to the subclinical non-shedder, and group 7 to the subclinical shedder. In addition, group 8 is occupied to the exposed group, and group 9 to the healthy control group. P, positive; N, negative*.

## Discussion

Detection of protein biomarkers in biological fluids can be a promising method to diagnose and predict disease progression in mycobacterial infections in both animal and human medicine (32). Eradication of JD in the herd can be achieved by detecting subclinical MAP infection, but the current diagnostic tools require improvement. The present study revealed differences in host protein expression within the serum of MAP-infected cattle during different stages of JD progression, suggesting the usefulness of a host biomarker-based diagnostic approach that provides improved sensitivity for subclinical infection.

We discovered 12 protein biomarkers for JD with the serum samples which have different stage of infection. Among the discovered biomarkers, three proteins (A2M, A1BG, and TTR) were selected according to their biological significance. One of the biologically significant protein was A2M. A2M is a large glycoprotein that involved in trapping a broad range of proteases for the host defense (33). A2M possesses available bait region for diverse proteases of pathogen and snares proteases using a cage-like structure through the structural rearrangement process with proteolytic activation (34). Mycobacterial proteases facilitate the invasion of host cells and promote growth during infection, and subsequently play an important role in intracellular survival and disease progression (35). MAP0403 is a transmembrane protein that has 86% amino acid homology to Rv3671c, a serine protease of *M. tuberculosis* (36). The transposon mutant, which has an insertion in Rv3671c, exhibited increased susceptibility to an acidic environment and growth defects in a mouse model (37). Furthermore, a previous study revealed that the essentiality of serine protease MAP0403 for maintaining the intra-bacterial pH and response to acid stress *in vitro* and *in vivo* for intracellular survival (36). Another serine protease, MAP3292c, significantly enhanced intracellular survival and damage to the liver, spleen, and lung in mice (38). Further, MAP3292c facilitated the release of inflammatory cytokines such as IL-1β, IL-6, and TNF-α in mice (38). Although the interaction between A2M and mycobacterial proteases has not yet been identified, the elevated serum A2M level in MAP-infected animals might be in response to the release of MAP serine protease during infection. The interaction between A2M and mycobacterial proteases will be elucidated in further research.

In addition to inhibiting proteases, A2M modulates the biological activity of cytokines and growth factors such as TNF-α, IL-1β, IL-2, IL-6, IL-10, bFGF, β-NGF, PDGF, and TGF-β (39). During the early stage of MAP infection, pro-inflammatory cytokines such as IFN-γ, IL-12, TNF-α and IL-6 are significantly increased in MAP-infected animals compared to healthy control which indicating Th1 dominant immune response (40). On the contrary, immunologic shift arises from Th1 to Th2 with the progression of the disease suggesting the modulation of host immune response by pathogen. Therefore, upregulation of A2M in MAP-infected animals might be related to modulation of the immune response during disease progression. Elevated plasma A2M levels in tuberculous lymphadenitis, pulmonary tuberculosis, and latent tuberculosis patients compared to healthy controls suggested a positive correlation between higher A2M levels and progression of mycobacterial infection (41). Similarly, A2M was also significantly increased in the serum of malnourished patients with active tuberculosis, suggesting a role for A2M in mycobacterial pathogenesis (42). A2M is mainly synthesized in liver and its production is affected by various cytokines such as TNF-α, IL-1 and IL-6 (42). In that regard, up-regulation of A2M can be induced by highly abundant pro-inflammatory cytokines in MAP-infected animal. In our study, A2M levels were significantly higher in MAP-infected animals which including exposed and subclinical cases. Taken together, we propose that A2M may serve as a promising biomarker for differentiation between MAP-infected and non-infected animals.

Other biologically significant proteins were A1BG and TTR. A1BG is a plasma glycoprotein which belongs to immunoglobulin supergene family that containing immunoglobulin like domains (43). TTR is a tetrameric plasma protein which involved in the transport of thyroid hormones and retinol (44). A1BG was significantly elevated only in exposed group compared to healthy control group. Also, elevated serum TTR level was detected in subclinical non-shedder group. However, there are no consistent changes between non-infected and infected cattle suggesting the low diagnostic value of A1BG and TTR in JD. These findings are contrary to previous research which have suggested that up-regulation of serum A1BG in experimentally MAP-infected cattle at 12 months post-infection (PI) compared to *M. bovis*-infected cattle (45). Furthermore, serum A1BG level was also up-regulated in MAP-infected cattle at 3 months PI while decreased in non-infected cattle at same time point (45). In addition, abundance of TTR in serum was increased in experimentally MAP-infected cattle at 3 months PI compared to non-infected cattle and elevated serum TTR level was consistent up to 10 months PI compared to non-infected cattle (45). Differences of A1BG and TTR expression in our study may be elucidated by the origin of sample. In detail, we used serum samples from naturally infected cattle which have over 1 year of age at least while Seth et al. (45) used experimentally infected neonatal calves at 6 weeks of age which leading to different immune response compared to adult cattle.

Our results indicated that A2M ELISA yielded higher AUC value and sensitivity than the two commercial ELISA kits for detection of subclinical MAP-infected animals. Indeed, A2M ELISA demonstrated superior diagnostic performance for detection of MAP-infected animals, including subclinical cases. A2M ELISA detected 85.18% of subclinical shedder animals, while the IDEXX and IDVET ELISA kits detected 0%. Further, A2M ELISA detected 90% of subclinical non-shedder cases, while the IDEXX and IDVET ELISA kits detected 96 and 52%, respectively. Similarly, A2M ELISA detected 100% of clinical shedder animals, while the IDEXX and IDVET ELISA kits detected 94.44 and 88.88%, respectively. Taken together, A2M ELISA improved the detection rate of MAP-infected animals, especially for the subclinical shedder group.

The MAP-exposed animals were negative for fecal PCR and both commercial ELISA kits. However, all exposed animals were raised together with MAP-infected animals, and some exposed animals demonstrated fecal PCR positivity at different time points, suggesting the high possibility of MAP infection within the exposed group. Serum A2M levels in the MAP-exposed and subclinical shedder groups were significantly higher than those in the healthy control group, while this was not observed for serum levels of the other two biomarkers. Furthermore, elevated serum A2M levels were consistent in subclinical shedder, subclinical non-shedder, and clinical shedder groups. Therefore, increased serum A2M levels might be related to the host immune response against MAP infection at early stage.

The current study provides valuable information regarding host serum biomarkers for JD at different infection stages. However, despite rigorous effort, we were unable to collect a significant number of truly healthy control animal samples due to difficulty finding a JD-free farm. Furthermore, the detailed infection status of each animal, such as histological observations and results from cytokine assays and tissue bacterial cultures, would provide more reliable information for statistical analysis.

In conclusion, the urgent need for JD biomarkers to diagnose MAP-exposed and subclinical MAP-infected cattle arises due to the low sensitivity of current diagnostics methods such as commercial ELISA kits and fecal PCR. The identified serum protein A2M may improve the diagnosis of JD, especially in subclinical cases. Furthermore, the significant change in serum A2M levels in infected animals suggests its important role in JD pathogenesis. The current study reveals new possibilities to improve current JD diagnostic tools with further testing of serum biomarkers for accurate detection of subclinical cases, which may help eradicate JD in the herd.

## Data Availability Statement

The original contributions presented in the study are included in the article/Supplementary Material, further inquiries can be directed to the corresponding author.

## Ethics Statement

The animal study was reviewed and approved by In accordance with local legislation, all animal procedures used in this study were approved by the Animal Ethics Committee of Seoul National University (SNU-200525-4). Written informed consent was obtained from the owners for the participation of their animals in this study.

## Author Contributions

H-EP, HSY, and M-KS: conceptualization. H-EP, J-GC, and J-SP: investigation. H-TP and J-IS: resources. DK: software. MJ and H-LK: validation. S-CB and W-KL: data curation. M-KS and H-EP: writing—original draft preparation. HSY and M-KS: funding acquisition. All authors read and approved the final manuscript.

## Conflict of Interest

The authors declare that the research was conducted in the absence of any commercial or financial relationships that could be construed as a potential conflict of interest.

## Supplementary Material

Supplementary Data 1 Mascot search results and raw data files for LC-MS/MS analysis for the identification of specific blood proteins in cattle with Johne's disease.
