# Supplementary material for: Alpha-2-Macroglobulin as a New Promising Biomarker Improving the Diagnostic Sensitivity of Bovine Paratuberculosis
Source: Front Vet Sci. 2021 Mar 5;8:637716. doi: 10.3389/fvets.2021.637716 (PMC7973028; doi:10.3389/fvets.2021.637716)
Supplement: Supplementary Data 1 — Mascot search results and raw data files for LC-MS/MS analysis for the identification of specific blood proteins in cattle with Johne's disease. [file Data_Sheet_1.zip › mascot/1 Complement C3_1.pdf]

PDF-XChange Click to buy NOW! www.docu-track.com

PDF-XChange Click to buy NOW! www.docu-track.com

MATRIX SCIENCE Mascot Search Results

```
User          :
Email         :
Search title  : BAEDW\2020_3_5\2020_3_5\MSMS 14\M17
Database      : Uniprot_bovine bovine_20200316 (134392 sequences; 76244912 residues)
Timestamp     : 31 Mar 2020 at 01:07:59 GMT
Warning       : A Peptide summary report will usually give a much clearer picture of MS/MS search results.
Top Score     : 86 for A0A4W2D411, Complement C3 OS=Bos indicus x Bos taurus OX=30522 GN=C3 PE=4 SV=1
```

Mascot Score Histogram

Protein score is -10*Log(P), where P is the probability that the observed match is a random event.
Protein scores greater than 64 are significant (p<0.05).
Protein scores are derived from ions scores as a non-probabilistic basis for ranking protein hits.

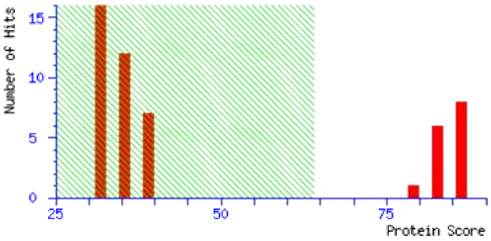

Protein Summary Report

Format As | Protein Summary (deprecated) | Help

Significance threshold p< 0.05 Max. number of hits 30

Preferred taxonomy All entries

Re-Search All | Search Unmatched

Index

	Accession	Mass	Score	Description
1.	A0A4W2D411	190190	86	Complement C3 OS=Bos indicus x Bos taurus OX=30522 GN=C3 PE=4 SV=1
2.	A0A4W2CZQ8	170639	86	Complement C3 OS=Bos indicus x Bos taurus OX=30522 GN=C3 PE=4 SV=1
3.	A0A4W2D441	186349	85	Complement C3 OS=Bos indicus x Bos taurus OX=30522 GN=C3 PE=4 SV=1
4.	A0A4W2CZN6	192486	85	Complement C3 OS=Bos indicus x Bos taurus OX=30522 GN=C3 PE=4 SV=1
5.	A0A4W2EPV7	179159	85	Complement C3 OS=Bos indicus x Bos taurus OX=30522 GN=C3 PE=4 SV=1
6.	A0A4W2D408	180682	85	Complement C3 OS=Bos indicus x Bos taurus OX=30522 GN=C3 PE=4 SV=1
7.	A0A4W2HFJ5	188690	85	Complement C3 OS=Bos indicus x Bos taurus OX=30522 GN=C3 PE=4 SV=1
8.	Q2UVX4	188675	85	Complement C3 OS=Bos taurus OX=9913 GN=C3 PE=1 SV=2
9.	A0A4W2D406	186684	84	Complement C3 OS=Bos indicus x Bos taurus OX=30522 GN=C3 PE=4 SV=1
10.	A0A3Q1MGT0	183008	84	Complement C3 OS=Bos taurus OX=9913 GN=C3 PE=1 SV=1
11.	A0A4W2D3Y9	185555	84	Complement C3 OS=Bos indicus x Bos taurus OX=30522 GN=C3 PE=4 SV=1
12.	A0A3Q1M2B2	185169	83	Complement C3 OS=Bos taurus OX=9913 GN=C3 PE=1 SV=1
13.	A0A0F6QNP7	188604	83	Complement component 3 OS=Bos taurus OX=9913 GN=C3 PE=2 SV=1
14.	A0A4W2EPI5	182918	82	Complement C3 OS=Bos indicus x Bos taurus OX=30522 GN=C3 PE=4 SV=1
15.	A0A3Q1MHV6	181738	81	Complement C3 OS=Bos taurus OX=9913 GN=C3 PE=1 SV=1
16.	A0A4W2FTD2	57549	40	Uncharacterized protein OS=Bos indicus x Bos taurus OX=30522 GN=LOC113883749 PE=3 SV=1
17.	A0A4W2BNQ3	57413	40	Uncharacterized protein OS=Bos indicus x Bos taurus OX=30522 GN=LOC113883746 PE=3 SV=1
18.	A0A4W2HPR9	57408	40	Uncharacterized protein OS=Bos indicus x Bos taurus OX=30522 GN=LOC113883749 PE=3 SV=1
19.	A0A4W2BMU5	57554	40	Uncharacterized protein OS=Bos indicus x Bos taurus OX=30522 GN=LOC113883746 PE=3 SV=1
20.	Q3T047	57540	40	Cytochrome P450, family 3, subfamily A, polypeptide 5 OS=Bos taurus OX=9913 GN=CYP3A5 PE=1 SV=1
21.	A0A3Q1MUB2	54957	39	Glucosaminyl (N-acetyl) transferase 4 OS=Bos taurus OX=9913 GN=GCNT4 PE=4 SV=1
22.	F1MDR5	92495	38	Uncharacterized protein OS=Bos taurus OX=9913 GN=MIA2 PE=4 SV=1
23.	Q08DU7	51251	37	mTERF protein (Fragment) OS=Bos taurus OX=9913 GN=MTERF PE=2 SV=1
24.	A0A4W2FV66	129413	37	Replication factor C subunit 1 OS=Bos indicus x Bos taurus OX=30522 GN=RFC1 PE=3 SV=1
25.	A0A4W2EXE5	129354	36	Replication factor C subunit 1 OS=Bos indicus x Bos taurus OX=30522 GN=RFC1 PE=3 SV=1
26.	A0A4W2BTQ2	97692	36	Kinesin-like protein OS=Bos indicus x Bos taurus OX=30522 GN=KIF9 PE=3 SV=1
27.	A0A0K2YTL5	9022	36	Uncharacterized protein OS=Cowpox virus OX=10243 GN=gCPXV0248 PE=4 SV=1
28.	A0A3Q1N5S1	128941	36	Replication factor C subunit 1 OS=Bos taurus OX=9913 GN=RFC1 PE=3 SV=1
29.	A0A4W2H4H4	50468	35	Interferon induced protein 44 like OS=Bos indicus x Bos taurus OX=30522 GN=IFI44L PE=4 SV=1
30.	A0A4W2BTP7	104127	35	Kinesin-like protein OS=Bos indicus x Bos taurus OX=30522 GN=KIF9 PE=3 SV=1

Results List

1. A0A4W2D411 **Mass:** 190190 **Score:** 86 **Expect:** 0.00032 **Matches:** 18
Complement C3 OS=Bos indicus x Bos taurus OX=30522 GN=C3 PE=4 SV=1

Observed	Mr(expt)	Mr(calc)	ppm	Start	End	Miss	Ions	Peptide
833.4543	832.4470	832.4807	-40.43	868 -	874	0	---	R.LPYSVVR.N
833.4543	832.4471	832.4807	-40.37	868 -	874	0	29	R.LPYSVVR.N
848.4947	847.4874	847.4400	56.0	1453 -	1460	0	---	K.TLSTGVDR.Y
850.5049	849.4976	849.4266	83.6	704 -	710	0	---	R.SVQLMEK.R + Oxidation (M)
864.5046	863.4973	863.4939	3.99	823 -	829	0	---	K.LMNVFLK.D
887.4306	886.4233	886.4508	-31.02	875 -	881	0	---	R.NEQVEIR.A
912.4536	911.4463	911.4865	-44.04	882 -	888	0	---	R.AILYNYR.E
912.4536	911.4463	911.4865	-44.03	882 -	888	0	28	R.AILYNYR.E
996.5558	995.5485	995.4672	81.7	715 -	723	0	---	K.AGQYSSDLR.K
1090.4866	1089.4793	1089.4873	-7.34	655 -	665	0	---	K.ADIGCTPGSGR.N
1164.6057	1163.5984	1163.6411	-36.67	915 -	924	0	---	R.HQQTITIPAR.S
1349.6270	1348.6197	1348.6809	-45.38	743 -	754	0	---	R.AQFILQGDACVK.A

PDF-XChange Click to buy NOW! www.docu-track.com

PDF-XChange Click to buy NOW! www.docu-track.com

1420.6680	1419.6607	1419.7147	-37.99	947 -	959	0	---	K.AAVYNHFISDGVK.K
1427.7352	1426.7279	1426.6625	45.9	521 -	531	1	---	R.YYTYMIMNKGK.L + Oxidation (M)
1796.8849	1795.8776	1795.9178	-22.39	897 -	912	0	---	R.VELLYNPAFCSLATAK.K
1977.9272	1976.9199	1976.9884	-34.62	797 -	812	0	---	R.SQFPESWLWTVIEDLK.Q
2052.0193	2051.0120	2051.0874	-36.73	895 -	912	1	---	K.VRVELLYNPAFCSLATAK.K
2488.1152	2487.1079	2487.1814	-29.56	847 -	867	0	---	K.GICVADPYEVTVMQDFFIDLR.L

No match to: 842.4758, 853.4832, 855.4396, 865.9906, 868.5154, 868.5154, 870.5118, 877.0043, 882.5307, 882.5307, 892.9702, 951.5852, 969.4808, 973.4983, 1017.6315, 1017.6315, 1019.6440, 1033.6599, 1045.5276, 1051.6682, 1051.6682, 1066.4620, 1131.6697, 1151.6833, 1157.5516, 1165.6611, 1165.6611, 1179.5520, 1193.5681, 1277.6617, 1277.6617, 1300.8000, 1307.6257, 1307.6257, 1320.5388, 1334.8374, 1334.8374, 1351.6090, 1365.5970, 1383.6307, 1434.7792, 1448.8667, 1458.6595, 1475.6919, 1487.6974, 1612.7894, 1618.0076, 1618.0076, 1697.8160, 1697.8160, 1707.7140, 1716.7928, 1732.0361, 1838.8502, 1901.1791, 1993.9021, 2211.0298, 2283.1060, 2398.9456

2. A0A4W2CZQ8 **Mass:** 170639 **Score:** 86 **Expect:** 0.00033 **Matches:** 18

Complement C3 OS=Bos indicus x Bos taurus OX=30522 GN=C3 PE=4 SV=1

Observed	Mr(expt)	Mr(calc)	ppm	Start	End	Miss	Ions	Peptide
833.4543	832.4470	832.4807	-40.43	803 -	809	0	---	R.LPYSVVR.N
833.4543	832.4471	832.4807	-40.37	803 -	809	0	29	R.LPYSVVR.N
848.4947	847.4874	847.4400	56.0	1281 -	1288	0	---	K.TLSTGVDR.Y
850.5049	849.4976	849.4266	83.6	639 -	645	0	---	R.SVQLMEK.R + Oxidation (M)
864.5046	863.4973	863.4939	3.99	758 -	764	0	---	K.LMNVFLK.D
887.4306	886.4233	886.4508	-31.02	810 -	816	0	---	R.NEQVEIR.A
912.4536	911.4463	911.4865	-44.04	817 -	823	0	---	R.AILYNYR.E
912.4536	911.4463	911.4865	-44.03	817 -	823	0	28	R.AILYNYR.E
996.5558	995.5485	995.4672	81.7	650 -	658	0	---	K.AGQYSSDLR.K
1090.4866	1089.4793	1089.4873	-7.34	590 -	600	0	---	K.ADIGCTPGSGR.N
1164.6057	1163.5984	1163.6411	-36.67	850 -	859	0	---	R.HQQTITIPAR.S
1349.6270	1348.6197	1348.6809	-45.38	678 -	689	0	---	R.AQFILQGDACVK.A
1420.6680	1419.6607	1419.7147	-37.99	882 -	894	0	---	K.AAVYNHFISDGVK.K
1427.7352	1426.7279	1426.6625	45.9	487 -	497	1	---	R.YYTYMIMNKGK.L + Oxidation (M)
1796.8849	1795.8776	1795.9178	-22.39	832 -	847	0	---	R.VELLYNPAFCSLATAK.K
1977.9272	1976.9199	1976.9884	-34.62	732 -	747	0	---	R.SQFPESWLWTVIEDLK.Q
2052.0193	2051.0120	2051.0874	-36.73	830 -	847	1	---	K.VRVELLYNPAFCSLATAK.K
2488.1152	2487.1079	2487.1814	-29.56	782 -	802	0	---	K.GICVADPYEVTVMQDFFIDLR.L

No match to: 842.4758, 853.4832, 855.4396, 865.9906, 868.5154, 868.5154, 870.5118, 877.0043, 882.5307, 882.5307, 892.9702, 951.5852, 969.4808, 973.4983, 1017.6315, 1017.6315, 1019.6440, 1033.6599, 1045.5276, 1051.6682, 1051.6682, 1066.4620, 1131.6697, 1151.6833, 1157.5516, 1165.6611, 1165.6611, 1179.5520, 1193.5681, 1277.6617, 1277.6617, 1300.8000, 1307.6257, 1307.6257, 1320.5388, 1334.8374, 1334.8374, 1351.6090, 1365.5970, 1383.6307, 1434.7792, 1448.8667, 1458.6595, 1475.6919, 1487.6974, 1612.7894, 1618.0076, 1618.0076, 1697.8160, 1697.8160, 1707.7140, 1716.7928, 1732.0361, 1838.8502, 1901.1791, 1993.9021, 2211.0298, 2283.1060, 2398.9456

3. A0A4W2D441 **Mass:** 186349 **Score:** 85 **Expect:** 0.00039 **Matches:** 18

Complement C3 OS=Bos indicus x Bos taurus OX=30522 GN=C3 PE=4 SV=1

Observed	Mr(expt)	Mr(calc)	ppm	Start	End	Miss	Ions	Peptide
833.4543	832.4470	832.4807	-40.43	834 -	840	0	---	R.LPYSVVR.N
833.4543	832.4471	832.4807	-40.37	834 -	840	0	29	R.LPYSVVR.N
848.4947	847.4874	847.4400	56.0	1419 -	1426	0	---	K.TLSTGVDR.Y
850.5049	849.4976	849.4266	83.6	670 -	676	0	---	R.SVQLMEK.R + Oxidation (M)
864.5046	863.4973	863.4939	3.99	789 -	795	0	---	K.LMNVFLK.D
887.4306	886.4233	886.4508	-31.02	841 -	847	0	---	R.NEQVEIR.A
912.4536	911.4463	911.4865	-44.04	848 -	854	0	---	R.AILYNYR.E
912.4536	911.4463	911.4865	-44.03	848 -	854	0	28	R.AILYNYR.E
996.5558	995.5485	995.4672	81.7	681 -	689	0	---	K.AGQYSSDLR.K
1090.4866	1089.4793	1089.4873	-7.34	621 -	631	0	---	K.ADIGCTPGSGR.N
1164.6057	1163.5984	1163.6411	-36.67	881 -	890	0	---	R.HQQTITIPAR.S
1349.6270	1348.6197	1348.6809	-45.38	709 -	720	0	---	R.AQFILQGDACVK.A
1420.6680	1419.6607	1419.7147	-37.99	913 -	925	0	---	K.AAVYNHFISDGVK.K
1427.7352	1426.7279	1426.6625	45.9	487 -	497	1	---	R.YYTYMIMNKGK.L + Oxidation (M)
1796.8849	1795.8776	1795.9178	-22.39	863 -	878	0	---	R.VELLYNPAFCSLATAK.K
1977.9272	1976.9199	1976.9884	-34.62	763 -	778	0	---	R.SQFPESWLWTVIEDLK.Q
2052.0193	2051.0120	2051.0874	-36.73	861 -	878	1	---	K.VRVELLYNPAFCSLATAK.K
2488.1152	2487.1079	2487.1814	-29.56	813 -	833	0	---	K.GICVADPYEVTVMQDFFIDLR.L

No match to: 842.4758, 853.4832, 855.4396, 865.9906, 868.5154, 868.5154, 870.5118, 877.0043, 882.5307, 882.5307, 892.9702, 951.5852, 969.4808, 973.4983, 1017.6315, 1017.6315, 1019.6440, 1033.6599, 1045.5276, 1051.6682, 1051.6682, 1066.4620, 1131.6697, 1151.6833, 1157.5516, 1165.6611, 1165.6611, 1179.5520, 1193.5681, 1277.6617, 1277.6617, 1300.8000, 1307.6257, 1307.6257, 1320.5388, 1334.8374, 1334.8374, 1351.6090, 1365.5970, 1383.6307, 1434.7792, 1448.8667, 1458.6595, 1475.6919, 1487.6974, 1612.7894, 1618.0076, 1618.0076, 1697.8160, 1697.8160, 1707.7140, 1716.7928, 1732.0361, 1838.8502, 1901.1791, 1993.9021, 2211.0298, 2283.1060, 2398.9456

4. A0A4W2CZN6 **Mass:** 192486 **Score:** 85 **Expect:** 0.00039 **Matches:** 18

Complement C3 OS=Bos indicus x Bos taurus OX=30522 GN=C3 PE=4 SV=1

Observed	Mr(expt)	Mr(calc)	ppm	Start	End	Miss	Ions	Peptide
833.4543	832.4470	832.4807	-40.43	868 -	874	0	---	R.LPYSVVR.N
833.4543	832.4471	832.4807	-40.37	868 -	874	0	29	R.LPYSVVR.N
848.4947	847.4874	847.4400	56.0	1453 -	1460	0	---	K.TLSTGVDR.Y
850.5049	849.4976	849.4266	83.6	704 -	710	0	---	R.SVQLMEK.R + Oxidation (M)
864.5046	863.4973	863.4939	3.99	823 -	829	0	---	K.LMNVFLK.D
887.4306	886.4233	886.4508	-31.02	875 -	881	0	---	R.NEQVEIR.A
912.4536	911.4463	911.4865	-44.04	882 -	888	0	---	R.AILYNYR.E
912.4536	911.4463	911.4865	-44.03	882 -	888	0	28	R.AILYNYR.E
996.5558	995.5485	995.4672	81.7	715 -	723	0	---	K.AGQYSSDLR.K
1090.4866	1089.4793	1089.4873	-7.34	655 -	665	0	---	K.ADIGCTPGSGR.N
1164.6057	1163.5984	1163.6411	-36.67	915 -	924	0	---	R.HQQTITIPAR.S
1349.6270	1348.6197	1348.6809	-45.38	743 -	754	0	---	R.AQFILQGDACVK.A
1420.6680	1419.6607	1419.7147	-37.99	947 -	959	0	---	K.AAVYNHFISDGVK.K
1427.7352	1426.7279	1426.6625	45.9	521 -	531	1	---	R.YYTYMIMNKGK.L + Oxidation (M)
1796.8849	1795.8776	1795.9178	-22.39	897 -	912	0	---	R.VELLYNPAFCSLATAK.K
1977.9272	1976.9199	1976.9884	-34.62	797 -	812	0	---	R.SQFPESWLWTVIEDLK.Q
2052.0193	2051.0120	2051.0874	-36.73	895 -	912	1	---	K.VRVELLYNPAFCSLATAK.K
2488.1152	2487.1079	2487.1814	-29.56	847 -	867	0	---	K.GICVADPYEVTVMQDFFIDLR.L

PDF-XChange Click to buy NOW! www.docu-track.com

No match to: 842.4758, 853.4832, 855.4396, 865.9906, 868.5154, 868.5154, 870.5118, 877.0043, 882.5307, 882.5307, 892.9702, 951.5852, 969.4808, 973.4983, 1017.6315, 1017.6315, 1019.6440, 1033.6599, 1045.5276, 1051.6682, 1051.6682, 1066.4620, 1131.6697, 1151.6833, 1157.5516, 1165.6611, 1165.6611, 1179.5520, 1193.5681, 1277.6617, 1277.6617, 1300.8000, 1307.6257, 1307.6257, 1320.5388, 1334.8374, 1334.8374, 1351.6090, 1365.5970, 1383.6307, 1434.7792, 1448.8667, 1458.6595, 1475.6919, 1487.6974, 1612.7894, 1618.0076, 1618.0076, 1697.8160, 1697.8160, 1707.7140, 1716.7928, 1732.0361, 1838.8502, 1901.1791, 1993.9021, 2211.0298, 2283.1060, 2398.9456

5. A0A4W2EPV7 **Mass:** 179159 **Score:** 85 **Expect:** 0.00041 **Matches:** 18

Complement C3 OS=Bos indicus x Bos taurus OX=30522 GN=C3 PE=4 SV=1

Observed	Mr(expt)	Mr(calc)	ppm	Start		End	Miss	Ions	Peptide
833.4543	832.4470	832.4807	-40.43	803	-	809	0	---	R.LPYSVVR.N
833.4543	832.4471	832.4807	-40.37	803	-	809	0	29	R.LPYSVVR.N
848.4947	847.4874	847.4400	56.0	1355	-	1362	0	---	K.TLSTGVDR.Y
850.5049	849.4976	849.4266	83.6	639	-	645	0	---	R.SVQLMEK.R + Oxidation (M)
864.5046	863.4973	863.4939	3.99	758	-	764	0	---	K.LMNVFLK.D
887.4306	886.4233	886.4508	-31.02	810	-	816	0	---	R.NEQVEIR.A
912.4536	911.4463	911.4865	-44.04	817	-	823	0	---	R.AILYNYR.E
912.4536	911.4463	911.4865	-44.03	817	-	823	0	28	R.AILYNYR.E
996.5558	995.5485	995.4672	81.7	650	-	658	0	---	K.AGQYSSDLR.K
1090.4866	1089.4793	1089.4873	-7.34	590	-	600	0	---	K.ADIGCTPGSGR.N
1164.6057	1163.5984	1163.6411	-36.67	850	-	859	0	---	R.HQQTITIPAR.S
1349.6270	1348.6197	1348.6809	-45.38	678	-	689	0	---	R.AQFILQGDACVK.A
1420.6680	1419.6607	1419.7147	-37.99	882	-	894	0	---	K.AAVYNHFISDGVK.K
1427.7352	1426.7279	1426.6625	45.9	487	-	497	1	---	R.YYTYMIMNKGK.L + Oxidation (M)
1796.8849	1795.8776	1795.9178	-22.39	832	-	847	0	---	R.VELLYNPAFCSLATAK.K
1977.9272	1976.9199	1976.9884	-34.62	732	-	747	0	---	R.SQFPESWLWTVIEDLK.Q
2052.0193	2051.0120	2051.0874	-36.73	830	-	847	1	---	K.VRVELLYNPAFCSLATAK.K
2488.1152	2487.1079	2487.1814	-29.56	782	-	802	0	---	K.GICVADPYEVTVMQDFFIDLR.L

No match to: 842.4758, 853.4832, 855.4396, 865.9906, 868.5154, 868.5154, 870.5118, 877.0043, 882.5307, 882.5307, 892.9702, 951.5852, 969.4808, 973.4983, 1017.6315, 1017.6315, 1019.6440, 1033.6599, 1045.5276, 1051.6682, 1051.6682, 1066.4620, 1131.6697, 1151.6833, 1157.5516, 1165.6611, 1165.6611, 1179.5520, 1193.5681, 1277.6617, 1277.6617, 1300.8000, 1307.6257, 1307.6257, 1320.5388, 1334.8374, 1334.8374, 1351.6090, 1365.5970, 1383.6307, 1434.7792, 1448.8667, 1458.6595, 1475.6919, 1487.6974, 1612.7894, 1618.0076, 1618.0076, 1697.8160, 1697.8160, 1707.7140, 1716.7928, 1732.0361, 1838.8502, 1901.1791, 1993.9021, 2211.0298, 2283.1060, 2398.9456

6. A0A4W2D408 **Mass:** 180682 **Score:** 85 **Expect:** 0.00046 **Matches:** 18

Complement C3 OS=Bos indicus x Bos taurus OX=30522 GN=C3 PE=4 SV=1

Observed	Mr(expt)	Mr(calc)	ppm	Start		End	Miss	Ions	Peptide
833.4543	832.4470	832.4807	-40.43	803	-	809	0	---	R.LPYSVVR.N
833.4543	832.4471	832.4807	-40.37	803	-	809	0	29	R.LPYSVVR.N
848.4947	847.4874	847.4400	56.0	1368	-	1375	0	---	K.TLSTGVDR.Y
850.5049	849.4976	849.4266	83.6	639	-	645	0	---	R.SVQLMEK.R + Oxidation (M)
864.5046	863.4973	863.4939	3.99	758	-	764	0	---	K.LMNVFLK.D
887.4306	886.4233	886.4508	-31.02	810	-	816	0	---	R.NEQVEIR.A
912.4536	911.4463	911.4865	-44.04	817	-	823	0	---	R.AILYNYR.E
912.4536	911.4463	911.4865	-44.03	817	-	823	0	28	R.AILYNYR.E
996.5558	995.5485	995.4672	81.7	650	-	658	0	---	K.AGQYSSDLR.K
1090.4866	1089.4793	1089.4873	-7.34	590	-	600	0	---	K.ADIGCTPGSGR.N
1164.6057	1163.5984	1163.6411	-36.67	850	-	859	0	---	R.HQQTITIPAR.S
1349.6270	1348.6197	1348.6809	-45.38	678	-	689	0	---	R.AQFILQGDACVK.A
1420.6680	1419.6607	1419.7147	-37.99	882	-	894	0	---	K.AAVYNHFISDGVK.K
1427.7352	1426.7279	1426.6625	45.9	487	-	497	1	---	R.YYTYMIMNKGK.L + Oxidation (M)
1796.8849	1795.8776	1795.9178	-22.39	832	-	847	0	---	R.VELLYNPAFCSLATAK.K
1977.9272	1976.9199	1976.9884	-34.62	732	-	747	0	---	R.SQFPESWLWTVIEDLK.Q
2052.0193	2051.0120	2051.0874	-36.73	830	-	847	1	---	K.VRVELLYNPAFCSLATAK.K
2488.1152	2487.1079	2487.1814	-29.56	782	-	802	0	---	K.GICVADPYEVTVMQDFFIDLR.L

No match to: 842.4758, 853.4832, 855.4396, 865.9906, 868.5154, 868.5154, 870.5118, 877.0043, 882.5307, 882.5307, 892.9702, 951.5852, 969.4808, 973.4983, 1017.6315, 1017.6315, 1019.6440, 1033.6599, 1045.5276, 1051.6682, 1051.6682, 1066.4620, 1131.6697, 1151.6833, 1157.5516, 1165.6611, 1165.6611, 1179.5520, 1193.5681, 1277.6617, 1277.6617, 1300.8000, 1307.6257, 1307.6257, 1320.5388, 1334.8374, 1334.8374, 1351.6090, 1365.5970, 1383.6307, 1434.7792, 1448.8667, 1458.6595, 1475.6919, 1487.6974, 1612.7894, 1618.0076, 1618.0076, 1697.8160, 1697.8160, 1707.7140, 1716.7928, 1732.0361, 1838.8502, 1901.1791, 1993.9021, 2211.0298, 2283.1060, 2398.9456

7. A0A4W2HFJ5 **Mass:** 188690 **Score:** 85 **Expect:** 0.00047 **Matches:** 18

Complement C3 OS=Bos indicus x Bos taurus OX=30522 GN=C3 PE=4 SV=1

Observed	Mr(expt)	Mr(calc)	ppm	Start		End	Miss	Ions	Peptide
833.4543	832.4470	832.4807	-40.43	834	-	840	0	---	R.LPYSVVR.N
833.4543	832.4471	832.4807	-40.37	834	-	840	0	29	R.LPYSVVR.N
848.4947	847.4874	847.4400	56.0	1419	-	1426	0	---	K.TLSTGVDR.Y
850.5049	849.4976	849.4266	83.6	670	-	676	0	---	R.SVQLMEK.R + Oxidation (M)
864.5046	863.4973	863.4939	3.99	789	-	795	0	---	K.LMNVFLK.D
887.4306	886.4233	886.4508	-31.02	841	-	847	0	---	R.NEQVEIR.A
912.4536	911.4463	911.4865	-44.04	848	-	854	0	---	R.AILYNYR.E
912.4536	911.4463	911.4865	-44.03	848	-	854	0	28	R.AILYNYR.E
996.5558	995.5485	995.4672	81.7	681	-	689	0	---	K.AGQYSSDLR.K
1090.4866	1089.4793	1089.4873	-7.34	621	-	631	0	---	K.ADIGCTPGSGR.N
1164.6057	1163.5984	1163.6411	-36.67	881	-	890	0	---	R.HQQTITIPAR.S
1349.6270	1348.6197	1348.6809	-45.38	709	-	720	0	---	R.AQFILQGDACVK.A
1420.6680	1419.6607	1419.7147	-37.99	913	-	925	0	---	K.AAVYNHFISDGVK.K
1427.7352	1426.7279	1426.6625	45.9	487	-	497	1	---	R.YYTYMIMNKGK.L + Oxidation (M)
1796.8849	1795.8776	1795.9178	-22.39	863	-	878	0	---	R.VELLYNPAFCSLATAK.K
1977.9272	1976.9199	1976.9884	-34.62	763	-	778	0	---	R.SQFPESWLWTVIEDLK.Q
2052.0193	2051.0120	2051.0874	-36.73	861	-	878	1	---	K.VRVELLYNPAFCSLATAK.K
2488.1152	2487.1079	2487.1814	-29.56	813	-	833	0	---	K.GICVADPYEVTVMQDFFIDLR.L

No match to: 842.4758, 853.4832, 855.4396, 865.9906, 868.5154, 868.5154, 870.5118, 877.0043, 882.5307, 882.5307, 892.9702, 951.5852, 969.4808, 973.4983, 1017.6315, 1017.6315, 1019.6440, 1033.6599, 1045.5276, 1051.6682, 1051.6682, 1066.4620, 1131.6697, 1151.6833, 1157.5516, 1165.6611, 1165.6611, 1179.5520, 1193.5681, 1277.6617, 1277.6617, 1300.8000, 1307.6257, 1307.6257, 1320.5388, 1334.8374, 1334.8374, 1351.6090, 1365.5970, 1383.6307, 1434.7792, 1448.8667, 1458.6595, 1475.6919, 1487.6974, 1612.7894, 1618.0076, 1618.0076, 1697.8160, 1697.8160, 1707.7140, 1716.7928, 1732.0361, 1838.8502, 1901.1791, 1993.9021, 2211.0298, 2283.1060, 2398.9456

PDF-XChange Click to buy NOW! www.docu-track.com

PDF-XChange Click to buy NOW! www.docu-track.com

8. Q2UVX4 **Mass:** 188675 **Score:** 85 **Expect:** 0.00047 **Matches:** 18

Complement C3 OS=Bos taurus OX=9913 GN=C3 PE=1 SV=2

Observed	Mr(expt)	Mr(calc)	ppm	Start	End	Miss	Ions	Peptide
833.4543	832.4470	832.4807	-40.43	834 -	840	0	---	R.LPYSVVR.N
833.4543	832.4471	832.4807	-40.37	834 -	840	0	29	R.LPYSVVR.N
848.4947	847.4874	847.4400	56.0	1419 -	1426	0	---	K.TLSTGVDR.Y
850.5049	849.4976	849.4266	83.6	670 -	676	0	---	R.SVQLMEK.R + Oxidation (M)
864.5046	863.4973	863.4939	3.99	789 -	795	0	---	K.LMNVFLK.D
887.4306	886.4233	886.4508	-31.02	841 -	847	0	---	R.NEQVEIR.A
912.4536	911.4463	911.4865	-44.04	848 -	854	0	---	R.AILYNYR.E
912.4536	911.4463	911.4865	-44.03	848 -	854	0	28	R.AILYNYR.E
996.5558	995.5485	995.4672	81.7	681 -	689	0	---	K.AGQYSSDLR.K
1090.4866	1089.4793	1089.4873	-7.34	621 -	631	0	---	K.ADIGCTPGSGR.N
1164.6057	1163.5984	1163.6411	-36.67	881 -	890	0	---	R.HQQTITIPAR.S
1349.6270	1348.6197	1348.6809	-45.38	709 -	720	0	---	R.AQFILQGDACVK.A
1420.6680	1419.6607	1419.7147	-37.99	913 -	925	0	---	K.AAVYNHFISDGVK.K
1427.7352	1426.7279	1426.6625	45.9	487 -	497	1	---	R.YYTYMIMNKGK.L + Oxidation (M)
1796.8849	1795.8776	1795.9178	-22.39	863 -	878	0	---	R.VELLYNPAFCSLATAK.K
1977.9272	1976.9199	1976.9884	-34.62	763 -	778	0	---	R.SQFPESWLWTVIEDLK.Q
2052.0193	2051.0120	2051.0874	-36.73	861 -	878	1	---	K.VRVELLYNPAFCSLATAK.K
2488.1152	2487.1079	2487.1814	-29.56	813 -	833	0	---	K.GICVADPYEVTVMQDFFIDLR.L

No match to: 842.4758, 853.4832, 855.4396, 865.9906, 868.5154, 868.5154, 870.5118, 877.0043, 882.5307, 882.5307, 892.9702, 951.5852, 969.4808, 973.4983, 1017.6315, 1017.6315, 1019.6440, 1033.6599, 1045.5276, 1051.6682, 1051.6682, 1066.4620, 1131.6697, 1151.6833, 1157.5516, 1165.6611, 1165.6611, 1179.5520, 1193.5681, 1277.6617, 1277.6617, 1300.8000, 1307.6257, 1307.6257, 1320.5388, 1334.8374, 1334.8374, 1351.6090, 1365.5970, 1383.6307, 1434.7792, 1448.8667, 1458.6595, 1475.6919, 1487.6974, 1612.7894, 1618.0076, 1618.0076, 1697.8160, 1697.8160, 1707.7140, 1716.7928, 1732.0361, 1838.8502, 1901.1791, 1993.9021, 2211.0298, 2283.1060, 2398.9456

9. A0A4W2D406 **Mass:** 186684 **Score:** 84 **Expect:** 0.00056 **Matches:** 18

Complement C3 OS=Bos indicus x Bos taurus OX=30522 GN=C3 PE=4 SV=1

Observed	Mr(expt)	Mr(calc)	ppm	Start	End	Miss	Ions	Peptide
833.4543	832.4470	832.4807	-40.43	837 -	843	0	---	R.LPYSVVR.N
833.4543	832.4471	832.4807	-40.37	837 -	843	0	29	R.LPYSVVR.N
848.4947	847.4874	847.4400	56.0	1422 -	1429	0	---	K.TLSTGVDR.Y
850.5049	849.4976	849.4266	83.6	673 -	679	0	---	R.SVQLMEK.R + Oxidation (M)
864.5046	863.4973	863.4939	3.99	792 -	798	0	---	K.LMNVFLK.D
887.4306	886.4233	886.4508	-31.02	844 -	850	0	---	R.NEQVEIR.A
912.4536	911.4463	911.4865	-44.04	851 -	857	0	---	R.AILYNYR.E
912.4536	911.4463	911.4865	-44.03	851 -	857	0	28	R.AILYNYR.E
996.5558	995.5485	995.4672	81.7	684 -	692	0	---	K.AGQYSSDLR.K
1090.4866	1089.4793	1089.4873	-7.34	624 -	634	0	---	K.ADIGCTPGSGR.N
1164.6057	1163.5984	1163.6411	-36.67	884 -	893	0	---	R.HQQTITIPAR.S
1349.6270	1348.6197	1348.6809	-45.38	712 -	723	0	---	R.AQFILQGDACVK.A
1420.6680	1419.6607	1419.7147	-37.99	916 -	928	0	---	K.AAVYNHFISDGVK.K
1427.7352	1426.7279	1426.6625	45.9	521 -	531	1	---	R.YYTYMIMNKGK.L + Oxidation (M)
1796.8849	1795.8776	1795.9178	-22.39	866 -	881	0	---	R.VELLYNPAFCSLATAK.K
1977.9272	1976.9199	1976.9884	-34.62	766 -	781	0	---	R.SQFPESWLWTVIEDLK.Q
2052.0193	2051.0120	2051.0874	-36.73	864 -	881	1	---	K.VRVELLYNPAFCSLATAK.K
2488.1152	2487.1079	2487.1814	-29.56	816 -	836	0	---	K.GICVADPYEVTVMQDFFIDLR.L

No match to: 842.4758, 853.4832, 855.4396, 865.9906, 868.5154, 868.5154, 870.5118, 877.0043, 882.5307, 882.5307, 892.9702, 951.5852, 969.4808, 973.4983, 1017.6315, 1017.6315, 1019.6440, 1033.6599, 1045.5276, 1051.6682, 1051.6682, 1066.4620, 1131.6697, 1151.6833, 1157.5516, 1165.6611, 1165.6611, 1179.5520, 1193.5681, 1277.6617, 1277.6617, 1300.8000, 1307.6257, 1307.6257, 1320.5388, 1334.8374, 1334.8374, 1351.6090, 1365.5970, 1383.6307, 1434.7792, 1448.8667, 1458.6595, 1475.6919, 1487.6974, 1612.7894, 1618.0076, 1618.0076, 1697.8160, 1697.8160, 1707.7140, 1716.7928, 1732.0361, 1838.8502, 1901.1791, 1993.9021, 2211.0298, 2283.1060, 2398.9456

10. A0A3Q1MGT0 **Mass:** 183008 **Score:** 84 **Expect:** 0.00056 **Matches:** 18

Complement C3 OS=Bos taurus OX=9913 GN=C3 PE=1 SV=1

Observed	Mr(expt)	Mr(calc)	ppm	Start	End	Miss	Ions	Peptide
833.4543	832.4470	832.4807	-40.43	803 -	809	0	---	R.LPYSVVR.N
833.4543	832.4471	832.4807	-40.37	803 -	809	0	29	R.LPYSVVR.N
848.4947	847.4874	847.4400	56.0	1368 -	1375	0	---	K.TLSTGVDR.Y
850.5049	849.4976	849.4266	83.6	639 -	645	0	---	R.SVQLMEK.R + Oxidation (M)
864.5046	863.4973	863.4939	3.99	758 -	764	0	---	K.LMNVFLK.D
887.4306	886.4233	886.4508	-31.02	810 -	816	0	---	R.NEQVEIR.A
912.4536	911.4463	911.4865	-44.04	817 -	823	0	---	R.AILYNYR.E
912.4536	911.4463	911.4865	-44.03	817 -	823	0	28	R.AILYNYR.E
996.5558	995.5485	995.4672	81.7	650 -	658	0	---	K.AGQYSSDLR.K
1090.4866	1089.4793	1089.4873	-7.34	590 -	600	0	---	K.ADIGCTPGSGR.N
1164.6057	1163.5984	1163.6411	-36.67	850 -	859	0	---	R.HQQTITIPAR.S
1349.6270	1348.6197	1348.6809	-45.38	678 -	689	0	---	R.AQFILQGDACVK.A
1420.6680	1419.6607	1419.7147	-37.99	882 -	894	0	---	K.AAVYNHFISDGVK.K
1427.7352	1426.7279	1426.6625	45.9	487 -	497	1	---	R.YYTYMIMNKGK.L + Oxidation (M)
1796.8849	1795.8776	1795.9178	-22.39	832 -	847	0	---	R.VELLYNPAFCSLATAK.K
1977.9272	1976.9199	1976.9884	-34.62	732 -	747	0	---	R.SQFPESWLWTVIEDLK.Q
2052.0193	2051.0120	2051.0874	-36.73	830 -	847	1	---	K.VRVELLYNPAFCSLATAK.K
2488.1152	2487.1079	2487.1814	-29.56	782 -	802	0	---	K.GICVADPYEVTVMQDFFIDLR.L

No match to: 842.4758, 853.4832, 855.4396, 865.9906, 868.5154, 868.5154, 870.5118, 877.0043, 882.5307, 882.5307, 892.9702, 951.5852, 969.4808, 973.4983, 1017.6315, 1017.6315, 1019.6440, 1033.6599, 1045.5276, 1051.6682, 1051.6682, 1066.4620, 1131.6697, 1151.6833, 1157.5516, 1165.6611, 1165.6611, 1179.5520, 1193.5681, 1277.6617, 1277.6617, 1300.8000, 1307.6257, 1307.6257, 1320.5388, 1334.8374, 1334.8374, 1351.6090, 1365.5970, 1383.6307, 1434.7792, 1448.8667, 1458.6595, 1475.6919, 1487.6974, 1612.7894, 1618.0076, 1618.0076, 1697.8160, 1697.8160, 1707.7140, 1716.7928, 1732.0361, 1838.8502, 1901.1791, 1993.9021, 2211.0298, 2283.1060, 2398.9456

11. A0A4W2D3Y9 **Mass:** 185555 **Score:** 84 **Expect:** 0.0006 **Matches:** 18

Complement C3 OS=Bos indicus x Bos taurus OX=30522 GN=C3 PE=4 SV=1

Observed	Mr(expt)	Mr(calc)	ppm	Start	End	Miss	Ions	Peptide
833.4543	832.4470	832.4807	-40.43	837 -	843	0	---	R.LPYSVVR.N
833.4543	832.4471	832.4807	-40.37	837 -	843	0	29	R.LPYSVVR.N

PDF-XChange Click to buy NOW! www.docu-track.com
PDF-XChange Click to buy NOW! www.docu-track.com

848.4947	847.4874	847.4400	56.0	1422	1429	0	---	K.TLSTGVDR.Y
850.5049	849.4976	849.4266	83.6	673	679	0	---	R.SVQLMEK.R + Oxidation (M)
864.5046	863.4973	863.4939	3.99	792	798	0	---	K.LMNVFLK.D
887.4306	886.4233	886.4508	-31.02	844	850	0	---	R.NEQVEIR.A
912.4536	911.4463	911.4865	-44.04	851	857	0	---	R.AILYNYR.E
912.4536	911.4463	911.4865	-44.03	851	857	0	28	R.AILYNYR.E
996.5558	995.5485	995.4672	81.7	684	692	0	---	K.AGQYSSDLR.K
1090.4866	1089.4793	1089.4873	-7.34	624	634	0	---	K.ADIGCTPGSGR.N
1164.6057	1163.5984	1163.6411	-36.67	884	893	0	---	R.HQQTITIPAR.S
1349.6270	1348.6197	1348.6809	-45.38	712	723	0	---	R.AQFILQGDACVK.A
1420.6680	1419.6607	1419.7147	-37.99	916	928	0	---	K.AAVYNHFISDGVK.K
1427.7352	1426.7279	1426.6625	45.9	521	531	1	---	R.YYTYMIMNKGK.L + Oxidation (M)
1796.8849	1795.8776	1795.9178	-22.39	866	881	0	---	R.VELLYNPAFCSLATAK.K
1977.9272	1976.9199	1976.9884	-34.62	766	781	0	---	R.SQFPESWLWTVIEDLK.Q
2052.0193	2051.0120	2051.0874	-36.73	864	881	1	---	K.VRVELLYNPAFCSLATAK.K
2488.1152	2487.1079	2487.1814	-29.56	816	836	0	---	K.GICVADPYEVTVMQDFFIDLR.L

No match to: 842.4758, 853.4832, 855.4396, 865.9906, 868.5154, 868.5154, 870.5118, 877.0043, 882.5307, 882.5307, 892.9702, 951.5852, 969.4808, 973.4983, 1017.6315, 1017.6315, 1019.6440, 1033.6599, 1045.5276, 1051.6682, 1051.6682, 1066.4620, 1131.6697, 1151.6833, 1157.5516, 1165.6611, 1165.6611, 1179.5520, 1193.5681, 1277.6617, 1277.6617, 1300.8000, 1307.6257, 1307.6257, 1320.5388, 1334.8374, 1334.8374, 1351.6090, 1365.5970, 1383.6307, 1434.7792, 1448.8667, 1458.6595, 1475.6919, 1487.6974, 1612.7894, 1618.0076, 1618.0076, 1697.8160, 1697.8160, 1707.7140, 1716.7928, 1732.0361, 1838.8502, 1901.1791, 1993.9021, 2211.0298, 2283.1060, 2398.9456

12. A0A3Q1M2B2 **Mass:** 185169 **Score:** 83 **Expect:** 0.00072 **Matches:** 18

Complement C3 OS=Bos taurus OX=9913 GN=C3 PE=1 SV=1

Observed	Mr(expt)	Mr(calc)	ppm	Start	End	Miss	Ions	Peptide
833.4543	832.4470	832.4807	-40.43	803	809	0	---	R.LPYSVVR.N
833.4543	832.4471	832.4807	-40.37	803	809	0	29	R.LPYSVVR.N
848.4947	847.4874	847.4400	56.0	1388	1395	0	---	K.TLSTGVDR.Y
850.5049	849.4976	849.4266	83.6	639	645	0	---	R.SVQLMEK.R + Oxidation (M)
864.5046	863.4973	863.4939	3.99	758	764	0	---	K.LMNVFLK.D
887.4306	886.4233	886.4508	-31.02	810	816	0	---	R.NEQVEIR.A
912.4536	911.4463	911.4865	-44.04	817	823	0	---	R.AILYNYR.E
912.4536	911.4463	911.4865	-44.03	817	823	0	28	R.AILYNYR.E
996.5558	995.5485	995.4672	81.7	650	658	0	---	K.AGQYSSDLR.K
1090.4866	1089.4793	1089.4873	-7.34	590	600	0	---	K.ADIGCTPGSGR.N
1164.6057	1163.5984	1163.6411	-36.67	850	859	0	---	R.HQQTITIPAR.S
1349.6270	1348.6197	1348.6809	-45.38	678	689	0	---	R.AQFILQGDACVK.A
1420.6680	1419.6607	1419.7147	-37.99	882	894	0	---	K.AAVYNHFISDGVK.K
1427.7352	1426.7279	1426.6625	45.9	487	497	1	---	R.YYTYMIMNKGK.L + Oxidation (M)
1796.8849	1795.8776	1795.9178	-22.39	832	847	0	---	R.VELLYNPAFCSLATAK.K
1977.9272	1976.9199	1976.9884	-34.62	732	747	0	---	R.SQFPESWLWTVIEDLK.Q
2052.0193	2051.0120	2051.0874	-36.73	830	847	1	---	K.VRVELLYNPAFCSLATAK.K
2488.1152	2487.1079	2487.1814	-29.56	782	802	0	---	K.GICVADPYEVTVMQDFFIDLR.L

No match to: 842.4758, 853.4832, 855.4396, 865.9906, 868.5154, 868.5154, 870.5118, 877.0043, 882.5307, 882.5307, 892.9702, 951.5852, 969.4808, 973.4983, 1017.6315, 1017.6315, 1019.6440, 1033.6599, 1045.5276, 1051.6682, 1051.6682, 1066.4620, 1131.6697, 1151.6833, 1157.5516, 1165.6611, 1165.6611, 1179.5520, 1193.5681, 1277.6617, 1277.6617, 1300.8000, 1307.6257, 1307.6257, 1320.5388, 1334.8374, 1334.8374, 1351.6090, 1365.5970, 1383.6307, 1434.7792, 1448.8667, 1458.6595, 1475.6919, 1487.6974, 1612.7894, 1618.0076, 1618.0076, 1697.8160, 1697.8160, 1707.7140, 1716.7928, 1732.0361, 1838.8502, 1901.1791, 1993.9021, 2211.0298, 2283.1060, 2398.9456

13. A0A0F6QNP7 **Mass:** 188604 **Score:** 83 **Expect:** 0.00074 **Matches:** 18

Complement component 3 OS=Bos taurus OX=9913 GN=C3 PE=2 SV=1

Observed	Mr(expt)	Mr(calc)	ppm	Start	End	Miss	Ions	Peptide
833.4543	832.4470	832.4807	-40.43	834	840	0	---	R.LPYSVVR.N
833.4543	832.4471	832.4807	-40.37	834	840	0	29	R.LPYSVVR.N
848.4947	847.4874	847.4400	56.0	1419	1426	0	---	K.TLSTGVDR.Y
850.5049	849.4976	849.4266	83.6	670	676	0	---	R.SVQLMEK.R + Oxidation (M)
853.4832	852.4759	852.4779	-2.32	789	795	0	---	K.LMSVFLK.D + Oxidation (M)
887.4306	886.4233	886.4508	-31.02	841	847	0	---	R.NEQVEIR.A
912.4536	911.4463	911.4865	-44.04	848	854	0	---	R.AILYNYR.E
912.4536	911.4463	911.4865	-44.03	848	854	0	28	R.AILYNYR.E
996.5558	995.5485	995.4672	81.7	681	689	0	---	K.AGQYSSDLR.K
1090.4866	1089.4793	1089.4873	-7.34	621	631	0	---	K.ADIGCTPGSGR.N
1164.6057	1163.5984	1163.6411	-36.67	881	890	0	---	R.HQQTITIPAR.S
1349.6270	1348.6197	1348.6809	-45.38	709	720	0	---	R.AQFILQGDACVK.A
1420.6680	1419.6607	1419.7147	-37.99	913	925	0	---	K.AAVYNHFISDGVK.K
1427.7352	1426.7279	1426.6625	45.9	487	497	1	---	R.YYTYMIMNKGK.L + Oxidation (M)
1796.8849	1795.8776	1795.9178	-22.39	863	878	0	---	R.VELLYNPAFCSLATAK.K
1977.9272	1976.9199	1976.9884	-34.62	763	778	0	---	R.SQFPESWLWTVIEDLK.Q
2052.0193	2051.0120	2051.0874	-36.73	861	878	1	---	K.VRVELLYNPAFCSLATAK.K
2488.1152	2487.1079	2487.1814	-29.56	813	833	0	---	K.GICVADPYEVTVMQDFFIDLR.L

No match to: 842.4758, 855.4396, 864.5046, 865.9906, 868.5154, 868.5154, 870.5118, 877.0043, 882.5307, 882.5307, 892.9702, 951.5852, 969.4808, 973.4983, 1017.6315, 1017.6315, 1019.6440, 1033.6599, 1045.5276, 1051.6682, 1051.6682, 1066.4620, 1131.6697, 1151.6833, 1157.5516, 1165.6611, 1165.6611, 1179.5520, 1193.5681, 1277.6617, 1277.6617, 1300.8000, 1307.6257, 1307.6257, 1320.5388, 1334.8374, 1334.8374, 1351.6090, 1365.5970, 1383.6307, 1434.7792, 1448.8667, 1458.6595, 1475.6919, 1487.6974, 1612.7894, 1618.0076, 1618.0076, 1697.8160, 1697.8160, 1707.7140, 1716.7928, 1732.0361, 1838.8502, 1901.1791, 1993.9021, 2211.0298, 2283.1060, 2398.9456

14. A0A4W2EPI5 **Mass:** 182918 **Score:** 82 **Expect:** 0.00077 **Matches:** 17

Complement C3 OS=Bos indicus x Bos taurus OX=30522 GN=C3 PE=4 SV=1

Observed	Mr(expt)	Mr(calc)	ppm	Start	End	Miss	Ions	Peptide
833.4543	832.4470	832.4807	-40.43	834	840	0	---	R.LPYSVVR.N
833.4543	832.4807	832.4807	-40.37	834	840	0	29	R.LPYSVVR.N
850.5049	849.4976	849.4266	83.6	670	676	0	---	R.SVQLMEK.R + Oxidation (M)
864.5046	863.4973	863.4939	3.99	789	795	0	---	K.LMNVFLK.D
887.4306	886.4233	886.4508	-31.02	841	847	0	---	R.NEQVEIR.A
912.4536	911.4463	911.4865	-44.04	848	854	0	---	R.AILYNYR.E
912.4536	911.4463	911.4865	-44.03	848	854	0	28	R.AILYNYR.E
996.5558	995.5485	995.4672	81.7	681	689	0	---	K.AGQYSSDLR.K

PDF-XChange Click to buy NOW! www.docu-track.com

1090.4866	1089.4793	1089.4873	-7.34	621	631	0	---	K.ADIGCTPGSGR.N
1164.6057	1163.5984	1163.6411	-36.67	881	890	0	---	R.HQQTITIPAR.S
1349.6270	1348.6197	1348.6809	-45.38	709	720	0	---	R.AQFILQGDACVK.A
1420.6680	1419.6607	1419.7147	-37.99	913	925	0	---	K.AAVYNHFISDGVK.K
1427.7352	1426.7279	1426.6625	45.9	487	497	1	---	R.YYTYMIMNKGK.L + Oxidation (M)
1796.8849	1795.8776	1795.9178	-22.39	863	878	0	---	R.VELLYNPAFCSLATAK.K
1977.9272	1976.9199	1976.9884	-34.62	763	778	0	---	R.SQFPESWLWTVIEDLK.Q
2052.0193	2051.0120	2051.0874	-36.73	861	878	1	---	K.VRVELLYNPAFCSLATAK.K
2488.1152	2487.1079	2487.1814	-29.56	813	833	0	---	K.GICVADPYEVTVMQDFFIDLR.L

No match to: 842.4758, 848.4947, 853.4832, 855.4396, 865.9906, 868.5154, 868.5154, 870.5118, 877.0043, 882.5307, 882.5307, 892.9702, 951.5852, 969.4808, 973.4983, 1017.6315, 1017.6315, 1019.6440, 1033.6599, 1045.5276, 1051.6682, 1051.6682, 1066.4620, 1131.6697, 1151.6833, 1157.5516, 1165.6611, 1165.6611, 1179.5520, 1193.5681, 1277.6617, 1277.6617, 1300.8000, 1307.6257, 1307.6257, 1320.5388, 1334.8374, 1334.8374, 1351.6090, 1365.5970, 1383.6307, 1434.7792, 1448.8667, 1458.6595, 1475.6919, 1487.6974, 1612.7894, 1618.0076, 1618.0076, 1697.8160, 1697.8160, 1707.7140, 1716.7928, 1732.0361, 1838.8502, 1901.1791, 1993.9021, 2211.0298, 2283.1060, 2398.9456

15. A0A3Q1MHV6 **Mass:** 181738 **Score:** 81 **Expect:** 0.0011 **Matches:** 17

Complement C3 OS=Bos taurus OX=9913 GN=C3 PE=1 SV=1

Observed	Mr(expt)	Mr(calc)	ppm	Start	End	Miss	Ions	Peptide
833.4543	832.4470	832.4807	-40.43	803	809	0	---	R.LPYSVVR.N
833.4543	832.4471	832.4807	-40.37	803	809	0	29	R.LPYSVVR.N
850.5049	849.4976	849.4266	83.6	639	645	0	---	R.SVQLMEK.R + Oxidation (M)
864.5046	863.4973	863.4939	3.99	758	764	0	---	K.LMNVFLK.D
887.4306	886.4233	886.4508	-31.02	810	816	0	---	R.NEQVEIR.A
912.4536	911.4463	911.4865	-44.04	817	823	0	---	R.AILYNYR.E
912.4536	911.4463	911.4865	-44.03	817	823	0	28	R.AILYNYR.E
996.5558	995.5485	995.4672	81.7	650	658	0	---	K.AGQYSSDLR.K
1090.4866	1089.4793	1089.4873	-7.34	590	600	0	---	K.ADIGCTPGSGR.N
1164.6057	1163.5984	1163.6411	-36.67	850	859	0	---	R.HQQTITIPAR.S
1349.6270	1348.6197	1348.6809	-45.38	678	689	0	---	R.AQFILQGDACVK.A
1420.6680	1419.6607	1419.7147	-37.99	882	894	0	---	K.AAVYNHFISDGVK.K
1427.7352	1426.7279	1426.6625	45.9	487	497	1	---	R.YYTYMIMNKGK.L + Oxidation (M)
1796.8849	1795.8776	1795.9178	-22.39	832	847	0	---	R.VELLYNPAFCSLATAK.K
1977.9272	1976.9199	1976.9884	-34.62	732	747	0	---	R.SQFPESWLWTVIEDLK.Q
2052.0193	2051.0120	2051.0874	-36.73	830	847	1	---	K.VRVELLYNPAFCSLATAK.K
2488.1152	2487.1079	2487.1814	-29.56	782	802	0	---	K.GICVADPYEVTVMQDFFIDLR.L

No match to: 842.4758, 848.4947, 853.4832, 855.4396, 865.9906, 868.5154, 868.5154, 870.5118, 877.0043, 882.5307, 882.5307, 892.9702, 951.5852, 969.4808, 973.4983, 1017.6315, 1017.6315, 1019.6440, 1033.6599, 1045.5276, 1051.6682, 1051.6682, 1066.4620, 1131.6697, 1151.6833, 1157.5516, 1165.6611, 1165.6611, 1179.5520, 1193.5681, 1277.6617, 1277.6617, 1300.8000, 1307.6257, 1307.6257, 1320.5388, 1334.8374, 1334.8374, 1351.6090, 1365.5970, 1383.6307, 1434.7792, 1448.8667, 1458.6595, 1475.6919, 1487.6974, 1612.7894, 1618.0076, 1618.0076, 1697.8160, 1697.8160, 1707.7140, 1716.7928, 1732.0361, 1838.8502, 1901.1791, 1993.9021, 2211.0298, 2283.1060, 2398.9456

16. A0A4W2FTD2 **Mass:** 57549 **Score:** 40 **Expect:** 13 **Matches:** 5

Uncharacterized protein OS=Bos indicus x Bos taurus OX=30522 GN=LOC113883749 PE=3 SV=1

Observed	Mr(expt)	Mr(calc)	ppm	Start	End	Miss	Ions	Peptide
833.4543	832.4470	832.4629	-19.10	366	372	0	---	R.MFPIAVR.L
833.4543	832.4471	832.4629	-19.04	366	372	0	29	R.MFPIAVR.L
1151.6833	1150.6760	1150.6234	45.7	131	141	0	---	R.TLLSPTFTSGK.L
1193.5681	1192.5608	1192.6452	-70.74	380	390	0	---	K.DVEIHGVSIPK.G
2052.0193	2051.0120	2051.1680	-76.05	36	54	0	---	K.LGVPGPRPLPLFGNVLSYR.K

No match to: 842.4758, 848.4947, 850.5049, 853.4832, 855.4396, 864.5046, 865.9906, 868.5154, 868.5154, 870.5118, 877.0043, 882.5307, 882.5307, 887.4306, 892.9702, 912.4536, 912.4536, 951.5852, 969.4808, 973.4983, 996.5558, 1017.6315, 1017.6315, 1019.6440, 1033.6599, 1045.5276, 1051.6682, 1051.6682, 1066.4620, 1090.4866, 1131.6697, 1157.5516, 1164.6057, 1165.6611, 1165.6611, 1179.5520, 1277.6617, 1277.6617, 1300.8000, 1307.6257, 1307.6257, 1320.5388, 1334.8374, 1334.8374, 1349.6270, 1351.6090, 1365.5970, 1383.6307, 1420.6680, 1427.7352, 1434.7792, 1448.8667, 1458.6595, 1475.6919, 1487.6974, 1612.7894, 1618.0076, 1618.0076, 1697.8160, 1697.8160, 1707.7140, 1716.7928, 1732.0361, 1796.8849, 1838.8502, 1901.1791, 1977.9272, 1993.9021, 2211.0298, 2283.1060, 2398.9456, 2488.1152

17. A0A4W2BNQ3 **Mass:** 57413 **Score:** 40 **Expect:** 13 **Matches:** 5

Uncharacterized protein OS=Bos indicus x Bos taurus OX=30522 GN=LOC113883746 PE=3 SV=1

Observed	Mr(expt)	Mr(calc)	ppm	Start	End	Miss	Ions	Peptide
833.4543	832.4470	832.4629	-19.10	366	372	0	---	R.MFPIAVR.L
833.4543	832.4471	832.4629	-19.04	366	372	0	29	R.MFPIAVR.L
1151.6833	1150.6760	1150.6234	45.7	131	141	0	---	R.TLLSPTFTSGK.L
1193.5681	1192.5608	1192.6452	-70.74	380	390	0	---	K.DVEIHGVSIPK.G
2052.0193	2051.0120	2051.1680	-76.05	36	54	0	---	K.LGVPGPRPLPLFGNVLSYR.K

No match to: 842.4758, 848.4947, 850.5049, 853.4832, 855.4396, 864.5046, 865.9906, 868.5154, 868.5154, 870.5118, 877.0043, 882.5307, 882.5307, 887.4306, 892.9702, 912.4536, 912.4536, 951.5852, 969.4808, 973.4983, 996.5558, 1017.6315, 1017.6315, 1019.6440, 1033.6599, 1045.5276, 1051.6682, 1051.6682, 1066.4620, 1090.4866, 1131.6697, 1157.5516, 1164.6057, 1165.6611, 1165.6611, 1179.5520, 1277.6617, 1277.6617, 1300.8000, 1307.6257, 1307.6257, 1320.5388, 1334.8374, 1334.8374, 1349.6270, 1351.6090, 1365.5970, 1383.6307, 1420.6680, 1427.7352, 1434.7792, 1448.8667, 1458.6595, 1475.6919, 1487.6974, 1612.7894, 1618.0076, 1618.0076, 1697.8160, 1697.8160, 1707.7140, 1716.7928, 1732.0361, 1796.8849, 1838.8502, 1901.1791, 1977.9272, 1993.9021, 2211.0298, 2283.1060, 2398.9456, 2488.1152

18. A0A4W2HPR9 **Mass:** 57408 **Score:** 40 **Expect:** 13 **Matches:** 5

Uncharacterized protein OS=Bos indicus x Bos taurus OX=30522 GN=LOC113883749 PE=3 SV=1

Observed	Mr(expt)	Mr(calc)	ppm	Start	End	Miss	Ions	Peptide
833.4543	832.4470	832.4629	-19.10	366	372	0	---	R.MFPIAVR.L
833.4543	832.4471	832.4629	-19.04	366	372	0	29	R.MFPIAVR.L
1151.6833	1150.6760	1150.6234	45.7	131	141	0	---	R.TLLSPTFTSGK.L
1193.5681	1192.5608	1192.6452	-70.74	380	390	0	---	K.DVEIHGVSIPK.G
2052.0193	2051.0120	2051.1680	-76.05	36	54	0	---	K.LGVPGPRPLPLFGNVLSYR.K

No match to: 842.4758, 848.4947, 850.5049, 853.4832, 855.4396, 864.5046, 865.9906, 868.5154, 868.5154, 870.5118, 877.0043, 882.5307, 882.5307, 887.4306, 892.9702, 912.4536, 912.4536, 951.5852, 969.4808, 973.4983, 996.5558, 1017.6315, 1017.6315, 1019.6440, 1033.6599, 1045.5276, 1051.6682, 1051.6682, 1066.4620, 1090.4866, 1131.6697, 1157.5516, 1164.6057, 1165.6611, 1165.6611, 1179.5520, 1277.6617, 1277.6617, 1300.8000, 1307.6257, 1307.6257, 1320.5388, 1334.8374, 1334.8374, 1349.6270, 1351.6090, 1365.5970, 1383.6307, 1420.6680, 1427.7352, 1434.7792, 1448.8667, 1458.6595, 1475.6919, 1487.6974, 1612.7894, 1618.0076, 1618.0076, 1697.8160, 1697.8160, 1707.7140, 1716.7928, 1732.0361, 1796.8849, 1838.8502, 1901.1791, 1977.9272, 1993.9021, 2211.0298, 2283.1060, 2398.9456, 2488.1152

19. A0A4W2BMU5 **Mass:** 57554 **Score:** 40 **Expect:** 13 **Matches:** 5

PDF-XChange Click to buy NOW! www.docu-track.com

Uncharacterized protein OS=Bos indicus x Bos taurus OX=30522 GN=LOC113883746 PE=3 SV=1

Observed	Mr(expt)	Mr(calc)	ppm	Start	End	Miss	Ions	Peptide
833.4543	832.4470	832.4629	-19.10	366	372	0	---	R.MFPIAVR.L
833.4543	832.4471	832.4629	-19.04	366	372	0	29	R.MFPIAVR.L
1151.6833	1150.6760	1150.6234	45.7	131	141	0	---	R.TLLSPTFTSGK.L
1193.5681	1192.5608	1192.6452	-70.74	380	390	0	---	K.DVEIHGVSIPK.G
2052.0193	2051.0120	2051.1680	-76.05	36	54	0	---	K.LGVPGPRPLPLFGNVLSYR.K

No match to: 842.4758, 848.4947, 850.5049, 855.4396, 864.5046, 865.9906, 868.5154, 868.5154, 870.5118, 877.0043, 882.5307, 882.5307, 887.4306, 892.9702, 912.4536, 912.4536, 951.5852, 969.4808, 973.4983, 996.5558, 1017.6315, 1017.6315, 1019.6440, 1033.6599, 1045.5276, 1051.6682, 1051.6682, 1066.4620, 1090.4866, 1131.6697, 1157.5516, 1164.6057, 1165.6611, 1165.6611, 1179.5520, 1277.6617, 1277.6617, 1300.8000, 1307.6257, 1307.6257, 1320.5388, 1334.8374, 1334.8374, 1349.6270, 1351.6090, 1365.5970, 1383.6307, 1420.6680, 1427.7352, 1434.7792, 1448.8667, 1458.6595, 1475.6919, 1487.6974, 1612.7894, 1618.0076, 1618.0076, 1697.8160, 1697.8160, 1707.7140, 1716.7928, 1732.0361, 1796.8849, 1838.8502, 1901.1791, 1977.9272, 1993.9021, 2211.0298, 2283.1060, 2398.9456, 2488.1152

20. Q3T047 **Mass:** 57540 **Score:** 40 **Expect:** 13 **Matches:** 5

Cytochrome P450, family 3, subfamily A, polypeptide 5 OS=Bos taurus OX=9913 GN=CYP3A5 PE=1 SV=1

Observed	Mr(expt)	Mr(calc)	ppm	Start	End	Miss	Ions	Peptide
833.4543	832.4470	832.4629	-19.10	366	372	0	---	R.MFPIAVR.L
833.4543	832.4471	832.4629	-19.04	366	372	0	29	R.MFPIAVR.L
1151.6833	1150.6760	1150.6234	45.7	131	141	0	---	R.TLLSPTFTSGK.L
1193.5681	1192.5608	1192.6452	-70.74	380	390	0	---	K.DVEIHGVSIPK.G
2052.0193	2051.0120	2051.1680	-76.05	36	54	0	---	K.LGVPGPRPLPLFGNVLSYR.K

No match to: 842.4758, 848.4947, 850.5049, 853.4832, 855.4396, 864.5046, 865.9906, 868.5154, 868.5154, 870.5118, 877.0043, 882.5307, 882.5307, 887.4306, 892.9702, 912.4536, 912.4536, 951.5852, 969.4808, 973.4983, 996.5558, 1017.6315, 1017.6315, 1019.6440, 1033.6599, 1045.5276, 1051.6682, 1051.6682, 1066.4620, 1090.4866, 1131.6697, 1157.5516, 1164.6057, 1165.6611, 1165.6611, 1179.5520, 1277.6617, 1277.6617, 1300.8000, 1307.6257, 1307.6257, 1320.5388, 1334.8374, 1334.8374, 1349.6270, 1351.6090, 1365.5970, 1383.6307, 1420.6680, 1427.7352, 1434.7792, 1448.8667, 1458.6595, 1475.6919, 1487.6974, 1612.7894, 1618.0076, 1618.0076, 1697.8160, 1697.8160, 1707.7140, 1716.7928, 1732.0361, 1796.8849, 1838.8502, 1901.1791, 1977.9272, 1993.9021, 2211.0298, 2283.1060, 2398.9456, 2488.1152

21. A0A3Q1MUB2 **Mass:** 54957 **Score:** 39 **Expect:** 18 **Matches:** 12

Glucosaminyl (N-acetyl) transferase 4 OS=Bos taurus OX=9913 GN=GCNT4 PE=4 SV=1

Observed	Mr(expt)	Mr(calc)	ppm	Start	End	Miss	Ions	Peptide
833.4543	832.4470	832.4178	35.1	137	143	0	---	K.LVSEEEK.S
833.4543	832.4471	832.4178	35.2	137	143	0	---	K.LVSEEEK.S
996.5558	995.5485	995.5651	-16.69	359	368	0	---	R.VPGIPGEISK.T
1033.6599	1032.6526	1032.5968	54.1	448	456	1	---	R.KWITLSSAK.L
1131.6697	1130.6624	1130.5760	76.4	328	336	0	---	K.YIFNNSFVK.D
1165.6611	1164.6538	1164.6026	44.0	249	258	0	---	K.SNFELVSELK.K
1165.6611	1164.6539	1164.6026	44.0	249	258	0	---	K.SNFELVSELK.K
1448.8667	1447.8594	1447.7267	91.7	369	381	1	---	K.TAQDVSDLQSKTR.L
1697.8160	1696.8087	1696.8939	-50.19	2	15	0	---	M.CLLCHRPLIISGMK.T
1697.8160	1696.8088	1696.8939	-50.17	2	15	0	---	M.CLLCHRPLIISGMK.T
1732.0361	1731.0288	1730.8873	81.8	260	275	0	---	K.LNGSNMLETVKPPSTK.T + Oxidation (M)
1796.8849	1795.8776	1795.9113	-18.75	403	417	1	---	R.SVCIYGAAELRWLMK.Y

No match to: 842.4758, 848.4947, 850.5049, 853.4832, 855.4396, 864.5046, 865.9906, 868.5154, 868.5154, 870.5118, 877.0043, 882.5307, 882.5307, 887.4306, 892.9702, 912.4536, 912.4536, 951.5852, 969.4808, 973.4983, 1017.6315, 1017.6315, 1019.6440, 1045.5276, 1051.6682, 1051.6682, 1066.4620, 1090.4866, 1151.6833, 1157.5516, 1164.6057, 1179.5520, 1193.5681, 1277.6617, 1277.6617, 1300.8000, 1307.6257, 1307.6257, 1320.5388, 1334.8374, 1334.8374, 1349.6270, 1351.6090, 1365.5970, 1383.6307, 1420.6680, 1427.7352, 1434.7792, 1458.6595, 1475.6919, 1487.6974, 1612.7894, 1618.0076, 1618.0076, 1707.7140, 1716.7928, 1838.8502, 1901.1791, 1977.9272, 1993.9021, 2052.0193, 2211.0298, 2283.1060, 2398.9456, 2488.1152

22. F1MDR5 **Mass:** 92495 **Score:** 38 **Expect:** 21 **Matches:** 16

Uncharacterized protein OS=Bos taurus OX=9913 GN=MIA2 PE=4 SV=1

Observed	Mr(expt)	Mr(calc)	ppm	Start	End	Miss	Ions	Peptide
842.4758	841.4685	841.4657	3.30	640	647	0	---	R.LSGPAELR.S
850.5049	849.4976	849.4821	18.3	67	73	1	---	R.SRLYVGR.E
864.5046	863.4973	863.4865	12.6	69	75	1	---	R.LYVGREK.K
868.5154	867.5081	867.4311	88.8	485	491	1	---	R.KENAHNR.Q
868.5154	867.5082	867.4311	88.9	485	491	1	---	R.KENAHNR.Q
973.4983	972.4910	972.4876	3.53	240	247	0	---	R.EQVNELNK.Q
996.5558	995.5485	995.4897	59.1	486	493	1	---	K.ENAHNRQK.L
1066.4620	1065.4547	1065.4913	-34.33	202	209	0	---	K.IFQMNEER.L
1157.5516	1156.5443	1156.5183	22.5	248	256	1	---	K.QRMTFEDSK.V + Oxidation (M)
1179.5520	1178.5447	1178.5489	-3.52	168	177	0	---	K.QDELMSEISK.R
1307.6257	1306.6184	1306.6703	-39.72	202	211	1	---	K.IFQMNEERLK.I
1307.6257	1306.6185	1306.6703	-39.69	202	211	1	---	K.IFQMNEERLK.I
1351.6090	1350.6017	1350.6449	-31.96	168	178	1	---	K.QDELMSEISKR.I + Oxidation (M)
1707.7140	1706.7067	1706.8239	-68.66	722	738	0	---	R.GPPFPPPPPGSMYGGPR.D
1838.8502	1837.8429	1837.9356	-50.44	257	271	1	---	K.VHVEQVLCDKENQIK.S
1993.9021	1992.8948	1992.9476	-26.47	521	538	0	---	R.EHSPYGPSPLGRPSPEMR.A

No match to: 833.4543, 833.4543, 848.4947, 853.4832, 855.4396, 865.9906, 870.5118, 877.0043, 882.5307, 882.5307, 887.4306, 892.9702, 912.4536, 912.4536, 951.5852, 969.4808, 1017.6315, 1017.6315, 1019.6440, 1033.6599, 1045.5276, 1051.6682, 1051.6682, 1090.4866, 1131.6697, 1151.6833, 1164.6057, 1165.6611, 1165.6611, 1193.5681, 1277.6617, 1277.6617, 1300.8000, 1320.5388, 1334.8374, 1334.8374, 1349.6270, 1365.5970, 1383.6307, 1420.6680, 1427.7352, 1434.7792, 1448.8667, 1458.6595, 1475.6919, 1487.6974, 1612.7894, 1618.0076, 1618.0076, 1697.8160, 1697.8160, 1716.7928, 1732.0361, 1796.8849, 1901.1791, 1977.9272, 2052.0193, 2211.0298, 2283.1060, 2398.9456, 2488.1152

23. Q08DU7 **Mass:** 51251 **Score:** 37 **Expect:** 27 **Matches:** 10

mTERF protein (Fragment) OS=Bos taurus OX=9913 GN=MTERF PE=2 SV=1

Observed	Mr(expt)	Mr(calc)	ppm	Start	End	Miss	Ions	Peptide
973.4983	972.4910	972.5253	-35.29	128	135	1	---	K.RQPGVFNR.T
1164.6057	1163.5984	1163.6339	-30.48	218	227	0	---	K.FLYSVGLTHK.D
1179.5520	1178.5447	1178.5965	-43.94	334	344	1	---	K.DKLSSLGCTAK.E
1193.5681	1192.5608	1192.6750	-95.77	50	60	0	---	K.GLGHLTIMVPR.K
1334.8374	1333.8301	1333.7024	95.8	336	347	1	---	K.LSSLGCTAKEIR.K
1334.8374	1333.8301	1333.7024	95.8	336	347	1	---	K.LSSLGCTAKEIR.K
1365.5970	1364.5897	1364.6540	-47.13	317	327	1	---	K.ILDLSSDCMRR.S
1475.6919	1474.6846	1474.8031	-80.33	430	441	1	---	K.KLYIEQNIVLEN.-
1732.0361	1731.0288	1730.9679	35.2	151	166	1	---	K.GASIEVIASIISRYPR.A

PDF-XChange Click to buy NOW! www.docu-track.com

PDF-XChange Click to buy NOW! www.docu-track.com

2211.0298	2210.0225	2210.0603	-17.11	199 -	217	1	---	R.SPESFFRSSDNLNLENNIK.F

No match to: 833.4543, 833.4543, 842.4758, 848.4947, 850.5049, 853.4832, 855.4396, 864.5046, 865.9906, 868.5154, 868.5154, 870.5118, 877.0043, 882.5307, 882.5307, 887.4306, 892.9702, 912.4536, 912.4536, 951.5852, 969.4808, 996.5558, 1017.6315, 1017.6315, 1019.6440, 1033.6599, 1045.5276, 1051.6682, 1051.6682, 1066.4620, 1090.4866, 1131.6697, 1151.6833, 1157.5516, 1165.6611, 1165.6611, 1277.6617, 1277.6617, 1300.8000, 1307.6257, 1307.6257, 1320.5388, 1349.6270, 1351.6090, 1383.6307, 1420.6680, 1427.7352, 1434.7792, 1448.8667, 1458.6595, 1487.6974, 1612.7894, 1618.0076, 1618.0076, 1697.8160, 1697.8160, 1707.7140, 1716.7928, 1796.8849, 1838.8502, 1901.1791, 1977.9272, 1993.9021, 2052.0193, 2283.1060, 2398.9456, 2488.1152

24. A0A4W2FV66 **Mass:** 129413 **Score:** 37 **Expect:** 29 **Matches:** 15
Replication factor C subunit 1 OS=Bos indicus x Bos taurus OX=30522 GN=RFC1 PE=3 SV=1

Observed	Mr(expt)	Mr(calc)	ppm	Start	End	Miss	Ions	Peptide
951.5852	950.5779	950.5226	58.2	6 -	14	0	---	K.FFGVIPSGK.K
969.4808	968.4735	968.4749	-1.47	443 -	450	0	---	K.TNYLVMGR.D + Oxidation (M)
1090.4866	1089.4793	1089.4826	-2.98	363 -	372	0	---	K.ESVSPDEAEK.R
1151.6833	1150.6760	1150.5982	67.6	824 -	833	1	---	R.NKALTYDQAK.A
1300.8000	1299.7927	1299.7411	39.7	771 -	780	1	---	R.FQRPRVEQIK.G
1307.6257	1306.6184	1306.5832	27.0	745 -	754	0	---	K.IPIICMCNDR.N + Oxidation (M)
1307.6257	1306.6185	1306.5832	27.0	745 -	754	0	---	K.IPIICMCNDR.N + Oxidation (M)
1349.6270	1348.6197	1348.6623	-31.54	51 -	61	1	---	K.EDEFKPKQSNK.K
1383.6307	1382.6234	1382.7340	-79.98	974 -	985	0	---	R.TVQDLALHMSLR.T
1458.6595	1457.6522	1457.6998	-32.62	206 -	217	0	---	K.QLQLDEDAELER.Q
1475.6919	1474.6846	1474.7164	-21.57	826 -	838	1	---	K.ALTYDQAKADSHR.A
1796.8849	1795.8776	1795.8121	36.5	854 -	869	0	---	K.VFVSGEETAHMSLMDK.S + Oxidation (M)
1977.9272	1976.9199	1977.0353	-58.36	794 -	811	0	---	K.IPPPAMNEIILGANQDIR.Q + Oxidation (M)
1993.9021	1992.8948	1993.0105	-58.02	761 -	775	1	---	R.SLVHYCFDLRFQRPR.V
2398.9456	2397.9383	2398.0900	-63.23	1032 -	1052	0	---	K.DDFENIMEISSWGGRPSPFSK.L

No match to: 833.4543, 833.4543, 842.4758, 848.4947, 850.5049, 853.4832, 855.4396, 864.5046, 865.9906, 868.5154, 868.5154, 870.5118, 877.0043, 882.5307, 882.5307, 887.4306, 892.9702, 912.4536, 912.4536, 973.4983, 996.5558, 1017.6315, 1017.6315, 1019.6440, 1033.6599, 1045.5276, 1051.6682, 1051.6682, 1066.4620, 1131.6697, 1157.5516, 1164.6057, 1165.6611, 1165.6611, 1179.5520, 1193.5681, 1277.6617, 1277.6617, 1320.5388, 1334.8374, 1334.8374, 1351.6090, 1365.5970, 1420.6680, 1427.7352, 1434.7792, 1448.8667, 1487.6974, 1612.7894, 1618.0076, 1618.0076, 1697.8160, 1697.8160, 1707.7140, 1716.7928, 1732.0361, 1838.8502, 1901.1791, 2052.0193, 2211.0298, 2283.1060, 2488.1152

25. A0A4W2EXE5 **Mass:** 129354 **Score:** 36 **Expect:** 32 **Matches:** 15
Replication factor C subunit 1 OS=Bos indicus x Bos taurus OX=30522 GN=RFC1 PE=3 SV=1

Observed	Mr(expt)	Mr(calc)	ppm	Start	End	Miss	Ions	Peptide
951.5852	950.5779	950.5226	58.2	6 -	14	0	---	K.FFGVIPSGK.K
969.4808	968.4735	968.4749	-1.47	443 -	450	0	---	K.TNYLVMGR.D + Oxidation (M)
1090.4866	1089.4793	1089.4826	-2.98	363 -	372	0	---	K.ESVSPDEAEK.R
1151.6833	1150.6760	1150.5982	67.6	823 -	832	1	---	R.NKALTYDQAK.A
1300.8000	1299.7927	1299.7411	39.7	770 -	779	1	---	R.FQRPRVEQIK.G
1307.6257	1306.6184	1306.5832	27.0	744 -	753	0	---	K.IPIICMCNDR.N + Oxidation (M)
1307.6257	1306.6185	1306.5832	27.0	744 -	753	0	---	K.IPIICMCNDR.N + Oxidation (M)
1349.6270	1348.6197	1348.6623	-31.54	51 -	61	1	---	K.EDEFKPKQSNK.K
1383.6307	1382.6234	1382.7340	-79.98	973 -	984	0	---	R.TVQDLALHMSLR.T
1458.6595	1457.6522	1457.6998	-32.62	206 -	217	0	---	K.QLQLDEDAELER.Q
1475.6919	1474.6846	1474.7164	-21.57	825 -	837	1	---	K.ALTYDQAKADSHR.A
1796.8849	1795.8776	1795.8121	36.5	853 -	868	0	---	K.VFVSGEETAHMSLMDK.S + Oxidation (M)
1977.9272	1976.9199	1977.0353	-58.36	793 -	810	0	---	K.IPPPAMNEIILGANQDIR.Q + Oxidation (M)
1993.9021	1992.8948	1993.0105	-58.02	760 -	774	1	---	R.SLVHYCFDLRFQRPR.V
2398.9456	2397.9383	2398.0900	-63.23	1031 -	1051	0	---	K.DDFENIMEISSWGGRPSPFSK.L

No match to: 833.4543, 833.4543, 842.4758, 848.4947, 850.5049, 853.4832, 855.4396, 864.5046, 865.9906, 868.5154, 868.5154, 870.5118, 877.0043, 882.5307, 882.5307, 887.4306, 892.9702, 912.4536, 912.4536, 973.4983, 996.5558, 1017.6315, 1017.6315, 1019.6440, 1033.6599, 1045.5276, 1051.6682, 1051.6682, 1066.4620, 1131.6697, 1157.5516, 1164.6057, 1165.6611, 1165.6611, 1179.5520, 1193.5681, 1277.6617, 1277.6617, 1320.5388, 1334.8374, 1334.8374, 1351.6090, 1365.5970, 1420.6680, 1427.7352, 1434.7792, 1448.8667, 1487.6974, 1612.7894, 1618.0076, 1618.0076, 1697.8160, 1697.8160, 1707.7140, 1716.7928, 1732.0361, 1838.8502, 1901.1791, 2052.0193, 2211.0298, 2283.1060, 2488.1152

26. A0A4W2BTQ2 **Mass:** 97692 **Score:** 36 **Expect:** 32 **Matches:** 12
Kinesin-like protein OS=Bos indicus x Bos taurus OX=30522 GN=KIF9 PE=3 SV=1

Observed	Mr(expt)	Mr(calc)	ppm	Start	End	Miss	Ions	Peptide
833.4543	832.4470	832.4807	-40.43	184 -	190	0	---	R.ALQQVFK.M
833.4543	832.4471	832.4807	-40.37	184 -	190	0	---	R.ALQQVFK.M
848.4947	847.4874	847.5140	-31.40	177 -	183	1	---	K.HRGILPR.A
864.5046	863.4973	863.4236	85.4	586 -	593	0	---	K.DLDVSTSK.T
887.4306	886.4233	886.4984	-84.72	665 -	671	1	---	K.SILNERR.K
996.5558	995.5485	995.4892	59.6	49 -	56	1	---	K.LSQMMRSK.N + Oxidation (M)
1179.5520	1178.5447	1178.5931	-41.07	515 -	525	0	---	K.NDYAAISAVQK.A
1458.6595	1457.6522	1457.7409	-60.80	268 -	280	1	---	R.IIASHTMNKNSSR.S
1475.6919	1474.6846	1474.6787	4.01	614 -	627	0	---	R.ETSNTEPLASDSPK.E
1901.1791	1900.1718	1900.0014	89.7	492 -	507	1	---	R.VVLSQQEQEVESTLRR.K
1977.9272	1976.9199	1977.0294	-55.40	33 -	48	0	---	R.HHPVLQAACYLIWELK.L
2488.1152	2487.1079	2487.1523	-17.83	141 -	164	0	---	K.DVVSQALNGFNGTIMCYGQTGAGK.T

No match to: 842.4758, 850.5049, 853.4832, 855.4396, 865.9906, 868.5154, 868.5154, 870.5118, 877.0043, 882.5307, 882.5307, 892.9702, 912.4536, 912.4536, 951.5852, 969.4808, 973.4983, 1017.6315, 1017.6315, 1019.6440, 1033.6599, 1045.5276, 1051.6682, 1051.6682, 1066.4620, 1090.4866, 1131.6697, 1151.6833, 1157.5516, 1164.6057, 1165.6611, 1165.6611, 1193.5681, 1277.6617, 1277.6617, 1300.8000, 1307.6257, 1307.6257, 1320.5388, 1334.8374, 1334.8374, 1349.6270, 1351.6090, 1365.5970, 1383.6307, 1420.6680, 1427.7352, 1434.7792, 1448.8667, 1487.6974, 1612.7894, 1618.0076, 1618.0076, 1697.8160, 1697.8160, 1707.7140, 1716.7928, 1732.0361, 1796.8849, 1838.8502, 1993.9021, 2052.0193, 2211.0298, 2283.1060, 2398.9456

27. A0A0K2YTL5 **Mass:** 9022 **Score:** 36 **Expect:** 33 **Matches:** 6
Uncharacterized protein OS=Cowpox virus OX=10243 GN=gCPXV0248 PE=4 SV=1

Observed	Mr(expt)	Mr(calc)	ppm	Start	End	Miss	Ions	Peptide
1131.6697	1130.6624	1130.6270	31.3	31 -	39	0	---	K.YIVLMIAHR.V + Oxidation (M)
1277.6617	1276.6544	1276.7292	-58.53	70 -	79	1	---	R.LKFNLYRPNL.-
1277.6617	1276.6545	1276.7292	-58.50	70 -	79	1	---	R.LKFNLYRPNL.-
1334.8374	1333.8301	1333.7275	76.9	58 -	69	0	---	R.STMELILLTASR.L
1334.8374	1333.8301	1333.7275	76.9	58 -	69	0	---	R.STMELILLTASR.L
2052.0193	2051.0120	2051.1714	-77.69	13 -	30	1	---	K.LALRVLVISNGYCHVPLK.Y

PDF-XChange Click to buy NOW! www.docu-track.com
PDF-XChange Click to buy NOW! www.docu-track.com

No match to: 833.4543, 833.4543, 842.4758, 848.4947, 850.5049, 853.4832, 855.4396, 864.5046, 865.9906, 868.5154, 868.5154, 870.5118, 877.0043, 882.5307, 882.5307, 887.4306, 892.9702, 912.4536, 912.4536, 951.5852, 969.4808, 973.4983, 996.5558, 1017.6315, 1017.6315, 1019.6440, 1033.6599, 1045.5276, 1051.6682, 1051.6682, 1066.4620, 1090.4866, 1151.6833, 1157.5516, 1164.6057, 1165.6611, 1165.6611, 1179.5520, 1193.5681, 1300.8000, 1307.6257, 1307.6257, 1320.5388, 1349.6270, 1351.6090, 1365.5970, 1383.6307, 1420.6680, 1427.7352, 1434.7792, 1448.8667, 1458.6595, 1475.6919, 1487.6974, 1612.7894, 1618.0076, 1618.0076, 1697.8160, 1697.8160, 1707.7140, 1716.7928, 1732.0361, 1796.8849, 1838.8502, 1901.1791, 1977.9272, 1993.9021, 2211.0298, 2283.1060, 2398.9456, 2488.1152

28. A0A3Q1N5S1 **Mass:** 128941 **Score:** 36 **Expect:** 38 **Matches:** 14

Replication factor C subunit 1 OS=Bos taurus OX=9913 GN=RFC1 PE=3 SV=1

Observed	Mr(expt)	Mr(calc)	ppm	Start		End	Miss	Ions	Peptide
969.4808	968.4735	968.4749	-1.47	437	-	444	0	---	K.TNYLVMGR.D + Oxidation (M)
1090.4866	1089.4793	1089.4826	-2.98	357	-	366	0	---	K.ESVSPDEAEK.R
1151.6833	1150.6760	1150.5982	67.6	818	-	827	1	---	R.NKALTYDQAK.A
1300.8000	1299.7927	1299.7411	39.7	765	-	774	1	---	R.FQRPRVEQIK.G
1307.6257	1306.6184	1306.5832	27.0	739	-	748	0	---	K.IPIICMCNDR.N + Oxidation (M)
1307.6257	1306.6185	1306.5832	27.0	739	-	748	0	---	K.IPIICMCNDR.N + Oxidation (M)
1349.6270	1348.6197	1348.6623	-31.54	34	-	44	1	---	K.EDEFKPKQSNK.K
1383.6307	1382.6234	1382.7340	-79.98	968	-	979	0	---	R.TVQDLALHMSLR.T
1458.6595	1457.6522	1457.6998	-32.62	203	-	214	0	---	K.QLQLDEDAELER.Q
1475.6919	1474.6846	1474.7164	-21.57	820	-	832	1	---	K.ALTYDQAKADSHR.A
1796.8849	1795.8776	1795.8121	36.5	848	-	863	0	---	K.VFVSGEETAHMSLMDK.S + Oxidation (M)
1977.9272	1976.9199	1977.0353	-58.36	788	-	805	0	---	K.IPPPAMNEIILGANQDIR.Q + Oxidation (M)
1993.9021	1992.8948	1993.0105	-58.02	755	-	769	1	---	R.SLVHYCFDLRFQRPR.V
2398.9456	2397.9383	2398.0900	-63.23	1026	-	1046	0	---	K.DDFENIMEISSWGGRPSPFSK.L

No match to: 833.4543, 833.4543, 842.4758, 848.4947, 850.5049, 853.4832, 855.4396, 864.5046, 865.9906, 868.5154, 868.5154, 870.5118, 877.0043, 882.5307, 882.5307, 887.4306, 892.9702, 912.4536, 912.4536, 951.5852, 973.4983, 996.5558, 1017.6315, 1017.6315, 1019.6440, 1033.6599, 1045.5276, 1051.6682, 1051.6682, 1066.4620, 1131.6697, 1157.5516, 1164.6057, 1165.6611, 1165.6611, 1179.5520, 1193.5681, 1277.6617, 1277.6617, 1320.5388, 1334.8374, 1334.8374, 1351.6090, 1365.5970, 1420.6680, 1427.7352, 1434.7792, 1448.8667, 1487.6974, 1612.7894, 1618.0076, 1618.0076, 1697.8160, 1697.8160, 1707.7140, 1716.7928, 1732.0361, 1838.8502, 1901.1791, 2052.0193, 2211.0298, 2283.1060, 2488.1152

29. A0A4W2H4H4 **Mass:** 50468 **Score:** 35 **Expect:** 39 **Matches:** 11

Interferon induced protein 44 like OS=Bos indicus x Bos taurus OX=30522 GN=IFI44L PE=4 SV=1

Observed	Mr(expt)	Mr(calc)	ppm	Start		End	Miss	Ions	Peptide
868.5154	867.5081	867.5542	-53.07	392	-	399	0	---	K.ILGIPLSR.I
868.5154	867.5082	867.5542	-53.03	392	-	399	0	---	K.ILGIPLSR.I
887.4306	886.4233	886.4872	-72.07	181	-	188	0	---	R.NSLLADVR.A
1307.6257	1306.6184	1306.6629	-34.06	165	-	175	1	---	K.DEAGYINRITR.A
1307.6257	1306.6185	1306.6629	-34.04	165	-	175	1	---	K.DEAGYINRITR.A
1420.6680	1419.6607	1419.6994	-27.24	2	-	13	0	---	M.AVTTTLTWNEER.S
1434.7792	1433.7719	1433.7554	11.5	293	-	304	0	---	R.YEFSPQKPITPK.H
1838.8502	1837.8429	1837.9026	-32.46	376	-	391	1	---	K.MNKAMISQSQIQNVSK.I + 2 Oxidation (M)
1977.9272	1976.9199	1976.9877	-34.28	400	-	416	1	---	R.ILVVDNYASEREMDPVK.D
1993.9021	1992.8948	1992.9826	-44.05	400	-	416	1	---	R.ILVVDNYASEREMDPVK.D + Oxidation (M)
2283.1060	2282.0987	2282.2780	-78.55	379	-	399	1	---	K.AMISQSQIQNVSKILGIPLSR.I

No match to: 833.4543, 833.4543, 842.4758, 848.4947, 850.5049, 853.4832, 855.4396, 864.5046, 865.9906, 870.5118, 877.0043, 882.5307, 882.5307, 892.9702, 912.4536, 912.4536, 951.5852, 969.4808, 973.4983, 996.5558, 1017.6315, 1017.6315, 1019.6440, 1033.6599, 1045.5276, 1051.6682, 1051.6682, 1066.4620, 1090.4866, 1131.6697, 1151.6833, 1157.5516, 1164.6057, 1165.6611, 1165.6611, 1179.5520, 1193.5681, 1277.6617, 1277.6617, 1300.8000, 1320.5388, 1334.8374, 1334.8374, 1349.6270, 1351.6090, 1365.5970, 1383.6307, 1427.7352, 1448.8667, 1458.6595, 1475.6919, 1487.6974, 1612.7894, 1618.0076, 1618.0076, 1697.8160, 1697.8160, 1707.7140, 1716.7928, 1732.0361, 1796.8849, 1901.1791, 2052.0193, 2211.0298, 2398.9456, 2488.1152

30. A0A4W2BTP7 **Mass:** 104127 **Score:** 35 **Expect:** 40 **Matches:** 12

Kinesin-like protein OS=Bos indicus x Bos taurus OX=30522 GN=KIF9 PE=3 SV=1

Observed	Mr(expt)	Mr(calc)	ppm	Start		End	Miss	Ions	Peptide
833.4543	832.4470	832.4807	-40.43	184	-	190	0	---	R.ALQQVFK.M
833.4543	832.4471	832.4807	-40.37	184	-	190	0	---	R.ALQQVFK.M
848.4947	847.4874	847.5140	-31.40	177	-	183	1	---	K.HRGILPR.A
864.5046	863.4973	863.4236	85.4	586	-	593	0	---	K.DLDVSTSK.T
887.4306	886.4233	886.4984	-84.72	665	-	671	1	---	K.SILNERR.K
996.5558	995.5485	995.4892	59.6	49	-	56	1	---	K.LSQMMRSK.N + Oxidation (M)
1179.5520	1178.5447	1178.5931	-41.07	515	-	525	0	---	K.NDYAAISAVQK.A
1458.6595	1457.6522	1457.7409	-60.80	268	-	280	1	---	R.IIASHTMNKNSSR.S
1475.6919	1474.6846	1474.6787	4.01	614	-	627	0	---	R.ETSNTEPLASDSPK.E
1901.1791	1900.1718	1900.0014	89.7	492	-	507	1	---	R.VVLSQQEQEVESTLRR.K
1977.9272	1976.9199	1977.0294	-55.40	33	-	48	0	---	R.HHPVLQAACYLIWELK.L
2488.1152	2487.1079	2487.1523	-17.83	141	-	164	0	---	K.DVVSQALNGFNGTIMCYGQTGAGK.T

No match to: 842.4758, 850.5049, 853.4832, 855.4396, 865.9906, 868.5154, 868.5154, 870.5118, 877.0043, 882.5307, 882.5307, 892.9702, 912.4536, 912.4536, 951.5852, 969.4808, 973.4983, 1017.6315, 1017.6315, 1019.6440, 1033.6599, 1045.5276, 1051.6682, 1051.6682, 1066.4620, 1090.4866, 1131.6697, 1151.6833, 1157.5516, 1164.6057, 1165.6611, 1165.6611, 1193.5681, 1277.6617, 1277.6617, 1300.8000, 1307.6257, 1307.6257, 1320.5388, 1334.8374, 1334.8374, 1349.6270, 1351.6090, 1365.5970, 1383.6307, 1420.6680, 1427.7352, 1434.7792, 1448.8667, 1487.6974, 1612.7894, 1618.0076, 1618.0076, 1697.8160, 1697.8160, 1707.7140, 1716.7928, 1732.0361, 1796.8849, 1838.8502, 1993.9021, 2052.0193, 2211.0298, 2283.1060, 2398.9456

Search Parameters

Type of search : Sequence Query
Enzyme : Trypsin
Fixed modifications : Carbamidomethyl (C)
Variable modifications : Oxidation (M)
Mass values : Monoisotopic
Protein Mass : Unrestricted
Peptide Mass Tolerance : ± 100 ppm
Fragment Mass Tolerance: ± 0.3 Da
Max Missed Cleavages : 1
Instrument type : MALDI-TOF-TOF
Query1 (833.4543,1+): <no title>
Query2 (833.4543,1+): Locus:1..14.0.1
Query3 (842.4758,1+): <no title>
Query4 (848.4947,1+): <no title>

PDF-XChange Click to buy NOW! www.docu-track.com

Query5 (850.5049,1+): <no title>
Query6 (853.4832,1+): <no title>
Query7 (855.4396,1+): <no title>
Query8 (864.5046,1+): <no title>
Query9 (865.9906,1+): <no title>
Query10 (868.5154,1+): <no title>
Query11 (868.5154,1+): Locus:1..14.0.12
Query12 (870.5118,1+): <no title>
Query13 (877.0043,1+): <no title>
Query14 (882.5307,1+): <no title>
Query15 (882.5307,1+): Locus:1..14.0.3
Query16 (887.4306,1+): <no title>
Query17 (892.9702,1+): <no title>
Query18 (912.4536,1+): <no title>
Query19 (912.4536,1+): Locus:1..14.0.2
Query20 (951.5852,1+): <no title>
Query21 (969.4808,1+): <no title>
Query22 (973.4983,1+): <no title>
Query23 (996.5558,1+): <no title>
Query24 (1017.6315,1+): Locus:1..14.0.10
Query25 (1017.6315,1+): <no title>
Query26 (1019.6440,1+): <no title>
Query27 (1033.6599,1+): <no title>
Query28 (1045.5276,1+): <no title>
Query29 (1051.6682,1+): <no title>
Query30 (1051.6682,1+): Locus:1..14.0.4
Query31 (1066.4620,1+): <no title>
Query32 (1090.4866,1+): <no title>
Query33 (1131.6697,1+): <no title>
Query34 (1151.6833,1+): <no title>
Query35 (1157.5516,1+): <no title>
Query36 (1164.6057,1+): <no title>
Query37 (1165.6611,1+): <no title>
Query38 (1165.6611,1+): Locus:1..14.0.7
Query39 (1179.5520,1+): <no title>
Query40 (1193.5681,1+): <no title>
Query41 (1277.6617,1+): <no title>
Query42 (1277.6617,1+): Locus:1..14.0.8
Query43 (1300.8000,1+): <no title>
Query44 (1307.6257,1+): <no title>
Query45 (1307.6257,1+): Locus:1..14.0.9
Query46 (1320.5388,1+): <no title>
Query47 (1334.8374,1+): <no title>
Query48 (1334.8374,1+): Locus:1..14.0.6
Query49 (1349.6270,1+): <no title>
Query50 (1351.6090,1+): <no title>
Query51 (1365.5970,1+): <no title>
Query52 (1383.6307,1+): <no title>
Query53 (1420.6680,1+): <no title>
Query54 (1427.7352,1+): <no title>
Query55 (1434.7792,1+): <no title>
Query56 (1448.8667,1+): <no title>
Query57 (1458.6595,1+): <no title>
Query58 (1475.6919,1+): <no title>
Query59 (1487.6974,1+): <no title>
Query60 (1612.7894,1+): <no title>
Query61 (1618.0076,1+): Locus:1..14.0.11
Query62 (1618.0076,1+): <no title>
Query63 (1697.8160,1+): <no title>
Query64 (1697.8160,1+): Locus:1..14.0.5
Query65 (1707.7140,1+): <no title>
Query66 (1716.7928,1+): <no title>
Query67 (1732.0361,1+): <no title>
Query68 (1796.8849,1+): <no title>
Query69 (1838.8502,1+): <no title>
Query70 (1901.1791,1+): <no title>
Query71 (1977.9272,1+): <no title>
Query72 (1993.9021,1+): <no title>
Query73 (2052.0193,1+): <no title>
Query74 (2211.0298,1+): <no title>
Query75 (2283.1060,1+): <no title>
Query76 (2398.9456,1+): <no title>
Query77 (2488.1152,1+): <no title>

Mascot: http://www.matrixscience.com/
