# Supplementary material for: Alpha-2-Macroglobulin as a New Promising Biomarker Improving the Diagnostic Sensitivity of Bovine Paratuberculosis
Source: Front Vet Sci. 2021 Mar 5;8:637716. doi: 10.3389/fvets.2021.637716 (PMC7973028; doi:10.3389/fvets.2021.637716)
Supplement: Supplementary Data 1 — Mascot search results and raw data files for LC-MS/MS analysis for the identification of specific blood proteins in cattle with Johne's disease. [file Data_Sheet_1.zip › mascot/1 Complement C3_2.pdf]

PDF-XChange Click to buy NOW! www.docu-track.com

PDF-XChange Click to buy NOW! www.docu-track.com

MATRIX SCIENCE

MASCOT Search Results

Protein View: A0A4W2D411

Complement C3 OS=Bos indicus x Bos taurus OX=30522 GN=C3 PE=4 SV=1

Database:	Uniprot_bovine
Score:	86
Expect:	0.00032
Nominal mass (M_r):	190190
Calculated pI:	8.36

Sequence similarity is available as an NCBI BLAST search of A0A4W2D411 against nr.

Search parameters

Enzyme:	Trypsin: cuts C-term side of KR unless next residue is P.
Fixed modifications:	Carbamidomethyl (C)
Variable modifications:	Oxidation (M)

Protein sequence coverage: 9%

Matched peptides shown in *bold red*.

1 MGCEAPPLPP PPPPRPQLSA GLRIPCETRD WGRALTLGKG WQPGLPGMTA
51 PRPMLPSGRY SMITPNILRL ESEETVVLEA HGGQGTIQVS VTVHDFPAKK
101 QVLSNENTQL NSNNGYLSTV TIKIPASKEL KSDKGHKFVT VVATFGNVQV
151 EKVVLISLQS GYLFIQTDKT IYTPGSTVLY RVFTVDHKLL PVGQTVFITI
201 ETPDGIPVKR DSKSSQNQFG ILTLSWNIPE LVNMGVWKIK AYYEDSPQQV
251 FSAEFEVKEY VLPSFEVQLE PEEKFYYIDD PDGLKVNIIA RFLYGEQVDG
301 TAFVIFGVQD GDRRISLTHS LTRVPINDGN GEAILKRQVL LNGVQPSRAD
351 ALVGKSIYVS ATVILQSGSD MVEAERTGIP IVTSPYQIHF TKTPKFFKPA
401 MPFDLMVYVT NPDGSPARHI PVVTQGSNVQ SLTQDDGVAK LSINTQNKRD
451 PLTITVRTKK DNIPEGRQAT RTMQALPYNT QGNSNNYLHL SVPRVELKPG
501 ETLNVNFHLR TDPGEQAKIR **YYTYMIMNKG** **K**LLKVGRQYR EPGQDLVVLP
551 LTITSDFIPS FRLVAYYTLI NAKGQREVVA DSVWVDVKDS CMGTLVVKNG
601 GKEEKHHRPG QQITLKIEAD QGARVGLVAV DKGVFVLNKK NKLTQRKIWD
651 VVEK**ADIGCT** **PGSGR**NYAGV FTDAGLTLKT SQGLETQQRA DPQCPQPATR
701 RRR**SVQLMEK** RMDK**AGQYSS** **DLR**KCCEDGM RDNPMKFPCQ RR**AQFILQGD**
751 **ACVK**AFLDCC EYITQLRQQH SRDGALELAR SDLDDDIIPE EDIISR**SQFP**
801 **ESWLWTVIED** **LK**QADKNGIS TK**LMNVFLK**D SITTWEILAV SLSDKK**GICV**
851 **ADPYEVTVMQ** **DFFIDLRLPY** **SVVRNEQVEI** **RAILYNYR**EA ENLK**VRVELL**
901 **YNPAFCSLAT** **AK**KR**HQQTIT** **IPAR**SSVAVP YVIVPLKIGL HEVEVK**AAVY**
951 **NHFISDGVK**K TLKVVPEGVR VNKTVAVRTL NPEHLGQGGV QREEVPAADL
1001 SDQVPDTESE TKILLQGTPV AQMTEDAIDG ERLKHLIQTP SGCGEQNMIG
1051 MTPTVIAVHY LDSTDQWEKF GLEKRQESLE LIRKGYTQQL AFRQKSSAYA
1101 AFQYRPPSTW LTAYVVKVFA LAANLIAIDS KDLCETVKWL ILEKQKPDGI

```
1151 FQEDGPVIHQ EMIGGFRDTR EKDVSLTAFV LIALHEAKDI CEAQVNSLGR
1201 SIAKAGDFLE NHYRELRRPY TVAIAAYALA LLGKLEGDRL TKFLNTAKEK
1251 NRWEEPNQKL YNVEATSYAL LALLARKDYD TTPPVVRWLN EQRYYGGGYG
1301 STQATFMVFQ ALAQYQKDVP DHKELNLDVS IQLPSRNSAV RHRILWESAS
1351 LLRSEETKEN ERFTVKAEGK GQGTLSVVTV YHAKLKGKVS CKKFDLRVSI
1401 RPAPETVKKP QDAKGSMILD ICTKYLGDQD ATMSILDISM MTGFSPDVED
1451 LKTLSTGVDR YISKYEMNRD SNKNTLIIYL DKVSHTVEDC LSFKVHQYFN
1501 VGLIQPGAVK VYSYYNLDET CIRFYHPDKE DGMLSKLCHK DTCRCAEENC
1551 FMHHTEKEVT LEDRLDKACE PGVDYVYKTR LIQKKLEDDF DEYIMVIENI
1601 IKSGSATSSG RTPGWSCGPR LKNVKMRRTR NSARTWPTSQ RTWSSLAAPT
1651 DPTHIHPLIH TVHAIKLHFC STCLLKSGGL
```

Unformatted sequence string: 1680 residues (for pasting into other applications).

Sort peptides by Residue Number Increasing Mass Decreasing Mass

Show predicted peptides also

Query	Start – End	Observed	Mr(expt)	Mr(calc)	ppm	M	Score	Peptic
54	521 – 531	1427.7352	1426.7279	1426.6625	45.9	1		R.YYTY + Oxidat (M)
32	655 – 665	1090.4866	1089.4793	1089.4873	-7.34	0		K.ADIG
5	704 – 710	850.5049	849.4976	849.4266	83.6	0		R.SVQL + Oxidat (M)
23	715 – 723	996.5558	995.5485	995.4672	81.7	0		K.AGQY
49	743 – 754	1349.6270	1348.6197	1348.6809	-45.4	0		R.AQFI
71	797 – 812	1977.9272	1976.9199	1976.9884	-34.6	0		R.SQFF
8	823 – 829	864.5046	863.4973	863.4939	3.99	0		K.LMNV
77	847 – 867	2488.1152	2487.1079	2487.1814	-29.6	0		K.GICV
1	868 – 874	833.4543	832.4470	832.4807	-40.4	0		R.LPYS
2	868 – 874	833.4543	832.4471	832.4807	-40.4	0	29	R.LPYS
16	875 – 881	887.4306	886.4233	886.4508	-31.0	0		R.NEQV
18	882 – 888	912.4536	911.4463	911.4865	-44.0	0		R.AILY
19	882 – 888	912.4536	911.4463	911.4865	-44.0	0	28	R.AILY
73	895 – 912	2052.0193	2051.0120	2051.0874	-36.7	1		K.VRVE
68	897 – 912	1796.8849	1795.8776	1795.9178	-22.4	0		R.VELL
36	915 – 924	1164.6057	1163.5984	1163.6411	-36.7	0		R.HQQT
53	947 – 959	1420.6680	1419.6607	1419.7147	-38.0	0		K.AAVY
4	1453 – 1460	848.4947	847.4874	847.4400	56.0	0		K.TLST

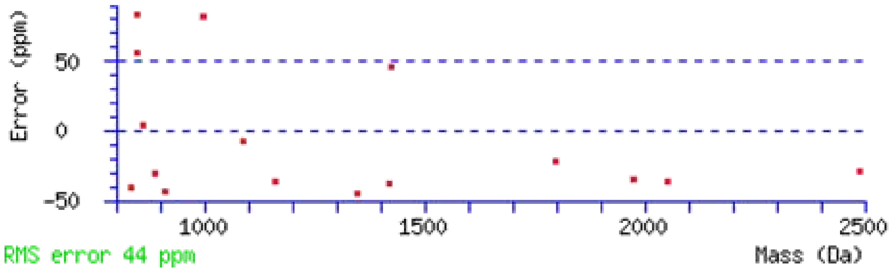

PDF-XChange Click to buy NOW! www.docu-track.com

```
ID   A0A4W2D411_BOBOX        Unreviewed;      1680 AA.
AC   A0A4W2D411;
DT   18-SEP-2019, integrated into UniProtKB/TrEMBL.
DT   18-SEP-2019, sequence version 1.
DT   26-FEB-2020, entry version 5.
DE   SubName: Full=Complement C3 {ECO:0000313|Ensembl:ENSBIXP00000018941};
GN   Name=C3 {ECO:0000313|Ensembl:ENSBIXP00000018941};
OS   Bos indicus x Bos taurus (Hybrid cattle).
OC   Eukaryota; Metazoa; Chordata; Craniata; Vertebrata; Euteleostomi; Mammalia;
OC   Eutheria; Laurasiatheria; Artiodactyla; Ruminantia; Pecora; Bovidae;
OC   Bovinae; Bos.
OX   NCBI_TaxID=30522 {ECO:0000313|Ensembl:ENSBIXP00000018941, ECO:0000313|Prote
RN   [1] {ECO:0000313|Ensembl:ENSBIXP00000018941, ECO:0000313|Proteomes:UP000314
RP   NUCLEOTIDE SEQUENCE [LARGE SCALE GENOMIC DNA].
RA   Low W.Y., Tearle R., Bickhart D.M., Rosen B.D., Koren S., Rhie A.,
RA   Hiendleder S., Phillippy A.M., Smith T.P.L., Williams J.L.;
RT   "Haplotype-resolved cattle genomes.";
RL   Submitted (NOV-2018) to the EMBL/GenBank/DDBJ databases.
RN   [2] {ECO:0000313|Ensembl:ENSBIXP00000018941}
RP   IDENTIFICATION.
RG   Ensembl;
RL   Submitted (JUL-2019) to UniProtKB.
CC   -----------------------------------------------------------------------
CC   Copyrighted by the UniProt Consortium, see https://www.uniprot.org/terms
CC   Distributed under the Creative Commons Attribution (CC BY 4.0) License
CC   -----------------------------------------------------------------------
DR   Ensembl; ENSBIXT00000032285; ENSBIXP00000018941; ENSBIXG00000022699.
DR   Proteomes; UP000314981; Chromosome 7.
DR   GO; GO:0005615; C:extracellular space; IEA:InterPro.
DR   GO; GO:0004866; F:endopeptidase inhibitor activity; IEA:InterPro.
DR   GO; GO:0006956; P:complement activation; IEA:InterPro.
DR   GO; GO:0006954; P:inflammatory response; IEA:InterPro.
DR   CDD; cd00017; ANATO; 1.
DR   Gene3D; 2.40.50.120; -; 1.
DR   Gene3D; 2.60.40.10; -; 2.
DR   Gene3D; 2.60.40.690; -; 1.
DR   InterPro; IPR009048; A-macroglobulin_rcpt-bd.
DR   InterPro; IPR036595; A-macroglobulin_rcpt-bd_sf.
DR   InterPro; IPR011625; A2M_N_BRD.
DR   InterPro; IPR011626; Alpha-macroglobulin_TED.
DR   InterPro; IPR000020; Anaphylatoxin/fibulin.
DR   InterPro; IPR018081; Anaphylatoxin_comp_syst.
DR   InterPro; IPR001840; Anaphylatoxn_comp_syst_dom.
DR   InterPro; IPR041425; C3/4/5_MG1.
DR   InterPro; IPR035711; Complement_C3-like.
DR   InterPro; IPR013783; Ig-like_fold.
DR   InterPro; IPR001599; Macroglobln_a2.
DR   InterPro; IPR019742; MacrogloblnA2_CS.
DR   InterPro; IPR002890; MG2.
DR   InterPro; IPR041555; MG3.
DR   InterPro; IPR040839; MG4.
DR   InterPro; IPR018933; Netrin_module_non-TIMP.
DR   InterPro; IPR008930; Terpenoid_cyclase/PrenylTrfase.
DR   InterPro; IPR008993; TIMP-like_OB-fold.
DR   PANTHER; PTHR11412:SF81; PTHR11412:SF81; 1.
DR   Pfam; PF00207; A2M; 1.
DR   Pfam; PF07703; A2M_BRD; 1.
DR   Pfam; PF07677; A2M_recep; 1.
DR   Pfam; PF01821; ANATO; 1.
DR   Pfam; PF17790; MG1; 1.
DR   Pfam; PF01835; MG2; 1.
DR   Pfam; PF17791; MG3; 1.
DR   Pfam; PF17789; MG4; 1.
DR   Pfam; PF01759; NTR; 1.
DR   Pfam; PF07678; TED_complement; 1.
```

```
DR   PRINTS; PR00004; ANAPHYLATOXN.
DR   SMART; SM01360; A2M; 1.
DR   SMART; SM01359; A2M_N_2; 1.
DR   SMART; SM01361; A2M_recep; 1.
DR   SMART; SM00104; ANATO; 1.
DR   SUPFAM; SSF47686; SSF47686; 1.
DR   SUPFAM; SSF48239; SSF48239; 1.
DR   SUPFAM; SSF49410; SSF49410; 1.
DR   SUPFAM; SSF50242; SSF50242; 1.
DR   PROSITE; PS00477; ALPHA_2_MACROGLOBULIN; 1.
DR   PROSITE; PS01178; ANAPHYLATOXIN_2; 1.
PE   4: Predicted;
KW   Reference proteome {ECO:0000313|Proteomes:UP000314981}.
FT   DOMAIN          725..760
FT                   /note="Anaphylatoxin-like"
FT                   /evidence="ECO:0000259|PROSITE:PS01178"
FT   REGION          1..21
FT                   /note="Disordered"
FT                   /evidence="ECO:0000256|SAM:MobiDB-lite"
FT   COMPBIAS        1..19
FT                   /note="Pro-rich"
FT                   /evidence="ECO:0000256|SAM:MobiDB-lite"
SQ   SEQUENCE   1680 AA;  188712 MW;  8BF7AA0F008ADC2B CRC64;
     MGCEAPPLPP PPPPRPQLSA GLRIPCETRD WGRALTLGKG WQPGLPGMTA PRPMLPSGRY
     SMITPNILRL ESEETVVLEA HGGQGTIQVS VTVHDFPAKK QVLSNENTQL NSNNGYLSTV
     TIKIPASKEL KSDKGHKFVT VVATFGNVQV EKVVLISLQS GYLFIQTDKT IYTPGSTVLY
     RVFTVDHKLL PVGQTVFITI ETPDGIPVKR DSKSSQNQFG ILTLSWNIPE LVNMGVWKIK
     AYYEDSPQQV FSAEFEVKEY VLPSFEVQLE PEEKFYYIDD PDGLKVNIIA RFLYGEQVDG
     TAFVIFGVQD GDRRISLTHS LTRVPINDGN GEAILKRQVL LNGVQPSRAD ALVGKSIYVS
     ATVILQSGSD MVEAERTGIP IVTSPYQIHF TKTPKFFKPA MPFDLMVYVT NPDGSPARHI
     PVVTQGSNVQ SLTQDDGVAK LSINTQNKRD PLTITVRTKK DNIPEGRQAT RTMQALPYNT
     QGNSNNYLHL SVPRVELKPG ETLNVNFHLR TDPGEQAKIR YYTYMIMNKG KLLKVGRQYR
     EPGQDLVVLP LTITSDFIPS FRLVAYYTLI NAKGQREVVA DSVWVDVKDS CMGTLVVKNG
     GKEEKHHRPG QQITLKIEAD QGARVGLVAV DKGVFVLNKK NKLTQRKIWD VVEKADIGCT
     PGSGRNYAGV FTDAGLTLKT SQGLETQQRA DPQCPQPATR RRRSVQLMEK RMDKAGQYSS
     DLRKCCEDGM RDNPMKFPCQ RRAQFILQGD ACVKAFLDCC EYITQLRQQH SRDGALELAR
     SDLDDDIIPE EDIISRSQFP ESWLWTVIED LKQADKNGIS TKLMNVFLKD SITTWEILAV
     SLSDKKGICV ADPYEVTVMQ DFFIDLRLPY SVVRNEQVEI RAILYNYREA ENLKVRVELL
     YNPAFCSLAT AKKRHQQTIT IPARSSVAVP YVIVPLKIGL HEVEVKAAVY NHFISDGVKK
     TLKVVPEGVR VNKTVAVRTL NPEHLGQGGV QREEVPAADL SDQVPDTESE TKILLQGTPV
     AQMTEDAIDG ERLKHLIQTP SGCGEQNMIG MTPTVIAVHY LDSTDQWEKF GLEKRQESLE
     LIRKGYTQQL AFRQKSSAYA AFQYRPPSTW LTAYVVKVFA LAANLIAIDS KDLCETVKWL
     ILEKQKPDGI FQEDGPVIHQ EMIGGFRDTR EKDVSLTAFV LIALHEAKDI CEAQVNSLGR
     SIAKAGDFLE NHYRELRRPY TVAIAAYALA LLGKLEGDRL TKFLNTAKEK NRWEEPNQKL
     YNVEATSYAL LALLARKDYD TTPPVVRWLN EQRYYGGGYG STQATFMVFQ ALAQYQKDVP
     DHKELNLDVS IQLPSRNSAV RHRILWESAS LLRSEETKEN ERFTVKAEGK GQGTLSVVTV
     YHAKLKGKVS CKKFDLRVSI RPAPETVKKP QDAKGSMILD ICTKYLGDQD ATMSILDISM
     MTGFSPDVED LKTLSTGVDR YISKYEMNRD SNKNTLIIYL DKVSHTVEDC LSFKVHQYFN
     VGLIQPGAVK VYSYYNLDET CIRFYHPDKE DGMLSKLCHK DTCRCAEENC FMHHTEKEVT
     LEDRLDKACE PGVDYVYKTR LIQKKLEDDF DEYIMVIENI IKSGSATSSG RTPGWSCGPR
     LKNVKMRRTR NSARTWPTSQ RTWSSLAAPT DPTHIHPLIH TVHAIKLHFC STCLLKSGGL
//
```

Mascot: http://www.matrixscience.com/
