# Supplementary material for: Alpha-2-Macroglobulin as a New Promising Biomarker Improving the Diagnostic Sensitivity of Bovine Paratuberculosis
Source: Front Vet Sci. 2021 Mar 5;8:637716. doi: 10.3389/fvets.2021.637716 (PMC7973028; doi:10.3389/fvets.2021.637716)
Supplement: Supplementary Data 1 — Mascot search results and raw data files for LC-MS/MS analysis for the identification of specific blood proteins in cattle with Johne's disease. [file Data_Sheet_1.zip › mascot/10 Uncharacterized protein_1.pdf]

PDF-XChange Click to buy NOW! www.docu-track.com

PDF-XChange Click to buy NOW! www.docu-track.com

MATRIX SCIENCE

Mascot Search Results

User :
Email :
Search title : BAEDW\2020_3_5\2020_3_5\MSMS 14\M20
Database : Uniprot_bovine bovine_20200316 (134392 sequences; 76244912 residues)
Timestamp : 31 Mar 2020 at 01:08:06 GMT
Warning : A Peptide summary report will usually give a much clearer picture of MS/MS search results.
Top Score : 82 for A0A3Q1M3L6, Uncharacterized protein OS=Bos taurus OX=9913 PE=1 SV=1

Mascot Score Histogram

Protein score is -10*Log(P), where P is the probability that the observed match is a random event.
Protein scores greater than 64 are significant (p<0.05).
Protein scores are derived from ions scores as a non-probabilistic basis for ranking protein hits.

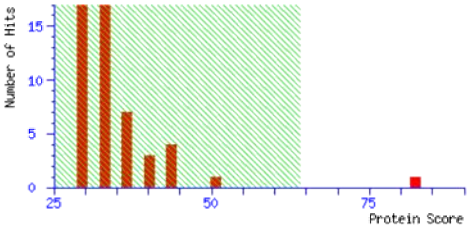

Protein Summary Report

Format As | Protein Summary (deprecated) | Help

Significance threshold p< 0.05 Max. number of hits 30

Preferred taxonomy All entries

Re-Search All | Search Unmatched

Index

	Accession	Mass	Score	Description
1.	A0A3Q1M3L6	41077	82	Uncharacterized protein OS=Bos taurus OX=9913 PE=1 SV=1
2.	Q3SYZ4	57456	51	Aspartate--tRNA ligase, cytoplasmic OS=Bos taurus OX=9913 GN=DARS1 PE=2 SV=1
3.	A0A4W2CKQ7	47880	45	Sorting nexin OS=Bos indicus x Bos taurus OX=30522 GN=SNX5 PE=3 SV=1
4.	A0A4W2DFG4	53946	44	Aspartyl-tRNA synthetase OS=Bos indicus x Bos taurus OX=30522 GN=DARS PE=3 SV=1
5.	A0A4W2HFK9	43131	44	PX domain-containing protein OS=Bos indicus x Bos taurus OX=30522 GN=SNX5 PE=4 SV=1
6.	A0A4W2DFC3	57472	42	Aspartyl-tRNA synthetase OS=Bos indicus x Bos taurus OX=30522 GN=DARS PE=3 SV=1
7.	A0A4W2FGA1	47047	42	Sorting nexin OS=Bos indicus x Bos taurus OX=30522 GN=SNX5 PE=3 SV=1
8.	Q3ZBM5	47075	42	Sorting nexin-5 OS=Bos taurus OX=9913 GN=SNX5 PE=2 SV=1
9.	P46166	4698	39	Beta-defensin 8 OS=Bos taurus OX=9913 GN=DEFB8 PE=1 SV=1
10.	A0A3Q1MFP5	136327	37	Phospholipase OS=Bos taurus OX=9913 GN=PLD1 PE=3 SV=1
11.	A0A4W2FRG9	61477	36	Transmembrane protein 200C OS=Bos indicus x Bos taurus OX=30522 GN=TMEM200C PE=4 SV=1
12.	A0A1R3UGP4	28249	36	Kallikrein G OS=Bos taurus OX=9913 GN=KLNG PE=3 SV=1
13.	A0A4W2F0S3	54184	36	Aspartyl-tRNA synthetase OS=Bos indicus x Bos taurus OX=30522 GN=DARS PE=3 SV=1
14.	A0A4W2HLP0	45410	36	Secernin 1 OS=Bos indicus x Bos taurus OX=30522 GN=SCRN1 PE=4 SV=1
15.	A0A3Q1LXT8	59645	35	Aspartate--tRNA ligase, cytoplasmic OS=Bos taurus OX=9913 GN=DARS1 PE=3 SV=1
16.	A0A4W2C922	39936	35	N(alpha)-acetyltransferase 40, NatD catalytic subunit OS=Bos indicus x Bos taurus OX=30522 GN=NAA40 PE=[illegible]
17.	A0A4W2ETY0	130578	34	Phospholipase OS=Bos indicus x Bos taurus OX=30522 GN=PLD1 PE=3 SV=1
18.	F1MTX7	57472	34	Aspartate--tRNA ligase, cytoplasmic OS=Bos taurus OX=9913 GN=DARS1 PE=3 SV=1
19.	A0A4W2H2Q0	21512	34	Methionine sulfoxide reductase A OS=Bos indicus x Bos taurus OX=30522 GN=MSRA PE=3 SV=1
20.	E1BKP4	84228	33	TANK binding kinase 1 OS=Bos taurus OX=9913 GN=TBK1 PE=4 SV=2
21.	A0A4W2CVJ0	84228	33	TANK binding kinase 1 OS=Bos indicus x Bos taurus OX=30522 GN=TBK1 PE=4 SV=1
22.	A0A4W2HQ46	39881	33	N(alpha)-acetyltransferase 40, NatD catalytic subunit OS=Bos indicus x Bos taurus OX=30522 GN=NAA40 PE=[illegible]
23.	A5PKK1	27850	33	N(alpha)-acetyltransferase 40, NatD catalytic subunit OS=Bos taurus OX=9913 GN=NAA40 PE=2 SV=1
24.	A0A3Q1M909	23483	32	Mitochondrial peptide methionine sulfoxide reductase OS=Bos taurus OX=9913 GN=MSRA PE=1 SV=1
25.	A0A4W2H5U8	23483	32	Methionine sulfoxide reductase A OS=Bos indicus x Bos taurus OX=30522 GN=MSRA PE=3 SV=1
26.	A0A3Q1LVV9	13591	32	RNA_pol_L_2 domain-containing protein OS=Bos taurus OX=9913 PE=4 SV=1
27.	A0A4W2E767	68716	32	Acyl-CoA dehydrogenase very long chain OS=Bos indicus x Bos taurus OX=30522 GN=ACADVL PE=3 SV=1
28.	A0A4W2GRX4	68688	32	Acyl-CoA dehydrogenase very long chain OS=Bos indicus x Bos taurus OX=30522 GN=ACADVL PE=3 SV=1
29.	A0A3Q1LG54	89438	32	Protein phosphatase 1 regulatory subunit 21 OS=Bos taurus OX=9913 GN=PPP1R21 PE=4 SV=1
30.	A5D7H6	88869	32	KLRAQ1 protein OS=Bos taurus OX=9913 GN=PPP1R21 PE=2 SV=1

Results List

1. A0A3Q1M3L6 **Mass:** 41077 **Score:** 82 **Expect:** 0.00079 **Matches:** 9
Uncharacterized protein OS=Bos taurus OX=9913 PE=1 SV=1

Observed	Mr(expt)	Mr(calc)	ppm	Start - End	Miss	Ions	Peptide
1019.6312	1018.6239	1018.5560	66.7	353 - 360	1	---	K.RTIVPDYR.N
1169.5049	1168.4976	1168.5625	-55.54	156 - 165	0	---	R.IQHQDWTGGK.E
1301.6583	1300.6510	1300.7252	-57.00	171 - 182	0	---	K.VHNEGLPAPIVR.T
1301.6583	1300.6511	1300.7252	-56.97	171 - 182	0	---	K.VHNEGLPAPIVR.T
1363.6659	1362.6586	1362.7660	-78.76	343 - 353	1	---	K.WIFSSVVELKR.T
1826.8557	1825.8484	1825.9462	-53.52	193 - 208	0	---	R.EPQVYVLAPPQEELSK.S
2111.9341	2110.9268	2111.0324	-50.02	123 - 140	0	46	K.FSWFVDDVEVNTATTKPR.E
2111.9341	2110.9268	2111.0324	-50.01	123 - 140	0	---	K.FSWFVDDVEVNTATTKPR.E
2753.2139	2752.2066	2752.2877	-29.45	209 - 231	0	---	K.STVSLTCMVTSFYPDYIAVEWQR.N

No match to: 842.4648, 848.4858, 850.0170, 850.4961, 853.4749, 861.0225, 864.5088, 865.9901, 868.5096, 870.5018, 876.9943, 881.9693, 882.5198, 882.5198, 892.9673, 892.9674, 908.9358, 927.4315, 951.5755, 1017.6181, 1017.6181, 1033.6478, 1045.5127, 1051.6508, 1051.6508, 1131.6466, 1147.6801, 1151.6693, 1165.6774, 1165.6774, 1179.5454, 1201.5295, 1334.8121, 1334.8121, 1417.6385, 1433.6392, 1439.6532, 1448.8452, 1475.6703, 1479.7141, 1479.7141, 1567.6675, 1593.7277, 1597.6893, 1597.6893, 1617.9797, 1673.7250, 1690.7394, 1732.0092, 1838.8295, 1848.8546, 1873.8428, 1901.1566, 1939.9556, 1993.8798, 2004.8711, 2015.1777, 2093.9248, 2150.9111, 2150.9111, 2210.9976, 2233.0032, 2283.0698, 2305.0757, 2321.0444, 2351.1042, 3337.6887

2. Q3SYZ4 **Mass:** 57456 **Score:** 51 **Expect:** 1.1 **Matches:** 11

PDF-XChange Click to buy NOW! www.docu-track.com
PDF-XChange Click to buy NOW! www.docu-track.com

Aspartate--tRNA ligase, cytoplasmic OS=Bos taurus OX=9913 GN=DARS1 PE=2 SV=1

Observed	Mr(expt)	Mr(calc)	ppm	Start	End	Miss	Ions	Peptide
1201.5295	1200.5222	1200.6350	-93.95	111	121	0	---	K.ESIVDVEGVVR.K
1301.6583	1300.6510	1300.5250	96.9	255	264	0	---	K.QMCICADFEK.V
1301.6583	1300.6511	1300.5250	96.9	255	264	0	---	K.QMCICADFEK.V
1363.6659	1362.6586	1362.7256	-49.12	320	330	1	---	K.RFQTEIQTVNK.Q
1838.8295	1837.8222	1837.9185	-52.39	394	408	0	---	K.YPLAVRPFYTMPDPR.N + Oxidation (M)
1901.1566	1900.1493	1899.9625	98.3	198	213	1	---	R.LQSGICHPFRETLTNK.G
1939.9556	1938.9483	1938.9912	-22.10	82	99	0	---	R.QQQFNVQALVAVGDHASK.Q
2004.8711	2003.8638	2003.9636	-49.79	265	281	1	---	K.VFCIGPVFRAEDSNTHR.H
2210.9976	2209.9903	2210.1114	-54.77	123	141	1	---	K.VNQKIGSCTQQDVELHVQK.I
2321.0444	2320.0371	2320.2288	-82.59	432	451	1	---	R.IHDPQLVTERALHHGIDLEK.I
2351.1042	2350.0969	2350.0318	27.7	412	431	1	---	K.QSNSYDMFMRGEEILSGAQR.I + 2 Oxidation (M)

No match to: 842.4648, 848.4858, 850.0170, 850.4961, 853.4749, 861.0225, 864.5088, 865.9901, 868.5096, 870.5018, 876.9943, 881.9693, 882.5198, 882.5198, 892.9673, 892.9674, 908.9358, 927.4315, 951.5755, 1017.6181, 1017.6181, 1019.6312, 1033.6478, 1045.5127, 1051.6508, 1051.6508, 1131.6466, 1147.6801, 1151.6693, 1165.6774, 1165.6774, 1169.5049, 1179.5454, 1334.8121, 1334.8121, 1417.6385, 1433.6392, 1439.6532, 1448.8452, 1475.6703, 1479.7141, 1479.7141, 1567.6675, 1593.7277, 1597.6893, 1597.6893, 1617.9797, 1673.7250, 1690.7394, 1732.0092, 1826.8557, 1848.8546, 1873.8428, 1993.8798, 2015.1777, 2093.9248, 2111.9341, 2111.9341, 2150.9111, 2150.9111, 2233.0032, 2283.0698, 2305.0757, 2753.2139, 3337.6887

3. A0A4W2CKQ7 **Mass:** 47880 **Score:** 45 **Expect:** 4.1 **Matches:** 12

Sorting nexin OS=Bos indicus x Bos taurus OX=30522 GN=SNX5 PE=3 SV=1

Observed	Mr(expt)	Mr(calc)	ppm	Start	End	Miss	Ions	Peptide
882.5198	881.5125	881.4317	91.7	119	125	1	---	K.EEFAKMK.Q
882.5198	881.5125	881.4317	91.7	119	125	1	---	K.EEFAKMK.Q
951.5755	950.5682	950.5371	32.7	264	270	1	---	K.KIVYICR.Y
1151.6693	1150.6620	1150.5870	65.2	193	203	0	---	K.SADEVLFSGVK.E
1334.8121	1333.8048	1333.6799	93.7	381	391	0	---	K.NLIEMSELEIK.H + Oxidation (M)
1334.8121	1333.8049	1333.6799	93.7	381	391	0	---	K.NLIEMSELEIK.H + Oxidation (M)
1363.6659	1362.6586	1362.6627	-2.97	285	296	1	---	K.VESRVSSDEDLK.L
1439.6532	1438.6459	1438.7344	-61.47	126	137	0	---	K.QELEAEYLAVFK.K
1593.7277	1592.7204	1592.8046	-52.82	321	334	1	---	K.ALIDYENSNKALDK.A
1617.9797	1616.9724	1616.8621	68.2	289	302	1	---	R.VSSDEDLKLTELLR.Y
1826.8557	1825.8484	1825.9170	-37.54	2	17	1	---	M.AAVPEVLQQQEEDRSK.L
2015.1777	2014.1704	2014.0418	63.9	392	408	1	---	K.HARNNVSLLQSCIDLFK.N

No match to: 842.4648, 848.4858, 850.0170, 850.4961, 853.4749, 861.0225, 864.5088, 865.9901, 868.5096, 870.5018, 876.9943, 881.9693, 892.9673, 892.9674, 908.9358, 927.4315, 1017.6181, 1017.6181, 1019.6312, 1033.6478, 1045.5127, 1051.6508, 1051.6508, 1131.6466, 1147.6801, 1165.6774, 1165.6774, 1169.5049, 1179.5454, 1201.5295, 1301.6583, 1301.6583, 1417.6385, 1433.6392, 1448.8452, 1475.6703, 1479.7141, 1479.7141, 1567.6675, 1597.6893, 1597.6893, 1673.7250, 1690.7394, 1732.0092, 1838.8295, 1848.8546, 1873.8428, 1901.1566, 1939.9556, 1993.8798, 2004.8711, 2093.9248, 2111.9341, 2111.9341, 2150.9111, 2150.9111, 2210.9976, 2233.0032, 2283.0698, 2305.0757, 2321.0444, 2351.1042, 2753.2139, 3337.6887

4. A0A4W2DFG4 **Mass:** 53946 **Score:** 44 **Expect:** 5.2 **Matches:** 10

Aspartyl-tRNA synthetase OS=Bos indicus x Bos taurus OX=30522 GN=DARS PE=3 SV=1

Observed	Mr(expt)	Mr(calc)	ppm	Start	End	Miss	Ions	Peptide
1201.5295	1200.5222	1200.6350	-93.95	80	90	0	---	K.ESIVDVEGVVR.K
1301.6583	1300.6510	1300.5250	96.9	224	233	0	---	K.QMCICADFEK.V
1301.6583	1300.6511	1300.5250	96.9	224	233	0	---	K.QMCICADFEK.V
1363.6659	1362.6586	1362.7256	-49.12	289	299	1	---	K.RFQTEIQTVNK.Q
1838.8295	1837.8222	1837.9185	-52.39	363	377	0	---	K.YPLAVRPFYTMPDPR.N + Oxidation (M)
1939.9556	1938.9483	1938.9469	0.73	27	43	1	---	K.ERYGVSSMIQSQEKPGK.Q + Oxidation (M)
2004.8711	2003.8638	2003.9636	-49.79	234	250	1	---	K.VFCIGPVFRAEDSNTHR.H
2210.9976	2209.9903	2210.1114	-54.77	92	110	1	---	K.VNQKIGSCTQQDVELHVQK.I
2321.0444	2320.0371	2320.2288	-82.59	401	420	1	---	R.IHDPQLVTERALHHGIDLEK.I
2351.1042	2350.0969	2350.0318	27.7	381	400	1	---	K.QSNSYDMFMRGEEILSGAQR.I + 2 Oxidation (M)

No match to: 842.4648, 848.4858, 850.0170, 850.4961, 853.4749, 861.0225, 864.5088, 865.9901, 868.5096, 870.5018, 876.9943, 881.9693, 882.5198, 882.5198, 892.9673, 892.9674, 908.9358, 927.4315, 951.5755, 1017.6181, 1017.6181, 1019.6312, 1033.6478, 1045.5127, 1051.6508, 1051.6508, 1131.6466, 1147.6801, 1151.6693, 1165.6774, 1165.6774, 1169.5049, 1179.5454, 1334.8121, 1334.8121, 1417.6385, 1433.6392, 1439.6532, 1448.8452, 1475.6703, 1479.7141, 1479.7141, 1567.6675, 1593.7277, 1597.6893, 1597.6893, 1617.9797, 1673.7250, 1690.7394, 1732.0092, 1826.8557, 1848.8546, 1873.8428, 1901.1566, 1993.8798, 2015.1777, 2093.9248, 2111.9341, 2111.9341, 2150.9111, 2150.9111, 2233.0032, 2283.0698, 2305.0757, 2753.2139, 3337.6887

5. A0A4W2HFK9 **Mass:** 43131 **Score:** 44 **Expect:** 5.4 **Matches:** 11

PX domain-containing protein OS=Bos indicus x Bos taurus OX=30522 GN=SNX5 PE=4 SV=1

Observed	Mr(expt)	Mr(calc)	ppm	Start	End	Miss	Ions	Peptide
882.5198	881.5125	881.4317	91.7	84	90	1	---	K.EEFAKMK.Q
882.5198	881.5125	881.4317	91.7	84	90	1	---	K.EEFAKMK.Q
1151.6693	1150.6620	1150.5870	65.2	158	168	0	---	K.SADEVLFSGVK.E
1334.8121	1333.8048	1333.6799	93.7	340	350	0	---	K.NLIEMSELEIK.H + Oxidation (M)
1334.8121	1333.8049	1333.6799	93.7	340	350	0	---	K.NLIEMSELEIK.H + Oxidation (M)
1363.6659	1362.6586	1362.6627	-2.97	244	255	1	---	K.VESRVSSDEDLK.L
1439.6532	1438.6459	1438.7344	-61.47	91	102	0	---	K.QELEAEYLAVFK.K
1593.7277	1592.7204	1592.8046	-52.82	280	293	1	---	K.ALIDYENSNKALDK.A
1617.9797	1616.9724	1616.8621	68.2	248	261	1	---	R.VSSDEDLKLTELLR.Y
1826.8557	1825.8484	1825.9170	-37.54	2	17	1	---	M.AAVPEVLQQQEEDRSK.T
2015.1777	2014.1704	2014.0418	63.9	351	367	1	---	K.HARNNVSLLQSCIDLFK.N

No match to: 842.4648, 848.4858, 850.0170, 850.4961, 853.4749, 861.0225, 864.5088, 865.9901, 868.5096, 870.5018, 876.9943, 881.9693, 892.9673, 892.9674, 908.9358, 927.4315, 951.5755, 1017.6181, 1017.6181, 1019.6312, 1033.6478, 1045.5127, 1051.6508, 1051.6508, 1131.6466, 1147.6801, 1165.6774, 1165.6774, 1169.5049, 1179.5454, 1201.5295, 1301.6583, 1301.6583, 1417.6385, 1433.6392, 1448.8452, 1475.6703, 1479.7141, 1479.7141, 1567.6675, 1597.6893, 1597.6893, 1673.7250, 1690.7394, 1732.0092, 1838.8295, 1848.8546, 1873.8428, 1901.1566, 1939.9556, 1993.8798, 2004.8711, 2093.9248, 2111.9341, 2111.9341, 2150.9111, 2150.9111, 2210.9976, 2233.0032, 2283.0698, 2305.0757, 2321.0444, 2351.1042, 2753.2139, 3337.6887

6. A0A4W2DFC3 **Mass:** 57472 **Score:** 42 **Expect:** 7.9 **Matches:** 10

Aspartyl-tRNA synthetase OS=Bos indicus x Bos taurus OX=30522 GN=DARS PE=3 SV=1

Observed	Mr(expt)	Mr(calc)	ppm	Start	End	Miss	Ions	Peptide
1201.5295	1200.5222	1200.6350	-93.95	111	121	0	---	K.ESIVDVEGVVR.K
1301.6583	1300.6510	1300.5250	96.9	255	264	0	---	K.QMCICADFEK.V
1301.6583	1300.6511	1300.5250	96.9	255	264	0	---	K.QMCICADFEK.V
1363.6659	1362.6586	1362.7256	-49.12	320	330	1	---	K.RFQTEIQTVNK.Q
1838.8295	1837.8222	1837.9185	-52.39	394	408	0	---	K.YPLAVRPFYTMPDPR.N + Oxidation (M)
1939.9556	1938.9483	1938.9912	-22.10	82	99	0	---	R.QQQFNVQALVAVGDHASK.Q
2004.8711	2003.8638	2003.9636	-49.79	265	281	1	---	K.VFCIGPVFRAEDSNTHR.H
2210.9976	2209.9903	2210.1114	-54.77	123	141	1	---	K.VNQKIGSCTQQDVELHVQK.I
2321.0444	2320.0371	2320.2288	-82.59	432	451	1	---	R.IHDPQLVTERALHHGIDLEK.I

PDF-XChange Click to buy NOW! www.docu-track.com

PDF-XChange Click to buy NOW! www.docu-track.com

2351.1042	2350.0969	2350.0318	27.7	412 - 431	1	---	K.QSNSYDMFMRGEEILSGAQR.I + 2 Oxidation (M)

No match to: 842.4648, 848.4858, 850.0170, 850.4961, 853.4749, 861.0225, 864.5088, 865.9901, 868.5096, 870.5018, 876.9943, 881.9693, 882.5198, 882.5198, 892.9673, 892.9674, 908.9358, 927.4315, 951.5755, 1017.6181, 1017.6181, 1019.6312, 1033.6478, 1045.5127, 1051.6508, 1051.6508, 1131.6466, 1147.6801, 1151.6693, 1165.6774, 1165.6774, 1169.5049, 1179.5454, 1334.8121, 1334.8121, 1417.6385, 1433.6392, 1439.6532, 1448.8452, 1475.6703, 1479.7141, 1479.7141, 1567.6675, 1593.7277, 1597.6893, 1597.6893, 1617.9797, 1673.7250, 1690.7394, 1732.0092, 1826.8557, 1848.8546, 1873.8428, 1901.1566, 1993.8798, 2015.1777, 2093.9248, 2111.9341, 2111.9341, 2150.9111, 2150.9111, 2233.0032, 2283.0698, 2305.0757, 2753.2139, 3337.6887

7. A0A4W2FGA1 **Mass:** 47047 **Score:** 42 **Expect:** 9.1 **Matches:** 11

Sorting nexin OS=Bos indicus x Bos taurus OX=30522 GN=SNX5 PE=3 SV=1

Observed	Mr(expt)	Mr(calc)	ppm	Start - End	Miss	Ions	Peptide
882.5198	881.5125	881.4317	91.7	119 - 125	1	---	K.EEFAKMK.Q
882.5198	881.5125	881.4317	91.7	119 - 125	1	---	K.EEFAKMK.Q
1151.6693	1150.6620	1150.5870	65.2	193 - 203	0	---	K.SADEVLFSGVK.E
1334.8121	1333.8048	1333.6799	93.7	375 - 385	0	---	K.NLIEMSELEIK.H + Oxidation (M)
1334.8121	1333.8049	1333.6799	93.7	375 - 385	0	---	K.NLIEMSELEIK.H + Oxidation (M)
1363.6659	1362.6586	1362.6627	-2.97	279 - 290	1	---	K.VESRVSSDEDLK.L
1439.6532	1438.6459	1438.7344	-61.47	126 - 137	0	---	K.QELEAEYLAVFK.K
1593.7277	1592.7204	1592.8046	-52.82	315 - 328	1	---	K.ALIDYENSNKALDK.A
1617.9797	1616.9724	1616.8621	68.2	283 - 296	1	---	R.VSSDEDLKLTELLR.Y
1826.8557	1825.8484	1825.9170	-37.54	2 - 17	1	---	M.AAVPEVLQQQEEDRSK.L
2015.1777	2014.1704	2014.0418	63.9	386 - 402	1	---	K.HARNNVSLLQSCIDLFK.N

No match to: 842.4648, 848.4858, 850.0170, 850.4961, 853.4749, 861.0225, 864.5088, 865.9901, 868.5096, 870.5018, 876.9943, 881.9693, 892.9673, 892.9674, 908.9358, 927.4315, 951.5755, 1017.6181, 1017.6181, 1019.6312, 1033.6478, 1045.5127, 1051.6508, 1051.6508, 1131.6466, 1147.6801, 1165.6774, 1165.6774, 1169.5049, 1179.5454, 1201.5295, 1301.6583, 1301.6583, 1417.6385, 1433.6392, 1448.8452, 1475.6703, 1479.7141, 1479.7141, 1567.6675, 1597.6893, 1597.6893, 1673.7250, 1690.7394, 1732.0092, 1838.8295, 1848.8546, 1873.8428, 1901.1566, 1939.9556, 1993.8798, 2004.8711, 2093.9248, 2111.9341, 2111.9341, 2150.9111, 2150.9111, 2210.9976, 2233.0032, 2283.0698, 2305.0757, 2321.0444, 2351.1042, 2753.2139, 3337.6887

8. Q3ZBM5 **Mass:** 47075 **Score:** 42 **Expect:** 9.1 **Matches:** 11

Sorting nexin-5 OS=Bos taurus OX=9913 GN=SNX5 PE=2 SV=1

Observed	Mr(expt)	Mr(calc)	ppm	Start - End	Miss	Ions	Peptide
882.5198	881.5125	881.4317	91.7	119 - 125	1	---	K.EEFAKMK.Q
882.5198	881.5125	881.4317	91.7	119 - 125	1	---	K.EEFAKMK.Q
1151.6693	1150.6620	1150.5870	65.2	193 - 203	0	---	K.SADEVLFSGVK.E
1334.8121	1333.8048	1333.6799	93.7	375 - 385	0	---	K.NLIEMSELEIK.H + Oxidation (M)
1334.8121	1333.8049	1333.6799	93.7	375 - 385	0	---	K.NLIEMSELEIK.H + Oxidation (M)
1363.6659	1362.6586	1362.6627	-2.97	279 - 290	1	---	K.VESRVSSDEDLK.L
1439.6532	1438.6459	1438.7344	-61.47	126 - 137	0	---	K.QELEAEYLAVFK.K
1593.7277	1592.7204	1592.8046	-52.82	315 - 328	1	---	K.ALIDYENSNKALDK.A
1617.9797	1616.9724	1616.8621	68.2	283 - 296	1	---	R.VSSDEDLKLTELLR.Y
1826.8557	1825.8484	1825.9170	-37.54	2 - 17	1	---	M.AAVPEVLQQQEEDRSK.L
2015.1777	2014.1704	2014.0418	63.9	386 - 402	1	---	K.HARNNVSLLQSCIDLFK.N

No match to: 842.4648, 848.4858, 850.0170, 850.4961, 853.4749, 861.0225, 864.5088, 865.9901, 868.5096, 870.5018, 876.9943, 881.9693, 892.9673, 892.9674, 908.9358, 927.4315, 951.5755, 1017.6181, 1017.6181, 1019.6312, 1033.6478, 1045.5127, 1051.6508, 1051.6508, 1131.6466, 1147.6801, 1165.6774, 1165.6774, 1169.5049, 1179.5454, 1201.5295, 1301.6583, 1301.6583, 1417.6385, 1433.6392, 1448.8452, 1475.6703, 1479.7141, 1479.7141, 1567.6675, 1597.6893, 1597.6893, 1673.7250, 1690.7394, 1732.0092, 1838.8295, 1848.8546, 1873.8428, 1901.1566, 1939.9556, 1993.8798, 2004.8711, 2093.9248, 2111.9341, 2111.9341, 2150.9111, 2150.9111, 2210.9976, 2233.0032, 2283.0698, 2305.0757, 2321.0444, 2351.1042, 2753.2139, 3337.6887

9. P46166 **Mass:** 4698 **Score:** 39 **Expect:** 17 **Matches:** 5

Beta-defensin 8 OS=Bos taurus OX=9913 GN=DEFB8 PE=1 SV=1

Observed	Mr(expt)	Mr(calc)	ppm	Start - End	Miss	Ions	Peptide
848.4858	847.4785	847.4375	48.5	12 - 18	0	---	R.GFCVPIR.C
1051.6508	1050.6435	1050.5393	99.2	1 - 8	1	---	-.VRNFVTCR.I
1051.6508	1050.6435	1050.5393	99.2	1 - 8	1	---	-.VRNFVTCR.I
1179.5454	1178.5381	1178.5979	-50.68	3 - 11	1	---	R.NFVTCRINR.G
1690.7394	1689.7321	1689.8113	-46.86	25 - 38	1	---	R.QIGTCLGPQIKCCR.-

No match to: 842.4648, 850.0170, 850.4961, 853.4749, 861.0225, 864.5088, 865.9901, 868.5096, 870.5018, 876.9943, 881.9693, 882.5198, 882.5198, 892.9673, 892.9674, 908.9358, 927.4315, 951.5755, 1017.6181, 1017.6181, 1019.6312, 1033.6478, 1045.5127, 1131.6466, 1147.6801, 1151.6693, 1165.6774, 1165.6774, 1169.5049, 1201.5295, 1301.6583, 1301.6583, 1334.8121, 1334.8121, 1363.6659, 1417.6385, 1433.6392, 1439.6532, 1448.8452, 1475.6703, 1479.7141, 1479.7141, 1567.6675, 1593.7277, 1597.6893, 1597.6893, 1617.9797, 1673.7250, 1732.0092, 1826.8557, 1838.8295, 1848.8546, 1873.8428, 1901.1566, 1939.9556, 1993.8798, 2004.8711, 2015.1777, 2093.9248, 2111.9341, 2111.9341, 2150.9111, 2150.9111, 2210.9976, 2233.0032, 2283.0698, 2305.0757, 2321.0444, 2351.1042, 2753.2139, 3337.6887

10. A0A3Q1MFP5 **Mass:** 136327 **Score:** 37 **Expect:** 26 **Matches:** 13

Phospholipase OS=Bos taurus OX=9913 GN=PLD1 PE=3 SV=1

Observed	Mr(expt)	Mr(calc)	ppm	Start - End	Miss	Ions	Peptide
951.5755	950.5682	950.5371	32.7	806 - 812	1	---	K.IMKPKYR.C + Oxidation (M)
1051.6508	1050.6435	1050.5718	68.2	291 - 298	1	---	K.LLKMPMYR.N
1051.6508	1050.6435	1050.5718	68.3	291 - 298	1	---	K.LLKMPMYR.N
1131.6466	1130.6393	1130.6699	-27.06	1118 - 1127	0	---	R.DFISKPILAK.E
1363.6659	1362.6586	1362.7758	-86.01	283 - 293	1	---	K.QLEDYLTKLLK.M
1448.8452	1447.8379	1447.7030	93.2	776 - 788	0	---	R.MPWHDIASAVHGK.A
1848.8546	1847.8473	1847.9278	-43.56	438 - 454	1	---	R.DHRFGSYAAIQGNTLAK.W
1873.8428	1872.8355	1872.8312	2.31	266 - 280	0	---	R.TSENMIQQEEQFFSR.R
2233.0032	2231.9959	2232.1613	-74.07	813 - 831	1	---	R.CLSYPFLLPKSQTTAHELK.Y
2283.0698	2282.0625	2282.0294	14.5	1024 - 1044	0	---	R.DSEMAVIVQDTETVPSVMDGK.E + 2 Oxidation (M)
2351.1042	2350.0969	2350.0576	16.8	455 - 474	1	---	K.WYVNAKGYFEDVANAMEEAK.E + Oxidation (M)
2753.2139	2752.2066	2752.3259	-43.34	604 - 629	1	---	R.VVGGPSLGSLTAETTESMESLCLRDK.N + Oxidation (M)
3337.6887	3336.6814	3336.6455	10.8	31 - 61	1	---	K.EGNASQACLRQVHSLLPHSTSALGGYSLMPR.Q

No match to: 842.4648, 848.4858, 850.0170, 850.4961, 853.4749, 861.0225, 864.5088, 865.9901, 868.5096, 870.5018, 876.9943, 881.9693, 882.5198, 882.5198, 892.9673, 892.9674, 908.9358, 927.4315, 1017.6181, 1017.6181, 1019.6312, 1033.6478, 1045.5127, 1147.6801, 1151.6693, 1165.6774, 1165.6774, 1169.5049, 1179.5454, 1201.5295, 1301.6583, 1301.6583, 1334.8121, 1334.8121, 1417.6385, 1433.6392, 1439.6532, 1475.6703, 1479.7141, 1479.7141, 1567.6675, 1593.7277, 1597.6893, 1597.6893, 1617.9797, 1673.7250, 1690.7394, 1732.0092, 1826.8557, 1838.8295, 1901.1566, 1939.9556, 1993.8798, 2004.8711, 2015.1777, 2093.9248, 2111.9341, 2111.9341, 2150.9111, 2150.9111, 2210.9976, 2305.0757, 2321.0444

11. A0A4W2FRG9 **Mass:** 61477 **Score:** 36 **Expect:** 31 **Matches:** 10

Transmembrane protein 200C OS=Bos indicus x Bos taurus OX=30522 GN=TMEM200C PE=4 SV=1

Observed	Mr(expt)	Mr(calc)	ppm	Start - End	Miss	Ions	Peptide
870.5018	869.4945	869.4620	37.4	412 - 419	1	---	R.GGARWAPR.E
1147.6801	1146.6728	1146.5629	95.8	100 - 111	0	---	R.VPSVTSSSGGNR.N
1151.6693	1150.6620	1150.5731	77.3	449 - 458	1	---	R.RSTSGLPDYR.A
1201.5295	1200.5222	1200.5887	-55.41	88 - 99	0	---	K.QPPPPPGDSGPR.V

PDF-XChange Click to buy NOW! www.docu-track.com

1417.6385	1416.6312	1416.7070	-53.46	100	113	1	---	R.VPSVTSSSGGNRNR.S
1479.7141	1478.7068	1478.7266	-13.36	416	428	1	---	R.WAPREPEEPAAAR.T
1479.7141	1478.7068	1478.7266	-13.35	416	428	1	---	R.WAPREPEEPAAAR.T
1838.8295	1837.8222	1837.8932	-38.60	375	392	1	---	R.DGAAGAASRGWQRPPGER.G
2233.0032	2231.9959	2232.1360	-62.73	284	307	1	---	R.SGGGGGTPGAASPPDLVSSPRRPR.E
2321.0444	2320.0371	2320.1005	-27.31	571	592	1	---	K.LYMISRSHASGVEDGELESAGI.-

No match to: 842.4648, 848.4858, 850.0170, 850.4961, 853.4749, 861.0225, 864.5088, 865.9901, 868.5096, 876.9943, 881.9693, 882.5198, 882.5198, 892.9673, 892.9674, 908.9358, 927.4315, 951.5755, 1017.6181, 1017.6181, 1019.6312, 1033.6478, 1045.5127, 1051.6508, 1051.6508, 1131.6466, 1165.6774, 1165.6774, 1169.5049, 1179.5454, 1301.6583, 1301.6583, 1334.8121, 1334.8121, 1363.6659, 1433.6392, 1439.6532, 1448.8452, 1475.6703, 1567.6675, 1593.7277, 1597.6893, 1597.6893, 1617.9797, 1673.7250, 1690.7394, 1732.0092, 1826.8557, 1848.8546, 1873.8428, 1901.1566, 1939.9556, 1993.8798, 2004.8711, 2015.1777, 2093.9248, 2111.9341, 2111.9341, 2150.9111, 2150.9111, 2210.9976, 2283.0698, 2305.0757, 2351.1042, 2753.2139, 3337.6887

12. A0A1R3UGP4 **Mass:** 28249 **Score:** 36 **Expect:** 32 **Matches:** 7
Kallikrein G OS=Bos taurus OX=9913 GN=KLNG PE=3 SV=1

Observed	Mr(expt)	Mr(calc)	ppm	Start	End	Miss	Ions	Peptide
848.4858	847.4785	847.4473	36.8	109	115	0	---	R.NDIMLVK.M + Oxidation (M)
951.5755	950.5682	950.4862	86.3	239	245	0	---	K.YVDWIQK.T
1179.5454	1178.5381	1178.6482	-93.36	229	238	1	---	K.KPGVYTKVCK.Y
2015.1777	2014.1704	2013.9908	89.2	88	105	1	---	R.TATKSFPHPDFNNSLPNK.D
2283.0698	2282.0625	2282.0975	-15.31	68	87	1	---	R.YVVHLGAHSLGRQDGCEQTR.T
2305.0757	2304.0684	2304.1793	-48.10	1	21	0	---	-.MMTLQLIMFALVTGHVGGETR.I
2321.0444	2320.0371	2320.1742	-59.07	1	21	0	---	-.MMTLQLIMFALVTGHVGGETR.I + Oxidation (M)

No match to: 842.4648, 850.0170, 850.4961, 853.4749, 861.0225, 864.5088, 865.9901, 868.5096, 870.5018, 876.9943, 881.9693, 882.5198, 882.5198, 892.9673, 892.9674, 908.9358, 927.4315, 1017.6181, 1017.6181, 1019.6312, 1033.6478, 1045.5127, 1051.6508, 1051.6508, 1131.6466, 1147.6801, 1151.6693, 1165.6774, 1169.5049, 1201.5295, 1301.6583, 1301.6583, 1334.8121, 1334.8121, 1363.6659, 1417.6385, 1433.6392, 1439.6532, 1448.8452, 1475.6703, 1479.7141, 1479.7141, 1567.6675, 1593.7277, 1597.6893, 1597.6893, 1617.9797, 1673.7250, 1690.7394, 1732.0092, 1826.8557, 1838.8295, 1848.8546, 1873.8428, 1901.1566, 1939.9556, 1993.8798, 2004.8711, 2093.9248, 2111.9341, 2111.9341, 2150.9111, 2150.9111, 2210.9976, 2233.0032, 2351.1042, 2753.2139, 3337.6887

13. A0A4W2F0S3 **Mass:** 54184 **Score:** 36 **Expect:** 34 **Matches:** 9
Aspartyl-tRNA synthetase OS=Bos indicus x Bos taurus OX=30522 GN=DARS PE=3 SV=1

Observed	Mr(expt)	Mr(calc)	ppm	Start	End	Miss	Ions	Peptide
1201.5295	1200.5222	1200.6350	-93.95	111	121	0	---	K.ESIVDVEGVVR.K
1301.6583	1300.6510	1300.5250	96.9	255	264	0	---	K.QMCICADFEK.V
1301.6583	1300.6511	1300.5250	96.9	255	264	0	---	K.QMCICADFEK.V
1363.6659	1362.6586	1362.7256	-49.12	320	330	1	---	K.RFQTEIQTVNK.Q
1939.9556	1938.9483	1938.9912	-22.10	82	99	0	---	R.QQQFNVQALVAVGDHASK.Q
2004.8711	2003.8638	2003.9636	-49.79	265	281	1	---	K.VFCIGPVFRAEDSNTHR.H
2210.9976	2209.9903	2210.1114	-54.77	123	141	1	---	K.VNQKIGSCTQQDVELHVQK.I
2321.0444	2320.0371	2320.2288	-82.59	405	424	1	---	R.IHDPQLVTERALHHGIDLEK.I
2351.1042	2350.0969	2350.0318	27.7	385	404	1	---	K.QSNSYDMFMRGEEILSGAQR.I + 2 Oxidation (M)

No match to: 842.4648, 848.4858, 850.0170, 850.4961, 853.4749, 861.0225, 864.5088, 865.9901, 868.5096, 870.5018, 876.9943, 881.9693, 882.5198, 882.5198, 892.9673, 892.9674, 908.9358, 927.4315, 951.5755, 1017.6181, 1017.6181, 1019.6312, 1033.6478, 1045.5127, 1051.6508, 1051.6508, 1131.6466, 1147.6801, 1151.6693, 1165.6774, 1165.6774, 1169.5049, 1179.5454, 1334.8121, 1334.8121, 1417.6385, 1433.6392, 1439.6532, 1448.8452, 1475.6703, 1479.7141, 1479.7141, 1567.6675, 1593.7277, 1597.6893, 1597.6893, 1617.9797, 1673.7250, 1690.7394, 1732.0092, 1826.8557, 1838.8295, 1848.8546, 1873.8428, 1901.1566, 1993.8798, 2015.1777, 2093.9248, 2111.9341, 2111.9341, 2150.9111, 2150.9111, 2233.0032, 2283.0698, 2305.0757, 2753.2139, 3337.6887

14. A0A4W2HLP0 **Mass:** 45410 **Score:** 36 **Expect:** 36 **Matches:** 8
Secernin 1 OS=Bos indicus x Bos taurus OX=30522 GN=SCRN1 PE=4 SV=1

Observed	Mr(expt)	Mr(calc)	ppm	Start	End	Miss	Ions	Peptide
1301.6583	1300.6510	1300.6524	-1.04	320	329	1	---	K.EPRFQEKPDR.R
1301.6583	1300.6511	1300.6524	-1.01	320	329	1	---	K.EPRFQEKPDR.R
1417.6385	1416.6312	1416.7296	-69.44	2	13	0	---	M.LSLLPHDLCHGR.S
1439.6532	1438.6459	1438.7105	-44.91	331	341	1	---	R.HELYKAHEWAR.A
2004.8711	2003.8638	2004.0197	-77.80	231	247	1	---	K.QEESITVQTMIDVLRDK.A
2210.9976	2209.9903	2210.1261	-61.45	32	50	0	---	R.VLGSLMLMCTYISINQVPR.T + Oxidation (M)
2283.0698	2282.0625	2282.1940	-57.60	85	105	1	---	R.EPAAETEALLGMDLVRLGLER.G
2351.1042	2350.0969	2350.2323	-57.61	31	50	1	---	R.RVLGSLMLMCTYISINQVPR.T

No match to: 842.4648, 848.4858, 850.0170, 850.4961, 853.4749, 861.0225, 864.5088, 865.9901, 868.5096, 870.5018, 876.9943, 881.9693, 882.5198, 882.5198, 892.9673, 892.9674, 908.9358, 927.4315, 951.5755, 1017.6181, 1017.6181, 1019.6312, 1033.6478, 1045.5127, 1051.6508, 1051.6508, 1131.6466, 1147.6801, 1151.6693, 1165.6774, 1165.6774, 1169.5049, 1179.5454, 1201.5295, 1334.8121, 1334.8121, 1363.6659, 1433.6392, 1448.8452, 1475.6703, 1479.7141, 1479.7141, 1567.6675, 1593.7277, 1597.6893, 1597.6893, 1617.9797, 1673.7250, 1690.7394, 1732.0092, 1826.8557, 1838.8295, 1848.8546, 1873.8428, 1901.1566, 1939.9556, 1993.8798, 2015.1777, 2093.9248, 2111.9341, 2111.9341, 2150.9111, 2150.9111, 2233.0032, 2305.0757, 2321.0444, 2753.2139, 3337.6887

15. A0A3Q1LXT8 **Mass:** 59645 **Score:** 35 **Expect:** 41 **Matches:** 9
Aspartate--tRNA ligase, cytoplasmic OS=Bos taurus OX=9913 GN=DARS1 PE=3 SV=1

Observed	Mr(expt)	Mr(calc)	ppm	Start	End	Miss	Ions	Peptide
1201.5295	1200.5222	1200.6350	-93.95	114	124	0	---	K.ESIVDVEGVVR.K
1301.6583	1300.6510	1300.5250	96.9	258	267	0	---	K.QMCICADFEK.V
1301.6583	1300.6511	1300.5250	96.9	258	267	0	---	K.QMCICADFEK.V
1363.6659	1362.6586	1362.7256	-49.12	323	333	1	---	K.RFQTEIQTVNK.Q
1838.8295	1837.8222	1837.9185	-52.39	397	411	0	---	K.YPLAVRPFYTMPDPR.N + Oxidation (M)
1939.9556	1938.9483	1938.9912	-22.10	85	102	0	---	R.QQQFNVQALVAVGDHASK.Q
2004.8711	2003.8638	2003.9636	-49.79	268	284	1	---	K.VFCIGPVFRAEDSNTHR.H
2210.9976	2209.9903	2210.1114	-54.77	126	144	1	---	K.VNQKIGSCTQQDVELHVQK.I
2351.1042	2350.0969	2350.0318	27.7	415	434	1	---	K.QSNSYDMFMRGEEILSGAQR.I + 2 Oxidation (M)

No match to: 842.4648, 848.4858, 850.0170, 850.4961, 853.4749, 861.0225, 864.5088, 865.9901, 868.5096, 870.5018, 876.9943, 881.9693, 882.5198, 882.5198, 892.9673, 892.9674, 908.9358, 927.4315, 951.5755, 1017.6181, 1017.6181, 1019.6312, 1033.6478, 1045.5127, 1051.6508, 1051.6508, 1131.6466, 1147.6801, 1151.6693, 1165.6774, 1165.6774, 1169.5049, 1179.5454, 1334.8121, 1334.8121, 1417.6385, 1433.6392, 1439.6532, 1448.8452, 1475.6703, 1479.7141, 1479.7141, 1567.6675, 1593.7277, 1597.6893, 1597.6893, 1617.9797, 1673.7250, 1690.7394, 1732.0092, 1826.8557, 1848.8546, 1873.8428, 1901.1566, 1993.8798, 2015.1777, 2093.9248, 2111.9341, 2111.9341, 2150.9111, 2150.9111, 2233.0032, 2283.0698, 2305.0757, 2321.0444, 2753.2139, 3337.6887

16. A0A4W2C922 **Mass:** 39936 **Score:** 35 **Expect:** 42 **Matches:** 9
N(alpha)-acetyltransferase 40, NatD catalytic subunit OS=Bos indicus x Bos taurus OX=30522 GN=NAA40 PE=4 SV=1

Observed	Mr(expt)	Mr(calc)	ppm	Start	End	Miss	Ions	Peptide
853.4749	852.4676	852.4779	-12.08	279	285	0	---	K.VMLTVFK.H + Oxidation (M)
1479.7141	1478.7068	1478.6857	14.3	124	136	1	---	R.LEERAAMDAVCAK.V + Oxidation (M)
1479.7141	1478.7068	1478.6857	14.3	124	136	1	---	R.LEERAAMDAVCAK.V + Oxidation (M)
1567.6675	1566.6602	1566.7460	-54.76	156	168	1	---	K.YDRNGLNVSIECK.R
1597.6893	1596.6820	1596.8089	-79.47	72	85	1	---	R.GACAVRGRPPLQCR.R

PDF-XChange Click to buy NOW! www.docu-track.com

PDF-XChange Click to buy NOW! www.docu-track.com

1597.6893	1596.6821	1596.8089	-79.46	72	85	1	---	R.GACAVRGRPPLQCR.R
1848.8546	1847.8473	1847.9305	-45.03	170	186	0	---	R.VSGLEPATVDWAFDLTK.T
2004.8711	2003.8638	2004.0316	-83.74	169	186	1	---	K.RVSGLEPATVDWAFDLTK.T
2351.1042	2350.0969	2350.1528	-23.79	1	21	1	---	-.MAIDTAWREGGGPWLTSFLAR.H + Oxidation (M)

No match to: 842.4648, 848.4858, 850.0170, 850.4961, 861.0225, 864.5088, 865.9901, 868.5096, 870.5018, 876.9943, 881.9693, 882.5198, 882.5198, 892.9673, 892.9674, 908.9358, 927.4315, 951.5755, 1017.6181, 1017.6181, 1019.6312, 1033.6478, 1045.5127, 1051.6508, 1051.6508, 1131.6466, 1147.6801, 1151.6693, 1165.6774, 1165.6774, 1169.5049, 1179.5454, 1201.5295, 1301.6583, 1301.6583, 1334.8121, 1334.8121, 1363.6659, 1417.6385, 1433.6392, 1439.6532, 1448.8452, 1475.6703, 1593.7277, 1617.9797, 1673.7250, 1690.7394, 1732.0092, 1826.8557, 1838.8295, 1873.8428, 1901.1566, 1939.9556, 1993.8798, 2015.1777, 2093.9248, 2111.9341, 2111.9341, 2150.9111, 2210.9976, 2233.0032, 2283.0698, 2305.0757, 2321.0444, 2753.2139, 3337.6887

17. A0A4W2ETY0 **Mass:** 130578 **Score:** 34 **Expect:** 50 **Matches:** 12

Phospholipase OS=Bos indicus x Bos taurus OX=30522 GN=PLD1 PE=3 SV=1

Observed	Mr(expt)	Mr(calc)	ppm	Start	End	Miss	Ions	Peptide
951.5755	950.5682	950.5371	32.7	806	812	1	---	K.IMKPKYR.C + Oxidation (M)
1051.6508	1050.6435	1050.5718	68.2	291	298	1	---	K.LLKMPMYR.N
1051.6508	1050.6435	1050.5718	68.3	291	298	1	---	K.LLKMPMYR.N
1363.6659	1362.6586	1362.7758	-86.01	283	293	1	---	K.QLEDYLTKLLK.M
1448.8452	1447.8379	1447.7030	93.2	776	788	0	---	R.MPWHDIASAVHGK.A
1848.8546	1847.8473	1847.9278	-43.56	438	454	1	---	R.DHRFGSYAAIQGNTLAK.W
1873.8428	1872.8355	1872.8312	2.31	266	280	0	---	R.TSENMIQQEEQFFSR.R
2233.0032	2231.9959	2232.1613	-74.07	813	831	1	---	R.CLSYPFLLPKSQTTAHELK.Y
2283.0698	2282.0625	2282.0294	14.5	1024	1044	0	---	R.DSEMAVIVQDTETVPSVMDGK.E + 2 Oxidation (M)
2351.1042	2350.0969	2350.0576	16.8	455	474	1	---	K.WYVNAKGYFEDVANAMEEAK.E + Oxidation (M)
2753.2139	2752.2066	2752.3259	-43.34	604	629	1	---	R.VVGGPSLGSLTAETTESMESLCLRDK.N + Oxidation (M)
3337.6887	3336.6814	3336.6455	10.8	31	61	1	---	K.EGNASQACLRQVHSLLPHSTSALGGYSLMPR.Q

No match to: 842.4648, 848.4858, 850.0170, 850.4961, 853.4749, 861.0225, 864.5088, 865.9901, 868.5096, 870.5018, 876.9943, 881.9693, 882.5198, 882.5198, 892.9673, 892.9674, 908.9358, 927.4315, 1017.6181, 1017.6181, 1019.6312, 1033.6478, 1045.5127, 1131.6466, 1147.6801, 1151.6693, 1165.6774, 1165.6774, 1169.5049, 1179.5454, 1201.5295, 1301.6583, 1301.6583, 1334.8121, 1334.8121, 1417.6385, 1433.6392, 1439.6532, 1475.6703, 1479.7141, 1479.7141, 1567.6675, 1593.7277, 1597.6893, 1597.6893, 1617.9797, 1673.7250, 1690.7394, 1732.0092, 1826.8557, 1838.8295, 1901.1566, 1939.9556, 1993.8798, 2004.8711, 2015.1777, 2093.9248, 2111.9341, 2111.9341, 2150.9111, 2150.9111, 2210.9976, 2305.0757, 2321.0444

18. F1MTX7 **Mass:** 57472 **Score:** 34 **Expect:** 51 **Matches:** 9

Aspartate--tRNA ligase, cytoplasmic OS=Bos taurus OX=9913 GN=DARS1 PE=3 SV=1

Observed	Mr(expt)	Mr(calc)	ppm	Start	End	Miss	Ions	Peptide
1201.5295	1200.5222	1200.6350	-93.95	111	121	0	---	K.ESIVDVEGVVR.K
1301.6583	1300.6510	1300.5250	96.9	255	264	0	---	K.QMCICADFEK.V
1301.6583	1300.6511	1300.5250	96.9	255	264	0	---	K.QMCICADFEK.V
1363.6659	1362.6586	1362.7256	-49.12	320	330	1	---	K.RFQTEIQTVNK.Q
1838.8295	1837.8222	1837.9185	-52.39	394	408	0	---	K.YPLAVRPFYTMPDPR.N + Oxidation (M)
1939.9556	1938.9483	1938.9912	-22.10	82	99	0	---	R.QQQFNVQALVAVGDHASK.Q
2004.8711	2003.8638	2003.9636	-49.79	265	281	1	---	K.VFCIGPVFRAEDSNTHR.H
2210.9976	2209.9903	2210.1114	-54.77	123	141	1	---	K.VNQKIGSCTQQDVELHVQK.I
2351.1042	2350.0969	2350.0318	27.7	412	431	1	---	K.QSNSYDMFMRGEEILSGAQR.I + 2 Oxidation (M)

No match to: 842.4648, 848.4858, 850.0170, 850.4961, 853.4749, 861.0225, 864.5088, 865.9901, 868.5096, 870.5018, 876.9943, 881.9693, 882.5198, 882.5198, 892.9673, 892.9674, 908.9358, 927.4315, 951.5755, 1017.6181, 1017.6181, 1019.6312, 1033.6478, 1045.5127, 1051.6508, 1051.6508, 1131.6466, 1147.6801, 1151.6693, 1165.6774, 1165.6774, 1169.5049, 1179.5454, 1334.8121, 1334.8121, 1417.6385, 1433.6392, 1439.6532, 1448.8452, 1475.6703, 1479.7141, 1479.7141, 1567.6675, 1593.7277, 1597.6893, 1597.6893, 1617.9797, 1673.7250, 1690.7394, 1732.0092, 1826.8557, 1848.8546, 1873.8428, 1901.1566, 1993.8798, 2015.1777, 2093.9248, 2111.9341, 2111.9341, 2150.9111, 2150.9111, 2233.0032, 2283.0698, 2305.0757, 2321.0444, 2753.2139, 3337.6887

19. A0A4W2H2Q0 **Mass:** 21512 **Score:** 34 **Expect:** 57 **Matches:** 6

Methionine sulfoxide reductase A OS=Bos indicus x Bos taurus OX=30522 GN=MSRA PE=3 SV=1

Observed	Mr(expt)	Mr(calc)	ppm	Start	End	Miss	Ions	Peptide
850.4961	849.4888	849.4491	46.8	1	7	1	---	-.MLSATRR.A + Oxidation (M)
868.5096	867.5023	867.4563	53.1	71	78	0	---	K.TGHAEVVR.V
1826.8557	1825.8484	1825.9356	-47.75	21	38	1	---	R.MGDSAAKIVSPQEALPGR.K
2150.9111	2149.9038	2150.0137	-51.08	173	193	1	---	K.DPDGYCGLGGTGVSCPLGIKK.-
2150.9111	2149.9039	2150.0137	-51.07	173	193	1	---	K.DPDGYCGLGGTGVSCPLGIKK.-
2321.0444	2320.0371	2320.1699	-57.24	135	154	1	---	K.EDYQKVLSEHGFGLITTDIR.E

No match to: 842.4648, 848.4858, 850.0170, 853.4749, 861.0225, 864.5088, 865.9901, 870.5018, 876.9943, 881.9693, 882.5198, 882.5198, 892.9673, 892.9674, 908.9358, 927.4315, 951.5755, 1017.6181, 1017.6181, 1019.6312, 1033.6478, 1045.5127, 1051.6508, 1051.6508, 1131.6466, 1147.6801, 1151.6693, 1165.6774, 1165.6774, 1169.5049, 1179.5454, 1201.5295, 1301.6583, 1301.6583, 1334.8121, 1334.8121, 1363.6659, 1417.6385, 1433.6392, 1439.6532, 1448.8452, 1475.6703, 1479.7141, 1479.7141, 1567.6675, 1593.7277, 1597.6893, 1597.6893, 1617.9797, 1673.7250, 1690.7394, 1732.0092, 1838.8295, 1848.8546, 1873.8428, 1901.1566, 1939.9556, 1993.8798, 2004.8711, 2015.1777, 2093.9248, 2111.9341, 2111.9341, 2210.9976, 2233.0032, 2283.0698, 2305.0757, 2351.1042, 2753.2139, 3337.6887

20. E1BKP4 **Mass:** 84228 **Score:** 33 **Expect:** 64 **Matches:** 11

TANK binding kinase 1 OS=Bos taurus OX=9913 GN=TBK1 PE=4 SV=2

Observed	Mr(expt)	Mr(calc)	ppm	Start	End	Miss	Ions	Peptide
850.4961	849.4888	849.4345	64.0	155	162	0	---	K.LTDFGAAR.E
864.5088	863.5015	863.4753	30.4	31	38	0	---	K.TGDLFAIK.V
927.4315	926.4242	926.4531	-31.20	230	236	1	---	R.NKEVMYK.I + Oxidation (M)
1151.6693	1150.6620	1150.6135	42.2	588	596	1	---	K.QKLYYHATK.A
1169.5049	1168.4976	1168.5547	-48.82	714	724	0	---	R.FGSLTMDGGLR.N + Oxidation (M)
1201.5295	1200.5222	1200.4969	21.1	406	416	0	---	R.YDLDGDASMAK.A + Oxidation (M)
1593.7277	1592.7204	1592.8596	-87.39	461	473	1	---	K.KTEVVITLDFCIR.N
1690.7394	1689.7321	1689.7781	-27.19	402	416	1	---	K.VHPRYDLDGDASMAK.A + Oxidation (M)
1848.8546	1847.8473	1847.9788	-71.15	128	143	1	---	R.ENGIVHRDIKPGNIMR.V
1901.1566	1900.1493	1900.0921	30.1	385	401	1	---	R.EPLSTIGLIYEKISLPK.V
2305.0757	2304.0684	2304.2172	-64.58	505	525	1	---	K.LLRLSSSQGTIETSLQDIESK.L

No match to: 842.4648, 848.4858, 850.0170, 853.4749, 861.0225, 865.9901, 868.5096, 870.5018, 876.9943, 881.9693, 882.5198, 882.5198, 892.9673, 892.9674, 908.9358, 951.5755, 1017.6181, 1017.6181, 1019.6312, 1033.6478, 1045.5127, 1051.6508, 1051.6508, 1131.6466, 1147.6801, 1165.6774, 1165.6774, 1179.5454, 1301.6583, 1301.6583, 1334.8121, 1334.8121, 1363.6659, 1417.6385, 1433.6392, 1439.6532, 1448.8452, 1475.6703, 1479.7141, 1479.7141, 1567.6675, 1597.6893, 1597.6893, 1617.9797, 1673.7250, 1732.0092, 1826.8557, 1838.8295, 1873.8428, 1939.9556, 1993.8798, 2004.8711, 2015.1777, 2093.9248, 2111.9341, 2111.9341, 2150.9111, 2150.9111, 2210.9976, 2233.0032, 2283.0698, 2321.0444, 2351.1042, 2753.2139, 3337.6887

21. A0A4W2CVJ0 **Mass:** 84228 **Score:** 33 **Expect:** 64 **Matches:** 11

TANK binding kinase 1 OS=Bos indicus x Bos taurus OX=30522 GN=TBK1 PE=4 SV=1

Observed	Mr(expt)	Mr(calc)	ppm	Start	End	Miss	Ions	Peptide
850.4961	849.4888	849.4345	64.0	155	162	0	---	K.LTDFGAAR.E
864.5088	863.5015	863.4753	30.4	31	38	0	---	K.TGDLFAIK.V
927.4315	926.4242	926.4531	-31.20	230	236	1	---	R.NKEVMYK.I + Oxidation (M)

PDF-XChange Click to buy NOW! www.docu-track.com

1151.6693	1150.6620	1150.6135	42.2	588 -	596	1	---	K.QKLYYHATK.A
1169.5049	1168.4976	1168.5547	-48.82	714 -	724	0	---	R.FGSLTMDGGLR.N + Oxidation (M)
1201.5295	1200.5222	1200.4969	21.1	406 -	416	0	---	R.YDLDGDASMAK.A + Oxidation (M)
1593.7277	1592.7204	1592.8596	-87.39	461 -	473	1	---	K.KTEVVITLDFCIR.N
1690.7394	1689.7321	1689.7781	-27.19	402 -	416	1	---	K.VHPRYDLDGDASMAK.A + Oxidation (M)
1848.8546	1847.8473	1847.9788	-71.15	128 -	143	1	---	R.ENGIVHRDIKPGNIMR.V
1901.1566	1900.1493	1900.0921	30.1	385 -	401	1	---	R.EPLSTIGLIYEKISLPK.V
2305.0757	2304.0684	2304.2172	-64.58	505 -	525	1	---	K.LLRLSSSQGTIETSLQDIESK.L

No match to: 842.4648, 848.4858, 850.0170, 853.4749, 861.0225, 865.9901, 868.5096, 870.5018, 876.9943, 881.9693, 882.5198, 882.5198, 892.9673, 892.9674, 908.9358, 951.5755, 1017.6181, 1017.6181, 1019.6312, 1033.6478, 1045.5127, 1051.6508, 1051.6508, 1131.6466, 1147.6801, 1165.6774, 1165.6774, 1179.5454, 1301.6583, 1301.6583, 1334.8121, 1334.8121, 1363.6659, 1417.6385, 1433.6392, 1439.6532, 1448.8452, 1475.6703, 1479.7141, 1479.7141, 1567.6675, 1597.6893, 1597.6893, 1617.9797, 1673.7250, 1732.0092, 1826.8557, 1838.8295, 1873.8428, 1939.9556, 1993.8798, 2004.8711, 2015.1777, 2093.9248, 2111.9341, 2111.9341, 2150.9111, 2150.9111, 2210.9976, 2233.0032, 2283.0698, 2321.0444, 2351.1042, 2753.2139, 3337.6887

22. A0A4W2HQ46 **Mass:** 39881 **Score:** 33 **Expect:** 71 **Matches:** 9
N(alpha)-acetyltransferase 40, NatD catalytic subunit OS=Bos indicus x Bos taurus OX=30522 GN=NAA40 PE=4 SV=1

Observed	Mr(expt)	Mr(calc)	ppm	Start	End	Miss	Ions	Peptide
853.4749	852.4676	852.4779	-12.08	279 -	285	0	---	K.VMLTVFK.H + Oxidation (M)
1479.7141	1478.7068	1478.6857	14.3	124 -	136	1	---	R.LEERAAMDAVCAK.V + Oxidation (M)
1479.7141	1478.7068	1478.6857	14.3	124 -	136	1	---	R.LEERAAMDAVCAK.V + Oxidation (M)
1567.6675	1566.6602	1566.7460	-54.76	156 -	168	1	---	K.YDRNGLNVSIECK.R
1597.6893	1596.6820	1596.8089	-79.47	72 -	85	1	---	R.GACAVRGRPPLQCR.R
1597.6893	1596.6821	1596.8089	-79.46	72 -	85	1	---	R.GACAVRGRPPLQCR.R
1848.8546	1847.8473	1847.9305	-45.03	170 -	186	0	---	R.VSGLEPATVDWAFDLTK.T
2004.8711	2003.8638	2004.0316	-83.74	169 -	186	1	---	K.RVSGLEPATVDWAFDLTK.T
2351.1042	2350.0969	2350.1627	-28.00	170 -	190	1	---	R.VSGLEPATVDWAFDLTKTNMR.T

No match to: 842.4648, 848.4858, 850.0170, 850.4961, 861.0225, 864.5088, 865.9901, 868.5096, 870.5018, 876.9943, 881.9693, 882.5198, 882.5198, 892.9673, 892.9674, 908.9358, 927.4315, 951.5755, 1017.6181, 1017.6181, 1019.6312, 1033.6478, 1045.5127, 1051.6508, 1051.6508, 1131.6466, 1147.6801, 1165.6774, 1165.6774, 1169.5049, 1179.5454, 1201.5295, 1301.6583, 1301.6583, 1334.8121, 1334.8121, 1363.6659, 1417.6385, 1433.6392, 1439.6532, 1448.8452, 1475.6703, 1593.7277, 1617.9797, 1673.7250, 1690.7394, 1732.0092, 1826.8557, 1838.8295, 1873.8428, 1901.1566, 1939.9556, 1993.8798, 2015.1777, 2093.9248, 2111.9341, 2111.9341, 2150.9111, 2150.9111, 2210.9976, 2233.0032, 2283.0698, 2305.0757, 2321.0444, 2753.2139, 3337.6887

23. A5PKK1 **Mass:** 27850 **Score:** 33 **Expect:** 72 **Matches:** 7
N(alpha)-acetyltransferase 40, NatD catalytic subunit OS=Bos taurus OX=9913 GN=NAA40 PE=2 SV=1

Observed	Mr(expt)	Mr(calc)	ppm	Start	End	Miss	Ions	Peptide
853.4749	852.4676	852.4779	-12.08	171 -	177	0	---	K.VMLTVFK.H + Oxidation (M)
1479.7141	1478.7068	1478.6857	14.3	16 -	28	1	---	R.LEERAAMDAVCAK.V + Oxidation (M)
1479.7141	1478.7068	1478.6857	14.3	16 -	28	1	---	R.LEERAAMDAVCAK.V + Oxidation (M)
1567.6675	1566.6602	1566.7460	-54.76	48 -	60	1	---	K.YDRNGLNVSIECK.R
1848.8546	1847.8473	1847.9305	-45.03	62 -	78	0	---	R.VSGLEPATVDWAFDLTK.T
2004.8711	2003.8638	2004.0316	-83.74	61 -	78	1	---	K.RVSGLEPATVDWAFDLTK.T
2351.1042	2350.0969	2350.1627	-28.00	62 -	82	1	---	R.VSGLEPATVDWAFDLTKTNMR.T

No match to: 842.4648, 848.4858, 850.0170, 850.4961, 861.0225, 864.5088, 865.9901, 868.5096, 870.5018, 876.9943, 881.9693, 882.5198, 882.5198, 892.9673, 892.9674, 908.9358, 927.4315, 951.5755, 1017.6181, 1017.6181, 1019.6312, 1033.6478, 1045.5127, 1051.6508, 1051.6508, 1131.6466, 1147.6801, 1151.6693, 1165.6774, 1165.6774, 1169.5049, 1179.5454, 1201.5295, 1301.6583, 1301.6583, 1334.8121, 1334.8121, 1363.6659, 1417.6385, 1433.6392, 1439.6532, 1448.8452, 1475.6703, 1593.7277, 1597.6893, 1597.6893, 1617.9797, 1673.7250, 1690.7394, 1732.0092, 1826.8557, 1838.8295, 1873.8428, 1901.1566, 1939.9556, 1993.8798, 2015.1777, 2093.9248, 2111.9341, 2111.9341, 2150.9111, 2150.9111, 2210.9976, 2233.0032, 2283.0698, 2305.0757, 2321.0444, 2753.2139, 3337.6887

24. A0A3Q1M909 **Mass:** 23483 **Score:** 32 **Expect:** 81 **Matches:** 6
Mitochondrial peptide methionine sulfoxide reductase OS=Bos taurus OX=9913 GN=MSRA PE=1 SV=1

Observed	Mr(expt)	Mr(calc)	ppm	Start	End	Miss	Ions	Peptide
850.4961	849.4888	849.4491	46.8	1 -	7	1	---	-.MLSATRR.A + Oxidation (M)
868.5096	867.5023	867.4563	53.1	88 -	95	0	---	K.TGHAEVVR.V
1826.8557	1825.8484	1825.9356	-47.75	21 -	38	1	---	R.MGDSAAKIVSPQEALPGR.K
2150.9111	2149.9038	2150.0137	-51.08	190 -	210	1	---	K.DPDGYCGLGGTGVSCPLGIKK.-
2150.9111	2149.9039	2150.0137	-51.07	190 -	210	1	---	K.DPDGYCGLGGTGVSCPLGIKK.-
2321.0444	2320.0371	2320.1699	-57.24	152 -	171	1	---	K.EDYQKVLSEHGFGLITTDIR.E

No match to: 842.4648, 848.4858, 850.0170, 853.4749, 861.0225, 864.5088, 865.9901, 870.5018, 876.9943, 881.9693, 882.5198, 882.5198, 892.9673, 892.9674, 908.9358, 927.4315, 951.5755, 1017.6181, 1017.6181, 1019.6312, 1033.6478, 1045.5127, 1051.6508, 1051.6508, 1131.6466, 1147.6801, 1151.6693, 1165.6774, 1165.6774, 1169.5049, 1179.5454, 1201.5295, 1301.6583, 1301.6583, 1334.8121, 1334.8121, 1363.6659, 1417.6385, 1433.6392, 1439.6532, 1448.8452, 1475.6703, 1479.7141, 1479.7141, 1567.6675, 1593.7277, 1597.6893, 1597.6893, 1617.9797, 1673.7250, 1690.7394, 1732.0092, 1838.8295, 1848.8546, 1873.8428, 1901.1566, 1939.9556, 1993.8798, 2004.8711, 2015.1777, 2093.9248, 2111.9341, 2111.9341, 2210.9976, 2233.0032, 2283.0698, 2305.0757, 2351.1042, 2753.2139, 3337.6887

25. A0A4W2H5U8 **Mass:** 23483 **Score:** 32 **Expect:** 81 **Matches:** 6
Methionine sulfoxide reductase A OS=Bos indicus x Bos taurus OX=30522 GN=MSRA PE=3 SV=1

Observed	Mr(expt)	Mr(calc)	ppm	Start	End	Miss	Ions	Peptide
850.4961	849.4888	849.4491	46.8	1 -	7	1	---	-.MLSATRR.A + Oxidation (M)
868.5096	867.5023	867.4563	53.1	88 -	95	0	---	K.TGHAEVVR.V
1826.8557	1825.8484	1825.9356	-47.75	21 -	38	1	---	R.MGDSAAKIVSPQEALPGR.K
2150.9111	2149.9038	2150.0137	-51.08	190 -	210	1	---	K.DPDGYCGLGGTGVSCPLGIKK.-
2150.9111	2149.9039	2150.0137	-51.07	190 -	210	1	---	K.DPDGYCGLGGTGVSCPLGIKK.-
2321.0444	2320.0371	2320.1699	-57.24	152 -	171	1	---	K.EDYQKVLSEHGFGLITTDIR.E

No match to: 842.4648, 848.4858, 850.0170, 853.4749, 861.0225, 864.5088, 865.9901, 870.5018, 876.9943, 881.9693, 882.5198, 882.5198, 892.9673, 892.9674, 908.9358, 927.4315, 951.5755, 1017.6181, 1017.6181, 1019.6312, 1033.6478, 1045.5127, 1051.6508, 1051.6508, 1131.6466, 1147.6801, 1151.6693, 1165.6774, 1165.6774, 1169.5049, 1179.5454, 1201.5295, 1301.6583, 1301.6583, 1334.8121, 1334.8121, 1363.6659, 1417.6385, 1433.6392, 1439.6532, 1448.8452, 1475.6703, 1479.7141, 1479.7141, 1567.6675, 1593.7277, 1597.6893, 1597.6893, 1617.9797, 1673.7250, 1690.7394, 1732.0092, 1838.8295, 1848.8546, 1873.8428, 1901.1566, 1939.9556, 1993.8798, 2004.8711, 2015.1777, 2093.9248, 2111.9341, 2111.9341, 2210.9976, 2233.0032, 2283.0698, 2305.0757, 2351.1042, 2753.2139, 3337.6887

26. A0A3Q1LVV9 **Mass:** 13591 **Score:** 32 **Expect:** 85 **Matches:** 6
RNA_pol_L_2 domain-containing protein OS=Bos taurus OX=9913 PE=4 SV=1

Observed	Mr(expt)	Mr(calc)	ppm	Start	End	Miss	Ions	Peptide
1033.6478	1032.6405	1032.6305	9.74	57 -	64	1	---	K.VRRPLQHK.T
1045.5127	1044.5054	1044.5426	-35.62	110 -	119	0	---	K.MSGGLHLALF.-
1334.8121	1333.8048	1333.7466	43.6	79 -	90	1	---	R.RLPPVPSQTSPR.N
1334.8121	1333.8049	1333.7466	43.7	79 -	90	1	---	R.RLPPVPSQTSPR.N
1363.6659	1362.6586	1362.7408	-60.31	47 -	58	1	---	K.APQVQFAGYKVR.R
1690.7394	1689.7321	1689.8798	-87.39	32 -	46	0	---	K.ENHTLGNISHSQLLK.A

PDF-XChange Click to buy NOW! www.docu-track.com
PDF-XChange Click to buy NOW! www.docu-track.com

No match to: 842.4648, 848.4858, 850.0170, 850.4961, 853.4749, 861.0225, 864.5088, 865.9901, 868.5096, 870.5018, 876.9943, 881.9693, 882.5198, 882.5198, 892.9673, 892.9674, 908.9358, 927.4315, 951.5755, 1017.6181, 1017.6181, 1051.6508, 1051.6508, 1131.6466, 1147.6801, 1151.6693, 1165.6774, 1165.6774, 1169.5049, 1179.5454, 1201.5295, 1301.6583, 1301.6583, 1417.6385, 1433.6392, 1439.6532, 1448.8452, 1475.6703, 1479.7141, 1479.7141, 1567.6675, 1593.7277, 1597.6893, 1597.6893, 1617.9797, 1673.7250, 1732.0092, 1826.8557, 1838.8295, 1848.8546, 1873.8428, 1901.1566, 1939.9556, 1993.8798, 2004.8711, 2015.1777, 2093.9248, 2111.9341, 2111.9341, 2150.9111, 2150.9111, 2210.9976, 2233.0032, 2283.0698, 2305.0757, 2321.0444, 2351.1042, 2753.2139, 3337.6887

27. A0A4W2E767 **Mass:** 68716 **Score:** 32 **Expect:** 85 **Matches:** 9

Acyl-CoA dehydrogenase very long chain OS=Bos indicus x Bos taurus OX=30522 GN=ACADVL PE=3 SV=1

Observed	Mr(expt)	Mr(calc)	ppm	Start	End	Miss	Ions	Peptide
848.4858	847.4785	847.5167	-45.09	166	173	0	---	K.GILLFGTK.A
853.4749	852.4676	852.4202	55.6	337	344	0	---	K.AVDHAANR.T
870.5018	869.4945	869.4971	-2.93	82	89	0	---	K.ELVGPVTR.F
1151.6693	1150.6620	1150.5540	93.9	106	115	0	---	R.VEETTMQGLK.E + Oxidation (M)
1169.5049	1168.4976	1168.5400	-36.30	90	99	0	---	R.FFEEVNDAAK.N
1417.6385	1416.6312	1416.7460	-81.04	243	256	1	---	K.TPVTDTATGAVKEK.I
1593.7277	1592.7204	1592.8675	-92.31	435	447	1	---	R.IFRIFEGTNDILR.L
1848.8546	1847.8473	1847.7926	29.6	407	422	0	---	K.VTDECIQIMGGMGFMK.E + 2 Oxidation (M)
1873.8428	1872.8355	1872.9040	-36.56	278	294	1	---	K.MGIKASNTAEVYFDGVR.V + Oxidation (M)

No match to: 842.4648, 850.0170, 850.4961, 861.0225, 864.5088, 865.9901, 868.5096, 876.9943, 881.9693, 882.5198, 882.5198, 892.9673, 892.9674, 908.9358, 927.4315, 951.5755, 1017.6181, 1017.6181, 1019.6312, 1033.6478, 1045.5127, 1051.6508, 1051.6508, 1131.6466, 1147.6801, 1165.6774, 1165.6774, 1179.5454, 1201.5295, 1301.6583, 1301.6583, 1334.8121, 1334.8121, 1363.6659, 1433.6392, 1439.6532, 1448.8452, 1475.6703, 1479.7141, 1479.7141, 1567.6675, 1597.6893, 1597.6893, 1617.9797, 1673.7250, 1690.7394, 1732.0092, 1826.8557, 1838.8295, 1901.1566, 1939.9556, 1993.8798, 2004.8711, 2015.1777, 2093.9248, 2111.9341, 2111.9341, 2150.9111, 2150.9111, 2210.9976, 2233.0032, 2283.0698, 2305.0757, 2321.0444, 2351.1042, 2753.2139, 3337.6887

28. A0A4W2GRX4 **Mass:** 68688 **Score:** 32 **Expect:** 85 **Matches:** 9

Acyl-CoA dehydrogenase very long chain OS=Bos indicus x Bos taurus OX=30522 GN=ACADVL PE=3 SV=1

Observed	Mr(expt)	Mr(calc)	ppm	Start	End	Miss	Ions	Peptide
848.4858	847.4785	847.5167	-45.09	166	173	0	---	K.GILLFGTK.A
853.4749	852.4676	852.4202	55.6	337	344	0	---	K.AVDHAANR.T
870.5018	869.4945	869.4971	-2.93	82	89	0	---	K.ELVGPVTR.F
1151.6693	1150.6620	1150.5540	93.9	106	115	0	---	R.VEETTMQGLK.E + Oxidation (M)
1169.5049	1168.4976	1168.5400	-36.30	90	99	0	---	R.FFEEVNDAAK.N
1417.6385	1416.6312	1416.7460	-81.04	243	256	1	---	K.TPVTDTATGAVKEK.I
1593.7277	1592.7204	1592.8675	-92.31	435	447	1	---	R.IFRIFEGTNDILR.L
1848.8546	1847.8473	1847.7926	29.6	407	422	0	---	K.VTDECIQIMGGMGFMK.E + 2 Oxidation (M)
1873.8428	1872.8355	1872.9040	-36.56	278	294	1	---	K.MGIKASNTAEVYFDGVR.V + Oxidation (M)

No match to: 842.4648, 850.0170, 850.4961, 861.0225, 864.5088, 865.9901, 868.5096, 876.9943, 881.9693, 882.5198, 882.5198, 892.9673, 892.9674, 908.9358, 927.4315, 951.5755, 1017.6181, 1017.6181, 1019.6312, 1033.6478, 1045.5127, 1051.6508, 1051.6508, 1131.6466, 1147.6801, 1165.6774, 1165.6774, 1179.5454, 1201.5295, 1301.6583, 1301.6583, 1334.8121, 1334.8121, 1363.6659, 1433.6392, 1439.6532, 1448.8452, 1475.6703, 1479.7141, 1479.7141, 1567.6675, 1597.6893, 1597.6893, 1617.9797, 1673.7250, 1690.7394, 1732.0092, 1826.8557, 1838.8295, 1901.1566, 1939.9556, 1993.8798, 2004.8711, 2015.1777, 2093.9248, 2111.9341, 2111.9341, 2150.9111, 2150.9111, 2210.9976, 2233.0032, 2283.0698, 2305.0757, 2321.0444, 2351.1042, 2753.2139, 3337.6887

29. A0A3Q1LG54 **Mass:** 89438 **Score:** 32 **Expect:** 91 **Matches:** 14

Protein phosphatase 1 regulatory subunit 21 OS=Bos taurus OX=9913 GN=PPP1R21 PE=4 SV=1

Observed	Mr(expt)	Mr(calc)	ppm	Start	End	Miss	Ions	Peptide
842.4648	841.4575	841.5134	-66.34	70	76	1	---	R.NLQLAKR.V
853.4749	852.4676	852.4453	26.1	139	145	0	---	K.HVEAELR.S
1033.6478	1032.6405	1032.5451	92.4	187	195	1	---	K.SQTLEKEAK.E
1131.6466	1130.6393	1130.5931	40.9	763	771	1	---	K.QREEIDTLK.M
1147.6801	1146.6728	1146.6357	32.4	553	562	1	---	R.RVLLSSTESR.E
1165.6774	1164.6701	1164.5696	86.3	713	722	1	---	K.SKEALTEEMK.V
1165.6774	1164.6701	1164.5696	86.3	713	722	1	---	K.SKEALTEEMK.V
1301.6583	1300.6510	1300.6623	-8.65	34	46	0	---	K.GVVDEQANSAALK.E
1301.6583	1300.6511	1300.6623	-8.63	34	46	0	---	K.GVVDEQANSAALK.E
1417.6385	1416.6312	1416.7395	-76.41	199	209	1	---	R.LRTEECQLQLK.N
1567.6675	1566.6602	1566.7613	-64.50	693	705	1	---	K.SVHFYAECRALSK.R
1617.9797	1616.9724	1616.8734	61.3	608	623	1	---	K.LKSTSSGQVVGVAQEK.A
2004.8711	2003.8638	2003.9332	-34.64	508	525	0	---	K.TGSGFISPLSAECMLQYK.K + Oxidation (M)
2015.1777	2014.1704	2014.0346	67.4	343	358	1	---	K.LKTFSEHLTSYICFLR.K

No match to: 848.4858, 850.0170, 850.4961, 861.0225, 864.5088, 865.9901, 868.5096, 870.5018, 876.9943, 881.9693, 882.5198, 882.5198, 892.9673, 892.9674, 908.9358, 927.4315, 951.5755, 1017.6181, 1019.6312, 1045.5127, 1051.6508, 1051.6508, 1151.6693, 1169.5049, 1179.5454, 1201.5295, 1334.8121, 1334.8121, 1363.6659, 1433.6392, 1439.6532, 1448.8452, 1475.6703, 1479.7141, 1479.7141, 1593.7277, 1597.6893, 1597.6893, 1673.7250, 1690.7394, 1732.0092, 1826.8557, 1838.8295, 1848.8546, 1873.8428, 1901.1566, 1939.9556, 1993.8798, 2093.9248, 2111.9341, 2111.9341, 2150.9111, 2150.9111, 2210.9976, 2233.0032, 2283.0698, 2305.0757, 2321.0444, 2351.1042, 2753.2139, 3337.6887

30. A5D7H6 **Mass:** 88869 **Score:** 32 **Expect:** 95 **Matches:** 14

KLRAQ1 protein OS=Bos taurus OX=9913 GN=PPP1R21 PE=2 SV=1

Observed	Mr(expt)	Mr(calc)	ppm	Start	End	Miss	Ions	Peptide
842.4648	841.4575	841.5134	-66.34	66	72	1	---	R.NLQLAKR.V
853.4749	852.4676	852.4453	26.1	135	141	0	---	K.HVEAELR.S
1033.6478	1032.6405	1032.5451	92.4	183	191	1	---	K.SQTLEKEAK.E
1131.6466	1130.6393	1130.5931	40.9	759	767	1	---	K.QREEIDTLK.M
1147.6801	1146.6728	1146.6357	32.4	549	558	1	---	R.RVLLSSTESR.E
1165.6774	1164.6701	1164.5696	86.3	709	718	1	---	K.SKEALTEEMK.V
1165.6774	1164.6701	1164.5696	86.3	709	718	1	---	K.SKEALTEEMK.V
1301.6583	1300.6510	1300.6623	-8.65	30	42	0	---	K.GVVDEQANSAALK.E
1301.6583	1300.6511	1300.6623	-8.63	30	42	0	---	K.GVVDEQANSAALK.E
1417.6385	1416.6312	1416.7395	-76.41	195	205	1	---	R.LRTEECQLQLK.N
1567.6675	1566.6602	1566.7613	-64.50	689	701	1	---	K.SVHFYAECRALSK.R
1617.9797	1616.9724	1616.8734	61.3	604	619	1	---	K.LKSTSSGQVVGVAQEK.A
2004.8711	2003.8638	2003.9332	-34.64	504	521	0	---	K.TGSGFISPLSAECMLQYK.K + Oxidation (M)
2015.1777	2014.1704	2014.0346	67.4	339	354	1	---	K.LKTFSEHLTSYICFLR.K

No match to: 848.4858, 850.0170, 850.4961, 861.0225, 864.5088, 865.9901, 868.5096, 870.5018, 876.9943, 881.9693, 882.5198, 882.5198, 892.9673, 892.9674, 908.9358, 927.4315, 951.5755, 1017.6181, 1019.6312, 1045.5127, 1051.6508, 1051.6508, 1151.6693, 1169.5049, 1179.5454, 1201.5295, 1334.8121, 1334.8121, 1363.6659, 1433.6392, 1439.6532, 1448.8452, 1475.6703, 1479.7141, 1479.7141, 1593.7277, 1597.6893, 1597.6893, 1673.7250, 1690.7394, 1732.0092, 1826.8557, 1838.8295, 1848.8546, 1873.8428, 1901.1566, 1939.9556, 1993.8798, 2093.9248, 2111.9341, 2111.9341, 2150.9111, 2150.9111, 2210.9976, 2233.0032, 2283.0698, 2305.0757, 2321.0444, 2351.1042, 2753.2139, 3337.6887

PDF-XChange
Click to buy NOW!
www.docu-track.com

PDF-XChange
Click to buy NOW!
www.docu-track.com

Search Parameters

```
Type of search         : Sequence Query
Enzyme                 : Trypsin
Fixed modifications    : Carbamidomethyl (C)
Variable modifications : Oxidation (M)
Mass values            : Monoisotopic
Protein Mass           : Unrestricted
Peptide Mass Tolerance : ± 100 ppm
Fragment Mass Tolerance: ± 0.3 Da
Max Missed Cleavages   : 1
Instrument type        : MALDI-TOF-TOF
   Query1 (842.4648,1+): <no title>
   Query2 (848.4858,1+): <no title>
   Query3 (850.0170,1+): <no title>
   Query4 (850.4961,1+): <no title>
   Query5 (853.4749,1+): <no title>
   Query6 (861.0225,1+): <no title>
   Query7 (864.5088,1+): <no title>
   Query8 (865.9901,1+): <no title>
   Query9 (868.5096,1+): <no title>
  Query10 (870.5018,1+): <no title>
  Query11 (876.9943,1+): <no title>
  Query12 (881.9693,1+): <no title>
  Query13 (882.5198,1+): Locus:1..14.0.3
  Query14 (882.5198,1+): <no title>
  Query15 (892.9673,1+): <no title>
  Query16 (892.9674,1+): Locus:1..14.0.10
  Query17 (908.9358,1+): <no title>
  Query18 (927.4315,1+): <no title>
  Query19 (951.5755,1+): <no title>
 Query20 (1017.6181,1+): <no title>
 Query21 (1017.6181,1+): Locus:1..14.0.11
 Query22 (1019.6312,1+): <no title>
 Query23 (1033.6478,1+): <no title>
 Query24 (1045.5127,1+): <no title>
 Query25 (1051.6508,1+): Locus:1..14.0.5
 Query26 (1051.6508,1+): <no title>
 Query27 (1131.6466,1+): <no title>
 Query28 (1147.6801,1+): <no title>
 Query29 (1151.6693,1+): <no title>
 Query30 (1165.6774,1+): Locus:1..14.0.6
 Query31 (1165.6774,1+): <no title>
 Query32 (1169.5049,1+): <no title>
 Query33 (1179.5454,1+): <no title>
 Query34 (1201.5295,1+): <no title>
 Query35 (1301.6583,1+): <no title>
 Query36 (1301.6583,1+): Locus:1..14.0.4
 Query37 (1334.8121,1+): <no title>
 Query38 (1334.8121,1+): Locus:1..14.0.7
 Query39 (1363.6659,1+): <no title>
 Query40 (1417.6385,1+): <no title>
 Query41 (1433.6392,1+): <no title>
 Query42 (1439.6532,1+): <no title>
 Query43 (1448.8452,1+): <no title>
 Query44 (1475.6703,1+): <no title>
 Query45 (1479.7141,1+): <no title>
 Query46 (1479.7141,1+): Locus:1..14.0.9
 Query47 (1567.6675,1+): <no title>
 Query48 (1593.7277,1+): <no title>
 Query49 (1597.6893,1+): <no title>
 Query50 (1597.6893,1+): Locus:1..14.0.1
 Query51 (1617.9797,1+): <no title>
 Query52 (1673.7250,1+): <no title>
 Query53 (1690.7394,1+): <no title>
 Query54 (1732.0092,1+): <no title>
 Query55 (1826.8557,1+): <no title>
 Query56 (1838.8295,1+): <no title>
 Query57 (1848.8546,1+): <no title>
 Query58 (1873.8428,1+): <no title>
 Query59 (1901.1566,1+): <no title>
 Query60 (1939.9556,1+): <no title>
 Query61 (1993.8798,1+): <no title>
 Query62 (2004.8711,1+): <no title>
 Query63 (2015.1777,1+): <no title>
 Query64 (2093.9248,1+): <no title>
 Query65 (2111.9341,1+): Locus:1..14.0.2
 Query66 (2111.9341,1+): <no title>
 Query67 (2150.9111,1+): <no title>
 Query68 (2150.9111,1+): Locus:1..14.0.12
 Query69 (2210.9976,1+): <no title>
 Query70 (2233.0032,1+): <no title>
 Query71 (2283.0698,1+): <no title>
 Query72 (2305.0757,1+): <no title>
 Query73 (2321.0444,1+): <no title>
 Query74 (2351.1042,1+): <no title>
 Query75 (2753.2139,1+): <no title>
 Query76 (3337.6887,1+): <no title>
```

Mascot: http://www.matrixscience.com/
