# Supplementary material for: Alpha-2-Macroglobulin as a New Promising Biomarker Improving the Diagnostic Sensitivity of Bovine Paratuberculosis
Source: Front Vet Sci. 2021 Mar 5;8:637716. doi: 10.3389/fvets.2021.637716 (PMC7973028; doi:10.3389/fvets.2021.637716)
Supplement: Supplementary Data 1 — Mascot search results and raw data files for LC-MS/MS analysis for the identification of specific blood proteins in cattle with Johne's disease. [file Data_Sheet_1.zip › mascot/10 Uncharacterized protein_2.pdf]

PDF-XChange Click to buy NOW! www.docu-track.com

MATRIX SCIENCE MASCOT Search Results

Protein View: A0A3Q1M3L6

Uncharacterized protein OS=Bos taurus OX=9913 PE=1 SV=1

Database:	Uniprot_bovine
Score:	82
Expect:	0.00079
Nominal mass (M_r):	41077
Calculated pI:	5.16

Sequence similarity is available as an NCBI BLAST search of A0A3Q1M3L6 against nr.

Search parameters

Enzyme:	Trypsin: cuts C-term side of KR unless next residue is P.
Fixed modifications:	Carbamidomethyl (C)
Variable modifications:	Oxidation (M)

Protein sequence coverage: 26%

Matched peptides shown in *bold red*.

```
  1 MPEPVTVTWN SGALKSGVHT FPAVLQSSGL YSLSSMVTVP ASSSGQTFTC
 51 NVAHPASSTK VDKAVDPTCK PSPCDCCPPP ELPGGPSVFI FPPKPKDTLT
101 ISGTPEVTCV VVDVGHDDPE VKFSWFVDDV EVNTATTKPR EEQFNSTYRV
151 VSALRIQHQD WTGGKEFKCK VHNEGLPAPI VRTISRTKGP AREPQVYVLA
201 PPQEELSKST VSLTCMVTSF YPDYIAVEWQ RNGQPESEDK YGTTPPQLDA
251 DSSYFLYSKL RVDRNSWQEG DTYTCVVMHE ALHNHYTQKS TSKSADLLLE
301 EEICADAQDG ELDGLWTTIS IFITLFLLSV CYSATVTLFK VKWIFSSVVE
351 LKRTIVPDYR NMIGQGA
```

Unformatted sequence string: 367 residues (for pasting into other applications).

Sort peptides by Residue Number Increasing Mass Decreasing Mass

Show predicted peptides also

Query	Start – End	Observed	Mr(expt)	Mr(calc)	ppm	M	Score	Peptide
65	123 – 140	2111.9341	2110.9268	2111.0324	-50.0	0	46	K.FSWFVDDVEVNTATTKPR.E
66	123 – 140	2111.9341	2110.9268	2111.0324	-50.0	0		K.FSWFVDDVEVNTATTKPR.E
32	156 – 165	1169.5049	1168.4976	1168.5625	-55.5	0		R.IQHQDWTGGK.E
35	171 – 182	1301.6583	1300.6510	1300.7252	-57.0	0		K.VHNEGLPAPIVR.T
36	171 – 182	1301.6583	1300.6511	1300.7252	-57.0	0		K.VHNEGLPAPIVR.T
55	193 – 208	1826.8557	1825.8484	1825.9462	-53.5	0		R.EPQVYVLAPPQEELSK.S
75	209 – 231	2753.2139	2752.2066	2752.2877	-29.5	0		K.STVSLTCMVTSFYPDYIAVEWQR.N
39	343 – 353	1363.6659	1362.6586	1362.7660	-78.8	1		K.WIFSSVVELKR.T
22	353 – 360	1019.6312	1018.6239	1018.5560	66.7	1		K.RTIVPDYR.N

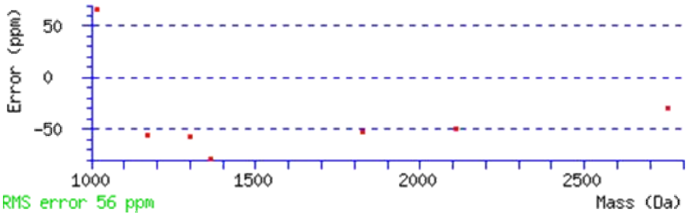


```
ID   A0A3Q1M3L6_BOVIN        Unreviewed;        367 AA.
AC   A0A3Q1M3L6;
DT   10-APR-2019, integrated into UniProtKB/TrEMBL.
DT   10-APR-2019, sequence version 1.
DT   11-DEC-2019, entry version 5.
DE   SubName: Full=Uncharacterized protein {ECO:0000313|Ensembl:ENSBTAP00000064749};
OS   Bos taurus (Bovine).
OC   Eukaryota; Metazoa; Chordata; Craniata; Vertebrata; Euteleostomi; Mammalia;
```

PDF-XChange Click to buy NOW! www.docu-track.com

```
[illegible]C   Eutheria; Laurasiatheria; Artiodactyla; Ruminantia; Pecora; Bovidae;
OC   Bovinae; Bos.
OX   NCBI_TaxID=9913 {ECO:0000313|Ensembl:ENSBTAP00000064749, ECO:0000313|Proteomes:UP000009136};
RN   [1] {ECO:0000313|Ensembl:ENSBTAP00000064749, ECO:0000313|Proteomes:UP000009136}
RP   NUCLEOTIDE SEQUENCE [LARGE SCALE GENOMIC DNA].
RC   STRAIN=Hereford {ECO:0000313|Ensembl:ENSBTAP00000064749,
RC   ECO:0000313|Proteomes:UP000009136};
RA   Rosen B.D., Bickhart D.M., Koren S., Schnabel R.D., Hall R., Zimin A.,
RA   Dreischer C., Schultheiss S., Schroeder S.G., Elsik C.G., Couldrey C.,
RA   Liu G.E., Van Tassell C.P., Phillippy A.M., Smith T.P.L., Medrano J.F.;
RT   "ARS-UCD1.2.";
RL   Submitted (MAR-2018) to the EMBL/GenBank/DDBJ databases.
RN   [2] {ECO:0000313|Ensembl:ENSBTAP00000064749}
RP   IDENTIFICATION.
RC   STRAIN=Hereford {ECO:0000313|Ensembl:ENSBTAP00000064749};
RG   Ensembl;
RL   Submitted (JAN-2019) to UniProtKB.
CC   -----------------------------------------------------------------------
CC   Copyrighted by the UniProt Consortium, see https://www.uniprot.org/terms
CC   Distributed under the Creative Commons Attribution (CC BY 4.0) License
CC   -----------------------------------------------------------------------
DR   Ensembl; ENSBTAT00000083559; ENSBTAP00000064749; ENSBTAG00000055240.
DR   GeneTree; ENSGT00940000162793; -.
DR   Proteomes; UP000009136; Chromosome 20.
DR   GO; GO:0016021; C:integral component of membrane; IEA:UniProtKB-KW.
DR   Gene3D; 2.60.40.10; -; 3.
DR   InterPro; IPR007110; Ig-like_dom.
DR   InterPro; IPR036179; Ig-like_dom_sf.
DR   InterPro; IPR013783; Ig-like_fold.
DR   InterPro; IPR003006; Ig/MHC_CS.
DR   InterPro; IPR003597; Ig_C1-set.
DR   Pfam; PF07654; C1-set; 3.
DR   SMART; SM00407; IGc1; 3.
DR   SUPFAM; SSF48726; SSF48726; 3.
DR   PROSITE; PS50835; IG_LIKE; 3.
DR   PROSITE; PS00290; IG_MHC; 2.
PE   1: Evidence at protein level;
KW   Immunoglobulin domain {ECO:0000256|SAAS:SAAS00653601};
KW   Membrane {ECO:0000256|SAM:Phobius};
KW   Proteomics identification {ECO:0000213|PeptideAtlas:A0A3Q1M3L6};
KW   Reference proteome {ECO:0000313|Proteomes:UP000009136};
KW   Transmembrane {ECO:0000256|SAM:Phobius};
KW   Transmembrane helix {ECO:0000256|SAM:Phobius}.
FT   TRANSMEM        314..339
FT                   /note="Helical"
FT                   /evidence="ECO:0000256|SAM:Phobius"
FT   DOMAIN          1..66
FT                   /note="Ig-like"
FT                   /evidence="ECO:0000259|PROSITE:PS50835"
FT   DOMAIN          86..185
FT                   /note="Ig-like"
FT                   /evidence="ECO:0000259|PROSITE:PS50835"
FT   DOMAIN          194..292
FT                   /note="Ig-like"
FT                   /evidence="ECO:0000259|PROSITE:PS50835"
SQ   SEQUENCE   367 AA;  40476 MW;  AF804286143DA888 CRC64;
     MPEPVTVTWN SGALKSGVHT FPAVLQSSGL YSLSSMVTVP ASSSGQTFTC NVAHPASSTK
     VDKAVDPTCK PSPCDCCPPP ELPGGPSVFI FPPKPKDTLT ISGTPEVTCV VVDVGHDDPE
     VKFSWFVDDV EVNTATTKPR EEQFNSTYRV VSALRIQHQD WTGGKEFKCK VHNEGLPAPI
     VRTISRTKGP AREPQVYVLA PPQEELSKST VSLTCMVTSF YPDYIAVEWQ RNGQPESEDK
     YGTTPPQLDA DSSYFLYSKL RVDRNSWQEG DTYTCVVMHE ALHNHYTQKS TSKSADLLLE
     EEICADAQDG ELDGLWTTIS IFITLFLLSV CYSATVTLFK VKWIFSSVVE LKRTIVPDYR
     NMIGQGA
//
```

Mascot: http://www.matrixscience.com/
