# Supplementary material for: Alpha-2-Macroglobulin as a New Promising Biomarker Improving the Diagnostic Sensitivity of Bovine Paratuberculosis
Source: Front Vet Sci. 2021 Mar 5;8:637716. doi: 10.3389/fvets.2021.637716 (PMC7973028; doi:10.3389/fvets.2021.637716)
Supplement: Supplementary Data 1 — Mascot search results and raw data files for LC-MS/MS analysis for the identification of specific blood proteins in cattle with Johne's disease. [file Data_Sheet_1.zip › mascot/11 hIgG1 heavy chain constant region_1.pdf]

PDF-XChange Click to buy NOW! www.docu-track.com

Mascot Search Results

User	:
Email	:
Search title	: BAEDW\2019_10_16\2019_10_16\MSMS 5\C13
Database	: NCBInr 20131104 (33815671 sequences; 11795550776 residues)
Timestamp	: 15 Oct 2019 at 09:37:24 GMT
Warning	: A Peptide summary report will usually give a much clearer picture of MS/MS search results.
Top Score	: 151 for gi|7547266, IgG1 heavy chain constant region [Bos taurus]

Mascot Score Histogram

Protein score is -10*Log(P), where P is the probability that the observed match is a random event.
Protein scores greater than 88 are significant (p<0.05).
Protein scores are derived from ions scores as a non-probabilistic basis for ranking protein hits.

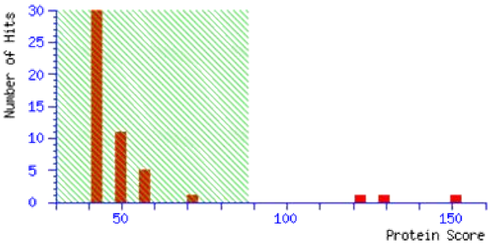

Protein Summary Report

Format As | Protein Summary (deprecated) | Help

Significance threshold p< 0.05 Max. number of hits 50

Re-Search All | Search Unmatched

Index

	Accession	Mass	Score	Description
1.	gi|7547266	36510	151	IgG1 heavy chain constant region [Bos taurus]
2.	gi|440891567	11475	129	Ig gamma-1 chain C region, partial [Bos grunniens mutus]
3.	gi|108750	51391	121	Ig heavy chain precursor (B/MT.4A.17.H5.A5) - bovine
4.	gi|91982959	36562	71	immunoglobulin gamma 1 heavy chain constant region [Bos taurus]
5.	gi|498046415	7331	56	hypothetical protein [Neisseria gonorrhoeae]
6.	gi|337290484	15574	56	hypothetical protein CULC22_00873 [Corynebacterium ulcerans BR-AD22]
7.	gi|546357233	26141	55	putative uncharacterized protein [Blautia hydrogenotrophica CAG:147]
8.	gi|489983378	9455	54	hypothetical protein [Mycobacterium phlei]
9.	gi|490762382	56174	54	Endoglucanase [Clostridium termitidis]
10.	gi|518548361	35653	53	LysR family transcriptional regulator [Ralstonia solanacearum]
11.	gi|327305893	11598	52	hypothetical protein TERG_02355 [Trichophyton rubrum CBS 118892]
12.	gi|171688784	30526	50	hypothetical protein [Podospora anserina S mat+]
13.	gi|495323294	9800	50	hypothetical protein [Reinekea blandensis]
14.	gi|255291985	7225	50	hypothetical protein [uncultured bacterium]
15.	gi|492883710	4310	50	hypothetical protein [Afipia broomeae]
16.	gi|551336008	5799	49	hypothetical protein [Perlucidibaca piscinae]
17.	gi|495360281	22699	48	hypothetical protein [Pseudomonas sp. GM80]
18.	gi|487598108	4479	48	propanediol utilization ferredoxin, partial [Salmonella enterica]
19.	gi|401763462	16967	48	hypothetical protein ECENHK_09860 [Enterobacter cloacae subsp. cloacae ENHKU01]
20.	gi|553884411	20219	47	integrase [Gloeobacter sp. JS]
21.	gi|497783917	30566	46	Beta-lactamase, partial [Burkholderia ubonensis]
22.	gi|150377816	75741	46	mechanosensitive ion channel MscS [Sinorhizobium medicae WSM419]
23.	gi|332024455	50459	46	Dual specificity protein phosphatase 22 [Acromyrmex echinatior]
24.	gi|489455877	40956	45	thiouridylase [Clostridium botulinum]
25.	gi|486622113	3493	45	hypothetical protein [Escherichia coli]
26.	gi|546373423	5165	45	hypothetical protein [Prevotella sp. CAG:1058]
27.	gi|495396594	25331	45	peptide ABC transporter ATP-binding protein [[Bacteroides] pectinophilus]
28.	gi|517950378	44454	44	hypothetical protein [Brevibacillus sp. phR]
29.	gi|493698297	5437	44	hypothetical protein [Haloterrigena thermotolerans]
30.	gi|163839610	17902	44	nucleotide-binding protein [Renibacterium salmoninarum ATCC 33209]
31.	gi|548180461	10887	44	uncharacterized protein [Bacteroides uniformis CAG:3]
32.	gi|339499150	28813	44	short-chain dehydrogenase/reductase SDR [Treponema caldaria DSM 7334]
33.	gi|522141702	27885	44	hypothetical protein [Massilia niastensis]
34.	gi|497966698	29777	44	peptide ABC transporter ATP-binding protein [Streptomyces sp. SA3_actF]
35.	gi|547298347	24974	43	lipoprotein ABC transporter ATP-binding protein LolD [Clostridium hathewayi CAG:224]
36.	gi|496545606	25186	43	peptide ABC transporter ATP-binding protein [Lachnospiraceae bacterium 3_1_57FAA_CT1]
37.	gi|479202615	25300	43	ABC-type antimicrobial peptide transport system, ATPase component [Roseburia intestinalis M50/1]
38.	gi|296132158	47550	43	respiratory-chain NADH dehydrogenase domain 51 kDa subunit [Thermincola potens JR]
39.	gi|494243326	26715	43	short-chain dehydrogenase [Methylophaga aminisulfidivorans]
40.	gi|491299814	25459	43	hypothetical protein [Amycolatopsis azurea]
41.	gi|312112921	46524	43	lytic murein transglycosylase [Rhodomicrobium vannielii ATCC 17100]
42.	gi|396482791	59448	43	predicted protein [Leptosphaeria maculans JN3]
43.	gi|550962788	10224	43	hypothetical protein [Pseudochrobactrum sp. AO18b]
44.	gi|297182578	12830	43	transposase and inactivated derivatives [uncultured Rhizobiales bacterium HF4000_32B18]
45.	gi|341890310	11784	43	hypothetical protein CAEBREN_08480 [Caenorhabditis brenneri]
46.	gi|123189376	27775	43	hypothetical protein [Trichomonas vaginalis G3]
47.	gi|493826333	24992	42	peptide ABC transporter ATP-binding protein [Clostridium hathewayi]
48.	gi|332235189	5332	42	PREDICTED: protein phosphatase 1 regulatory subunit 14D [Nomascus leucogenys]
49.	gi|520945468	15697	42	LysR family transcriptional regulator, partial [Pseudomonas syringae]
50.	gi|486208296	4883	42	hypothetical protein [Escherichia coli]

Results List

PDF-XChange Click to buy NOW! www.docu-track.com

gi|7547266 **Mass:** 36510 **Score:** 151 **Expect:** 2.7e-008 **Matches:** 10
IgG1 heavy chain constant region [Bos taurus]

Observed	Mr(expt)	Mr(calc)	ppm	Start	End	Miss	Ions	Peptide
1169.6356	1168.6283	1168.5625	56.3	188 -	197	0	---	R.IQHQDWTGGK.E
1169.6356	1168.6283	1168.5625	56.3	188 -	197	0	---	R.IQHQDWTGGK.E
1301.8031	1300.7958	1300.7252	54.3	203 -	214	0	---	K.VHNEGLPAPIVR.T
1301.8031	1300.7958	1300.7252	54.3	203 -	214	0	51	K.VHNEGLPAPIVR.T
2112.1482	2111.1409	2111.0324	51.4	155 -	172	0	46	K.FSWFVDDVEVNTATTKPR.E
2112.1482	2111.1409	2111.0324	51.4	155 -	172	0	---	K.FSWFVDDVEVNTATTKPR.E
2151.1111	2150.1038	2149.9957	50.3	273 -	291	0	13	K.YGTTPPQLDADGSYFLYSR.L
2151.1111	2150.1038	2149.9957	50.3	273 -	291	0	---	K.YGTTPPQLDADGSYFLYSR.L
2753.4365	2752.4292	2752.2877	51.4	241 -	263	0	---	K.STVSLTCMVTSFYPDYIAVEWQR.N
2753.4365	2752.4292	2752.2877	51.4	241 -	263	0	---	K.STVSLTCMVTSFYPDYIAVEWQR.N

No match to: 841.1960, 841.1960, 842.5637, 855.0995, 871.0715, 882.6186, 882.6186, 893.0596, 909.0323, 915.1290, 915.1290, 927.5462, 947.0018, 1051.7703, 1060.1125, 1060.1126, 1083.1046, 1313.8103, 1313.8103, 1325.8025, 1339.7694, 1674.8976, 1874.0144, 2005.0609, 2124.1565, 2124.1565, 2212.2224

2. gi|440891567 **Mass:** 11475 **Score:** 129 **Expect:** 4.3e-006 **Matches:** 6
Ig gamma-1 chain C region, partial [Bos grunniens mutus]

Observed	Mr(expt)	Mr(calc)	ppm	Start	End	Miss	Ions	Peptide
1169.6356	1168.6283	1168.5625	56.3	71 -	80	0	---	R.IQHQDWTGGK.E
1169.6356	1168.6283	1168.5625	56.3	71 -	80	0	---	R.IQHQDWTGGK.E
1301.8031	1300.7958	1300.7252	54.3	86 -	97	0	---	K.VHNEGLPAPIVR.T
1301.8031	1300.7958	1300.7252	54.3	86 -	97	0	51	K.VHNEGLPAPIVR.T
2112.1482	2111.1409	2111.0324	51.4	38 -	55	0	46	K.FSWFVDDVEVNTATTKPR.E
2112.1482	2111.1409	2111.0324	51.4	38 -	55	0	---	K.FSWFVDDVEVNTATTKPR.E

No match to: 841.1960, 841.1960, 842.5637, 855.0995, 871.0715, 882.6186, 882.6186, 893.0596, 909.0323, 915.1290, 915.1290, 927.5462, 947.0018, 1051.7703, 1060.1125, 1060.1126, 1083.1046, 1313.8103, 1313.8103, 1325.8025, 1339.7694, 1674.8976, 1874.0144, 2005.0609, 2124.1565, 2124.1565, 2151.1111, 2151.1111, 2212.2224, 2753.4365, 2753.4365

3. gi|108750 **Mass:** 51391 **Score:** 121 **Expect:** 2.7e-005 **Matches:** 8
Ig heavy chain precursor (B/MT.4A.17.H5.A5) - bovine

Observed	Mr(expt)	Mr(calc)	ppm	Start	End	Miss	Ions	Peptide
1169.6356	1168.6283	1168.5625	56.3	329 -	338	0	---	R.IQHQDWTGGK.E
1169.6356	1168.6283	1168.5625	56.3	329 -	338	0	---	R.IQHQDWTGGK.E
1301.8031	1300.7958	1300.7252	54.3	344 -	355	0	---	K.VHNEGLPAPIVR.T
1301.8031	1300.7958	1300.7252	54.3	344 -	355	0	51	K.VHNEGLPAPIVR.T
2112.1482	2111.1409	2111.0324	51.4	296 -	313	0	46	K.FSWFVDDVEVNTATTKPR.E
2112.1482	2111.1409	2111.0324	51.4	296 -	313	0	---	K.FSWFVDDVEVNTATTKPR.E
2753.4365	2752.4292	2752.2877	51.4	382 -	404	0	---	K.STVSLTCMVTSFYPDYIAVEWQR.N
2753.4365	2752.4292	2752.2877	51.4	382 -	404	0	---	K.STVSLTCMVTSFYPDYIAVEWQR.N

No match to: 841.1960, 841.1960, 842.5637, 855.0995, 871.0715, 882.6186, 882.6186, 893.0596, 909.0323, 915.1290, 915.1290, 927.5462, 947.0018, 1051.7703, 1060.1125, 1060.1126, 1083.1046, 1313.8103, 1313.8103, 1325.8025, 1339.7694, 1674.8976, 1874.0144, 2005.0609, 2124.1565, 2124.1565, 2151.1111, 2151.1111, 2212.2224

4. gi|91982959 **Mass:** 36562 **Score:** 71 **Expect:** 2.9 **Matches:** 6
immunoglobulin gamma 1 heavy chain constant region [Bos taurus]

Observed	Mr(expt)	Mr(calc)	ppm	Start	End	Miss	Ions	Peptide
1169.6356	1168.6283	1168.5625	56.3	188 -	197	0	---	R.IQHQDWTGGK.E
1169.6356	1168.6283	1168.5625	56.3	188 -	197	0	---	R.IQHQDWTGGK.E
1301.8031	1300.7958	1300.7252	54.3	203 -	214	0	---	K.VHNEGLPAPIVR.T
1301.8031	1300.7958	1300.7252	54.3	203 -	214	0	51	K.VHNEGLPAPIVR.T
2753.4365	2752.4292	2752.2877	51.4	241 -	263	0	---	K.STVSLTCMVTSFYPDYIAVEWQR.N
2753.4365	2752.4292	2752.2877	51.4	241 -	263	0	---	K.STVSLTCMVTSFYPDYIAVEWQR.N

No match to: 841.1960, 841.1960, 842.5637, 855.0995, 871.0715, 882.6186, 882.6186, 893.0596, 909.0323, 915.1290, 915.1290, 927.5462, 947.0018, 1051.7703, 1060.1125, 1060.1126, 1083.1046, 1313.8103, 1313.8103, 1325.8025, 1339.7694, 1674.8976, 1874.0144, 2005.0609, 2112.1482, 2112.1482, 2124.1565, 2124.1565, 2151.1111, 2151.1111, 2212.2224

5. gi|498046415 **Mass:** 7331 **Score:** 56 **Expect:** 83 **Matches:** 5
hypothetical protein [Neisseria gonorrhoeae]

Observed	Mr(expt)	Mr(calc)	ppm	Start	End	Miss	Ions	Peptide
1874.0144	1873.0071	1872.9516	29.6	2 -	17	1	---	M.PSERLSDGIFPCILNR.V
2005.0609	2004.0536	2003.9921	30.7	1 -	17	1	---	-.MPSERLSDGIFPCILNR.V
2212.2224	2211.2151	2211.0114	92.2	47 -	69	0	---	K.GLMPSEGSDGIGIGESEAVAHAQ.-
2753.4365	2752.4292	2752.1680	94.9	18 -	42	1	---	R.VYETPDAFGGNVGCCAMFGGDQRTK.C + Oxidation (M)
2753.4365	2752.4292	2752.1680	94.9	18 -	42	1	---	R.VYETPDAFGGNVGCCAMFGGDQRTK.C + Oxidation (M)

No match to: 841.1960, 841.1960, 842.5637, 855.0995, 871.0715, 882.6186, 882.6186, 893.0596, 909.0323, 915.1290, 915.1290, 927.5462, 947.0018, 1051.7703, 1060.1125, 1060.1126, 1083.1046, 1169.6356, 1169.6356, 1301.8031, 1301.8031, 1313.8103, 1313.8103, 1325.8025, 1339.7694, 1674.8976, 2112.1482, 2112.1482, 2124.1565, 2124.1565, 2151.1111, 2151.1111

6. gi|337290484 **Mass:** 15574 **Score:** 56 **Expect:** 91 **Matches:** 7
hypothetical protein CULC22_00873 [Corynebacterium ulcerans BR-AD22]

Observed	Mr(expt)	Mr(calc)	ppm	Start	End	Miss	Ions	Peptide
1169.6356	1168.6283	1168.6339	-4.81	73 -	83	0	---	R.LGLPSLDTPEK.G
1169.6356	1168.6283	1168.6339	-4.79	73 -	83	0	---	R.LGLPSLDTPEK.G
1313.8103	1312.8030	1312.6888	87.0	118 -	129	1	---	K.VEPASHATFARK.G
1313.8103	1312.8030	1312.6888	87.0	117 -	128	1	---	R.KVEPASHATFAR.K
1325.8025	1324.7952	1324.7350	45.4	72 -	83	1	---	K.RLGLPSLDTPEK.G
2753.4365	2752.4292	2752.4535	-8.81	73 -	98	1	---	R.LGLPSLDTPEKGPELGDNYLVLLDGK.G
2753.4365	2752.4292	2752.4535	-8.80	73 -	98	1	---	R.LGLPSLDTPEKGPELGDNYLVLLDGK.G

No match to: 841.1960, 841.1960, 842.5637, 855.0995, 871.0715, 882.6186, 882.6186, 893.0596, 909.0323, 915.1290, 915.1290, 927.5462, 947.0018, 1051.7703, 1060.1125, 1060.1126, 1083.1046, 1301.8031, 1301.8031, 1339.7694, 1674.8976, 1874.0144, 2005.0609, 2112.1482, 2112.1482, 2124.1565, 2124.1565, 2151.1111, 2151.1111, 2212.2224

7. gi|546357233 **Mass:** 26141 **Score:** 55 **Expect:** 1.1e+002 **Matches:** 9
putative uncharacterized protein [Blautia hydrogenotrophica CAG:147]

Observed	Mr(expt)	Mr(calc)	ppm	Start	End	Miss	Ions	Peptide
1169.6356	1168.6283	1168.6889	-51.86	105 -	114	0	---	K.NILMPLILDK.R
1169.6356	1168.6283	1168.6889	-51.84	105 -	114	0	---	K.NILMPLILDK.R
1313.8103	1312.8030	1312.7788	18.4	104 -	114	1	---	R.KNILMPLILDK.R + Oxidation (M)

PDF-XChange Click to buy NOW! www.docu-track.com

1313.8103	1312.8030	1312.7788	18.4	104 - 114	1	---	R.KNILMPLILDK.R + Oxidation (M)
1325.8025	1324.7952	1324.7900	3.91	105 - 115	1	---	K.NILMPLILDKR.K
2124.1565	2123.1492	2123.2143	-30.65	86 - 103	1	---	R.KVGLVYQFYNLIPTLTVR.K
2124.1565	2123.1492	2123.2143	-30.65	87 - 104	1	---	K.VGLVYQFYNLIPTLTVRK.N
2151.1111	2150.1038	2150.1372	-15.51	211 - 229	1	---	R.IITIEDGKIIHDVFHDSAK.W
2151.1111	2150.1038	2150.1372	-15.50	211 - 229	1	---	R.IITIEDGKIIHDVFHDSAK.W

No match to: 841.1960, 841.1960, 842.5637, 855.0995, 871.0715, 882.6186, 882.6186, 893.0596, 909.0323, 915.1290, 915.1290, 927.5462, 947.0018, 1051.7703, 1060.1125, 1060.1126, 1083.1046, 1301.8031, 1301.8031, 1339.7694, 1674.8976, 1874.0144, 2005.0609, 2112.1482, 2112.1482, 2212.2224, 2753.4365, 2753.4365

8. gi|489983378 **Mass:** 9455 **Score:** 54 **Expect:** 1.4e+002 **Matches:** 7
hypothetical protein [Mycobacterium phlei]

Observed	Mr(expt)	Mr(calc)	ppm	Start - End	Miss	Ions	Peptide
1874.0144	1873.0071	1872.9806	14.2	10 - 28	0	---	R.ALAAAHTAHADELTAIAAR.L
2112.1482	2111.1409	2110.9702	80.9	67 - 87	1	---	R.MAQTAVSAARAATAYDDADGR.V
2112.1482	2111.1409	2110.9702	80.9	67 - 87	1	---	R.MAQTAVSAARAATAYDDADGR.V
2124.1565	2123.1492	2123.1375	5.51	29 - 50	0	---	R.LSNTPTPAGLGPVGADFVAALR.A
2124.1565	2123.1492	2123.1375	5.52	29 - 50	0	---	R.LSNTPTPAGLGPVGADFVAALR.A
2753.4365	2752.4292	2752.4004	10.5	2 - 28	1	---	M.HADTDAIRALAAAHTAHADELTAIAAR.L
2753.4365	2752.4292	2752.4004	10.5	2 - 28	1	---	M.HADTDAIRALAAAHTAHADELTAIAAR.L

No match to: 841.1960, 841.1960, 842.5637, 855.0995, 871.0715, 882.6186, 882.6186, 893.0596, 909.0323, 915.1290, 915.1290, 927.5462, 947.0018, 1051.7703, 1060.1125, 1060.1126, 1083.1046, 1169.6356, 1169.6356, 1301.8031, 1301.8031, 1313.8103, 1313.8103, 1325.8025, 1339.7694, 1674.8976, 2005.0609, 2151.1111, 2151.1111, 2212.2224

9. gi|490762382 **Mass:** 56174 **Score:** 54 **Expect:** 1.4e+002 **Matches:** 11
Endoglucanase [Clostridium termitidis]

Observed	Mr(expt)	Mr(calc)	ppm	Start - End	Miss	Ions	Peptide
927.5462	926.5389	926.5185	22.0	223 - 230	1	---	K.AIRDIDPK.H
1301.8031	1300.7958	1300.6809	88.3	49 - 60	1	---	K.HMLDTLGSVKGK.M + Oxidation (M)
1301.8031	1300.7958	1300.6809	88.3	49 - 60	1	---	K.HMLDTLGSVKGK.M + Oxidation (M)
1313.8103	1312.8030	1312.7827	15.5	11 - 22	1	---	K.IINTSGKVVNLR.G
1313.8103	1312.8030	1312.7827	15.5	11 - 22	1	---	K.IINTSGKVVNLR.G
1874.0144	1873.0071	1872.9979	4.94	1 - 17	1	---	-.MDLLQIENGKIINTSGK.V
2151.1111	2150.1038	2150.0545	22.9	382 - 400	1	---	R.NLGAENFVAQYWDSPGRVK.S
2151.1111	2150.1038	2150.0545	22.9	382 - 400	1	---	R.NLGAENFVAQYWDSPGRVK.S
2212.2224	2211.2151	2211.0498	74.8	305 - 323	0	---	R.QHNVPIWVGEFGSQYHGEK.E
2753.4365	2752.4292	2752.1785	91.1	332 - 353	0	---	K.AMDDQLAVYNECDINWTTWTYK.D + Oxidation (M)
2753.4365	2752.4292	2752.1785	91.1	332 - 353	0	---	K.AMDDQLAVYNECDINWTTWTYK.D + Oxidation (M)

No match to: 841.1960, 841.1960, 842.5637, 855.0995, 871.0715, 882.6186, 882.6186, 893.0596, 909.0323, 915.1290, 915.1290, 947.0018, 1051.7703, 1060.1125, 1060.1126, 1083.1046, 1169.6356, 1169.6356, 1325.8025, 1339.7694, 1674.8976, 2005.0609, 2112.1482, 2112.1482, 2124.1565, 2124.1565

10. gi|518548361 **Mass:** 35653 **Score:** 53 **Expect:** 1.9e+002 **Matches:** 9
LysR family transcriptional regulator [Ralstonia solanacearum]

Observed	Mr(expt)	Mr(calc)	ppm	Start - End	Miss	Ions	Peptide
1339.7694	1338.7621	1338.7846	-16.77	266 - 277	1	---	R.ILPAPVAFPKMR.F
1874.0144	1873.0071	1872.8975	58.6	1 - 15	1	---	-.MQMHGKDHLDTYLLR.V + Oxidation (M)
2005.0609	2004.0536	2003.9986	27.5	298 - 316	0	---	R.MIGEVAAELPQHPQLETAA.-
2124.1565	2123.1492	2123.0444	49.4	194 - 211	0	---	K.HYIEMPHLAPAPYAVMQR.S
2124.1565	2123.1492	2123.0444	49.4	194 - 211	0	---	K.HYIEMPHLAPAPYAVMQR.S
2151.1111	2150.1038	2150.2074	-48.19	226 - 243	1	---	R.RIQVSLPYFGLVPYVLMR.T
2151.1111	2150.1038	2150.2074	-48.18	226 - 243	1	---	R.RIQVSLPYFGLVPYVLMR.T
2753.4365	2752.4292	2752.3636	23.8	61 - 85	1	---	K.SGMVPTERGHELLALAQQSLEAMER.I
2753.4365	2752.4292	2752.3636	23.9	61 - 85	1	---	K.SGMVPTERGHELLALAQQSLEAMER.I

No match to: 841.1960, 841.1960, 842.5637, 855.0995, 871.0715, 882.6186, 882.6186, 893.0596, 909.0323, 915.1290, 915.1290, 927.5462, 947.0018, 1051.7703, 1060.1125, 1060.1126, 1083.1046, 1169.6356, 1169.6356, 1301.8031, 1301.8031, 1313.8103, 1313.8103, 1325.8025, 1674.8976, 2112.1482, 2112.1482, 2212.2224

11. gi|327305893 **Mass:** 11598 **Score:** 52 **Expect:** 2.4e+002 **Matches:** 5
hypothetical protein TERG_02355 [Trichophyton rubrum CBS 118892]

Observed	Mr(expt)	Mr(calc)	ppm	Start - End	Miss	Ions	Peptide
1169.6356	1168.6283	1168.6604	-27.47	42 - 51	0	---	R.QLPAYVLHTK.E
1169.6356	1168.6283	1168.6604	-27.46	42 - 51	0	18	R.QLPAYVLHTK.E
2005.0609	2004.0536	2004.0462	3.68	13 - 29	1	---	K.VVDDQTWLMLVRSSSLR.I
2753.4365	2752.4292	2752.3048	45.2	1 - 24	1	---	-.MIGSQDEAASEKVVDDQTWLMLVR.S + 2 Oxidation (M)
2753.4365	2752.4292	2752.3048	45.2	1 - 24	1	---	-.MIGSQDEAASEKVVDDQTWLMLVR.S + 2 Oxidation (M)

No match to: 841.1960, 841.1960, 842.5637, 855.0995, 871.0715, 882.6186, 882.6186, 893.0596, 909.0323, 915.1290, 915.1290, 927.5462, 947.0018, 1051.7703, 1060.1125, 1060.1126, 1083.1046, 1301.8031, 1301.8031, 1313.8103, 1313.8103, 1325.8025, 1339.7694, 1674.8976, 1874.0144, 2112.1482, 2112.1482, 2124.1565, 2124.1565, 2151.1111, 2151.1111, 2212.2224

12. gi|171688784 **Mass:** 30526 **Score:** 50 **Expect:** 3.1e+002 **Matches:** 10
hypothetical protein [Podospora anserina S mat+]

Observed	Mr(expt)	Mr(calc)	ppm	Start - End	Miss	Ions	Peptide
1169.6356	1168.6283	1168.6564	-24.03	11 - 21	0	---	R.LQGVIANLEGR.V
1169.6356	1168.6283	1168.6564	-24.01	11 - 21	0	---	R.LQGVIANLEGR.V
1301.8031	1300.7958	1300.6987	74.7	264 - 276	1	---	K.AGSTILSKLTHSS.-
1301.8031	1300.7958	1300.6987	74.7	264 - 276	1	---	K.AGSTILSKLTHSS.-
1325.8025	1324.7952	1324.7575	28.5	10 - 21	1	---	K.RLQGVIANLEGR.V
2005.0609	2004.0536	2004.0635	-4.94	92 - 110	1	---	K.KIMDAGGLVSDEIVIGMIK.E + Oxidation (M)
2112.1482	2111.1409	2111.0687	34.2	214 - 230	1	---	R.LETYHKQTTPVVNYYQK.T
2112.1482	2111.1409	2111.0687	34.2	214 - 230	1	---	R.LETYHKQTTPVVNYYQK.T
2124.1565	2123.1492	2122.9711	83.9	65 - 82	1	---	K.FNCCHLATGDMLRSQVAK.K + Oxidation (M)
2124.1565	2123.1492	2122.9711	83.9	65 - 82	1	---	K.FNCCHLATGDMLRSQVAK.K + Oxidation (M)

No match to: 841.1960, 841.1960, 842.5637, 855.0995, 871.0715, 882.6186, 882.6186, 893.0596, 909.0323, 915.1290, 915.1290, 927.5462, 947.0018, 1051.7703, 1060.1125, 1060.1126, 1083.1046, 1313.8103, 1313.8103, 1339.7694, 1674.8976, 1874.0144, 2151.1111, 2151.1111, 2212.2224, 2753.4365, 2753.4365

13. gi|495323294 **Mass:** 9800 **Score:** 50 **Expect:** 3.1e+002 **Matches:** 5
hypothetical protein [Reinekea blandensis]

Observed	Mr(expt)	Mr(calc)	ppm	Start - End	Miss	Ions	Peptide

PDF-XChange Click to buy NOW! www.docu-track.com

PDF-XChange Click to buy NOW! www.docu-track.com

1301.8031	1300.7958	1300.7350	46.7	27 -	38	1	---	R.AKLSQKPTVDSK.R
1301.8031	1300.7958	1300.7350	46.7	27 -	38	1	---	R.AKLSQKPTVDSK.R
1339.7694	1338.7621	1338.6425	89.4	74 -	85	1	---	R.VGFDAVCGKCVK.R
2753.4365	2752.4292	2752.4258	1.23	41 -	64	1	---	K.VSQTDGIPLVNMENFALFVFLDRK.T
2753.4365	2752.4292	2752.4258	1.24	40 -	63	1	---	R.KVSQTDGIPLVNMENFALFVFLDR.K

No match to: 841.1960, 841.1960, 842.5637, 855.0995, 871.0715, 882.6186, 882.6186, 893.0596, 909.0323, 915.1290, 915.1290, 927.5462, 947.0018, 1051.7703, 1060.1125, 1060.1126, 1083.1046, 1169.6356, 1169.6356, 1313.8103, 1313.8103, 1325.8025, 1674.8976, 1874.0144, 2005.0609, 2112.1482, 2112.1482, 2124.1565, 2124.1565, 2151.1111, 2151.1111, 2212.2224

14. gi|255291985 **Mass:** 7225 **Score:** 50 **Expect:** 3.4e+002 **Matches:** 6
hypothetical protein [uncultured bacterium]

Observed	Mr(expt)	Mr(calc)	ppm	Start	End	Miss	Ions	Peptide
1169.6356	1168.6283	1168.6022	22.3	3 -	12	0	---	R.MIAIHAQTER.K
1169.6356	1168.6283	1168.6022	22.3	3 -	12	0	---	R.MIAIHAQTER.K
1313.8103	1312.8030	1312.6921	84.5	3 -	13	1	---	R.MIAIHAQTERK.R + Oxidation (M)
1313.8103	1312.8030	1312.6921	84.5	3 -	13	1	---	R.MIAIHAQTERK.R + Oxidation (M)
1325.8025	1324.7952	1324.7034	69.4	2 -	12	1	---	M.RMIAIHAQTER.K
2212.2224	2211.2151	2211.1365	35.6	34 -	54	1	---	R.LRGLIRPDPAVCATASAACGR.K

No match to: 841.1960, 841.1960, 842.5637, 855.0995, 871.0715, 882.6186, 882.6186, 893.0596, 909.0323, 915.1290, 915.1290, 927.5462, 947.0018, 1051.7703, 1060.1125, 1060.1126, 1083.1046, 1301.8031, 1301.8031, 1339.7694, 1674.8976, 1874.0144, 2005.0609, 2112.1482, 2112.1482, 2124.1565, 2124.1565, 2151.1111, 2151.1111, 2753.4365, 2753.4365

15. gi|492883710 **Mass:** 4310 **Score:** 50 **Expect:** 3.5e+002 **Matches:** 4
hypothetical protein [Afipia broomeae]

Observed	Mr(expt)	Mr(calc)	ppm	Start	End	Miss	Ions	Peptide
1325.8025	1324.7952	1324.7134	61.7	1 -	11	0	---	-.MTMLAEIFLLK.L + Oxidation (M)
1339.7694	1338.7621	1338.7983	-27.02	12 -	23	1	---	K.LEALLRVAAIDR.R
1874.0144	1873.0071	1873.1110	-55.47	2 -	17	1	---	M.TMLAEIFLLKLEALLR.V
2005.0609	2004.0536	2004.1515	-48.84	1 -	17	1	---	-.MTMLAEIFLLKLEALLR.V

No match to: 841.1960, 841.1960, 842.5637, 855.0995, 871.0715, 882.6186, 882.6186, 893.0596, 909.0323, 915.1290, 915.1290, 927.5462, 947.0018, 1051.7703, 1060.1125, 1060.1126, 1083.1046, 1169.6356, 1169.6356, 1301.8031, 1301.8031, 1313.8103, 1313.8103, 1674.8976, 2112.1482, 2112.1482, 2124.1565, 2124.1565, 2151.1111, 2151.1111, 2212.2224, 2753.4365, 2753.4365

16. gi|551336008 **Mass:** 5799 **Score:** 49 **Expect:** 4.5e+002 **Matches:** 4
hypothetical protein [Perlucidibaca piscinae]

Observed	Mr(expt)	Mr(calc)	ppm	Start	End	Miss	Ions	Peptide
1301.8031	1300.7958	1300.6809	88.4	9 -	21	0	---	K.GIPSPSLVASMSR.C
1301.8031	1300.7958	1300.6809	88.4	9 -	21	0	13	K.GIPSPSLVASMSR.C
1674.8976	1673.8903	1673.8341	33.6	9 -	24	1	---	K.GIPSPSLVASMSRCGR.V
2005.0609	2004.0536	2004.0714	-8.87	3 -	21	1	---	R.VVTYPKGIPSPSLVASMSR.C + Oxidation (M)

No match to: 841.1960, 841.1960, 842.5637, 855.0995, 871.0715, 882.6186, 882.6186, 893.0596, 909.0323, 915.1290, 915.1290, 927.5462, 947.0018, 1051.7703, 1060.1125, 1060.1126, 1083.1046, 1169.6356, 1169.6356, 1313.8103, 1313.8103, 1325.8025, 1339.7694, 1874.0144, 2112.1482, 2112.1482, 2124.1565, 2124.1565, 2151.1111, 2151.1111, 2212.2224, 2753.4365, 2753.4365

17. gi|495360281 **Mass:** 22699 **Score:** 48 **Expect:** 5.1e+002 **Matches:** 8
hypothetical protein [Pseudomonas sp. GM80]

Observed	Mr(expt)	Mr(calc)	ppm	Start	End	Miss	Ions	Peptide
1169.6356	1168.6283	1168.6274	0.77	1 -	10	0	---	-.MPDLITPQVR.K
1169.6356	1168.6283	1168.6274	0.79	1 -	10	0	---	-.MPDLITPQVR.K
1313.8103	1312.8030	1312.7173	65.3	1 -	11	1	---	-.MPDLITPQVRK.L + Oxidation (M)
1313.8103	1312.8030	1312.7173	65.3	1 -	11	1	---	-.MPDLITPQVRK.L + Oxidation (M)
1674.8976	1673.8903	1673.8559	20.6	40 -	53	1	---	R.MRAWELADVDSLLR.E
2005.0609	2004.0536	2003.9548	49.3	12 -	30	0	---	K.LAAIGYGEASTDNNPQEVR.G
2112.1482	2111.1409	2110.9411	94.6	70 -	88	0	---	R.YNQLMAASEAAINADPNMR.S + 2 Oxidation (M)
2112.1482	2111.1409	2110.9411	94.6	70 -	88	0	---	R.YNQLMAASEAAINADPNMR.S + 2 Oxidation (M)

No match to: 841.1960, 841.1960, 842.5637, 855.0995, 871.0715, 882.6186, 882.6186, 893.0596, 909.0323, 915.1290, 915.1290, 927.5462, 947.0018, 1051.7703, 1060.1125, 1060.1126, 1083.1046, 1301.8031, 1301.8031, 1325.8025, 1339.7694, 1874.0144, 2124.1565, 2124.1565, 2151.1111, 2151.1111, 2212.2224, 2753.4365, 2753.4365

18. gi|487598108 **Mass:** 4479 **Score:** 48 **Expect:** 5.1e+002 **Matches:** 3
propanediol utilization ferredoxin, partial [Salmonella enterica]

Observed	Mr(expt)	Mr(calc)	ppm	Start	End	Miss	Ions	Peptide
2005.0609	2004.0536	2004.0905	-18.41	23 -	44	1	---	R.AAGVVGAGGAGFPAHVKLQAQV.-
2124.1565	2123.1492	2123.0164	62.6	2 -	20	1	21	M.STAINSVEMSLSADEIRER.V + Oxidation (M)
2124.1565	2123.1492	2123.0164	62.6	2 -	20	1	---	M.STAINSVEMSLSADEIRER.V + Oxidation (M)

No match to: 841.1960, 841.1960, 842.5637, 855.0995, 871.0715, 882.6186, 882.6186, 893.0596, 909.0323, 915.1290, 915.1290, 927.5462, 947.0018, 1051.7703, 1060.1125, 1060.1126, 1083.1046, 1169.6356, 1169.6356, 1301.8031, 1301.8031, 1313.8103, 1313.8103, 1325.8025, 1339.7694, 1674.8976, 1874.0144, 2112.1482, 2112.1482, 2151.1111, 2151.1111, 2212.2224, 2753.4365, 2753.4365

19. gi|401763462 **Mass:** 16967 **Score:** 48 **Expect:** 5.6e+002 **Matches:** 6
hypothetical protein ECENHK_09860 [Enterobacter cloacae subsp. cloacae ENHKU01]

Observed	Mr(expt)	Mr(calc)	ppm	Start	End	Miss	Ions	Peptide
842.5637	841.5564	841.5637	-8.60	77 -	84	0	---	K.IALATIIK.I
1169.6356	1168.6283	1168.6200	7.11	85 -	95	0	---	K.IGPGNIEINSR.R
1169.6356	1168.6283	1168.6135	12.7	33 -	42	1	---	K.RLISNHNGMK.K
1325.8025	1324.7952	1324.7211	55.9	85 -	96	1	---	K.IGPGNIEINSRR.V
2753.4365	2752.4292	2752.4125	6.06	110 -	132	0	---	K.QVQFYNNLTLWNHNFTAFLAAIK.R
2753.4365	2752.4292	2752.4125	6.07	110 -	132	0	---	K.QVQFYNNLTLWNHNFTAFLAAIK.R

No match to: 841.1960, 841.1960, 855.0995, 871.0715, 882.6186, 882.6186, 893.0596, 909.0323, 915.1290, 915.1290, 927.5462, 947.0018, 1051.7703, 1060.1125, 1060.1126, 1083.1046, 1301.8031, 1301.8031, 1313.8103, 1313.8103, 1339.7694, 1674.8976, 1874.0144, 2005.0609, 2112.1482, 2112.1482, 2124.1565, 2124.1565, 2151.1111, 2151.1111, 2212.2224

20. gi|553884411 **Mass:** 20219 **Score:** 47 **Expect:** 7.1e+002 **Matches:** 7
integrase [Gloeobacter sp. JS]

Observed	Mr(expt)	Mr(calc)	ppm	Start	End	Miss	Ions	Peptide
1169.6356	1168.6283	1168.5764	44.4	138 -	146	0	---	R.FIEEVYNQK.R
1169.6356	1168.6283	1168.5764	44.4	138 -	146	0	---	R.FIEEVYNQK.R
1301.8031	1300.7958	1300.6987	74.7	45 -	56	0	---	R.NIDGELTLSALR.M
1301.8031	1300.7958	1300.6987	74.7	45 -	56	0	---	R.NIDGELTLSALR.M

PDF-XChange Click to buy NOW! www.docu-track.com

1325.8025	1324.7952	1324.6775	88.9	138	147	1	---	R.FIEEVYNQKR.L
1874.0144	1873.0071	1872.9978	4.95	45	61	1	---	R.NIDGELTLSALRMALEK.R
2005.0609	2004.0536	2004.1044	-25.34	2	18	0	---	M.VLTGLNQLWISDITYIR.L

No match to: 841.1960, 841.1960, 842.5637, 855.0995, 871.0715, 882.6186, 882.6186, 893.0596, 909.0323, 915.1290, 915.1290, 927.5462, 947.0018, 1051.7703, 1060.1125, 1060.1126, 1083.1046, 1313.8103, 1313.8103, 1339.7694, 1674.8976, 2112.1482, 2112.1482, 2124.1565, 2124.1565, 2151.1111, 2151.1111, 2212.2224, 2753.4365, 2753.4365

21. gi|497783917 **Mass:** 30566 **Score:** 46 **Expect:** 8.7e+002 **Matches:** 9
Beta-lactamase, partial [Burkholderia ubonensis]

Observed	Mr(expt)	Mr(calc)	ppm	Start	End	Miss	Ions	Peptide
1313.8103	1312.8030	1312.7252	59.3	217	229	0	---	K.WLLGSANGVAGIR.T
1313.8103	1312.8030	1312.7252	59.3	217	229	0	---	K.WLLGSANGVAGIR.T
2112.1482	2111.1409	2111.0032	65.3	169	185	1	---	R.LDRWEPELNTARPDDER.D
2112.1482	2111.1409	2111.0032	65.3	169	185	1	---	R.LDRWEPELNTARPDDER.D
2151.1111	2150.1038	2150.1129	-4.22	71	90	1	---	R.ERFPLCGTFAVVAAAAMLAR.S
2151.1111	2150.1038	2150.1129	-4.21	71	90	1	---	R.ERFPLCGTFAVVAAAAMLAR.S
2212.2224	2211.2151	2211.1549	27.2	217	237	1	---	K.WLLGSANGVAGIRTGVPSDWR.V
2753.4365	2752.4292	2752.4483	-6.92	9	35	0	---	R.SLLLAAVAAPFVAACAPAPVGDHEHLR.A
2753.4365	2752.4292	2752.4483	-6.91	9	35	0	---	R.SLLLAAVAAPFVAACAPAPVGDHEHLR.A

No match to: 841.1960, 841.1960, 842.5637, 855.0995, 871.0715, 882.6186, 882.6186, 893.0596, 909.0323, 915.1290, 915.1290, 927.5462, 947.0018, 1051.7703, 1060.1125, 1060.1126, 1083.1046, 1169.6356, 1169.6356, 1301.8031, 1301.8031, 1325.8025, 1339.7694, 1674.8976, 1874.0144, 2005.0609, 2124.1565, 2124.1565

22. gi|150377816 **Mass:** 75741 **Score:** 46 **Expect:** 9.1e+002 **Matches:** 9
mechanosensitive ion channel MscS [Sinorhizobium medicae WSM419]

Observed	Mr(expt)	Mr(calc)	ppm	Start	End	Miss	Ions	Peptide
1301.8031	1300.7958	1300.7034	71.1	292	302	1	---	R.RPLVEMTAGRR.V + Oxidation (M)
1301.8031	1300.7958	1300.7034	71.1	292	302	1	12	R.RPLVEMTAGRR.V + Oxidation (M)
1313.8103	1312.8030	1312.7350	51.8	205	217	0	---	R.LLSAVASAVTEPR.Y
1313.8103	1312.8030	1312.7350	51.8	205	217	0	---	R.LLSAVASAVTEPR.Y
1339.7694	1338.7621	1338.7408	15.9	100	110	1	---	R.LVPEWIAARER.I
1674.8976	1673.8903	1673.8737	9.95	65	81	0	---	K.QTTATAAVIPPAGYASR.L
1874.0144	1873.0071	1873.0323	-13.42	390	406	1	---	K.LHGSPVIQGDVAKHIFR.G
2112.1482	2111.1409	2111.2024	-29.13	271	291	0	---	R.DLTAAVLGLVVLGMAIDAVVR.R + Oxidation (M)
2112.1482	2111.1409	2111.2024	-29.13	271	291	0	---	R.DLTAAVLGLVVLGMAIDAVVR.R + Oxidation (M)

No match to: 841.1960, 841.1960, 842.5637, 855.0995, 871.0715, 882.6186, 882.6186, 893.0596, 909.0323, 915.1290, 915.1290, 927.5462, 947.0018, 1051.7703, 1060.1125, 1060.1126, 1083.1046, 1169.6356, 1169.6356, 1325.8025, 2005.0609, 2124.1565, 2124.1565, 2151.1111, 2151.1111, 2212.2224, 2753.4365, 2753.4365

23. gi|332024455 **Mass:** 50459 **Score:** 46 **Expect:** 9.3e+002 **Matches:** 10
Dual specificity protein phosphatase 22 [Acromyrmex echinatior]

Observed	Mr(expt)	Mr(calc)	ppm	Start	End	Miss	Ions	Peptide
1301.8031	1300.7958	1300.6922	79.7	79	90	1	---	K.VPQVSGAKSCLR.T
1301.8031	1300.7958	1300.6922	79.7	79	90	1	---	K.VPQVSGAKSCLR.T
1339.7694	1338.7621	1338.6739	65.9	313	326	1	---	R.KSSTSSIGSVSSGR.T
1874.0144	1873.0071	1873.1374	-69.53	44	60	1	---	R.AQGARPPSVQLLLVLRR.F
2112.1482	2111.1409	2111.0508	42.7	358	379	1	---	R.SGPAGLHSYTGGSAPPSRAVSR.V
2112.1482	2111.1409	2111.0508	42.7	358	379	1	---	R.SGPAGLHSYTGGSAPPSRAVSR.V
2124.1565	2123.1492	2123.0317	55.4	257	275	1	---	R.FPSLALTESDAEACRSTLR.N
2124.1565	2123.1492	2123.0317	55.4	257	275	1	---	R.FPSLALTESDAEACRSTLR.N
2151.1111	2150.1038	2150.1419	-17.70	327	346	1	---	R.TPPQTPRMLPSAPPSPALHR.S
2151.1111	2150.1038	2150.1419	-17.69	327	346	1	---	R.TPPQTPRMLPSAPPSPALHR.S

No match to: 841.1960, 841.1960, 842.5637, 855.0995, 871.0715, 882.6186, 882.6186, 893.0596, 909.0323, 915.1290, 915.1290, 927.5462, 947.0018, 1051.7703, 1060.1125, 1060.1126, 1083.1046, 1169.6356, 1169.6356, 1313.8103, 1313.8103, 1325.8025, 1674.8976, 2005.0609, 2212.2224, 2753.4365, 2753.4365

24. gi|489455877 **Mass:** 40956 **Score:** 45 **Expect:** 9.8e+002 **Matches:** 9
thiouridylase [Clostridium botulinum]

Observed	Mr(expt)	Mr(calc)	ppm	Start	End	Miss	Ions	Peptide
927.5462	926.5389	926.4862	56.9	337	344	0	---	K.GQSVVFYK.D
1169.6356	1168.6283	1168.6241	3.65	236	245	0	---	K.GIVYYTIGQR.K
1169.6356	1168.6283	1168.6241	3.67	236	245	0	---	K.GIVYYTIGQR.K
1325.8025	1324.7952	1324.7463	36.9	313	324	1	---	K.AIITPIDDGRVR.V
1874.0144	1873.0071	1872.9945	6.74	306	322	1	---	R.YSQIPSKAIITPIDDGR.V
2124.1565	2123.1492	2123.1238	12.0	247	265	0	---	K.GLSIAFGKPMYVVDINPFR.N
2124.1565	2123.1492	2123.1238	12.0	247	265	0	---	K.GLSIAFGKPMYVVDINPFR.N
2151.1111	2150.1038	2150.1623	-27.22	337	356	1	---	K.GQSVVFYKDDLLIGGIIEK.-
2151.1111	2150.1038	2150.1623	-27.21	337	356	1	---	K.GQSVVFYKDDLLIGGIIEK.-

No match to: 841.1960, 841.1960, 842.5637, 855.0995, 871.0715, 882.6186, 882.6186, 893.0596, 909.0323, 915.1290, 915.1290, 947.0018, 1051.7703, 1060.1125, 1060.1126, 1083.1046, 1301.8031, 1301.8031, 1313.8103, 1313.8103, 1339.7694, 1674.8976, 2005.0609, 2112.1482, 2112.1482, 2212.2224, 2753.4365, 2753.4365

25. gi|486622113 **Mass:** 3493 **Score:** 45 **Expect:** 9.8e+002 **Matches:** 5
hypothetical protein [Escherichia coli]

Observed	Mr(expt)	Mr(calc)	ppm	Start	End	Miss	Ions	Peptide
1169.6356	1168.6283	1168.5665	52.9	22	30	1	---	R.ETLYRGFFH.-
1169.6356	1168.6283	1168.5665	52.9	22	30	1	---	R.ETLYRGFFH.-
1301.8031	1300.7958	1300.7391	43.6	2	13	0	---	M.LKPDSLPVTFGK.N
1301.8031	1300.7958	1300.7391	43.6	2	13	0	---	M.LKPDSLPVTFGK.N
2212.2224	2211.2151	2211.2263	-5.06	2	21	1	---	M.LKPDSLPVTFGKNDVEIIAR.E

No match to: 841.1960, 841.1960, 842.5637, 855.0995, 871.0715, 882.6186, 882.6186, 893.0596, 909.0323, 915.1290, 915.1290, 927.5462, 947.0018, 1051.7703, 1060.1125, 1060.1126, 1083.1046, 1313.8103, 1313.8103, 1325.8025, 1339.7694, 1674.8976, 1874.0144, 2005.0609, 2112.1482, 2112.1482, 2124.1565, 2124.1565, 2151.1111, 2151.1111, 2753.4365, 2753.4365

26. gi|546373423 **Mass:** 5165 **Score:** 45 **Expect:** 1.1e+003 **Matches:** 4
hypothetical protein [Prevotella sp. CAG:1058]

Observed	Mr(expt)	Mr(calc)	ppm	Start	End	Miss	Ions	Peptide
1874.0144	1873.0071	1872.8425	87.9	2	18	0	---	M.TKPATSSSYCFNGPSNR.T
2005.0609	2004.0536	2003.8829	85.2	1	18	0	---	-.MTKPATSSSYCFNGPSNR.T
2753.4365	2752.4292	2752.1707	93.9	23	48	0	---	R.LDGPFSYDAMVSGVPYSGDCIGVSMP.- + 2 Oxidation (M)

PDF-XChange Click to buy NOW! www.docu-track.com

PDF-XChange Click to buy NOW! www.docu-track.com

2753.4365	2752.4292	2752.1707	93.9	23 -	48	0	---	R.LDGPFSYDAMVSGVPYSGDCIGVSMP.- + 2 Oxidation (M)

No match to: 841.1960, 841.1960, 842.5637, 855.0995, 871.0715, 882.6186, 882.6186, 893.0596, 909.0323, 915.1290, 915.1290, 927.5462, 947.0018, 1051.7703, 1060.1125, 1060.1126, 1083.1046, 1169.6356, 1169.6356, 1301.8031, 1301.8031, 1313.8103, 1313.8103, 1325.8025, 1339.7694, 1674.8976, 2112.1482, 2112.1482, 2124.1565, 2124.1565, 2151.1111, 2151.1111, 2212.2224

27. gi|495396594 **Mass:** 25331 **Score:** 45 **Expect:** 1.2e+003 **Matches:** 7

peptide ABC transporter ATP-binding protein [[Bacteroides] pectinophilus]

Observed	Mr(expt)	Mr(calc)	ppm	Start	End	Miss	Ions	Peptide
1169.6356	1168.6283	1168.6889	-51.86	105 -	114	0	---	K.NILMPLLLDK.R
1169.6356	1168.6283	1168.6889	-51.84	105 -	114	0	---	K.NILMPLLLDK.R
1313.8103	1312.8030	1312.7788	18.4	104 -	114	1	---	R.KNILMPLLLDK.R + Oxidation (M)
1313.8103	1312.8030	1312.7788	18.4	104 -	114	1	---	R.KNILMPLLLDK.R + Oxidation (M)
1325.8025	1324.7952	1324.7900	3.91	105 -	115	1	---	K.NILMPLLLDKR.K
2124.1565	2123.1492	2123.2143	-30.65	86 -	103	1	---	R.KVGLVYQFYNLIPTLTVR.K
2124.1565	2123.1492	2123.2143	-30.65	87 -	104	1	---	K.VGLVYQFYNLIPTLTVRK.N

No match to: 841.1960, 841.1960, 842.5637, 855.0995, 871.0715, 882.6186, 882.6186, 893.0596, 909.0323, 915.1290, 915.1290, 927.5462, 947.0018, 1051.7703, 1060.1125, 1060.1126, 1083.1046, 1301.8031, 1301.8031, 1339.7694, 1674.8976, 1874.0144, 2005.0609, 2112.1482, 2112.1482, 2151.1111, 2151.1111, 2212.2224, 2753.4365, 2753.4365

28. gi|517950378 **Mass:** 44454 **Score:** 44 **Expect:** 1.2e+003 **Matches:** 9

hypothetical protein [Brevibacillus sp. phR]

Observed	Mr(expt)	Mr(calc)	ppm	Start	End	Miss	Ions	Peptide
842.5637	841.5564	841.5273	34.6	399 -	406	0	---	K.LLDAVLAK.I
1169.6356	1168.6283	1168.6023	22.3	36 -	45	0	---	R.VHVIMTENAR.K
1169.6356	1168.6283	1168.6023	22.3	36 -	45	0	---	R.VHVIMTENAR.K
1313.8103	1312.8030	1312.6921	84.5	36 -	46	1	---	R.VHVIMTENARK.F + Oxidation (M)
1313.8103	1312.8030	1312.6921	84.5	36 -	46	1	---	R.VHVIMTENARK.F + Oxidation (M)
1339.7694	1338.7621	1338.6350	95.0	226 -	237	1	---	K.MGYAIAEAARDR.G + Oxidation (M)
2005.0609	2004.0536	2004.1619	-54.03	8 -	28	1	---	K.TIVLGVTGGIAAYKAAALTSK.L
2124.1565	2123.1492	2123.1660	-7.92	1 -	21	1	---	-.MQSLTGKTIVLGVTGGIAAYK.A + Oxidation (M)
2124.1565	2123.1492	2123.1660	-7.92	1 -	21	1	---	-.MQSLTGKTIVLGVTGGIAAYK.A + Oxidation (M)

No match to: 841.1960, 841.1960, 855.0995, 871.0715, 882.6186, 882.6186, 893.0596, 909.0323, 915.1290, 915.1290, 927.5462, 947.0018, 1051.7703, 1060.1125, 1060.1126, 1083.1046, 1301.8031, 1301.8031, 1325.8025, 1674.8976, 1874.0144, 2112.1482, 2112.1482, 2151.1111, 2151.1111, 2212.2224, 2753.4365, 2753.4365

29. gi|493698297 **Mass:** 5437 **Score:** 44 **Expect:** 1.2e+003 **Matches:** 4

hypothetical protein [Haloterrigena thermotolerans]

Observed	Mr(expt)	Mr(calc)	ppm	Start	End	Miss	Ions	Peptide
1874.0144	1873.0071	1872.8523	82.7	2 -	17	0	---	M.TDAAECRPEPLEQETK.E
2005.0609	2004.0536	2003.8928	80.3	1 -	17	0	---	-.MTDAAECRPEPLEQETK.E
2753.4365	2752.4292	2752.1922	86.1	20 -	43	0	---	R.DDAHLDDIEEGAGCTEIWEHLAEK.R
2753.4365	2752.4292	2752.1922	86.1	20 -	43	0	---	R.DDAHLDDIEEGAGCTEIWEHLAEK.R

No match to: 841.1960, 841.1960, 842.5637, 855.0995, 871.0715, 882.6186, 882.6186, 893.0596, 909.0323, 915.1290, 915.1290, 927.5462, 947.0018, 1051.7703, 1060.1125, 1060.1126, 1083.1046, 1169.6356, 1169.6356, 1301.8031, 1301.8031, 1313.8103, 1313.8103, 1325.8025, 1339.7694, 1674.8976, 2112.1482, 2112.1482, 2124.1565, 2124.1565, 2151.1111, 2151.1111, 2212.2224

30. gi|163839610 **Mass:** 17902 **Score:** 44 **Expect:** 1.2e+003 **Matches:** 7

nucleotide-binding protein [Renibacterium salmoninarum ATCC 33209]

Observed	Mr(expt)	Mr(calc)	ppm	Start	End	Miss	Ions	Peptide
927.5462	926.5389	926.5185	22.0	113 -	120	1	---	K.LIRDEGPK.G
1169.6356	1168.6283	1168.5876	34.8	28 -	36	1	---	K.EIAQRYDFK.G
1169.6356	1168.6283	1168.5876	34.8	28 -	36	1	---	K.EIAQRYDFK.G
1313.8103	1312.8030	1312.7099	70.9	121 -	132	1	---	K.GVKAQIQGDELR.V
1313.8103	1312.8030	1312.6735	98.7	16 -	27	0	---	K.QEVANALNQAQK.E
2753.4365	2752.4292	2752.3742	20.0	139 -	162	1	---	R.DDLQATMTLLKGFDEADLQFVNLR.-
2753.4365	2752.4292	2752.3742	20.0	139 -	162	1	---	R.DDLQATMTLLKGFDEADLQFVNLR.-

No match to: 841.1960, 841.1960, 842.5637, 855.0995, 871.0715, 882.6186, 882.6186, 893.0596, 909.0323, 915.1290, 915.1290, 947.0018, 1051.7703, 1060.1125, 1060.1126, 1083.1046, 1301.8031, 1301.8031, 1325.8025, 1339.7694, 1674.8976, 1874.0144, 2005.0609, 2112.1482, 2112.1482, 2124.1565, 2124.1565, 2151.1111, 2151.1111, 2212.2224

31. gi|548180461 **Mass:** 10887 **Score:** 44 **Expect:** 1.3e+003 **Matches:** 7

uncharacterized protein [Bacteroides uniformis CAG:3]

Observed	Mr(expt)	Mr(calc)	ppm	Start	End	Miss	Ions	Peptide
1169.6356	1168.6283	1168.5585	59.8	2 -	10	1	---	M.ERHELENSR.E
1169.6356	1168.6283	1168.5585	59.8	2 -	10	1	---	M.ERHELENSR.E
1874.0144	1873.0071	1872.8754	70.3	17 -	31	1	---	K.ELEHALNDYGWNEKR.F
2124.1565	2123.1492	2123.1276	10.2	32 -	49	1	---	R.FASAVTTFHRTLQQTLFR.S
2124.1565	2123.1492	2123.1276	10.2	32 -	49	1	---	R.FASAVTTFHRTLQQTLFR.S
2151.1111	2150.1038	2150.0024	47.2	50 -	68	1	---	R.SMVEVIKTMGSEGYGYDLR.N + Oxidation (M)
2151.1111	2150.1038	2150.0024	47.2	50 -	68	1	---	R.SMVEVIKTMGSEGYGYDLR.N + Oxidation (M)

No match to: 841.1960, 841.1960, 842.5637, 855.0995, 871.0715, 882.6186, 882.6186, 893.0596, 909.0323, 915.1290, 915.1290, 927.5462, 947.0018, 1051.7703, 1060.1125, 1060.1126, 1083.1046, 1301.8031, 1301.8031, 1313.8103, 1313.8103, 1325.8025, 1339.7694, 1674.8976, 2005.0609, 2112.1482, 2112.1482, 2212.2224, 2753.4365, 2753.4365

32. gi|339499150 **Mass:** 28813 **Score:** 44 **Expect:** 1.3e+003 **Matches:** 8

short-chain dehydrogenase/reductase SDR [Treponema caldaria DSM 7334]

Observed	Mr(expt)	Mr(calc)	ppm	Start	End	Miss	Ions	Peptide
882.6186	881.6113	881.5334	88.3	180 -	187	0	---	K.SAIIHLTK.Y
882.6186	881.6113	881.5334	88.4	180 -	187	0	---	K.SAIIHLTK.Y
1169.6356	1168.6283	1168.7180	-76.71	15 -	27	0	---	R.IVVVGGAGLLGSK.I
1169.6356	1168.6283	1168.7180	-76.69	15 -	27	0	---	R.IVVVGGAGLLGSK.I
1325.8025	1324.7952	1324.8191	-18.00	14 -	27	1	---	K.RIVVVGGAGLLGSK.I
1874.0144	1873.0071	1872.9001	57.1	163 -	179	0	---	R.IYDGTAMTMPVEYAAIK.S
2753.4365	2752.4292	2752.4179	4.11	163 -	187	1	---	R.IYDGTAMTMPVEYAAIKSAIIHLTK.Y + Oxidation (M)
2753.4365	2752.4292	2752.4179	4.11	163 -	187	1	---	R.IYDGTAMTMPVEYAAIKSAIIHLTK.Y + Oxidation (M)

No match to: 841.1960, 841.1960, 842.5637, 855.0995, 871.0715, 893.0596, 909.0323, 915.1290, 915.1290, 927.5462, 947.0018, 1051.7703, 1060.1125, 1060.1126, 1083.1046, 1301.8031, 1301.8031, 1313.8103, 1313.8103, 1339.7694, 1674.8976, 2005.0609, 2112.1482, 2112.1482, 2124.1565, 2124.1565, 2151.1111, 2151.1111, 2212.2224

33. gi|522141702 **Mass:** 27885 **Score:** 44 **Expect:** 1.4e+003 **Matches:** 8

PDF-XChange Click to buy NOW! www.docu-track.com

hypothetical protein [Massilia niastensis]

Observed	Mr(expt)	Mr(calc)	ppm	Start	End	Miss	Ions	Peptide
842.5637	841.5564	841.5022	64.5	145	152	0	---	R.AVVGLLDR.M
1313.8103	1312.8030	1312.7285	56.7	145	156	1	---	R.AVVGLLDRMSPR.H
1313.8103	1312.8030	1312.7285	56.7	145	156	1	---	R.AVVGLLDRMSPR.H
1674.8976	1673.8903	1673.9213	-18.50	138	152	1	---	R.YQSGLQRAVVGLLDR.M
2124.1565	2123.1492	2123.0912	27.3	3	21	1	---	K.HLPPSHGTVIDGQTFRFSK.S
2124.1565	2123.1492	2123.0912	27.3	3	21	1	---	K.HLPPSHGTVIDGQTFRFSK.S
2753.4365	2752.4292	2752.4218	2.71	222	245	1	---	R.ILAERSTHFPQMSEPELVLEALAR.L + Oxidation (M)
2753.4365	2752.4292	2752.4218	2.72	222	245	1	---	R.ILAERSTHFPQMSEPELVLEALAR.L + Oxidation (M)

No match to: 841.1960, 841.1960, 855.0995, 871.0715, 882.6186, 882.6186, 893.0596, 909.0323, 915.1290, 915.1290, 927.5462, 947.0018, 1051.7703, 1060.1125, 1060.1126, 1083.1046, 1169.6356, 1169.6356, 1301.8031, 1301.8031, 1325.8025, 1339.7694, 1874.0144, 2005.0609, 2112.1482, 2112.1482, 2151.1111, 2151.1111, 2212.2224

34. gi|497966698 **Mass:** 29777 **Score:** 44 **Expect:** 1.5e+003 **Matches:** 7

peptide ABC transporter ATP-binding protein [Streptomyces sp. SA3_actF]

Observed	Mr(expt)	Mr(calc)	ppm	Start	End	Miss	Ions	Peptide
1301.8031	1300.7958	1300.7537	32.4	155	166	1	---	R.VISMVGLEGRLK.H
1301.8031	1300.7958	1300.7537	32.4	155	166	1	---	R.VISMVGLEGRLK.H
1874.0144	1873.0071	1872.9768	16.2	2	18	0	---	M.ACVATVPVSQEFPLVTR.L
2005.0609	2004.0536	2004.0173	18.1	1	18	0	---	-.MACVATVPVSQEFPLVTR.L
2151.1111	2150.1038	2150.1888	-39.54	115	133	1	---	R.DKIGFVFQAFNLLPTLTAR.E
2151.1111	2150.1038	2150.1888	-39.53	115	133	1	---	R.DKIGFVFQAFNLLPTLTAR.E
2212.2224	2211.2151	2211.1106	47.3	241	260	1	---	R.VVFLADGRIVDEMHGPTAER.V

No match to: 841.1960, 841.1960, 842.5637, 855.0995, 871.0715, 882.6186, 882.6186, 893.0596, 909.0323, 915.1290, 915.1290, 927.5462, 947.0018, 1051.7703, 1060.1125, 1060.1126, 1083.1046, 1169.6356, 1169.6356, 1313.8103, 1313.8103, 1325.8025, 1339.7694, 1674.8976, 2112.1482, 2112.1482, 2124.1565, 2124.1565, 2753.4365, 2753.4365

35. gi|547298347 **Mass:** 24974 **Score:** 43 **Expect:** 1.5e+003 **Matches:** 6

lipoprotein ABC transporter ATP-binding protein LolD [Clostridium hathewayi CAG:224]

Observed	Mr(expt)	Mr(calc)	ppm	Start	End	Miss	Ions	Peptide
1169.6356	1168.6283	1168.6889	-51.86	105	114	0	---	K.NILMPLLLDK.K
1169.6356	1168.6283	1168.6889	-51.84	105	114	0	---	K.NILMPLLLDK.K
1313.8103	1312.8030	1312.7788	18.4	105	115	1	---	K.NILMPLLLDKK.S + Oxidation (M)
1313.8103	1312.8030	1312.7788	18.4	104	114	1	---	R.KNILMPLLLDK.K + Oxidation (M)
2124.1565	2123.1492	2123.2143	-30.65	86	103	1	---	R.KVGLVYQFYNLIPTLTVR.K
2124.1565	2123.1492	2123.2143	-30.65	87	104	1	---	K.VGLVYQFYNLIPTLTVRK.N

No match to: 841.1960, 841.1960, 842.5637, 855.0995, 871.0715, 882.6186, 882.6186, 893.0596, 909.0323, 915.1290, 915.1290, 927.5462, 947.0018, 1051.7703, 1060.1125, 1060.1126, 1083.1046, 1301.8031, 1301.8031, 1325.8025, 1339.7694, 1674.8976, 1874.0144, 2005.0609, 2112.1482, 2112.1482, 2151.1111, 2151.1111, 2212.2224, 2753.4365, 2753.4365

36. gi|496545606 **Mass:** 25186 **Score:** 43 **Expect:** 1.5e+003 **Matches:** 7

peptide ABC transporter ATP-binding protein [Lachnospiraceae bacterium 3_1_57FAA_CT1]

Observed	Mr(expt)	Mr(calc)	ppm	Start	End	Miss	Ions	Peptide
1169.6356	1168.6283	1168.6889	-51.86	105	114	0	---	K.NILMPLLLDK.R
1169.6356	1168.6283	1168.6889	-51.84	105	114	0	---	K.NILMPLLLDK.R
1313.8103	1312.8030	1312.7788	18.4	104	114	1	---	R.KNILMPLLLDK.R + Oxidation (M)
1313.8103	1312.8030	1312.7788	18.4	104	114	1	---	R.KNILMPLLLDK.R + Oxidation (M)
1325.8025	1324.7952	1324.7900	3.91	105	115	1	---	K.NILMPLLLDKR.K
2124.1565	2123.1492	2123.2143	-30.65	86	103	1	---	R.KVGLVYQFYNLIPTLTVR.K
2124.1565	2123.1492	2123.2143	-30.65	87	104	1	---	K.VGLVYQFYNLIPTLTVRK.N

No match to: 841.1960, 841.1960, 842.5637, 855.0995, 871.0715, 882.6186, 882.6186, 893.0596, 909.0323, 915.1290, 915.1290, 927.5462, 947.0018, 1051.7703, 1060.1125, 1060.1126, 1083.1046, 1301.8031, 1301.8031, 1339.7694, 1674.8976, 1874.0144, 2005.0609, 2112.1482, 2112.1482, 2151.1111, 2151.1111, 2212.2224, 2753.4365, 2753.4365

37. gi|479202615 **Mass:** 25300 **Score:** 43 **Expect:** 1.5e+003 **Matches:** 7

ABC-type antimicrobial peptide transport system, ATPase component [Roseburia intestinalis M50/1]

Observed	Mr(expt)	Mr(calc)	ppm	Start	End	Miss	Ions	Peptide
1169.6356	1168.6283	1168.6889	-51.86	105	114	0	---	K.NILMPLLLDK.R
1169.6356	1168.6283	1168.6889	-51.84	105	114	0	---	K.NILMPLLLDK.R
1313.8103	1312.8030	1312.7788	18.4	104	114	1	---	R.KNILMPLLLDK.R + Oxidation (M)
1313.8103	1312.8030	1312.7788	18.4	104	114	1	---	R.KNILMPLLLDK.R + Oxidation (M)
1325.8025	1324.7952	1324.7900	3.91	105	115	1	---	K.NILMPLLLDKR.K
2124.1565	2123.1492	2123.2143	-30.65	86	103	1	---	R.KVGLVYQFYNLIPTLTVR.K
2124.1565	2123.1492	2123.2143	-30.65	87	104	1	---	K.VGLVYQFYNLIPTLTVRK.N

No match to: 841.1960, 841.1960, 842.5637, 855.0995, 871.0715, 882.6186, 882.6186, 893.0596, 909.0323, 915.1290, 915.1290, 927.5462, 947.0018, 1051.7703, 1060.1125, 1060.1126, 1083.1046, 1301.8031, 1301.8031, 1339.7694, 1674.8976, 1874.0144, 2005.0609, 2112.1482, 2112.1482, 2151.1111, 2151.1111, 2212.2224, 2753.4365, 2753.4365

38. gi|296132158 **Mass:** 47550 **Score:** 43 **Expect:** 1.5e+003 **Matches:** 8

respiratory-chain NADH dehydrogenase domain 51 kDa subunit [Thermincola potens JR]

Observed	Mr(expt)	Mr(calc)	ppm	Start	End	Miss	Ions	Peptide
1313.8103	1312.8030	1312.6921	84.5	237	247	1	---	R.KEITMAHTLNR.A
1313.8103	1312.8030	1312.6921	84.5	237	247	1	---	R.KEITMAHTLNR.A
1325.8025	1324.7952	1324.6874	81.4	185	196	0	---	R.ELVETANPVPEK.Y
1674.8976	1673.8903	1673.8130	46.2	258	270	1	---	K.CTEICPRYLLGHR.M
1874.0144	1873.0071	1873.1149	-57.53	220	236	1	---	K.ITKGLIVLPENHSLLAR.K
2151.1111	2150.1038	2150.2463	-66.25	355	373	1	---	R.LIIRLNLESYNLPAPLSPK.S
2151.1111	2150.1038	2150.2463	-66.24	355	373	1	---	R.LIIRLNLESYNLPAPLSPK.S
2212.2224	2211.2151	2211.1279	39.4	199	219	0	---	K.VIEGGPMMGTVLENPDKPVTK.I

No match to: 841.1960, 841.1960, 842.5637, 855.0995, 871.0715, 882.6186, 882.6186, 893.0596, 909.0323, 915.1290, 915.1290, 927.5462, 947.0018, 1051.7703, 1060.1125, 1060.1126, 1083.1046, 1169.6356, 1169.6356, 1301.8031, 1301.8031, 1339.7694, 2005.0609, 2112.1482, 2112.1482, 2124.1565, 2124.1565, 2753.4365, 2753.4365

39. gi|494243326 **Mass:** 26715 **Score:** 43 **Expect:** 1.6e+003 **Matches:** 7

short-chain dehydrogenase [Methylophaga aminisulfidivorans]

Observed	Mr(expt)	Mr(calc)	ppm	Start	End	Miss	Ions	Peptide
927.5462	926.5389	926.5120	29.1	14	21	1	---	R.VGLHMAKR.F + Oxidation (M)
1313.8103	1312.8030	1312.6995	78.8	154	165	0	---	K.AGLHNMMLVLAK.K + Oxidation (M)
1313.8103	1312.8030	1312.6995	78.8	154	165	0	---	K.AGLHNMMLVLAK.K + Oxidation (M)

PDF-XChange Click to buy NOW! www.docu-track.com

2124.1565	2123.1492	2123.0647	39.8	173 - 192	0	---	K.VNAVAPGPVLFTESHSDEVR.Q
2124.1565	2123.1492	2123.0647	39.8	173 - 192	0	---	K.VNAVAPGPVLFTESHSDEVR.Q
2753.4365	2752.4292	2752.4184	3.92	167 - 192	1	---	K.YAPAVKVNAVAPGPVLFTESHSDEVR.Q
2753.4365	2752.4292	2752.2432	67.6	35 - 58	1	---	R.TETDGVQEMREMGIHTIQADFNSK.Q + Oxidation (M)

No match to: 841.1960, 841.1960, 842.5637, 855.0995, 871.0715, 882.6186, 882.6186, 893.0596, 909.0323, 915.1290, 915.1290, 947.0018, 1051.7703, 1060.1125, 1060.1126, 1083.1046, 1169.6356, 1169.6356, 1301.8031, 1301.8031, 1325.8025, 1339.7694, 1674.8976, 1874.0144, 2005.0609, 2112.1482, 2112.1482, 2151.1111, 2151.1111, 2212.2224

40. gi|491299814 **Mass:** 25459 **Score:** 43 **Expect:** 1.7e+003 **Matches:** 7
hypothetical protein [Amycolatopsis azurea]

Observed	Mr(expt)	Mr(calc)	ppm	Start - End	Miss	Ions	Peptide
842.5637	841.5564	841.5134	51.2	206 - 212	1	---	R.AVVRELR.A
1874.0144	1873.0071	1872.9000	57.2	46 - 63	1	---	R.RQADACDVIVAEGVGDAK.S
2112.1482	2111.1409	2111.2136	-34.44	1 - 19	1	---	-.MEIIVSNELGVRAVVLTLR.A
2112.1482	2111.1409	2111.2136	-34.43	1 - 19	1	---	-.MEIIVSNELGVRAVVLTLR.A
2151.1111	2150.1038	2150.1095	-2.66	29 - 46	1	---	R.LFPMVHIGTQAYFDTVRR.Q
2151.1111	2150.1038	2150.1095	-2.65	29 - 46	1	---	R.LFPMVHIGTQAYFDTVRR.Q
2212.2224	2211.2151	2211.1908	11.0	120 - 139	1	---	R.LMMSLLAPTLGVFLRGFGTR.G + 2 Oxidation (M)

No match to: 841.1960, 841.1960, 855.0995, 871.0715, 882.6186, 882.6186, 893.0596, 909.0323, 915.1290, 915.1290, 927.5462, 947.0018, 1051.7703, 1060.1125, 1060.1126, 1083.1046, 1169.6356, 1169.6356, 1301.8031, 1301.8031, 1313.8103, 1313.8103, 1325.8025, 1339.7694, 1674.8976, 2005.0609, 2124.1565, 2124.1565, 2753.4365, 2753.4365

41. gi|312112921 **Mass:** 46524 **Score:** 43 **Expect:** 1.7e+003 **Matches:** 9
lytic murein transglycosylase [Rhodomicrobium vannielii ATCC 17100]

Observed	Mr(expt)	Mr(calc)	ppm	Start - End	Miss	Ions	Peptide
842.5637	841.5564	841.5134	51.2	114 - 121	1	---	K.ARQLIGGK.Y
1169.6356	1168.6283	1168.5546	63.1	162 - 171	0	---	R.SLATLAYDCR.R
1169.6356	1168.6283	1168.5546	63.1	162 - 171	0	---	R.SLATLAYDCR.R
2005.0609	2004.0536	2004.0428	5.38	233 - 251	1	---	R.SAPDVIGSSANYLKALGWR.R
2151.1111	2150.1038	2150.0103	43.5	90 - 108	1	---	R.GQSVFSQSFLEFSDRMAAK.Y + Oxidation (M)
2151.1111	2150.1038	2150.0103	43.5	90 - 108	1	---	R.GQSVFSQSFLEFSDRMAAK.Y + Oxidation (M)
2212.2224	2211.2151	2211.1899	11.4	395 - 414	1	---	K.VQQKLGLPVDSYPSPELVSR.L
2753.4365	2752.4292	2752.2915	50.0	262 - 284	0	---	R.VPESMDWSQADLTIQHPLSEWQR.M
2753.4365	2752.4292	2752.2915	50.0	262 - 284	0	---	R.VPESMDWSQADLTIQHPLSEWQR.M

No match to: 841.1960, 841.1960, 855.0995, 871.0715, 882.6186, 882.6186, 893.0596, 909.0323, 915.1290, 915.1290, 927.5462, 947.0018, 1051.7703, 1060.1125, 1060.1126, 1083.1046, 1301.8031, 1301.8031, 1313.8103, 1313.8103, 1325.8025, 1339.7694, 1674.8976, 1874.0144, 2112.1482, 2112.1482, 2124.1565, 2124.1565

42. gi|396482791 **Mass:** 59448 **Score:** 43 **Expect:** 1.7e+003 **Matches:** 5
predicted protein [Leptosphaeria maculans JN3]

Observed	Mr(expt)	Mr(calc)	ppm	Start - End	Miss	Ions	Peptide
1169.6356	1168.6283	1168.6313	-2.51	412 - 423	1	---	R.AAVATGRASHTK.Q
1169.6356	1168.6283	1168.6313	-2.49	412 - 423	1	18	R.AAVATGRASHTK.Q
2212.2224	2211.2151	2211.1106	47.3	1 - 20	0	---	-.MTLATNSEAKPPLASSVWHR.G + Oxidation (M)
2753.4365	2752.4292	2752.3755	19.5	113 - 138	1	---	R.ELGTEGGVGGLVEPAWGSLAPCVWRR.A
2753.4365	2752.4292	2752.3755	19.5	112 - 137	1	---	R.RELGTEGGVGGLVEPAWGSLAPCVWR.R

No match to: 841.1960, 841.1960, 842.5637, 855.0995, 871.0715, 882.6186, 882.6186, 893.0596, 909.0323, 915.1290, 915.1290, 927.5462, 947.0018, 1051.7703, 1060.1125, 1060.1126, 1083.1046, 1301.8031, 1301.8031, 1313.8103, 1313.8103, 1325.8025, 1339.7694, 1674.8976, 1874.0144, 2005.0609, 2112.1482, 2112.1482, 2124.1565, 2124.1565, 2151.1111, 2151.1111

43. gi|550962788 **Mass:** 10224 **Score:** 43 **Expect:** 1.8e+003 **Matches:** 4
hypothetical protein [Pseudochrobactrum sp. AO18b]

Observed	Mr(expt)	Mr(calc)	ppm	Start - End	Miss	Ions	Peptide
1169.6356	1168.6283	1168.6676	-33.64	18 - 28	1	---	R.AQLTPSGLARR.A
1169.6356	1168.6283	1168.6676	-33.62	17 - 27	1	---	K.RAQLTPSGLAR.R
1674.8976	1673.8903	1673.8413	29.3	2 - 16	0	---	M.FTHAELWSAIDSVAK.R
2212.2224	2211.2151	2211.1167	44.5	59 - 78	0	---	K.ILEVTAMDVMTFAQLLAGEK.S + 2 Oxidation (M)

No match to: 841.1960, 841.1960, 842.5637, 855.0995, 871.0715, 882.6186, 882.6186, 893.0596, 909.0323, 915.1290, 915.1290, 927.5462, 947.0018, 1051.7703, 1060.1125, 1060.1126, 1083.1046, 1301.8031, 1301.8031, 1313.8103, 1313.8103, 1325.8025, 1339.7694, 1874.0144, 2005.0609, 2112.1482, 2112.1482, 2124.1565, 2124.1565, 2151.1111, 2151.1111, 2753.4365, 2753.4365

44. gi|297182578 **Mass:** 12830 **Score:** 43 **Expect:** 1.8e+003 **Matches:** 5
transposase and inactivated derivatives [uncultured Rhizobiales bacterium HF4000_32B18]

Observed	Mr(expt)	Mr(calc)	ppm	Start - End	Miss	Ions	Peptide
1339.7694	1338.7621	1338.7619	0.15	22 - 35	0	---	K.GIAGLAAAAQEVLR.Q
1874.0144	1873.0071	1872.9556	27.5	88 - 103	0	---	R.LTAAQLAMLWEGIDWR.R
2005.0609	2004.0536	2004.0179	17.8	54 - 69	1	---	R.LKLLTWDGQGFCLYYK.V
2151.1111	2150.1038	2150.0435	28.0	1 - 20	1	---	-.MIGPGTGVRVYLACGPTDMR.K
2151.1111	2150.1038	2150.0435	28.1	1 - 20	1	---	-.MIGPGTGVRVYLACGPTDMR.K

No match to: 841.1960, 841.1960, 842.5637, 855.0995, 871.0715, 882.6186, 882.6186, 893.0596, 909.0323, 915.1290, 915.1290, 927.5462, 947.0018, 1051.7703, 1060.1125, 1060.1126, 1083.1046, 1169.6356, 1169.6356, 1301.8031, 1301.8031, 1313.8103, 1313.8103, 1325.8025, 1674.8976, 2112.1482, 2112.1482, 2124.1565, 2124.1565, 2212.2224, 2753.4365, 2753.4365

45. gi|341890310 **Mass:** 11784 **Score:** 43 **Expect:** 1.9e+003 **Matches:** 5
hypothetical protein CAEBREN_08480 [Caenorhabditis brenneri]

Observed	Mr(expt)	Mr(calc)	ppm	Start - End	Miss	Ions	Peptide
842.5637	841.5564	841.5385	21.3	65 - 71	1	---	K.IAIRIEK.H
1169.6356	1168.6283	1168.5836	38.2	23 - 32	0	---	K.IELNHSVDSR.R
1169.6356	1168.6283	1168.5836	38.3	23 - 32	0	---	K.IELNHSVDSR.R
1325.8025	1324.7952	1324.6847	83.4	23 - 33	1	---	K.IELNHSVDSRR.I
1339.7694	1338.7621	1338.6667	71.3	48 - 59	1	---	K.KSSDGFTEDLIK.V

No match to: 841.1960, 841.1960, 855.0995, 871.0715, 882.6186, 882.6186, 893.0596, 909.0323, 915.1290, 915.1290, 927.5462, 947.0018, 1051.7703, 1060.1125, 1060.1126, 1083.1046, 1301.8031, 1301.8031, 1313.8103, 1313.8103, 1674.8976, 1874.0144, 2005.0609, 2112.1482, 2112.1482, 2124.1565, 2124.1565, 2151.1111, 2151.1111, 2212.2224, 2753.4365, 2753.4365

46. gi|123189376 **Mass:** 27775 **Score:** 43 **Expect:** 1.9e+003 **Matches:** 7
hypothetical protein [Trichomonas vaginalis G3]

Observed	Mr(expt)	Mr(calc)	ppm	Start - End	Miss	Ions	Peptide
1301.8031	1300.7958	1300.6744	93.4	141 - 150	1	---	K.DMVMLHLVRR.N + 2 Oxidation (M)
1301.8031	1300.7958	1300.6744	93.4	141 - 150	1	---	K.DMVMLHLVRR.N + 2 Oxidation (M)

PDF-XChange Click to buy NOW! www.docu-track.com

1874.0144	1873.0071	1872.8750	70.5	2 - 17	0	---	M.YQMQSILTACFAPDTK.L
2005.0609	2004.0536	2003.9155	68.9	1 - 17	0	---	-.MYQMQSILTACFAPDTK.L
2212.2224	2211.2151	2211.1068	49.0	2 - 20	1	---	M.YQMQSILTACFAPDTKLPK.D
2753.4365	2752.4292	2752.3564	26.4	186 - 208	1	---	K.QIVQDTLPQTHYDMKGCTILTFK.E + Oxidation (M)
2753.4365	2752.4292	2752.3564	26.4	186 - 208	1	---	K.QIVQDTLPQTHYDMKGCTILTFK.E + Oxidation (M)

No match to: 841.1960, 841.1960, 842.5637, 855.0995, 871.0715, 882.6186, 882.6186, 893.0596, 909.0323, 915.1290, 915.1290, 927.5462, 947.0018, 1051.7703, 1060.1125, 1060.1126, 1083.1046, 1169.6356, 1169.6356, 1313.8103, 1313.8103, 1325.8025, 1339.7694, 1674.8976, 2112.1482, 2112.1482, 2124.1565, 2124.1565, 2151.1111, 2151.1111

47. gi|493826333 **Mass:** 24992 **Score:** 42 **Expect:** 1.9e+003 **Matches:** 6
peptide ABC transporter ATP-binding protein [Clostridium hathewayi]

Observed	Mr(expt)	Mr(calc)	ppm	Start - End	Miss	Ions	Peptide
1169.6356	1168.6283	1168.6889	-51.86	105 - 114	0	---	K.NILMPLLLDK.K
1169.6356	1168.6283	1168.6889	-51.84	105 - 114	0	---	K.NILMPLLLDK.K
1313.8103	1312.8030	1312.7788	18.4	105 - 115	1	---	K.NILMPLLLDKK.S + Oxidation (M)
1313.8103	1312.8030	1312.7788	18.4	104 - 114	1	---	R.KNILMPLLLDK.K + Oxidation (M)
2124.1565	2123.1492	2123.2143	-30.65	86 - 103	1	---	R.KVGLVYQFYNLIPTLTVR.K
2124.1565	2123.1492	2123.2143	-30.65	87 - 104	1	---	K.VGLVYQFYNLIPTLTVRK.N

No match to: 841.1960, 841.1960, 842.5637, 855.0995, 871.0715, 882.6186, 882.6186, 893.0596, 909.0323, 915.1290, 915.1290, 927.5462, 947.0018, 1051.7703, 1060.1125, 1060.1126, 1083.1046, 1301.8031, 1301.8031, 1325.8025, 1339.7694, 1674.8976, 1874.0144, 2005.0609, 2112.1482, 2112.1482, 2151.1111, 2151.1111, 2212.2224, 2753.4365, 2753.4365

48. gi|332235189 **Mass:** 5332 **Score:** 42 **Expect:** 1.9e+003 **Matches:** 3
PREDICTED: protein phosphatase 1 regulatory subunit 14D [Nomascus leucogenys]

Observed	Mr(expt)	Mr(calc)	ppm	Start - End	Miss	Ions	Peptide
1874.0144	1873.0071	1872.9792	14.9	2 - 17	1	---	M.DLSTEEQKTQLEAILR.N
2005.0609	2004.0536	2004.0197	16.9	1 - 17	1	---	-.MDLSTEEQKTQLEAILR.N
2212.2224	2211.2151	2211.1358	35.9	18 - 36	1	---	R.NCPHPTEAFISELLSQLKK.L

No match to: 841.1960, 841.1960, 842.5637, 855.0995, 871.0715, 882.6186, 882.6186, 893.0596, 909.0323, 915.1290, 915.1290, 927.5462, 947.0018, 1051.7703, 1060.1125, 1060.1126, 1083.1046, 1169.6356, 1169.6356, 1301.8031, 1301.8031, 1313.8103, 1313.8103, 1325.8025, 1339.7694, 1674.8976, 2112.1482, 2112.1482, 2124.1565, 2124.1565, 2151.1111, 2151.1111, 2753.4365, 2753.4365

49. gi|520945468 **Mass:** 15697 **Score:** 42 **Expect:** 1.9e+003 **Matches:** 7
LysR family transcriptional regulator, partial [Pseudomonas syringae]

Observed	Mr(expt)	Mr(calc)	ppm	Start - End	Miss	Ions	Peptide
1169.6356	1168.6283	1168.5665	52.9	129 - 138	0	---	R.EYFAVHHAPV.-
1169.6356	1168.6283	1168.5665	52.9	129 - 138	0	---	R.EYFAVHHAPV.-
1313.8103	1312.8030	1312.7616	31.6	75 - 85	1	---	R.GVLLRSLWDVR.T
1313.8103	1312.8030	1312.7616	31.6	75 - 85	1	---	R.GVLLRSLWDVR.T
2112.1482	2111.1409	2111.0548	40.8	35 - 53	0	---	R.DRPFGIWHLNGSQGAETVK.V
2112.1482	2111.1409	2111.0548	40.8	35 - 53	0	---	R.DRPFGIWHLNGSQGAETVK.V
2212.2224	2211.2151	2211.1106	47.3	102 - 121	1	---	R.QDADIWAVHTSPLMSSAKVR.V

No match to: 841.1960, 841.1960, 842.5637, 855.0995, 871.0715, 882.6186, 882.6186, 893.0596, 909.0323, 915.1290, 915.1290, 927.5462, 947.0018, 1051.7703, 1060.1125, 1060.1126, 1083.1046, 1301.8031, 1301.8031, 1325.8025, 1339.7694, 1674.8976, 1874.0144, 2005.0609, 2124.1565, 2124.1565, 2151.1111, 2151.1111, 2753.4365, 2753.4365

50. gi|486208296 **Mass:** 4883 **Score:** 42 **Expect:** 2e+003 **Matches:** 4
hypothetical protein [Escherichia coli]

Observed	Mr(expt)	Mr(calc)	ppm	Start - End	Miss	Ions	Peptide
1325.8025	1324.7952	1324.7714	18.0	5 - 17	0	---	R.NINVITALSVPGK.T
1874.0144	1873.0071	1872.9913	8.42	1 - 17	1	---	-.MNMRNINVITALSVPGK.T + Oxidation (M)
2753.4365	2752.4292	2752.4218	2.70	18 - 42	1	---	K.TVSDDFIHAVLSNCATRIVLPAPEK.F
2753.4365	2752.4292	2752.4218	2.70	18 - 42	1	---	K.TVSDDFIHAVLSNCATRIVLPAPEK.F

No match to: 841.1960, 841.1960, 842.5637, 855.0995, 871.0715, 882.6186, 882.6186, 893.0596, 909.0323, 915.1290, 915.1290, 927.5462, 947.0018, 1051.7703, 1060.1125, 1060.1126, 1083.1046, 1169.6356, 1169.6356, 1301.8031, 1301.8031, 1313.8103, 1313.8103, 1339.7694, 1674.8976, 2005.0609, 2112.1482, 2112.1482, 2124.1565, 2124.1565, 2151.1111, 2151.1111, 2212.2224

Search Parameters

Type of search : Sequence Query
Enzyme : Trypsin
Fixed modifications : Carbamidomethyl (C)
Variable modifications : Oxidation (M)
Mass values : Monoisotopic
Protein Mass : Unrestricted
Peptide Mass Tolerance : ± 100 ppm
Fragment Mass Tolerance: ± 0.3 Da
Max Missed Cleavages : 1
Instrument type : MALDI-TOF-TOF
Query1 (841.1960,1+) : Locus:1..5.0.4
Query2 (841.1960,1+) : <no title>
Query3 (842.5637,1+) : <no title>
Query4 (855.0995,1+) : <no title>
Query5 (871.0715,1+) : <no title>
Query6 (882.6186,1+) : Locus:1..5.0.7
Query7 (882.6186,1+) : <no title>
Query8 (893.0596,1+) : <no title>
Query9 (909.0323,1+) : <no title>
Query10 (915.1290,1+) : Locus:1..5.0.12
Query11 (915.1290,1+) : <no title>
Query12 (927.5462,1+) : <no title>
Query13 (947.0018,1+) : <no title>
Query14 (1051.7703,1+) : <no title>
Query15 (1060.1125,1+) : <no title>
Query16 (1060.1126,1+) : Locus:1..5.0.6
Query17 (1083.1046,1+) : <no title>
Query18 (1169.6356,1+) : <no title>
Query19 (1169.6356,1+) : Locus:1..5.0.5
Query20 (1301.8031,1+) : <no title>
Query21 (1301.8031,1+) : Locus:1..5.0.1
Query22 (1313.8103,1+) : <no title>
Query23 (1313.8103,1+) : Locus:1..5.0.9
Query24 (1325.8025,1+) : <no title>

PDF-XChange
Click to buy NOW!
www.docu-track.com
PDF-XChange
Click to buy NOW!
www.docu-track.com

[illegible]ry25 (1339.7694,1+) : <no title>
[illegible]uery26 (1674.8976,1+) : <no title>
Query27 (1874.0144,1+) : <no title>
Query28 (2005.0609,1+) : <no title>
Query29 (2112.1482,1+) : Locus:1..5.0.2
Query30 (2112.1482,1+) : <no title>
Query31 (2124.1565,1+) : Locus:1..5.0.10
Query32 (2124.1565,1+) : <no title>
Query33 (2151.1111,1+) : Locus:1..5.0.3
Query34 (2151.1111,1+) : <no title>
Query35 (2212.2224,1+) : <no title>
Query36 (2753.4365,1+) : <no title>
Query37 (2753.4365,1+) : Locus:1..5.0.8

Mascot: http://www.matrixscience.com/
