# Supplementary material for: Alpha-2-Macroglobulin as a New Promising Biomarker Improving the Diagnostic Sensitivity of Bovine Paratuberculosis
Source: Front Vet Sci. 2021 Mar 5;8:637716. doi: 10.3389/fvets.2021.637716 (PMC7973028; doi:10.3389/fvets.2021.637716)
Supplement: Supplementary Data 1 — Mascot search results and raw data files for LC-MS/MS analysis for the identification of specific blood proteins in cattle with Johne's disease. [file Data_Sheet_1.zip › mascot/11 hIgG1 heavy chain constant region_2.pdf]

PDF-XChange Click to buy NOW! www.docu-track.com

PDF-XChange Click to buy NOW! www.docu-track.com

MATRIX SCIENCE

Mascot Search Results

Protein View

Match to: **gi|7547266** Score: **151** Expect: **2.7e-008**
IgG1 heavy chain constant region [Bos taurus]

Nominal mass (M_r): **36510**; Calculated pI value: **6.09**
NCBI BLAST search of gi|7547266 against nr
Unformatted sequence string for pasting into other applications

Taxonomy: Bos taurus

Fixed modifications: Carbamidomethyl (C)
Variable modifications: Oxidation (M)
Cleavage by Trypsin: cuts C-term side of KR unless next residue is P
Sequence Coverage: **24%**

Matched peptides shown in **Bold Red**

1 ASTTAPKVYP LSSCCGDKSS STVTLGCLVS SYMPEPVTVT WNSGALKSGV
51 HTFPAVLQSS GLYSLSSMVT VPGSTSGTQT FTCNVAHPAS STKVDKAVDP
101 RCKTTCDCCP PPELPGGPSV FIFPPKPKDT LTISGTPEVT CVVVDVGHDD
151 PEVK**FSWFVD DVEVNTATTK PR**EEQFNSTY RVVSALR**IQH QDWTGGK**EFK
201 CK**VHNEGLPA PIVR**TISRTK GPAREPQVYV LAPPQEELSK **STVSLTCMVT**
251 **SFYPDYIAVE WQR**NGQPESE DK**YGTTPPQL DADGSYFLYS R**LRVDRNSWQ
301 EGDTYTCVVM HEALHNHYTQ KSTSKSAGK

Show predicted peptides also

Sort Peptides By ◉ Residue Number ○ Increasing Mass ○ Decreasing Mass

Start - End	Observed	Mr(expt)	Mr(calc)	ppm	Miss	Sequence
155 - 172	2112.1482	2111.1409	2111.0324	51	0	K.FSWFVDDVEVNTATTKPR.E (Ions score 46)
155 - 172	2112.1482	2111.1409	2111.0324	51	0	K.FSWFVDDVEVNTATTKPR.E (No match)
188 - 197	1169.6356	1168.6283	1168.5625	56	0	R.IQHQDWTGGK.E (No match)
188 - 197	1169.6356	1168.6283	1168.5625	56	0	R.IQHQDWTGGK.E (No match)
203 - 214	1301.8031	1300.7958	1300.7252	54	0	K.VHNEGLPAPIVR.T (No match)
203 - 214	1301.8031	1300.7958	1300.7252	54	0	K.VHNEGLPAPIVR.T (Ions score 51)
241 - 263	2753.4365	2752.4292	2752.2877	51	0	K.STVSLTCMVTSFYPDYIAVEWQR.N (No match)
241 - 263	2753.4365	2752.4292	2752.2877	51	0	K.STVSLTCMVTSFYPDYIAVEWQR.N (No match)
273 - 291	2151.1111	2150.1038	2149.9957	50	0	K.YGTTPPQLDADGSYFLYSR.L (Ions score 13)
273 - 291	2151.1111	2150.1038	2149.9957	50	0	K.YGTTPPQLDADGSYFLYSR.L (No match)

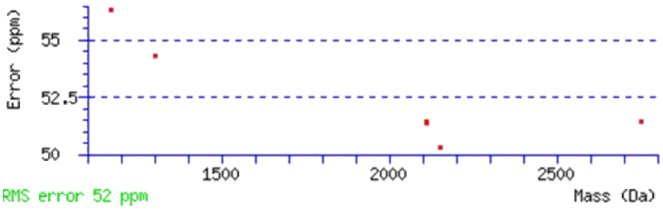


```
LOCUS       AAB37381                 329 aa            linear   MAM 26-JUL-2016
DEFINITION  IgG1 heavy chain constant region, partial [Bos taurus].
ACCESSION   AAB37381
VERSION     AAB37381.2
DBSOURCE    accession S82409.1
KEYWORDS    .
SOURCE      Bos taurus (cattle)
  ORGANISM  Bos taurus
            Eukaryota; Metazoa; Chordata; Craniata; Vertebrata; Euteleostomi;
            Mammalia; Eutheria; Laurasiatheria; Cetartiodactyla; Ruminantia;
            Pecora; Bovidae; Bovinae; Bos.
REFERENCE   1  (residues 1 to 329)
  AUTHORS   Kacskovics,I. and Butler,J.E.
  TITLE     The heterogeneity of bovine IgG2--VIII. The complete cDNA sequence
            of bovine IgG2a (A2) and an IgG1
  JOURNAL   Mol. Immunol. 33 (2), 189-195 (1996)
   PUBMED   8649440
  REMARK    GenBank staff at the National Library of Medicine created this
            entry [NCBI gibbsq 178340] from the original journal article.
COMMENT     On Apr 13, 2000 this sequence version replaced AAB37381.1.
            Method: conceptual translation supplied by author.
FEATURES             Location/Qualifiers
     source          1..329
                     /organism="Bos taurus"
                     /strain="breed Holstein-Friesian"
                     /db_xref="taxon:9913"
```

PDF-XChange Click to buy NOW! www.docu-track.com

```
                /cell_type="peripheral blood leukocytes"
                /note="A2/A2 allotype"
Protein         <1..329
                /product="IgG1 heavy chain constant region"
Region          5..96
                /region_name="IgC_CH1"
                /note="CH1 domain (first constant Ig domain of the heavy
                chain) in immunoglobulin; cd04985"
                /db_xref="CDD:143186"
Site            order(9..10,12,30,53,55..56,65..66,68)
                /site_type="other"
                /note="heterodimer interface [polypeptide binding]"
                /db_xref="CDD:143186"
Site            61
                /site_type="other"
                /note="IgV domain interface [polypeptide binding]"
                /db_xref="CDD:143186"
Region          120..217
                /region_name="Ig"
                /note="Immunoglobulin domain; cl11960"
                /db_xref="CDD:299845"
Region          226..322
                /region_name="Ig"
                /note="Immunoglobulin domain; cl11960"
                /db_xref="CDD:299845"
CDS             1..329
                /gene="IgC-gamma"
                /coded_by="S82409.1:<1..990"
                /note="conceptual translation presented here differs from
                translation in publication"
```

Mascot: http://www.matrixscience.com/
