# Supplementary material for: Alpha-2-Macroglobulin as a New Promising Biomarker Improving the Diagnostic Sensitivity of Bovine Paratuberculosis
Source: Front Vet Sci. 2021 Mar 5;8:637716. doi: 10.3389/fvets.2021.637716 (PMC7973028; doi:10.3389/fvets.2021.637716)
Supplement: Supplementary Data 1 — Mascot search results and raw data files for LC-MS/MS analysis for the identification of specific blood proteins in cattle with Johne's disease. [file Data_Sheet_1.zip › mascot/12 Inter-alpha-trypsin inhibitor heavy chain_1.pdf]

PDF-XChange Click to buy NOW! www.docu-track.com

PDF-XChange Click to buy NOW! www.docu-track.com

MATRIX SCIENCE Mascot Search Results

```
User          :
Email         :
Search title  : BAEDW\2020_3_5\2020_3_5\MSMS 14\N12
Database      : Uniprot_bovine bovine_20200316 (134392 sequences; 76244912 residues)
Timestamp     : 31 Mar 2020 at 01:08:42 GMT
Warning       : A Peptide summary report will usually give a much clearer picture of MS/MS search results.
Top Score     : 74 for F1MMD7, Inter-alpha-trypsin inhibitor heavy chain H4 OS=Bos taurus OX=9913 GN=ITIH4 PE=4 SV=3
```

Mascot Score Histogram

Protein score is -10*Log(P), where P is the probability that the observed match is a random event.
Protein scores greater than 64 are significant (p<0.05).
Protein scores are derived from ions scores as a non-probabilistic basis for ranking protein hits.

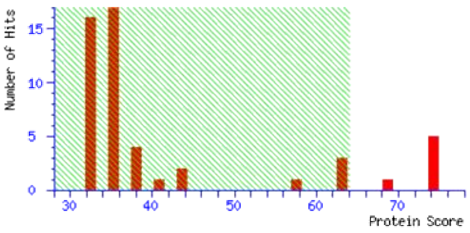

Protein Summary Report

Format As | Protein Summary (deprecated) | Help

Significance threshold p< 0.05 | Max. number of hits 30

Preferred taxonomy All entries

Re-Search All | Search Unmatched

Index

	Accession	Mass	Score	Description
1.	F1MMD7	101620	74	Inter-alpha-trypsin inhibitor heavy chain H4 OS=Bos taurus OX=9913 GN=ITIH4 PE=4 SV=3
2.	A0A4W2C1E9	101620	74	Inter-alpha-trypsin inhibitor heavy chain 4 OS=Bos indicus x Bos taurus OX=30522 GN=ITIH4 PE=4 SV=1
3.	Q5EA67	101617	74	Inter-alpha (Globulin) inhibitor H4 (Plasma Kallikrein-sensitive glycoprotein) OS=Bos taurus OX=9913 GN=
4.	Q3T052	101620	74	Inter-alpha-trypsin inhibitor heavy chain H4 OS=Bos taurus OX=9913 GN=ITIH4 PE=1 SV=1
5.	A0A3Q1MGQ6	103466	74	Inter-alpha-trypsin inhibitor heavy chain H4 OS=Bos taurus OX=9913 GN=ITIH4 PE=4 SV=1
6.	A0A4W2HBJ7	101584	68	Inter-alpha-trypsin inhibitor heavy chain 4 OS=Bos indicus x Bos taurus OX=30522 GN=ITIH4 PE=4 SV=1
7.	A0A3Q1MA31	99755	64	Inter-alpha-trypsin inhibitor heavy chain H4 OS=Bos taurus OX=9913 GN=ITIH4 PE=4 SV=1
8.	A0A4W2DPE8	99755	64	Inter-alpha-trypsin inhibitor heavy chain 4 OS=Bos indicus x Bos taurus OX=30522 GN=ITIH4 PE=4 SV=1
9.	A0A3Q1LZ09	100847	63	Inter-alpha-trypsin inhibitor heavy chain H4 OS=Bos taurus OX=9913 GN=ITIH4 PE=4 SV=1
10.	A0A4W2F8Z8	99718	59	Inter-alpha-trypsin inhibitor heavy chain 4 OS=Bos indicus x Bos taurus OX=30522 GN=ITIH4 PE=4 SV=1
11.	A0A4W2DTX3	38736	42	Glutathione S-transferase omega 1 OS=Bos indicus x Bos taurus OX=30522 GN=GSTO1 PE=3 SV=1
12.	A0A4W2FV73	38767	42	Glutathione S-transferase omega 1 OS=Bos indicus x Bos taurus OX=30522 GN=GSTO1 PE=3 SV=1
13.	A0A3Q1LRF3	39309	41	Uncharacterized protein OS=Bos taurus OX=9913 PE=4 SV=1
14.	A0A4W2F6I5	532453	39	Plectin OS=Bos indicus x Bos taurus OX=30522 GN=PLEC PE=4 SV=1
15.	A0A4W2FGN9	123016	39	Coiled-coil domain containing 150 OS=Bos indicus x Bos taurus OX=30522 GN=CCDC150 PE=4 SV=1
16.	A0A4W2DYM0	53619	38	NAD-dependent protein lipoamidase sirtuin-4, mitochondrial OS=Bos indicus x Bos taurus OX=30522 GN=SIRT
17.	A0A4W2GWY7	132560	38	Euchromatic histone lysine methyltransferase 1 OS=Bos indicus x Bos taurus OX=30522 GN=EHMT1 PE=4 SV=1
18.	A0A4W2E8Y3	132257	36	Euchromatic histone lysine methyltransferase 1 OS=Bos indicus x Bos taurus OX=30522 GN=EHMT1 PE=4 SV=1
19.	A0A3Q1LPD0	132257	36	Euchromatic histone lysine methyltransferase 1 OS=Bos taurus OX=9913 GN=EHMT1 PE=4 SV=1
20.	A0A4W2C1C0	67085	36	Heparanase 2 (inactive) OS=Bos indicus x Bos taurus OX=30522 GN=HPSE2 PE=4 SV=1
21.	O46770	19686	35	MHC class I heavy chain (Fragment) OS=Bos taurus OX=9913 PE=2 SV=1
22.	U5TGS8	86845	34	CPXV068 protein OS=Cowpox virus OX=10243 GN=CPXV068 PE=4 SV=1
23.	A0A4W2CHB4	38688	34	Glutathione S-transferase omega 1 OS=Bos indicus x Bos taurus OX=30522 GN=GSTO1 PE=3 SV=1
24.	A0A4W2FWN4	38719	34	Glutathione S-transferase omega 1 OS=Bos indicus x Bos taurus OX=30522 GN=GSTO1 PE=3 SV=1
25.	A0A0K2YTL5	9022	34	Uncharacterized protein OS=Cowpox virus OX=10243 GN=gCPXV0248 PE=4 SV=1
26.	O46724	19602	34	MHC class I heavy chain (Fragment) OS=Bos taurus OX=9913 PE=2 SV=1
27.	O46765	19602	34	MHC class I heavy chain (Fragment) OS=Bos taurus OX=9913 PE=2 SV=1
28.	A0A4W2BML3	25099	34	HESX homeobox 1 OS=Bos indicus x Bos taurus OX=30522 GN=HESX1 PE=4 SV=1
29.	A0A4W2E8V9	138235	34	Euchromatic histone lysine methyltransferase 1 OS=Bos indicus x Bos taurus OX=30522 GN=EHMT1 PE=4 SV=1
30.	A0A3Q1MVR5	138235	34	Euchromatic histone lysine methyltransferase 1 OS=Bos taurus OX=9913 GN=EHMT1 PE=4 SV=1

Results List

1. F1MMD7 **Mass:** 101620 **Score:** 74 **Expect:** 0.005 **Matches:** 14

Inter-alpha-trypsin inhibitor heavy chain H4 OS=Bos taurus OX=9913 GN=ITIH4 PE=4 SV=3

Observed	Mr(expt)	Mr(calc)	ppm	Start	End	Miss	Ions	Peptide
835.4214	834.4141	834.4422	-33.63	619	625	0	---	R.VYLGPMR.F
853.4803	852.4730	852.3946	92.0	283	290	1	---	K.SGSMMGRK.I
853.4804	852.4731	852.3946	92.1	283	290	1	---	K.SGSMMGRK.I
972.5108	971.5035	971.5400	-37.52	845	852	0	---	R.LLLQNTDR.F
1031.5133	1030.5060	1030.5229	-16.41	430	439	0	---	K.MALENGGLAR.R
1151.6821	1150.6748	1150.5917	72.2	616	625	1	---	R.GSRVYLGPMR.F + Oxidation (M)
1211.5695	1210.5622	1210.6135	-42.34	539	547	0	---	K.YIFHSFIER.L
1349.6478	1348.6405	1348.6987	-43.13	230	241	0	---	K.QDTVLDGSFIVR.Y
1368.6882	1367.6809	1367.7409	-43.82	500	511	0	---	R.EQSPDVLLAQIR.G
1402.7100	1401.7027	1401.7657	-44.89	829	841	0	---	R.VTIGLLFWDGPGK.G
1567.6902	1566.6829	1566.8116	-82.12	800	812	1	---	K.WKETLYSVMPGLK.V + Oxidation (M)
1581.7833	1580.7760	1580.8450	-43.62	141	153	0	---	K.VTFELVYEELLAR.H
1581.7833	1580.7761	1580.8450	-43.60	141	153	0	35	K.VTFELVYEELLAR.H
1637.8650	1636.8577	1636.9260	-41.73	498	511	1	---	K.LREQSPDVLLAQIR.G

No match to: 842.4716, 848.4913, 850.5007, 864.5184, 868.5114, 868.5114, 870.5096, 882.5266, 882.5266, 951.5828, 953.4929, 982.5417, 996.5513, 1007.6395, 1017.6248, 1017.6248, 1019.6375, 1033.6567, 1033.6567, 1036.5132, 1045.5249, 1051.6622, 1051.6622, 1131.6577, 1133.6731, 1136.6519, 1147.6853, 1165.6888, 1165.6888, 1179.5533, 1194.5757, 1234.7560, 1236.6587,

PDF-XChange Click to buy NOW! www.docu-track.com

PDF-XChange Click to buy NOW! www.docu-track.com

1277.6544, 1279.7057, 1300.7963, 1302.8082, 1316.8273, 1334.8298, 1334.8298, 1383.6498, 1421.6836, 1434.8480, 1448.8584, 1448.8584, 1475.6868, 1479.7338, 1617.9998, 1617.9998, 1707.7130, 1732.0293, 1732.0293, 1734.8334, 1838.8518, 1900.9630, 1901.1735, 1993.8977, 2015.2006, 2184.3567, 2211.0339, 2215.0857, 2283.0969, 2872.3440

2. A0A4W2C1E9 **Mass:** 101620 **Score:** 74 **Expect:** 0.005 **Matches:** 14

Inter-alpha-trypsin inhibitor heavy chain 4 OS=Bos indicus x Bos taurus OX=30522 GN=ITIH4 PE=4 SV=1

Observed	Mr(expt)	Mr(calc)	ppm	Start	End	Miss	Ions	Peptide
835.4214	834.4141	834.4422	-33.63	619	625	0	---	R.VYLGPMR.F
853.4803	852.4730	852.3946	92.0	283	290	1	---	K.SGSMMGRK.I
853.4804	852.4731	852.3946	92.1	283	290	1	---	K.SGSMMGRK.I
972.5108	971.5035	971.5400	-37.52	845	852	0	---	R.LLLQNTDR.F
1031.5133	1030.5060	1030.5229	-16.41	430	439	0	---	K.MALENGGLAR.R
1151.6821	1150.6748	1150.5917	72.2	616	625	1	---	R.GSRVYLGPMR.F + Oxidation (M)
1211.5695	1210.5622	1210.6135	-42.34	539	547	0	---	K.YIFHSFIER.L
1349.6478	1348.6405	1348.6987	-43.13	230	241	0	---	K.QDTVLDGSFIVR.Y
1368.6882	1367.6809	1367.7409	-43.82	500	511	0	---	R.EQSPDVLLAQIR.G
1402.7100	1401.7027	1401.7657	-44.89	829	841	0	---	R.VTIGLLFWDGPGK.G
1567.6902	1566.6829	1566.8116	-82.12	800	812	1	---	K.WKETLYSVMPGLK.V + Oxidation (M)
1581.7833	1580.7760	1580.8450	-43.62	141	153	0	---	K.VTFELVYEELLAR.H
1581.7833	1580.7761	1580.8450	-43.60	141	153	0	35	K.VTFELVYEELLAR.H
1637.8650	1636.8577	1636.9260	-41.73	498	511	1	---	K.LREQSPDVLLAQIR.G

No match to: 842.4716, 848.4913, 850.5007, 864.5184, 868.5114, 868.5114, 870.5096, 882.5266, 882.5266, 951.5828, 953.4929, 982.5417, 996.5513, 1007.6395, 1017.6248, 1017.6248, 1019.6375, 1033.6567, 1033.6567, 1036.5132, 1045.5249, 1051.6622, 1051.6622, 1131.6577, 1133.6731, 1136.6519, 1147.6853, 1165.6888, 1165.6888, 1179.5533, 1194.5757, 1234.7560, 1236.6587, 1277.6544, 1279.7057, 1300.7963, 1302.8082, 1316.8273, 1334.8298, 1334.8298, 1383.6498, 1421.6836, 1434.8480, 1448.8584, 1448.8584, 1475.6868, 1479.7338, 1617.9998, 1617.9998, 1707.7130, 1732.0293, 1732.0293, 1734.8334, 1838.8518, 1900.9630, 1901.1735, 1993.8977, 2015.2006, 2184.3567, 2211.0339, 2215.0857, 2283.0969, 2872.3440

3. Q5EA67 **Mass:** 101617 **Score:** 74 **Expect:** 0.005 **Matches:** 14

Inter-alpha (Globulin) inhibitor H4 (Plasma Kallikrein-sensitive glycoprotein) OS=Bos taurus OX=9913 GN=ITIH4 PE=2 SV=1

Observed	Mr(expt)	Mr(calc)	ppm	Start	End	Miss	Ions	Peptide
835.4214	834.4141	834.4422	-33.63	619	625	0	---	R.VYLGPMR.F
853.4803	852.4730	852.3946	92.0	283	290	1	---	K.SGSMMGRK.I
853.4804	852.4731	852.3946	92.1	283	290	1	---	K.SGSMMGRK.I
972.5108	971.5035	971.5400	-37.52	845	852	0	---	R.LLLQNTDR.F
1031.5133	1030.5060	1030.5229	-16.41	430	439	0	---	K.MALENGGLAR.R
1151.6821	1150.6748	1150.5917	72.2	616	625	1	---	R.GSRVYLGPMR.F + Oxidation (M)
1211.5695	1210.5622	1210.6135	-42.34	539	547	0	---	K.YIFHSFIER.L
1349.6478	1348.6405	1348.6987	-43.13	230	241	0	---	K.QDTVLDGSFIVR.Y
1368.6882	1367.6809	1367.7409	-43.82	500	511	0	---	R.EQSPDVLLAQIR.G
1402.7100	1401.7027	1401.7657	-44.89	829	841	0	---	R.VTIGLLFWDGPGK.G
1567.6902	1566.6829	1566.8116	-82.12	800	812	1	---	K.WKETLYSVMPGLK.V + Oxidation (M)
1581.7833	1580.7760	1580.8450	-43.62	141	153	0	---	K.VTFELVYEELLAR.H
1581.7833	1580.7761	1580.8450	-43.60	141	153	0	35	K.VTFELVYEELLAR.H
1637.8650	1636.8577	1636.9260	-41.73	498	511	1	---	K.LREQSPDVLLAQIR.G

No match to: 842.4716, 848.4913, 850.5007, 864.5184, 868.5114, 868.5114, 870.5096, 882.5266, 882.5266, 951.5828, 953.4929, 982.5417, 996.5513, 1007.6395, 1017.6248, 1017.6248, 1019.6375, 1033.6567, 1033.6567, 1036.5132, 1045.5249, 1051.6622, 1051.6622, 1131.6577, 1133.6731, 1136.6519, 1147.6853, 1165.6888, 1165.6888, 1179.5533, 1194.5757, 1234.7560, 1236.6587, 1277.6544, 1279.7057, 1300.7963, 1302.8082, 1316.8273, 1334.8298, 1334.8298, 1383.6498, 1421.6836, 1434.8480, 1448.8584, 1448.8584, 1475.6868, 1479.7338, 1617.9998, 1617.9998, 1707.7130, 1732.0293, 1732.0293, 1734.8334, 1838.8518, 1900.9630, 1901.1735, 1993.8977, 2015.2006, 2184.3567, 2211.0339, 2215.0857, 2283.0969, 2872.3440

4. Q3T052 **Mass:** 101620 **Score:** 74 **Expect:** 0.005 **Matches:** 14

Inter-alpha-trypsin inhibitor heavy chain H4 OS=Bos taurus OX=9913 GN=ITIH4 PE=1 SV=1

Observed	Mr(expt)	Mr(calc)	ppm	Start	End	Miss	Ions	Peptide
835.4214	834.4141	834.4422	-33.63	619	625	0	---	R.VYLGPMR.F
853.4803	852.4730	852.3946	92.0	283	290	1	---	K.SGSMMGRK.I
853.4804	852.4731	852.3946	92.1	283	290	1	---	K.SGSMMGRK.I
972.5108	971.5035	971.5400	-37.52	845	852	0	---	R.LLLQNTDR.F
1031.5133	1030.5060	1030.5229	-16.41	430	439	0	---	K.MALENGGLAR.R
1151.6821	1150.6748	1150.5917	72.2	616	625	1	---	R.GSRVYLGPMR.F + Oxidation (M)
1211.5695	1210.5622	1210.6135	-42.34	539	547	0	---	K.YIFHSFIER.L
1349.6478	1348.6405	1348.6987	-43.13	230	241	0	---	K.QDTVIDGSFIVR.Y
1368.6882	1367.6809	1367.7409	-43.82	500	511	0	---	R.EQSPDVLLAQIR.G
1402.7100	1401.7027	1401.7657	-44.89	829	841	0	---	R.VTIGLLFWDGPGK.G
1567.6902	1566.6829	1566.8116	-82.12	800	812	1	---	K.WKETLYSVMPGLK.V + Oxidation (M)
1581.7833	1580.7760	1580.8450	-43.62	141	153	0	---	K.VTFELVYEELLAR.H
1581.7833	1580.7761	1580.8450	-43.60	141	153	0	35	K.VTFELVYEELLAR.H
1637.8650	1636.8577	1636.9260	-41.73	498	511	1	---	K.LREQSPDVLLAQIR.G

No match to: 842.4716, 848.4913, 850.5007, 864.5184, 868.5114, 868.5114, 870.5096, 882.5266, 882.5266, 951.5828, 953.4929, 982.5417, 996.5513, 1007.6395, 1017.6248, 1017.6248, 1019.6375, 1033.6567, 1033.6567, 1036.5132, 1045.5249, 1051.6622, 1051.6622, 1131.6577, 1133.6731, 1136.6519, 1147.6853, 1165.6888, 1165.6888, 1179.5533, 1194.5757, 1234.7560, 1236.6587, 1277.6544, 1279.7057, 1300.7963, 1302.8082, 1316.8273, 1334.8298, 1334.8298, 1383.6498, 1421.6836, 1434.8480, 1448.8584, 1448.8584, 1475.6868, 1479.7338, 1617.9998, 1617.9998, 1707.7130, 1732.0293, 1732.0293, 1734.8334, 1838.8518, 1900.9630, 1901.1735, 1993.8977, 2015.2006, 2184.3567, 2211.0339, 2215.0857, 2283.0969, 2872.3440

5. A0A3Q1MGQ6 **Mass:** 103466 **Score:** 74 **Expect:** 0.006 **Matches:** 14

Inter-alpha-trypsin inhibitor heavy chain H4 OS=Bos taurus OX=9913 GN=ITIH4 PE=4 SV=1

Observed	Mr(expt)	Mr(calc)	ppm	Start	End	Miss	Ions	Peptide
835.4214	834.4141	834.4422	-33.63	619	625	0	---	R.VYLGPMR.F
853.4803	852.4730	852.3946	92.0	283	290	1	---	K.SGSMMGRK.I
853.4804	852.4731	852.3946	92.1	283	290	1	---	K.SGSMMGRK.I
972.5108	971.5035	971.5400	-37.52	845	852	0	---	R.LLLQNTDR.F
1031.5133	1030.5060	1030.5229	-16.41	430	439	0	---	K.MALENGGLAR.R
1151.6821	1150.6748	1150.5917	72.2	616	625	1	---	R.GSRVYLGPMR.F + Oxidation (M)
1211.5695	1210.5622	1210.6135	-42.34	539	547	0	---	K.YIFHSFIER.L
1349.6478	1348.6405	1348.6987	-43.13	230	241	0	---	K.QDTVLDGSFIVR.Y
1368.6882	1367.6809	1367.7409	-43.82	500	511	0	---	R.EQSPDVLLAQIR.G
1402.7100	1401.7027	1401.7657	-44.89	829	841	0	---	R.VTIGLLFWDGPGK.G
1567.6902	1566.6829	1566.8116	-82.12	800	812	1	---	K.WKETLYSVMPGLK.V + Oxidation (M)
1581.7833	1580.7760	1580.8450	-43.62	141	153	0	---	K.VTFELVYEELLAR.H
1581.7833	1580.7761	1580.8450	-43.60	141	153	0	35	K.VTFELVYEELLAR.H
1637.8650	1636.8577	1636.9260	-41.73	498	511	1	---	K.LREQSPDVLLAQIR.G

No match to: 842.4716, 848.4913, 850.5007, 864.5184, 868.5114, 868.5114, 870.5096, 882.5266, 882.5266, 951.5828, 953.4929, 982.5417, 996.5513, 1007.6395, 1017.6248, 1017.6248, 1019.6375, 1033.6567, 1033.6567, 1036.5132, 1045.5249, 1051.6622,

PDF-XChange Click to buy NOW! www.docu-track.com

1051.6622, 1131.6577, 1133.6731, 1136.6519, 1147.6853, 1165.6888, 1165.6888, 1179.5533, 1194.5757, 1234.7560, 1236.6587, 1277.6544, 1279.7057, 1300.7963, 1302.8082, 1316.8273, 1334.8298, 1334.8298, 1383.6498, 1421.6836, 1434.8480, 1448.8584, 1448.8584, 1475.6868, 1479.7338, 1617.9998, 1617.9998, 1707.7130, 1732.0293, 1732.0293, 1734.8334, 1838.8518, 1900.9630, 1901.1735, 1993.8977, 2015.2006, 2184.3567, 2211.0339, 2215.0857, 2283.0969, 2872.3440

6. A0A4W2HBJ7 **Mass:** 101584 **Score:** 68 **Expect:** 0.019 **Matches:** 13

Inter-alpha-trypsin inhibitor heavy chain 4 OS=Bos indicus x Bos taurus OX=30522 GN=ITIH4 PE=4 SV=1

Observed	Mr(expt)	Mr(calc)	ppm	Start	End	Miss	Ions	Peptide
835.4214	834.4141	834.4422	-33.63	619	625	0	---	R.VYLGPMR.F
853.4803	852.4730	852.3946	92.0	283	290	1	---	K.SGSMMGRK.I
853.4804	852.4731	852.3946	92.1	283	290	1	---	K.SGSMMGRK.I
972.5108	971.5035	971.5400	-37.52	845	852	0	---	R.LLLQNTDR.F
1031.5133	1030.5060	1030.5229	-16.41	430	439	0	---	K.MALENGGLAR.R
1151.6821	1150.6748	1150.5917	72.2	616	625	1	---	R.GSRVYLGPMR.F + Oxidation (M)
1349.6478	1348.6405	1348.6987	-43.13	230	241	0	---	K.QDTVLDGSFIVR.Y
1368.6882	1367.6809	1367.7409	-43.82	500	511	0	---	R.EQSPDVLLAQIR.G
1402.7100	1401.7027	1401.7657	-44.89	829	841	0	---	R.VTIGLLFWDGPGK.G
1567.6902	1566.6829	1566.8116	-82.12	800	812	1	---	K.WKETLYSVMPGLK.V + Oxidation (M)
1581.7833	1580.7760	1580.8450	-43.62	141	153	0	---	K.VTFELVYEELLAR.H
1581.7833	1580.7761	1580.8450	-43.60	141	153	0	35	K.VTFELVYEELLAR.H
1637.8650	1636.8577	1636.9260	-41.73	498	511	1	---	K.LREQSPDVLLAQIR.G

No match to: 842.4716, 848.4913, 850.5007, 864.5184, 868.5114, 868.5114, 870.5096, 882.5266, 882.5266, 951.5828, 953.4929, 982.5417, 996.5513, 1007.6395, 1017.6248, 1017.6248, 1019.6375, 1033.6567, 1033.6567, 1036.5132, 1045.5249, 1051.6622, 1051.6622, 1131.6577, 1133.6731, 1136.6519, 1147.6853, 1165.6888, 1165.6888, 1179.5533, 1194.5757, 1211.5695, 1234.7560, 1236.6587, 1277.6544, 1279.7057, 1300.7963, 1302.8082, 1316.8273, 1334.8298, 1334.8298, 1383.6498, 1421.6836, 1434.8480, 1448.8584, 1448.8584, 1475.6868, 1479.7338, 1617.9998, 1617.9998, 1707.7130, 1732.0293, 1732.0293, 1734.8334, 1838.8518, 1900.9630, 1901.1735, 1993.8977, 2015.2006, 2184.3567, 2211.0339, 2215.0857, 2283.0969, 2872.3440

7. A0A3Q1MA31 **Mass:** 99755 **Score:** 64 **Expect:** 0.057 **Matches:** 12

Inter-alpha-trypsin inhibitor heavy chain H4 OS=Bos taurus OX=9913 GN=ITIH4 PE=4 SV=1

Observed	Mr(expt)	Mr(calc)	ppm	Start	End	Miss	Ions	Peptide
853.4803	852.4730	852.3946	92.0	283	290	1	---	K.SGSMMGRK.I
853.4804	852.4731	852.3946	92.1	283	290	1	---	K.SGSMMGRK.I
972.5108	971.5035	971.5400	-37.52	828	835	0	---	R.LLLQNTDR.F
1031.5133	1030.5060	1030.5229	-16.41	430	439	0	---	K.MALENGGLAR.R
1211.5695	1210.5622	1210.6135	-42.34	539	547	0	---	K.YIFHSFIER.L
1349.6478	1348.6405	1348.6987	-43.13	230	241	0	---	K.QDTVLDGSFIVR.Y
1368.6882	1367.6809	1367.7409	-43.82	500	511	0	---	R.EQSPDVLLAQIR.G
1402.7100	1401.7027	1401.7657	-44.89	812	824	0	---	R.VTIGLLFWDGPGK.G
1567.6902	1566.6829	1566.8116	-82.12	783	795	1	---	K.WKETLYSVMPGLK.V + Oxidation (M)
1581.7833	1580.7760	1580.8450	-43.62	141	153	0	---	K.VTFELVYEELLAR.H
1581.7833	1580.7761	1580.8450	-43.60	141	153	0	35	K.VTFELVYEELLAR.H
1637.8650	1636.8577	1636.9260	-41.73	498	511	1	---	K.LREQSPDVLLAQIR.G

No match to: 835.4214, 842.4716, 848.4913, 850.5007, 864.5184, 868.5114, 868.5114, 870.5096, 882.5266, 882.5266, 951.5828, 953.4929, 982.5417, 996.5513, 1007.6395, 1017.6248, 1017.6248, 1019.6375, 1033.6567, 1033.6567, 1036.5132, 1045.5249, 1051.6622, 1051.6622, 1131.6577, 1133.6731, 1136.6519, 1147.6853, 1151.6821, 1165.6888, 1179.5533, 1194.5757, 1234.7560, 1236.6587, 1277.6544, 1279.7057, 1300.7963, 1302.8082, 1316.8273, 1334.8298, 1334.8298, 1383.6498, 1421.6836, 1434.8480, 1448.8584, 1448.8584, 1475.6868, 1479.7338, 1617.9998, 1617.9998, 1707.7130, 1732.0293, 1732.0293, 1734.8334, 1838.8518, 1900.9630, 1901.1735, 1993.8977, 2015.2006, 2184.3567, 2211.0339, 2215.0857, 2283.0969, 2872.3440

8. A0A4W2DPE8 **Mass:** 99755 **Score:** 64 **Expect:** 0.057 **Matches:** 12

Inter-alpha-trypsin inhibitor heavy chain 4 OS=Bos indicus x Bos taurus OX=30522 GN=ITIH4 PE=4 SV=1

Observed	Mr(expt)	Mr(calc)	ppm	Start	End	Miss	Ions	Peptide
853.4803	852.4730	852.3946	92.0	283	290	1	---	K.SGSMMGRK.I
853.4804	852.4731	852.3946	92.1	283	290	1	---	K.SGSMMGRK.I
972.5108	971.5035	971.5400	-37.52	828	835	0	---	R.LLLQNTDR.F
1031.5133	1030.5060	1030.5229	-16.41	430	439	0	---	K.MALENGGLAR.R
1211.5695	1210.5622	1210.6135	-42.34	539	547	0	---	K.YIFHSFIER.L
1349.6478	1348.6405	1348.6987	-43.13	230	241	0	---	K.QDTVLDGSFIVR.Y
1368.6882	1367.6809	1367.7409	-43.82	500	511	0	---	R.EQSPDVLLAQIR.G
1402.7100	1401.7027	1401.7657	-44.89	812	824	0	---	R.VTIGLLFWDGPGK.G
1567.6902	1566.6829	1566.8116	-82.12	783	795	1	---	K.WKETLYSVMPGLK.V + Oxidation (M)
1581.7833	1580.7760	1580.8450	-43.62	141	153	0	---	K.VTFELVYEELLAR.H
1581.7833	1580.7761	1580.8450	-43.60	141	153	0	35	K.VTFELVYEELLAR.H
1637.8650	1636.8577	1636.9260	-41.73	498	511	1	---	K.LREQSPDVLLAQIR.G

No match to: 835.4214, 842.4716, 848.4913, 850.5007, 864.5184, 868.5114, 868.5114, 870.5096, 882.5266, 882.5266, 951.5828, 953.4929, 982.5417, 996.5513, 1007.6395, 1017.6248, 1017.6248, 1019.6375, 1033.6567, 1033.6567, 1036.5132, 1045.5249, 1051.6622, 1051.6622, 1131.6577, 1133.6731, 1136.6519, 1147.6853, 1151.6821, 1165.6888, 1165.6888, 1179.5533, 1194.5757, 1234.7560, 1236.6587, 1277.6544, 1279.7057, 1300.7963, 1302.8082, 1316.8273, 1334.8298, 1334.8298, 1383.6498, 1421.6836, 1434.8480, 1448.8584, 1448.8584, 1475.6868, 1479.7338, 1617.9998, 1617.9998, 1707.7130, 1732.0293, 1732.0293, 1734.8334, 1838.8518, 1900.9630, 1901.1735, 1993.8977, 2015.2006, 2184.3567, 2211.0339, 2215.0857, 2283.0969, 2872.3440

9. A0A3Q1LZ09 **Mass:** 100847 **Score:** 63 **Expect:** 0.061 **Matches:** 12

Inter-alpha-trypsin inhibitor heavy chain H4 OS=Bos taurus OX=9913 GN=ITIH4 PE=4 SV=1

Observed	Mr(expt)	Mr(calc)	ppm	Start	End	Miss	Ions	Peptide
853.4803	852.4730	852.3946	92.0	283	290	1	---	K.SGSMMGRK.I
853.4804	852.4731	852.3946	92.1	283	290	1	---	K.SGSMMGRK.I
972.5108	971.5035	971.5400	-37.52	839	846	0	---	R.LLLQNTDR.F
1031.5133	1030.5060	1030.5229	-16.41	430	439	0	---	K.MALENGGLAR.R
1211.5695	1210.5622	1210.6135	-42.34	539	547	0	---	K.YIFHSFIER.L
1349.6478	1348.6405	1348.6987	-43.13	230	241	0	---	K.QDTVLDGSFIVR.Y
1368.6882	1367.6809	1367.7409	-43.82	500	511	0	---	R.EQSPDVLLAQIR.G
1402.7100	1401.7027	1401.7657	-44.89	823	835	0	---	R.VTIGLLFWDGPGK.G
1567.6902	1566.6829	1566.8116	-82.12	794	806	1	---	K.WKETLYSVMPGLK.V + Oxidation (M)
1581.7833	1580.7760	1580.8450	-43.62	141	153	0	---	K.VTFELVYEELLAR.H
1581.7833	1580.7761	1580.8450	-43.60	141	153	0	35	K.VTFELVYEELLAR.H
1637.8650	1636.8577	1636.9260	-41.73	498	511	1	---	K.LREQSPDVLLAQIR.G

No match to: 835.4214, 842.4716, 848.4913, 850.5007, 864.5184, 868.5114, 868.5114, 870.5096, 882.5266, 882.5266, 951.5828, 953.4929, 982.5417, 996.5513, 1007.6395, 1017.6248, 1017.6248, 1019.6375, 1033.6567, 1033.6567, 1036.5132, 1045.5249, 1051.6622, 1051.6622, 1131.6577, 1133.6731, 1136.6519, 1147.6853, 1151.6821, 1165.6888, 1165.6888, 1179.5533, 1194.5757, 1234.7560, 1236.6587, 1277.6544, 1279.7057, 1300.7963, 1302.8082, 1316.8273, 1334.8298, 1334.8298, 1383.6498, 1421.6836, 1434.8480, 1448.8584, 1448.8584, 1475.6868, 1479.7338, 1617.9998, 1617.9998, 1707.7130, 1732.0293, 1732.0293, 1734.8334, 1838.8518, 1900.9630, 1901.1735, 1993.8977, 2015.2006, 2184.3567, 2211.0339, 2215.0857, 2283.0969, 2872.3440

10. A0A4W2F8Z8 **Mass:** 99718 **Score:** 59 **Expect:** 0.18 **Matches:** 11

PDF-XChange Click to buy NOW! www.docu-track.com

Inter-alpha-trypsin inhibitor heavy chain 4 OS=Bos indicus x Bos taurus OX=30522 GN=ITIH4 PE=4 SV=1

Observed	Mr(expt)	Mr(calc)	ppm	Start	End	Miss	Ions	Peptide
853.4803	852.4730	852.3946	92.0	283	290	1	---	K.SGSMMGRK.I
853.4804	852.4731	852.3946	92.1	283	290	1	---	K.SGSMMGRK.I
972.5108	971.5035	971.5400	-37.52	828	835	0	---	R.LLLQNTDR.F
1031.5133	1030.5060	1030.5229	-16.41	430	439	0	---	K.MALENGGLAR.R
1349.6478	1348.6405	1348.6987	-43.13	230	241	0	---	K.QDTVLDGSFIVR.Y
1368.6882	1367.6809	1367.7409	-43.82	500	511	0	---	R.EQSPDVLLAQIR.G
1402.7100	1401.7027	1401.7657	-44.89	812	824	0	---	R.VTIGLLFWDGPGK.G
1567.6902	1566.6829	1566.8116	-82.12	783	795	1	---	K.WKETLYSVMPGLK.V + Oxidation (M)
1581.7833	1580.7760	1580.8450	-43.62	141	153	0	---	K.VTFELVYEELLAR.H
1581.7833	1580.7761	1580.8450	-43.60	141	153	0	35	K.VTFELVYEELLAR.H
1637.8650	1636.8577	1636.9260	-41.73	498	511	1	---	K.LREQSPDVLLAQIR.G

No match to: 835.4214, 842.4716, 848.4913, 850.5007, 864.5184, 868.5114, 868.5114, 870.5096, 882.5266, 882.5266, 951.5828, 953.4929, 982.5417, 996.5513, 1007.6395, 1017.6248, 1017.6248, 1019.6375, 1033.6567, 1033.6567, 1036.5132, 1045.5249, 1051.6622, 1051.6622, 1131.6577, 1133.6731, 1136.6519, 1147.6853, 1151.6821, 1165.6888, 1165.6888, 1179.5533, 1194.5757, 1211.5695, 1234.7560, 1236.6587, 1277.6544, 1279.7057, 1300.7963, 1302.8082, 1316.8273, 1334.8298, 1334.8298, 1383.6498, 1421.6836, 1434.8480, 1448.8584, 1448.8584, 1475.6868, 1479.7338, 1617.9998, 1617.9998, 1707.7130, 1732.0293, 1732.0293, 1734.8334, 1838.8518, 1900.9630, 1901.1735, 1993.8977, 2015.2006, 2184.3567, 2211.0339, 2215.0857, 2283.0969, 2872.3440

11. A0A4W2DTX3 **Mass:** 38736 **Score:** 42 **Expect:** 7.6 **Matches:** 10

Glutathione S-transferase omega 1 OS=Bos indicus x Bos taurus OX=30522 GN=GSTO1 PE=3 SV=1

Observed	Mr(expt)	Mr(calc)	ppm	Start	End	Miss	Ions	Peptide
850.5007	849.4934	849.4241	81.6	304	310	0	---	K.LWMAAMK.K
870.5096	869.5023	869.5811	-90.54	140	146	1	---	K.RTLLVLR.A
1031.5133	1030.5060	1030.5335	-26.66	205	213	0	---	K.LLPGDPYEK.A
1279.7057	1278.6984	1278.5874	86.8	236	246	1	---	R.TQNKEDCSGLK.E
1300.7963	1299.7890	1299.6969	70.9	68	79	1	---	R.ADGCPSLALLRK.I
1402.7100	1401.7027	1401.7715	-49.07	311	323	1	---	K.KDPTVSSLLTDVK.T
1421.6836	1420.6763	1420.6631	9.28	129	139	1	---	R.VYSMRFCPYAK.R
1732.0293	1731.0220	1730.9679	31.3	111	128	1	---	R.SLGKGSAPPGPVPEGLIR.V
1732.0293	1731.0220	1730.9679	31.3	111	128	1	---	R.SLGKGSAPPGPVPEGLIR.V
1734.8334	1733.8261	1733.9790	-88.16	218	232	1	---	K.MVFESFSKVPPLILK.I

No match to: 835.4214, 842.4716, 848.4913, 853.4803, 853.4804, 864.5184, 868.5114, 868.5114, 882.5266, 882.5266, 951.5828, 953.4929, 972.5108, 982.5417, 996.5513, 1007.6395, 1017.6248, 1017.6248, 1019.6375, 1033.6567, 1033.6567, 1036.5132, 1045.5249, 1051.6622, 1051.6622, 1131.6577, 1133.6731, 1136.6519, 1147.6853, 1151.6821, 1165.6888, 1165.6888, 1179.5533, 1194.5757, 1211.5695, 1234.7560, 1236.6587, 1277.6544, 1302.8082, 1316.8273, 1334.8298, 1334.8298, 1349.6478, 1368.6882, 1383.6498, 1434.8480, 1448.8584, 1448.8584, 1475.6868, 1479.7338, 1567.6902, 1581.7833, 1581.7833, 1617.9998, 1617.9998, 1637.8650, 1707.7130, 1838.8518, 1900.9630, 1901.1735, 1993.8977, 2015.2006, 2184.3567, 2211.0339, 2215.0857, 2283.0969, 2872.3440

12. A0A4W2FV73 **Mass:** 38767 **Score:** 42 **Expect:** 7.6 **Matches:** 10

Glutathione S-transferase omega 1 OS=Bos indicus x Bos taurus OX=30522 GN=GSTO1 PE=3 SV=1

Observed	Mr(expt)	Mr(calc)	ppm	Start	End	Miss	Ions	Peptide
850.5007	849.4934	849.4241	81.6	304	310	0	---	K.LWMAAMK.K
870.5096	869.5023	869.5811	-90.54	140	146	1	---	K.RTLLVLR.A
1031.5133	1030.5060	1030.5335	-26.66	205	213	0	---	K.LLPGDPYEK.A
1279.7057	1278.6984	1278.5874	86.8	236	246	1	---	R.TQNKEDCSGLK.E
1300.7963	1299.7890	1299.6969	70.9	68	79	1	---	R.ADGCPSLALLRK.I
1402.7100	1401.7027	1401.7715	-49.07	311	323	1	---	K.KDPTVSSLLTDVK.T
1421.6836	1420.6763	1420.6631	9.28	129	139	1	---	R.VYSMRFCPYAK.R
1732.0293	1731.0220	1730.9679	31.3	111	128	1	---	R.SLGKGSAPPGPVPEGLIR.V
1732.0293	1731.0220	1730.9679	31.3	111	128	1	---	R.SLGKGSAPPGPVPEGLIR.V
1734.8334	1733.8261	1733.9790	-88.16	218	232	1	---	K.MVFESFSKVPPLILK.I

No match to: 835.4214, 842.4716, 848.4913, 853.4803, 853.4804, 864.5184, 868.5114, 868.5114, 882.5266, 882.5266, 951.5828, 953.4929, 972.5108, 982.5417, 996.5513, 1007.6395, 1017.6248, 1017.6248, 1019.6375, 1033.6567, 1033.6567, 1036.5132, 1045.5249, 1051.6622, 1051.6622, 1131.6577, 1133.6731, 1136.6519, 1147.6853, 1151.6821, 1165.6888, 1165.6888, 1179.5533, 1194.5757, 1211.5695, 1234.7560, 1236.6587, 1277.6544, 1302.8082, 1316.8273, 1334.8298, 1334.8298, 1349.6478, 1368.6882, 1383.6498, 1434.8480, 1448.8584, 1448.8584, 1475.6868, 1479.7338, 1567.6902, 1581.7833, 1581.7833, 1617.9998, 1617.9998, 1637.8650, 1707.7130, 1838.8518, 1900.9630, 1901.1735, 1993.8977, 2015.2006, 2184.3567, 2211.0339, 2215.0857, 2283.0969, 2872.3440

13. A0A3Q1LRF3 **Mass:** 39309 **Score:** 41 **Expect:** 9.7 **Matches:** 5

Uncharacterized protein OS=Bos taurus OX=9913 PE=4 SV=1

Observed	Mr(expt)	Mr(calc)	ppm	Start	End	Miss	Ions	Peptide
1236.6587	1235.6514	1235.6193	26.0	337	346	1	---	K.NVNALACRYR.-
1421.6836	1420.6763	1420.7674	-64.11	106	117	1	---	R.LFSNTLSDQIRK.I
1732.0293	1731.0220	1730.8587	94.3	28	43	0	---	R.NPNNSWNSSSALSLLK.Y
1732.0293	1731.0220	1730.8587	94.3	28	43	0	24	R.NPNNSWNSSSALSLLK.Y
2872.3440	2871.3367	2871.5640	-79.16	293	318	0	---	K.SVQLNGQPLVMVDDGTLPELKPRPLR.A

No match to: 835.4214, 842.4716, 848.4913, 850.5007, 853.4803, 853.4804, 864.5184, 868.5114, 868.5114, 870.5096, 882.5266, 882.5266, 951.5828, 953.4929, 972.5108, 982.5417, 996.5513, 1007.6395, 1017.6248, 1017.6248, 1019.6375, 1031.5133, 1033.6567, 1033.6567, 1036.5132, 1045.5249, 1051.6622, 1051.6622, 1131.6577, 1133.6731, 1136.6519, 1147.6853, 1151.6821, 1165.6888, 1165.6888, 1179.5533, 1194.5757, 1211.5695, 1234.7560, 1277.6544, 1279.7057, 1300.7963, 1302.8082, 1316.8273, 1334.8298, 1334.8298, 1349.6478, 1368.6882, 1383.6498, 1402.7100, 1434.8480, 1448.8584, 1448.8584, 1475.6868, 1479.7338, 1567.6902, 1581.7833, 1581.7833, 1617.9998, 1617.9998, 1637.8650, 1707.7130, 1734.8334, 1838.8518, 1900.9630, 1901.1735, 1993.8977, 2015.2006, 2184.3567, 2211.0339, 2215.0857, 2283.0969

14. A0A4W2F6I5 **Mass:** 532453 **Score:** 39 **Expect:** 15 **Matches:** 35

Plectin OS=Bos indicus x Bos taurus OX=30522 GN=PLEC PE=4 SV=1

Observed	Mr(expt)	Mr(calc)	ppm	Start	End	Miss	Ions	Peptide
835.4214	834.4141	834.4269	-15.32	4492	4499	0	---	K.MSAAQALK.K + Oxidation (M)
850.5007	849.4934	849.4596	39.8	723	729	0	---	R.LDLQYAK.L
864.5184	863.5111	863.4501	70.7	2299	2305	0	---	K.FAEQTLR.Q
953.4929	952.4856	952.4403	47.6	3635	3642	0	---	K.GFFDPNTR.E
972.5108	971.5035	971.5148	-11.61	2205	2213	1	---	R.LRGEAEAAR.R
982.5417	981.5344	981.4953	39.8	3493	3500	0	---	K.VTIYEAMR.R
996.5513	995.5440	995.5261	18.0	924	932	1	---	R.NPAQPTRGR.V
1017.6248	1016.6175	1016.5502	66.2	4587	4596	0	---	R.LLEAAAQSSK.G
1017.6248	1016.6175	1016.5502	66.2	4587	4596	0	---	R.LLEAAAQSSK.G
1031.5133	1030.5060	1030.5043	1.68	2266	2274	1	---	R.KEAEQEAAR.R
1036.5132	1035.5059	1035.4985	7.15	130	139	0	---	R.GPPPTEDPAR.E
1045.5249	1044.5176	1044.5312	-12.99	1565	1573	1	---	R.QVEAAERSR.L
1131.6577	1130.6504	1130.6183	28.5	1976	1985	1	---	K.TEAEIALKEK.E
1133.6731	1132.6658	1132.5910	66.0	1111	1119	1	---	R.CISELKDIR.L

PDF-XChange Click to buy NOW! www.docu-track.com

PDF-XChange Click to buy NOW! www.docu-track.com

1165.6888	1164.6815	1164.5662	99.0	4326 -	4335	0	---	R.QYDIDEAIAK.S
1165.6888	1164.6816	1164.5662	99.0	4326 -	4335	0	---	R.QYDIDEAIAK.S
1179.5533	1178.5460	1178.5237	18.9	2501 -	2510	0	---	R.QLEMSAEAER.L + Oxidation (M)
1194.5757	1193.5684	1193.6842	-96.98	4146 -	4155	1	---	K.LISLFQAMKK.G + Oxidation (M)
1234.7560	1233.7487	1233.6618	70.4	59 -	68	1	---	R.GLVRETFAWR.H
1236.6587	1235.6514	1235.6220	23.8	4117 -	4127	1	---	R.MGIVGPEFKDK.L + Oxidation (M)
1279.7057	1278.6984	1278.7183	-15.56	3550 -	3561	1	---	K.LLSAEKAVTGYK.D
1300.7963	1299.7890	1299.7333	42.9	4468 -	4478	1	---	K.IMVDRINLAQK.A
1316.8273	1315.8200	1315.7282	69.8	4468 -	4478	1	---	K.IMVDRINLAQK.A + Oxidation (M)
1383.6498	1382.6425	1382.7630	-87.11	2161 -	2172	1	---	R.QLQLAQEAAQKR.L
1434.8480	1433.8407	1433.7626	54.5	3643 -	3653	1	---	R.ENLTYRQLLER.C
1448.8584	1447.8511	1447.7089	98.3	2501 -	2512	1	---	R.QLEMSAEAERLR.L + Oxidation (M)
1448.8584	1447.8511	1447.7089	98.3	2501 -	2512	1	---	R.QLEMSAEAERLR.L + Oxidation (M)
1475.6868	1474.6795	1474.7263	-31.74	3290 -	3303	1	---	K.ETSTALTAPRDDAK.T
1637.8650	1636.8577	1636.8672	-5.76	1240 -	1255	0	---	K.EAQAVPAALPELEATK.A
1732.0293	1731.0220	1730.9203	58.8	3991 -	4006	1	---	R.DEASGLFLLPLSDARK.L
1732.0293	1731.0220	1730.8549	96.6	392 -	406	0	---	K.SIITYVSSLYDAMPR.V + Oxidation (M)
1900.9630	1899.9557	1899.8396	61.1	951 -	966	0	---	K.GDECQMLGPAQPFHWK.V
1993.8977	1992.8904	1993.0593	-84.71	3257 -	3275	1	---	R.LLDAQLSTGGIVDPSKSHR.L
2211.0339	2210.0266	2210.1039	-34.96	2245 -	2265	1	---	K.QAAEEQAQAQAQAQAAAEKLR.K
2215.0857	2214.0784	2214.1168	-17.34	149 -	170	0	---	K.EPEEGAPEPPVVPATTPGTLAR.Q

No match to: 842.4716, 848.4913, 853.4803, 853.4804, 868.5114, 868.5114, 870.5096, 882.5266, 882.5266, 951.5828, 1007.6395, 1019.6375, 1033.6567, 1033.6567, 1051.6622, 1051.6622, 1136.6519, 1147.6853, 1151.6821, 1211.5695, 1277.6544, 1302.8082, 1334.8298, 1334.8298, 1349.6478, 1368.6882, 1402.7100, 1421.6836, 1479.7338, 1567.6902, 1581.7833, 1581.7833, 1617.9998, 1617.9998, 1707.7130, 1734.8334, 1838.8518, 1901.1735, 2015.2006, 2184.3567, 2283.0969, 2872.3440

15. A0A4W2FGN9 **Mass:** 123016 **Score:** 39 **Expect:** 17 **Matches:** 17

Coiled-coil domain containing 150 OS=Bos indicus x Bos taurus OX=30522 GN=CCDC150 PE=4 SV=1

Observed	Mr(expt)	Mr(calc)	ppm	Start	End	Miss	Ions	Peptide
848.4913	847.4840	847.4664	20.8	642 -	648	1	---	K.KVGNFQR.Q
864.5184	863.5111	863.4422	79.8	826 -	832	1	---	R.EVTEMKK.A
868.5114	867.5041	867.4450	68.1	766 -	772	0	---	K.LDHVQEK.L
868.5114	867.5041	867.4450	68.1	766 -	772	0	---	K.LDHVQEK.L
1031.5133	1030.5060	1030.5658	-58.04	441 -	449	0	---	K.ELLESTIAR.L
1147.6853	1146.6780	1146.6067	62.2	893 -	901	1	---	R.MKQIETELR.Q
1279.7057	1278.6984	1278.6754	18.0	110 -	119	1	---	K.MNIFRLQTEK.D
1300.7963	1299.7890	1299.7510	29.2	441 -	451	1	---	K.ELLESTIARLR.G
1402.7100	1401.7027	1401.7351	-23.09	295 -	306	1	---	R.DDIISKLVEENK.N
1448.8584	1447.8511	1447.7453	73.1	30 -	41	1	---	R.MRIVEEQTSSLR.D
1448.8584	1447.8511	1447.7453	73.1	30 -	41	1	---	R.MRIVEEQTSSLR.D
1475.6868	1474.6795	1474.6535	17.6	163 -	174	1	---	K.EEEDKAQDEVQR.L
1479.7338	1478.7265	1478.7551	-19.35	985 -	996	1	---	K.CRFDGLQLELTK.N
1637.8650	1636.8577	1636.7913	40.6	1 -	14	0	---	-.METTMSRPVLSPTR.I + 2 Oxidation (M)
2184.3567	2183.3494	2183.1368	97.4	747 -	765	1	---	R.KLAMSLEQALQTNNHLQTK.L + Oxidation (M)
2211.0339	2210.0266	2210.0498	-10.47	964 -	981	1	---	R.QELENRCQITANLEEAHR.W
2283.0969	2282.0896	2282.1324	-18.76	359 -	377	1	---	K.MENTRIIADHQAILQEEQK.M + Oxidation (M)

No match to: 835.4214, 842.4716, 850.5007, 853.4803, 853.4804, 870.5096, 882.5266, 882.5266, 951.5828, 953.4929, 972.5108, 982.5417, 996.5513, 1007.6395, 1017.6248, 1017.6248, 1019.6375, 1033.6567, 1033.6567, 1036.5132, 1045.5249, 1051.6622, 1051.6622, 1131.6577, 1133.6731, 1136.6519, 1151.6821, 1165.6888, 1165.6888, 1179.5533, 1194.5757, 1211.5695, 1234.7560, 1236.6587, 1277.6544, 1302.8082, 1316.8273, 1334.8298, 1334.8298, 1349.6478, 1368.6882, 1383.6498, 1421.6836, 1434.8480, 1567.6902, 1581.7833, 1581.7833, 1617.9998, 1617.9998, 1707.7130, 1732.0293, 1732.0293, 1734.8334, 1838.8518, 1900.9630, 1901.1735, 1993.8977, 2015.2006, 2215.0857, 2872.3440

16. A0A4W2DYM0 **Mass:** 53619 **Score:** 38 **Expect:** 21 **Matches:** 8

NAD-dependent protein lipoamidase sirtuin-4, mitochondrial OS=Bos indicus x Bos taurus OX=30522 GN=SIRT4 PE=3 SV=1

Observed	Mr(expt)	Mr(calc)	ppm	Start	End	Miss	Ions	Peptide
848.4913	847.4840	847.4287	65.3	444 -	451	0	---	R.SDDLASLK.L
864.5184	863.5111	863.5229	-13.61	202 -	208	1	---	R.FITLSKR.L
1234.7560	1233.7487	1233.7081	32.9	198 -	207	1	---	K.ELQRFITLSK.R
1383.6498	1382.6425	1382.6976	-39.86	50 -	61	1	---	R.MTTAFSLRVSDR.Q
1734.8334	1733.8261	1733.9438	-67.85	240 -	254	1	---	R.RPIQHGDFVRSAPVR.Q
1900.9630	1899.9557	1899.9169	20.4	257 -	271	1	---	R.YWARNFVGWPQFSSR.Q
2283.0969	2282.0896	2282.1841	-41.41	38 -	57	1	---	K.LENVSTHPLPPRMTTAFSLR.V + Oxidation (M)
2872.3440	2871.3367	2871.4225	-29.88	170 -	195	1	---	R.ANFSQQCSLRSTGLFVPPSPPLDPEK.V

No match to: 835.4214, 842.4716, 850.5007, 853.4803, 853.4804, 868.5114, 868.5114, 870.5096, 882.5266, 882.5266, 951.5828, 953.4929, 972.5108, 982.5417, 996.5513, 1007.6395, 1017.6248, 1017.6248, 1019.6375, 1031.5133, 1033.6567, 1033.6567, 1036.5132, 1045.5249, 1051.6622, 1051.6622, 1131.6577, 1133.6731, 1136.6519, 1147.6853, 1151.6821, 1165.6888, 1165.6888, 1179.5533, 1194.5757, 1211.5695, 1236.6587, 1277.6544, 1279.7057, 1300.7963, 1302.8082, 1316.8273, 1334.8298, 1334.8298, 1349.6478, 1368.6882, 1402.7100, 1421.6836, 1434.8480, 1448.8584, 1448.8584, 1475.6868, 1479.7338, 1567.6902, 1581.7833, 1581.7833, 1617.9998, 1617.9998, 1637.8650, 1707.7130, 1732.0293, 1732.0293, 1838.8518, 1901.1735, 1993.8977, 2015.2006, 2184.3567, 2211.0339, 2215.0857

17. A0A4W2GWY7 **Mass:** 132560 **Score:** 38 **Expect:** 24 **Matches:** 14

Euchromatic histone lysine methyltransferase 1 OS=Bos indicus x Bos taurus OX=30522 GN=EHMT1 PE=4 SV=1

Observed	Mr(expt)	Mr(calc)	ppm	Start	End	Miss	Ions	Peptide
842.4716	841.4643	841.4082	66.6	1095 -	1101	0	---	R.FYGNVSR.F
848.4913	847.4840	847.4222	73.0	486 -	492	1	---	R.METPKSR.E
953.4929	952.4856	952.5342	-50.96	159 -	168	1	---	K.KAPAPGADVK.V
1019.6375	1018.6302	1018.5295	98.9	866 -	874	1	---	R.DSDVTLKNK.E
1151.6821	1150.6748	1150.6169	50.4	271 -	279	1	---	K.QRTVIEMFK.S
1211.5695	1210.5622	1210.5587	2.91	1157 -	1166	1	---	K.LFSCRCGSPK.C
1368.6882	1367.6809	1367.6317	36.0	198 -	208	1	---	R.DHEEPKEELSR.G
1475.6868	1474.6795	1474.7086	-19.71	339 -	352	1	---	R.ASKDGVLAPDCSQK.V
1479.7338	1478.7265	1478.8358	-73.86	393 -	405	1	---	K.GKPDSPWIKPARK.R
1567.6902	1566.6829	1566.8154	-84.56	645 -	657	1	---	K.QLYFSARQGELQK.V
1732.0293	1731.0220	1730.8675	89.3	1102 -	1115	0	---	R.FINHHCEPNLVPVR.V
1732.0293	1731.0220	1730.8675	89.3	1102 -	1115	0	---	R.FINHHCEPNLVPVR.V
1838.8518	1837.8445	1837.9244	-43.46	273 -	289	1	---	R.TVIEMFKSITHSTGGSK.G + Oxidation (M)
2872.3440	2871.3367	2871.4185	-28.48	875 -	900	0	---	K.EGETPLQCASLNSQVWSALQVSQALR.D

No match to: 835.4214, 850.5007, 853.4803, 853.4804, 864.5184, 868.5114, 868.5114, 870.5096, 882.5266, 882.5266, 951.5828, 972.5108, 982.5417, 996.5513, 1007.6395, 1017.6248, 1017.6248, 1031.5133, 1033.6567, 1033.6567, 1036.5132, 1045.5249, 1051.6622, 1051.6622, 1131.6577, 1133.6731, 1136.6519, 1147.6853, 1165.6888, 1165.6888, 1179.5533, 1194.5757, 1234.7560, 1236.6587, 1277.6544, 1279.7057, 1300.7963, 1302.8082, 1316.8273, 1334.8298, 1334.8298, 1349.6478, 1383.6498, 1402.7100, 1421.6836, 1434.8480, 1448.8584, 1448.8584, 1581.7833, 1581.7833, 1617.9998, 1617.9998, 1637.8650, 1707.7130, 1734.8334, 1900.9630, 1901.1735, 1993.8977, 2015.2006, 2184.3567, 2211.0339, 2215.0857, 2283.0969

PDF-XChange Click to buy NOW! www.docu-track.com
PDF-XChange Click to buy NOW! www.docu-track.com

18. A0A4W2E8Y3 **Mass:** 132257 **Score:** 36 **Expect:** 31 **Matches:** 14
Euchromatic histone lysine methyltransferase 1 OS=Bos indicus x Bos taurus OX=30522 GN=EHMT1 PE=4 SV=1

Observed	Mr(expt)	Mr(calc)	ppm	Start	End	Miss	Ions	Peptide
842.4716	841.4643	841.4082	66.6	1153 -	1159	0	---	R.FYGNVSR.F
848.4913	847.4840	847.4222	73.0	486 -	492	1	---	R.METPKSR.E
953.4929	952.4856	952.5342	-50.96	159 -	168	1	---	K.KAPAPGADVK.V
1019.6375	1018.6302	1018.5295	98.9	924 -	932	1	---	R.DSDVTLKNK.E
1151.6821	1150.6748	1150.6169	50.4	271 -	279	1	---	K.QRTVIEMFK.S
1211.5695	1210.5622	1210.5288	27.6	1143 -	1152	0	---	K.DGELYCIDAR.F
1368.6882	1367.6809	1367.6317	36.0	198 -	208	1	---	R.DHEEPKEELSR.G
1475.6868	1474.6795	1474.7086	-19.71	339 -	352	1	---	R.ASKDGVLAPDCSQK.V
1479.7338	1478.7265	1478.8358	-73.86	393 -	405	1	---	K.GKPDSPWIKPARK.R
1567.6902	1566.6829	1566.8154	-84.56	703 -	715	1	---	K.QLYFSARQGELQK.V
1732.0293	1731.0220	1730.8675	89.3	1160 -	1173	0	---	R.FINHHCEPNLVPVR.V
1732.0293	1731.0220	1730.8675	89.3	1160 -	1173	0	---	R.FINHHCEPNLVPVR.V
1838.8518	1837.8445	1837.9244	-43.46	273 -	289	1	---	R.TVIEMFKSITHSTGGSK.G + Oxidation (M)
2872.3440	2871.3367	2871.4185	-28.48	933 -	958	0	---	K.EGETPLQCASLNSQVWSALQVSQALR.D

No match to: 835.4214, 850.5007, 853.4803, 853.4804, 864.5184, 868.5114, 868.5114, 870.5096, 882.5266, 882.5266, 951.5828, 972.5108, 982.5417, 996.5513, 1007.6395, 1017.6248, 1017.6248, 1031.5133, 1033.6567, 1033.6567, 1036.5132, 1045.5249, 1051.6622, 1051.6622, 1131.6577, 1133.6731, 1136.6519, 1147.6853, 1165.6888, 1165.6888, 1179.5533, 1194.5757, 1234.7560, 1236.6587, 1277.6544, 1279.7057, 1300.7963, 1302.8082, 1316.8273, 1334.8298, 1334.8298, 1349.6478, 1383.6498, 1402.7100, 1421.6836, 1434.8480, 1448.8584, 1448.8584, 1581.7833, 1581.7833, 1617.9998, 1617.9998, 1637.8650, 1707.7130, 1734.8334, 1900.9630, 1901.1735, 1993.8977, 2015.2006, 2184.3567, 2211.0339, 2215.0857, 2283.0969

19. A0A3Q1LPD0 **Mass:** 132257 **Score:** 36 **Expect:** 31 **Matches:** 14
Euchromatic histone lysine methyltransferase 1 OS=Bos taurus OX=9913 GN=EHMT1 PE=4 SV=1

Observed	Mr(expt)	Mr(calc)	ppm	Start	End	Miss	Ions	Peptide
842.4716	841.4643	841.4082	66.6	1153 -	1159	0	---	R.FYGNVSR.F
848.4913	847.4840	847.4222	73.0	486 -	492	1	---	R.METPKSR.E
953.4929	952.4856	952.5342	-50.96	159 -	168	1	---	K.KAPAPGADVK.V
1019.6375	1018.6302	1018.5295	98.9	924 -	932	1	---	R.DSDVTLKNK.E
1151.6821	1150.6748	1150.6169	50.4	271 -	279	1	---	K.QRTVIEMFK.S
1211.5695	1210.5622	1210.5288	27.6	1143 -	1152	0	---	K.DGELYCIDAR.F
1368.6882	1367.6809	1367.6317	36.0	198 -	208	1	---	R.DHEEPKEELSR.G
1475.6868	1474.6795	1474.7086	-19.71	339 -	352	1	---	R.ASKDGVLAPDCSQK.V
1479.7338	1478.7265	1478.8358	-73.86	393 -	405	1	---	K.GKPDSPWIKPARK.R
1567.6902	1566.6829	1566.8154	-84.56	703 -	715	1	---	K.QLYFSARQGELQK.V
1732.0293	1731.0220	1730.8675	89.3	1160 -	1173	0	---	R.FINHHCEPNLVPVR.V
1732.0293	1731.0220	1730.8675	89.3	1160 -	1173	0	---	R.FINHHCEPNLVPVR.V
1838.8518	1837.8445	1837.9244	-43.46	273 -	289	1	---	R.TVIEMFKSITHSTGGSK.G + Oxidation (M)
2872.3440	2871.3367	2871.4185	-28.48	933 -	958	0	---	K.EGETPLQCASLNSQVWSALQVSQALR.D

No match to: 835.4214, 850.5007, 853.4803, 853.4804, 864.5184, 868.5114, 868.5114, 870.5096, 882.5266, 882.5266, 951.5828, 972.5108, 982.5417, 996.5513, 1007.6395, 1017.6248, 1017.6248, 1031.5133, 1033.6567, 1033.6567, 1036.5132, 1045.5249, 1051.6622, 1051.6622, 1131.6577, 1133.6731, 1136.6519, 1147.6853, 1165.6888, 1165.6888, 1179.5533, 1194.5757, 1234.7560, 1236.6587, 1277.6544, 1279.7057, 1300.7963, 1302.8082, 1316.8273, 1334.8298, 1334.8298, 1349.6478, 1383.6498, 1402.7100, 1421.6836, 1434.8480, 1448.8584, 1448.8584, 1581.7833, 1581.7833, 1617.9998, 1617.9998, 1637.8650, 1707.7130, 1734.8334, 1900.9630, 1901.1735, 1993.8977, 2015.2006, 2184.3567, 2211.0339, 2215.0857, 2283.0969

20. A0A4W2C1C0 **Mass:** 67085 **Score:** 36 **Expect:** 33 **Matches:** 5
Heparanase 2 (inactive) OS=Bos indicus x Bos taurus OX=30522 GN=HPSE2 PE=4 SV=1

Observed	Mr(expt)	Mr(calc)	ppm	Start	End	Miss	Ions	Peptide
1236.6587	1235.6514	1235.6584	-5.63	312 -	322	0	---	K.NVIALLDGFMK.V + Oxidation (M)
1300.7963	1299.7890	1299.7047	64.8	299 -	310	0	---	R.ASLYGPNIGRPR.K
1732.0293	1731.0220	1730.8587	94.3	231 -	246	0	---	R.NPNNSWNSSSALSLLK.Y
1732.0293	1731.0220	1730.8587	94.3	231 -	246	0	24	R.NPNNSWNSSSALSLLK.Y
2872.3440	2871.3367	2871.5640	-79.16	539 -	564	0	---	K.SVQLNGQPLVMVDDGTLPELKPRPLR.A

No match to: 835.4214, 842.4716, 848.4913, 850.5007, 853.4803, 853.4804, 864.5184, 868.5114, 868.5114, 870.5096, 882.5266, 882.5266, 951.5828, 953.4929, 972.5108, 982.5417, 996.5513, 1007.6395, 1017.6248, 1017.6248, 1019.6375, 1031.5133, 1033.6567, 1033.6567, 1036.5132, 1045.5249, 1051.6622, 1051.6622, 1131.6577, 1133.6731, 1136.6519, 1147.6853, 1151.6821, 1165.6888, 1165.6888, 1179.5533, 1194.5757, 1211.5695, 1234.7560, 1277.6544, 1279.7057, 1302.8082, 1316.8273, 1334.8298, 1334.8298, 1349.6478, 1368.6882, 1383.6498, 1402.7100, 1421.6836, 1434.8480, 1448.8584, 1448.8584, 1475.6868, 1479.7338, 1567.6902, 1581.7833, 1581.7833, 1617.9998, 1617.9998, 1637.8650, 1707.7130, 1734.8334, 1838.8518, 1900.9630, 1901.1735, 1993.8977, 2015.2006, 2184.3567, 2211.0339, 2215.0857, 2283.0969

21. O46770 **Mass:** 19686 **Score:** 35 **Expect:** 43 **Matches:** 5
MHC class I heavy chain (Fragment) OS=Bos taurus OX=9913 PE=2 SV=1

Observed	Mr(expt)	Mr(calc)	ppm	Start	End	Miss	Ions	Peptide
1279.7057	1278.6984	1278.5993	77.5	142 -	152	0	---	K.WEAAGYAEVQR.N
1349.6478	1348.6405	1348.5983	31.3	106 -	116	1	---	R.RGFMQYGYDGR.D
1421.6836	1420.6763	1420.6834	-4.99	127 -	139	0	---	R.SWTAGETEAQITK.R
1567.6902	1566.6829	1566.7137	-19.61	153 -	164	0	---	R.NYLEGECVEWLR.R
2872.3440	2871.3367	2871.1977	48.4	78 -	103	0	---	R.GYYNQSEAGSHTLQWMSGCDVGPDGR.L

No match to: 835.4214, 842.4716, 848.4913, 850.5007, 853.4803, 853.4804, 864.5184, 868.5114, 868.5114, 870.5096, 882.5266, 882.5266, 951.5828, 953.4929, 972.5108, 982.5417, 996.5513, 1007.6395, 1017.6248, 1017.6248, 1019.6375, 1031.5133, 1033.6567, 1033.6567, 1036.5132, 1045.5249, 1051.6622, 1051.6622, 1131.6577, 1133.6731, 1136.6519, 1147.6853, 1151.6821, 1165.6888, 1165.6888, 1179.5533, 1194.5757, 1211.5695, 1234.7560, 1236.6587, 1277.6544, 1300.7963, 1302.8082, 1316.8273, 1334.8298, 1334.8298, 1368.6882, 1383.6498, 1402.7100, 1434.8480, 1448.8584, 1448.8584, 1475.6868, 1479.7338, 1581.7833, 1581.7833, 1617.9998, 1617.9998, 1637.8650, 1707.7130, 1732.0293, 1732.0293, 1734.8334, 1838.8518, 1900.9630, 1901.1735, 1993.8977, 2015.2006, 2184.3567, 2211.0339, 2215.0857, 2283.0969

22. U5TGS8 **Mass:** 86845 **Score:** 34 **Expect:** 48 **Matches:** 10
CPXV068 protein OS=Cowpox virus OX=10243 GN=CPXV068 PE=4 SV=1

Observed	Mr(expt)	Mr(calc)	ppm	Start	End	Miss	Ions	Peptide
842.4716	841.4643	841.4909	-31.59	511 -	518	0	---	K.IDSIPGLK.T
848.4913	847.4840	847.4803	4.34	223 -	229	0	---	R.FLDIVNK.N
850.5007	849.4934	849.4266	78.7	714 -	720	0	---	K.TLSLCEK.V
1368.6882	1367.6809	1367.5495	96.1	417 -	426	1	---	K.YGFCPMMMKR.L + 3 Oxidation (M)
1402.7100	1401.7027	1401.7292	-18.90	561 -	572	0	---	R.LASYGLYYIPSR.Y
1421.6836	1420.6763	1420.6544	15.4	519 -	529	1	---	K.TYNMKDITYEK.S + Oxidation (M)
1479.7338	1478.7265	1478.7803	-36.35	511 -	523	1	---	K.IDSIPGLKTYNMK.D
1900.9630	1899.9557	1899.9149	21.5	491 -	506	1	---	K.TNRVLCFDSLENSAFK.S
2015.2006	2014.1933	2014.0292	81.5	674 -	691	0	---	K.INELLSEPVSGVCIELDK.I
2872.3440	2871.3367	2871.4793	-49.66	265 -	289	1	---	R.TEDLGIYANIFFEDAIDITKLEITK.T

No match to: 835.4214, 853.4803, 853.4804, 864.5184, 868.5114, 868.5114, 870.5096, 882.5266, 882.5266, 951.5828, 953.4929, 972.5108, 982.5417, 996.5513, 1007.6395, 1017.6248, 1017.6248, 1019.6375, 1031.5133, 1033.6567, 1033.6567, 1036.5132,

PDF-XChange Click to buy NOW! www.docu-track.com

PDF-XChange Click to buy NOW! www.docu-track.com

1045.5249, 1051.6622, 1051.6622, 1131.6577, 1133.6731, 1136.6519, 1147.6853, 1151.6821, 1165.6888, 1165.6888, 1179.5533, 1194.5757, 1211.5695, 1234.7560, 1236.6587, 1277.6544, 1279.7057, 1300.7963, 1302.8082, 1316.8273, 1334.8298, 1334.8298, 1349.6478, 1383.6498, 1434.8480, 1448.8584, 1448.8584, 1475.6868, 1567.6902, 1581.7833, 1581.7833, 1617.9998, 1617.9998, 1637.8650, 1707.7130, 1732.0293, 1732.0293, 1734.8334, 1838.8518, 1901.1735, 1993.8977, 2184.3567, 2211.0339, 2215.0857, 2283.0969

23. A0A4W2CHB4 **Mass:** 38688 **Score:** 34 **Expect:** 50 **Matches:** 9

Glutathione S-transferase omega 1 OS=Bos indicus x Bos taurus OX=30522 GN=GSTO1 PE=3 SV=1

Observed	Mr(expt)	Mr(calc)	ppm	Start	End	Miss	Ions	Peptide
850.5007	849.4934	849.4241	81.6	304 -	310	0	---	K.LWMAAMK.K
870.5096	869.5023	869.5811	-90.54	140 -	146	1	---	K.RTLLVLR.A
1031.5133	1030.5060	1030.5335	-26.66	205 -	213	0	---	K.LLPGDPYEK.A
1279.7057	1278.6984	1278.5874	86.8	236 -	246	1	---	R.TQNKEDCSGLK.E
1300.7963	1299.7890	1299.6969	70.9	68 -	79	1	---	R.ADGCPSLALLRK.I
1402.7100	1401.7027	1401.7715	-49.07	311 -	323	1	---	K.KDPTVSSLLTDVK.T
1421.6836	1420.6763	1420.6631	9.28	129 -	139	1	---	R.VYSMRFCPYAK.R
1732.0293	1731.0220	1730.9679	31.3	111 -	128	1	---	R.SLGKGSAPPGPVPEGLIR.V
1732.0293	1731.0220	1730.9679	31.3	111 -	128	1	---	R.SLGKGSAPPGPVPEGLIR.V

No match to: 835.4214, 842.4716, 848.4913, 853.4803, 853.4804, 864.5184, 868.5114, 868.5114, 882.5266, 882.5266, 951.5828, 953.4929, 972.5108, 982.5417, 996.5513, 1007.6395, 1017.6248, 1017.6248, 1019.6375, 1033.6567, 1033.6567, 1036.5132, 1045.5249, 1051.6622, 1051.6622, 1131.6577, 1133.6731, 1136.6519, 1147.6853, 1151.6821, 1165.6888, 1165.6888, 1179.5533, 1194.5757, 1211.5695, 1234.7560, 1236.6587, 1277.6544, 1302.8082, 1316.8273, 1334.8298, 1334.8298, 1349.6478, 1368.6882, 1383.6498, 1434.8480, 1448.8584, 1448.8584, 1475.6868, 1479.7338, 1567.6902, 1581.7833, 1581.7833, 1617.9998, 1617.9998, 1637.8650, 1707.7130, 1734.8334, 1838.8518, 1900.9630, 1901.1735, 1993.8977, 2015.2006, 2184.3567, 2211.0339, 2215.0857, 2283.0969, 2872.3440

24. A0A4W2FWN4 **Mass:** 38719 **Score:** 34 **Expect:** 50 **Matches:** 9

Glutathione S-transferase omega 1 OS=Bos indicus x Bos taurus OX=30522 GN=GSTO1 PE=3 SV=1

Observed	Mr(expt)	Mr(calc)	ppm	Start	End	Miss	Ions	Peptide
850.5007	849.4934	849.4241	81.6	304 -	310	0	---	K.LWMAAMK.K
870.5096	869.5023	869.5811	-90.54	140 -	146	1	---	K.RTLLVLR.A
1031.5133	1030.5060	1030.5335	-26.66	205 -	213	0	---	K.LLPGDPYEK.A
1279.7057	1278.6984	1278.5874	86.8	236 -	246	1	---	R.TQNKEDCSGLK.E
1300.7963	1299.7890	1299.6969	70.9	68 -	79	1	---	R.ADGCPSLALLRK.I
1402.7100	1401.7027	1401.7715	-49.07	311 -	323	1	---	K.KDPTVSSLLTDVK.T
1421.6836	1420.6763	1420.6631	9.28	129 -	139	1	---	R.VYSMRFCPYAK.R
1732.0293	1731.0220	1730.9679	31.3	111 -	128	1	---	R.SLGKGSAPPGPVPEGLIR.V
1732.0293	1731.0220	1730.9679	31.3	111 -	128	1	---	R.SLGKGSAPPGPVPEGLIR.V

No match to: 835.4214, 842.4716, 848.4913, 853.4803, 853.4804, 864.5184, 868.5114, 868.5114, 882.5266, 882.5266, 951.5828, 953.4929, 972.5108, 982.5417, 996.5513, 1007.6395, 1017.6248, 1017.6248, 1019.6375, 1033.6567, 1033.6567, 1036.5132, 1045.5249, 1051.6622, 1051.6622, 1131.6577, 1133.6731, 1136.6519, 1147.6853, 1151.6821, 1165.6888, 1165.6888, 1179.5533, 1194.5757, 1211.5695, 1234.7560, 1236.6587, 1277.6544, 1302.8082, 1316.8273, 1334.8298, 1334.8298, 1349.6478, 1368.6882, 1383.6498, 1434.8480, 1448.8584, 1448.8584, 1475.6868, 1479.7338, 1567.6902, 1581.7833, 1581.7833, 1617.9998, 1617.9998, 1637.8650, 1707.7130, 1734.8334, 1838.8518, 1900.9630, 1901.1735, 1993.8977, 2015.2006, 2184.3567, 2211.0339, 2215.0857, 2283.0969, 2872.3440

25. A0A0K2YTL5 **Mass:** 9022 **Score:** 34 **Expect:** 51 **Matches:** 5

Uncharacterized protein OS=Cowpox virus OX=10243 GN=gCPXV0248 PE=4 SV=1

Observed	Mr(expt)	Mr(calc)	ppm	Start	End	Miss	Ions	Peptide
1036.5132	1035.5059	1035.5501	-42.70	72 -	79	0	---	K.FNLYRPNL.-
1131.6577	1130.6504	1130.6270	20.7	31 -	39	0	---	K.YIVLMIAHR.V + Oxidation (M)
1277.6544	1276.6471	1276.7292	-64.25	70 -	79	1	---	R.LKFNLYRPNL.-
1334.8298	1333.8225	1333.7275	71.2	58 -	69	0	---	R.STMELILLTASR.L
1334.8298	1333.8226	1333.7275	71.3	58 -	69	0	---	R.STMELILLTASR.L

No match to: 835.4214, 842.4716, 848.4913, 850.5007, 853.4803, 853.4804, 864.5184, 868.5114, 868.5114, 870.5096, 882.5266, 882.5266, 951.5828, 953.4929, 972.5108, 982.5417, 996.5513, 1007.6395, 1017.6248, 1017.6248, 1019.6375, 1031.5133, 1033.6567, 1033.6567, 1045.5249, 1051.6622, 1051.6622, 1133.6731, 1136.6519, 1147.6853, 1151.6821, 1165.6888, 1165.6888, 1179.5533, 1194.5757, 1211.5695, 1234.7560, 1236.6587, 1279.7057, 1300.7963, 1302.8082, 1316.8273, 1349.6478, 1368.6882, 1383.6498, 1402.7100, 1421.6836, 1434.8480, 1448.8584, 1448.8584, 1475.6868, 1479.7338, 1567.6902, 1581.7833, 1581.7833, 1617.9998, 1617.9998, 1637.8650, 1707.7130, 1732.0293, 1732.0293, 1734.8334, 1838.8518, 1900.9630, 1901.1735, 1993.8977, 2015.2006, 2184.3567, 2211.0339, 2215.0857, 2283.0969, 2872.3440

26. O46724 **Mass:** 19602 **Score:** 34 **Expect:** 51 **Matches:** 5

MHC class I heavy chain (Fragment) OS=Bos taurus OX=9913 PE=2 SV=1

Observed	Mr(expt)	Mr(calc)	ppm	Start	End	Miss	Ions	Peptide
1279.7057	1278.6984	1278.5993	77.5	142 -	152	0	---	K.WEAAGYAEVQR.N
1349.6478	1348.6405	1348.5983	31.3	106 -	116	1	---	R.RGFMQYGYDGR.D
1421.6836	1420.6763	1420.6834	-4.99	127 -	139	0	---	R.SWTAGETEAQITK.R
1567.6902	1566.6829	1566.7137	-19.61	153 -	164	0	---	R.NYLEGECVEWLR.R
2872.3440	2871.3367	2871.1977	48.4	78 -	103	0	---	R.GYYNQSEAGSHTLQWMSGCDVGPDGR.L

No match to: 835.4214, 842.4716, 848.4913, 850.5007, 853.4803, 853.4804, 864.5184, 868.5114, 868.5114, 870.5096, 882.5266, 882.5266, 951.5828, 953.4929, 972.5108, 982.5417, 996.5513, 1007.6395, 1017.6248, 1017.6248, 1019.6375, 1031.5133, 1033.6567, 1033.6567, 1036.5132, 1045.5249, 1051.6622, 1051.6622, 1131.6577, 1133.6731, 1136.6519, 1147.6853, 1151.6821, 1165.6888, 1165.6888, 1179.5533, 1194.5757, 1211.5695, 1234.7560, 1236.6587, 1277.6544, 1300.7963, 1302.8082, 1316.8273, 1334.8298, 1334.8298, 1368.6882, 1383.6498, 1402.7100, 1434.8480, 1448.8584, 1448.8584, 1475.6868, 1479.7338, 1581.7833, 1581.7833, 1617.9998, 1617.9998, 1637.8650, 1707.7130, 1732.0293, 1732.0293, 1734.8334, 1838.8518, 1900.9630, 1901.1735, 1993.8977, 2015.2006, 2184.3567, 2211.0339, 2215.0857, 2283.0969

27. O46765 **Mass:** 19602 **Score:** 34 **Expect:** 51 **Matches:** 5

MHC class I heavy chain (Fragment) OS=Bos taurus OX=9913 PE=2 SV=1

Observed	Mr(expt)	Mr(calc)	ppm	Start	End	Miss	Ions	Peptide
1279.7057	1278.6984	1278.5993	77.5	142 -	152	0	---	K.WEAAGYAEVQR.N
1349.6478	1348.6405	1348.5983	31.3	106 -	116	1	---	R.RGFMQYGYDGR.D
1421.6836	1420.6763	1420.6834	-4.99	127 -	139	0	---	R.SWTAGETEAQITK.R
1567.6902	1566.6829	1566.7137	-19.61	153 -	164	0	---	R.NYLEGECVEWLR.R
2872.3440	2871.3367	2871.1977	48.4	78 -	103	0	---	R.GYYNQSEAGSHTLQWMSGCDVGPDGR.L

No match to: 835.4214, 842.4716, 848.4913, 850.5007, 853.4803, 853.4804, 864.5184, 868.5114, 868.5114, 870.5096, 882.5266, 882.5266, 951.5828, 953.4929, 972.5108, 982.5417, 996.5513, 1007.6395, 1017.6248, 1017.6248, 1019.6375, 1031.5133, 1033.6567, 1033.6567, 1036.5132, 1045.5249, 1051.6622, 1051.6622, 1131.6577, 1133.6731, 1136.6519, 1147.6853, 1151.6821, 1165.6888, 1165.6888, 1179.5533, 1194.5757, 1211.5695, 1234.7560, 1236.6587, 1277.6544, 1300.7963, 1302.8082, 1316.8273, 1334.8298, 1334.8298, 1368.6882, 1383.6498, 1402.7100, 1434.8480, 1448.8584, 1448.8584, 1475.6868, 1479.7338, 1581.7833, 1581.7833, 1617.9998, 1617.9998, 1637.8650, 1707.7130, 1732.0293, 1732.0293, 1734.8334, 1838.8518, 1900.9630, 1901.1735, 1993.8977, 2015.2006, 2184.3567, 2211.0339, 2215.0857, 2283.0969

28. A0A4W2BML3 **Mass:** 25099 **Score:** 34 **Expect:** 52 **Matches:** 7

HESX homeobox 1 OS=Bos indicus x Bos taurus OX=30522 GN=HESX1 PE=4 SV=1

Observed	Mr(expt)	Mr(calc)	ppm	Start	End	Miss	Ions	Peptide

PDF-XChange
Click to buy NOW!
www.docu-track.com

PDF-XChange
Click to buy NOW!
www.docu-track.com

853.4803	852.4730	852.4130	70.4	132 - 137	0	---	R.ELSWYR.G
853.4804	852.4731	852.4130	70.5	132 - 137	0	---	R.ELSWYR.G
953.4929	952.4856	952.4688	17.7	199 - 206	0	---	R.ESQFLMAK.K
1131.6577	1130.6504	1130.5502	88.6	1 - 9	1	---	-.MQPDSELRR.L
1136.6519	1135.6446	1135.6237	18.4	35 - 45	1	---	K.FSGAIGKSLEK.R
1349.6478	1348.6405	1348.6557	-11.29	196 - 206	1	---	R.SHRESQFLMAK.K + Oxidation (M)
1383.6498	1382.6425	1382.6678	-18.27	23 - 34	0	---	K.DTDLHDITTPQK.F

No match to: 835.4214, 842.4716, 848.4913, 850.5007, 864.5184, 868.5114, 868.5114, 870.5096, 882.5266, 882.5266, 951.5828, 972.5108, 982.5417, 996.5513, 1007.6395, 1017.6248, 1017.6248, 1019.6375, 1031.5133, 1033.6567, 1033.6567, 1036.5132, 1045.5249, 1051.6622, 1051.6622, 1133.6731, 1147.6853, 1151.6821, 1165.6888, 1165.6888, 1179.5533, 1194.5757, 1211.5695, 1234.7560, 1236.6587, 1277.6544, 1279.7057, 1300.7963, 1302.8082, 1316.8273, 1334.8298, 1334.8298, 1368.6882, 1402.7100, 1421.6836, 1434.8480, 1448.8584, 1448.8584, 1475.6868, 1479.7338, 1567.6902, 1581.7833, 1581.7833, 1617.9998, 1617.9998, 1637.8650, 1707.7130, 1732.0293, 1732.0293, 1734.8334, 1838.8518, 1900.9630, 1901.1735, 1993.8977, 2015.2006, 2184.3567, 2211.0339, 2215.0857, 2283.0969, 2872.3440

29. A0A4W2E8V9 **Mass:** 138235 **Score:** 34 **Expect:** 52 **Matches:** 14

Euchromatic histone lysine methyltransferase 1 OS=Bos indicus x Bos taurus OX=30522 GN=EHMT1 PE=4 SV=1

Observed	Mr(expt)	Mr(calc)	ppm	Start - End	Miss	Ions	Peptide
842.4716	841.4643	841.4082	66.6	1153 - 1159	0	---	R.FYGNVSR.F
848.4913	847.4840	847.4222	73.0	486 - 492	1	---	R.METPKSR.E
953.4929	952.4856	952.5342	-50.96	159 - 168	1	---	K.KAPAPGADVK.V
1019.6375	1018.6302	1018.5295	98.9	924 - 932	1	---	R.DSDVTLKNK.E
1151.6821	1150.6748	1150.6169	50.4	271 - 279	1	---	K.QRTVIEMFK.S
1211.5695	1210.5622	1210.5587	2.91	1215 - 1224	1	---	K.LFSCRCGSPK.C
1368.6882	1367.6809	1367.6317	36.0	198 - 208	1	---	R.DHEEPKEELSR.G
1475.6868	1474.6795	1474.7086	-19.71	339 - 352	1	---	R.ASKDGVLAPDCSQK.V
1479.7338	1478.7265	1478.8358	-73.86	393 - 405	1	---	K.GKPDSPWIKPARK.R
1567.6902	1566.6829	1566.8154	-84.56	703 - 715	1	---	K.QLYFSARQGELQK.V
1732.0293	1731.0220	1730.8675	89.3	1160 - 1173	0	---	R.FINHHCEPNLVPVR.V
1732.0293	1731.0220	1730.8675	89.3	1160 - 1173	0	---	R.FINHHCEPNLVPVR.V
1838.8518	1837.8445	1837.9244	-43.46	273 - 289	1	---	R.TVIEMFKSITHSTGGSK.G + Oxidation (M)
2872.3440	2871.3367	2871.4185	-28.48	933 - 958	0	---	K.EGETPLQCASLNSQVWSALQVSQALR.D

No match to: 835.4214, 850.5007, 853.4803, 853.4804, 864.5184, 868.5114, 868.5114, 870.5096, 882.5266, 882.5266, 951.5828, 972.5108, 982.5417, 996.5513, 1007.6395, 1017.6248, 1017.6248, 1031.5133, 1033.6567, 1033.6567, 1036.5132, 1045.5249, 1051.6622, 1051.6622, 1131.6577, 1133.6731, 1136.6519, 1147.6853, 1165.6888, 1165.6888, 1179.5533, 1194.5757, 1234.7560, 1236.6587, 1277.6544, 1279.7057, 1300.7963, 1302.8082, 1316.8273, 1334.8298, 1334.8298, 1349.6478, 1383.6498, 1402.7100, 1421.6836, 1434.8480, 1448.8584, 1448.8584, 1581.7833, 1581.7833, 1617.9998, 1617.9998, 1637.8650, 1707.7130, 1734.8334, 1900.9630, 1901.1735, 1993.8977, 2015.2006, 2184.3567, 2211.0339, 2215.0857, 2283.0969

30. A0A3Q1MVR5 **Mass:** 138235 **Score:** 34 **Expect:** 52 **Matches:** 14

Euchromatic histone lysine methyltransferase 1 OS=Bos taurus OX=9913 GN=EHMT1 PE=4 SV=1

Observed	Mr(expt)	Mr(calc)	ppm	Start - End	Miss	Ions	Peptide
842.4716	841.4643	841.4082	66.6	1153 - 1159	0	---	R.FYGNVSR.F
848.4913	847.4840	847.4222	73.0	486 - 492	1	---	R.METPKSR.E
953.4929	952.4856	952.5342	-50.96	159 - 168	1	---	K.KAPAPGADVK.V
1019.6375	1018.6302	1018.5295	98.9	924 - 932	1	---	R.DSDVTLKNK.E
1151.6821	1150.6748	1150.6169	50.4	271 - 279	1	---	K.QRTVIEMFK.S
1211.5695	1210.5622	1210.5587	2.91	1215 - 1224	1	---	K.LFSCRCGSPK.C
1368.6882	1367.6809	1367.6317	36.0	198 - 208	1	---	R.DHEEPKEELSR.G
1475.6868	1474.6795	1474.7086	-19.71	339 - 352	1	---	R.ASKDGVLAPDCSQK.V
1479.7338	1478.7265	1478.8358	-73.86	393 - 405	1	---	K.GKPDSPWIKPARK.R
1567.6902	1566.6829	1566.8154	-84.56	703 - 715	1	---	K.QLYFSARQGELQK.V
1732.0293	1731.0220	1730.8675	89.3	1160 - 1173	0	---	R.FINHHCEPNLVPVR.V
1732.0293	1731.0220	1730.8675	89.3	1160 - 1173	0	---	R.FINHHCEPNLVPVR.V
1838.8518	1837.8445	1837.9244	-43.46	273 - 289	1	---	R.TVIEMFKSITHSTGGSK.G + Oxidation (M)
2872.3440	2871.3367	2871.4185	-28.48	933 - 958	0	---	K.EGETPLQCASLNSQVWSALQVSQALR.D

No match to: 835.4214, 850.5007, 853.4803, 853.4804, 864.5184, 868.5114, 868.5114, 870.5096, 882.5266, 882.5266, 951.5828, 972.5108, 982.5417, 996.5513, 1007.6395, 1017.6248, 1017.6248, 1031.5133, 1033.6567, 1033.6567, 1036.5132, 1045.5249, 1051.6622, 1051.6622, 1131.6577, 1133.6731, 1136.6519, 1147.6853, 1165.6888, 1165.6888, 1179.5533, 1194.5757, 1234.7560, 1236.6587, 1277.6544, 1279.7057, 1300.7963, 1302.8082, 1316.8273, 1334.8298, 1334.8298, 1349.6478, 1383.6498, 1402.7100, 1421.6836, 1434.8480, 1448.8584, 1448.8584, 1581.7833, 1581.7833, 1617.9998, 1617.9998, 1637.8650, 1707.7130, 1734.8334, 1900.9630, 1901.1735, 1993.8977, 2015.2006, 2184.3567, 2211.0339, 2215.0857, 2283.0969

Search Parameters

**Type of search**	**: Sequence Query**
**Enzyme**	**: Trypsin**
**Fixed modifications**	**: Carbamidomethyl (C)**
**Variable modifications**	**: Oxidation (M)**
**Mass values**	**: Monoisotopic**
**Protein Mass**	**: Unrestricted**
**Peptide Mass Tolerance**	**: ± 100 ppm**
**Fragment Mass Tolerance**	**: ± 0.3 Da**
**Max Missed Cleavages**	**: 1**
**Instrument type**	**: MALDI-TOF-TOF**
**Query1 (835.4214,1+)**	**: <no title>**
**Query2 (842.4716,1+)**	**: <no title>**
**Query3 (848.4913,1+)**	**: <no title>**
**Query4 (850.5007,1+)**	**: <no title>**
**Query5 (853.4803,1+)**	**: <no title>**
**Query6 (853.4804,1+)**	**: Locus:1..14.0.8**
**Query7 (864.5184,1+)**	**: <no title>**
**Query8 (868.5114,1+)**	**: <no title>**
**Query9 (868.5114,1+)**	**: Locus:1..14.0.10**
**Query10 (870.5096,1+)**	**: <no title>**
**Query11 (882.5266,1+)**	**: <no title>**
**Query12 (882.5266,1+)**	**: Locus:1..14.0.4**
**Query13 (951.5828,1+)**	**: <no title>**
**Query14 (953.4929,1+)**	**: <no title>**
**Query15 (972.5108,1+)**	**: <no title>**
**Query16 (982.5417,1+)**	**: <no title>**
**Query17 (996.5513,1+)**	**: <no title>**
**Query18 (1007.6395,1+)**	**: <no title>**
**Query19 (1017.6248,1+)**	**: Locus:1..14.0.7**
**Query20 (1017.6248,1+)**	**: <no title>**
**Query21 (1019.6375,1+)**	**: <no title>**
**Query22 (1031.5133,1+)**	**: <no title>**
**Query23 (1033.6567,1+)**	**: <no title>**

PDF-XChange
Click to buy NOW!
www.docu-track.com

PDF-XChange
Click to buy NOW!
www.docu-track.com

```
[illegible]uery24 (1033.6567,1+): Locus:1..14.0.11
Query25 (1036.5132,1+): <no title>
Query26 (1045.5249,1+): <no title>
Query27 (1051.6622,1+): <no title>
Query28 (1051.6622,1+): Locus:1..14.0.1
Query29 (1131.6577,1+): <no title>
Query30 (1133.6731,1+): <no title>
Query31 (1136.6519,1+): <no title>
Query32 (1147.6853,1+): <no title>
Query33 (1151.6821,1+): <no title>
Query34 (1165.6888,1+): <no title>
Query35 (1165.6888,1+): Locus:1..14.0.6
Query36 (1179.5533,1+): <no title>
Query37 (1194.5757,1+): <no title>
Query38 (1211.5695,1+): <no title>
Query39 (1234.7560,1+): <no title>
Query40 (1236.6587,1+): <no title>
Query41 (1277.6544,1+): <no title>
Query42 (1279.7057,1+): <no title>
Query43 (1300.7963,1+): <no title>
Query44 (1302.8082,1+): <no title>
Query45 (1316.8273,1+): <no title>
Query46 (1334.8298,1+): <no title>
Query47 (1334.8298,1+): Locus:1..14.0.3
Query48 (1349.6478,1+): <no title>
Query49 (1368.6882,1+): <no title>
Query50 (1383.6498,1+): <no title>
Query51 (1402.7100,1+): <no title>
Query52 (1421.6836,1+): <no title>
Query53 (1434.8480,1+): <no title>
Query54 (1448.8584,1+): <no title>
Query55 (1448.8584,1+): Locus:1..14.0.9
Query56 (1475.6868,1+): <no title>
Query57 (1479.7338,1+): <no title>
Query58 (1567.6902,1+): <no title>
Query59 (1581.7833,1+): <no title>
Query60 (1581.7833,1+): Locus:1..14.0.2
Query61 (1617.9998,1+): Locus:1..14.0.5
Query62 (1617.9998,1+): <no title>
Query63 (1637.8650,1+): <no title>
Query64 (1707.7130,1+): <no title>
Query65 (1732.0293,1+): <no title>
Query66 (1732.0293,1+): Locus:1..14.0.12
Query67 (1734.8334,1+): <no title>
Query68 (1838.8518,1+): <no title>
Query69 (1900.9630,1+): <no title>
Query70 (1901.1735,1+): <no title>
Query71 (1993.8977,1+): <no title>
Query72 (2015.2006,1+): <no title>
Query73 (2184.3567,1+): <no title>
Query74 (2211.0339,1+): <no title>
Query75 (2215.0857,1+): <no title>
Query76 (2283.0969,1+): <no title>
Query77 (2872.3440,1+): <no title>
```

Mascot: http://www.matrixscience.com/
