# Supplementary material for: Alpha-2-Macroglobulin as a New Promising Biomarker Improving the Diagnostic Sensitivity of Bovine Paratuberculosis
Source: Front Vet Sci. 2021 Mar 5;8:637716. doi: 10.3389/fvets.2021.637716 (PMC7973028; doi:10.3389/fvets.2021.637716)
Supplement: Supplementary Data 1 — Mascot search results and raw data files for LC-MS/MS analysis for the identification of specific blood proteins in cattle with Johne's disease. [file Data_Sheet_1.zip › mascot/12 Inter-alpha-trypsin inhibitor heavy chain_2.pdf]

PDF-XChange Click to buy NOW! www.docu-track.com

PDF-XChange Click to buy NOW! www.docu-track.com

MATRIX SCIENCE MASCOT Search Results

Protein View: F1MMD7

Inter-alpha-trypsin inhibitor heavy chain H4 OS=Bos taurus OX=9913 GN=ITIH4 PE=4 SV=3

Database:	Uniprot_bovine
Score:	74
Expect:	0.005
Nominal mass (M_r):	101620
Calculated pI:	6.22

Sequence similarity is available as an NCBI BLAST search of F1MMD7 against nr.

Search parameters

Enzyme:	Trypsin: cuts C-term side of KR unless next residue is P.
Fixed modifications:	Carbamidomethyl (C)
Variable modifications:	Oxidation (M)

Protein sequence coverage: 12%

Matched peptides shown in ***bold red***.

**1**	MKTPAPGRIH	SIVLVLLSLA	VLQTSKAQKV	QNDIDIYSLT	VDSKVSSRFA
**51**	HTVITSRVVN	KADAVREATF	QMELPKKAFI	TNFSMVIDGV	TYPGNIKEKA
**101**	AAQEQYSAAV	ARGESAGLVR	ATGRKTEQFQ	VSVSVAPAAK	**VTFELVYEEL**
**151**	**LAR**HLGAYEL	LLKVRPQQLV	KHLQMDIHIF	EPQGISFLET	ESTFMTNKLA
**201**	EALTTSQNKT	KAHVRFKPTL	SQQQKYPEK**Q**	**DTVLDGSFIV**	**R**YDVDRPLSG
**251**	GSIQIENGYF	VHYFAPDSLS	TIPKNVIFVI	DK**SGSMMGRK**	IKQTREALIK
**301**	ILDDLSPHDQ	FDLISFSSEA	TTWKPLLVPA	STENVNEAKS	YATGIQAQGG
**351**	TNINDAMLMA	VQLLEKANQE	ELLPEGSITL	IILLTDGDPT	VGETNPLNIQ
**401**	KNVRKAINGQ	HSLFCLGFGF	DVSYAFLEK**M**	**ALENGGLAR**R	IYEDSDSALQ
**451**	LQDFYQEVAN	PLMTSVAFEY	PSNAVESVTQ	DTFRVFFKGS	ELVVAGK**LRE**
**501**	**QSPDVLLAQI**	**R**GQLHRENIT	YMMMSHVAEQ	EEMFRSPK**YI**	**FHSFIER**LWA
**551**	YLTIQQLLEQ	MVSALDAEKQ	ALEARALSLS	LSYSFVTPLT	SMVITKPEGQ
**601**	EQSQVAEKPV	EDESR**GSRVY**	**LGPMR**FGHSV	GDRTSRKPGG	GLKLLNGTPL
**651**	FGPPGPPAAA	SPFHRMTSRL	VLPELMSPLA	PASAPSPTSG	PGGASHDTDF
**701**	RIKGTTPTAL	PFAPVQAPSV	ILPLPGQSVD	RLCVDLRRPQ	ELVNLLSDPD
**751**	QGVEVTGHFE	TAKARFSWIE	VTFENPQVQI	HASPEHVVMT	RNRRNSAYK**W**
**801**	**KETLYSVMPG**	**LK**VTMDKEGL	LLLSRPDR**VT**	**IGLLFWDGPG**	**K**GLR**LLLQNT**
**851**	**DR**FSSHVSGT	LGQFYQDVLW	GPLDTADDSK	RTLKVQGRDY	SATRELKLDY
**901**	QESPPGKEIS	CWSVEL			

Unformatted sequence string: 916 residues (for pasting into other applications).

Sort peptides by ◉ Residue Number ○ Increasing Mass ○ Decreasing Mass

Show predicted peptides also

PDF-XChange Click to buy NOW! www.docu-track.com
PDF-XChange Click to buy NOW! www.docu-track.com

Query	Start – End	Observed	Mr(expt)	Mr(calc)	ppm	M	Score	Pepti
59	141 – 153	1581.7833	1580.7760	1580.8450	-43.6	0		K.VTFE
60	141 – 153	1581.7833	1580.7761	1580.8450	-43.6	0	35	K.VTFE
48	230 – 241	1349.6478	1348.6405	1348.6987	-43.1	0		K.QDTV
5	283 – 290	853.4803	852.4730	852.3946	92.0	1		K.SGSM
6	283 – 290	853.4804	852.4731	852.3946	92.1	1		K.SGSM
22	430 – 439	1031.5133	1030.5060	1030.5229	-16.4	0		K.MALE
63	498 – 511	1637.8650	1636.8577	1636.9260	-41.7	1		K.LREQ
49	500 – 511	1368.6882	1367.6809	1367.7409	-43.8	0		R.EQSF
38	539 – 547	1211.5695	1210.5622	1210.6135	-42.3	0		K.YIFH
33	616 – 625	1151.6821	1150.6748	1150.5917	72.2	1		R.GSRV + Oxidat (M)
1	619 – 625	835.4214	834.4141	834.4422	-33.6	0		R.VYLG
58	800 – 812	1567.6902	1566.6829	1566.8116	-82.1	1		K.WKET + Oxidat (M)
51	829 – 841	1402.7100	1401.7027	1401.7657	-44.9	0		R.VTIG
15	845 – 852	972.5108	971.5035	971.5400	-37.5	0		R.LLLQ

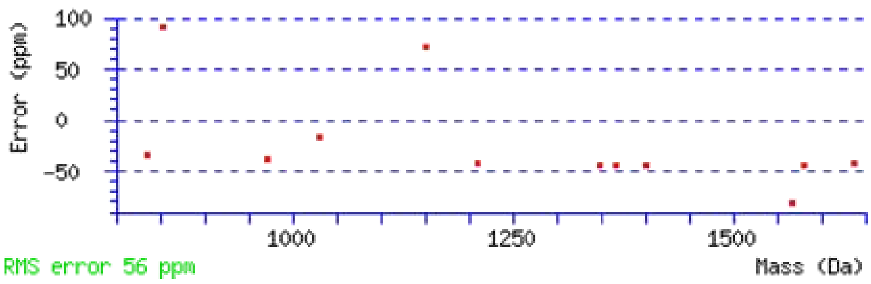

```
ID   F1MMD7_BOVIN            Unreviewed;       916 AA.
AC   F1MMD7;
DT   03-MAY-2011, integrated into UniProtKB/TrEMBL.
DT   10-APR-2019, sequence version 3.
DT   26-FEB-2020, entry version 60.
DE   SubName: Full=Inter-alpha-trypsin inhibitor heavy chain H4 {ECO:0000313|Ens
GN   Name=ITIH4 {ECO:0000313|Ensembl:ENSBTAP00000010330,
GN   ECO:0000313|VGNC:VGNC:30336};
OS   Bos taurus (Bovine).
OC   Eukaryota; Metazoa; Chordata; Craniata; Vertebrata; Euteleostomi; Mammalia;
OC   Eutheria; Laurasiatheria; Artiodactyla; Ruminantia; Pecora; Bovidae;
OC   Bovinae; Bos.
OX   NCBI_TaxID=9913 {ECO:0000313|Ensembl:ENSBTAP00000010330, ECO:0000313|Proteo
RN   [1] {ECO:0000313|Ensembl:ENSBTAP00000010330}
RP   IDENTIFICATION.
RC   STRAIN=Hereford {ECO:0000313|Ensembl:ENSBTAP00000010330};
RG   Ensembl;
RL   Submitted (MAR-2016) to UniProtKB.
RN   [2] {ECO:0000313|Ensembl:ENSBTAP00000010330, ECO:0000313|Proteomes:UP000009
RP   NUCLEOTIDE SEQUENCE [LARGE SCALE GENOMIC DNA].
RC   STRAIN=Hereford {ECO:0000313|Ensembl:ENSBTAP00000010330,
RC   ECO:0000313|Proteomes:UP000009136};
RA   Rosen B.D., Bickhart D.M., Koren S., Schnabel R.D., Hall R., Zimin A.,
RA   Dreischer C., Schultheiss S., Schroeder S.G., Elsik C.G., Couldrey C.,
RA   Liu G.E., Van Tassell C.P., Phillippy A.M., Smith T.P.L., Medrano J.F.;
RT   "ARS-UCD1.2.";
RL   Submitted (MAR-2018) to the EMBL/GenBank/DDBJ databases.
CC   -----------------------------------------------------------------------
CC   Copyrighted by the UniProt Consortium, see https://www.uniprot.org/terms
```

CC   Distributed under the Creative Commons Attribution (CC BY 4.0) License
CC   ---

```
DR   PRIDE; F1MMD7; -.
DR   Ensembl; ENSBTAT00000010330; ENSBTAP00000010330; ENSBTAG00000007850.
DR   VGNC; VGNC:30336; ITIH4.
DR   eggNOG; ENOG410IEJB; Eukaryota.
DR   eggNOG; COG2304; LUCA.
DR   GeneTree; ENSGT00940000161039; -.
DR   HOGENOM; CLU_008101_0_0_1; -.
DR   OrthoDB; 955432at2759; -.
DR   TreeFam; TF328982; -.
DR   Reactome; R-BTA-114608; Platelet degranulation.
DR   Proteomes; UP000009136; Chromosome 22.
DR   Bgee; ENSBTAG00000007850; Expressed in liver and 8 other tissues.
DR   ExpressionAtlas; F1MMD7; baseline.
DR   GO; GO:0004867; F:serine-type endopeptidase inhibitor activity; IEA:InterPr
DR   GO; GO:0030212; P:hyaluronan metabolic process; IEA:InterPro.
DR   Gene3D; 3.40.50.410; -; 1.
DR   InterPro; IPR010600; ITI_HC_C.
DR   InterPro; IPR013694; VIT.
DR   InterPro; IPR002035; VWF_A.
DR   InterPro; IPR036465; vWFA_dom_sf.
DR   Pfam; PF06668; ITI_HC_C; 1.
DR   Pfam; PF08487; VIT; 1.
DR   Pfam; PF00092; VWA; 1.
DR   SMART; SM00609; VIT; 1.
DR   SMART; SM00327; VWA; 1.
DR   SUPFAM; SSF53300; SSF53300; 1.
DR   PROSITE; PS51468; VIT; 1.
DR   PROSITE; PS50234; VWFA; 1.
PE   4: Predicted;
KW   Coiled coil {ECO:0000256|SAM:Coils};
KW   Reference proteome {ECO:0000313|Proteomes:UP000009136};
KW   Signal {ECO:0000256|SAM:SignalP}.
FT   REGION          597..616
FT                   /note="Disordered"
FT                   /evidence="ECO:0000256|SAM:MobiDB-lite"
FT   REGION          678..701
FT                   /note="Disordered"
FT                   /evidence="ECO:0000256|SAM:MobiDB-lite"
SQ   SEQUENCE   916 AA;  101513 MW;  C870BDBB3392C46F CRC64;
     MKTPAPGRIH SIVLVLLSLA VLQTSKAQKV QNDIDIYSLT VDSKVSSRFA HTVITSRVVN
     KADAVREATF QMELPKKAFI TNFSMVIDGV TYPGNIKEKA AAQEQYSAAV ARGESAGLVR
     ATGRKTEQFQ VSVSVAPAAK VTFELVYEEL LARHLGAYEL LLKVRPQQLV KHLQMDIHIF
     EPQGISFLET ESTFMTNKLA EALTTSQNKT KAHVRFKPTL SQQQKYPEKQ DTVLDGSFIV
     RYDVDRPLSG GSIQIENGYF VHYFAPDSLS TIPKNVIFVI DKSGSMMGRK IKQTREALIK
     ILDDLSPHDQ FDLISFSSEA TTWKPLLVPA STENVNEAKS YATGIQAQGG TNINDAMLMA
     VQLLEKANQE ELLPEGSITL IILLTDGDPT VGETNPLNIQ KNVRKAINGQ HSLFCLGFGF
     DVSYAFLEKM ALENGGLARR IYEDSDSALQ LQDFYQEVAN PLMTSVAFEY PSNAVESVTQ
     DTFRVFFKGS ELVVAGKLRE QSPDVLLAQI RGQLHRENIT YMMMSHVAEQ EEMFRSPKYI
     FHSFIERLWA YLTIQQLLEQ MVSALDAEKQ ALEARALSLS LSYSFVTPLT SMVITKPEGQ
     EQSQVAEKPV EDESRGSRVY LGPMRFGHSV GDRTSRKPGG GLKLLNGTPL FGPPGPPAAA
     SPFHRMTSRL VLPELMSPLA PASAPSPTSG PGGASHDTDF RIKGTTPTAL PFAPVQAPSV
     ILPLPGQSVD RLCVDLRRPQ ELVNLLSDPD QGVEVTGHFE TAKARFSWIE VTFENPQVQI
     HASPEHVVMT RNRRNSAYKW KETLYSVMPG LKVTMDKEGL LLLSRPDRVT IGLLFWDGPG
     KGLRLLLQNT DRFSSHVSGT LGQFYQDVLW GPLDTADDSK RTLKVQGRDY SATRELKLDY
     QESPPGKEIS CWSVEL
//
```

Mascot: http://www.matrixscience.com/
