# Supplementary material for: Alpha-2-Macroglobulin as a New Promising Biomarker Improving the Diagnostic Sensitivity of Bovine Paratuberculosis
Source: Front Vet Sci. 2021 Mar 5;8:637716. doi: 10.3389/fvets.2021.637716 (PMC7973028; doi:10.3389/fvets.2021.637716)
Supplement: Supplementary Data 1 — Mascot search results and raw data files for LC-MS/MS analysis for the identification of specific blood proteins in cattle with Johne's disease. [file Data_Sheet_1.zip › mascot/2 Transthyretin_1.pdf]

PDF-XChange
Click to buy NOW!
www.docu-track.com

PDF-XChange
Click to buy NOW!
www.docu-track.com

MATRIX SCIENCE Mascot Search Results

```
User          :
Email         :
Search title  : BAEDW\2020_3_5\2020_3_5\MSMS 14\N3
Database      : Uniprot_bovine bovine_20200316 (134392 sequences; 76244912 residues)
Timestamp     : 31 Mar 2020 at 01:08:22 GMT
Warning       : A Peptide summary report will usually give a much clearer picture of MS/MS search results.
Top Score     : 88 for A0A4W2BU20, Transthyretin OS=Bos indicus x Bos taurus OX=30522 GN=TTR PE=3 SV=1
```

Mascot Score Histogram

Protein score is -10*Log(P), where P is the probability that the observed match is a random event.
Protein scores greater than 64 are significant (p<0.05).
Protein scores are derived from ions scores as a non-probabilistic basis for ranking protein hits.

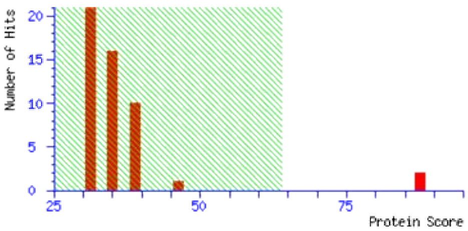

Protein Summary Report

Format As | Protein Summary (deprecated) | Help

Significance threshold p< 0.05   Max. number of hits 30

Preferred taxonomy All entries

Re-Search All   Search Unmatched

Index

	Accession	Mass	Score	Description
1.	A0A4W2BU20	15831	88	Transthyretin OS=Bos indicus x Bos taurus OX=30522 GN=TTR PE=3 SV=1
2.	O46375	15831	88	Transthyretin OS=Bos taurus OX=9913 GN=TTR PE=1 SV=1
3.	A0A4W2HSZ8	42719	47	Family with sequence similarity 50 member A OS=Bos indicus x Bos taurus OX=30522 GN=FAM50A PE=4 SV=1
4.	A0A4W2DZA0	147145	40	SRC kinase signaling inhibitor 1 OS=Bos indicus x Bos taurus OX=30522 GN=SRCIN1 PE=4 SV=1
5.	A0A3Q1ME33	130395	40	SRC kinase signaling inhibitor 1 OS=Bos taurus OX=9913 GN=SRCIN1 PE=4 SV=1
6.	A0A4W2F662	130890	38	SRC kinase signaling inhibitor 1 OS=Bos indicus x Bos taurus OX=30522 GN=SRCIN1 PE=4 SV=1
7.	A0A4W2FSP5	32222	38	TATA-box binding protein associated factor, RNA polymerase I subunit D OS=Bos indicus x Bos taurus OX=3
8.	Q32LB6	32222	38	TATA box-binding protein-associated factor RNA polymerase I subunit D OS=Bos taurus OX=9913 GN=TAF1D PE
9.	A0A3Q1LZS3	135577	38	SRC kinase signaling inhibitor 1 OS=Bos taurus OX=9913 GN=SRCIN1 PE=4 SV=1
10.	A0A4W2EDE8	135577	38	SRC kinase signaling inhibitor 1 OS=Bos indicus x Bos taurus OX=30522 GN=SRCIN1 PE=4 SV=1
11.	A0A3Q1MIM5	16207	37	Uncharacterized protein OS=Bos taurus OX=9913 PE=4 SV=1
12.	A0A452DJF3	31845	37	Phosphatidylinositol transfer protein beta isoform OS=Bos taurus OX=9913 GN=PITPNB PE=4 SV=1
13.	A0A3Q1N5E1	133286	37	SRC kinase signaling inhibitor 1 OS=Bos taurus OX=9913 GN=SRCIN1 PE=4 SV=1
14.	A0A4W2E9X7	28070	37	Family with sequence similarity 166 member B OS=Bos indicus x Bos taurus OX=30522 GN=FAM166B PE=4 SV=1
15.	A0A4W2FA54	140160	36	Calmodulin regulated spectrin associated protein 1 OS=Bos indicus x Bos taurus OX=30522 GN=CAMSAP1 PE=3
16.	A0A4W2GH39	119304	36	MCF.2 cell line derived transforming sequence-like 2 OS=Bos indicus x Bos taurus OX=30522 GN=MCF2L2 PE=
17.	A0A4W2F647	127627	36	SRC kinase signaling inhibitor 1 OS=Bos indicus x Bos taurus OX=30522 GN=SRCIN1 PE=4 SV=1
18.	A0A4W2DZ95	132512	35	SRC kinase signaling inhibitor 1 OS=Bos indicus x Bos taurus OX=30522 GN=SRCIN1 PE=4 SV=1
19.	A0A4W2CC31	172499	35	Calmodulin regulated spectrin associated protein 1 OS=Bos indicus x Bos taurus OX=30522 GN=CAMSAP1 PE=3
20.	A0A3Q1N0Q9	136628	35	SRC kinase signaling inhibitor 1 OS=Bos taurus OX=9913 GN=SRCIN1 PE=4 SV=1
21.	A0A4W2GMF0	148991	35	Coiled-coil domain containing 171 OS=Bos indicus x Bos taurus OX=30522 GN=CCDC171 PE=4 SV=1
22.	A0A3Q1LVB1	134506	34	SRC kinase signaling inhibitor 1 OS=Bos taurus OX=9913 GN=SRCIN1 PE=4 SV=1
23.	Q80RC9	50101	34	Bo17 (Fragment) OS=Bovine herpesvirus 4 OX=10385 GN=Bo17 PE=4 SV=1
24.	Q80RD0	49973	34	Bo17 (Fragment) OS=Bovine herpesvirus 4 OX=10385 GN=Bo17 PE=4 SV=1
25.	A0A4W2IBE1	60650	34	RIC8 guanine nucleotide exchange factor B OS=Bos indicus x Bos taurus OX=30522 GN=RIC8B PE=4 SV=1
26.	A0A4W2GMI1	61548	33	RIC8 guanine nucleotide exchange factor B OS=Bos indicus x Bos taurus OX=30522 GN=RIC8B PE=4 SV=1
27.	E1BD35	61548	33	RIC8 guanine nucleotide exchange factor B OS=Bos taurus OX=9913 GN=RIC8B PE=4 SV=3
28.	A0A3Q1M3W0	173871	33	Calmodulin regulated spectrin associated protein 1 OS=Bos taurus OX=9913 GN=CAMSAP1 PE=3 SV=1
29.	A0A4W2IDJ5	62224	33	RIC8 guanine nucleotide exchange factor B OS=Bos indicus x Bos taurus OX=30522 GN=RIC8B PE=4 SV=1
30.	A0A4W2F4T9	128155	33	SRC kinase signaling inhibitor 1 OS=Bos indicus x Bos taurus OX=30522 GN=SRCIN1 PE=4 SV=1

Results List

1. A0A4W2BU20 **Mass:** 15831 **Score:** 88 **Expect:** 0.00024 **Matches:** 5

Transthyretin OS=Bos indicus x Bos taurus OX=30522 GN=TTR PE=3 SV=1

Observed	Mr(expt)	Mr(calc)	ppm	Start	End	Miss	Ions	Peptide
1408.5692	1407.5619	1407.6306	-48.82	56 -	68	0	---	K.AADETWEPFASGK.T
1408.5692	1407.5619	1407.6306	-48.81	56 -	68	0	---	K.AADETWEPFASGK.T
2470.2014	2469.1941	2469.2791	-34.42	124 -	146	0	---	R.HYTIAALLSPYSYSTTALVSSPK.A
2477.1138	2476.1065	2476.2023	-38.69	101 -	123	0	66	K.SLGISPFHEFAEVVFTANDSGPR.H
2477.1138	2476.1065	2476.2023	-38.68	101 -	123	0	---	K.SLGISPFHEFAEVVFTANDSGPR.H

No match to: 811.4696, 842.4688, 848.4903, 850.0149, 850.5021, 853.4834, 856.4824, 864.4868, 865.9890, 868.5110, 870.5007, 876.9973, 882.5262, 882.5263, 892.9677, 951.5844, 953.5057, 996.5507, 1017.6264, 1017.6264, 1033.6548, 1033.6548, 1045.5144, 1045.5144, 1051.6622, 1051.6622, 1131.6659, 1133.6655, 1147.6840, 1151.6809, 1165.6895, 1165.6895, 1179.5537, 1236.6508, 1300.7965, 1302.8025, 1307.6320, 1316.8201, 1334.8311, 1334.8311, 1427.7416, 1430.5653, 1448.8605, 1448.8605, 1457.6237, 1475.6832, 1617.9929, 1617.9929, 1732.0273, 1775.7623, 1901.1764, 1940.8547, 1993.8938, 2056.9478, 2193.0378, 2211.0134, 2221.0344, 2225.0469, 2230.1272, 2233.0085, 2239.0554, 2248.9812, 2261.0361, 2265.0583, 2283.0833, 2305.0723, 2321.0693, 2459.1550, 2492.1714, 2499.1167, 3337.7234

2. O46375 **Mass:** 15831 **Score:** 88 **Expect:** 0.00024 **Matches:** 5

Transthyretin OS=Bos taurus OX=9913 GN=TTR PE=1 SV=1

Observed	Mr(expt)	Mr(calc)	ppm	Start	End	Miss	Ions	Peptide
1408.5692	1407.5619	1407.6306	-48.82	56 -	68	0	---	K.AADETWEPFASGK.T

PDF-XChange Click to buy NOW! www.docu-track.com
PDF-XChange Click to buy NOW! www.docu-track.com

1408.5692	1407.5619	1407.6306	-48.81	56	68	0	---	K.AADETWEPFASGK.T
2470.2014	2469.1941	2469.2791	-34.42	124	146	0	---	R.HYTIAALLSPYSYSTTALVSSPK.A
2477.1138	2476.1065	2476.2023	-38.69	101	123	0	66	K.SLGISPFHEFAEVVFTANDSGPR.H
2477.1138	2476.1065	2476.2023	-38.68	101	123	0	---	K.SLGISPFHEFAEVVFTANDSGPR.H

No match to: 811.4696, 842.4688, 848.4903, 850.0149, 850.5021, 853.4834, 856.4824, 864.4868, 865.9890, 868.5110, 870.5007, 876.9973, 882.5262, 882.5263, 892.9677, 951.5844, 953.5057, 996.5507, 1017.6264, 1017.6264, 1033.6548, 1045.5144, 1045.5144, 1051.6622, 1051.6622, 1131.6659, 1133.6655, 1147.6840, 1151.6809, 1165.6895, 1165.6895, 1179.5537, 1236.6508, 1300.7965, 1302.8025, 1307.6320, 1316.8201, 1334.8311, 1334.8311, 1427.7416, 1430.5653, 1448.8605, 1448.8605, 1457.6237, 1475.6832, 1617.9929, 1617.9929, 1732.0273, 1775.7623, 1901.1764, 1940.8547, 1993.8938, 2056.9478, 2193.0378, 2211.0134, 2221.0344, 2225.0469, 2230.1272, 2233.0085, 2239.0554, 2248.9812, 2261.0361, 2265.0583, 2283.0833, 2305.0723, 2321.0693, 2459.1550, 2492.1714, 2499.1167, 3337.7234

3. A0A4W2HSZ8 **Mass:** 42719 **Score:** 47 **Expect:** 2.9 **Matches:** 9
Family with sequence similarity 50 member A OS=Bos indicus x Bos taurus OX=30522 GN=FAM50A PE=4 SV=1

Observed	Mr(expt)	Mr(calc)	ppm	Start	End	Miss	Ions	Peptide
842.4688	841.4615	841.5385	-91.47	258	264	1	---	K.ALEILRK.D
848.4903	847.4830	847.4651	21.2	175	181	1	---	R.EEITTKK.K
1179.5537	1178.5464	1178.6448	-83.44	334	342	1	---	K.VVLRSWYEK.N
1775.7623	1774.7550	1774.8308	-42.71	34	50	0	---	K.EYSCNAGDLGLIPGPGR.S
2211.0134	2210.0061	2210.1160	-49.73	282	299	0	---	K.EDLIIPHHHSFYDFIVTK.A
2321.0693	2320.0620	2320.1158	-23.16	58	78	0	---	R.YPLQYSGLDNSMDNIVHGVAK.N
2477.1138	2476.1065	2476.3301	-90.29	2	24	0	---	M.PPPSLYPCPLDHAVIGPHLGVLK.G
2477.1138	2476.1065	2476.2169	-44.56	57	78	1	---	K.RYPLQYSGLDNSMDNIVHGVAK.N
2499.1167	2498.1094	2498.2516	-56.90	83	104	1	---	R.LSDFDFTHFTVGLVTLNDMKAK.Q

No match to: 811.4696, 850.0149, 850.5021, 853.4834, 856.4824, 864.4868, 865.9890, 868.5110, 870.5007, 876.9973, 882.5262, 882.5263, 892.9677, 951.5844, 953.5057, 996.5507, 1017.6264, 1017.6264, 1033.6548, 1045.5144, 1045.5144, 1051.6622, 1051.6622, 1131.6659, 1133.6655, 1147.6840, 1151.6809, 1165.6895, 1165.6895, 1236.6508, 1300.7965, 1302.8025, 1307.6320, 1316.8201, 1334.8311, 1334.8311, 1408.5692, 1408.5692, 1427.7416, 1430.5653, 1448.8605, 1448.8605, 1457.6237, 1475.6832, 1617.9929, 1617.9929, 1732.0273, 1901.1764, 1940.8547, 1993.8938, 2056.9478, 2193.0378, 2221.0344, 2225.0469, 2230.1272, 2233.0085, 2239.0554, 2248.9812, 2261.0361, 2265.0583, 2283.0833, 2305.0723, 2459.1550, 2470.2014, 2492.1714, 3337.7234

4. A0A4W2DZA0 **Mass:** 147145 **Score:** 40 **Expect:** 13 **Matches:** 19
SRC kinase signaling inhibitor 1 OS=Bos indicus x Bos taurus OX=30522 GN=SRCIN1 PE=4 SV=1

Observed	Mr(expt)	Mr(calc)	ppm	Start	End	Miss	Ions	Peptide
853.4834	852.4761	852.4705	6.59	436	443	0	---	R.LPPSSPQK.L
864.4868	863.4795	863.3998	92.3	712	718	0	---	R.DVSHNHR.L
953.5057	952.4984	952.4548	45.8	1320	1326	1	---	K.QRAEYMR.I
1033.6548	1032.6475	1032.5465	97.9	468	476	1	---	R.GSPVRQSFR.K
1033.6548	1032.6475	1032.5465	97.9	468	476	1	---	R.GSPVRQSFR.K
1131.6659	1130.6586	1130.6183	35.7	734	743	0	---	K.QLGETLTELK.A
1300.7965	1299.7892	1299.6783	85.4	1302	1314	1	---	R.LKTAQGQAGSPDK.G
1408.5692	1407.5619	1407.6664	-74.20	867	879	1	---	R.SMDKAVSVEAAER.D + Oxidation (M)
1408.5692	1407.5619	1407.6664	-74.19	867	879	1	---	R.SMDKAVSVEAAER.D + Oxidation (M)
1427.7416	1426.7343	1426.7780	-30.58	196	208	1	---	K.SPNTAILIKDEAR.N
1448.8605	1447.8532	1447.7208	91.4	1192	1206	0	---	R.GGAGIPSPWGPAPER.T
1448.8605	1447.8532	1447.7208	91.5	1192	1206	0	---	R.GGAGIPSPWGPAPER.T
1457.6237	1456.6164	1456.6735	-39.21	421	435	0	---	K.AAGGGPLYGDGYGFR.L
1617.9929	1616.9856	1616.8900	59.2	11	25	1	---	R.VPPQLGWQGPAGRVR.L
1617.9929	1616.9856	1616.8900	59.2	11	25	1	---	R.VPPQLGWQGPAGRVR.L
1775.7623	1774.7550	1774.7904	-19.94	567	585	0	---	K.IEGSNGAATPSAPCGSGSR.S
1993.8938	1992.8865	1993.0051	-59.48	1322	1338	1	---	R.AEYMRIQAQQQATKPSK.E + Oxidation (M)
2305.0723	2304.0650	2304.2181	-66.43	536	556	1	---	R.MEAMEKQIASLTGLVQSALLR.G + Oxidation (M)
2321.0693	2320.0620	2320.2130	-65.08	536	556	1	---	R.MEAMEKQIASLTGLVQSALLR.G + 2 Oxidation (M)

No match to: 811.4696, 842.4688, 848.4903, 850.0149, 850.5021, 856.4824, 865.9890, 868.5110, 870.5007, 876.9973, 882.5262, 882.5263, 892.9677, 951.5844, 996.5507, 1017.6264, 1017.6264, 1045.5144, 1045.5144, 1051.6622, 1051.6622, 1133.6655, 1147.6840, 1151.6809, 1165.6895, 1165.6895, 1179.5537, 1236.6508, 1302.8025, 1307.6320, 1316.8201, 1334.8311, 1334.8311, 1430.5653, 1475.6832, 1732.0273, 1901.1764, 1940.8547, 2056.9478, 2193.0378, 2211.0134, 2221.0344, 2225.0469, 2230.1272, 2233.0085, 2239.0554, 2248.9812, 2261.0361, 2265.0583, 2283.0833, 2459.1550, 2470.2014, 2477.1138, 2477.1138, 2492.1714, 2499.1167, 3337.7234

5. A0A3Q1ME33 **Mass:** 130395 **Score:** 40 **Expect:** 14 **Matches:** 16
SRC kinase signaling inhibitor 1 OS=Bos taurus OX=9913 GN=SRCIN1 PE=4 SV=1

Observed	Mr(expt)	Mr(calc)	ppm	Start	End	Miss	Ions	Peptide
853.4834	852.4761	852.4705	6.59	497	504	0	---	R.LPPSSPQK.L
864.4868	863.4795	863.3998	92.3	773	779	0	---	R.DVSHNHR.L
953.5057	952.4984	952.4548	45.8	1154	1160	1	---	K.QRAEYMR.I
1033.6548	1032.6475	1032.5465	97.9	529	537	1	---	R.GSPVRQSFR.K
1033.6548	1032.6475	1032.5465	97.9	529	537	1	---	R.GSPVRQSFR.K
1131.6659	1130.6586	1130.6183	35.7	795	804	0	---	K.QLGETLTELK.A
1300.7965	1299.7892	1299.6783	85.4	1136	1148	1	---	R.LKTAQGQAGSPDK.G
1408.5692	1407.5619	1407.6664	-74.20	928	940	1	---	R.SMDKAVSVEAAER.D + Oxidation (M)
1408.5692	1407.5619	1407.6664	-74.19	928	940	1	---	R.SMDKAVSVEAAER.D + Oxidation (M)
1427.7416	1426.7343	1426.7780	-30.58	257	269	1	---	K.SPNTAILIKDEAR.N
1457.6237	1456.6164	1456.6735	-39.21	482	496	0	---	K.AAGGGPLYGDGYGFR.L
1775.7623	1774.7550	1774.7904	-19.94	628	646	0	---	K.IEGSNGAATPSAPCGSGSR.S
1993.8938	1992.8865	1993.0051	-59.48	1156	1172	1	---	R.AEYMRIQAQQQATKPSK.E + Oxidation (M)
2305.0723	2304.0650	2304.2181	-66.43	597	617	1	---	R.MEAMEKQIASLTGLVQSALLR.G + Oxidation (M)
2321.0693	2320.0620	2320.2130	-65.08	597	617	1	---	R.MEAMEKQIASLTGLVQSALLR.G + 2 Oxidation (M)
2459.1550	2458.1477	2458.1169	12.5	1105	1127	1	---	K.DEDDEDRIIAELESGGGSVPPMK.V

No match to: 811.4696, 842.4688, 848.4903, 850.0149, 850.5021, 856.4824, 865.9890, 868.5110, 870.5007, 876.9973, 882.5262, 882.5263, 892.9677, 951.5844, 996.5507, 1017.6264, 1017.6264, 1045.5144, 1045.5144, 1051.6622, 1051.6622, 1133.6655, 1147.6840, 1151.6809, 1165.6895, 1165.6895, 1179.5537, 1236.6508, 1302.8025, 1307.6320, 1316.8201, 1334.8311, 1334.8311, 1430.5653, 1448.8605, 1448.8605, 1475.6832, 1617.9929, 1617.9929, 1732.0273, 1901.1764, 1940.8547, 2056.9478, 2193.0378, 2211.0134, 2221.0344, 2225.0469, 2230.1272, 2233.0085, 2239.0554, 2248.9812, 2261.0361, 2265.0583, 2283.0833, 2470.2014, 2477.1138, 2477.1138, 2492.1714, 2499.1167, 3337.7234

6. A0A4W2F662 **Mass:** 130890 **Score:** 38 **Expect:** 20 **Matches:** 16
SRC kinase signaling inhibitor 1 OS=Bos indicus x Bos taurus OX=30522 GN=SRCIN1 PE=4 SV=1

Observed	Mr(expt)	Mr(calc)	ppm	Start	End	Miss	Ions	Peptide
853.4834	852.4761	852.4705	6.59	504	511	0	---	R.LPPSSPQK.L
864.4868	863.4795	863.3998	92.3	780	786	0	---	R.DVSHNHR.L
953.5057	952.4984	952.4548	45.8	1161	1167	1	---	K.QRAEYMR.I
1033.6548	1032.6475	1032.5465	97.9	536	544	1	---	R.GSPVRQSFR.K
1033.6548	1032.6475	1032.5465	97.9	536	544	1	---	R.GSPVRQSFR.K
1131.6659	1130.6586	1130.6183	35.7	802	811	0	---	K.QLGETLTELK.A

PDF-XChange Click to buy NOW! www.docu-track.com

PDF-XChange Click to buy NOW! www.docu-track.com

1300.7965	1299.7892	1299.6783	85.4	1143 -	1155	1	---	R.LKTAQGQAGSPDK.G
1408.5692	1407.5619	1407.6664	-74.20	935 -	947	1	---	R.SMDKAVSVEAAER.D + Oxidation (M)
1408.5692	1407.5619	1407.6664	-74.19	935 -	947	1	---	R.SMDKAVSVEAAER.D + Oxidation (M)
1427.7416	1426.7343	1426.7780	-30.58	264 -	276	1	---	K.SPNTAILIKDEAR.N
1457.6237	1456.6164	1456.6735	-39.21	489 -	503	0	---	K.AAGGGPLYGDGYGFR.L
1775.7623	1774.7550	1774.7904	-19.94	635 -	653	0	---	K.IEGSNGAATPSAPCGSGSR.S
1993.8938	1992.8865	1993.0051	-59.48	1163 -	1179	1	---	R.AEYMRIQAQQQATKPSK.E + Oxidation (M)
2305.0723	2304.0650	2304.2181	-66.43	604 -	624	1	---	R.MEAMEKQIASLTGLVQSALLR.G + Oxidation (M)
2321.0693	2320.0620	2320.2130	-65.08	604 -	624	1	---	R.MEAMEKQIASLTGLVQSALLR.G + 2 Oxidation (M)
2459.1550	2458.1477	2458.1169	12.5	1112 -	1134	1	---	K.DEDDEDRIIAELESGGGSVPPMK.V

No match to: 811.4696, 842.4688, 848.4903, 850.0149, 850.5021, 856.4824, 865.9890, 868.5110, 870.5007, 876.9973, 882.5262, 882.5263, 892.9677, 951.5844, 996.5507, 1017.6264, 1017.6264, 1045.5144, 1045.5144, 1051.6622, 1051.6622, 1133.6655, 1147.6840, 1151.6809, 1165.6895, 1165.6895, 1179.5537, 1236.6508, 1302.8025, 1307.6320, 1316.8201, 1334.8311, 1334.8311, 1430.5653, 1448.8605, 1448.8605, 1475.6832, 1617.9929, 1617.9929, 1732.0273, 1901.1764, 1940.8547, 2056.9478, 2193.0378, 2211.0134, 2221.0344, 2225.0469, 2230.1272, 2233.0085, 2239.0554, 2248.9812, 2261.0361, 2265.0583, 2283.0833, 2470.2014, 2477.1138, 2477.1138, 2492.1714, 2499.1167, 3337.7234

7. A0A4W2FSP5 **Mass:** 32222 **Score:** 38 **Expect:** 20 **Matches:** 8

TATA-box binding protein associated factor, RNA polymerase I subunit D OS=Bos indicus x Bos taurus OX=30522 GN=TAF1D PE=4 SV=1

Observed	Mr(expt)	Mr(calc)	ppm	Start	End	Miss	Ions	Peptide
856.4824	855.4751	855.5039	-33.61	100 -	107	1	---	R.SVGRPKGR.R
868.5110	867.5037	867.5331	-33.81	49 -	55	1	---	R.KFVHIPK.N
1045.5144	1044.5071	1044.4698	35.7	267 -	274	1	---	K.HWIEDKMA.- + Oxidation (M)
1045.5144	1044.5071	1044.4698	35.7	267 -	274	1	---	K.HWIEDKMA.- + Oxidation (M)
1147.6840	1146.6767	1146.6397	32.3	144 -	153	0	---	K.ILTFEQAVAR.G
1901.1764	1900.1691	1899.9982	90.0	225 -	240	1	---	K.LVENSYFIISSEFPKK.K
2261.0361	2260.0288	2260.1183	-39.57	56 -	76	0	---	K.NTQATESSSDSSIEPRPLTLK.A
2283.0833	2282.0760	2282.2072	-57.49	124 -	142	1	---	K.RPGFPFLESENGRKPLPWR.K

No match to: 811.4696, 842.4688, 848.4903, 850.0149, 850.5021, 853.4834, 864.4868, 865.9890, 870.5007, 876.9973, 882.5262, 882.5263, 892.9677, 951.5844, 953.5057, 996.5507, 1017.6264, 1017.6264, 1033.6548, 1033.6548, 1051.6622, 1051.6622, 1131.6659, 1133.6655, 1151.6809, 1165.6895, 1165.6895, 1179.5537, 1236.6508, 1300.7965, 1302.8025, 1307.6320, 1316.8201, 1334.8311, 1334.8311, 1408.5692, 1408.5692, 1427.7416, 1430.5653, 1448.8605, 1448.8605, 1457.6237, 1475.6832, 1617.9929, 1617.9929, 1732.0273, 1775.7623, 1940.8547, 1993.8938, 2056.9478, 2193.0378, 2211.0134, 2221.0344, 2225.0469, 2230.1272, 2233.0085, 2239.0554, 2248.9812, 2265.0583, 2305.0723, 2321.0693, 2459.1550, 2470.2014, 2477.1138, 2477.1138, 2492.1714, 2499.1167, 3337.7234

8. Q32LB6 **Mass:** 32222 **Score:** 38 **Expect:** 20 **Matches:** 8

TATA box-binding protein-associated factor RNA polymerase I subunit D OS=Bos taurus OX=9913 GN=TAF1D PE=2 SV=1

Observed	Mr(expt)	Mr(calc)	ppm	Start	End	Miss	Ions	Peptide
856.4824	855.4751	855.5039	-33.61	100 -	107	1	---	R.SVGRPKGR.R
868.5110	867.5037	867.5331	-33.81	49 -	55	1	---	R.KFVHIPK.N
1045.5144	1044.5071	1044.4698	35.7	267 -	274	1	---	K.HWIEDKMA.- + Oxidation (M)
1045.5144	1044.5071	1044.4698	35.7	267 -	274	1	---	K.HWIEDKMA.- + Oxidation (M)
1147.6840	1146.6767	1146.6397	32.3	144 -	153	0	---	K.ILTFEQAVAR.G
1901.1764	1900.1691	1899.9982	90.0	225 -	240	1	---	K.LVENSYFIISSEFPKK.K
2261.0361	2260.0288	2260.1183	-39.57	56 -	76	0	---	K.NTQATESSSDSSIEPRPLTLK.A
2283.0833	2282.0760	2282.2072	-57.49	124 -	142	1	---	K.RPGFPFLESENGRKPLPWR.K

No match to: 811.4696, 842.4688, 848.4903, 850.0149, 850.5021, 853.4834, 864.4868, 865.9890, 870.5007, 876.9973, 882.5262, 882.5263, 892.9677, 951.5844, 953.5057, 996.5507, 1017.6264, 1017.6264, 1033.6548, 1033.6548, 1051.6622, 1051.6622, 1131.6659, 1133.6655, 1151.6809, 1165.6895, 1165.6895, 1179.5537, 1236.6508, 1300.7965, 1302.8025, 1307.6320, 1316.8201, 1334.8311, 1334.8311, 1408.5692, 1408.5692, 1427.7416, 1430.5653, 1448.8605, 1448.8605, 1457.6237, 1475.6832, 1617.9929, 1617.9929, 1732.0273, 1775.7623, 1940.8547, 1993.8938, 2056.9478, 2193.0378, 2211.0134, 2221.0344, 2225.0469, 2230.1272, 2233.0085, 2239.0554, 2248.9812, 2265.0583, 2305.0723, 2321.0693, 2459.1550, 2470.2014, 2477.1138, 2477.1138, 2492.1714, 2499.1167, 3337.7234

9. A0A3Q1LZS3 **Mass:** 135577 **Score:** 38 **Expect:** 21 **Matches:** 16

SRC kinase signaling inhibitor 1 OS=Bos taurus OX=9913 GN=SRCIN1 PE=4 SV=1

Observed	Mr(expt)	Mr(calc)	ppm	Start	End	Miss	Ions	Peptide
853.4834	852.4761	852.4705	6.59	546 -	553	0	---	R.LPPSSPQK.L
864.4868	863.4795	863.3998	92.3	822 -	828	0	---	R.DVSHNHR.L
953.5057	952.4984	952.4548	45.8	1203 -	1209	1	---	K.QRAEYMR.I
1033.6548	1032.6475	1032.5465	97.9	578 -	586	1	---	R.GSPVRQSFR.K
1033.6548	1032.6475	1032.5465	97.9	578 -	586	1	---	R.GSPVRQSFR.K
1131.6659	1130.6586	1130.6183	35.7	844 -	853	0	---	K.QLGETLTELK.A
1300.7965	1299.7892	1299.6783	85.4	1185 -	1197	1	---	R.LKTAQGQAGSPDK.G
1408.5692	1407.5619	1407.6664	-74.20	977 -	989	1	---	R.SMDKAVSVEAAER.D + Oxidation (M)
1408.5692	1407.5619	1407.6664	-74.19	977 -	989	1	---	R.SMDKAVSVEAAER.D + Oxidation (M)
1427.7416	1426.7343	1426.7780	-30.58	306 -	318	1	---	K.SPNTAILIKDEAR.N
1457.6237	1456.6164	1456.6735	-39.21	531 -	545	0	---	K.AAGGGPLYGDGYGFR.L
1775.7623	1774.7550	1774.7904	-19.94	677 -	695	0	---	K.IEGSNGAATPSAPCGSGSR.S
1993.8938	1992.8865	1993.0051	-59.48	1205 -	1221	1	---	R.AEYMRIQAQQQATKPSK.E + Oxidation (M)
2305.0723	2304.0650	2304.2181	-66.43	646 -	666	1	---	R.MEAMEKQIASLTGLVQSALLR.G + Oxidation (M)
2321.0693	2320.0620	2320.2130	-65.08	646 -	666	1	---	R.MEAMEKQIASLTGLVQSALLR.G + 2 Oxidation (M)
2459.1550	2458.1477	2458.1169	12.5	1154 -	1176	1	---	K.DEDDEDRIIAELESGGGSVPPMK.V

No match to: 811.4696, 842.4688, 848.4903, 850.0149, 850.5021, 856.4824, 865.9890, 868.5110, 870.5007, 876.9973, 882.5262, 882.5263, 892.9677, 951.5844, 996.5507, 1017.6264, 1017.6264, 1045.5144, 1045.5144, 1051.6622, 1051.6622, 1133.6655, 1147.6840, 1151.6809, 1165.6895, 1165.6895, 1179.5537, 1236.6508, 1302.8025, 1307.6320, 1316.8201, 1334.8311, 1334.8311, 1430.5653, 1448.8605, 1448.8605, 1475.6832, 1617.9929, 1617.9929, 1732.0273, 1901.1764, 1940.8547, 2056.9478, 2193.0378, 2211.0134, 2221.0344, 2225.0469, 2230.1272, 2233.0085, 2239.0554, 2248.9812, 2261.0361, 2265.0583, 2283.0833, 2470.2014, 2477.1138, 2477.1138, 2492.1714, 2499.1167, 3337.7234

10. A0A4W2EDE8 **Mass:** 135577 **Score:** 38 **Expect:** 21 **Matches:** 16

SRC kinase signaling inhibitor 1 OS=Bos indicus x Bos taurus OX=30522 GN=SRCIN1 PE=4 SV=1

Observed	Mr(expt)	Mr(calc)	ppm	Start	End	Miss	Ions	Peptide
853.4834	852.4761	852.4705	6.59	546 -	553	0	---	R.LPPSSPQK.L
864.4868	863.4795	863.3998	92.3	822 -	828	0	---	R.DVSHNHR.L
953.5057	952.4984	952.4548	45.8	1203 -	1209	1	---	K.QRAEYMR.I
1033.6548	1032.6475	1032.5465	97.9	578 -	586	1	---	R.GSPVRQSFR.K
1033.6548	1032.6475	1032.5465	97.9	578 -	586	1	---	R.GSPVRQSFR.K
1131.6659	1130.6586	1130.6183	35.7	844 -	853	0	---	K.QLGETLTELK.A
1300.7965	1299.7892	1299.6783	85.4	1185 -	1197	1	---	R.LKTAQGQAGSPDK.G
1408.5692	1407.5619	1407.6664	-74.20	977 -	989	1	---	R.SMDKAVSVEAAER.D + Oxidation (M)
1408.5692	1407.5619	1407.6664	-74.19	977 -	989	1	---	R.SMDKAVSVEAAER.D + Oxidation (M)
1427.7416	1426.7343	1426.7780	-30.58	306 -	318	1	---	K.SPNTAILIKDEAR.N
1457.6237	1456.6164	1456.6735	-39.21	531 -	545	0	---	K.AAGGGPLYGDGYGFR.L

PDF-XChange Click to buy NOW! www.docu-track.com

PDF-XChange Click to buy NOW! www.docu-track.com

1775.7623	1774.7550	1774.7904	-19.94	677	695	0	---	K.IEGSNGAATPSAPCGSGSR.S
1993.8938	1992.8865	1993.0051	-59.48	1205	1221	1	---	R.AEYMRIQAQQQATKPSK.E + Oxidation (M)
2305.0723	2304.0650	2304.2181	-66.43	646	666	1	---	R.MEAMEKQIASLTGLVQSALLR.G + Oxidation (M)
2321.0693	2320.0620	2320.2130	-65.08	646	666	1	---	R.MEAMEKQIASLTGLVQSALLR.G + 2 Oxidation (M)
2459.1550	2458.1477	2458.1169	12.5	1154	1176	1	---	K.DEDDEDRIIAELESGGGSVPPMK.V

No match to: 811.4696, 842.4688, 848.4903, 850.0149, 850.5021, 856.4824, 865.9890, 868.5110, 870.5007, 876.9973, 882.5262, 882.5263, 892.9677, 951.5844, 996.5507, 1017.6264, 1017.6264, 1045.5144, 1045.5144, 1051.6622, 1051.6622, 1133.6655, 1147.6840, 1151.6809, 1165.6895, 1165.6895, 1179.5537, 1236.6508, 1302.8025, 1307.6320, 1316.8201, 1334.8311, 1334.8311, 1430.5653, 1448.8605, 1448.8605, 1475.6832, 1617.9929, 1617.9929, 1732.0273, 1901.1764, 1940.8547, 2056.9478, 2193.0378, 2211.0134, 2221.0344, 2225.0469, 2230.1272, 2233.0085, 2239.0554, 2248.9812, 2261.0361, 2265.0583, 2283.0833, 2470.2014, 2477.1138, 2477.1138, 2492.1714, 2499.1167, 3337.7234

11. A0A3Q1MIM5 **Mass:** 16207 **Score:** 37 **Expect:** 24 **Matches:** 5

Uncharacterized protein OS=Bos taurus OX=9913 PE=4 SV=1

Observed	Mr(expt)	Mr(calc)	ppm	Start	End	Miss	Ions	Peptide
870.5007	869.4934	869.4130	92.5	40	47	0	---	K.AEEEAPPK.E
951.5844	950.5771	950.5081	72.6	134	140	1	---	K.MVKMLWK.I + Oxidation (M)
1940.8547	1939.8474	1940.0190	-88.42	55	73	1	---	K.TPAGHAIPPKTGSMVGIYK.G + Oxidation (M)
2056.9478	2055.9405	2055.9901	-24.13	114	130	0	---	K.LFYQGSEEIESSVHYLR.E
3337.7234	3336.7161	3336.6105	31.7	76	104	1	---	K.TFTQSPTQPGQHQDHSSGFIPFKWPAWPK.T

No match to: 811.4696, 842.4688, 848.4903, 850.0149, 850.5021, 853.4834, 856.4824, 864.4868, 865.9890, 868.5110, 876.9973, 882.5262, 882.5263, 892.9677, 953.5057, 996.5507, 1017.6264, 1017.6264, 1033.6548, 1033.6548, 1045.5144, 1045.5144, 1051.6622, 1051.6622, 1131.6659, 1133.6655, 1147.6840, 1151.6809, 1165.6895, 1165.6895, 1179.5537, 1236.6508, 1300.7965, 1302.8025, 1307.6320, 1316.8201, 1334.8311, 1334.8311, 1408.5692, 1408.5692, 1427.7416, 1430.5653, 1448.8605, 1448.8605, 1457.6237, 1475.6832, 1617.9929, 1617.9929, 1732.0273, 1775.7623, 1901.1764, 1993.8938, 2193.0378, 2211.0134, 2221.0344, 2225.0469, 2230.1272, 2233.0085, 2239.0554, 2248.9812, 2261.0361, 2265.0583, 2283.0833, 2305.0723, 2321.0693, 2459.1550, 2470.2014, 2477.1138, 2477.1138, 2492.1714, 2499.1167

12. A0A452DJF3 **Mass:** 31845 **Score:** 37 **Expect:** 24 **Matches:** 9

Phosphatidylinositol transfer protein beta isoform OS=Bos taurus OX=9913 GN=PITPNB PE=4 SV=1

Observed	Mr(expt)	Mr(calc)	ppm	Start	End	Miss	Ions	Peptide
848.4903	847.4830	847.5531	-82.70	195	201	1	---	K.LVTIKFK.W
868.5110	867.5037	867.4603	50.1	171	178	0	---	R.GPLGPNWK.K
996.5507	995.5434	995.5552	-11.87	171	179	1	---	R.GPLGPNWKK.E
1051.6622	1050.6549	1050.5896	62.2	1	8	1	---	-.MVLIKEFR.V + Oxidation (M)
1051.6622	1050.6550	1050.5896	62.2	1	8	1	---	-.MVLIKEFR.V + Oxidation (M)
1179.5537	1178.5464	1178.6237	-65.53	200	208	1	---	K.FKWWGLQSK.V
1307.6320	1306.6247	1306.6227	1.53	235	244	0	---	K.WIDLTMEDIR.R + Oxidation (M)
1457.6237	1456.6164	1456.7384	-83.75	74	86	0	---	R.MIAPEGSLVFHEK.A
2321.0693	2320.0620	2320.0768	-6.39	87	104	1	---	K.AWNAYPYCRTILQNEYMK.D

No match to: 811.4696, 842.4688, 850.0149, 850.5021, 853.4834, 856.4824, 864.4868, 865.9890, 870.5007, 876.9973, 882.5262, 882.5263, 892.9677, 951.5844, 953.5057, 1017.6264, 1017.6264, 1033.6548, 1033.6548, 1045.5144, 1045.5144, 1131.6659, 1133.6655, 1147.6840, 1151.6809, 1165.6895, 1165.6895, 1236.6508, 1300.7965, 1302.8025, 1316.8201, 1334.8311, 1334.8311, 1408.5692, 1408.5692, 1427.7416, 1430.5653, 1448.8605, 1448.8605, 1475.6832, 1617.9929, 1617.9929, 1732.0273, 1775.7623, 1901.1764, 1940.8547, 1993.8938, 2056.9478, 2193.0378, 2211.0134, 2221.0344, 2225.0469, 2230.1272, 2233.0085, 2239.0554, 2248.9812, 2261.0361, 2265.0583, 2283.0833, 2305.0723, 2459.1550, 2470.2014, 2477.1138, 2477.1138, 2492.1714, 2499.1167, 3337.7234

13. A0A3Q1N5E1 **Mass:** 133286 **Score:** 37 **Expect:** 27 **Matches:** 16

SRC kinase signaling inhibitor 1 OS=Bos taurus OX=9913 GN=SRCIN1 PE=4 SV=1

Observed	Mr(expt)	Mr(calc)	ppm	Start	End	Miss	Ions	Peptide
853.4834	852.4761	852.4705	6.59	521	528	0	---	R.LPPSSPQK.L
864.4868	863.4795	863.3998	92.3	797	803	0	---	R.DVSHNHR.L
953.5057	952.4984	952.4548	45.8	1178	1184	1	---	K.QRAEYMR.I
1033.6548	1032.6475	1032.5465	97.9	553	561	1	---	R.GSPVRQSFR.K
1033.6548	1032.6475	1032.5465	97.9	553	561	1	---	R.GSPVRQSFR.K
1131.6659	1130.6586	1130.6183	35.7	819	828	0	---	K.QLGETLTELK.A
1300.7965	1299.7892	1299.6783	85.4	1160	1172	1	---	R.LKTAQGQAGSPDK.G
1408.5692	1407.5619	1407.6664	-74.20	952	964	1	---	R.SMDKAVSVEAAER.D + Oxidation (M)
1408.5692	1407.5619	1407.6664	-74.19	952	964	1	---	R.SMDKAVSVEAAER.D + Oxidation (M)
1427.7416	1426.7343	1426.7780	-30.58	281	293	1	---	K.SPNTAILIKDEAR.N
1457.6237	1456.6164	1456.6735	-39.21	506	520	0	---	K.AAGGGPLYGDGYGFR.L
1775.7623	1774.7550	1774.7904	-19.94	652	670	0	---	K.IEGSNGAATPSAPCGSGSR.S
1993.8938	1992.8865	1993.0051	-59.48	1180	1196	1	---	R.AEYMRIQAQQQATKPSK.E + Oxidation (M)
2305.0723	2304.0650	2304.2181	-66.43	621	641	1	---	R.MEAMEKQIASLTGLVQSALLR.G + Oxidation (M)
2321.0693	2320.0620	2320.2130	-65.08	621	641	1	---	R.MEAMEKQIASLTGLVQSALLR.G + 2 Oxidation (M)
2459.1550	2458.1477	2458.1169	12.5	1129	1151	1	---	K.DEDDEDRIIAELESGGGSVPPMK.V

No match to: 811.4696, 842.4688, 848.4903, 850.0149, 850.5021, 856.4824, 865.9890, 868.5110, 870.5007, 876.9973, 882.5262, 882.5263, 892.9677, 951.5844, 996.5507, 1017.6264, 1017.6264, 1045.5144, 1045.5144, 1051.6622, 1051.6622, 1133.6655, 1147.6840, 1151.6809, 1165.6895, 1165.6895, 1179.5537, 1236.6508, 1302.8025, 1307.6320, 1316.8201, 1334.8311, 1334.8311, 1430.5653, 1448.8605, 1448.8605, 1475.6832, 1617.9929, 1617.9929, 1732.0273, 1901.1764, 1940.8547, 2056.9478, 2193.0378, 2211.0134, 2221.0344, 2225.0469, 2230.1272, 2233.0085, 2239.0554, 2248.9812, 2261.0361, 2265.0583, 2283.0833, 2470.2014, 2477.1138, 2477.1138, 2492.1714, 2499.1167, 3337.7234

14. A0A4W2E9X7 **Mass:** 28070 **Score:** 37 **Expect:** 28 **Matches:** 8

Family with sequence similarity 166 member B OS=Bos indicus x Bos taurus OX=30522 GN=FAM166B PE=4 SV=1

Observed	Mr(expt)	Mr(calc)	ppm	Start	End	Miss	Ions	Peptide
1131.6659	1130.6586	1130.6812	-19.94	59	68	0	---	R.TLLPPIQPPR.S
1133.6655	1132.6582	1132.6353	20.3	208	217	1	---	K.DPKHLPALSR.T
1147.6840	1146.6767	1146.6258	44.4	76	85	0	---	R.SLPVRPGHER.L
1408.5692	1407.5619	1407.6646	-72.92	165	176	0	---	K.FFMSGFTGYVPR.A
1408.5692	1407.5619	1407.6646	-72.91	165	176	0	---	K.FFMSGFTGYVPR.A
2233.0085	2232.0012	2231.9753	11.6	145	163	1	---	K.DQEPKPEASPYSMDDRDPR.K
2248.9812	2247.9739	2247.9702	1.65	145	163	1	---	K.DQEPKPEASPYSMDDRDPR.K + Oxidation (M)
2492.1714	2491.1641	2491.2094	-18.15	179	200	0	---	R.FLFGSSFPVLSNQALQEFGEMK.S + Oxidation (M)

No match to: 811.4696, 842.4688, 848.4903, 850.0149, 850.5021, 853.4834, 856.4824, 864.4868, 865.9890, 868.5110, 870.5007, 876.9973, 882.5262, 882.5263, 892.9677, 951.5844, 953.5057, 996.5507, 1017.6264, 1017.6264, 1033.6548, 1033.6548, 1045.5144, 1045.5144, 1051.6622, 1051.6622, 1151.6809, 1165.6895, 1165.6895, 1179.5537, 1236.6508, 1300.7965, 1302.8025, 1307.6320, 1316.8201, 1334.8311, 1334.8311, 1427.7416, 1430.5653, 1448.8605, 1448.8605, 1457.6237, 1475.6832, 1617.9929, 1617.9929, 1732.0273, 1775.7623, 1901.1764, 1940.8547, 1993.8938, 2056.9478, 2193.0378, 2211.0134, 2221.0344, 2225.0469, 2230.1272, 2239.0554, 2261.0361, 2265.0583, 2283.0833, 2305.0723, 2321.0693, 2459.1550, 2470.2014, 2477.1138, 2477.1138, 2499.1167, 3337.7234

15. A0A4W2FA54 **Mass:** 140160 **Score:** 36 **Expect:** 30 **Matches:** 16

Calmodulin regulated spectrin associated protein 1 OS=Bos indicus x Bos taurus OX=30522 GN=CAMSAP1 PE=3 SV=1

Observed	Mr(expt)	Mr(calc)	ppm	Start	End	Miss	Ions	Peptide

PDF-XChange Click to buy NOW! www.docu-track.com

PDF-XChange Click to buy NOW! www.docu-track.com

842.4688	841.4615	841.5134	-61.61	598	604	1	---	R.QRLQLGK.A
864.4868	863.4795	863.4613	21.1	1018	1023	1	---	K.QEYLRR.K
870.5007	869.4934	869.4243	79.5	471	477	1	---	K.VKEHDDK.D
951.5844	950.5771	950.4821	100.0	2	9	0	---	M.ADSLYNIR.L
1045.5144	1044.5071	1044.5451	-36.38	64	72	1	---	R.DVQELKDAK.S
1045.5144	1044.5071	1044.5451	-36.38	64	72	1	---	R.DVQELKDAK.S
1151.6809	1150.6736	1150.5843	77.6	318	328	1	---	R.SGDGHQPLGRK.K
1165.6895	1164.6822	1164.5775	89.9	895	904	0	---	K.ENVPLDEPPR.S
1165.6895	1164.6822	1164.5775	89.9	895	904	0	---	K.ENVPLDEPPR.S
1179.5537	1178.5464	1178.5905	-37.37	317	327	1	---	R.RSGDGHQPLGR.K
1302.8025	1301.7952	1301.7078	67.1	907	918	0	---	K.ASLIEVDLSDLK.A
2193.0378	2192.0305	2192.1048	-33.90	1248	1266	0	---	K.TMSVSVDALTIHNHLWQPK.R + Oxidation (M)
2305.0723	2304.0650	2304.2188	-66.72	277	296	1	---	K.TDSFFLEPLMPAVLRPAKEK.Q + Oxidation (M)
2459.1550	2458.1477	2458.1711	-9.50	907	929	1	---	K.ASLIEVDLSDLKAPDEDGETEGR.D
2477.1138	2476.1065	2476.2420	-54.72	1174	1193	1	---	K.NSILEELEKCDANHYIILFR.D
2477.1138	2476.1065	2476.2420	-54.71	1174	1193	1	---	K.NSILEELEKCDANHYIILFR.D

No match to: 811.4696, 848.4903, 850.0149, 850.5021, 853.4834, 856.4824, 865.9890, 868.5110, 876.9973, 882.5262, 882.5263, 892.9677, 953.5057, 996.5507, 1017.6264, 1017.6264, 1033.6548, 1033.6548, 1051.6622, 1051.6622, 1131.6659, 1133.6655, 1147.6840, 1236.6508, 1300.7965, 1307.6320, 1316.8201, 1334.8311, 1408.5692, 1408.5692, 1427.7416, 1430.5653, 1448.8605, 1448.8605, 1457.6237, 1475.6832, 1617.9929, 1617.9929, 1732.0273, 1775.7623, 1901.1764, 1940.8547, 1993.8938, 2056.9478, 2211.0134, 2221.0344, 2225.0469, 2230.1272, 2233.0085, 2239.0554, 2248.9812, 2261.0361, 2265.0583, 2283.0833, 2321.0693, 2470.2014, 2492.1714, 2499.1167, 3337.7234

16. A0A4W2GH39 **Mass:** 119304 **Score:** 36 **Expect:** 34 **Matches:** 18
MCF.2 cell line derived transforming sequence-like 2 OS=Bos indicus x Bos taurus OX=30522 GN=MCF2L2 PE=4 SV=1

Observed	Mr(expt)	Mr(calc)	ppm	Start	End	Miss	Ions	Peptide
811.4696	810.4623	810.4309	38.7	759	764	0	---	K.YQMLLK.G + Oxidation (M)
853.4834	852.4761	852.4568	22.7	1	6	0	---	-.MLFWLK.E + Oxidation (M)
882.5262	881.5189	881.4970	24.8	419	425	1	---	K.KHDILEK.S
882.5263	881.5190	881.4970	24.9	419	425	1	---	K.KHDILEK.S
1045.5144	1044.5071	1044.5134	-6.05	748	755	1	---	K.CLREPSQR.L
1045.5144	1044.5071	1044.5134	-6.05	748	755	1	---	K.CLREPSQR.L
1133.6655	1132.6582	1132.6353	20.3	48	58	1	---	R.QFAILSGGRGK.D
1165.6895	1164.6822	1164.6940	-10.17	756	764	1	---	R.LIKYQMLLK.G + Oxidation (M)
1165.6895	1164.6822	1164.6940	-10.12	756	764	1	---	R.LIKYQMLLK.G + Oxidation (M)
1179.5537	1178.5464	1178.5303	13.7	580	589	1	---	K.EDDETKSEVK.N
1307.6320	1306.6247	1306.7245	-76.33	1007	1017	1	---	K.KETSVIYKPSR.I
1334.8311	1333.8238	1333.7354	66.3	552	563	0	---	K.ISQPSTLVPHQK.T
1334.8311	1333.8238	1333.7354	66.3	552	563	0	---	K.ISQPSTLVPHQK.T
1940.8547	1939.8474	1939.9502	-52.98	2	16	1	---	M.LFWLKEMAPQEFTR.R + Oxidation (M)
2056.9478	2055.9405	2056.0166	-37.02	664	679	1	---	K.EFLFGNIRELYEFHSR.T
2221.0344	2220.0271	2219.9187	48.8	721	737	0	---	R.AIWQECQDCTYFGVCQR.Q
2248.9812	2247.9739	2248.1865	-94.54	193	211	1	---	R.HSQWINHRTAIENFALALK.T
2321.0693	2320.0620	2320.2541	-82.77	634	652	1	---	K.EIKNIIDGYIIPMDFIWLK.H

No match to: 842.4688, 848.4903, 850.0149, 850.5021, 856.4824, 864.4868, 865.9890, 868.5110, 870.5007, 876.9973, 892.9677, 951.5844, 953.5057, 996.5507, 1017.6264, 1017.6264, 1033.6548, 1033.6548, 1051.6622, 1051.6622, 1131.6659, 1147.6840, 1151.6809, 1236.6508, 1300.7965, 1302.8025, 1316.8201, 1408.5692, 1408.5692, 1427.7416, 1430.5653, 1448.8605, 1448.8605, 1457.6237, 1475.6832, 1617.9929, 1617.9929, 1732.0273, 1775.7623, 1901.1764, 1993.8938, 2193.0378, 2211.0134, 2225.0469, 2230.1272, 2233.0085, 2239.0554, 2261.0361, 2265.0583, 2283.0833, 2305.0723, 2459.1550, 2470.2014, 2477.1138, 2477.1138, 2492.1714, 2499.1167, 3337.7234

17. A0A4W2F647 **Mass:** 127627 **Score:** 36 **Expect:** 36 **Matches:** 16
SRC kinase signaling inhibitor 1 OS=Bos indicus x Bos taurus OX=30522 GN=SRCIN1 PE=4 SV=1

Observed	Mr(expt)	Mr(calc)	ppm	Start	End	Miss	Ions	Peptide
848.4903	847.4830	847.4586	28.9	10	16	0	---	R.LLSTVCR.K
853.4834	852.4761	852.4705	6.59	501	508	0	---	R.LPPSSPQK.L
864.4868	863.4795	863.3998	92.3	777	783	0	---	R.DVSHNHR.L
953.5057	952.4984	952.4548	45.8	1158	1164	1	---	K.QRAEYMR.I
1033.6548	1032.6475	1032.5465	97.9	533	541	1	---	R.GSPVRQSFR.K
1033.6548	1032.6475	1032.5465	97.9	533	541	1	---	R.GSPVRQSFR.K
1131.6659	1130.6586	1130.6183	35.7	799	808	0	---	K.QLGETLTELK.A
1300.7965	1299.7892	1299.6783	85.4	1140	1152	1	---	R.LKTAQGQAGSPDK.G
1408.5692	1407.5619	1407.6664	-74.20	932	944	1	---	R.SMDKAVSVEAAER.D + Oxidation (M)
1408.5692	1407.5619	1407.6664	-74.19	932	944	1	---	R.SMDKAVSVEAAER.D + Oxidation (M)
1427.7416	1426.7343	1426.7780	-30.58	261	273	1	---	K.SPNTAILIKDEAR.N
1457.6237	1456.6164	1456.6735	-39.21	486	500	0	---	K.AAGGGPLYGDGYGFR.L
1775.7623	1774.7550	1774.7904	-19.94	632	650	0	---	K.IEGSNGAATPSAPCGSGSR.S
2305.0723	2304.0650	2304.2181	-66.43	601	621	1	---	R.MEAMEKQIASLTGLVQSALLR.G + Oxidation (M)
2321.0693	2320.0620	2320.2130	-65.08	601	621	1	---	R.MEAMEKQIASLTGLVQSALLR.G + 2 Oxidation (M)
2459.1550	2458.1477	2458.1169	12.5	1109	1131	1	---	K.DEDDEDRIIAELESGGGSVPPMK.V

No match to: 811.4696, 842.4688, 850.0149, 850.5021, 856.4824, 865.9890, 868.5110, 870.5007, 876.9973, 882.5262, 882.5263, 892.9677, 951.5844, 996.5507, 1017.6264, 1017.6264, 1045.5144, 1045.5144, 1051.6622, 1051.6622, 1133.6655, 1147.6840, 1151.6809, 1165.6895, 1165.6895, 1179.5537, 1236.6508, 1302.8025, 1307.6320, 1316.8201, 1334.8311, 1334.8311, 1430.5653, 1448.8605, 1448.8605, 1475.6832, 1617.9929, 1617.9929, 1732.0273, 1901.1764, 1940.8547, 1993.8938, 2056.9478, 2193.0378, 2211.0134, 2221.0344, 2225.0469, 2230.1272, 2233.0085, 2239.0554, 2248.9812, 2261.0361, 2265.0583, 2283.0833, 2470.2014, 2477.1138, 2477.1138, 2492.1714, 2499.1167, 3337.7234

18. A0A4W2DZ95 **Mass:** 132512 **Score:** 35 **Expect:** 39 **Matches:** 16
SRC kinase signaling inhibitor 1 OS=Bos indicus x Bos taurus OX=30522 GN=SRCIN1 PE=4 SV=1

Observed	Mr(expt)	Mr(calc)	ppm	Start	End	Miss	Ions	Peptide
853.4834	852.4761	852.4705	6.59	520	527	0	---	R.LPPSSPQK.L
864.4868	863.4795	863.3998	92.3	796	802	0	---	R.DVSHNHR.L
953.5057	952.4984	952.4548	45.8	1177	1183	1	---	K.QRAEYMR.I
1033.6548	1032.6475	1032.5465	97.9	552	560	1	---	R.GSPVRQSFR.K
1033.6548	1032.6475	1032.5465	97.9	552	560	1	---	R.GSPVRQSFR.K
1131.6659	1130.6586	1130.6183	35.7	818	827	0	---	K.QLGETLTELK.A
1300.7965	1299.7892	1299.6783	85.4	1159	1171	1	---	R.LKTAQGQAGSPDK.G
1408.5692	1407.5619	1407.6664	-74.20	951	963	1	---	R.SMDKAVSVEAAER.D + Oxidation (M)
1408.5692	1407.5619	1407.6664	-74.19	951	963	1	---	R.SMDKAVSVEAAER.D + Oxidation (M)
1427.7416	1426.7343	1426.7780	-30.58	280	292	1	---	K.SPNTAILIKDEAR.N
1457.6237	1456.6164	1456.6735	-39.21	505	519	0	---	K.AAGGGPLYGDGYGFR.L
1775.7623	1774.7550	1774.7904	-19.94	651	669	0	---	K.IEGSNGAATPSAPCGSGSR.S
1993.8938	1992.8865	1993.0051	-59.48	1179	1195	1	---	R.AEYMRIQAQQQATKPSK.E + Oxidation (M)
2305.0723	2304.0650	2304.2181	-66.43	620	640	1	---	R.MEAMEKQIASLTGLVQSALLR.G + Oxidation (M)

PDF-XChange Click to buy NOW! www.docu-track.com

2321.0693	2320.0620	2320.2130	-65.08	620 -	640	1	---	R.MEAMEKQIASLTGLVQSALLR.G + 2 Oxidation (M)
2459.1550	2458.1477	2458.1169	12.5	1128 -	1150	1	---	K.DEDDEDRIIAELESGGGSVPPMK.V

No match to: 811.4696, 842.4688, 848.4903, 850.0149, 850.5021, 856.4824, 865.9890, 868.5110, 870.5007, 876.9973, 882.5262, 882.5263, 892.9677, 951.5844, 996.5507, 1017.6264, 1017.6264, 1045.5144, 1045.5144, 1051.6622, 1051.6622, 1133.6655, 1147.6840, 1151.6809, 1165.6895, 1165.6895, 1179.5537, 1236.6508, 1302.8025, 1307.6320, 1316.8201, 1334.8311, 1334.8311, 1430.5653, 1448.8605, 1448.8605, 1475.6832, 1617.9929, 1617.9929, 1732.0273, 1901.1764, 1940.8547, 2056.9478, 2193.0378, 2211.0134, 2221.0344, 2225.0469, 2230.1272, 2233.0085, 2239.0554, 2248.9812, 2261.0361, 2265.0583, 2283.0833, 2470.2014, 2477.1138, 2477.1138, 2492.1714, 2499.1167, 3337.7234

19. A0A4W2CC31 **Mass:** 172499 **Score:** 35 **Expect:** 40 **Matches:** 17
Calmodulin regulated spectrin associated protein 1 OS=Bos indicus x Bos taurus OX=30522 GN=CAMSAP1 PE=3 SV=1

Observed	Mr(expt)	Mr(calc)	ppm	Start	End	Miss	Ions	Peptide
842.4688	841.4615	841.5134	-61.61	882 -	888	1	---	R.QRLQLGK.A
864.4868	863.4795	863.4613	21.1	1302 -	1307	1	---	K.QEYLRR.K
870.5007	869.4934	869.4243	79.5	755 -	761	1	---	K.VKEHDDK.D
1045.5144	1044.5071	1044.5451	-36.38	342 -	350	1	---	R.DVQELKDAK.S
1045.5144	1044.5071	1044.5451	-36.38	342 -	350	1	---	R.DVQELKDAK.S
1151.6809	1150.6736	1150.5843	77.6	602 -	612	1	---	R.SGDGHQPLGRK.K
1165.6895	1164.6822	1164.5775	89.9	1179 -	1188	0	---	K.ENVPLDEPPR.S
1165.6895	1164.6822	1164.5775	89.9	1179 -	1188	0	---	K.ENVPLDEPPR.S
1179.5537	1178.5464	1178.5905	-37.37	601 -	611	1	---	R.RSGDGHQPLGR.K
1302.8025	1301.7952	1301.7078	67.1	1191 -	1202	0	---	K.ASLIEVDLSDLK.A
1993.8938	1992.8865	1993.0341	-74.05	98 -	116	0	---	K.GDQVASLQGHQSVIQALSR.K
2193.0378	2192.0305	2192.1048	-33.90	1532 -	1550	0	---	K.TMSVSVDALTIHNHLWQPK.R + Oxidation (M)
2305.0723	2304.0650	2304.2188	-66.72	561 -	580	1	---	K.TDSFFLEPLMPAVLRPAKEK.Q + Oxidation (M)
2459.1550	2458.1477	2458.1711	-9.50	1191 -	1213	1	---	K.ASLIEVDLSDLKAPDEDGETEGR.D
2477.1138	2476.1065	2476.2420	-54.72	1458 -	1477	1	---	K.NSILEELEKCDANHYIILFR.D
2477.1138	2476.1065	2476.2420	-54.71	1458 -	1477	1	---	K.NSILEELEKCDANHYIILFR.D
2492.1714	2491.1641	2491.2707	-42.77	370 -	394	1	---	K.RSFLGSPAAASPADLQLGEPEPPGK.G

No match to: 811.4696, 848.4903, 850.0149, 850.5021, 853.4834, 856.4824, 865.9890, 868.5110, 876.9973, 882.5262, 882.5263, 892.9677, 951.5844, 953.5057, 996.5507, 1017.6264, 1017.6264, 1033.6548, 1033.6548, 1051.6622, 1131.6659, 1133.6655, 1147.6840, 1236.6508, 1300.7965, 1307.6320, 1316.8201, 1334.8311, 1334.8311, 1408.5692, 1408.5692, 1427.7416, 1430.5653, 1448.8605, 1448.8605, 1457.6237, 1475.6832, 1617.9929, 1617.9929, 1732.0273, 1775.7623, 1901.1764, 1940.8547, 2056.9478, 2211.0134, 2221.0344, 2225.0469, 2230.1272, 2233.0085, 2239.0554, 2248.9812, 2261.0361, 2265.0583, 2283.0833, 2321.0693, 2470.2014, 2499.1167, 3337.7234

20. A0A3Q1N0Q9 **Mass:** 136628 **Score:** 35 **Expect:** 43 **Matches:** 16
SRC kinase signaling inhibitor 1 OS=Bos taurus OX=9913 GN=SRCIN1 PE=4 SV=1

Observed	Mr(expt)	Mr(calc)	ppm	Start	End	Miss	Ions	Peptide
853.4834	852.4761	852.4705	6.59	554 -	561	0	---	R.LPPSSPQK.L
864.4868	863.4795	863.3998	92.3	830 -	836	0	---	R.DVSHNHR.L
953.5057	952.4984	952.4548	45.8	1211 -	1217	1	---	K.QRAEYMR.I
1033.6548	1032.6475	1032.5465	97.9	586 -	594	1	---	R.GSPVRQSFR.K
1033.6548	1032.6475	1032.5465	97.9	586 -	594	1	---	R.GSPVRQSFR.K
1131.6659	1130.6586	1130.6183	35.7	852 -	861	0	---	K.QLGETLTELK.A
1300.7965	1299.7892	1299.6783	85.4	1193 -	1205	1	---	R.LKTAQGQAGSPDK.G
1408.5692	1407.5619	1407.6664	-74.20	985 -	997	1	---	R.SMDKAVSVEAAER.D + Oxidation (M)
1408.5692	1407.5619	1407.6664	-74.19	985 -	997	1	---	R.SMDKAVSVEAAER.D + Oxidation (M)
1427.7416	1426.7343	1426.7780	-30.58	314 -	326	1	---	K.SPNTAILIKDEAR.N
1457.6237	1456.6164	1456.6735	-39.21	539 -	553	0	---	K.AAGGGPLYGDGYGFR.L
1775.7623	1774.7550	1774.7904	-19.94	685 -	703	0	---	K.IEGSNGAATPSAPCGSGSR.S
1993.8938	1992.8865	1993.0051	-59.48	1213 -	1229	1	---	R.AEYMRIQAQQQATKPSK.E + Oxidation (M)
2305.0723	2304.0650	2304.2181	-66.43	654 -	674	1	---	R.MEAMEKQIASLTGLVQSALLR.G + Oxidation (M)
2321.0693	2320.0620	2320.2130	-65.08	654 -	674	1	---	R.MEAMEKQIASLTGLVQSALLR.G + 2 Oxidation (M)
2459.1550	2458.1477	2458.1169	12.5	1162 -	1184	1	---	K.DEDDEDRIIAELESGGGSVPPMK.V

No match to: 811.4696, 842.4688, 848.4903, 850.0149, 850.5021, 856.4824, 865.9890, 868.5110, 870.5007, 876.9973, 882.5262, 882.5263, 892.9677, 951.5844, 996.5507, 1017.6264, 1017.6264, 1045.5144, 1045.5144, 1051.6622, 1051.6622, 1133.6655, 1147.6840, 1151.6809, 1165.6895, 1165.6895, 1179.5537, 1236.6508, 1302.8025, 1307.6320, 1316.8201, 1334.8311, 1334.8311, 1430.5653, 1448.8605, 1448.8605, 1475.6832, 1617.9929, 1617.9929, 1732.0273, 1901.1764, 1940.8547, 2056.9478, 2193.0378, 2211.0134, 2221.0344, 2225.0469, 2230.1272, 2233.0085, 2239.0554, 2248.9812, 2261.0361, 2265.0583, 2283.0833, 2470.2014, 2477.1138, 2477.1138, 2492.1714, 2499.1167, 3337.7234

21. A0A4W2GMF0 **Mass:** 148991 **Score:** 35 **Expect:** 47 **Matches:** 16
Coiled-coil domain containing 171 OS=Bos indicus x Bos taurus OX=30522 GN=CCDC171 PE=4 SV=1

Observed	Mr(expt)	Mr(calc)	ppm	Start	End	Miss	Ions	Peptide
864.4868	863.4795	863.4389	47.1	650 -	656	0	---	K.FQEIAEK.N
996.5507	995.5434	995.5189	24.7	940 -	947	0	---	K.QIFGFTQR.L
1045.5144	1044.5071	1044.4447	59.8	620 -	627	0	---	K.AQESCWHK.Q
1045.5144	1044.5071	1044.4447	59.8	620 -	627	0	---	K.AQESCWHK.Q
1179.5537	1178.5464	1178.5965	-42.47	1055 -	1064	1	---	K.SLSEAKMELR.R + Oxidation (M)
1236.6508	1235.6435	1235.6180	20.7	18 -	28	1	---	K.NASSDIKQMVK.S + Oxidation (M)
1475.6832	1474.6759	1474.7337	-39.18	277 -	290	0	---	K.ASQAEAVADLEMIK.N
1617.9929	1616.9856	1616.8444	87.4	798 -	812	1	---	R.EGIGVLVCTGELKDK.H
1617.9929	1616.9856	1616.8444	87.4	798 -	812	1	---	R.EGIGVLVCTGELKDK.H
1732.0273	1731.0200	1730.8547	95.5	2 -	17	1	---	M.NSNTSSNTPGDIQRLK.N
1993.8938	1992.8865	1992.9826	-48.20	277 -	294	1	---	K.ASQAEAVADLEMIKNEFK.E
2221.0344	2220.0271	2220.1790	-68.41	718 -	735	1	---	R.DFLQEQVNTFELLKLEVR.T
2261.0361	2260.0288	2260.0794	-22.36	157 -	174	1	---	R.DNIIQNCHQEYDLLTKEK.S
2477.1138	2476.1065	2476.2494	-57.71	688 -	709	1	---	R.EKTSLLVACALMAGAFYPLYSR.S + Oxidation (M)
2477.1138	2476.1065	2476.2494	-57.70	688 -	709	1	---	R.EKTSLLVACALMAGAFYPLYSR.S + Oxidation (M)
2492.1714	2491.1641	2491.2476	-33.49	1259 -	1283	0	---	K.ETLINSVPLASSSNSSPVTMSATAK.R

No match to: 811.4696, 842.4688, 848.4903, 850.0149, 850.5021, 853.4834, 856.4824, 865.9890, 868.5110, 870.5007, 876.9973, 882.5262, 882.5263, 892.9677, 951.5844, 953.5057, 1017.6264, 1017.6264, 1033.6548, 1033.6548, 1051.6622, 1051.6622, 1131.6659, 1133.6655, 1147.6840, 1151.6809, 1165.6895, 1165.6895, 1300.7965, 1302.8025, 1307.6320, 1316.8201, 1334.8311, 1334.8311, 1408.5692, 1408.5692, 1427.7416, 1430.5653, 1448.8605, 1448.8605, 1457.6237, 1775.7623, 1901.1764, 1940.8547, 2056.9478, 2193.0378, 2211.0134, 2225.0469, 2230.1272, 2233.0085, 2239.0554, 2248.9812, 2265.0583, 2283.0833, 2305.0723, 2321.0693, 2459.1550, 2470.2014, 2499.1167, 3337.7234

22. A0A3Q1LVB1 **Mass:** 134506 **Score:** 34 **Expect:** 49 **Matches:** 16
SRC kinase signaling inhibitor 1 OS=Bos taurus OX=9913 GN=SRCIN1 PE=4 SV=1

Observed	Mr(expt)	Mr(calc)	ppm	Start	End	Miss	Ions	Peptide
853.4834	852.4761	852.4705	6.59	533 -	540	0	---	R.LPPSSPQK.L
864.4868	863.4795	863.3998	92.3	809 -	815	0	---	R.DVSHNHR.L
953.5057	952.4984	952.4548	45.8	1190 -	1196	1	---	K.QRAEYMR.I
1033.6548	1032.6475	1032.5465	97.9	565 -	573	1	---	R.GSPVRQSFR.K

PDF-XChange Click to buy NOW! www.docu-track.com

PDF-XChange Click to buy NOW! www.docu-track.com

1033.6548	1032.6475	1032.5465	97.9	565	573	1	---	R.GSPVRQSFR.K
1131.6659	1130.6586	1130.6183	35.7	831	840	0	---	K.QLGETLTELK.A
1300.7965	1299.7892	1299.6783	85.4	1172	1184	1	---	R.LKTAQGQAGSPDK.G
1408.5692	1407.5619	1407.6664	-74.20	964	976	1	---	R.SMDKAVSVEAAER.D + Oxidation (M)
1408.5692	1407.5619	1407.6664	-74.19	964	976	1	---	R.SMDKAVSVEAAER.D + Oxidation (M)
1427.7416	1426.7343	1426.7780	-30.58	293	305	1	---	K.SPNTAILIKDEAR.N
1457.6237	1456.6164	1456.6735	-39.21	518	532	0	---	K.AAGGGPLYGDGYGFR.L
1775.7623	1774.7550	1774.7904	-19.94	664	682	0	---	K.IEGSNGAATPSAPCGSGSR.S
1993.8938	1992.8865	1993.0051	-59.48	1192	1208	1	---	R.AEYMRIQAQQQATKPSK.E + Oxidation (M)
2305.0723	2304.0650	2304.2181	-66.43	633	653	1	---	R.MEAMEKQIASLTGLVQSALLR.G + Oxidation (M)
2321.0693	2320.0620	2320.2130	-65.08	633	653	1	---	R.MEAMEKQIASLTGLVQSALLR.G + 2 Oxidation (M)
2459.1550	2458.1477	2458.1169	12.5	1141	1163	1	---	K.DEDDEDRIIAELESGGGSVPPMK.V

No match to: 811.4696, 842.4688, 848.4903, 850.0149, 850.5021, 856.4824, 865.9890, 868.5110, 870.5007, 876.9973, 882.5262, 882.5263, 892.9677, 951.5844, 996.5507, 1017.6264, 1017.6264, 1045.5144, 1045.5144, 1051.6622, 1051.6622, 1133.6655, 1147.6840, 1151.6809, 1165.6895, 1165.6895, 1179.5537, 1236.6508, 1302.8025, 1307.6320, 1316.8201, 1334.8311, 1334.8311, 1430.5653, 1448.8605, 1448.8605, 1475.6832, 1617.9929, 1617.9929, 1732.0273, 1901.1764, 1940.8547, 2056.9478, 2193.0378, 2211.0134, 2221.0344, 2225.0469, 2230.1272, 2233.0085, 2239.0554, 2248.9812, 2261.0361, 2265.0583, 2283.0833, 2470.2014, 2477.1138, 2477.1138, 2492.1714, 2499.1167, 3337.7234

23. Q80RC9 **Mass:** 50101 **Score:** 34 **Expect:** 50 **Matches:** 9

Bo17 (Fragment) OS=Bovine herpesvirus 4 OX=10385 GN=Bo17 PE=4 SV=1

Observed	Mr(expt)	Mr(calc)	ppm	Start	End	Miss	Ions	Peptide
1475.6832	1474.6759	1474.6973	-14.50	247	259	1	---	K.NSMESEIPPESKK.N
1617.9929	1616.9856	1616.8708	71.0	348	361	1	---	K.YHISDMTAIARLVK.W
1617.9929	1616.9856	1616.8708	71.0	348	361	1	---	K.YHISDMTAIARLVK.W
1940.8547	1939.8474	1940.0214	-89.70	73	90	1	---	R.GDQEAVAQALLDNLEVKK.K
1993.8938	1992.8865	1993.0376	-75.82	171	188	1	---	K.EAVKAIISCLPNVFMASK.L + Oxidation (M)
2225.0469	2224.0396	2224.0259	6.17	280	299	0	---	K.DPPPDNLPMFTGNAYFVASR.A + Oxidation (M)
2233.0085	2232.0012	2232.0555	-24.31	200	218	0	---	R.VQADLNCMEDLLQSSVPWK.Y
2248.9812	2247.9739	2248.0504	-34.02	200	218	0	---	R.VQADLNCMEDLLQSSVPWK.Y + Oxidation (M)
2283.0833	2282.0760	2282.1113	-15.48	92	109	1	---	K.RPPLTDTYYLNITRDCER.F

No match to: 811.4696, 842.4688, 848.4903, 850.0149, 850.5021, 853.4834, 856.4824, 864.4868, 865.9890, 868.5110, 870.5007, 876.9973, 882.5262, 882.5263, 892.9677, 951.5844, 953.5057, 996.5507, 1017.6264, 1017.6264, 1033.6548, 1033.6548, 1045.5144, 1045.5144, 1051.6622, 1051.6622, 1131.6659, 1133.6655, 1147.6840, 1151.6809, 1165.6895, 1165.6895, 1179.5537, 1236.6508, 1300.7965, 1302.8025, 1307.6320, 1316.8201, 1334.8311, 1334.8311, 1408.5692, 1408.5692, 1427.7416, 1430.5653, 1448.8605, 1448.8605, 1457.6237, 1732.0273, 1775.7623, 1901.1764, 2056.9478, 2193.0378, 2211.0134, 2221.0344, 2230.1272, 2239.0554, 2261.0361, 2265.0583, 2305.0723, 2321.0693, 2459.1550, 2470.2014, 2477.1138, 2477.1138, 2492.1714, 2499.1167, 3337.7234

24. Q80RD0 **Mass:** 49973 **Score:** 34 **Expect:** 56 **Matches:** 9

Bo17 (Fragment) OS=Bovine herpesvirus 4 OX=10385 GN=Bo17 PE=4 SV=1

Observed	Mr(expt)	Mr(calc)	ppm	Start	End	Miss	Ions	Peptide
1475.6832	1474.6759	1474.6973	-14.50	246	258	1	---	K.NSMESEIPPESKK.N
1617.9929	1616.9856	1616.8708	71.0	347	360	1	---	K.YHISDMTAIARLVK.W
1617.9929	1616.9856	1616.8708	71.0	347	360	1	---	K.YHISDMTAIARLVK.W
1940.8547	1939.8474	1940.0214	-89.70	72	89	1	---	R.GDQEAVAQALLDNLEVKK.K
1993.8938	1992.8865	1993.0376	-75.82	170	187	1	---	K.EAVKAIISCLPNVFMASK.L + Oxidation (M)
2225.0469	2224.0396	2224.0259	6.17	279	298	0	---	K.DPPPDNLPMFTGNAYFVASR.A + Oxidation (M)
2233.0085	2232.0012	2232.0555	-24.31	199	217	0	---	R.VQADLNCMEDLLQSSVPWK.Y
2248.9812	2247.9739	2248.0504	-34.02	199	217	0	---	R.VQADLNCMEDLLQSSVPWK.Y + Oxidation (M)
2283.0833	2282.0760	2282.1113	-15.48	91	108	1	---	K.RPPLTDTYYLNITRDCER.F

No match to: 811.4696, 842.4688, 848.4903, 850.0149, 850.5021, 853.4834, 856.4824, 864.4868, 865.9890, 868.5110, 870.5007, 876.9973, 882.5262, 882.5263, 892.9677, 951.5844, 953.5057, 996.5507, 1017.6264, 1017.6264, 1033.6548, 1033.6548, 1045.5144, 1045.5144, 1051.6622, 1051.6622, 1131.6659, 1133.6655, 1147.6840, 1151.6809, 1165.6895, 1165.6895, 1179.5537, 1236.6508, 1300.7965, 1302.8025, 1307.6320, 1316.8201, 1334.8311, 1334.8311, 1408.5692, 1408.5692, 1427.7416, 1430.5653, 1448.8605, 1448.8605, 1457.6237, 1732.0273, 1775.7623, 1901.1764, 2056.9478, 2193.0378, 2211.0134, 2221.0344, 2230.1272, 2239.0554, 2261.0361, 2265.0583, 2305.0723, 2321.0693, 2459.1550, 2470.2014, 2477.1138, 2477.1138, 2492.1714, 2499.1167, 3337.7234

25. A0A4W2IBE1 **Mass:** 60650 **Score:** 34 **Expect:** 57 **Matches:** 6

RIC8 guanine nucleotide exchange factor B OS=Bos indicus x Bos taurus OX=30522 GN=RIC8B PE=4 SV=1

Observed	Mr(expt)	Mr(calc)	ppm	Start	End	Miss	Ions	Peptide
848.4903	847.4830	847.4473	42.1	515	521	0	---	K.LVNMLDK.L + Oxidation (M)
856.4824	855.4751	855.5430	-79.30	79	86	0	---	K.VLVPVTTK.E
1775.7623	1774.7550	1774.8784	-69.53	247	261	1	---	K.ESESHQFRVMAAVLR.H + Oxidation (M)
2225.0469	2224.0396	2224.1158	-34.24	356	375	1	---	K.GSSYREGLTPVLSLLTECSR.A
2477.1138	2476.1065	2476.2584	-61.36	98	119	1	17	K.LNESDDSLEKVSEFPVIVESLK.C
2477.1138	2476.1065	2476.2584	-61.35	98	119	1	---	K.LNESDDSLEKVSEFPVIVESLK.C

No match to: 811.4696, 842.4688, 850.0149, 850.5021, 853.4834, 864.4868, 865.9890, 868.5110, 870.5007, 876.9973, 882.5262, 882.5263, 892.9677, 951.5844, 953.5057, 996.5507, 1017.6264, 1017.6264, 1033.6548, 1033.6548, 1045.5144, 1045.5144, 1051.6622, 1051.6622, 1131.6659, 1133.6655, 1147.6840, 1151.6809, 1165.6895, 1165.6895, 1179.5537, 1236.6508, 1300.7965, 1302.8025, 1307.6320, 1316.8201, 1334.8311, 1334.8311, 1408.5692, 1408.5692, 1427.7416, 1430.5653, 1448.8605, 1448.8605, 1457.6237, 1475.6832, 1617.9929, 1617.9929, 1732.0273, 1901.1764, 1940.8547, 1993.8938, 2056.9478, 2193.0378, 2211.0134, 2221.0344, 2230.1272, 2233.0085, 2239.0554, 2248.9812, 2261.0361, 2265.0583, 2283.0833, 2305.0723, 2321.0693, 2459.1550, 2470.2014, 2492.1714, 2499.1167, 3337.7234

26. A0A4W2GMI1 **Mass:** 61548 **Score:** 33 **Expect:** 61 **Matches:** 6

RIC8 guanine nucleotide exchange factor B OS=Bos indicus x Bos taurus OX=30522 GN=RIC8B PE=4 SV=1

Observed	Mr(expt)	Mr(calc)	ppm	Start	End	Miss	Ions	Peptide
848.4903	847.4830	847.4473	42.1	515	521	0	---	K.LVNMLDK.L + Oxidation (M)
856.4824	855.4751	855.5430	-79.30	79	86	0	---	K.VLVPVTTK.E
1775.7623	1774.7550	1774.8784	-69.53	247	261	1	---	K.ESESHQFRVMAAVLR.H + Oxidation (M)
2225.0469	2224.0396	2224.1158	-34.24	356	375	1	---	K.GSSYREGLTPVLSLLTECSR.A
2477.1138	2476.1065	2476.2584	-61.36	98	119	1	17	K.LNESDDSLEKVSEFPVIVESLK.C
2477.1138	2476.1065	2476.2584	-61.35	98	119	1	---	K.LNESDDSLEKVSEFPVIVESLK.C

No match to: 811.4696, 842.4688, 850.0149, 850.5021, 853.4834, 864.4868, 865.9890, 868.5110, 870.5007, 876.9973, 882.5262, 882.5263, 892.9677, 951.5844, 953.5057, 996.5507, 1017.6264, 1017.6264, 1033.6548, 1033.6548, 1045.5144, 1045.5144, 1051.6622, 1051.6622, 1131.6659, 1133.6655, 1147.6840, 1151.6809, 1165.6895, 1165.6895, 1179.5537, 1236.6508, 1300.7965, 1302.8025, 1307.6320, 1316.8201, 1334.8311, 1334.8311, 1408.5692, 1408.5692, 1427.7416, 1430.5653, 1448.8605, 1448.8605, 1457.6237, 1475.6832, 1617.9929, 1617.9929, 1732.0273, 1901.1764, 1940.8547, 1993.8938, 2056.9478, 2193.0378, 2211.0134, 2221.0344, 2230.1272, 2233.0085, 2239.0554, 2248.9812, 2261.0361, 2265.0583, 2283.0833, 2305.0723, 2321.0693, 2459.1550, 2470.2014, 2492.1714, 2499.1167, 3337.7234

27. E1BD35 **Mass:** 61548 **Score:** 33 **Expect:** 61 **Matches:** 6

RIC8 guanine nucleotide exchange factor B OS=Bos taurus OX=9913 GN=RIC8B PE=4 SV=3

Observed	Mr(expt)	Mr(calc)	ppm	Start	End	Miss	Ions	Peptide
848.4903	847.4830	847.4473	42.1	515	521	0	---	K.LVNMLDK.L + Oxidation (M)
856.4824	855.4751	855.5430	-79.30	79	86	0	---	K.VLVPVTTK.E

PDF-XChange Click to buy NOW! www.docu-track.com

PDF-XChange Click to buy NOW! www.docu-track.com

1775.7623	1774.7550	1774.8784	-69.53	247	261	1	---	K.ESESHQFRVMAAVLR.H + Oxidation (M)
2225.0469	2224.0396	2224.1158	-34.24	356	375	1	---	K.GSSYREGLTPVLSLLTECSR.A
2477.1138	2476.1065	2476.2584	-61.36	98	119	1	17	K.LNESDDSLEKVSEFPVIVESLK.C
2477.1138	2476.1065	2476.2584	-61.35	98	119	1	---	K.LNESDDSLEKVSEFPVIVESLK.C

No match to: 811.4696, 842.4688, 850.0149, 850.5021, 853.4834, 864.4868, 865.9890, 868.5110, 870.5007, 876.9973, 882.5262, 882.5263, 892.9677, 951.5844, 953.5057, 996.5507, 1017.6264, 1017.6264, 1033.6548, 1033.6548, 1045.5144, 1045.5144, 1051.6622, 1051.6622, 1131.6659, 1133.6655, 1147.6840, 1151.6809, 1165.6895, 1165.6895, 1179.5537, 1236.6508, 1300.7965, 1302.8025, 1307.6320, 1316.8201, 1334.8311, 1334.8311, 1408.5692, 1408.5692, 1427.7416, 1430.5653, 1448.8605, 1448.8605, 1457.6237, 1475.6832, 1617.9929, 1617.9929, 1732.0273, 1901.1764, 1940.8547, 1993.8938, 2056.9478, 2193.0378, 2211.0134, 2221.0344, 2230.1272, 2233.0085, 2239.0554, 2248.9812, 2261.0361, 2265.0583, 2283.0833, 2305.0723, 2321.0693, 2459.1550, 2470.2014, 2492.1714, 2499.1167, 3337.7234

28. A0A3Q1M3W0 **Mass:** 173871 **Score:** 33 **Expect:** 63 **Matches:** 17

Calmodulin regulated spectrin associated protein 1 OS=Bos taurus OX=9913 GN=CAMSAP1 PE=3 SV=1

Observed	Mr(expt)	Mr(calc)	ppm	Start	End	Miss	Ions	Peptide
842.4688	841.4615	841.5134	-61.61	893	899	1	---	R.QRLQLGK.A
864.4868	863.4795	863.4613	21.1	1313	1318	1	---	K.QEYLRR.K
870.5007	869.4934	869.4243	79.5	766	772	1	---	K.VKEHDDK.D
1045.5144	1044.5071	1044.5451	-36.38	353	361	1	---	R.DVQELKDAK.S
1045.5144	1044.5071	1044.5451	-36.38	353	361	1	---	R.DVQELKDAK.S
1151.6809	1150.6736	1150.5843	77.6	613	623	1	---	R.SGDGHQPLGRK.K
1165.6895	1164.6822	1164.5775	89.9	1190	1199	0	---	K.ENVPLDEPPR.S
1165.6895	1164.6822	1164.5775	89.9	1190	1199	0	---	K.ENVPLDEPPR.S
1179.5537	1178.5464	1178.5905	-37.37	612	622	1	---	R.RSGDGHQPLGR.K
1302.8025	1301.7952	1301.7078	67.1	1202	1213	0	---	K.ASLIEVDLSDLK.A
1993.8938	1992.8865	1993.0341	-74.05	98	116	0	---	K.GDQVASLQGHQSVIQALSR.K
2193.0378	2192.0305	2192.1048	-33.90	1543	1561	0	---	K.TMSVSVDALTIHNHLWQPK.R + Oxidation (M)
2305.0723	2304.0650	2304.2188	-66.72	572	591	1	---	K.TDSFFLEPLMPAVLRPAKEK.Q + Oxidation (M)
2459.1550	2458.1477	2458.1711	-9.50	1202	1224	1	---	K.ASLIEVDLSDLKAPDEDGETEGR.D
2477.1138	2476.1065	2476.2420	-54.72	1469	1488	1	---	K.NSILEELEKCDANHYIILFR.D
2477.1138	2476.1065	2476.2420	-54.71	1469	1488	1	---	K.NSILEELEKCDANHYIILFR.D
2492.1714	2491.1641	2491.2707	-42.77	381	405	1	---	K.RSFLGSPAAASPADLQLGEPEPPGK.G

No match to: 811.4696, 848.4903, 850.0149, 850.5021, 853.4834, 856.4824, 865.9890, 868.5110, 876.9973, 882.5262, 882.5263, 892.9677, 951.5844, 953.5057, 996.5507, 1017.6264, 1017.6264, 1033.6548, 1033.6548, 1051.6622, 1051.6622, 1131.6659, 1133.6655, 1147.6840, 1236.6508, 1300.7965, 1307.6320, 1316.8201, 1334.8311, 1334.8311, 1408.5692, 1408.5692, 1427.7416, 1430.5653, 1448.8605, 1448.8605, 1457.6237, 1475.6832, 1617.9929, 1617.9929, 1732.0273, 1775.7623, 1901.1764, 1940.8547, 2056.9478, 2211.0134, 2221.0344, 2225.0469, 2230.1272, 2233.0085, 2239.0554, 2248.9812, 2261.0361, 2265.0583, 2283.0833, 2321.0693, 2470.2014, 2499.1167, 3337.7234

29. A0A4W2IDJ5 **Mass:** 62224 **Score:** 33 **Expect:** 64 **Matches:** 6

RIC8 guanine nucleotide exchange factor B OS=Bos indicus x Bos taurus OX=30522 GN=RIC8B PE=4 SV=1

Observed	Mr(expt)	Mr(calc)	ppm	Start	End	Miss	Ions	Peptide
848.4903	847.4830	847.4473	42.1	499	505	0	---	K.LVNMLDK.L + Oxidation (M)
856.4824	855.4751	855.5430	-79.30	63	70	0	---	K.VLVPVTTK.E
1775.7623	1774.7550	1774.8784	-69.53	231	245	1	---	K.ESESHQFRVMAAVLR.H + Oxidation (M)
2225.0469	2224.0396	2224.1158	-34.24	340	359	1	---	K.GSSYREGLTPVLSLLTECSR.A
2477.1138	2476.1065	2476.2584	-61.36	82	103	1	17	K.LNESDDSLEKVSEFPVIVESLK.C
2477.1138	2476.1065	2476.2584	-61.35	82	103	1	---	K.LNESDDSLEKVSEFPVIVESLK.C

No match to: 811.4696, 842.4688, 850.0149, 850.5021, 853.4834, 864.4868, 865.9890, 868.5110, 870.5007, 876.9973, 882.5262, 882.5263, 892.9677, 951.5844, 953.5057, 996.5507, 1017.6264, 1017.6264, 1033.6548, 1033.6548, 1045.5144, 1045.5144, 1051.6622, 1051.6622, 1131.6659, 1133.6655, 1147.6840, 1151.6809, 1165.6895, 1165.6895, 1179.5537, 1236.6508, 1300.7965, 1302.8025, 1307.6320, 1316.8201, 1334.8311, 1334.8311, 1408.5692, 1408.5692, 1427.7416, 1430.5653, 1448.8605, 1448.8605, 1457.6237, 1475.6832, 1617.9929, 1617.9929, 1732.0273, 1901.1764, 1940.8547, 1993.8938, 2056.9478, 2193.0378, 2211.0134, 2221.0344, 2230.1272, 2233.0085, 2239.0554, 2248.9812, 2261.0361, 2265.0583, 2283.0833, 2305.0723, 2321.0693, 2459.1550, 2470.2014, 2492.1714, 2499.1167, 3337.7234

30. A0A4W2F4T9 **Mass:** 128155 **Score:** 33 **Expect:** 66 **Matches:** 15

SRC kinase signaling inhibitor 1 OS=Bos indicus x Bos taurus OX=30522 GN=SRCIN1 PE=4 SV=1

Observed	Mr(expt)	Mr(calc)	ppm	Start	End	Miss	Ions	Peptide
853.4834	852.4761	852.4705	6.59	512	519	0	---	R.LPPSSPQK.L
864.4868	863.4795	863.3998	92.3	788	794	0	---	R.DVSHNHR.L
953.5057	952.4984	952.4548	45.8	1169	1175	1	---	K.QRAEYMR.I
1033.6548	1032.6475	1032.5465	97.9	544	552	1	---	R.GSPVRQSFR.K
1033.6548	1032.6475	1032.5465	97.9	544	552	1	---	R.GSPVRQSFR.K
1131.6659	1130.6586	1130.6183	35.7	810	819	0	---	K.QLGETLTELK.A
1300.7965	1299.7892	1299.6783	85.4	1151	1163	1	---	R.LKTAQGQAGSPDK.G
1408.5692	1407.5619	1407.6664	-74.20	943	955	1	---	R.SMDKAVSVEAAER.D + Oxidation (M)
1408.5692	1407.5619	1407.6664	-74.19	943	955	1	---	R.SMDKAVSVEAAER.D + Oxidation (M)
1427.7416	1426.7343	1426.7780	-30.58	272	284	1	---	K.SPNTAILIKDEAR.N
1457.6237	1456.6164	1456.6735	-39.21	497	511	0	---	K.AAGGGPLYGDGYGFR.L
1775.7623	1774.7550	1774.7904	-19.94	643	661	0	---	K.IEGSNGAATPSAPCGSGSR.S
2305.0723	2304.0650	2304.2181	-66.43	612	632	1	---	R.MEAMEKQIASLTGLVQSALLR.G + Oxidation (M)
2321.0693	2320.0620	2320.2130	-65.08	612	632	1	---	R.MEAMEKQIASLTGLVQSALLR.G + 2 Oxidation (M)
2459.1550	2458.1477	2458.1169	12.5	1120	1142	1	---	K.DEDDEDRIIAELESGGGSVPPMK.V

No match to: 811.4696, 842.4688, 848.4903, 850.0149, 850.5021, 856.4824, 865.9890, 868.5110, 870.5007, 876.9973, 882.5262, 882.5263, 892.9677, 951.5844, 996.5507, 1017.6264, 1017.6264, 1045.5144, 1045.5144, 1051.6622, 1051.6622, 1133.6655, 1147.6840, 1151.6809, 1165.6895, 1165.6895, 1179.5537, 1236.6508, 1302.8025, 1307.6320, 1316.8201, 1334.8311, 1334.8311, 1430.5653, 1448.8605, 1448.8605, 1475.6832, 1617.9929, 1617.9929, 1732.0273, 1901.1764, 1940.8547, 1993.8938, 2056.9478, 2193.0378, 2211.0134, 2221.0344, 2225.0469, 2230.1272, 2233.0085, 2239.0554, 2248.9812, 2261.0361, 2265.0583, 2283.0833, 2470.2014, 2477.1138, 2477.1138, 2492.1714, 2499.1167, 3337.7234

Search Parameters

Type of search : Sequence Query
Enzyme : Trypsin
Fixed modifications : Carbamidomethyl (C)
Variable modifications : Oxidation (M)
Mass values : Monoisotopic
Protein Mass : Unrestricted
Peptide Mass Tolerance : ± 100 ppm
Fragment Mass Tolerance: ± 0.3 Da
Max Missed Cleavages : 1
Instrument type : MALDI-TOF-TOF
Query1 (811.4696,1+): <no title>
Query2 (842.4688,1+): <no title>
Query3 (848.4903,1+): <no title>

PDF-XChange Click to buy NOW! www.docu-track.com

```
 Query4 (850.0149,1+): <no title>
 Query5 (850.5021,1+): <no title>
 Query6 (853.4834,1+): <no title>
 Query7 (856.4824,1+): <no title>
 Query8 (864.4868,1+): <no title>
 Query9 (865.9890,1+): <no title>
Query10 (868.5110,1+): <no title>
Query11 (870.5007,1+): <no title>
Query12 (876.9973,1+): <no title>
Query13 (882.5262,1+): <no title>
Query14 (882.5263,1+): Locus:1..14.0.2
Query15 (892.9677,1+): <no title>
Query16 (951.5844,1+): <no title>
Query17 (953.5057,1+): <no title>
Query18 (996.5507,1+): <no title>
Query19 (1017.6264,1+): <no title>
Query20 (1017.6264,1+): Locus:1..14.0.8
Query21 (1033.6548,1+): Locus:1..14.0.10
Query22 (1033.6548,1+): <no title>
Query23 (1045.5144,1+): <no title>
Query24 (1045.5144,1+): Locus:1..14.0.5
Query25 (1051.6622,1+): <no title>
Query26 (1051.6622,1+): Locus:1..14.0.3
Query27 (1131.6659,1+): <no title>
Query28 (1133.6655,1+): <no title>
Query29 (1147.6840,1+): <no title>
Query30 (1151.6809,1+): <no title>
Query31 (1165.6895,1+): Locus:1..14.0.4
Query32 (1165.6895,1+): <no title>
Query33 (1179.5537,1+): <no title>
Query34 (1236.6508,1+): <no title>
Query35 (1300.7965,1+): <no title>
Query36 (1302.8025,1+): <no title>
Query37 (1307.6320,1+): <no title>
Query38 (1316.8201,1+): <no title>
Query39 (1334.8311,1+): Locus:1..14.0.7
Query40 (1334.8311,1+): <no title>
Query41 (1408.5692,1+): <no title>
Query42 (1408.5692,1+): Locus:1..14.0.9
Query43 (1427.7416,1+): <no title>
Query44 (1430.5653,1+): <no title>
Query45 (1448.8605,1+): Locus:1..14.0.11
Query46 (1448.8605,1+): <no title>
Query47 (1457.6237,1+): <no title>
Query48 (1475.6832,1+): <no title>
Query49 (1617.9929,1+): <no title>
Query50 (1617.9929,1+): Locus:1..14.0.6
Query51 (1732.0273,1+): <no title>
Query52 (1775.7623,1+): <no title>
Query53 (1901.1764,1+): <no title>
Query54 (1940.8547,1+): <no title>
Query55 (1993.8938,1+): <no title>
Query56 (2056.9478,1+): <no title>
Query57 (2193.0378,1+): <no title>
Query58 (2211.0134,1+): <no title>
Query59 (2221.0344,1+): <no title>
Query60 (2225.0469,1+): <no title>
Query61 (2230.1272,1+): <no title>
Query62 (2233.0085,1+): <no title>
Query63 (2239.0554,1+): <no title>
Query64 (2248.9812,1+): <no title>
Query65 (2261.0361,1+): <no title>
Query66 (2265.0583,1+): <no title>
Query67 (2283.0833,1+): <no title>
Query68 (2305.0723,1+): <no title>
Query69 (2321.0693,1+): <no title>
Query70 (2459.1550,1+): <no title>
Query71 (2470.2014,1+): <no title>
Query72 (2477.1138,1+): Locus:1..14.0.1
Query73 (2477.1138,1+): <no title>
Query74 (2492.1714,1+): <no title>
Query75 (2499.1167,1+): <no title>
Query76 (3337.7234,1+): <no title>
```

Mascot: http://www.matrixscience.com/
