# Supplementary material for: Alpha-2-Macroglobulin as a New Promising Biomarker Improving the Diagnostic Sensitivity of Bovine Paratuberculosis
Source: Front Vet Sci. 2021 Mar 5;8:637716. doi: 10.3389/fvets.2021.637716 (PMC7973028; doi:10.3389/fvets.2021.637716)
Supplement: Supplementary Data 1 — Mascot search results and raw data files for LC-MS/MS analysis for the identification of specific blood proteins in cattle with Johne's disease. [file Data_Sheet_1.zip › mascot/2 Transthyretin_2.pdf]

PDF-XChange Click to buy NOW! www.docu-track.com

MATRIX SCIENCE MASCOT Search Results

Protein View: A0A4W2BU20

Transthyretin OS=Bos indicus x Bos taurus OX=30522 GN=TTR PE=3 SV=1

Database:	Uniprot_bovine
Score:	88
Expect:	0.00024
Nominal mass (M_r):	15831
Calculated pI:	5.91

Sequence similarity is available as an NCBI BLAST search of A0A4W2BU20 against nr.

Search parameters

Enzyme:	Trypsin: cuts C-term side of KR unless next residue is P.
Fixed modifications:	Carbamidomethyl (C)
Variable modifications:	Oxidation (M)

Protein sequence coverage: 40%

Matched peptides shown in ***bold red***.

```
  1 MASFRLFLLC LAGLVFVSEA GSVGAGEPKC PLMVKVLDAV RGSPAANVGV
 51 KVFKKAADET WEPFASGKTS ESGELHGLTT EDKFVEGLYK VELDTKSYWK
101 SLGISPFHEF AEVVFTANDS GPRHYTIAAL LSPYSYSTTA LVSSPKA
```

Unformatted sequence string: 147 residues (for pasting into other applications).

Sort peptides by ◉ Residue Number ○ Increasing Mass ○ Decreasing Mass

Show predicted peptides also

Query	Start – End	Observed	Mr(expt)	Mr(calc)	ppm	M	Score	Peptide
41	56 – 68	1408.5692	1407.5619	1407.6306	-48.8	0		K.AADETWEPFASGK.T
42	56 – 68	1408.5692	1407.5619	1407.6306	-48.8	0		K.AADETWEPFASGK.T
72	101 – 123	2477.1138	2476.1065	2476.2023	-38.7	0	66	K.SLGISPFHEFAEVVFTANDSGPR.H
73	101 – 123	2477.1138	2476.1065	2476.2023	-38.7	0		K.SLGISPFHEFAEVVFTANDSGPR.H
71	124 – 146	2470.2014	2469.1941	2469.2791	-34.4	0		R.HYTIAALLSPYSYSTTALVSSPK.A

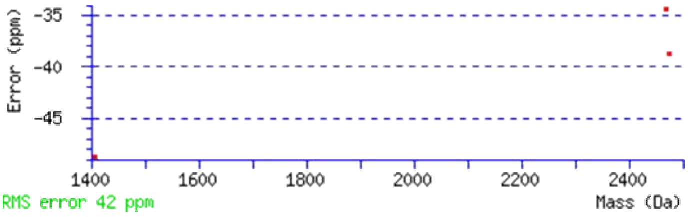


```
ID   A0A4W2BU20_BOBOX        Unreviewed;       147 AA.
AC   A0A4W2BU20;
DT   18-SEP-2019, integrated into UniProtKB/TrEMBL.
DT   18-SEP-2019, sequence version 1.
DT   26-FEB-2020, entry version 5.
DE   RecName: Full=Transthyretin {ECO:0000256|RuleBase:RU361269};
GN   Name=TTR {ECO:0000313|Ensembl:ENSBIXP00000002455};
OS   Bos indicus x Bos taurus (Hybrid cattle).
OC   Eukaryota; Metazoa; Chordata; Craniata; Vertebrata; Euteleostomi; Mammalia;
OC   Eutheria; Laurasiatheria; Artiodactyla; Ruminantia; Pecora; Bovidae;
OC   Bovinae; Bos.
OX   NCBI_TaxID=30522 {ECO:0000313|Ensembl:ENSBIXP00000002455, ECO:0000313|Proteomes:UP000314981};
RN   [1] {ECO:0000313|Ensembl:ENSBIXP00000002455, ECO:0000313|Proteomes:UP000314981}
RP   NUCLEOTIDE SEQUENCE [LARGE SCALE GENOMIC DNA].
RA   Low W.Y., Tearle R., Bickhart D.M., Rosen B.D., Koren S., Rhie A.,
RA   Hiendleder S., Phillippy A.M., Smith T.P.L., Williams J.L.;
RT   "Haplotype-resolved cattle genomes.";
RL   Submitted (NOV-2018) to the EMBL/GenBank/DDBJ databases.
RN   [2] {ECO:0000313|Ensembl:ENSBIXP00000002455}
RP   IDENTIFICATION.
```

PDF-XChange Click to buy NOW! www.docu-track.com

```
[illegible]G   Ensembl;
RL   Submitted (JUL-2019) to UniProtKB.
CC   -!- FUNCTION: Thyroid hormone-binding protein. Probably transports
CC       thyroxine from the bloodstream to the brain.
CC       {ECO:0000256|RuleBase:RU361269}.
CC   -!- SUBUNIT: Homotetramer. {ECO:0000256|RuleBase:RU361269}.
CC   -!- SUBCELLULAR LOCATION: Secreted {ECO:0000256|RuleBase:RU361269}.
CC   -!- SIMILARITY: Belongs to the transthyretin family.
CC       {ECO:0000256|RuleBase:RU361269}.
CC   ---------------------------------------------------------------------------
CC   Copyrighted by the UniProt Consortium, see https://www.uniprot.org/terms
CC   Distributed under the Creative Commons Attribution (CC BY 4.0) License
CC   ---------------------------------------------------------------------------
DR   Ensembl; ENSBIXT00000011885; ENSBIXP00000002455; ENSBIXG00000008920.
DR   Ensembl; ENSBIXT00005046533; ENSBIXP00005035955; ENSBIXG00005017301.
DR   Proteomes; UP000314981; Chromosome 24.
DR   GO; GO:0005576; C:extracellular region; IEA:UniProtKB-SubCell.
DR   GO; GO:0042802; F:identical protein binding; IEA:Ensembl.
DR   GO; GO:0070324; F:thyroid hormone binding; IEA:InterPro.
DR   GO; GO:0042572; P:retinol metabolic process; IEA:InterPro.
DR   GO; GO:0070327; P:thyroid hormone transport; IEA:InterPro.
DR   Gene3D; 2.60.40.180; -; 1.
DR   InterPro; IPR023418; Thyroxine_BS.
DR   InterPro; IPR030178; Transthyretin.
DR   InterPro; IPR000895; Transthyretin/HIU_hydrolase.
DR   InterPro; IPR023416; Transthyretin/HIU_hydrolase_d.
DR   InterPro; IPR036817; Transthyretin/HIU_hydrolase_sf.
DR   InterPro; IPR023419; Transthyretin_CS.
DR   PANTHER; PTHR10395; PTHR10395; 1.
DR   PANTHER; PTHR10395:SF12; PTHR10395:SF12; 1.
DR   Pfam; PF00576; Transthyretin; 1.
DR   PRINTS; PR00189; TRNSTHYRETIN.
DR   SMART; SM00095; TR_THY; 1.
DR   SUPFAM; SSF49472; SSF49472; 1.
DR   PROSITE; PS00768; TRANSTHYRETIN_1; 1.
DR   PROSITE; PS00769; TRANSTHYRETIN_2; 1.
PE   3: Inferred from homology;
KW   Hormone {ECO:0000256|RuleBase:RU361269};
KW   Reference proteome {ECO:0000313|Proteomes:UP000314981};
KW   Secreted {ECO:0000256|RuleBase:RU361269};
KW   Signal {ECO:0000256|RuleBase:RU361269};
KW   Thyroid hormone {ECO:0000256|RuleBase:RU361269}.
FT   SIGNAL          1..20
FT                   /evidence="ECO:0000256|RuleBase:RU361269"
FT   CHAIN           21..147
FT                   /note="Transthyretin"
FT                   /evidence="ECO:0000256|RuleBase:RU361269"
FT                   /id="PRO_5023949286"
FT   DOMAIN          27..147
FT                   /note="TR_THY"
FT                   /evidence="ECO:0000259|SMART:SM00095"
SQ   SEQUENCE   147 AA;  15727 MW;  54C8CA7EC04478B7 CRC64;
     MASFRLFLLC LAGLVFVSEA GSVGAGEPKC PLMVKVLDAV RGSPAANVGV KVFKKAADET
     WEPFASGKTS ESGELHGLTT EDKFVEGLYK VELDTKSYWK SLGISPFHEF AEVVFTANDS
     GPRHYTIAAL LSPYSYSTTA LVSSPKA
//
```

Mascot: http://www.matrixscience.com/
