# Supplementary material for: Alpha-2-Macroglobulin as a New Promising Biomarker Improving the Diagnostic Sensitivity of Bovine Paratuberculosis
Source: Front Vet Sci. 2021 Mar 5;8:637716. doi: 10.3389/fvets.2021.637716 (PMC7973028; doi:10.3389/fvets.2021.637716)
Supplement: Supplementary Data 1 — Mascot search results and raw data files for LC-MS/MS analysis for the identification of specific blood proteins in cattle with Johne's disease. [file Data_Sheet_1.zip › mascot/3 Apolipoprotein A-IV_1.pdf]

PDF-XChange Click to buy NOW! www.docu-track.com

MATRIX SCIENCE Mascot Search Results

```
User          :
Email         :
Search title  : BAEDW\2020_3_5\2020_3_5\MSMS 14\N20
Database      : Uniprot_bovine bovine_20200316 (134392 sequences; 76244912 residues)
Timestamp     : 31 Mar 2020 at 01:09:00 GMT
Warning       : A Peptide summary report will usually give a much clearer picture of MS/MS search results.
Top Score     : 283 for V6F7X3, Apolipoprotein A-IV OS=Bos taurus OX=9913 GN=APOA4 PE=3 SV=1
```

Mascot Score Histogram

Protein score is -10*Log(P), where P is the probability that the observed match is a random event.
Protein scores greater than 64 are significant (p<0.05).
Protein scores are derived from ions scores as a non-probabilistic basis for ranking protein hits.

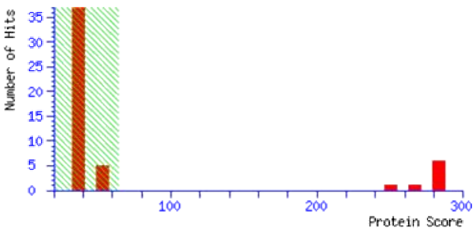

Protein Summary Report

Format As | Protein Summary (deprecated) | Help

Significance threshold p< 0.05 Max. number of hits 30

Preferred taxonomy All entries

Re-Search All | Search Unmatched

Index

	Accession	Mass	Score	Description
1.	V6F7X3	42963	283	Apolipoprotein A-IV OS=Bos taurus OX=9913 GN=APOA4 PE=3 SV=1
2.	A0A4W2CCP9	42963	283	Apolipoprotein A4 OS=Bos indicus x Bos taurus OX=30522 GN=APOA4 PE=3 SV=1
3.	A0A3Q1M3N0	43165	283	Apolipoprotein A-IV OS=Bos taurus OX=9913 GN=APOA4 PE=3 SV=1
4.	A0A3Q1MZJ9	45782	282	Apolipoprotein A-IV OS=Bos taurus OX=9913 GN=APOA4 PE=3 SV=1
5.	A0A4W2GRG6	43654	281	Apolipoprotein A4 OS=Bos indicus x Bos taurus OX=30522 GN=APOA4 PE=3 SV=1
6.	A0A4W2GSL9	46566	279	Apolipoprotein A4 OS=Bos indicus x Bos taurus OX=30522 GN=APOA4 PE=3 SV=1
7.	F1N3Q7	52487	271	Apolipoprotein A-IV OS=Bos taurus OX=9913 GN=APOA4 PE=3 SV=2
8.	Q32PJ2	42991	248	Apolipoprotein A-IV OS=Bos taurus OX=9913 GN=APOA4 PE=2 SV=1
9.	F1N6W4	145999	48	DENN domain containing 5A OS=Bos taurus OX=9913 GN=DENND5A PE=4 SV=3
10.	A0A3Q1MUN3	148571	48	DENN domain containing 5A OS=Bos taurus OX=9913 GN=DENND5A PE=4 SV=1
11.	A0A4W2DBN8	47465	46	Reverse transcriptase domain-containing protein OS=Bos indicus x Bos taurus OX=30522 PE=4 SV=1
12.	A0A4W2FJP8	473166	45	Dynein axonemal heavy chain 6 OS=Bos indicus x Bos taurus OX=30522 GN=DNAH6 PE=4 SV=1
13.	A0A4W2HIT4	478985	45	Dynein axonemal heavy chain 6 OS=Bos indicus x Bos taurus OX=30522 GN=DNAH6 PE=4 SV=1
14.	A0A3Q1LWT9	14058	44	Uncharacterized protein OS=Bos taurus OX=9913 PE=4 SV=1
15.	A0A4W2DIE4	14058	44	Uncharacterized protein OS=Bos indicus x Bos taurus OX=30522 PE=4 SV=1
16.	A0A4W2EFU9	24150	44	Hypoxanthine phosphoribosyltransferase OS=Bos indicus x Bos taurus OX=30522 PE=3 SV=1
17.	E1BA06	24150	44	Hypoxanthine phosphoribosyltransferase OS=Bos taurus OX=9913 PE=3 SV=2
18.	A0A4W2HDP5	60337	43	Zinc finger protein 382 OS=Bos indicus x Bos taurus OX=30522 GN=ZNF382 PE=4 SV=1
19.	A0A4W2FFL4	343058	43	Kalirin RhoGEF kinase OS=Bos indicus x Bos taurus OX=30522 GN=KALRN PE=4 SV=1
20.	A0A4W2EN51	85445	43	Cyclin and CBS domain divalent metal cation transport mediator 4 OS=Bos indicus x Bos taurus OX=30522 G
21.	A0A4W2F4Z6	60709	42	Uncharacterized protein OS=Bos indicus x Bos taurus OX=30522 PE=4 SV=1
22.	A0A4W2HZ94	170798	42	Lysine demethylase 2B OS=Bos indicus x Bos taurus OX=30522 GN=KDM2B PE=4 SV=1
23.	A0A3Q1MCG0	472745	42	Dynein axonemal heavy chain 6 OS=Bos taurus OX=9913 GN=DNAH6 PE=4 SV=1
24.	A0A4W2GJX2	108528	40	Nebulette OS=Bos indicus x Bos taurus OX=30522 GN=NEBL PE=4 SV=1
25.	A0A4W2EAZ9	473215	40	Dynein axonemal heavy chain 6 OS=Bos indicus x Bos taurus OX=30522 GN=DNAH6 PE=4 SV=1
26.	A0A4W2CJR0	108457	40	Nebulette OS=Bos indicus x Bos taurus OX=30522 GN=NEBL PE=4 SV=1
27.	A0A3Q1MXR2	108457	40	Nebulette OS=Bos taurus OX=9913 GN=NEBL PE=4 SV=1
28.	A0A4W2GHX5	112926	40	Nebulette OS=Bos indicus x Bos taurus OX=30522 GN=NEBL PE=4 SV=1
29.	A0A4W2CVR3	479034	40	Dynein axonemal heavy chain 6 OS=Bos indicus x Bos taurus OX=30522 GN=DNAH6 PE=4 SV=1
30.	A0A4W2I420	167724	40	Lysine demethylase 2B OS=Bos indicus x Bos taurus OX=30522 GN=KDM2B PE=4 SV=1

Results List

1. V6F7X3 **Mass:** 42963 **Score:** 283 **Expect:** 6.7e-024 **Matches:** 24

Apolipoprotein A-IV OS=Bos taurus OX=9913 GN=APOA4 PE=3 SV=1

Observed	Mr(expt)	Mr(calc)	ppm	Start - End	Miss	Ions	Peptide
862.4257	861.4184	861.4556	-43.12	288 - 295	0	---	K.SLAELSSR.L
977.4595	976.4522	976.4978	-46.64	156 - 163	0	---	R.QLTPYAER.M
977.4595	976.4523	976.4978	-46.61	156 - 163	0	15	R.QLTPYAER.M
1047.4978	1046.4905	1046.5396	-46.92	135 - 143	0	---	R.LGPYAEELR.T
1047.4978	1046.4905	1046.5396	-46.92	135 - 143	0	20	R.LGPYAEELR.T
1133.5562	1132.5489	1132.5989	-44.10	155 - 163	1	---	R.RQLTPYAER.M
1149.5057	1148.4984	1148.5462	-41.60	296 - 304	0	---	R.LDQQVEDFR.R
1149.5057	1148.4985	1148.5462	-41.57	296 - 304	0	20	R.LDQQVEDFR.R
1174.5699	1173.5626	1173.6176	-46.83	317 - 326	0	---	K.AMVQQLDTLR.Q
1174.5700	1173.5627	1173.6176	-46.79	317 - 326	0	50	K.AMVQQLDTLR.Q
1190.5620	1189.5547	1189.6125	-48.57	317 - 326	0	---	K.AMVQQLDTLR.Q + Oxidation (M)
1212.5294	1211.5221	1211.5823	-49.63	306 - 316	0	---	R.TVGPYGETFNK.A
1277.6251	1276.6178	1276.6663	-37.98	201 - 211	0	---	R.LTPYADQLQTK.I
1287.6068	1286.5995	1286.6579	-45.36	144 - 154	0	---	R.TQVDTQAQQLR.R
1287.6068	1286.5995	1286.6579	-45.35	144 - 154	0	14	R.TQVDTQAQQLR.R
1305.5947	1304.5874	1304.6473	-45.90	296 - 305	1	---	R.LDQQVEDFRR.T

PDF-XChange Click to buy NOW! www.docu-track.com

1311.6462	1310.6389	1310.6983	-45.28	80 -	90	0	---	K.LVPFATELHER.L
1311.6462	1310.6390	1310.6983	-45.25	80 -	90	0	14	K.LVPFATELHER.L
1439.7417	1438.7344	1438.7932	-40.87	79 -	90	1	---	K.KLVPFATELHER.L
1528.7235	1527.7162	1527.7868	-46.17	234 -	246	0	---	K.LNHQLEGLAFQMK.K
1664.7775	1663.7702	1663.8417	-42.96	52 -	65	0	---	K.SELTQQLNTLFQDK.L
1839.8657	1838.8584	1838.9414	-45.13	329 -	345	0	---	K.LGPLAGDVEDHLSFLEK.D
2700.2822	2699.2749	2699.3514	-28.34	170 -	193	0	---	R.QNLDQLQASLAPYAEELQATVNQR.V
2700.2822	2699.2750	2699.3514	-28.33	170 -	193	0	36	R.QNLDQLQASLAPYAEELQATVNQR.V

No match to: 834.4748, 842.4698, 848.4878, 850.0129, 850.4987, 853.4788, 864.5128, 865.9851, 868.5118, 870.5065, 876.9959, 882.5245, 882.5245, 892.9658, 951.5779, 960.4399, 996.5483, 1017.6229, 1017.6229, 1019.6358, 1033.6520, 1045.5190, 1051.6609, 1069.4803, 1131.6592, 1151.6812, 1156.5675, 1165.6880, 1165.6880, 1179.5543, 1182.6278, 1182.6278, 1194.5756, 1269.6084, 1300.7948, 1334.8268, 1425.6982, 1448.8599, 1475.6879, 1617.9976, 1691.7629, 1695.6990, 1702.7892, 1732.0278, 1880.9835, 1901.1722, 1904.9435, 2015.2070, 2211.0264, 2283.1013, 2682.3079, 2683.2788

2. A0A4W2CCP9 **Mass:** 42963 **Score:** 283 **Expect:** 6.7e-024 **Matches:** 24

Apolipoprotein A4 OS=Bos indicus x Bos taurus OX=30522 GN=APOA4 PE=3 SV=1

Observed	Mr(expt)	Mr(calc)	ppm	Start	End	Miss	Ions	Peptide
862.4257	861.4184	861.4556	-43.12	288 -	295	0	---	K.SLAELSSR.L
977.4595	976.4522	976.4978	-46.64	156 -	163	0	---	R.QLTPYAER.M
977.4595	976.4523	976.4978	-46.61	156 -	163	0	15	R.QLTPYAER.M
1047.4978	1046.4905	1046.5396	-46.92	135 -	143	0	---	R.LGPYAEELR.T
1047.4978	1046.4905	1046.5396	-46.92	135 -	143	0	20	R.LGPYAEELR.T
1133.5562	1132.5489	1132.5989	-44.10	155 -	163	1	---	R.RQLTPYAER.M
1149.5057	1148.4984	1148.5462	-41.60	296 -	304	0	---	R.LDQQVEDFR.R
1149.5057	1148.4985	1148.5462	-41.57	296 -	304	0	20	R.LDQQVEDFR.R
1174.5699	1173.5626	1173.6176	-46.83	317 -	326	0	---	K.AMVQQLDTLR.Q
1174.5700	1173.5627	1173.6176	-46.79	317 -	326	0	50	K.AMVQQLDTLR.Q
1190.5620	1189.5547	1189.6125	-48.57	317 -	326	0	---	K.AMVQQLDTLR.Q + Oxidation (M)
1212.5294	1211.5221	1211.5823	-49.63	306 -	316	0	---	R.TVGPYGETFNK.A
1277.6251	1276.6178	1276.6663	-37.98	201 -	211	0	---	R.LTPYADQLQTK.I
1287.6068	1286.5995	1286.6579	-45.36	144 -	154	0	---	R.TQVDTQAQQLR.R
1287.6068	1286.5995	1286.6579	-45.35	144 -	154	0	14	R.TQVDTQAQQLR.R
1305.5947	1304.5874	1304.6473	-45.90	296 -	305	1	---	R.LDQQVEDFRR.T
1311.6462	1310.6389	1310.6983	-45.28	80 -	90	0	---	K.LVPFATELHER.L
1311.6462	1310.6390	1310.6983	-45.25	80 -	90	0	14	K.LVPFATELHER.L
1439.7417	1438.7344	1438.7932	-40.87	79 -	90	1	---	K.KLVPFATELHER.L
1528.7235	1527.7162	1527.7868	-46.17	234 -	246	0	---	K.LNHQLEGLAFQMK.K
1664.7775	1663.7702	1663.8417	-42.96	52 -	65	0	---	K.SELTQQLNTLFQDK.L
1839.8657	1838.8584	1838.9414	-45.13	329 -	345	0	---	K.LGPLAGDVEDHLSFLEK.D
2700.2822	2699.2749	2699.3514	-28.34	170 -	193	0	---	R.QNLDQLQASLAPYAEELQATVNQR.V
2700.2822	2699.2750	2699.3514	-28.33	170 -	193	0	36	R.QNLDQLQASLAPYAEELQATVNQR.V

No match to: 834.4748, 842.4698, 848.4878, 850.0129, 850.4987, 853.4788, 864.5128, 865.9851, 868.5118, 870.5065, 876.9959, 882.5245, 882.5245, 892.9658, 951.5779, 960.4399, 996.5483, 1017.6229, 1017.6229, 1019.6358, 1033.6520, 1045.5190, 1051.6609, 1069.4803, 1131.6592, 1151.6812, 1156.5675, 1165.6880, 1165.6880, 1179.5543, 1182.6278, 1182.6278, 1194.5756, 1269.6084, 1300.7948, 1334.8268, 1425.6982, 1448.8599, 1475.6879, 1617.9976, 1691.7629, 1695.6990, 1702.7892, 1732.0278, 1880.9835, 1901.1722, 1904.9435, 2015.2070, 2211.0264, 2283.1013, 2682.3079, 2683.2788

3. A0A3Q1M3N0 **Mass:** 43165 **Score:** 283 **Expect:** 6.7e-024 **Matches:** 24

Apolipoprotein A-IV OS=Bos taurus OX=9913 GN=APOA4 PE=3 SV=1

Observed	Mr(expt)	Mr(calc)	ppm	Start	End	Miss	Ions	Peptide
862.4257	861.4184	861.4556	-43.12	313 -	320	0	---	K.SLAELSSR.L
977.4595	976.4522	976.4978	-46.64	181 -	188	0	---	R.QLTPYAER.M
977.4595	976.4523	976.4978	-46.61	181 -	188	0	15	R.QLTPYAER.M
1047.4978	1046.4905	1046.5396	-46.92	160 -	168	0	---	R.LGPYAEELR.T
1047.4978	1046.4905	1046.5396	-46.92	160 -	168	0	20	R.LGPYAEELR.T
1133.5562	1132.5489	1132.5989	-44.10	180 -	188	1	---	R.RQLTPYAER.M
1149.5057	1148.4984	1148.5462	-41.60	321 -	329	0	---	R.LDQQVEDFR.R
1149.5057	1148.4985	1148.5462	-41.57	321 -	329	0	20	R.LDQQVEDFR.R
1174.5699	1173.5626	1173.6176	-46.83	342 -	351	0	---	K.AMVQQLDTLR.Q
1174.5700	1173.5627	1173.6176	-46.79	342 -	351	0	50	K.AMVQQLDTLR.Q
1190.5620	1189.5547	1189.6125	-48.57	342 -	351	0	---	K.AMVQQLDTLR.Q + Oxidation (M)
1212.5294	1211.5221	1211.5823	-49.63	331 -	341	0	---	R.TVGPYGETFNK.A
1277.6251	1276.6178	1276.6663	-37.98	226 -	236	0	---	R.LTPYADQLQTK.I
1287.6068	1286.5995	1286.6579	-45.36	169 -	179	0	---	R.TQVDTQAQQLR.R
1287.6068	1286.5995	1286.6579	-45.35	169 -	179	0	14	R.TQVDTQAQQLR.R
1305.5947	1304.5874	1304.6473	-45.90	321 -	330	1	---	R.LDQQVEDFRR.T
1311.6462	1310.6389	1310.6983	-45.28	105 -	115	0	---	K.LVPFATELHER.L
1311.6462	1310.6390	1310.6983	-45.25	105 -	115	0	14	K.LVPFATELHER.L
1439.7417	1438.7344	1438.7932	-40.87	104 -	115	1	---	K.KLVPFATELHER.L
1528.7235	1527.7162	1527.7868	-46.17	259 -	271	0	---	K.LNHQLEGLAFQMK.K
1664.7775	1663.7702	1663.8417	-42.96	77 -	90	0	---	K.SELTQQLNTLFQDK.L
1839.8657	1838.8584	1838.9414	-45.13	354 -	370	0	---	K.LGPLAGDVEDHLSFLEK.D
2700.2822	2699.2749	2699.3514	-28.34	195 -	218	0	---	R.QNLDQLQASLAPYAEELQATVNQR.V
2700.2822	2699.2750	2699.3514	-28.33	195 -	218	0	36	R.QNLDQLQASLAPYAEELQATVNQR.V

No match to: 834.4748, 842.4698, 848.4878, 850.0129, 850.4987, 853.4788, 864.5128, 865.9851, 868.5118, 870.5065, 876.9959, 882.5245, 882.5245, 892.9658, 951.5779, 960.4399, 996.5483, 1017.6229, 1017.6229, 1019.6358, 1033.6520, 1045.5190, 1051.6609, 1069.4803, 1131.6592, 1151.6812, 1156.5675, 1165.6880, 1165.6880, 1179.5543, 1182.6278, 1182.6278, 1194.5756, 1269.6084, 1300.7948, 1334.8268, 1425.6982, 1448.8599, 1475.6879, 1617.9976, 1691.7629, 1695.6990, 1702.7892, 1732.0278, 1880.9835, 1901.1722, 1904.9435, 2015.2070, 2211.0264, 2283.1013, 2682.3079, 2683.2788

4. A0A3Q1MZJ9 **Mass:** 45782 **Score:** 282 **Expect:** 8.5e-024 **Matches:** 24

Apolipoprotein A-IV OS=Bos taurus OX=9913 GN=APOA4 PE=3 SV=1

Observed	Mr(expt)	Mr(calc)	ppm	Start	End	Miss	Ions	Peptide
862.4257	861.4184	861.4556	-43.12	339 -	346	0	---	K.SLAELSSR.L
977.4595	976.4522	976.4978	-46.64	207 -	214	0	---	R.QLTPYAER.M
977.4595	976.4523	976.4978	-46.61	207 -	214	0	15	R.QLTPYAER.M
1047.4978	1046.4905	1046.5396	-46.92	186 -	194	0	---	R.LGPYAEELR.T
1047.4978	1046.4905	1046.5396	-46.92	186 -	194	0	20	R.LGPYAEELR.T
1133.5562	1132.5489	1132.5989	-44.10	206 -	214	1	---	R.RQLTPYAER.M
1149.5057	1148.4984	1148.5462	-41.60	347 -	355	0	---	R.LDQQVEDFR.R
1149.5057	1148.4985	1148.5462	-41.57	347 -	355	0	20	R.LDQQVEDFR.R
1174.5699	1173.5626	1173.6176	-46.83	368 -	377	0	---	K.AMVQQLDTLR.Q
1174.5700	1173.5627	1173.6176	-46.79	368 -	377	0	50	K.AMVQQLDTLR.Q
1190.5620	1189.5547	1189.6125	-48.57	368 -	377	0	---	K.AMVQQLDTLR.Q + Oxidation (M)

PDF-XChange Click to buy NOW! www.docu-track.com

1212.5294	1211.5221	1211.5823	-49.63	357	367	0	---	R.TVGPYGETFNK.A
1277.6251	1276.6178	1276.6663	-37.98	252	262	0	---	R.LTPYADQLQTK.I
1287.6068	1286.5995	1286.6579	-45.36	195	205	0	---	R.TQVDTQAQQLR.R
1287.6068	1286.5995	1286.6579	-45.35	195	205	0	14	R.TQVDTQAQQLR.R
1305.5947	1304.5874	1304.6473	-45.90	347	356	1	---	R.LDQQVEDFRR.T
1311.6462	1310.6389	1310.6983	-45.28	131	141	0	---	K.LVPFATELHER.L
1311.6462	1310.6390	1310.6983	-45.25	131	141	0	14	K.LVPFATELHER.L
1439.7417	1438.7344	1438.7932	-40.87	130	141	1	---	K.KLVPFATELHER.L
1528.7235	1527.7162	1527.7868	-46.17	285	297	0	---	K.LNHQLEGLAFQMK.K
1664.7775	1663.7702	1663.8417	-42.96	103	116	0	---	K.SELTQQLNTLFQDK.L
1839.8657	1838.8584	1838.9414	-45.13	380	396	0	---	K.LGPLAGDVEDHLSFLEK.D
2700.2822	2699.2749	2699.3514	-28.34	221	244	0	---	R.QNLDQLQASLAPYAEELQATVNQR.V
2700.2822	2699.2750	2699.3514	-28.33	221	244	0	36	R.QNLDQLQASLAPYAEELQATVNQR.V

No match to: 834.4748, 842.4698, 848.4878, 850.0129, 850.4987, 853.4788, 864.5128, 865.9851, 868.5118, 870.5065, 876.9959, 882.5245, 882.5245, 892.9658, 951.5779, 960.4399, 996.5483, 1017.6229, 1017.6229, 1019.6358, 1033.6520, 1045.5190, 1051.6609, 1069.4803, 1131.6592, 1151.6812, 1156.5675, 1165.6880, 1165.6880, 1179.5543, 1182.6278, 1182.6278, 1194.5756, 1269.6084, 1300.7948, 1334.8268, 1425.6982, 1448.8599, 1475.6879, 1617.9976, 1691.7629, 1695.6990, 1702.7892, 1732.0278, 1880.9835, 1901.1722, 1904.9435, 2015.2070, 2211.0264, 2283.1013, 2682.3079, 2683.2788

5. A0A4W2GRG6 **Mass:** 43654 **Score:** 281 **Expect:** 1.1e-023 **Matches:** 24

Apolipoprotein A4 OS=Bos indicus x Bos taurus OX=30522 GN=APOA4 PE=3 SV=1

Observed	Mr(expt)	Mr(calc)	ppm	Start	End	Miss	Ions	Peptide
862.4257	861.4184	861.4556	-43.12	318	325	0	---	K.SLAELSSR.L
977.4595	976.4522	976.4978	-46.64	186	193	0	---	R.QLTPYAER.M
977.4595	976.4523	976.4978	-46.61	186	193	0	15	R.QLTPYAER.M
1047.4978	1046.4905	1046.5396	-46.92	165	173	0	---	R.LGPYAEELR.T
1047.4978	1046.4905	1046.5396	-46.92	165	173	0	20	R.LGPYAEELR.T
1133.5562	1132.5489	1132.5989	-44.10	185	193	1	---	R.RQLTPYAER.M
1149.5057	1148.4984	1148.5462	-41.60	326	334	0	---	R.LDQQVEDFR.R
1149.5057	1148.4985	1148.5462	-41.57	326	334	0	20	R.LDQQVEDFR.R
1174.5699	1173.5626	1173.6176	-46.83	347	356	0	---	K.AMVQQLDTLR.Q
1174.5700	1173.5627	1173.6176	-46.79	347	356	0	50	K.AMVQQLDTLR.Q
1190.5620	1189.5547	1189.6125	-48.57	347	356	0	---	K.AMVQQLDTLR.Q + Oxidation (M)
1212.5294	1211.5221	1211.5823	-49.63	336	346	0	---	R.TVGPYGETFNK.A
1277.6251	1276.6178	1276.6663	-37.98	231	241	0	---	R.LTPYADQLQTK.I
1287.6068	1286.5995	1286.6579	-45.36	174	184	0	---	R.TQVDTQAQQLR.R
1287.6068	1286.5995	1286.6579	-45.35	174	184	0	14	R.TQVDTQAQQLR.R
1305.5947	1304.5874	1304.6473	-45.90	326	335	1	---	R.LDQQVEDFRR.T
1311.6462	1310.6389	1310.6983	-45.28	110	120	0	---	K.LVPFATELHER.L
1311.6462	1310.6390	1310.6983	-45.25	110	120	0	14	K.LVPFATELHER.L
1439.7417	1438.7344	1438.7932	-40.87	109	120	1	---	K.KLVPFATELHER.L
1528.7235	1527.7162	1527.7868	-46.17	264	276	0	---	K.LNHQLEGLAFQMK.K
1664.7775	1663.7702	1663.8417	-42.96	82	95	0	---	K.SELTQQLNTLFQDK.L
1839.8657	1838.8584	1838.9414	-45.13	359	375	0	---	K.LGPLAGDVEDHLSFLEK.D
2700.2822	2699.2749	2699.3514	-28.34	200	223	0	---	R.QNLDQLQASLAPYAEELQATVNQR.V
2700.2822	2699.2750	2699.3514	-28.33	200	223	0	36	R.QNLDQLQASLAPYAEELQATVNQR.V

No match to: 834.4748, 842.4698, 848.4878, 850.0129, 850.4987, 853.4788, 864.5128, 865.9851, 868.5118, 870.5065, 876.9959, 882.5245, 882.5245, 892.9658, 951.5779, 960.4399, 996.5483, 1017.6229, 1017.6229, 1019.6358, 1033.6520, 1045.5190, 1051.6609, 1069.4803, 1131.6592, 1151.6812, 1156.5675, 1165.6880, 1165.6880, 1179.5543, 1182.6278, 1182.6278, 1194.5756, 1269.6084, 1300.7948, 1334.8268, 1425.6982, 1448.8599, 1475.6879, 1617.9976, 1691.7629, 1695.6990, 1702.7892, 1732.0278, 1880.9835, 1901.1722, 1904.9435, 2015.2070, 2211.0264, 2283.1013, 2682.3079, 2683.2788

6. A0A4W2GSL9 **Mass:** 46566 **Score:** 279 **Expect:** 1.7e-023 **Matches:** 24

Apolipoprotein A4 OS=Bos indicus x Bos taurus OX=30522 GN=APOA4 PE=3 SV=1

Observed	Mr(expt)	Mr(calc)	ppm	Start	End	Miss	Ions	Peptide
862.4257	861.4184	861.4556	-43.12	318	325	0	---	K.SLAELSSR.L
977.4595	976.4522	976.4978	-46.64	186	193	0	---	R.QLTPYAER.M
977.4595	976.4523	976.4978	-46.61	186	193	0	15	R.QLTPYAER.M
1047.4978	1046.4905	1046.5396	-46.92	165	173	0	---	R.LGPYAEELR.T
1047.4978	1046.4905	1046.5396	-46.92	165	173	0	20	R.LGPYAEELR.T
1133.5562	1132.5489	1132.5989	-44.10	185	193	1	---	R.RQLTPYAER.M
1149.5057	1148.4984	1148.5462	-41.60	326	334	0	---	R.LDQQVEDFR.R
1149.5057	1148.4985	1148.5462	-41.57	326	334	0	20	R.LDQQVEDFR.R
1174.5699	1173.5626	1173.6176	-46.83	347	356	0	---	K.AMVQQLDTLR.Q
1174.5700	1173.5627	1173.6176	-46.79	347	356	0	50	K.AMVQQLDTLR.Q
1190.5620	1189.5547	1189.6125	-48.57	347	356	0	---	K.AMVQQLDTLR.Q + Oxidation (M)
1212.5294	1211.5221	1211.5823	-49.63	336	346	0	---	R.TVGPYGETFNK.A
1277.6251	1276.6178	1276.6663	-37.98	231	241	0	---	R.LTPYADQLQTK.I
1287.6068	1286.5995	1286.6579	-45.36	174	184	0	---	R.TQVDTQAQQLR.R
1287.6068	1286.5995	1286.6579	-45.35	174	184	0	14	R.TQVDTQAQQLR.R
1305.5947	1304.5874	1304.6473	-45.90	326	335	1	---	R.LDQQVEDFRR.T
1311.6462	1310.6389	1310.6983	-45.28	110	120	0	---	K.LVPFATELHER.L
1311.6462	1310.6390	1310.6983	-45.25	110	120	0	14	K.LVPFATELHER.L
1439.7417	1438.7344	1438.7932	-40.87	109	120	1	---	K.KLVPFATELHER.L
1528.7235	1527.7162	1527.7868	-46.17	264	276	0	---	K.LNHQLEGLAFQMK.K
1664.7775	1663.7702	1663.8417	-42.96	82	95	0	---	K.SELTQQLNTLFQDK.L
1839.8657	1838.8584	1838.9414	-45.13	359	375	0	---	K.LGPLAGDVEDHLSFLEK.D
2700.2822	2699.2749	2699.3514	-28.34	200	223	0	---	R.QNLDQLQASLAPYAEELQATVNQR.V
2700.2822	2699.2750	2699.3514	-28.33	200	223	0	36	R.QNLDQLQASLAPYAEELQATVNQR.V

No match to: 834.4748, 842.4698, 848.4878, 850.0129, 850.4987, 853.4788, 864.5128, 865.9851, 868.5118, 870.5065, 876.9959, 882.5245, 882.5245, 892.9658, 951.5779, 960.4399, 996.5483, 1017.6229, 1017.6229, 1019.6358, 1033.6520, 1045.5190, 1051.6609, 1069.4803, 1131.6592, 1151.6812, 1156.5675, 1165.6880, 1165.6880, 1179.5543, 1182.6278, 1182.6278, 1194.5756, 1269.6084, 1300.7948, 1334.8268, 1425.6982, 1448.8599, 1475.6879, 1617.9976, 1691.7629, 1695.6990, 1702.7892, 1732.0278, 1880.9835, 1901.1722, 1904.9435, 2015.2070, 2211.0264, 2283.1013, 2682.3079, 2683.2788

7. F1N3Q7 **Mass:** 52487 **Score:** 271 **Expect:** 1.1e-022 **Matches:** 24

Apolipoprotein A-IV OS=Bos taurus OX=9913 GN=APOA4 PE=3 SV=2

Observed	Mr(expt)	Mr(calc)	ppm	Start	End	Miss	Ions	Peptide
862.4257	861.4184	861.4556	-43.12	373	380	0	---	K.SLAELSSR.L
977.4595	976.4522	976.4978	-46.64	241	248	0	---	R.QLTPYAER.M
977.4595	976.4523	976.4978	-46.61	241	248	0	15	R.QLTPYAER.M
1047.4978	1046.4905	1046.5396	-46.92	220	228	0	---	R.LGPYAEELR.T
1047.4978	1046.4905	1046.5396	-46.92	220	228	0	20	R.LGPYAEELR.T
1133.5562	1132.5489	1132.5989	-44.10	240	248	1	---	R.RQLTPYAER.M

PDF-XChange Click to buy NOW! www.docu-track.com

PDF-XChange Click to buy NOW! www.docu-track.com

1149.5057	1148.4984	1148.5462	-41.60	381 -	389	0	---	R.LDQQVEDFR.R
1149.5057	1148.4985	1148.5462	-41.57	381 -	389	0	20	R.LDQQVEDFR.R
1174.5699	1173.5626	1173.6176	-46.83	402 -	411	0	---	K.AMVQQLDTLR.Q
1174.5700	1173.5627	1173.6176	-46.79	402 -	411	0	50	K.AMVQQLDTLR.Q
1190.5620	1189.5547	1189.6125	-48.57	402 -	411	0	---	K.AMVQQLDTLR.Q + Oxidation (M)
1212.5294	1211.5221	1211.5823	-49.63	391 -	401	0	---	R.TVGPYGETFNK.A
1277.6251	1276.6178	1276.6663	-37.98	286 -	296	0	---	R.LTPYADQLQTK.I
1287.6068	1286.5995	1286.6579	-45.36	229 -	239	0	---	R.TQVDTQAQQLR.R
1287.6068	1286.5995	1286.6579	-45.35	229 -	239	0	14	R.TQVDTQAQQLR.R
1305.5947	1304.5874	1304.6473	-45.90	381 -	390	1	---	R.LDQQVEDFRR.T
1311.6462	1310.6389	1310.6983	-45.28	165 -	175	0	---	K.LVPFATELHER.L
1311.6462	1310.6390	1310.6983	-45.25	165 -	175	0	14	K.LVPFATELHER.L
1439.7417	1438.7344	1438.7932	-40.87	164 -	175	1	---	K.KLVPFATELHER.L
1528.7235	1527.7162	1527.7868	-46.17	319 -	331	0	---	K.LNHQLEGLAFQMK.K
1664.7775	1663.7702	1663.8417	-42.96	137 -	150	0	---	K.SELTQQLNTLFQDK.L
1839.8657	1838.8584	1838.9414	-45.13	414 -	430	0	---	K.LGPLAGDVEDHLSFLEK.D
2700.2822	2699.2749	2699.3514	-28.34	255 -	278	0	---	R.QNLDQLQASLAPYAEELQATVNQR.V
2700.2822	2699.2750	2699.3514	-28.33	255 -	278	0	36	R.QNLDQLQASLAPYAEELQATVNQR.V

No match to: 834.4748, 842.4698, 848.4878, 850.0129, 850.4987, 853.4788, 864.5128, 865.9851, 868.5118, 870.5065, 876.9959, 882.5245, 882.5245, 892.9658, 951.5779, 960.4399, 996.5483, 1017.6229, 1017.6229, 1019.6358, 1033.6520, 1045.5190, 1051.6609, 1069.4803, 1131.6592, 1151.6812, 1156.5675, 1165.6880, 1165.6880, 1179.5543, 1182.6278, 1182.6278, 1194.5756, 1269.6084, 1300.7948, 1334.8268, 1425.6982, 1448.8599, 1475.6879, 1617.9976, 1691.7629, 1695.6990, 1702.7892, 1732.0278, 1880.9835, 1901.1722, 1904.9435, 2015.2070, 2211.0264, 2283.1013, 2682.3079, 2683.2788

8. Q32PJ2 **Mass:** 42991 **Score:** 248 **Expect:** 2.1e-020 **Matches:** 21

Apolipoprotein A-IV OS=Bos taurus OX=9913 GN=APOA4 PE=2 SV=1

Observed	Mr(expt)	Mr(calc)	ppm	Start	End	Miss	Ions	Peptide
862.4257	861.4184	861.4556	-43.12	288 -	295	0	---	K.SLAELSSR.L
1047.4978	1046.4905	1046.5396	-46.92	135 -	143	0	---	R.LGPYAEELR.T
1047.4978	1046.4905	1046.5396	-46.92	135 -	143	0	20	R.LGPYAEELR.T
1149.5057	1148.4984	1148.5462	-41.60	296 -	304	0	---	R.LDQQVEDFR.R
1149.5057	1148.4985	1148.5462	-41.57	296 -	304	0	20	R.LDQQVEDFR.R
1174.5699	1173.5626	1173.6176	-46.83	317 -	326	0	---	K.AMVQQLDTLR.Q
1174.5700	1173.5627	1173.6176	-46.79	317 -	326	0	50	K.AMVQQLDTLR.Q
1190.5620	1189.5547	1189.6125	-48.57	317 -	326	0	---	K.AMVQQLDTLR.Q + Oxidation (M)
1212.5294	1211.5221	1211.5823	-49.63	306 -	316	0	---	R.TVGPYGETFNK.A
1277.6251	1276.6178	1276.6663	-37.98	201 -	211	0	---	R.LTPYADQLQTK.I
1287.6068	1286.5995	1286.6579	-45.36	144 -	154	0	---	R.TQVDTQAQQLR.R
1287.6068	1286.5995	1286.6579	-45.35	144 -	154	0	14	R.TQVDTQAQQLR.R
1305.5947	1304.5874	1304.6473	-45.90	296 -	305	1	---	R.LDQQVEDFRR.T
1311.6462	1310.6389	1310.6983	-45.28	80 -	90	0	---	K.LVPFATELHER.L
1311.6462	1310.6390	1310.6983	-45.25	80 -	90	0	14	K.LVPFATELHER.L
1439.7417	1438.7344	1438.7932	-40.87	79 -	90	1	---	K.KLVPFATELHER.L
1528.7235	1527.7162	1527.7868	-46.17	234 -	246	0	---	K.LNHQLEGLAFQMK.K
1664.7775	1663.7702	1663.8417	-42.96	52 -	65	0	---	K.SELTQQLNTLFQDK.L
1839.8657	1838.8584	1838.9414	-45.13	329 -	345	0	---	K.LGPLAGDVEDHLSFLEK.D
2700.2822	2699.2749	2699.3514	-28.34	170 -	193	0	---	R.QNLDQLQASLAPYAEELQATVNQR.V
2700.2822	2699.2750	2699.3514	-28.33	170 -	193	0	36	R.QNLDQLQASLAPYAEELQATVNQR.V

No match to: 834.4748, 842.4698, 848.4878, 850.0129, 850.4987, 853.4788, 864.5128, 865.9851, 868.5118, 870.5065, 876.9959, 882.5245, 882.5245, 892.9658, 951.5779, 960.4399, 977.4595, 977.4595, 996.5483, 1017.6229, 1017.6229, 1019.6358, 1033.6520, 1045.5190, 1051.6609, 1069.4803, 1131.6592, 1133.5562, 1151.6812, 1156.5675, 1165.6880, 1165.6880, 1179.5543, 1182.6278, 1182.6278, 1194.5756, 1269.6084, 1300.7948, 1334.8268, 1425.6982, 1448.8599, 1475.6879, 1617.9976, 1691.7629, 1695.6990, 1702.7892, 1732.0278, 1880.9835, 1901.1722, 1904.9435, 2015.2070, 2211.0264, 2283.1013, 2682.3079, 2683.2788

9. F1N6W4 **Mass:** 145999 **Score:** 48 **Expect:** 2 **Matches:** 12

DENN domain containing 5A OS=Bos taurus OX=9913 GN=DENND5A PE=4 SV=3

Observed	Mr(expt)	Mr(calc)	ppm	Start	End	Miss	Ions	Peptide
842.4698	841.4625	841.4195	51.2	670 -	676	0	---	R.NAPAQWR.R
848.4878	847.4805	847.4287	61.2	419 -	426	0	---	R.ASELVSDK.R
1051.6609	1050.6536	1050.5822	68.0	849 -	857	0	---	R.HIQNIGEIK.T
1131.6592	1130.6519	1130.5866	57.8	704 -	713	1	---	R.NMGSTIRQPK.L
1174.5699	1173.5626	1173.5123	42.9	2 -	17	0	---	M.SGGGGGGGGGGSAPSR.F
1174.5700	1173.5627	1173.5123	42.9	2 -	17	0	8	M.SGGGGGGGGGGSAPSR.F
1182.6278	1181.6205	1181.7244	-87.92	592 -	601	1	---	R.LLNVRTPTLR.T
1182.6278	1181.6205	1181.7244	-87.92	592 -	601	1	24	R.LLNVRTPTLR.T
1194.5756	1193.5683	1193.6049	-30.66	1197 -	1206	0	---	K.FQMLVCLGAR.D
1305.5947	1304.5874	1304.5528	26.5	1 -	17	0	---	-.MSGGGGGGGGGGSAPSR.F
1311.6462	1310.6389	1310.7194	-61.39	882 -	892	1	---	K.QLLSDHELTKK.L
1311.6462	1310.6390	1310.7194	-61.36	882 -	892	1	---	K.QLLSDHELTKK.L

No match to: 834.4748, 850.0129, 850.4987, 853.4788, 862.4257, 864.5128, 865.9851, 868.5118, 870.5065, 876.9959, 882.5245, 882.5245, 892.9658, 951.5779, 960.4399, 977.4595, 977.4595, 996.5483, 1017.6229, 1017.6229, 1019.6358, 1033.6520, 1045.5190, 1047.4978, 1047.4978, 1069.4803, 1133.5562, 1149.5057, 1149.5057, 1151.6812, 1156.5675, 1165.6880, 1165.6880, 1179.5543, 1190.5620, 1212.5294, 1269.6084, 1277.6251, 1287.6068, 1287.6068, 1300.7948, 1334.8268, 1425.6982, 1439.7417, 1448.8599, 1475.6879, 1528.7235, 1617.9976, 1664.7775, 1691.7629, 1695.6990, 1702.7892, 1732.0278, 1839.8657, 1880.9835, 1901.1722, 1904.9435, 2015.2070, 2211.0264, 2283.1013, 2682.3079, 2683.2788, 2700.2822, 2700.2822

10. A0A3Q1MUN3 **Mass:** 148571 **Score:** 48 **Expect:** 2.2 **Matches:** 12

DENN domain containing 5A OS=Bos taurus OX=9913 GN=DENND5A PE=4 SV=1

Observed	Mr(expt)	Mr(calc)	ppm	Start	End	Miss	Ions	Peptide
842.4698	841.4625	841.4195	51.2	694 -	700	0	---	R.NAPAQWR.R
848.4878	847.4805	847.4287	61.2	443 -	450	0	---	R.ASELVSDK.R
1051.6609	1050.6536	1050.5822	68.0	873 -	881	0	---	R.HIQNIGEIK.T
1131.6592	1130.6519	1130.5866	57.8	728 -	737	1	---	R.NMGSTIRQPK.L
1174.5699	1173.5626	1173.5123	42.9	2 -	17	0	---	M.SGGGGGGGGGGSAPSR.F
1174.5700	1173.5627	1173.5123	42.9	2 -	17	0	8	M.SGGGGGGGGGGSAPSR.F
1182.6278	1181.6205	1181.7244	-87.92	616 -	625	1	---	R.LLNVRTPTLR.T
1182.6278	1181.6205	1181.7244	-87.92	616 -	625	1	24	R.LLNVRTPTLR.T
1194.5756	1193.5683	1193.6049	-30.66	1221 -	1230	0	---	K.FQMLVCLGAR.D
1305.5947	1304.5874	1304.5528	26.5	1 -	17	0	---	-.MSGGGGGGGGGGSAPSR.F
1311.6462	1310.6389	1310.7194	-61.39	906 -	916	1	---	K.QLLSDHELTKK.L
1311.6462	1310.6390	1310.7194	-61.36	906 -	916	1	---	K.QLLSDHELTKK.L

No match to: 834.4748, 850.0129, 850.4987, 853.4788, 862.4257, 864.5128, 865.9851, 868.5118, 870.5065, 876.9959, 882.5245, 882.5245, 892.9658, 951.5779, 960.4399, 977.4595, 977.4595, 996.5483, 1017.6229, 1017.6229, 1019.6358, 1033.6520, 1045.5190, 1047.4978, 1047.4978, 1069.4803, 1133.5562, 1149.5057, 1149.5057, 1151.6812, 1156.5675, 1165.6880, 1165.6880, 1179.5543,

PDF-XChange Click to buy NOW! www.docu-track.com

PDF-XChange Click to buy NOW! www.docu-track.com

1190.5620, 1212.5294, 1269.6084, 1277.6251, 1287.6068, 1287.6068, 1300.7948, 1334.8268, 1425.6982, 1439.7417, 1448.8599, 1475.6879, 1528.7235, 1617.9976, 1664.7775, 1691.7629, 1695.6990, 1702.7892, 1732.0278, 1839.8657, 1880.9835, 1901.1722, 1904.9435, 2015.2070, 2211.0264, 2283.1013, 2682.3079, 2683.2788, 2700.2822, 2700.2822

11. A0A4W2DBN8 **Mass:** 47465 **Score:** 46 **Expect:** 3.2 **Matches:** 14

Reverse transcriptase domain-containing protein OS=Bos indicus x Bos taurus OX=30522 PE=4 SV=1

Observed	Mr(expt)	Mr(calc)	ppm	Start	End	Miss	Ions	Peptide
834.4748	833.4675	833.5044	-44.29	305	311	1	---	K.SLLMKVK.E + Oxidation (M)
848.4878	847.4805	847.4287	61.2	310	316	1	---	K.VKEESEK.V
882.5245	881.5172	881.4607	64.2	387	393	1	---	K.SRDITYK.G
882.5245	881.5173	881.4607	64.2	387	393	1	---	K.SRDITYK.G
1131.6592	1130.6519	1130.5720	70.7	148	157	0	---	R.ELPDVQAGFR.K
1133.5562	1132.5489	1132.6162	-59.38	377	386	0	---	K.VMTNLDSILK.S
1149.5057	1148.4984	1148.6111	-98.10	377	386	0	---	K.VMTNLDSILK.S + Oxidation (M)
1149.5057	1148.4985	1148.6111	-98.06	377	386	0	---	K.VMTNLDSILK.S + Oxidation (M)
1269.6084	1268.6011	1268.7101	-85.93	279	288	1	---	K.IAWRNINNLR.Y
1277.6251	1276.6178	1276.7061	-69.11	376	386	1	---	R.KVMTNLDSILK.S + Oxidation (M)
1287.6068	1286.5995	1286.6731	-57.20	147	157	1	---	K.RELPDVQAGFR.K
1287.6068	1286.5995	1286.6731	-57.20	147	157	1	---	K.RELPDVQAGFR.K
1702.7892	1701.7819	1701.7570	14.7	394	409	1	---	K.GPSSQGYGFSSGRVWM.-
2683.2788	2682.2715	2682.2644	2.65	245	266	0	---	K.GVCQGYILSPCLFNLYAEYIMR.N + Oxidation (M)

No match to: 842.4698, 850.0129, 850.4987, 853.4788, 862.4257, 864.5128, 865.9851, 868.5118, 870.5065, 876.9959, 892.9658, 951.5779, 960.4399, 977.4595, 977.4595, 996.5483, 1017.6229, 1017.6229, 1019.6358, 1033.6520, 1045.5190, 1047.4978, 1047.4978, 1051.6609, 1069.4803, 1151.6812, 1156.5675, 1165.6880, 1165.6880, 1174.5699, 1174.5700, 1179.5543, 1182.6278, 1182.6278, 1190.5620, 1194.5756, 1212.5294, 1300.7948, 1305.5947, 1311.6462, 1311.6462, 1334.8268, 1425.6982, 1439.7417, 1448.8599, 1475.6879, 1528.7235, 1617.9976, 1664.7775, 1691.7629, 1695.6990, 1732.0278, 1839.8657, 1880.9835, 1901.1722, 1904.9435, 2015.2070, 2211.0264, 2283.1013, 2682.3079, 2700.2822, 2700.2822

12. A0A4W2FJP8 **Mass:** 473166 **Score:** 45 **Expect:** 4.1 **Matches:** 30

Dynein axonemal heavy chain 6 OS=Bos indicus x Bos taurus OX=30522 GN=DNAH6 PE=4 SV=1

Observed	Mr(expt)	Mr(calc)	ppm	Start	End	Miss	Ions	Peptide
834.4748	833.4675	833.4647	3.40	1784	1790	0	---	K.TYVLNPK.S
848.4878	847.4805	847.4334	55.6	2431	2436	1	---	R.MIRQER.G + Oxidation (M)
864.5128	863.5055	863.4753	35.0	925	931	0	---	K.FVTQLEK.G
868.5118	867.5045	867.4464	67.1	649	654	1	---	K.YHRQHK.D
960.4399	959.4326	959.4672	-36.05	2545	2553	0	---	K.EVGISEGNR.D
977.4595	976.4522	976.5012	-50.11	1810	1818	0	---	K.DGLMALSVR.A + Oxidation (M)
977.4595	976.4523	976.5012	-50.08	1810	1818	0	---	K.DGLMALSVR.A + Oxidation (M)
1045.5190	1044.5117	1044.5451	-31.97	3977	3986	0	---	R.DQATVIEAAK.T
1131.6592	1130.6519	1130.5608	80.6	232	240	1	---	K.NDYYTISKK.A
1149.5057	1148.4984	1148.4855	11.2	2320	2327	0	---	R.LFCHECQR.V
1149.5057	1148.4985	1148.4855	11.3	2320	2327	0	---	R.LFCHECQR.V
1156.5675	1155.5602	1155.5746	-12.48	1551	1559	1	---	R.FMFEGREIK.L
1182.6278	1181.6205	1181.5750	38.5	1280	1289	0	---	K.VEEAMFTSLR.R
1182.6278	1181.6205	1181.5750	38.5	1280	1289	0	5	K.VEEAMFTSLR.R
1287.6068	1286.5995	1286.7207	-94.20	1205	1215	1	---	R.NPQAVQPHLRK.C
1287.6068	1286.5995	1286.7207	-94.19	1205	1215	1	---	R.NPQAVQPHLRK.C
1425.6982	1424.6909	1424.7088	-12.57	3334	3344	0	---	R.LTEAEWNFFLR.G
1439.7417	1438.7344	1438.6472	60.6	1889	1900	0	---	R.CGMVFVDPEELK.W + Oxidation (M)
1448.8599	1447.8526	1447.9238	-49.18	1355	1368	1	---	R.LNALAAIVRGILPK.L
1475.6879	1474.6806	1474.7701	-60.67	2856	2869	0	---	R.AAQAELDITMATLK.E
1528.7235	1527.7162	1527.8046	-57.81	1030	1042	1	---	K.TTEFVVLPHRDSK.D
1617.9976	1616.9903	1616.8596	80.9	1119	1132	1	---	R.QLPAEAKMFLQVDK.S
1664.7775	1663.7702	1663.9185	-89.12	3454	3468	0	---	K.VVFALTDFVIENLGK.A
1691.7629	1690.7556	1690.8414	-50.71	907	921	0	---	K.FDSLDPELLNSQVSK.Y
1880.9835	1879.9762	1879.9138	33.2	4092	4107	1	---	K.DYWIAKGSALLCQLNE.-
1901.1722	1900.1649	1900.0319	70.0	2415	2430	1	---	K.LVFFQDAIEHVSRIAR.M
1904.9435	1903.9362	1903.9778	-21.86	3818	3834	0	---	R.VPEILETENASESLFVK.D
2211.0264	2210.0191	2210.0677	-22.00	1271	1289	1	---	R.GNVEEWLGKVEEAMFTSLR.R + Oxidation (M)
2682.3079	2681.3006	2681.3871	-32.27	3145	3167	1	---	K.SGLEDQLLSDVVRLEKPELEEQR.I
2683.2788	2682.2715	2682.3725	-37.66	2999	3022	1	---	R.QWNTDGLPRDLISTENGILVTQGR.R

No match to: 842.4698, 850.0129, 850.4987, 853.4788, 862.4257, 865.9851, 870.5065, 876.9959, 882.5245, 882.5245, 892.9658, 951.5779, 996.5483, 1017.6229, 1017.6229, 1019.6358, 1033.6520, 1047.4978, 1047.4978, 1051.6609, 1069.4803, 1133.5562, 1151.6812, 1165.6880, 1165.6880, 1174.5699, 1174.5700, 1179.5543, 1190.5620, 1194.5756, 1212.5294, 1269.6084, 1277.6251, 1300.7948, 1305.5947, 1311.6462, 1311.6462, 1334.8268, 1695.6990, 1702.7892, 1732.0278, 1839.8657, 2015.2070, 2283.1013, 2700.2822, 2700.2822

13. A0A4W2HIT4 **Mass:** 478985 **Score:** 45 **Expect:** 4.3 **Matches:** 30

Dynein axonemal heavy chain 6 OS=Bos indicus x Bos taurus OX=30522 GN=DNAH6 PE=4 SV=1

Observed	Mr(expt)	Mr(calc)	ppm	Start	End	Miss	Ions	Peptide
834.4748	833.4675	833.4647	3.40	1784	1790	0	---	K.TYVLNPK.S
848.4878	847.4805	847.4334	55.6	2431	2436	1	---	R.MIRQER.G + Oxidation (M)
864.5128	863.5055	863.4753	35.0	925	931	0	---	K.FVTQLEK.G
868.5118	867.5045	867.4464	67.1	649	654	1	---	K.YHRQHK.D
960.4399	959.4326	959.4672	-36.05	2545	2553	0	---	K.EVGISEGNR.D
977.4595	976.4522	976.5012	-50.11	1810	1818	0	---	K.DGLMALSVR.A + Oxidation (M)
977.4595	976.4523	976.5012	-50.08	1810	1818	0	---	K.DGLMALSVR.A + Oxidation (M)
1045.5190	1044.5117	1044.5451	-31.97	4024	4033	0	---	R.DQATVIEAAK.T
1131.6592	1130.6519	1130.5608	80.6	232	240	1	---	K.NDYYTISKK.A
1149.5057	1148.4984	1148.4855	11.2	2320	2327	0	---	R.LFCHECQR.V
1149.5057	1148.4985	1148.4855	11.3	2320	2327	0	---	R.LFCHECQR.V
1156.5675	1155.5602	1155.5746	-12.48	1551	1559	1	---	R.FMFEGREIK.L
1182.6278	1181.6205	1181.5750	38.5	1280	1289	0	---	K.VEEAMFTSLR.R
1182.6278	1181.6205	1181.5750	38.5	1280	1289	0	5	K.VEEAMFTSLR.R
1287.6068	1286.5995	1286.7207	-94.20	1205	1215	1	---	R.NPQAVQPHLRK.C
1287.6068	1286.5995	1286.7207	-94.19	1205	1215	1	---	R.NPQAVQPHLRK.C
1425.6982	1424.6909	1424.7088	-12.57	3381	3391	0	---	R.LTEAEWNFFLR.G
1439.7417	1438.7344	1438.6472	60.6	1889	1900	0	---	R.CGMVFVDPEELK.W + Oxidation (M)
1448.8599	1447.8526	1447.9238	-49.18	1355	1368	1	---	R.LNALAAIVRGILPK.L
1475.6879	1474.6806	1474.7701	-60.67	2903	2916	0	---	R.AAQAELDITMATLK.E
1528.7235	1527.7162	1527.8046	-57.81	1030	1042	1	---	K.TTEFVVLPHRDSK.D
1617.9976	1616.9903	1616.8596	80.9	1119	1132	1	---	R.QLPAEAKMFLQVDK.S
1664.7775	1663.7702	1663.9185	-89.12	3501	3515	0	---	K.VVFALTDFVIENLGK.A
1691.7629	1690.7556	1690.8414	-50.71	907	921	0	---	K.FDSLDPELLNSQVSK.Y

PDF-XChange Click to buy NOW! www.docu-track.com

PDF-XChange Click to buy NOW! www.docu-track.com

1880.9835	1879.9762	1879.9138	33.2	4139	4154	1	---	K.DYWIAKGSALLCQLNE.-
1901.1722	1900.1649	1900.0319	70.0	2415	2430	1	---	K.LVFFQDAIEHVSRIAR.M
1904.9435	1903.9362	1903.9778	-21.86	3865	3881	0	---	R.VPEILETENASESLFVK.D
2211.0264	2210.0191	2210.0677	-22.00	1271	1289	1	---	R.GNVEEWLGKVEEAMFTSLR.R + Oxidation (M)
2682.3079	2681.3006	2681.3871	-32.27	3192	3214	1	---	K.SGLEDQLLSDVVRLEKPELEEQR.I
2683.2788	2682.2715	2682.3725	-37.66	3046	3069	1	---	R.QWNTDGLPRDLISTENGILVTQGR.R

No match to: 842.4698, 850.0129, 850.4987, 853.4788, 862.4257, 865.9851, 870.5065, 876.9959, 882.5245, 882.5245, 892.9658, 951.5779, 996.5483, 1017.6229, 1017.6229, 1019.6358, 1033.6520, 1047.4978, 1047.4978, 1051.6609, 1069.4803, 1133.5562, 1151.6812, 1165.6880, 1165.6880, 1174.5699, 1174.5700, 1179.5543, 1190.5620, 1194.5756, 1212.5294, 1269.6084, 1277.6251, 1300.7948, 1305.5947, 1311.6462, 1311.6462, 1334.8268, 1695.6990, 1702.7892, 1732.0278, 1839.8657, 2015.2070, 2283.1013, 2700.2822, 2700.2822

14. A0A3Q1LWT9 **Mass:** 14058 **Score:** 44 **Expect:** 5.4 **Matches:** 7

Uncharacterized protein OS=Bos taurus OX=9913 PE=4 SV=1

Observed	Mr(expt)	Mr(calc)	ppm	Start	End	Miss	Ions	Peptide
862.4257	861.4184	861.4416	-26.96	96	104	1	---	R.GASASSRAR.V
977.4595	976.4522	976.4839	-32.39	94	102	1	---	R.WRGASASSR.A
977.4595	976.4523	976.4839	-32.36	94	102	1	15	R.WRGASASSR.A
1149.5057	1148.4984	1148.5682	-60.73	30	39	0	---	R.LDTAQMAMIR.Y
1149.5057	1148.4985	1148.5682	-60.69	30	39	0	2	R.LDTAQMAMIR.Y
1287.6068	1286.5995	1286.5739	20.0	18	29	0	---	R.SAVYSSSESTNR.L
1287.6068	1286.5995	1286.5739	20.0	18	29	0	---	R.SAVYSSSESTNR.L

No match to: 834.4748, 842.4698, 848.4878, 850.0129, 850.4987, 853.4788, 864.5128, 865.9851, 868.5118, 870.5065, 876.9959, 882.5245, 882.5245, 892.9658, 951.5779, 960.4399, 996.5483, 1017.6229, 1017.6229, 1019.6358, 1033.6520, 1045.5190, 1047.4978, 1047.4978, 1051.6609, 1069.4803, 1131.6592, 1133.5562, 1151.6812, 1156.5675, 1165.6880, 1165.6880, 1174.5699, 1174.5700, 1179.5543, 1182.6278, 1182.6278, 1190.5620, 1194.5756, 1212.5294, 1269.6084, 1277.6251, 1300.7948, 1305.5947, 1311.6462, 1311.6462, 1334.8268, 1425.6982, 1439.7417, 1448.8599, 1475.6879, 1528.7235, 1617.9976, 1664.7775, 1691.7629, 1695.6990, 1702.7892, 1732.0278, 1839.8657, 1880.9835, 1901.1722, 1904.9435, 2015.2070, 2211.0264, 2283.1013, 2682.3079, 2683.2788, 2700.2822, 2700.2822

15. A0A4W2DIE4 **Mass:** 14058 **Score:** 44 **Expect:** 5.4 **Matches:** 7

Uncharacterized protein OS=Bos indicus x Bos taurus OX=30522 PE=4 SV=1

Observed	Mr(expt)	Mr(calc)	ppm	Start	End	Miss	Ions	Peptide
862.4257	861.4184	861.4416	-26.96	96	104	1	---	R.GASASSRAR.V
977.4595	976.4522	976.4839	-32.39	94	102	1	---	R.WRGASASSR.A
977.4595	976.4523	976.4839	-32.36	94	102	1	15	R.WRGASASSR.A
1149.5057	1148.4984	1148.5682	-60.73	30	39	0	---	R.LDTAQMAMIR.Y
1149.5057	1148.4985	1148.5682	-60.69	30	39	0	2	R.LDTAQMAMIR.Y
1287.6068	1286.5995	1286.5739	20.0	18	29	0	---	R.SAVYSSSESTNR.L
1287.6068	1286.5995	1286.5739	20.0	18	29	0	---	R.SAVYSSSESTNR.L

No match to: 834.4748, 842.4698, 848.4878, 850.0129, 850.4987, 853.4788, 864.5128, 865.9851, 868.5118, 870.5065, 876.9959, 882.5245, 882.5245, 892.9658, 951.5779, 960.4399, 996.5483, 1017.6229, 1017.6229, 1019.6358, 1033.6520, 1045.5190, 1047.4978, 1047.4978, 1051.6609, 1069.4803, 1131.6592, 1133.5562, 1151.6812, 1156.5675, 1165.6880, 1165.6880, 1174.5699, 1174.5700, 1179.5543, 1182.6278, 1182.6278, 1190.5620, 1194.5756, 1212.5294, 1269.6084, 1277.6251, 1300.7948, 1305.5947, 1311.6462, 1311.6462, 1334.8268, 1425.6982, 1439.7417, 1448.8599, 1475.6879, 1528.7235, 1617.9976, 1664.7775, 1691.7629, 1695.6990, 1702.7892, 1732.0278, 1839.8657, 1880.9835, 1901.1722, 1904.9435, 2015.2070, 2211.0264, 2283.1013, 2682.3079, 2683.2788, 2700.2822, 2700.2822

16. A0A4W2EFU9 **Mass:** 24150 **Score:** 44 **Expect:** 5.5 **Matches:** 9

Hypoxanthine phosphoribosyltransferase OS=Bos indicus x Bos taurus OX=30522 PE=3 SV=1

Observed	Mr(expt)	Mr(calc)	ppm	Start	End	Miss	Ions	Peptide
848.4878	847.4805	847.4586	25.9	49	55	1	---	R.LARDVMK.E + Oxidation (M)
882.5245	881.5172	881.4395	88.1	74	80	0	---	K.FFADLNR.N
882.5245	881.5173	881.4395	88.2	74	80	0	---	K.FFADLNR.N
1133.5562	1132.5489	1132.6526	-91.51	135	144	0	---	K.TMQTLLSLVK.K
1194.5756	1193.5683	1193.6115	-36.13	85	94	0	---	K.SIPMTVDFIR.L + Oxidation (M)
1277.6251	1276.6178	1276.7424	-97.61	135	145	1	---	K.TMQTLLSLVKK.H + Oxidation (M)
1287.6068	1286.5995	1286.6408	-32.05	70	80	1	---	K.GGYKFFADLNR.N
1287.6068	1286.5995	1286.6408	-32.04	70	80	1	---	K.GGYKFFADLNR.N
2682.3079	2681.3006	2681.1738	47.3	1	24	0	---	-.MAAHSPSVVNSNDEPGYDLDLFCK.T + Oxidation (M)

No match to: 834.4748, 842.4698, 850.0129, 850.4987, 853.4788, 862.4257, 864.5128, 865.9851, 868.5118, 870.5065, 876.9959, 892.9658, 951.5779, 960.4399, 977.4595, 977.4595, 996.5483, 1017.6229, 1017.6229, 1019.6358, 1033.6520, 1045.5190, 1047.4978, 1047.4978, 1051.6609, 1069.4803, 1131.6592, 1149.5057, 1149.5057, 1151.6812, 1156.5675, 1165.6880, 1165.6880, 1174.5699, 1174.5700, 1179.5543, 1182.6278, 1182.6278, 1190.5620, 1212.5294, 1269.6084, 1300.7948, 1305.5947, 1311.6462, 1311.6462, 1334.8268, 1425.6982, 1439.7417, 1448.8599, 1475.6879, 1528.7235, 1617.9976, 1664.7775, 1691.7629, 1695.6990, 1702.7892, 1732.0278, 1839.8657, 1880.9835, 1901.1722, 1904.9435, 2015.2070, 2211.0264, 2283.1013, 2683.2788, 2700.2822, 2700.2822

17. E1BA06 **Mass:** 24150 **Score:** 44 **Expect:** 5.5 **Matches:** 9

Hypoxanthine phosphoribosyltransferase OS=Bos taurus OX=9913 PE=3 SV=2

Observed	Mr(expt)	Mr(calc)	ppm	Start	End	Miss	Ions	Peptide
848.4878	847.4805	847.4586	25.9	49	55	1	---	R.LARDVMK.E + Oxidation (M)
882.5245	881.5172	881.4395	88.1	74	80	0	---	K.FFADLNR.N
882.5245	881.5173	881.4395	88.2	74	80	0	---	K.FFADLNR.N
1133.5562	1132.5489	1132.6526	-91.51	135	144	0	---	K.TMQTLLSLVK.K
1194.5756	1193.5683	1193.6115	-36.13	85	94	0	---	K.SIPMTVDFIR.L + Oxidation (M)
1277.6251	1276.6178	1276.7424	-97.61	135	145	1	---	K.TMQTLLSLVKK.H + Oxidation (M)
1287.6068	1286.5995	1286.6408	-32.05	70	80	1	---	K.GGYKFFADLNR.N
1287.6068	1286.5995	1286.6408	-32.04	70	80	1	---	K.GGYKFFADLNR.N
2682.3079	2681.3006	2681.1738	47.3	1	24	0	---	-.MAAHSPSVVNSNDEPGYDLDLFCK.T + Oxidation (M)

No match to: 834.4748, 842.4698, 850.0129, 850.4987, 853.4788, 862.4257, 864.5128, 865.9851, 868.5118, 870.5065, 876.9959, 892.9658, 951.5779, 960.4399, 977.4595, 977.4595, 996.5483, 1017.6229, 1017.6229, 1019.6358, 1033.6520, 1045.5190, 1047.4978, 1047.4978, 1051.6609, 1069.4803, 1131.6592, 1149.5057, 1149.5057, 1151.6812, 1156.5675, 1165.6880, 1165.6880, 1174.5699, 1174.5700, 1179.5543, 1182.6278, 1182.6278, 1190.5620, 1212.5294, 1269.6084, 1300.7948, 1305.5947, 1311.6462, 1311.6462, 1334.8268, 1425.6982, 1439.7417, 1448.8599, 1475.6879, 1528.7235, 1617.9976, 1664.7775, 1691.7629, 1695.6990, 1702.7892, 1732.0278, 1839.8657, 1880.9835, 1901.1722, 1904.9435, 2015.2070, 2211.0264, 2283.1013, 2683.2788, 2700.2822, 2700.2822

18. A0A4W2HDP5 **Mass:** 60337 **Score:** 43 **Expect:** 6.6 **Matches:** 8

Zinc finger protein 382 OS=Bos indicus x Bos taurus OX=30522 GN=ZNF382 PE=4 SV=1

Observed	Mr(expt)	Mr(calc)	ppm	Start	End	Miss	Ions	Peptide
1045.5190	1044.5117	1044.5352	-22.49	262	269	0	---	K.SYLIEHQR.I
1047.4978	1046.4905	1046.5331	-40.71	234	241	0	---	K.SIFIMHQR.S + Oxidation (M)
1047.4978	1046.4905	1046.5331	-40.71	234	241	0	---	K.SIFIMHQR.S + Oxidation (M)
1051.6609	1050.6536	1050.5934	57.3	150	157	1	---	K.QELIQHRK.V
1151.6812	1150.6739	1150.5838	78.3	1	9	1	---	-.MTKPDMIRK.L + 2 Oxidation (M)
1182.6278	1181.6205	1181.6880	-57.13	78	87	1	---	K.NHIISKTTLR.E
1182.6278	1181.6205	1181.6880	-57.12	78	87	1	24	K.NHIISKTTLR.E

PDF-XChange Click to buy NOW! www.docu-track.com

Observed	Mr(expt)	Mr(calc)	ppm	Start	End	Miss	Ions	Peptide
1194.5756	1193.5683	1193.6193	-42.69	219 -	228	1	---	K.KPHSYSKYGK.F

No match to: 834.4748, 842.4698, 848.4878, 850.0129, 850.4987, 853.4788, 862.4257, 864.5128, 865.9851, 868.5118, 870.5065, 876.9959, 882.5245, 882.5245, 892.9658, 951.5779, 960.4399, 977.4595, 977.4595, 996.5483, 1017.6229, 1017.6229, 1019.6358, 1033.6520, 1069.4803, 1131.6592, 1133.5562, 1149.5057, 1149.5057, 1156.5675, 1165.6880, 1165.6880, 1174.5699, 1174.5700, 1179.5543, 1190.5620, 1212.5294, 1269.6084, 1277.6251, 1287.6068, 1287.6068, 1300.7948, 1305.5947, 1311.6462, 1311.6462, 1334.8268, 1425.6982, 1439.7417, 1448.8599, 1475.6879, 1528.7235, 1617.9976, 1664.7775, 1691.7629, 1695.6990, 1702.7892, 1732.0278, 1839.8657, 1880.9835, 1901.1722, 1904.9435, 2015.2070, 2211.0264, 2283.1013, 2682.3079, 2683.2788, 2700.2822, 2700.2822

19. A0A4W2FFL4 **Mass:** 343058 **Score:** 43 **Expect:** 6.9 **Matches:** 21

Kalirin RhoGEF kinase OS=Bos indicus x Bos taurus OX=30522 GN=KALRN PE=4 SV=1

Observed	Mr(expt)	Mr(calc)	ppm	Start	End	Miss	Ions	Peptide
834.4748	833.4675	833.3879	95.5	723 -	730	0	---	R.DSAVSNNK.T
848.4878	847.4805	847.4440	43.1	1212 -	1218	0	---	K.WVTTVDK.H
951.5779	950.5706	950.5410	31.2	618 -	625	1	---	K.AARHLEVR.I
996.5483	995.5410	995.5036	37.6	138 -	146	1	---	K.QKTNFGSSK.F
1017.6229	1016.6156	1016.6019	13.5	765 -	772	0	---	K.LDIFLQLR.I
1017.6229	1016.6156	1016.6019	13.6	765 -	772	0	---	K.LDIFLQLR.I
1033.6520	1032.6447	1032.5465	95.2	1202 -	1210	0	---	K.GHIHATEIR.K
1051.6609	1050.6536	1050.5532	95.6	93 -	101	0	---	R.GFTVIIDMR.G
1156.5675	1155.5602	1155.6360	-65.56	1574 -	1582	1	---	K.NIREVIQER.I
1174.5699	1173.5626	1173.5713	-7.40	2400 -	2408	1	---	K.KSCSWHTLR.M
1174.5700	1173.5627	1173.5713	-7.35	2400 -	2408	1	16	K.KSCSWHTLR.M
1269.6084	1268.6011	1268.6765	-59.39	2279 -	2290	0	---	K.GSSYNPPLPPLK.I
1448.8599	1447.8526	1447.7493	71.3	411 -	423	0	---	R.STILAMSAVFHQK.A + Oxidation (M)
1664.7775	1663.7702	1663.8682	-58.88	2914 -	2926	1	---	R.DFINVILQEDFRR.R
1702.7892	1701.7819	1701.9274	-85.49	2566 -	2581	0	---	K.VQGVPAAPNRPIAQER.S
1839.8657	1838.8584	1838.8291	15.9	1900 -	1916	0	---	K.DPTGCLNEGMTPPTPPR.N
1880.9835	1879.9762	1879.8337	75.8	2291 -	2308	0	---	K.ISTSNGSPGFDYHQAGDK.F
1904.9435	1903.9362	1903.9879	-27.15	2014 -	2027	1	---	R.KLHIYVWYCQNKPR.S
2683.2788	2682.2715	2682.2781	-2.47	2177 -	2198	1	---	K.MNYLVLEENVDNDPCKFALLNR.E + Oxidation (M)
2700.2822	2699.2749	2699.3303	-20.52	1378 -	1400	0	---	K.NKPDSNQLILEHAGTFFDEIQQR.H
2700.2822	2699.2750	2699.3303	-20.51	1378 -	1400	0	0	K.NKPDSNQLILEHAGTFFDEIQQR.H

No match to: 842.4698, 850.0129, 850.4987, 853.4788, 862.4257, 864.5128, 865.9851, 868.5118, 870.5065, 876.9959, 882.5245, 882.5245, 892.9658, 960.4399, 977.4595, 977.4595, 1019.6358, 1045.5190, 1047.4978, 1047.4978, 1069.4803, 1131.6592, 1133.5562, 1149.5057, 1149.5057, 1151.6812, 1165.6880, 1165.6880, 1179.5543, 1182.6278, 1182.6278, 1190.5620, 1194.5756, 1212.5294, 1277.6251, 1287.6068, 1287.6068, 1300.7948, 1305.5947, 1311.6462, 1311.6462, 1334.8268, 1425.6982, 1439.7417, 1475.6879, 1528.7235, 1617.9976, 1691.7629, 1695.6990, 1732.0278, 1901.1722, 2015.2070, 2211.0264, 2283.1013, 2682.3079

20. A0A4W2EN51 **Mass:** 85445 **Score:** 43 **Expect:** 7.2 **Matches:** 8

Cyclin and CBS domain divalent metal cation transport mediator 4 OS=Bos indicus x Bos taurus OX=30522 GN=CNNM4 PE=4 SV=1

Observed	Mr(expt)	Mr(calc)	ppm	Start	End	Miss	Ions	Peptide
1047.4978	1046.4905	1046.5179	-26.14	216 -	224	0	---	R.IVQNCGTQK.E
1047.4978	1046.4905	1046.5179	-26.14	216 -	224	0	---	R.IVQNCGTQK.E
1174.5699	1173.5626	1173.5989	-30.94	710 -	721	0	---	R.SLAAAEGADTLR.A
1174.5700	1173.5627	1173.5989	-30.90	710 -	721	0	31	R.SLAAAEGADTLR.A
1287.6068	1286.5995	1286.6520	-40.77	601 -	610	0	---	R.LASHHYLYQR.S
1287.6068	1286.5995	1286.6520	-40.76	601 -	610	0	---	R.LASHHYLYQR.S
1439.7417	1438.7344	1438.7099	17.0	1 -	16	0	---	-.MAPVGGGGRPGSGPAR.G + Oxidation (M)
1904.9435	1903.9362	1904.0401	-54.56	120 -	137	1	---	K.LVNVSRGDTSGMLVVLTK.F + Oxidation (M)

No match to: 834.4748, 842.4698, 848.4878, 850.0129, 850.4987, 853.4788, 862.4257, 864.5128, 865.9851, 868.5118, 870.5065, 876.9959, 882.5245, 882.5245, 892.9658, 951.5779, 960.4399, 977.4595, 977.4595, 996.5483, 1017.6229, 1017.6229, 1019.6358, 1033.6520, 1045.5190, 1051.6609, 1069.4803, 1131.6592, 1133.5562, 1149.5057, 1149.5057, 1151.6812, 1156.5675, 1165.6880, 1165.6880, 1179.5543, 1182.6278, 1182.6278, 1190.5620, 1194.5756, 1212.5294, 1269.6084, 1277.6251, 1300.7948, 1305.5947, 1311.6462, 1311.6462, 1334.8268, 1425.6982, 1448.8599, 1475.6879, 1528.7235, 1617.9976, 1664.7775, 1691.7629, 1695.6990, 1702.7892, 1732.0278, 1839.8657, 1880.9835, 1901.1722, 2015.2070, 2211.0264, 2283.1013, 2682.3079, 2683.2788, 2700.2822, 2700.2822

21. A0A4W2F4Z6 **Mass:** 60709 **Score:** 42 **Expect:** 8.1 **Matches:** 14

Uncharacterized protein OS=Bos indicus x Bos taurus OX=30522 PE=4 SV=1

Observed	Mr(expt)	Mr(calc)	ppm	Start	End	Miss	Ions	Peptide
848.4878	847.4805	847.4188	72.8	521 -	528	1	---	K.SRSSFPPA.-
862.4257	861.4184	861.4742	-64.76	411 -	417	1	---	K.MQKTQVK.C
1017.6229	1016.6156	1016.5729	42.1	162 -	170	0	---	K.TLLMPFAPK.E
1017.6229	1016.6156	1016.5729	42.1	162 -	170	0	---	K.TLLMPFAPK.E
1033.6520	1032.6447	1032.5678	74.5	162 -	170	0	---	K.TLLMPFAPK.E + Oxidation (M)
1045.5190	1044.5117	1044.5280	-15.61	381 -	389	0	---	R.SFYSFGPLK.S
1156.5675	1155.5602	1155.5812	-18.13	357 -	365	0	---	R.EEVTFIYQK.L
1174.5699	1173.5626	1173.5414	18.1	171 -	180	0	---	K.EEGHYLSPSR.S
1174.5700	1173.5627	1173.5414	18.1	171 -	180	0	---	K.EEGHYLSPSR.S
1182.6278	1181.6205	1181.5057	97.2	224 -	233	0	---	K.STSMIPDCQK.L + Oxidation (M)
1182.6278	1181.6205	1181.5057	97.2	224 -	233	0	---	K.STSMIPDCQK.L + Oxidation (M)
1311.6462	1310.6389	1310.6367	1.66	291 -	301	1	---	K.EGHKWDVVEGR.Q
1311.6462	1310.6390	1310.6295	7.20	92 -	101	0	---	K.TLEYTFHWSK.D
1425.6982	1424.6909	1424.7663	-52.93	357 -	367	1	---	R.EEVTFIYQKLR.E

No match to: 834.4748, 842.4698, 850.0129, 850.4987, 853.4788, 864.5128, 865.9851, 868.5118, 870.5065, 876.9959, 882.5245, 882.5245, 892.9658, 951.5779, 960.4399, 977.4595, 977.4595, 996.5483, 1019.6358, 1047.4978, 1047.4978, 1051.6609, 1069.4803, 1131.6592, 1133.5562, 1149.5057, 1149.5057, 1151.6812, 1165.6880, 1165.6880, 1179.5543, 1190.5620, 1194.5756, 1212.5294, 1269.6084, 1277.6251, 1287.6068, 1287.6068, 1300.7948, 1305.5947, 1334.8268, 1439.7417, 1448.8599, 1475.6879, 1528.7235, 1617.9976, 1664.7775, 1691.7629, 1695.6990, 1702.7892, 1732.0278, 1839.8657, 1880.9835, 1901.1722, 1904.9435, 2015.2070, 2211.0264, 2283.1013, 2682.3079, 2683.2788, 2700.2822, 2700.2822

22. A0A4W2HZ94 **Mass:** 170798 **Score:** 42 **Expect:** 8.5 **Matches:** 22

Lysine demethylase 2B OS=Bos indicus x Bos taurus OX=30522 GN=KDM2B PE=4 SV=1

Observed	Mr(expt)	Mr(calc)	ppm	Start	End	Miss	Ions	Peptide
842.4698	841.4625	841.4698	-8.61	18 -	24	0	---	R.LPVEWAK.L
868.5118	867.5045	867.4199	97.6	925 -	931	1	---	R.RSDEHPK.K
977.4595	976.4522	976.3920	61.7	916 -	923	0	---	R.SECEEAPR.R
977.4595	976.4523	976.3920	61.7	916 -	923	0	9	R.SECEEAPR.R
1047.4978	1046.4905	1046.5066	-15.38	471 -	479	0	---	R.ESMLIDAPR.K + Oxidation (M)
1047.4978	1046.4905	1046.5066	-15.38	471 -	479	0	0	R.ESMLIDAPR.K + Oxidation (M)
1069.4803	1068.4730	1068.4878	-13.85	730 -	737	1	---	R.MKQSCIMR.Q + Oxidation (M)
1133.5562	1132.5489	1132.4931	49.3	916 -	924	1	---	R.SECEEAPRR.R
1156.5675	1155.5602	1155.5196	35.1	243 -	251	0	---	R.YYETPEAQR.D
1165.6880	1164.6807	1164.6437	31.7	109 -	118	0	---	R.RPPVPPIMSR.A + Oxidation (M)

PDF-XChange Click to buy NOW! www.docu-track.com

1165.6880	1164.6807	1164.6437	31.8	109	118	0	---	R.RPPVPPIMSR.A + Oxidation (M)
1194.5756	1193.5683	1193.6040	-29.91	425	433	1	---	R.IYEIEDRTR.V
1277.6251	1276.6178	1276.6333	-12.14	221	231	0	---	R.LVDVMDVNTQK.G + Oxidation (M)
1287.6068	1286.5995	1286.6871	-68.03	1325	1335	0	---	R.TLDVQWVEGLK.D
1287.6068	1286.5995	1286.6871	-68.02	1325	1335	0	---	R.TLDVQWVEGLK.D
1300.7948	1299.7875	1299.7584	22.4	1260	1271	0	---	K.SITPLMLSGIIR.R
1311.6462	1310.6389	1310.7129	-56.44	83	95	1	---	K.VAPVPKVGCTGAR.G
1311.6462	1310.6390	1310.7129	-56.41	83	95	1	---	K.VAPVPKVGCTGAR.G
1334.8268	1333.8195	1333.6990	90.4	954	964	1	---	K.YEKPQELSGRK.R
1702.7892	1701.7819	1701.8111	-17.11	175	187	1	---	K.DFNYEYVQREALR.V
1904.9435	1903.9362	1903.9462	-5.24	1325	1340	1	---	R.TLDVQWVEGLKDAQMR.D + Oxidation (M)
2683.2788	2682.2715	2682.4817	-78.36	819	846	0	---	K.VSAGLQVPGVSPGGVDLGLARPSLSPPR.R

No match to: 834.4748, 848.4878, 850.0129, 850.4987, 853.4788, 862.4257, 864.5128, 865.9851, 870.5065, 876.9959, 882.5245, 882.5245, 892.9658, 951.5779, 960.4399, 996.5483, 1017.6229, 1017.6229, 1019.6358, 1033.6520, 1045.5190, 1051.6609, 1131.6592, 1149.5057, 1149.5057, 1151.6812, 1174.5699, 1174.5700, 1179.5543, 1182.6278, 1182.6278, 1190.5620, 1212.5294, 1269.6084, 1305.5947, 1425.6982, 1439.7417, 1448.8599, 1475.6879, 1528.7235, 1617.9976, 1664.7775, 1691.7629, 1695.6990, 1732.0278, 1839.8657, 1880.9835, 1901.1722, 2015.2070, 2211.0264, 2283.1013, 2682.3079, 2700.2822, 2700.2822

23. A0A3Q1MCG0 **Mass:** 472745 **Score:** 42 **Expect:** 9.5 **Matches:** 29

Dynein axonemal heavy chain 6 OS=Bos taurus OX=9913 GN=DNAH6 PE=4 SV=1

Observed	Mr(expt)	Mr(calc)	ppm	Start	End	Miss	Ions	Peptide
834.4748	833.4675	833.4647	3.40	1732	1738	0	---	K.TYVLNPK.S
848.4878	847.4805	847.4334	55.6	2379	2384	1	---	R.MIRQER.G + Oxidation (M)
864.5128	863.5055	863.4753	35.0	925	931	0	---	K.FVTQLEK.G
868.5118	867.5045	867.4464	67.1	649	654	1	---	K.YHRQHK.D
960.4399	959.4326	959.4672	-36.05	2493	2501	0	---	K.EVGISEGNR.D
977.4595	976.4522	976.5012	-50.11	1758	1766	0	---	K.DGLMALSVR.A + Oxidation (M)
977.4595	976.4523	976.5012	-50.08	1758	1766	0	---	K.DGLMALSVR.A + Oxidation (M)
1045.5190	1044.5117	1044.5451	-31.97	3972	3981	0	---	R.DQATVIEAAK.T
1131.6592	1130.6519	1130.5608	80.6	232	240	1	---	K.NDYYTISKK.A
1149.5057	1148.4984	1148.4855	11.2	2268	2275	0	---	R.LFCHECQR.V
1149.5057	1148.4985	1148.4855	11.3	2268	2275	0	---	R.LFCHECQR.V
1156.5675	1155.5602	1155.5746	-12.48	1499	1507	1	---	R.FMFEGREIK.L
1182.6278	1181.6205	1181.5750	38.5	1280	1289	0	---	K.VEEAMFTSLR.R
1182.6278	1181.6205	1181.5750	38.5	1280	1289	0	5	K.VEEAMFTSLR.R
1287.6068	1286.5995	1286.7207	-94.20	1205	1215	1	---	R.NPQAVQPHLRK.C
1287.6068	1286.5995	1286.7207	-94.19	1205	1215	1	---	R.NPQAVQPHLRK.C
1425.6982	1424.6909	1424.7088	-12.57	3329	3339	0	---	R.LTEAEWNFFLR.G
1439.7417	1438.7344	1438.6472	60.6	1837	1848	0	---	R.CGMVFVDPEELK.W + Oxidation (M)
1448.8599	1447.8526	1447.9238	-49.18	1355	1368	1	---	R.LNALAAIVRGILPK.L
1475.6879	1474.6806	1474.7701	-60.67	2851	2864	0	---	R.AAQAELDITMATLK.E
1528.7235	1527.7162	1527.8046	-57.81	1030	1042	1	---	K.TTEFVVLPHRDSK.D
1617.9976	1616.9903	1616.8596	80.9	1119	1132	1	---	R.QLPAEAKMFLQVDK.S
1664.7775	1663.7702	1663.9185	-89.12	3449	3463	0	---	K.VVFALTDFVIENLGK.A
1691.7629	1690.7556	1690.8414	-50.71	907	921	0	---	K.FDSLDPELLNSQVSK.Y
1880.9835	1879.9762	1879.9138	33.2	4087	4102	1	---	K.DYWIAKGSALLCQLNE.-
1901.1722	1900.1649	1900.0319	70.0	2363	2378	1	---	K.LVFFQDAIEHVSRIAR.M
2211.0264	2210.0191	2210.0677	-22.00	1271	1289	1	---	R.GNVEEWLGKVEEAMFTSLR.R + Oxidation (M)
2682.3079	2681.3006	2681.3871	-32.27	3140	3162	1	---	K.SGLEDQLLSDVVRLEKPELEEQR.I
2683.2788	2682.2715	2682.3725	-37.66	2994	3017	1	---	R.QWNTDGLPRDLISTENGILVTQGR.R

No match to: 842.4698, 850.0129, 850.4987, 853.4788, 862.4257, 865.9851, 870.5065, 876.9959, 882.5245, 882.5245, 892.9658, 951.5779, 996.5483, 1017.6229, 1017.6229, 1019.6358, 1033.6520, 1047.4978, 1047.4978, 1051.6609, 1069.4803, 1133.5562, 1151.6812, 1165.6880, 1174.5699, 1174.5700, 1179.5543, 1190.5620, 1194.5756, 1212.5294, 1269.6084, 1277.6251, 1300.7948, 1305.5947, 1311.6462, 1311.6462, 1334.8268, 1695.6990, 1702.7892, 1732.0278, 1839.8657, 1904.9435, 2015.2070, 2283.1013, 2700.2822, 2700.2822

24. A0A4W2GJX2 **Mass:** 108528 **Score:** 40 **Expect:** 12 **Matches:** 17

Nebulette OS=Bos indicus x Bos taurus OX=30522 GN=NEBL PE=4 SV=1

Observed	Mr(expt)	Mr(calc)	ppm	Start	End	Miss	Ions	Peptide
864.5128	863.5055	863.5116	-7.07	329	335	0	---	K.YITNLLK.E
960.4399	959.4326	959.4746	-43.74	792	799	0	---	R.SLHMLSEK.A + Oxidation (M)
996.5483	995.5410	995.4923	48.9	825	832	0	---	K.SEISEIYR.S
1017.6229	1016.6156	1016.5251	89.1	544	552	0	---	K.NQQNISSVK.Y
1017.6229	1016.6156	1016.5251	89.1	544	552	0	---	K.NQQNISSVK.Y
1045.5190	1044.5117	1044.5451	-31.96	453	461	1	---	K.DLENEIKGK.G
1069.4803	1068.4730	1068.5134	-37.83	876	884	0	---	R.SMQHSPNLR.T
1179.5543	1178.5470	1178.4575	76.0	18	27	0	---	K.TGEEENEEDK.V
1269.6084	1268.6011	1268.6612	-47.36	621	632	0	---	K.QATAISDPPELK.R
1287.6068	1286.5995	1286.5812	14.2	441	451	1	---	K.ASEMASQKEYK.K + Oxidation (M)
1287.6068	1286.5995	1286.5812	14.2	441	451	1	---	K.ASEMASQKEYK.K + Oxidation (M)
1305.5947	1304.5874	1304.6296	-32.30	750	760	0	---	K.GVHPHIVEMDR.R + Oxidation (M)
1425.6982	1424.6909	1424.7623	-50.11	621	633	1	---	K.QATAISDPPELKR.V
1664.7775	1663.7702	1663.8199	-29.88	423	437	1	---	R.GKGMQVSTDTLDIQR.A + Oxidation (M)
1691.7629	1690.7556	1690.8204	-38.34	462	476	1	---	K.GMQVSMDIPDMLRAK.R
1702.7892	1701.7819	1701.8719	-52.89	351	365	1	---	K.GMELNSEVLDIQRAK.R
2682.3079	2681.3006	2681.3231	-8.40	200	224	1	---	K.GQGVMNKEPAVIGRPDFEHAVEASK.L + Oxidation (M)

No match to: 834.4748, 842.4698, 848.4878, 850.0129, 850.4987, 853.4788, 862.4257, 865.9851, 868.5118, 870.5065, 876.9959, 882.5245, 882.5245, 892.9658, 951.5779, 977.4595, 977.4595, 1019.6358, 1033.6520, 1047.4978, 1047.4978, 1051.6609, 1131.6592, 1133.5562, 1149.5057, 1149.5057, 1151.6812, 1156.5675, 1165.6880, 1165.6880, 1174.5699, 1174.5700, 1182.6278, 1182.6278, 1190.5620, 1194.5756, 1212.5294, 1277.6251, 1300.7948, 1311.6462, 1311.6462, 1334.8268, 1439.7417, 1448.8599, 1475.6879, 1528.7235, 1617.9976, 1695.6990, 1732.0278, 1839.8657, 1880.9835, 1901.1722, 1904.9435, 2015.2070, 2211.0264, 2283.1013, 2683.2788, 2700.2822, 2700.2822

25. A0A4W2EAZ9 **Mass:** 473215 **Score:** 40 **Expect:** 13 **Matches:** 29

Dynein axonemal heavy chain 6 OS=Bos indicus x Bos taurus OX=30522 GN=DNAH6 PE=4 SV=1

Observed	Mr(expt)	Mr(calc)	ppm	Start	End	Miss	Ions	Peptide
834.4748	833.4675	833.4647	3.40	1784	1790	0	---	K.TYVLNPK.S
848.4878	847.4805	847.4334	55.6	2431	2436	1	---	R.MIRQER.G + Oxidation (M)
864.5128	863.5055	863.4753	35.0	925	931	0	---	K.FVTQLEK.G
868.5118	867.5045	867.4464	67.1	649	654	1	---	K.YHRQHK.D
960.4399	959.4326	959.4672	-36.05	2545	2553	0	---	K.EVGISEGNR.D
977.4595	976.4522	976.5012	-50.11	1810	1818	0	---	K.DGLMALSVR.A + Oxidation (M)
977.4595	976.4523	976.5012	-50.08	1810	1818	0	---	K.DGLMALSVR.A + Oxidation (M)
1045.5190	1044.5117	1044.5451	-31.97	3977	3986	0	---	R.DQATVIEAAK.T

PDF-XChange Click to buy NOW! www.docu-track.com

1131.6592	1130.6519	1130.5608	80.6	232	240	1	---	K.NDYYTISKK.A
1149.5057	1148.4984	1148.4855	11.2	2320	2327	0	---	R.LFCHECQR.V
1149.5057	1148.4985	1148.4855	11.3	2320	2327	0	---	R.LFCHECQR.V
1156.5675	1155.5602	1155.5746	-12.48	1551	1559	1	---	R.FMFEGREIK.L
1182.6278	1181.6205	1181.5750	38.5	1280	1289	0	---	K.VEEAMFTSLR.R
1182.6278	1181.6205	1181.5750	38.5	1280	1289	0	5	K.VEEAMFTSLR.R
1287.6068	1286.5995	1286.7207	-94.20	1205	1215	1	---	R.NPQAVQPHLRK.C
1287.6068	1286.5995	1286.7207	-94.19	1205	1215	1	---	R.NPQAVQPHLRK.C
1425.6982	1424.6909	1424.7088	-12.57	3334	3344	0	---	R.LTEAEWNFFLR.G
1439.7417	1438.7344	1438.6472	60.6	1889	1900	0	---	R.CGMVFVDPEELK.W + Oxidation (M)
1448.8599	1447.8526	1447.9238	-49.18	1355	1368	1	---	R.LNALAAIVRGILPK.L
1475.6879	1474.6806	1474.7701	-60.67	2856	2869	0	---	R.AAQAELDITMATLK.E
1528.7235	1527.7162	1527.8046	-57.81	1030	1042	1	---	K.TTEFVVLPHRDSK.D
1617.9976	1616.9903	1616.8596	80.9	1119	1132	1	---	R.QLPAEAKMFLQVDK.S
1664.7775	1663.7702	1663.9185	-89.12	3454	3468	0	---	K.VVFALTDFVIENLGK.A
1691.7629	1690.7556	1690.8414	-50.71	907	921	0	---	K.FDSLDPELLNSQVSK.Y
1880.9835	1879.9762	1879.9138	33.2	4092	4107	1	---	K.DYWIAKGSALLCQLNE.-
1901.1722	1900.1649	1900.0319	70.0	2415	2430	1	---	K.LVFFQDAIEHVSRIAR.M
2211.0264	2210.0191	2210.0677	-22.00	1271	1289	1	---	R.GNVEEWLGKVEEAMFTSLR.R + Oxidation (M)
2682.3079	2681.3006	2681.3871	-32.27	3145	3167	1	---	K.SGLEDQLLSDVVRLEKPELEEQR.I
2683.2788	2682.2715	2682.3725	-37.66	2999	3022	1	---	R.QWNTDGLPRDLISTENGILVTQGR.R

No match to: 842.4698, 850.0129, 850.4987, 853.4788, 862.4257, 865.9851, 870.5065, 876.9959, 882.5245, 882.5245, 892.9658, 951.5779, 996.5483, 1017.6229, 1017.6229, 1019.6358, 1033.6520, 1047.4978, 1047.4978, 1051.6609, 1069.4803, 1133.5562, 1151.6812, 1165.6880, 1165.6880, 1174.5699, 1174.5700, 1179.5543, 1190.5620, 1194.5756, 1212.5294, 1269.6084, 1277.6251, 1300.7948, 1305.5947, 1311.6462, 1311.6462, 1334.8268, 1695.6990, 1702.7892, 1732.0278, 1839.8657, 1904.9435, 2015.2070, 2283.1013, 2700.2822, 2700.2822

26. A0A4W2CJR0 **Mass:** 108457 **Score:** 40 **Expect:** 13 **Matches:** 17
Nebulette OS=Bos indicus x Bos taurus OX=30522 GN=NEBL PE=4 SV=1

Observed	Mr(expt)	Mr(calc)	ppm	Start	End	Miss	Ions	Peptide
864.5128	863.5055	863.5116	-7.07	329	335	0	---	K.YITNLLK.E
960.4399	959.4326	959.4746	-43.74	792	799	0	---	R.SLHMLSEK.A + Oxidation (M)
996.5483	995.5410	995.4923	48.9	825	832	0	---	K.SEISEIYR.S
1017.6229	1016.6156	1016.5251	89.1	544	552	0	---	K.NQQNISSVK.Y
1017.6229	1016.6156	1016.5251	89.1	544	552	0	---	K.NQQNISSVK.Y
1045.5190	1044.5117	1044.5451	-31.96	453	461	1	---	K.DLENEIKGK.G
1069.4803	1068.4730	1068.5134	-37.83	876	884	0	---	R.SMQHSPNLR.T
1179.5543	1178.5470	1178.4575	76.0	18	27	0	---	K.TGEEENEEDK.V
1269.6084	1268.6011	1268.6612	-47.36	621	632	0	---	K.QATAISDPPELK.R
1287.6068	1286.5995	1286.5812	14.2	441	451	1	---	K.ASEMASQKEYK.K + Oxidation (M)
1287.6068	1286.5995	1286.5812	14.2	441	451	1	---	K.ASEMASQKEYK.K + Oxidation (M)
1305.5947	1304.5874	1304.6296	-32.30	750	760	0	---	K.GVHPHIVEMDR.R + Oxidation (M)
1425.6982	1424.6909	1424.7623	-50.11	621	633	1	---	K.QATAISDPPELKR.V
1664.7775	1663.7702	1663.8199	-29.88	423	437	1	---	R.GKGMQVSTDTLDIQR.A + Oxidation (M)
1691.7629	1690.7556	1690.8204	-38.34	462	476	1	---	K.GMQVSMDIPDMLRAK.R
1702.7892	1701.7819	1701.8719	-52.89	351	365	1	---	K.GMELNSEVLDIQRAK.R
2682.3079	2681.3006	2681.3231	-8.40	200	224	1	---	K.GQGVMNKEPAVIGRPDFEHAVEASK.L + Oxidation (M)

No match to: 834.4748, 842.4698, 848.4878, 850.0129, 850.4987, 853.4788, 862.4257, 865.9851, 868.5118, 870.5065, 876.9959, 882.5245, 882.5245, 892.9658, 951.5779, 977.4595, 977.4595, 1019.6358, 1033.6520, 1047.4978, 1047.4978, 1051.6609, 1131.6592, 1133.5562, 1149.5057, 1149.5057, 1151.6812, 1156.5675, 1165.6880, 1165.6880, 1174.5699, 1174.5700, 1182.6278, 1182.6278, 1190.5620, 1194.5756, 1212.5294, 1277.6251, 1300.7948, 1311.6462, 1311.6462, 1334.8268, 1439.7417, 1448.8599, 1475.6879, 1528.7235, 1617.9976, 1695.6990, 1732.0278, 1839.8657, 1880.9835, 1901.1722, 1904.9435, 2015.2070, 2211.0264, 2283.1013, 2683.2788, 2700.2822, 2700.2822

27. A0A3Q1MXR2 **Mass:** 108457 **Score:** 40 **Expect:** 13 **Matches:** 17
Nebulette OS=Bos taurus OX=9913 GN=NEBL PE=4 SV=1

Observed	Mr(expt)	Mr(calc)	ppm	Start	End	Miss	Ions	Peptide
864.5128	863.5055	863.5116	-7.07	329	335	0	---	K.YITNLLK.E
960.4399	959.4326	959.4746	-43.74	792	799	0	---	R.SLHMLSEK.A + Oxidation (M)
996.5483	995.5410	995.4923	48.9	825	832	0	---	K.SEISEIYR.S
1017.6229	1016.6156	1016.5251	89.1	544	552	0	---	K.NQQNISSVK.Y
1017.6229	1016.6156	1016.5251	89.1	544	552	0	---	K.NQQNISSVK.Y
1045.5190	1044.5117	1044.5451	-31.96	453	461	1	---	K.DLENEIKGK.G
1069.4803	1068.4730	1068.5134	-37.83	876	884	0	---	R.SMQHSPNLR.T
1179.5543	1178.5470	1178.4575	76.0	18	27	0	---	K.TGEEENEEDK.V
1269.6084	1268.6011	1268.6612	-47.36	621	632	0	---	K.QATAISDPPELK.R
1287.6068	1286.5995	1286.5812	14.2	441	451	1	---	K.ASEMASQKEYK.K + Oxidation (M)
1287.6068	1286.5995	1286.5812	14.2	441	451	1	---	K.ASEMASQKEYK.K + Oxidation (M)
1305.5947	1304.5874	1304.6296	-32.30	750	760	0	---	K.GVHPHIVEMDR.R + Oxidation (M)
1425.6982	1424.6909	1424.7623	-50.11	621	633	1	---	K.QATAISDPPELKR.V
1664.7775	1663.7702	1663.8199	-29.88	423	437	1	---	R.GKGMQVSTDTLDIQR.A + Oxidation (M)
1691.7629	1690.7556	1690.8204	-38.34	462	476	1	---	K.GMQVSMDIPDMLRAK.R
1702.7892	1701.7819	1701.8719	-52.89	351	365	1	---	K.GMELNSEVLDIQRAK.R
2682.3079	2681.3006	2681.3231	-8.40	200	224	1	---	K.GQGVMNKEPAVIGRPDFEHAVEASK.L + Oxidation (M)

No match to: 834.4748, 842.4698, 848.4878, 850.0129, 850.4987, 853.4788, 862.4257, 865.9851, 868.5118, 870.5065, 876.9959, 882.5245, 882.5245, 892.9658, 951.5779, 977.4595, 977.4595, 1019.6358, 1033.6520, 1047.4978, 1047.4978, 1051.6609, 1131.6592, 1133.5562, 1149.5057, 1149.5057, 1151.6812, 1156.5675, 1165.6880, 1165.6880, 1174.5699, 1174.5700, 1182.6278, 1182.6278, 1190.5620, 1194.5756, 1212.5294, 1277.6251, 1300.7948, 1311.6462, 1311.6462, 1334.8268, 1439.7417, 1448.8599, 1475.6879, 1528.7235, 1617.9976, 1695.6990, 1732.0278, 1839.8657, 1880.9835, 1901.1722, 1904.9435, 2015.2070, 2211.0264, 2283.1013, 2683.2788, 2700.2822, 2700.2822

28. A0A4W2GHX5 **Mass:** 112926 **Score:** 40 **Expect:** 14 **Matches:** 17
Nebulette OS=Bos indicus x Bos taurus OX=30522 GN=NEBL PE=4 SV=1

Observed	Mr(expt)	Mr(calc)	ppm	Start	End	Miss	Ions	Peptide
864.5128	863.5055	863.5116	-7.07	367	373	0	---	K.YITNLLK.E
960.4399	959.4326	959.4746	-43.74	830	837	0	---	R.SLHMLSEK.A + Oxidation (M)
996.5483	995.5410	995.4923	48.9	863	870	0	---	K.SEISEIYR.S
1017.6229	1016.6156	1016.5251	89.1	582	590	0	---	K.NQQNISSVK.Y
1017.6229	1016.6156	1016.5251	89.1	582	590	0	---	K.NQQNISSVK.Y
1045.5190	1044.5117	1044.5451	-31.96	491	499	1	---	K.DLENEIKGK.G
1069.4803	1068.4730	1068.5134	-37.83	914	922	0	---	R.SMQHSPNLR.T
1179.5543	1178.5470	1178.4575	76.0	18	27	0	---	K.TGEEENEEDK.V
1269.6084	1268.6011	1268.6612	-47.36	659	670	0	---	K.QATAISDPPELK.R
1287.6068	1286.5995	1286.5812	14.2	479	489	1	---	K.ASEMASQKEYK.K + Oxidation (M)

PDF-XChange Click to buy NOW! www.docu-track.com

PDF-XChange Click to buy NOW! www.docu-track.com

1287.6068	1286.5995	1286.5812	14.2	479 - 489	1	---	K.ASEMASQKEYK.K + Oxidation (M)
1305.5947	1304.5874	1304.6296	-32.30	788 - 798	0	---	K.GVHPHIVEMDR.R + Oxidation (M)
1425.6982	1424.6909	1424.7623	-50.11	659 - 671	1	---	K.QATAISDPPELKR.V
1664.7775	1663.7702	1663.8199	-29.88	461 - 475	1	---	R.GKGMQVSTDTLDIQR.A + Oxidation (M)
1691.7629	1690.7556	1690.8204	-38.34	500 - 514	1	---	K.GMQVSMDIPDMLRAK.R
1702.7892	1701.7819	1701.8719	-52.89	389 - 403	1	---	K.GMELNSEVLDIQRAK.R
2682.3079	2681.3006	2681.3231	-8.40	200 - 224	1	---	K.GQGVMNKEPAVIGRPDFEHAVEASK.L + Oxidation (M)

No match to: 834.4748, 842.4698, 848.4878, 850.0129, 850.4987, 853.4788, 862.4257, 865.9851, 868.5118, 870.5065, 876.9959, 882.5245, 882.5245, 892.9658, 951.5779, 977.4595, 977.4595, 1019.6358, 1033.6520, 1047.4978, 1047.4978, 1051.6609, 1131.6592, 1133.5562, 1149.5057, 1149.5057, 1151.6812, 1156.5675, 1165.6880, 1165.6880, 1174.5699, 1174.5700, 1182.6278, 1182.6278, 1190.5620, 1194.5756, 1212.5294, 1277.6251, 1300.7948, 1311.6462, 1311.6462, 1334.8268, 1439.7417, 1448.8599, 1475.6879, 1528.7235, 1617.9976, 1695.6990, 1732.0278, 1839.8657, 1880.9835, 1901.1722, 1904.9435, 2015.2070, 2211.0264, 2283.1013, 2683.2788, 2700.2822, 2700.2822

29. A0A4W2CVR3 **Mass:** 479034 **Score:** 40 **Expect:** 14 **Matches:** 29

Dynein axonemal heavy chain 6 OS=Bos indicus x Bos taurus OX=30522 GN=DNAH6 PE=4 SV=1

Observed	Mr(expt)	Mr(calc)	ppm	Start - End	Miss	Ions	Peptide
834.4748	833.4675	833.4647	3.40	1784 - 1790	0	---	K.TYVLNPK.S
848.4878	847.4805	847.4334	55.6	2431 - 2436	1	---	R.MIRQER.G + Oxidation (M)
864.5128	863.5055	863.4753	35.0	925 - 931	0	---	K.FVTQLEK.G
868.5118	867.5045	867.4464	67.1	649 - 654	1	---	K.YHRQHK.D
960.4399	959.4326	959.4672	-36.05	2545 - 2553	0	---	K.EVGISEGNR.D
977.4595	976.4522	976.5012	-50.11	1810 - 1818	0	---	K.DGLMALSVR.A + Oxidation (M)
977.4595	976.4523	976.5012	-50.08	1810 - 1818	0	---	K.DGLMALSVR.A + Oxidation (M)
1045.5190	1044.5117	1044.5451	-31.97	4024 - 4033	0	---	R.DQATVIEAAK.T
1131.6592	1130.6519	1130.5608	80.6	232 - 240	1	---	K.NDYYTISKK.A
1149.5057	1148.4984	1148.4855	11.2	2320 - 2327	0	---	R.LFCHECQR.V
1149.5057	1148.4985	1148.4855	11.3	2320 - 2327	0	---	R.LFCHECQR.V
1156.5675	1155.5602	1155.5746	-12.48	1551 - 1559	1	---	R.FMFEGREIK.L
1182.6278	1181.6205	1181.5750	38.5	1280 - 1289	0	---	K.VEEAMFTSLR.R
1182.6278	1181.6205	1181.5750	38.5	1280 - 1289	0	5	K.VEEAMFTSLR.R
1287.6068	1286.5995	1286.7207	-94.20	1205 - 1215	1	---	R.NPQAVQPHLRK.C
1287.6068	1286.5995	1286.7207	-94.19	1205 - 1215	1	---	R.NPQAVQPHLRK.C
1425.6982	1424.6909	1424.7088	-12.57	3381 - 3391	0	---	R.LTEAEWNFFLR.G
1439.7417	1438.7344	1438.6472	60.6	1889 - 1900	0	---	R.CGMVFVDPEELK.W + Oxidation (M)
1448.8599	1447.8526	1447.9238	-49.18	1355 - 1368	1	---	R.LNALAAIVRGILPK.L
1475.6879	1474.6806	1474.7701	-60.67	2903 - 2916	0	---	R.AAQAELDITMATLK.E
1528.7235	1527.7162	1527.8046	-57.81	1030 - 1042	1	---	K.TTEFVVLPHRDSK.D
1617.9976	1616.9903	1616.8596	80.9	1119 - 1132	1	---	R.QLPAEAKMFLQVDK.S
1664.7775	1663.7702	1663.9185	-89.12	3501 - 3515	0	---	K.VVFALTDFVIENLGK.A
1691.7629	1690.7556	1690.8414	-50.71	907 - 921	0	---	K.FDSLDPELLNSQVSK.Y
1880.9835	1879.9762	1879.9138	33.2	4139 - 4154	1	---	K.DYWIAKGSALLCQLNE.-
1901.1722	1900.1649	1900.0319	70.0	2415 - 2430	1	---	K.LVFFQDAIEHVSRIAR.M
2211.0264	2210.0191	2210.0677	-22.00	1271 - 1289	1	---	R.GNVEEWLGKVEEAMFTSLR.R + Oxidation (M)
2682.3079	2681.3006	2681.3871	-32.27	3192 - 3214	1	---	K.SGLEDQLLSDVVRLEKPELEEQR.I
2683.2788	2682.2715	2682.3725	-37.66	3046 - 3069	1	---	R.QWNTDGLPRDLISTENGILVTQGR.R

No match to: 842.4698, 850.0129, 850.4987, 853.4788, 862.4257, 865.9851, 870.5065, 876.9959, 882.5245, 882.5245, 892.9658, 951.5779, 996.5483, 1017.6229, 1017.6229, 1019.6358, 1033.6520, 1047.4978, 1047.4978, 1051.6609, 1069.4803, 1133.5562, 1151.6812, 1165.6880, 1165.6880, 1174.5699, 1174.5700, 1179.5543, 1190.5620, 1194.5756, 1212.5294, 1269.6084, 1277.6251, 1300.7948, 1305.5947, 1311.6462, 1311.6462, 1334.8268, 1695.6990, 1702.7892, 1732.0278, 1839.8657, 1904.9435, 2015.2070, 2283.1013, 2700.2822, 2700.2822

30. A0A4W2I420 **Mass:** 167724 **Score:** 40 **Expect:** 14 **Matches:** 21

Lysine demethylase 2B OS=Bos indicus x Bos taurus OX=30522 GN=KDM2B PE=4 SV=1

Observed	Mr(expt)	Mr(calc)	ppm	Start - End	Miss	Ions	Peptide
842.4698	841.4625	841.4698	-8.61	18 - 24	0	---	R.LPVEWAK.L
868.5118	867.5045	867.4199	97.6	896 - 902	1	---	R.RSDEHPK.K
977.4595	976.4522	976.3920	61.7	887 - 894	0	---	R.SECEEAPR.R
977.4595	976.4523	976.3920	61.7	887 - 894	0	9	R.SECEEAPR.R
1047.4978	1046.4905	1046.5066	-15.38	471 - 479	0	---	R.ESMLIDAPR.K + Oxidation (M)
1047.4978	1046.4905	1046.5066	-15.38	471 - 479	0	0	R.ESMLIDAPR.K + Oxidation (M)
1069.4803	1068.4730	1068.4878	-13.85	730 - 737	1	---	R.MKQSCIMR.Q + Oxidation (M)
1133.5562	1132.5489	1132.4931	49.3	887 - 895	1	---	R.SECEEAPRR.R
1156.5675	1155.5602	1155.5196	35.1	243 - 251	0	---	R.YYETPEAQR.D
1165.6880	1164.6807	1164.6437	31.7	109 - 118	0	---	R.RPPVPPIMSR.A + Oxidation (M)
1165.6880	1164.6807	1164.6437	31.8	109 - 118	0	---	R.RPPVPPIMSR.A + Oxidation (M)
1194.5756	1193.5683	1193.6040	-29.91	425 - 433	1	---	R.IYEIEDRTR.V
1277.6251	1276.6178	1276.6333	-12.14	221 - 231	0	---	R.LVDVMDVNTQK.G + Oxidation (M)
1287.6068	1286.5995	1286.6871	-68.03	1296 - 1306	0	---	R.TLDVQWVEGLK.D
1287.6068	1286.5995	1286.6871	-68.02	1296 - 1306	0	---	R.TLDVQWVEGLK.D
1300.7948	1299.7875	1299.7584	22.4	1231 - 1242	0	---	K.SITPLMLSGIIR.R
1311.6462	1310.6389	1310.7129	-56.44	83 - 95	1	---	K.VAPVPKVGCTGAR.G
1311.6462	1310.6390	1310.7129	-56.41	83 - 95	1	---	K.VAPVPKVGCTGAR.G
1334.8268	1333.8195	1333.6990	90.4	925 - 935	1	---	K.YEKPQELSGRK.R
1702.7892	1701.7819	1701.8111	-17.11	175 - 187	1	---	K.DFNYEYVQREALR.V
1904.9435	1903.9362	1903.9462	-5.24	1296 - 1311	1	---	R.TLDVQWVEGLKDAQMR.D + Oxidation (M)

No match to: 834.4748, 848.4878, 850.0129, 850.4987, 853.4788, 862.4257, 864.5128, 865.9851, 870.5065, 876.9959, 882.5245, 882.5245, 892.9658, 951.5779, 960.4399, 996.5483, 1017.6229, 1017.6229, 1019.6358, 1033.6520, 1045.5190, 1051.6609, 1131.6592, 1149.5057, 1149.5057, 1151.6812, 1174.5699, 1174.5700, 1179.5543, 1182.6278, 1182.6278, 1190.5620, 1212.5294, 1269.6084, 1305.5947, 1425.6982, 1439.7417, 1448.8599, 1475.6879, 1528.7235, 1617.9976, 1664.7775, 1691.7629, 1695.6990, 1732.0278, 1839.8657, 1880.9835, 1901.1722, 2015.2070, 2211.0264, 2283.1013, 2682.3079, 2683.2788, 2700.2822, 2700.2822

Search Parameters

Type of search : Sequence Query
Enzyme : Trypsin
Fixed modifications : Carbamidomethyl (C)
Variable modifications : Oxidation (M)
Mass values : Monoisotopic
Protein Mass : Unrestricted
Peptide Mass Tolerance : ± 100 ppm
Fragment Mass Tolerance: ± 0.3 Da
Max Missed Cleavages : 1
Instrument type : MALDI-TOF-TOF

PDF-XChange Click to buy NOW! www.docu-track.com
PDF-XChange Click to buy NOW! www.docu-track.com

Query1 (834.4748,1+): <no title>
Query2 (842.4698,1+): <no title>
Query3 (848.4878,1+): <no title>
Query4 (850.0129,1+): <no title>
Query5 (850.4987,1+): <no title>
Query6 (853.4788,1+): <no title>
Query7 (862.4257,1+): <no title>
Query8 (864.5128,1+): <no title>
Query9 (865.9851,1+): <no title>
Query10 (868.5118,1+): <no title>
Query11 (870.5065,1+): <no title>
Query12 (876.9959,1+): <no title>
Query13 (882.5245,1+): <no title>
Query14 (882.5245,1+): Locus:1..14.0.7
Query15 (892.9658,1+): <no title>
Query16 (951.5779,1+): <no title>
Query17 (960.4399,1+): <no title>
Query18 (977.4595,1+): <no title>
Query19 (977.4595,1+): Locus:1..14.0.5
Query20 (996.5483,1+): <no title>
Query21 (1017.6229,1+): <no title>
Query22 (1017.6229,1+): Locus:1..14.0.11
Query23 (1019.6358,1+): <no title>
Query24 (1033.6520,1+): <no title>
Query25 (1045.5190,1+): <no title>
Query26 (1047.4978,1+): <no title>
Query27 (1047.4978,1+): Locus:1..14.0.1
Query28 (1051.6609,1+): <no title>
Query29 (1069.4803,1+): <no title>
Query30 (1131.6592,1+): <no title>
Query31 (1133.5562,1+): <no title>
Query32 (1149.5057,1+): <no title>
Query33 (1149.5057,1+): Locus:1..14.0.9
Query34 (1151.6812,1+): <no title>
Query35 (1156.5675,1+): <no title>
Query36 (1165.6880,1+): Locus:1..14.0.10
Query37 (1165.6880,1+): <no title>
Query38 (1174.5699,1+): <no title>
Query39 (1174.5700,1+): Locus:1..14.0.2
Query40 (1179.5543,1+): <no title>
Query41 (1182.6278,1+): <no title>
Query42 (1182.6278,1+): Locus:1..14.0.8
Query43 (1190.5620,1+): <no title>
Query44 (1194.5756,1+): <no title>
Query45 (1212.5294,1+): <no title>
Query46 (1269.6084,1+): <no title>
Query47 (1277.6251,1+): <no title>
Query48 (1287.6068,1+): <no title>
Query49 (1287.6068,1+): Locus:1..14.0.4
Query50 (1300.7948,1+): <no title>
Query51 (1305.5947,1+): <no title>
Query52 (1311.6462,1+): <no title>
Query53 (1311.6462,1+): Locus:1..14.0.3
Query54 (1334.8268,1+): <no title>
Query55 (1425.6982,1+): <no title>
Query56 (1439.7417,1+): <no title>
Query57 (1448.8599,1+): <no title>
Query58 (1475.6879,1+): <no title>
Query59 (1528.7235,1+): <no title>
Query60 (1617.9976,1+): <no title>
Query61 (1664.7775,1+): <no title>
Query62 (1691.7629,1+): <no title>
Query63 (1695.6990,1+): <no title>
Query64 (1702.7892,1+): <no title>
Query65 (1732.0278,1+): <no title>
Query66 (1839.8657,1+): <no title>
Query67 (1880.9835,1+): <no title>
Query68 (1901.1722,1+): <no title>
Query69 (1904.9435,1+): <no title>
Query70 (2015.2070,1+): <no title>
Query71 (2211.0264,1+): <no title>
Query72 (2283.1013,1+): <no title>
Query73 (2682.3079,1+): <no title>
Query74 (2683.2788,1+): <no title>
Query75 (2700.2822,1+): <no title>
Query76 (2700.2822,1+): Locus:1..14.0.6

Mascot: http://www.matrixscience.com/
