# Supplementary material for: Alpha-2-Macroglobulin as a New Promising Biomarker Improving the Diagnostic Sensitivity of Bovine Paratuberculosis
Source: Front Vet Sci. 2021 Mar 5;8:637716. doi: 10.3389/fvets.2021.637716 (PMC7973028; doi:10.3389/fvets.2021.637716)
Supplement: Supplementary Data 1 — Mascot search results and raw data files for LC-MS/MS analysis for the identification of specific blood proteins in cattle with Johne's disease. [file Data_Sheet_1.zip › mascot/3 Apolipoprotein A-IV_2.pdf]

PDF-XChange Click to buy NOW! www.docu-track.com

PDF-XChange Click to buy NOW! www.docu-track.com

MATRIX SCIENCE

MASCOT Search Results

Protein View: V6F7X3

Apolipoprotein A-IV OS=Bos taurus OX=9913 GN=APOA4 PE=3 SV=1

Database:	Uniprot_bovine
Score:	283
Expect:	6.7e-024
Nominal mass (M_r):	42963
Calculated pI:	5.30

Sequence similarity is available as an NCBI BLAST search of V6F7X3 against nr.

Search parameters

Enzyme:	Trypsin: cuts C-term side of KR unless next residue is P.
Fixed modifications:	Carbamidomethyl (C)
Variable modifications:	Oxidation (M)

Protein sequence coverage: 41%

Matched peptides shown in ***bold red***.

```
  1 MFLKAVVLSL ALVAVTGAEA EVNADQVATV IWDYFSQLGN NAKKAVEHIQ
 51 KSELTQQLNT LFQDKLGEVS TYTDDLQKKL VPFATELHER LTKDSEKLKE
101 EIRKELEDLR ARLLPHATEV SQKIGDNVRE LQQRLGPYAE ELRTQVDTQA
151 QQLRRQLTPY AERMEKVMRQ NLDQLQASLA PYAEELQATV NQRVEELKGR
201 LTPYADQLQT KIEENVEELR RSLAPYAQDV QGKLNHQLEG LAFQMKKHAE
251 ELKAKISAKA EELRQGLVPL VNSVHGSQLG NAEDLQKSLA ELSSRLDQQV
301 EDFRRTVGPY GETFNKAMVQ QLDTLRQKLG PLAGDVEDHL SFLEKDLRDK
351 VSSFFNTLKE KESQAPALPA QEEMPVPLGG
```

Unformatted sequence string: 380 residues (for pasting into other applications).

Sort peptides by ◉ Residue Number ○ Increasing Mass ○ Decreasing Mass

Show predicted peptides also

Query	Start – End	Observed	Mr(expt)	Mr(calc)	ppm	M	Score	Peptide
61	52 – 65	1664.7775	1663.7702	1663.8417	-43.0	0		K.SELTQQLNTLFQDK.L
56	79 – 90	1439.7417	1438.7344	1438.7932	-40.9	1		K.KLVPFATELHER.L
52	80 – 90	1311.6462	1310.6389	1310.6983	-45.3	0		K.LVPFATELHER.L
53	80 – 90	1311.6462	1310.6390	1310.6983	-45.2	0	14	K.LVPFATELHER.L
26	135 – 143	1047.4978	1046.4905	1046.5396	-46.9	0		R.LGPYAEELR.T
27	135 – 143	1047.4978	1046.4905	1046.5396	-46.9	0	20	R.LGPYAEELR.T
48	144 – 154	1287.6068	1286.5995	1286.6579	-45.4	0		R.TQVDTQAQQLR.R
49	144 – 154	1287.6068	1286.5995	1286.6579	-45.3	0	14	R.TQVDTQAQQLR.R
31	155 – 163	1133.5562	1132.5489	1132.5989	-44.1	1		R.RQLTPYAER.M
18	156 – 163	977.4595	976.4522	976.4978	-46.6	0		R.QLTPYAER.M
19	156 – 163	977.4595	976.4523	976.4978	-46.6	0	15	R.QLTPYAER.M
75	170 – 193	2700.2822	2699.2749	2699.3514	-28.3	0		R.QNLDQLQASLAPYAEELQATVNQR.V
76	170 – 193	2700.2822	2699.2750	2699.3514	-28.3	0	36	R.QNLDQLQASLAPYAEELQATVNQR.V
47	201 – 211	1277.6251	1276.6178	1276.6663	-38.0	0		R.LTPYADQLQTK.I
59	234 – 246	1528.7235	1527.7162	1527.7868	-46.2	0		K.LNHQLEGLAFQMK.K
7	288 – 295	862.4257	861.4184	861.4556	-43.1	0		K.SLAELSSR.L
32	296 – 304	1149.5057	1148.4984	1148.5462	-41.6	0		R.LDQQVEDFR.R
33	296 – 304	1149.5057	1148.4985	1148.5462	-41.6	0	20	R.LDQQVEDFR.R
51	296 – 305	1305.5947	1304.5874	1304.6473	-45.9	1		R.LDQQVEDFRR.T
45	306 – 316	1212.5294	1211.5221	1211.5823	-49.6	0		R.TVGPYGETFNK.A
38	317 – 326	1174.5699	1173.5626	1173.6176	-46.8	0		K.AMVQQLDTLR.Q
39	317 – 326	1174.5700	1173.5627	1173.6176	-46.8	0	50	K.AMVQQLDTLR.Q
43	317 – 326	1190.5620	1189.5547	1189.6125	-48.6	0		K.AMVQQLDTLR.Q + Oxidation (M)
66	329 – 345	1839.8657	1838.8584	1838.9414	-45.1	0		K.LGPLAGDVEDHLSFLEK.D

PDF-XChange
Click to buy NOW!
www.docu-track.com

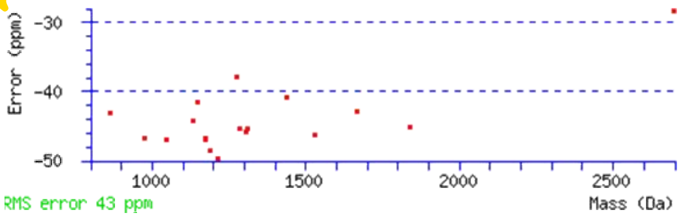


```
ID   V6F7X3_BOVIN            Unreviewed;       380 AA.
AC   V6F7X3;
DT   10-APR-2019, integrated into UniProtKB/TrEMBL.
DT   10-APR-2019, sequence version 1.
DT   26-FEB-2020, entry version 9.
DE   SubName: Full=Apolipoprotein A-IV {ECO:0000313|EMBL:DAA22387.1};
GN   Name=APOA4 {ECO:0000313|EMBL:DAA22387.1};
GN   ORFNames=BOS_14370 {ECO:0000313|EMBL:DAA22387.1};
OS   Bos taurus (Bovine).
OC   Eukaryota; Metazoa; Chordata; Craniata; Vertebrata; Euteleostomi; Mammalia;
OC   Eutheria; Laurasiatheria; Artiodactyla; Ruminantia; Pecora; Bovidae;
OC   Bovinae; Bos.
OX   NCBI_TaxID=9913 {ECO:0000313|EMBL:DAA22387.1, ECO:0000313|Proteomes:UP000321980};
RN   [1] {ECO:0000313|EMBL:DAA22387.1, ECO:0000313|Proteomes:UP000321980}
RP   NUCLEOTIDE SEQUENCE [LARGE SCALE GENOMIC DNA].
RX   PubMed=19393038; DOI=10.1186/gb-2009-10-4-r42;
RA   Zimin A.V., Delcher A.L., Florea L., Kelley D.R., Schatz M.C., Puiu D.,
RA   Hanrahan F., Pertea G., Van Tassell C.P., Sonstegard T.S., Marcais G.,
RA   Roberts M., Subramanian P., Yorke J.A., Salzberg S.L.;
RT   "A whole-genome assembly of the domestic cow, Bos taurus.";
RL   Genome Biol. 10:R42.01-R42.10(2009).
CC   -!- SUBCELLULAR LOCATION: Secreted {ECO:0000256|SAAS:SAAS00143437}.
CC   -!- SIMILARITY: Belongs to the apolipoprotein A1/A4/E family.
CC       {ECO:0000256|SAAS:SAAS00598075}.
CC   ---------------------------------------------------------------------------
CC   Copyrighted by the UniProt Consortium, see https://www.uniprot.org/terms
CC   Distributed under the Creative Commons Attribution (CC BY 4.0) License
CC   ---------------------------------------------------------------------------
DR   EMBL; GJ062397; DAA22387.1; -; Genomic_DNA.
DR   RefSeq; XP_005215912.1; XM_005215855.3.
DR   RefSeq; XP_010810673.1; XM_010812371.2.
DR   RefSeq; XP_015330105.1; XM_015474619.1.
DR   SMR; V6F7X3; -.
DR   GeneID; 537301; -.
DR   CTD; 337; -.
DR   OMA; TVVWDYF; -.
DR   OrthoDB; 1299087at2759; -.
DR   Proteomes; UP000321980; Chromosome 15.
DR   GO; GO:0005576; C:extracellular region; IEA:UniProtKB-SubCell.
DR   GO; GO:0008289; F:lipid binding; IEA:InterPro.
DR   GO; GO:0006869; P:lipid transport; IEA:UniProtKB-KW.
DR   GO; GO:0042157; P:lipoprotein metabolic process; IEA:InterPro.
DR   InterPro; IPR000074; ApoA_E.
DR   Pfam; PF01442; Apolipoprotein; 2.
PE   3: Inferred from homology;
KW   Chylomicron {ECO:0000256|SAAS:SAAS01245600};
KW   Coiled coil {ECO:0000256|SAM:Coils};
KW   Lipid transport {ECO:0000256|SAAS:SAAS00143435};
KW   Lipoprotein {ECO:0000313|EMBL:DAA22387.1};
KW   Secreted {ECO:0000256|SAAS:SAAS00143438}; Signal {ECO:0000256|SAM:SignalP};
KW   Transport {ECO:0000256|SAAS:SAAS00143435}.
FT   SIGNAL          1..20
FT                   /evidence="ECO:0000256|SAM:SignalP"
FT   CHAIN           21..380
FT                   /evidence="ECO:0000256|SAM:SignalP"
FT                   /id="PRO_5018663070"
FT   REGION          361..380
FT                   /note="Disordered"
FT                   /evidence="ECO:0000256|SAM:MobiDB-lite"
FT   COILED          88..112
FT                   /evidence="ECO:0000256|SAM:Coils"
FT   COILED          246..266
FT                   /evidence="ECO:0000256|SAM:Coils"
FT   COILED          282..302
FT                   /evidence="ECO:0000256|SAM:Coils"
SQ   SEQUENCE   380 AA;  42990 MW;  9D3F49D2A70BE77C CRC64;
     MFLKAVVLSL ALVAVTGAEA EVNADQVATV IWDYFSQLGN NAKKAVEHIQ KSELTQQLNT
     LFQDKLGEVS TYTDDLQKKL VPFATELHER LTKDSEKLKE EIRKELEDLR ARLLPHATEV
     SQKIGDNVRE LQQRLGPYAE ELRTQVDTQA QQLRRQLTPY AERMEKVMRQ NLDQLQASLA
     PYAEELQATV NQRVEELKGR LTPYADQLQT KIEENVEELR RSLAPYAQDV QGKLNHQLEG
     LAFQMKKHAE ELKAKISAKA EELRQGLVPL VNSVHGSQLG NAEDLQKSLA ELSSRLDQQV
```

```
    EDFRRTVGPY GETFNKAMVQ QLDTLRQKLG PLAGDVEDHL SFLEKDLRDK VSSFFNTLKE
    KESQAPALPA QEEMPVPLGG
//
```

Mascot: http://www.matrixscience.com/
