# Supplementary material for: Alpha-2-Macroglobulin as a New Promising Biomarker Improving the Diagnostic Sensitivity of Bovine Paratuberculosis
Source: Front Vet Sci. 2021 Mar 5;8:637716. doi: 10.3389/fvets.2021.637716 (PMC7973028; doi:10.3389/fvets.2021.637716)
Supplement: Supplementary Data 1 — Mascot search results and raw data files for LC-MS/MS analysis for the identification of specific blood proteins in cattle with Johne's disease. [file Data_Sheet_1.zip › mascot/4 Alpha-2-macroglobulin_1.pdf]

PDF-XChange Click to buy NOW! www.docu-track.com

MATRIX SCIENCE Mascot Search Results

User	:
Email	:
Search title	: BAEDW\2020_3_5\2020_3_5\MSMS 14\N11
Database	: Uniprot_bovine bovine_20200316 (134392 sequences; 76244912 residues)
Timestamp	: 31 Mar 2020 at 01:08:40 GMT
Warning	: A Peptide summary report will usually give a much clearer picture of MS/MS search results.
Top Score	: 90 for R9QSM8, Alpha-2-macroglobulin OS=Bos taurus OX=9913 GN=A2M PE=2 SV=1

Mascot Score Histogram

Protein score is -10*Log(P), where P is the probability that the observed match is a random event.
Protein scores greater than 64 are significant (p<0.05).
Protein scores are derived from ions scores as a non-probabilistic basis for ranking protein hits.

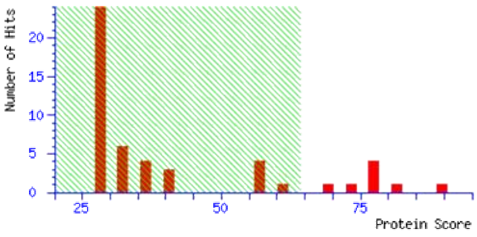

Protein Summary Report

Format As | Protein Summary (deprecated) | Help

Significance threshold p< 0.05 Max. number of hits 30

Preferred taxonomy All entries

Re-Search All | Search Unmatched

Index

	Accession	Mass	Score	Description
1.	R9QSM8	134613	90	Alpha-2-macroglobulin OS=Bos taurus OX=9913 GN=A2M PE=2 SV=1
2.	K4JF16	102208	83	Alpha-2-macroglobulin variant 23 OS=Bos taurus OX=9913 GN=A2M PE=2 SV=1
3.	K4JR88	78030	78	Alpha-2-macroglobulin variant 22 OS=Bos taurus OX=9913 GN=A2M PE=2 SV=1
4.	A0A4W2E185	169075	78	Alpha-2-macroglobulin OS=Bos indicus x Bos taurus OX=30522 GN=A2M PE=4 SV=1
5.	Q7SIH1	168953	78	Alpha-2-macroglobulin OS=Bos taurus OX=9913 GN=A2M PE=1 SV=2
6.	A0A4W2CTC9	169552	78	Alpha-2-macroglobulin OS=Bos indicus x Bos taurus OX=30522 GN=A2M PE=4 SV=1
7.	K4JBR5	116087	75	Alpha-2-macroglobulin variant 1 OS=Bos taurus OX=9913 GN=A2M PE=2 SV=1
8.	K4JDT2	99307	68	Alpha-2-macroglobulin variant 20 OS=Bos taurus OX=9913 GN=A2M PE=2 SV=1
9.	A0A4W2FQH1	164938	59	Alpha-2-macroglobulin OS=Bos indicus x Bos taurus OX=30522 GN=A2M PE=4 SV=1
10.	A0A4W2FQJ3	166227	59	Alpha-2-macroglobulin OS=Bos indicus x Bos taurus OX=30522 GN=A2M PE=4 SV=1
11.	A0A4W2FQM6	168925	58	Alpha-2-macroglobulin OS=Bos indicus x Bos taurus OX=30522 GN=A2M PE=4 SV=1
12.	A0A4W2HMW5	168978	58	Alpha-2-macroglobulin OS=Bos indicus x Bos taurus OX=30522 GN=A2M PE=4 SV=1
13.	A0A4W2HM51	168906	58	Alpha-2-macroglobulin OS=Bos indicus x Bos taurus OX=30522 GN=A2M PE=4 SV=1
14.	A0A4W2BQ12	48807	41	Tripartite motif containing 77 OS=Bos indicus x Bos taurus OX=30522 GN=TRIM77 PE=4 SV=1
15.	A0A4W2FVS5	48837	41	Uncharacterized protein OS=Bos indicus x Bos taurus OX=30522 GN=LOC113885841 PE=4 SV=1
16.	A0A1L7BC46	55660	39	NS5A (Fragment) OS=Bovine viral diarrhea virus OX=11099 PE=4 SV=1
17.	A0A4W2FCR6	52833	36	Reverse transcriptase domain-containing protein OS=Bos indicus x Bos taurus OX=30522 PE=4 SV=1
18.	A0A4W2HM23	53406	35	Glucosaminyl (N-acetyl) transferase 4 OS=Bos indicus x Bos taurus OX=30522 GN=GCNT4 PE=4 SV=1
19.	A0A1L7BC66	56212	35	NS5A (Fragment) OS=Bovine viral diarrhea virus OX=11099 PE=4 SV=1
20.	A0A3Q1MD56	430029	34	Lysosomal-trafficking regulator OS=Bos taurus OX=9913 GN=LYST PE=4 SV=1
21.	A0A4W2E9U7	76824	34	Nibrin OS=Bos indicus x Bos taurus OX=30522 GN=NBN PE=4 SV=1
22.	E1BNC9	124764	32	Ankyrin 3 OS=Bos taurus OX=9913 GN=ANK3 PE=4 SV=3
23.	G8JKV3	11943	32	Glutaredoxin domain-containing protein OS=Bos taurus OX=9913 PE=4 SV=1
24.	A0A4W2C8T2	11943	32	Glutaredoxin domain-containing protein OS=Bos indicus x Bos taurus OX=30522 PE=4 SV=1
25.	A0A3Q1MUB2	54957	31	Glucosaminyl (N-acetyl) transferase 4 OS=Bos taurus OX=9913 GN=GCNT4 PE=4 SV=1
26.	A0A3Q1M1F6	143195	30	Myomesin 3 OS=Bos taurus OX=9913 GN=MYOM3 PE=4 SV=1
27.	Q9QAQ5	12971	30	Non-structural protein of 12.7 kDa OS=Bovine coronavirus (strain OK-0514) OX=231432 GN=5a PE=3 SV=1
28.	Q6YPB4	16612	30	NOSIC domain-containing protein (Fragment) OS=Bos taurus OX=9913 PE=2 SV=1
29.	D8V071	35943	30	Phosphoprotein OS=Tibrogargan virus (strain CS132) OX=1559361 GN=P PE=3 SV=1
30.	A0A4W2DGE8	25670	29	Peptidylprolyl isomerase OS=Bos indicus x Bos taurus OX=30522 GN=FKBP11 PE=4 SV=1

Results List

1. R9QSM8 **Mass:** 134613 **Score:** 90 **Expect:** 0.00014 **Matches:** 20
Alpha-2-macroglobulin OS=Bos taurus OX=9913 GN=A2M PE=2 SV=1

Observed	Mr(expt)	Mr(calc)	ppm	Start		End	Miss	Ions	Peptide
996.5488	995.5415	995.4607	81.2	1111	-	1118	1	---	K.QKSNGCFR.S
1131.6523	1130.6450	1130.6295	13.7	1119	-	1129	0	---	R.STGTLLNNAIK.G
1250.6191	1249.6118	1249.6667	-43.88	350	-	360	0	---	K.LSFVTVDSNLR.R
1250.6191	1249.6119	1249.6667	-43.85	350	-	360	0	---	K.LSFVTVDSNLR.R
1287.6302	1286.6229	1286.6830	-46.71	777	-	789	0	---	R.DTGLGLSPTASLR.V
1302.6307	1301.6234	1301.6762	-40.52	587	-	598	0	---	R.VTASPQSLCALR.A
1421.6768	1420.6695	1420.7350	-46.15	455	-	466	0	14	R.SNSFVYLEPLPR.E
1421.6768	1420.6695	1420.7350	-46.12	455	-	466	0	---	R.SNSFVYLEPLPR.E
1479.7271	1478.7198	1478.6422	52.5	667	-	678	0	---	K.DMYSFLQDMGLK.A + 2 Oxidation (M)
1491.6775	1490.6702	1490.7340	-42.81	82	-	93	0	---	K.DLFHCVSFTLPR.S
1523.7185	1522.7112	1522.7814	-46.06	983	-	997	0	---	R.ASFSVLGDILGSAMR.N
1707.8094	1706.8021	1706.8913	-52.25	94	-	108	0	---	R.SPTSQEVMFLTIQVK.G
1900.9595	1899.9522	1900.0306	-41.25	540	-	557	0	13	R.LLIYAILPDGEVVGDSAR.Y
1900.9595	1899.9522	1900.0306	-41.23	540	-	557	0	---	R.LLIYAILPDGEVVGDSAR.Y
1991.8297	1990.8224	1990.8909	-34.39	718	-	734	0	---	R.DFVHFDDTSEPPTETVR.K

PDF-XChange Click to buy NOW! www.docu-track.com
PDF-XChange Click to buy NOW! www.docu-track.com

2001.9189	2000.9116	2000.9844	-36.36	210 -	228	0	---	K.GSGGTAEHPFTVEEFVLPK.F
2012.9465	2011.9392	2012.0228	-41.54	568 -	586	0	---	K.VGLNFSPGQSFPASQAHLR.V
2012.9465	2011.9393	2012.0228	-41.53	568 -	586	0	---	K.VGLNFSPGQSFPASQAHLR.V
2073.0088	2072.0015	2072.0805	-38.12	790 -	806	0	---	R.VFQPFFVELTMPYSVIR.G
2927.5508	2926.5435	2926.5837	-13.74	599 -	625	0	---	R.AVDQSVLLMRPEAELSAATVYNLLPVK.D

No match to: 842.4690, 848.4879, 850.0154, 850.4973, 853.4743, 853.4743, 861.0195, 864.5118, 865.9869, 868.5078, 870.5034, 876.9944, 881.9677, 882.5228, 882.5228, 892.9686, 951.5814, 953.4899, 1017.6209, 1017.6209, 1019.6330, 1033.6514, 1045.5168, 1051.6565, 1051.6565, 1136.6420, 1147.6807, 1151.6740, 1165.6849, 1165.6849, 1300.7900, 1316.8218, 1334.8221, 1334.8221, 1356.6021, 1448.8531, 1454.7075, 1475.6875, 1518.6587, 1552.7145, 1559.7947, 1567.6738, 1617.9915, 1617.9915, 1732.0206, 2015.1936, 2044.9634, 2193.0256, 2211.0200, 2230.1453, 2233.0505, 2239.0674, 2283.0967, 2284.0908, 2305.0867, 3337.7156

2. K4JF16 **Mass:** 102208 **Score:** 83 **Expect:** 0.00071 **Matches:** 17

Alpha-2-macroglobulin variant 23 OS=Bos taurus OX=9913 GN=A2M PE=2 SV=1

Observed	Mr(expt)	Mr(calc)	ppm	Start	End	Miss	Ions	Peptide
1250.6191	1249.6118	1249.6667	-43.88	350 -	360	0	---	K.LSFVTVDSNLR.R
1250.6191	1249.6119	1249.6667	-43.85	350 -	360	0	---	K.LSFVTVDSNLR.R
1287.6302	1286.6229	1286.6830	-46.71	777 -	789	0	---	R.DTGLGLSPTASLR.V
1302.6307	1301.6234	1301.6762	-40.52	587 -	598	0	---	R.VTASPQSLCALR.A
1421.6768	1420.6695	1420.7350	-46.15	455 -	466	0	14	R.SNSFVYLEPLPR.E
1421.6768	1420.6695	1420.7350	-46.12	455 -	466	0	---	R.SNSFVYLEPLPR.E
1479.7271	1478.7198	1478.6422	52.5	667 -	678	0	---	K.DMYSFLQDMGLK.A + 2 Oxidation (M)
1491.6775	1490.6702	1490.7340	-42.81	82 -	93	0	---	K.DLFHCVSFTLPR.S
1707.8094	1706.8021	1706.8913	-52.25	94 -	108	0	---	R.SPTSQEVMFLTIQVK.G
1900.9595	1899.9522	1900.0306	-41.25	540 -	557	0	13	R.LLIYAILPDGEVVGDSAR.Y
1900.9595	1899.9522	1900.0306	-41.23	540 -	557	0	---	R.LLIYAILPDGEVVGDSAR.Y
1991.8297	1990.8224	1990.8909	-34.39	718 -	734	0	---	R.DFVHFDDTSEPPTETVR.K
2001.9189	2000.9116	2000.9844	-36.36	210 -	228	0	---	K.GSGGTAEHPFTVEEFVLPK.F
2012.9465	2011.9392	2012.0228	-41.54	568 -	586	0	---	K.VGLNFSPGQSFPASQAHLR.V
2012.9465	2011.9393	2012.0228	-41.53	568 -	586	0	---	K.VGLNFSPGQSFPASQAHLR.V
2073.0088	2072.0015	2072.0805	-38.12	790 -	806	0	---	R.VFQPFFVELTMPYSVIR.G
2927.5508	2926.5435	2926.5837	-13.74	599 -	625	0	---	R.AVDQSVLLMRPEAELSAATVYNLLPVK.D

No match to: 842.4690, 848.4879, 850.0154, 850.4973, 853.4743, 853.4743, 861.0195, 864.5118, 865.9869, 868.5078, 870.5034, 876.9944, 881.9677, 882.5228, 882.5228, 892.9686, 951.5814, 953.4899, 996.5488, 1017.6209, 1017.6209, 1019.6330, 1033.6514, 1045.5168, 1051.6565, 1051.6565, 1131.6523, 1136.6420, 1147.6807, 1151.6740, 1165.6849, 1165.6849, 1300.7900, 1316.8218, 1334.8221, 1334.8221, 1356.6021, 1448.8531, 1454.7075, 1475.6875, 1518.6587, 1523.7185, 1552.7145, 1559.7947, 1567.6738, 1617.9915, 1617.9915, 1732.0206, 2015.1936, 2044.9634, 2193.0256, 2211.0200, 2230.1453, 2233.0505, 2239.0674, 2283.0967, 2284.0908, 2305.0867, 3337.7156

3. K4JR88 **Mass:** 78030 **Score:** 78 **Expect:** 0.0021 **Matches:** 15

Alpha-2-macroglobulin variant 22 OS=Bos taurus OX=9913 GN=A2M PE=2 SV=1

Observed	Mr(expt)	Mr(calc)	ppm	Start	End	Miss	Ions	Peptide
1250.6191	1249.6118	1249.6667	-43.88	350 -	360	0	---	K.LSFVTVDSNLR.R
1250.6191	1249.6119	1249.6667	-43.85	350 -	360	0	---	K.LSFVTVDSNLR.R
1287.6302	1286.6229	1286.6983	-58.57	473 -	483	0	---	K.TQTVQAHYVLK.G
1302.6307	1301.6234	1301.6762	-40.52	587 -	598	0	---	R.VTASPQSLCALR.A
1421.6768	1420.6695	1420.7350	-46.15	455 -	466	0	14	R.SNSFVYLEPLPR.E
1421.6768	1420.6695	1420.7350	-46.12	455 -	466	0	---	R.SNSFVYLEPLPR.E
1479.7271	1478.7198	1478.6422	52.5	667 -	678	0	---	K.DMYSFLQDMGLK.A + 2 Oxidation (M)
1491.6775	1490.6702	1490.7340	-42.81	82 -	93	0	---	K.DLFHCVSFTLPR.S
1707.8094	1706.8021	1706.8913	-52.25	94 -	108	0	---	R.SPTSQEVMFLTIQVK.G
1900.9595	1899.9522	1900.0306	-41.25	540 -	557	0	13	R.LLIYAILPDGEVVGDSAR.Y
1900.9595	1899.9522	1900.0306	-41.23	540 -	557	0	---	R.LLIYAILPDGEVVGDSAR.Y
2001.9189	2000.9116	2000.9844	-36.36	210 -	228	0	---	K.GSGGTAEHPFTVEEFVLPK.F
2012.9465	2011.9392	2012.0228	-41.54	568 -	586	0	---	K.VGLNFSPGQSFPASQAHLR.V
2012.9465	2011.9393	2012.0228	-41.53	568 -	586	0	---	K.VGLNFSPGQSFPASQAHLR.V
2927.5508	2926.5435	2926.5837	-13.74	599 -	625	0	---	R.AVDQSVLLMRPEAELSAATVYNLLPVK.D

No match to: 842.4690, 848.4879, 850.0154, 850.4973, 853.4743, 853.4743, 861.0195, 864.5118, 865.9869, 868.5078, 870.5034, 876.9944, 881.9677, 882.5228, 882.5228, 892.9686, 951.5814, 953.4899, 996.5488, 1017.6209, 1017.6209, 1019.6330, 1033.6514, 1045.5168, 1051.6565, 1051.6565, 1131.6523, 1136.6420, 1147.6807, 1151.6740, 1165.6849, 1165.6849, 1300.7900, 1316.8218, 1334.8221, 1334.8221, 1356.6021, 1448.8531, 1454.7075, 1475.6875, 1518.6587, 1523.7185, 1552.7145, 1559.7947, 1567.6738, 1617.9915, 1617.9915, 1732.0206, 1991.8297, 2015.1936, 2044.9634, 2073.0088, 2193.0256, 2211.0200, 2230.1453, 2233.0505, 2239.0674, 2283.0967, 2284.0908, 2305.0867, 3337.7156

4. A0A4W2E185 **Mass:** 169075 **Score:** 78 **Expect:** 0.0022 **Matches:** 20

Alpha-2-macroglobulin OS=Bos indicus x Bos taurus OX=30522 GN=A2M PE=4 SV=1

Observed	Mr(expt)	Mr(calc)	ppm	Start	End	Miss	Ions	Peptide
996.5488	995.5415	995.4607	81.2	1111 -	1118	1	---	K.QKSNGCFR.S
1131.6523	1130.6450	1130.6295	13.7	1119 -	1129	0	---	R.STGTLLNNAIK.G
1250.6191	1249.6118	1249.6667	-43.88	350 -	360	0	---	K.LSFVTVDSNLR.R
1250.6191	1249.6119	1249.6667	-43.85	350 -	360	0	---	K.LSFVTVDSNLR.R
1287.6302	1286.6229	1286.6830	-46.71	777 -	789	0	---	R.DTGLGLSPTASLR.V
1302.6307	1301.6234	1301.6762	-40.52	587 -	598	0	---	R.VTASPQSLCALR.A
1421.6768	1420.6695	1420.7350	-46.15	455 -	466	0	14	R.SNSFVYLEPLPR.E
1421.6768	1420.6695	1420.7350	-46.12	455 -	466	0	---	R.SNSFVYLEPLPR.E
1479.7271	1478.7198	1478.6422	52.5	667 -	678	0	---	K.DMYSFLQDMGLK.A + 2 Oxidation (M)
1491.6775	1490.6702	1490.7340	-42.81	82 -	93	0	---	K.DLFHCVSFTLPR.S
1523.7185	1522.7112	1522.7814	-46.06	983 -	997	0	---	R.ASFSVLGDILGSAMR.N
1707.8094	1706.8021	1706.8913	-52.25	94 -	108	0	---	R.SPTSQEVMFLTIQVK.G
1900.9595	1899.9522	1900.0306	-41.25	540 -	557	0	13	R.LLIYAILPDGEVVGDSAR.Y
1900.9595	1899.9522	1900.0306	-41.23	540 -	557	0	---	R.LLIYAILPDGEVVGDSAR.Y
1991.8297	1990.8224	1990.8909	-34.39	718 -	734	0	---	R.DFVHFDDTSEPPTETVR.K
2001.9189	2000.9116	2000.9844	-36.36	210 -	228	0	---	K.GSGGTAEHPFTVEEFVLPK.F
2012.9465	2011.9392	2012.0228	-41.54	568 -	586	0	---	K.VGLNFSPGQSFPASQAHLR.V
2012.9465	2011.9393	2012.0228	-41.53	568 -	586	0	---	K.VGLNFSPGQSFPASQAHLR.V
2073.0088	2072.0015	2072.0805	-38.12	790 -	806	0	---	R.VFQPFFVELTMPYSVIR.G
2927.5508	2926.5435	2926.5837	-13.74	599 -	625	0	---	R.AVDQSVLLMRPEAELSAATVYNLLPVK.D

No match to: 842.4690, 848.4879, 850.0154, 850.4973, 853.4743, 853.4743, 861.0195, 864.5118, 865.9869, 868.5078, 870.5034, 876.9944, 881.9677, 882.5228, 882.5228, 892.9686, 951.5814, 953.4899, 1017.6209, 1017.6209, 1019.6330, 1033.6514, 1045.5168, 1051.6565, 1051.6565, 1136.6420, 1147.6807, 1151.6740, 1165.6849, 1165.6849, 1300.7900, 1316.8218, 1334.8221, 1334.8221, 1356.6021, 1448.8531, 1454.7075, 1475.6875, 1518.6587, 1552.7145, 1559.7947, 1567.6738, 1617.9915, 1617.9915, 1732.0206, 2015.1936, 2044.9634, 2193.0256, 2211.0200, 2230.1453, 2233.0505, 2239.0674, 2283.0967, 2284.0908, 2305.0867, 3337.7156

5. Q7SIH1 **Mass:** 168953 **Score:** 78 **Expect:** 0.0022 **Matches:** 20

PDF-XChange Click to buy NOW! www.docu-track.com

Alpha-2-macroglobulin OS=Bos taurus OX=9913 GN=A2M PE=1 SV=2

Observed	Mr(expt)	Mr(calc)	ppm	Start	End	Miss	Ions	Peptide
996.5488	995.5415	995.4607	81.2	1111	1118	1	---	K.QKSNGCFR.S
1131.6523	1130.6450	1130.6295	13.7	1119	1129	0	---	R.STGTLLNNAIK.G
1250.6191	1249.6118	1249.6667	-43.88	350	360	0	---	K.LSFVTVDSNLR.R
1250.6191	1249.6119	1249.6667	-43.85	350	360	0	---	K.LSFVTVDSNLR.R
1287.6302	1286.6229	1286.6830	-46.71	777	789	0	---	R.DTGLGLSPTASLR.V
1302.6307	1301.6234	1301.6762	-40.52	587	598	0	---	R.VTASPQSLCALR.A
1421.6768	1420.6695	1420.7350	-46.15	455	466	0	14	R.SNSFVYLEPLPR.E
1421.6768	1420.6695	1420.7350	-46.12	455	466	0	---	R.SNSFVYLEPLPR.E
1479.7271	1478.7198	1478.6422	52.5	667	678	0	---	K.DMYSFLQDMGLK.A + 2 Oxidation (M)
1491.6775	1490.6702	1490.7340	-42.81	82	93	0	---	K.DLFHCVSFTLPR.S
1523.7185	1522.7112	1522.7814	-46.06	983	997	0	---	R.ASFSVLGDILGSAMR.N
1707.8094	1706.8021	1706.8913	-52.25	94	108	0	---	R.SPTSQEVMFLTIQVK.G
1900.9595	1899.9522	1900.0306	-41.25	540	557	0	13	R.LLIYAILPDGEVVGDSAR.Y
1900.9595	1899.9522	1900.0306	-41.23	540	557	0	---	R.LLIYAILPDGEVVGDSAR.Y
1991.8297	1990.8224	1990.8909	-34.39	718	734	0	---	R.DFVHFDDTSEPPTETVR.K
2001.9189	2000.9116	2000.9844	-36.36	210	228	0	---	K.GSGGTAEHPFTVEEFVLPK.F
2012.9465	2011.9392	2012.0228	-41.54	568	586	0	---	K.VGLNFSPGQSFPASQAHLR.V
2012.9465	2011.9393	2012.0228	-41.53	568	586	0	---	K.VGLNFSPGQSFPASQAHLR.V
2073.0088	2072.0015	2072.0805	-38.12	790	806	0	---	R.VFQPFFVELTMPYSVIR.G
2927.5508	2926.5435	2926.5837	-13.74	599	625	0	---	R.AVDQSVLLMRPEAELSAATVYNLLPVK.D

No match to: 842.4690, 848.4879, 850.0154, 850.4973, 853.4743, 853.4743, 861.0195, 864.5118, 865.9869, 868.5078, 870.5034, 876.9944, 881.9677, 882.5228, 882.5228, 892.9686, 951.5814, 953.4899, 1017.6209, 1017.6209, 1019.6330, 1033.6514, 1045.5168, 1051.6565, 1051.6565, 1136.6420, 1147.6807, 1151.6740, 1165.6849, 1165.6849, 1300.7900, 1316.8218, 1334.8221, 1334.8221, 1356.6021, 1448.8531, 1454.7075, 1475.6875, 1518.6587, 1552.7145, 1559.7947, 1567.6738, 1617.9915, 1617.9915, 1732.0206, 2015.1936, 2044.9634, 2193.0256, 2211.0200, 2230.1453, 2233.0505, 2239.0674, 2283.0967, 2284.0908, 2305.0867, 3337.7156

6. A0A4W2CTC9 **Mass:** 169552 **Score:** 78 **Expect:** 0.0023 **Matches:** 20

Alpha-2-macroglobulin OS=Bos indicus x Bos taurus OX=30522 GN=A2M PE=4 SV=1

Observed	Mr(expt)	Mr(calc)	ppm	Start	End	Miss	Ions	Peptide
996.5488	995.5415	995.4607	81.2	1111	1118	1	---	K.QKSNGCFR.S
1131.6523	1130.6450	1130.6295	13.7	1119	1129	0	---	R.STGTLLNNAIK.G
1250.6191	1249.6118	1249.6667	-43.88	350	360	0	---	K.LSFVTVDSNLR.R
1250.6191	1249.6119	1249.6667	-43.85	350	360	0	---	K.LSFVTVDSNLR.R
1287.6302	1286.6229	1286.6830	-46.71	777	789	0	---	R.DTGLGLSPTASLR.V
1302.6307	1301.6234	1301.6762	-40.52	587	598	0	---	R.VTASPQSLCALR.A
1421.6768	1420.6695	1420.7350	-46.15	455	466	0	14	R.SNSFVYLEPLPR.E
1421.6768	1420.6695	1420.7350	-46.12	455	466	0	---	R.SNSFVYLEPLPR.E
1479.7271	1478.7198	1478.6422	52.5	667	678	0	---	K.DMYSFLQDMGLK.A + 2 Oxidation (M)
1491.6775	1490.6702	1490.7340	-42.81	82	93	0	---	K.DLFHCVSFTLPR.S
1523.7185	1522.7112	1522.7814	-46.06	983	997	0	---	R.ASFSVLGDILGSAMR.N
1707.8094	1706.8021	1706.8913	-52.25	94	108	0	---	R.SPTSQEVMFLTIQVK.G
1900.9595	1899.9522	1900.0306	-41.25	540	557	0	13	R.LLIYAILPDGEVVGDSAR.Y
1900.9595	1899.9522	1900.0306	-41.23	540	557	0	---	R.LLIYAILPDGEVVGDSAR.Y
1991.8297	1990.8224	1990.8909	-34.39	718	734	0	---	R.DFVHFDDTSEPPTETVR.K
2001.9189	2000.9116	2000.9844	-36.36	210	228	0	---	K.GSGGTAEHPFTVEEFVLPK.F
2012.9465	2011.9392	2012.0228	-41.54	568	586	0	---	K.VGLNFSPGQSFPASQAHLR.V
2012.9465	2011.9393	2012.0228	-41.53	568	586	0	---	K.VGLNFSPGQSFPASQAHLR.V
2073.0088	2072.0015	2072.0805	-38.12	790	806	0	---	R.VFQPFFVELTMPYSVIR.G
2927.5508	2926.5435	2926.5837	-13.74	599	625	0	---	R.AVDQSVLLMRPEAELSAATVYNLLPVK.D

No match to: 842.4690, 848.4879, 850.0154, 850.4973, 853.4743, 853.4743, 861.0195, 864.5118, 865.9869, 868.5078, 870.5034, 876.9944, 881.9677, 882.5228, 882.5228, 892.9686, 951.5814, 953.4899, 1017.6209, 1017.6209, 1019.6330, 1033.6514, 1045.5168, 1051.6565, 1051.6565, 1136.6420, 1147.6807, 1151.6740, 1165.6849, 1165.6849, 1300.7900, 1316.8218, 1334.8221, 1334.8221, 1356.6021, 1448.8531, 1454.7075, 1475.6875, 1518.6587, 1552.7145, 1559.7947, 1567.6738, 1617.9915, 1617.9915, 1732.0206, 2015.1936, 2044.9634, 2193.0256, 2211.0200, 2230.1453, 2233.0505, 2239.0674, 2283.0967, 2284.0908, 2305.0867, 3337.7156

7. K4JBR5 **Mass:** 116087 **Score:** 75 **Expect:** 0.0041 **Matches:** 16

Alpha-2-macroglobulin variant 1 OS=Bos taurus OX=9913 GN=A2M PE=2 SV=1

Observed	Mr(expt)	Mr(calc)	ppm	Start	End	Miss	Ions	Peptide
1250.6191	1249.6118	1249.6667	-43.88	350	360	0	---	K.LSFVTVDSNLR.R
1250.6191	1249.6119	1249.6667	-43.85	350	360	0	---	K.LSFVTVDSNLR.R
1287.6302	1286.6229	1286.6830	-46.71	777	789	0	---	R.DTGLGLSPTASLR.V
1302.6307	1301.6234	1301.6762	-40.52	587	598	0	---	R.VTASPQSLCALR.A
1421.6768	1420.6695	1420.7350	-46.15	455	466	0	14	R.SNSFVYLEPLPR.E
1421.6768	1420.6695	1420.6367	23.1	667	678	0	---	K.DMYSFLQGMGLK.A + 2 Oxidation (M)
1491.6775	1490.6702	1490.7340	-42.81	82	93	0	---	K.DLFHCVSFTLPR.S
1707.8094	1706.8021	1706.8913	-52.25	94	108	0	---	R.SPTSQEVMFLTIQVK.G
1900.9595	1899.9522	1900.0306	-41.25	540	557	0	13	R.LLIYAILPDGEVVGDSAR.Y
1900.9595	1899.9522	1900.0306	-41.23	540	557	0	---	R.LLIYAILPDGEVVGDSAR.Y
1991.8297	1990.8224	1990.8909	-34.39	718	734	0	---	R.DFVHFDDTSEPPTETVR.K
2001.9189	2000.9116	2000.9844	-36.36	210	228	0	---	K.GSGGTAEHPFTVEEFVLPK.F
2012.9465	2011.9392	2012.0228	-41.54	568	586	0	---	K.VGLNFSPGQSFPASQAHLR.V
2012.9465	2011.9393	2012.0228	-41.53	568	586	0	---	K.VGLNFSPGQSFPASQAHLR.V
2073.0088	2072.0015	2072.0805	-38.12	790	806	0	---	R.VFQPFFVELTMPYSVIR.G
2927.5508	2926.5435	2926.5837	-13.74	599	625	0	---	R.AVDQSVLLMRPEAELSAATVYNLLPVK.D

No match to: 842.4690, 848.4879, 850.0154, 850.4973, 853.4743, 853.4743, 861.0195, 864.5118, 865.9869, 868.5078, 870.5034, 876.9944, 881.9677, 882.5228, 882.5228, 892.9686, 951.5814, 953.4899, 996.5488, 1017.6209, 1017.6209, 1019.6330, 1033.6514, 1045.5168, 1051.6565, 1051.6565, 1131.6523, 1136.6420, 1147.6807, 1151.6740, 1165.6849, 1165.6849, 1300.7900, 1316.8218, 1334.8221, 1334.8221, 1356.6021, 1448.8531, 1454.7075, 1475.6875, 1479.7271, 1518.6587, 1523.7185, 1552.7145, 1559.7947, 1567.6738, 1617.9915, 1617.9915, 1732.0206, 2015.1936, 2044.9634, 2193.0256, 2211.0200, 2230.1453, 2233.0505, 2239.0674, 2283.0967, 2284.0908, 2305.0867, 3337.7156

8. K4JDT2 **Mass:** 99307 **Score:** 68 **Expect:** 0.02 **Matches:** 15

Alpha-2-macroglobulin variant 20 OS=Bos taurus OX=9913 GN=A2M PE=2 SV=1

Observed	Mr(expt)	Mr(calc)	ppm	Start	End	Miss	Ions	Peptide
1250.6191	1249.6118	1249.6667	-43.88	350	360	0	---	K.LSFVTVDSNLR.R
1250.6191	1249.6119	1249.6667	-43.85	350	360	0	---	K.LSFVTVDSNLR.R
1287.6302	1286.6229	1286.6830	-46.71	777	789	0	---	R.DTGLGLSPTASLR.V
1421.6768	1420.6695	1420.7350	-46.15	455	466	0	14	R.SNSFVYLEPLPR.E
1421.6768	1420.6695	1420.7350	-46.12	455	466	0	---	R.SNSFVYLEPLPR.E
1479.7271	1478.7198	1478.6422	52.5	667	678	0	---	K.DMYSFLQDMGLK.A + 2 Oxidation (M)

PDF-XChange Click to buy NOW! www.docu-track.com

PDF-XChange Click to buy NOW! www.docu-track.com

1491.6775	1490.6702	1490.7340	-42.81	82 -	93	0	---	K.DLFHCVSFTLPR.S
1707.8094	1706.8021	1706.8913	-52.25	94 -	108	0	---	R.SPTSQEVMFLTIQVK.G
1900.9595	1899.9522	1900.0306	-41.25	540 -	557	0	13	R.LLIYAILPDGEVVGDSAR.Y
1900.9595	1899.9522	1900.0306	-41.23	540 -	557	0	---	R.LLIYAILPDGEVVGDSAR.Y
1991.8297	1990.8224	1990.8909	-34.39	718 -	734	0	---	R.DFVHFDDTSEPPTETVR.K
2001.9189	2000.9116	2000.9844	-36.36	210 -	228	0	---	K.GSGGTAEHPFTVEEFVLPK.F
2012.9465	2011.9392	2012.0228	-41.54	568 -	586	0	---	K.VGLNFSPGQSFPASQAHLR.V
2012.9465	2011.9393	2012.0228	-41.53	568 -	586	0	---	K.VGLNFSPGQSFPASQAHLR.V
2073.0088	2072.0015	2072.0805	-38.12	790 -	806	0	---	R.VFQPFFVELTMPYSVIR.G

No match to: 842.4690, 848.4879, 850.0154, 850.4973, 853.4743, 853.4743, 861.0195, 864.5118, 865.9869, 868.5078, 870.5034, 876.9944, 881.9677, 882.5228, 882.5228, 892.9686, 951.5814, 953.4899, 996.5488, 1017.6209, 1017.6209, 1019.6330, 1033.6514, 1045.5168, 1051.6565, 1051.6565, 1131.6523, 1136.6420, 1147.6807, 1151.6740, 1165.6849, 1165.6849, 1300.7900, 1302.6307, 1316.8218, 1334.8221, 1334.8221, 1356.6021, 1448.8531, 1454.7075, 1475.6875, 1518.6587, 1523.7185, 1552.7145, 1559.7947, 1567.6738, 1617.9915, 1617.9915, 1732.0206, 2015.1936, 2044.9634, 2193.0256, 2211.0200, 2230.1453, 2233.0505, 2239.0674, 2283.0967, 2284.0908, 2305.0867, 2927.5508, 3337.7156

9. A0A4W2FQH1 **Mass:** 164938 **Score:** 59 **Expect:** 0.17 **Matches:** 18

Alpha-2-macroglobulin OS=Bos indicus x Bos taurus OX=30522 GN=A2M PE=4 SV=1

Observed	Mr(expt)	Mr(calc)	ppm	Start	End	Miss	Ions	Peptide
996.5488	995.5415	995.4607	81.2	1073 -	1080	1	---	K.QKSNGCFR.S
1131.6523	1130.6450	1130.6295	13.7	1081 -	1091	0	---	R.STGTLLNNAIK.G
1250.6191	1249.6118	1249.6667	-43.88	350 -	360	0	---	K.LSFVTVDSNLR.R
1250.6191	1249.6119	1249.6667	-43.85	350 -	360	0	---	K.LSFVTVDSNLR.R
1287.6302	1286.6229	1286.6830	-46.71	775 -	787	0	---	R.DTGLGLSPTASLR.V
1302.6307	1301.6234	1301.6762	-40.52	587 -	598	0	---	R.VTASPQSLCALR.A
1479.7271	1478.7198	1478.6422	52.5	667 -	678	0	---	K.DMYSFLQDMGLK.A + 2 Oxidation (M)
1491.6775	1490.6702	1490.7340	-42.81	82 -	93	0	---	K.DLFHCVSFTLPR.S
1523.7185	1522.7112	1522.7814	-46.06	945 -	959	0	---	R.ASFSVLGDILGSAMR.N
1707.8094	1706.8021	1706.8913	-52.25	94 -	108	0	---	R.SPTSQEVMFLTIQVK.G
1900.9595	1899.9522	1900.0306	-41.25	540 -	557	0	13	R.LLIYAILPDGEVVGDSAR.Y
1900.9595	1899.9522	1900.0306	-41.23	540 -	557	0	---	R.LLIYAILPDGEVVGDSAR.Y
1991.8297	1990.8224	1990.8909	-34.39	716 -	732	0	---	R.DFVHFDDTSEPPTETVR.K
2001.9189	2000.9116	2000.9844	-36.36	210 -	228	0	---	K.GSGGTAEHPFTVEEFVLPK.F
2012.9465	2011.9392	2012.0228	-41.54	568 -	586	0	---	K.VGLNFSPGQSFPASQAHLR.V
2012.9465	2011.9393	2012.0228	-41.53	568 -	586	0	---	K.VGLNFSPGQSFPASQAHLR.V
2073.0088	2072.0015	2072.0805	-38.12	788 -	804	0	---	R.VFQPFFVELTMPYSVIR.G
2927.5508	2926.5435	2926.5837	-13.74	599 -	625	0	---	R.AVDQSVLLMRPEAELSAATVYNLLPVK.D

No match to: 842.4690, 848.4879, 850.0154, 850.4973, 853.4743, 853.4743, 861.0195, 864.5118, 865.9869, 868.5078, 870.5034, 876.9944, 881.9677, 882.5228, 882.5228, 892.9686, 951.5814, 953.4899, 1017.6209, 1017.6209, 1019.6330, 1033.6514, 1045.5168, 1051.6565, 1051.6565, 1136.6420, 1147.6807, 1151.6740, 1165.6849, 1165.6849, 1300.7900, 1316.8218, 1334.8221, 1334.8221, 1356.6021, 1421.6768, 1421.6768, 1448.8531, 1454.7075, 1475.6875, 1518.6587, 1552.7145, 1559.7947, 1567.6738, 1617.9915, 1617.9915, 1732.0206, 2015.1936, 2044.9634, 2193.0256, 2211.0200, 2230.1453, 2233.0505, 2239.0674, 2283.0967, 2284.0908, 2305.0867, 3337.7156

10. A0A4W2FQJ3 **Mass:** 166227 **Score:** 59 **Expect:** 0.19 **Matches:** 18

Alpha-2-macroglobulin OS=Bos indicus x Bos taurus OX=30522 GN=A2M PE=4 SV=1

Observed	Mr(expt)	Mr(calc)	ppm	Start	End	Miss	Ions	Peptide
996.5488	995.5415	995.4607	81.2	1111 -	1118	1	---	K.QKSNGCFR.S
1131.6523	1130.6450	1130.6295	13.7	1119 -	1129	0	---	R.STGTLLNNAIK.G
1250.6191	1249.6118	1249.6667	-43.88	350 -	360	0	---	K.LSFVTVDSNLR.R
1250.6191	1249.6119	1249.6667	-43.85	350 -	360	0	---	K.LSFVTVDSNLR.R
1287.6302	1286.6229	1286.6830	-46.71	777 -	789	0	---	R.DTGLGLSPTASLR.V
1302.6307	1301.6234	1301.6762	-40.52	587 -	598	0	---	R.VTASPQSLCALR.A
1479.7271	1478.7198	1478.6422	52.5	667 -	678	0	---	K.DMYSFLQDMGLK.A + 2 Oxidation (M)
1491.6775	1490.6702	1490.7340	-42.81	82 -	93	0	---	K.DLFHCVSFTLPR.S
1523.7185	1522.7112	1522.7814	-46.06	983 -	997	0	---	R.ASFSVLGDILGSAMR.N
1707.8094	1706.8021	1706.8913	-52.25	94 -	108	0	---	R.SPTSQEVMFLTIQVK.G
1900.9595	1899.9522	1900.0306	-41.25	540 -	557	0	13	R.LLIYAILPDGEVVGDSAR.Y
1900.9595	1899.9522	1900.0306	-41.23	540 -	557	0	---	R.LLIYAILPDGEVVGDSAR.Y
1991.8297	1990.8224	1990.8909	-34.39	718 -	734	0	---	R.DFVHFDDTSEPPTETVR.K
2001.9189	2000.9116	2000.9844	-36.36	210 -	228	0	---	K.GSGGTAEHPFTVEEFVLPK.F
2012.9465	2011.9392	2012.0228	-41.54	568 -	586	0	---	K.VGLNFSPGQSFPASQAHLR.V
2012.9465	2011.9393	2012.0228	-41.53	568 -	586	0	---	K.VGLNFSPGQSFPASQAHLR.V
2073.0088	2072.0015	2072.0805	-38.12	790 -	806	0	---	R.VFQPFFVELTMPYSVIR.G
2927.5508	2926.5435	2926.5837	-13.74	599 -	625	0	---	R.AVDQSVLLMRPEAELSAATVYNLLPVK.D

No match to: 842.4690, 848.4879, 850.0154, 850.4973, 853.4743, 853.4743, 861.0195, 864.5118, 865.9869, 868.5078, 870.5034, 876.9944, 881.9677, 882.5228, 882.5228, 892.9686, 951.5814, 953.4899, 1017.6209, 1017.6209, 1019.6330, 1033.6514, 1045.5168, 1051.6565, 1051.6565, 1136.6420, 1147.6807, 1151.6740, 1165.6849, 1165.6849, 1300.7900, 1316.8218, 1334.8221, 1334.8221, 1356.6021, 1421.6768, 1421.6768, 1448.8531, 1454.7075, 1475.6875, 1518.6587, 1552.7145, 1559.7947, 1567.6738, 1617.9915, 1617.9915, 1732.0206, 2015.1936, 2044.9634, 2193.0256, 2211.0200, 2230.1453, 2233.0505, 2239.0674, 2283.0967, 2284.0908, 2305.0867, 3337.7156

11. A0A4W2FQM6 **Mass:** 168925 **Score:** 58 **Expect:** 0.22 **Matches:** 18

Alpha-2-macroglobulin OS=Bos indicus x Bos taurus OX=30522 GN=A2M PE=4 SV=1

Observed	Mr(expt)	Mr(calc)	ppm	Start	End	Miss	Ions	Peptide
996.5488	995.5415	995.4607	81.2	1111 -	1118	1	---	K.QKSNGCFR.S
1131.6523	1130.6450	1130.6295	13.7	1119 -	1129	0	---	R.STGTLLNNAIK.G
1250.6191	1249.6118	1249.6667	-43.88	350 -	360	0	---	K.LSFVTVDSNLR.R
1250.6191	1249.6119	1249.6667	-43.85	350 -	360	0	---	K.LSFVTVDSNLR.R
1287.6302	1286.6229	1286.6830	-46.71	777 -	789	0	---	R.DTGLGLSPTASLR.V
1302.6307	1301.6234	1301.6762	-40.52	587 -	598	0	---	R.VTASPQSLCALR.A
1479.7271	1478.7198	1478.6422	52.5	667 -	678	0	---	K.DMYSFLQDMGLK.A + 2 Oxidation (M)
1491.6775	1490.6702	1490.7340	-42.81	82 -	93	0	---	K.DLFHCVSFTLPR.S
1523.7185	1522.7112	1522.7814	-46.06	983 -	997	0	---	R.ASFSVLGDILGSAMR.N
1707.8094	1706.8021	1706.8913	-52.25	94 -	108	0	---	R.SPTSQEVMFLTIQVK.G
1900.9595	1899.9522	1900.0306	-41.25	540 -	557	0	13	R.LLIYAILPDGEVVGDSAR.Y
1900.9595	1899.9522	1900.0306	-41.23	540 -	557	0	---	R.LLIYAILPDGEVVGDSAR.Y
1991.8297	1990.8224	1990.8909	-34.39	718 -	734	0	---	R.DFVHFDDTSEPPTETVR.K
2001.9189	2000.9116	2000.9844	-36.36	210 -	228	0	---	K.GSGGTAEHPFTVEEFVLPK.F
2012.9465	2011.9392	2012.0228	-41.54	568 -	586	0	---	K.VGLNFSPGQSFPASQAHLR.V
2012.9465	2011.9393	2012.0228	-41.53	568 -	586	0	---	K.VGLNFSPGQSFPASQAHLR.V
2073.0088	2072.0015	2072.0805	-38.12	790 -	806	0	---	R.VFQPFFVELTMPYSVIR.G
2927.5508	2926.5435	2926.5837	-13.74	599 -	625	0	---	R.AVDQSVLLMRPEAELSAATVYNLLPVK.D

PDF-XChange Click to buy NOW! www.docu-track.com
PDF-XChange Click to buy NOW! www.docu-track.com

No match to: 842.4690, 848.4879, 850.0154, 850.4973, 853.4743, 853.4743, 861.0195, 864.5118, 865.9869, 868.5078, 870.5034, 876.9944, 881.9677, 882.5228, 882.5228, 892.9686, 951.5814, 953.4899, 1017.6209, 1017.6209, 1019.6330, 1033.6514, 1045.5168, 1051.6565, 1051.6565, 1136.6420, 1147.6807, 1151.6740, 1165.6849, 1165.6849, 1300.7900, 1316.8218, 1334.8221, 1334.8221, 1356.6021, 1421.6768, 1421.6768, 1448.8531, 1454.7075, 1475.6875, 1518.6587, 1552.7145, 1559.7947, 1567.6738, 1617.9915, 1617.9915, 1732.0206, 2015.1936, 2044.9634, 2193.0256, 2211.0200, 2230.1453, 2233.0505, 2239.0674, 2283.0967, 2284.0908, 2305.0867, 3337.7156

12. A0A4W2HMW5 **Mass:** 168978 **Score:** 58 **Expect:** 0.22 **Matches:** 18

Alpha-2-macroglobulin OS=Bos indicus x Bos taurus OX=30522 GN=A2M PE=4 SV=1

Observed	Mr(expt)	Mr(calc)	ppm	Start	End	Miss	Ions	Peptide
996.5488	995.5415	995.4607	81.2	1111	1118	1	---	K.QKSNGCFR.S
1131.6523	1130.6450	1130.6295	13.7	1119	1129	0	---	R.STGTLLNNAIK.G
1250.6191	1249.6118	1249.6667	-43.88	350	360	0	---	K.LSFVTVDSNLR.R
1250.6191	1249.6119	1249.6667	-43.85	350	360	0	---	K.LSFVTVDSNLR.R
1287.6302	1286.6229	1286.6830	-46.71	777	789	0	---	R.DTGLGLSPTASLR.V
1302.6307	1301.6234	1301.6762	-40.52	587	598	0	---	R.VTASPQSLCALR.A
1479.7271	1478.7198	1478.6422	52.5	667	678	0	---	K.DMYSFLQDMGLK.A + 2 Oxidation (M)
1491.6775	1490.6702	1490.7340	-42.81	82	93	0	---	K.DLFHCVSFTLPR.S
1523.7185	1522.7112	1522.7814	-46.06	983	997	0	---	R.ASFSVLGDILGSAMR.N
1707.8094	1706.8021	1706.8913	-52.25	94	108	0	---	R.SPTSQEVMFLTIQVK.G
1900.9595	1899.9522	1900.0306	-41.25	540	557	0	13	R.LLIYAILPDGEVVGDSAR.Y
1900.9595	1899.9522	1900.0306	-41.23	540	557	0	---	R.LLIYAILPDGEVVGDSAR.Y
1991.8297	1990.8224	1990.8909	-34.39	718	734	0	---	R.DFVHFDDTSEPPTETVR.K
2001.9189	2000.9116	2000.9844	-36.36	210	228	0	---	K.GSGGTAEHPFTVEEFVLPK.F
2012.9465	2011.9392	2012.0228	-41.54	568	586	0	---	K.VGLNFSPGQSFPASQAHLR.V
2012.9465	2011.9393	2012.0228	-41.53	568	586	0	---	K.VGLNFSPGQSFPASQAHLR.V
2073.0088	2072.0015	2072.0805	-38.12	790	806	0	---	R.VFQPFFVELTMPYSVIR.G
2927.5508	2926.5435	2926.5837	-13.74	599	625	0	---	R.AVDQSVLLMRPEAELSAATVYNLLPVK.D

No match to: 842.4690, 848.4879, 850.0154, 850.4973, 853.4743, 853.4743, 861.0195, 864.5118, 865.9869, 868.5078, 870.5034, 876.9944, 881.9677, 882.5228, 882.5228, 892.9686, 951.5814, 953.4899, 1017.6209, 1017.6209, 1019.6330, 1033.6514, 1045.5168, 1051.6565, 1051.6565, 1136.6420, 1147.6807, 1151.6740, 1165.6849, 1165.6849, 1300.7900, 1316.8218, 1334.8221, 1334.8221, 1356.6021, 1421.6768, 1421.6768, 1448.8531, 1454.7075, 1475.6875, 1518.6587, 1552.7145, 1559.7947, 1567.6738, 1617.9915, 1617.9915, 1732.0206, 2015.1936, 2044.9634, 2193.0256, 2211.0200, 2230.1453, 2233.0505, 2239.0674, 2283.0967, 2284.0908, 2305.0867, 3337.7156

13. A0A4W2HM51 **Mass:** 168906 **Score:** 58 **Expect:** 0.22 **Matches:** 18

Alpha-2-macroglobulin OS=Bos indicus x Bos taurus OX=30522 GN=A2M PE=4 SV=1

Observed	Mr(expt)	Mr(calc)	ppm	Start	End	Miss	Ions	Peptide
996.5488	995.5415	995.4607	81.2	1111	1118	1	---	K.QKSNGCFR.S
1131.6523	1130.6450	1130.6295	13.7	1119	1129	0	---	R.STGTLLNNAIK.G
1250.6191	1249.6118	1249.6667	-43.88	350	360	0	---	K.LSFVTVDSNLR.R
1250.6191	1249.6119	1249.6667	-43.85	350	360	0	---	K.LSFVTVDSNLR.R
1287.6302	1286.6229	1286.6830	-46.71	777	789	0	---	R.DTGLGLSPTASLR.V
1302.6307	1301.6234	1301.6762	-40.52	587	598	0	---	R.VTASPQSLCALR.A
1479.7271	1478.7198	1478.6422	52.5	667	678	0	---	K.DMYSFLQDMGLK.A + 2 Oxidation (M)
1491.6775	1490.6702	1490.7340	-42.81	82	93	0	---	K.DLFHCVSFTLPR.S
1523.7185	1522.7112	1522.7814	-46.06	983	997	0	---	R.ASFSVLGDILGSAMR.N
1707.8094	1706.8021	1706.8913	-52.25	94	108	0	---	R.SPTSQEVMFLTIQVK.G
1900.9595	1899.9522	1900.0306	-41.25	540	557	0	13	R.LLIYAILPDGEVVGDSAR.Y
1900.9595	1899.9522	1900.0306	-41.23	540	557	0	---	R.LLIYAILPDGEVVGDSAR.Y
1991.8297	1990.8224	1990.8909	-34.39	718	734	0	---	R.DFVHFDDTSEPPTETVR.K
2001.9189	2000.9116	2000.9844	-36.36	210	228	0	---	K.GSGGTAEHPFTVEEFVLPK.F
2012.9465	2011.9392	2012.0228	-41.54	568	586	0	---	K.VGLNFSPGQSFPASQAHLR.V
2012.9465	2011.9393	2012.0228	-41.53	568	586	0	---	K.VGLNFSPGQSFPASQAHLR.V
2073.0088	2072.0015	2072.0805	-38.12	790	806	0	---	R.VFQPFFVELTMPYSVIR.G
2927.5508	2926.5435	2926.5837	-13.74	599	625	0	---	R.AVDQSVLLMRPEAELSAATVYNLLPVK.D

No match to: 842.4690, 848.4879, 850.0154, 850.4973, 853.4743, 853.4743, 861.0195, 864.5118, 865.9869, 868.5078, 870.5034, 876.9944, 881.9677, 882.5228, 882.5228, 892.9686, 951.5814, 953.4899, 1017.6209, 1017.6209, 1019.6330, 1033.6514, 1045.5168, 1051.6565, 1051.6565, 1136.6420, 1147.6807, 1151.6740, 1165.6849, 1165.6849, 1300.7900, 1316.8218, 1334.8221, 1334.8221, 1356.6021, 1421.6768, 1421.6768, 1448.8531, 1454.7075, 1475.6875, 1518.6587, 1552.7145, 1559.7947, 1567.6738, 1617.9915, 1617.9915, 1732.0206, 2015.1936, 2044.9634, 2193.0256, 2211.0200, 2230.1453, 2233.0505, 2239.0674, 2283.0967, 2284.0908, 2305.0867, 3337.7156

14. A0A4W2BQ12 **Mass:** 48807 **Score:** 41 **Expect:** 12 **Matches:** 11

Tripartite motif containing 77 OS=Bos indicus x Bos taurus OX=30522 GN=TRIM77 PE=4 SV=1

Observed	Mr(expt)	Mr(calc)	ppm	Start	End	Miss	Ions	Peptide
842.4690	841.4617	841.4882	-31.47	212	218	1	---	R.LNRNVAR.M
1045.5168	1044.5095	1044.5096	-0.07	138	145	0	---	R.EHLLMQMK.S + Oxidation (M)
1136.6420	1135.6347	1135.5510	73.8	20	29	0	---	K.DTFTDPATIR.C
1300.7900	1299.7827	1299.6540	99.1	91	100	1	---	K.QMCRTHQVIK.L
1518.6587	1517.6514	1517.7508	-65.47	76	90	0	---	R.STGNSVICQLPGSAK.Q
1559.7947	1558.7874	1558.8000	-8.04	138	149	1	---	R.EHLLMQMKSIWK.K + Oxidation (M)
1567.6738	1566.6665	1566.6906	-15.35	240	253	0	---	K.ADVDMLQGVEDTMK.R + Oxidation (M)
1707.8094	1706.8021	1706.7968	3.15	240	254	1	---	K.ADVDMLQGVEDTMKR.S
2012.9465	2011.9392	2012.0877	-73.79	195	211	1	---	K.QKHLEMLAIEGTVIFQR.L
2012.9465	2011.9393	2012.0877	-73.77	195	211	1	---	K.QKHLEMLAIEGTVIFQR.L
2193.0256	2192.0183	2192.1333	-52.46	366	383	1	---	R.NDMLLNSEDIFLLLCVKR.K

No match to: 848.4879, 850.0154, 850.4973, 853.4743, 853.4743, 861.0195, 864.5118, 865.9869, 868.5078, 870.5034, 876.9944, 881.9677, 882.5228, 882.5228, 892.9686, 951.5814, 953.4899, 996.5488, 1017.6209, 1017.6209, 1019.6330, 1033.6514, 1051.6565, 1051.6565, 1131.6523, 1147.6807, 1151.6740, 1165.6849, 1165.6849, 1250.6191, 1250.6191, 1287.6302, 1302.6307, 1316.8218, 1334.8221, 1334.8221, 1356.6021, 1421.6768, 1421.6768, 1448.8531, 1454.7075, 1475.6875, 1479.7271, 1491.6775, 1523.7185, 1552.7145, 1617.9915, 1617.9915, 1732.0206, 1900.9595, 1900.9595, 1991.8297, 2001.9189, 2015.1936, 2044.9634, 2073.0088, 2211.0200, 2230.1453, 2233.0505, 2239.0674, 2283.0967, 2284.0908, 2305.0867, 2927.5508, 3337.7156

15. A0A4W2FVS5 **Mass:** 48837 **Score:** 41 **Expect:** 12 **Matches:** 11

Uncharacterized protein OS=Bos indicus x Bos taurus OX=30522 GN=LOC113885841 PE=4 SV=1

Observed	Mr(expt)	Mr(calc)	ppm	Start	End	Miss	Ions	Peptide
842.4690	841.4617	841.4882	-31.47	212	218	1	---	R.LNRNVAR.M
1045.5168	1044.5095	1044.5096	-0.07	138	145	0	---	R.EHLLMQMK.S + Oxidation (M)
1136.6420	1135.6347	1135.5510	73.8	20	29	0	---	K.DTFTDPATIR.C
1300.7900	1299.7827	1299.6540	99.1	91	100	1	---	K.QMCRTHQVIK.L
1518.6587	1517.6514	1517.7508	-65.47	76	90	0	---	R.STGNSVICQLPGSAK.Q
1559.7947	1558.7874	1558.8000	-8.04	138	149	1	---	R.EHLLMQMKSIWK.K + Oxidation (M)

PDF-XChange Click to buy NOW! www.docu-track.com

PDF-XChange Click to buy NOW! www.docu-track.com

1567.6738	1566.6665	1566.6906	-15.35	240 - 253	0	---	K.ADVDMLQGVEDTMK.R + Oxidation (M)
1707.8094	1706.8021	1706.7968	3.15	240 - 254	1	---	K.ADVDMLQGVEDTMKR.S
2012.9465	2011.9392	2012.0877	-73.79	195 - 211	1	---	K.QKHLEMLAIEGTVIFQR.L
2012.9465	2011.9393	2012.0877	-73.77	195 - 211	1	---	K.QKHLEMLAIEGTVIFQR.L
2193.0256	2192.0183	2192.1333	-52.46	366 - 383	1	---	R.NDMLLNSEDIFLLLCVKR.K

No match to: 848.4879, 850.0154, 850.4973, 853.4743, 853.4743, 861.0195, 864.5118, 865.9869, 868.5078, 870.5034, 876.9944, 881.9677, 882.5228, 882.5228, 892.9686, 951.5814, 953.4899, 996.5488, 1017.6209, 1017.6209, 1019.6330, 1033.6514, 1051.6565, 1051.6565, 1131.6523, 1147.6807, 1151.6740, 1165.6849, 1165.6849, 1250.6191, 1250.6191, 1287.6302, 1302.6307, 1316.8218, 1334.8221, 1334.8221, 1356.6021, 1421.6768, 1421.6768, 1448.8531, 1454.7075, 1475.6875, 1479.7271, 1491.6775, 1523.7185, 1552.7145, 1617.9915, 1617.9915, 1732.0206, 1900.9595, 1900.9595, 1991.8297, 2001.9189, 2015.1936, 2044.9634, 2073.0088, 2211.0200, 2230.1453, 2233.0505, 2239.0674, 2283.0967, 2284.0908, 2305.0867, 2927.5508, 3337.7156

16. A0A1L7BC46 **Mass:** 55660 **Score:** 39 **Expect:** 15 **Matches:** 10
NS5A (Fragment) OS=Bovine viral diarrhea virus OX=11099 PE=4 SV=1

Observed	Mr(expt)	Mr(calc)	ppm	Start - End	Miss	Ions	Peptide
1033.6514	1032.6441	1032.5716	70.2	91 - 99	1	---	R.GPVNYRVTK.Y
1045.5168	1044.5095	1044.5240	-13.88	459 - 467	0	---	K.YHLVGDVDK.V
1250.6191	1249.6118	1249.6779	-52.84	356 - 366	1	---	K.TARNINLYTGK.D
1250.6191	1249.6119	1249.6779	-52.81	356 - 366	1	---	K.TARNINLYTGK.D
1356.6021	1355.5948	1355.6036	-6.46	57 - 67	1	---	K.CPCGYEMKALK.N
1448.8531	1447.8458	1447.7671	54.4	45 - 56	1	---	R.LPTDNYLRVETK.C
1523.7185	1522.7112	1522.8256	-75.11	143 - 155	1	---	K.WEVEHGVVTRLAK.R
1552.7145	1551.7072	1551.7457	-24.78	387 - 399	0	---	R.DFNPELSELVDFK.G
2001.9189	2000.9116	2001.0935	-90.88	107 - 123	1	---	K.EIKPVAKLEGLVDHYYK.G
2927.5508	2926.5435	2926.3945	50.9	298 - 326	1	---	K.TEPNADGGPSSIKIGLDEGSYPGPGLQDR.T

No match to: 842.4690, 848.4879, 850.0154, 850.4973, 853.4743, 853.4743, 861.0195, 864.5118, 865.9869, 868.5078, 870.5034, 876.9944, 881.9677, 882.5228, 882.5228, 892.9686, 951.5814, 953.4899, 996.5488, 1017.6209, 1017.6209, 1019.6330, 1051.6565, 1051.6565, 1131.6523, 1136.6420, 1147.6807, 1151.6740, 1165.6849, 1165.6849, 1287.6302, 1300.7900, 1302.6307, 1316.8218, 1334.8221, 1334.8221, 1421.6768, 1421.6768, 1454.7075, 1475.6875, 1479.7271, 1491.6775, 1518.6587, 1559.7947, 1567.6738, 1617.9915, 1617.9915, 1707.8094, 1732.0206, 1900.9595, 1900.9595, 1991.8297, 2012.9465, 2012.9465, 2015.1936, 2044.9634, 2073.0088, 2193.0256, 2211.0200, 2230.1453, 2233.0505, 2239.0674, 2283.0967, 2284.0908, 2305.0867, 3337.7156

17. A0A4W2FCR6 **Mass:** 52833 **Score:** 36 **Expect:** 36 **Matches:** 8
Reverse transcriptase domain-containing protein OS=Bos indicus x Bos taurus OX=30522 PE=4 SV=1

Observed	Mr(expt)	Mr(calc)	ppm	Start - End	Miss	Ions	Peptide
848.4879	847.4806	847.4287	61.3	381 - 387	1	---	K.VKEESEK.V
864.5118	863.5045	863.5453	-47.26	440 - 446	1	---	K.RHLLLGR.N
868.5078	867.5005	867.4888	13.5	376 - 382	1	---	K.SFLMKVK.E + Oxidation (M)
1131.6523	1130.6450	1130.5720	64.6	221 - 230	0	---	R.ELPDVQAGFR.K
1356.6021	1355.5948	1355.6642	-51.20	370 - 380	1	---	K.SEEELKSFLMK.V + Oxidation (M)
1479.7271	1478.7198	1478.6922	18.7	75 - 87	1	---	R.NGMDLTEAEDIKK.R + Oxidation (M)
2283.0967	2282.0894	2282.0249	28.3	255 - 272	1	---	K.NICFIDYAKTFDCVDHHK.L
2927.5508	2926.5435	2926.3767	57.0	99 - 123	1	---	K.GLRDPDNHDGMITNLEPDILEYEVR.W + Oxidation (M)

No match to: 842.4690, 850.0154, 850.4973, 853.4743, 853.4743, 861.0195, 865.9869, 870.5034, 876.9944, 881.9677, 882.5228, 882.5228, 892.9686, 951.5814, 953.4899, 996.5488, 1017.6209, 1017.6209, 1019.6330, 1033.6514, 1045.5168, 1051.6565, 1051.6565, 1136.6420, 1147.6807, 1151.6740, 1165.6849, 1165.6849, 1250.6191, 1250.6191, 1287.6302, 1300.7900, 1302.6307, 1316.8218, 1334.8221, 1334.8221, 1421.6768, 1421.6768, 1448.8531, 1454.7075, 1475.6875, 1491.6775, 1518.6587, 1523.7185, 1552.7145, 1559.7947, 1567.6738, 1617.9915, 1617.9915, 1707.8094, 1732.0206, 1900.9595, 1900.9595, 1991.8297, 2001.9189, 2012.9465, 2012.9465, 2015.1936, 2044.9634, 2073.0088, 2193.0256, 2211.0200, 2230.1453, 2233.0505, 2239.0674, 2284.0908, 2305.0867, 3337.7156

18. A0A4W2HM23 **Mass:** 53406 **Score:** 35 **Expect:** 40 **Matches:** 10
Glucosaminyl (N-acetyl) transferase 4 OS=Bos indicus x Bos taurus OX=30522 GN=GCNT4 PE=4 SV=1

Observed	Mr(expt)	Mr(calc)	ppm	Start - End	Miss	Ions	Peptide
996.5488	995.5415	995.5651	-23.72	346 - 355	0	---	R.VPGIPGEISK.T
1033.6514	1032.6441	1032.5968	45.9	435 - 443	1	---	R.KWITLSSAK.L
1131.6523	1130.6450	1130.5760	61.0	315 - 323	0	---	K.YIFNNSFVK.D
1165.6849	1164.6776	1164.6026	64.4	236 - 245	0	---	K.SNFELVSELK.K
1165.6849	1164.6777	1164.6026	64.4	236 - 245	0	---	K.SNFELVSELK.K
1316.8218	1315.8145	1315.6884	95.8	193 - 203	0	---	K.LETVQYAHISR.L
1448.8531	1447.8458	1447.7267	82.3	356 - 368	1	---	K.TAQDVSDLQSKTR.L
1732.0206	1731.0133	1730.8873	72.8	247 - 262	0	---	K.LNGSNMLETVKPPSTK.T + Oxidation (M)
2012.9465	2011.9392	2011.9832	-21.87	315 - 330	1	---	K.YIFNNSFVKDFFAWSK.D
2012.9465	2011.9393	2011.9832	-21.86	315 - 330	1	---	K.YIFNNSFVKDFFAWSK.D

No match to: 842.4690, 848.4879, 850.0154, 850.4973, 853.4743, 853.4743, 861.0195, 864.5118, 865.9869, 868.5078, 870.5034, 876.9944, 881.9677, 882.5228, 882.5228, 892.9686, 951.5814, 953.4899, 1017.6209, 1017.6209, 1019.6330, 1045.5168, 1051.6565, 1051.6565, 1136.6420, 1147.6807, 1151.6740, 1250.6191, 1250.6191, 1287.6302, 1300.7900, 1302.6307, 1334.8221, 1334.8221, 1356.6021, 1421.6768, 1421.6768, 1454.7075, 1475.6875, 1479.7271, 1491.6775, 1518.6587, 1523.7185, 1552.7145, 1559.7947, 1567.6738, 1617.9915, 1617.9915, 1707.8094, 1900.9595, 1900.9595, 1991.8297, 2001.9189, 2015.1936, 2044.9634, 2073.0088, 2193.0256, 2211.0200, 2230.1453, 2233.0505, 2239.0674, 2283.0967, 2284.0908, 2305.0867, 2927.5508, 3337.7156

19. A0A1L7BC66 **Mass:** 56212 **Score:** 35 **Expect:** 46 **Matches:** 11
NS5A (Fragment) OS=Bovine viral diarrhea virus OX=11099 PE=4 SV=1

Observed	Mr(expt)	Mr(calc)	ppm	Start - End	Miss	Ions	Peptide
882.5228	881.5155	881.4494	75.0	129 - 135	1	---	K.IDYSKEK.T
882.5228	881.5155	881.4494	75.0	129 - 135	1	---	K.IDYSKEK.T
1033.6514	1032.6441	1032.5716	70.2	91 - 99	1	---	R.GPVNYRVTK.Y
1131.6523	1130.6450	1130.5866	51.7	370 - 379	1	---	R.EIRDLMAAGR.M
1147.6807	1146.6734	1146.5815	80.2	370 - 379	1	---	R.EIRDLMAAGR.M + Oxidation (M)
1302.6307	1301.6234	1301.7303	-82.12	474 - 485	1	---	K.TLGATDQTRIVK.E
1518.6587	1517.6514	1517.8011	-98.62	284 - 297	0	---	R.ETLMTTGVTPVLEK.V
1523.7185	1522.7112	1522.6861	16.5	387 - 399	0	---	R.DVDPELSEMVDFK.G
1900.9595	1899.9522	1899.9538	-0.83	298 - 315	1	---	K.VEPDASNNQSSVKIGLDK.G
1900.9595	1899.9522	1899.9538	-0.81	298 - 315	1	---	K.VEPDASNNQSSVKIGLDK.G
2001.9189	2000.9116	2000.9990	-43.65	136 - 152	1	---	K.TLLATDKWEVEHGVMTR.L + Oxidation (M)

No match to: 842.4690, 848.4879, 850.0154, 850.4973, 853.4743, 853.4743, 861.0195, 864.5118, 865.9869, 868.5078, 870.5034, 876.9944, 881.9677, 892.9686, 951.5814, 953.4899, 996.5488, 1017.6209, 1017.6209, 1019.6330, 1045.5168, 1051.6565, 1051.6565, 1136.6420, 1151.6740, 1165.6849, 1165.6849, 1250.6191, 1250.6191, 1287.6302, 1300.7900, 1316.8218, 1334.8221, 1334.8221, 1356.6021, 1421.6768, 1421.6768, 1448.8531, 1454.7075, 1475.6875, 1479.7271, 1491.6775, 1552.7145, 1559.7947, 1567.6738, 1617.9915, 1617.9915, 1707.8094, 1732.0206, 1991.8297, 2012.9465, 2012.9465, 2015.1936, 2044.9634, 2073.0088, 2193.0256, 2211.0200, 2230.1453, 2233.0505, 2239.0674, 2283.0967, 2284.0908, 2305.0867, 2927.5508, 3337.7156

20. A0A3Q1MD56 **Mass:** 430029 **Score:** 34 **Expect:** 48 **Matches:** 27
Lysosomal-trafficking regulator OS=Bos taurus OX=9913 GN=LYST PE=4 SV=1

PDF-XChange Click to buy NOW! www.docu-track.com

Observed	Mr(expt)	Mr(calc)	ppm	Start	End	Miss	Ions	Peptide
842.4690	841.4617	841.4545	8.57	86 -	92	0	---	K.IPVQEEK.A
853.4743	852.4670	852.4164	59.4	2381 -	2387	0	---	K.NMGLFQK.W + Oxidation (M)
853.4743	852.4670	852.4164	59.4	2381 -	2387	0	---	K.NMGLFQK.W + Oxidation (M)
1017.6209	1016.6136	1016.5754	37.6	1034 -	1042	0	---	K.EDLLSLTVK.I
1017.6209	1016.6136	1016.5754	37.6	1034 -	1042	0	---	K.EDLLSLTVK.I
1019.6330	1018.6257	1018.5308	93.2	149 -	156	1	---	K.TTHRYSVR.D
1045.5168	1044.5095	1044.5175	-7.64	982 -	990	0	---	R.LGGFQVCHK.L
1250.6191	1249.6118	1249.5840	22.3	3234 -	3243	0	---	R.TFHSTNTTWR.L
1250.6191	1249.6119	1249.7044	-74.03	184 -	193	1	---	R.RSHVLQHFVK.Q
1300.7900	1299.7827	1299.6922	69.7	1043 -	1054	0	---	K.IDPTPTELSSLK.K
1302.6307	1301.6234	1301.6993	-58.27	3284 -	3294	0	---	R.VNHVNLPPWAR.N
1316.8218	1315.8145	1315.7724	32.0	2606 -	2616	1	---	R.LQRLTVFAVNR.I
1334.8221	1333.8148	1333.6911	92.7	1351 -	1361	0	---	R.TCSEELTLLLR.I
1334.8221	1333.8149	1333.6911	92.8	1351 -	1361	0	---	R.TCSEELTLLLR.I
1448.8531	1447.8458	1447.7168	89.1	2827 -	2838	0	---	K.TVNNNQQSLFQR.L
1518.6587	1517.6514	1517.6966	-29.79	2575 -	2587	0	---	R.SVASDELHVMMQR.R + Oxidation (M)
1523.7185	1522.7112	1522.8429	-86.45	2560 -	2572	1	---	R.KFLLAQTDSLLMK.M + Oxidation (M)
1559.7947	1558.7874	1558.8541	-42.79	1617 -	1629	1	---	R.KPDVTLDFMLPRK.T
1707.8094	1706.8021	1706.7278	43.5	1011 -	1024	1	---	K.EGDRTMNENQDLNR.I + Oxidation (M)
1732.0206	1731.0133	1730.9607	30.4	3417 -	3431	1	---	K.EITYPSPLSWIKGLK.W
1900.9595	1899.9522	1900.0088	-29.78	2983 -	3000	1	---	R.CISVAPSRETAGELLLGK.C
1900.9595	1899.9522	1900.0088	-29.76	2983 -	3000	1	---	R.CISVAPSRETAGELLLGK.C
2193.0256	2192.0183	2192.0406	-10.14	2767 -	2785	0	---	K.DELTEEELDTAELLMNALK.L + Oxidation (M)
2211.0200	2210.0127	2210.0098	1.33	2364 -	2380	1	---	K.YMCTLKFECIFDLEDVK.N
2230.1453	2229.1380	2229.1059	14.4	1015 -	1033	1	---	R.TMNENQDLNRISQPEITVK.E
2233.0505	2232.0432	2232.0595	-7.30	2370 -	2387	1	---	K.FECIFDLEDVKNMGLFQK.W
2927.5508	2926.5435	2926.4600	28.5	2944 -	2969	1	---	K.SEDVVKPPLSYLFEDKTHSSFSSTVK.D

No match to: 848.4879, 850.0154, 850.4973, 861.0195, 864.5118, 865.9869, 868.5078, 870.5034, 876.9944, 881.9677, 882.5228, 882.5228, 892.9686, 951.5814, 953.4899, 996.5488, 1033.6514, 1051.6565, 1051.6565, 1131.6523, 1136.6420, 1147.6807, 1151.6740, 1165.6849, 1165.6849, 1287.6302, 1356.6021, 1421.6768, 1421.6768, 1454.7075, 1475.6875, 1479.7271, 1491.6775, 1552.7145, 1567.6738, 1617.9915, 1617.9915, 1991.8297, 2001.9189, 2012.9465, 2012.9465, 2015.1936, 2044.9634, 2073.0088, 2239.0674, 2283.0967, 2284.0908, 2305.0867, 3337.7156

21. A0A4W2E9U7 **Mass:** 76824 **Score:** 34 **Expect:** 52 **Matches:** 12

Nibrin OS=Bos indicus x Bos taurus OX=30522 GN=NBN PE=4 SV=1

Observed	Mr(expt)	Mr(calc)	ppm	Start	End	Miss	Ions	Peptide
850.4973	849.4900	849.4232	78.6	547 -	553	0	---	K.ESLWSTK.E
951.5814	950.5741	950.5073	70.3	457 -	464	0	---	K.SLFTENLK.S
1131.6523	1130.6450	1130.6118	29.4	1 -	10	0	---	-.MQNGLSQILK.S
1147.6807	1146.6734	1146.6067	58.2	1 -	10	0	---	-.MQNGLSQILK.S + Oxidation (M)
1316.8218	1315.8145	1315.6925	92.8	15 -	25	1	---	R.VTFGVFESKFR.V
1421.6768	1420.6695	1420.7344	-45.70	311 -	322	1	---	K.CRILSQETSTVK.K
1421.6768	1420.6695	1420.7344	-45.67	311 -	322	1	---	K.CRILSQETSTVK.K
1479.7271	1478.7198	1478.7518	-21.60	374 -	385	1	---	R.DYFQPLTSPQKR.E
1991.8297	1990.8224	1990.8612	-19.46	554 -	570	0	---	K.ELSNNCELQDGDEMLPK.K
2012.9465	2011.9392	2011.9231	8.02	26 -	43	0	---	R.VEYEPLVACSSCLDVSGK.T
2012.9465	2011.9393	2011.9231	8.04	26 -	43	0	---	R.VEYEPLVACSSCLDVSGK.T
2211.0200	2210.0127	2210.0559	-19.53	402 -	420	0	---	R.METSCSLLEQTQPTTSLIR.K + Oxidation (M)

No match to: 842.4690, 848.4879, 850.0154, 853.4743, 853.4743, 861.0195, 864.5118, 865.9869, 868.5078, 870.5034, 876.9944, 881.9677, 882.5228, 882.5228, 892.9686, 953.4899, 996.5488, 1017.6209, 1017.6209, 1019.6330, 1033.6514, 1045.5168, 1051.6565, 1051.6565, 1136.6420, 1151.6740, 1165.6849, 1165.6849, 1250.6191, 1250.6191, 1287.6302, 1300.7900, 1302.6307, 1334.8221, 1334.8221, 1356.6021, 1448.8531, 1454.7075, 1475.6875, 1491.6775, 1518.6587, 1523.7185, 1552.7145, 1559.7947, 1567.6738, 1617.9915, 1617.9915, 1707.8094, 1732.0206, 1900.9595, 1900.9595, 2001.9189, 2015.1936, 2044.9634, 2073.0088, 2193.0256, 2230.1453, 2233.0505, 2239.0674, 2283.0967, 2284.0908, 2305.0867, 2927.5508, 3337.7156

22. E1BNC9 **Mass:** 124764 **Score:** 32 **Expect:** 77 **Matches:** 15

Ankyrin 3 OS=Bos taurus OX=9913 GN=ANK3 PE=4 SV=3

Observed	Mr(expt)	Mr(calc)	ppm	Start	End	Miss	Ions	Peptide
842.4690	841.4617	841.4004	72.9	1096 -	1102	1	---	K.GEAYKMK.T + Oxidation (M)
953.4899	952.4826	952.4410	43.7	156 -	163	1	---	R.GSRHHGMR.I + Oxidation (M)
996.5488	995.5415	995.5651	-23.71	861 -	869	0	---	K.DVKPAEPLK.Q
1302.6307	1301.6234	1301.6510	-21.19	1069 -	1080	1	---	K.MSRTSPADGKPR.L
1316.8218	1315.8145	1315.7136	76.7	354 -	365	0	---	K.ATFSPIVTVEPR.R
1334.8221	1333.8148	1333.7089	79.4	692 -	704	0	---	K.NATTDALTSVLTK.I
1334.8221	1333.8149	1333.7089	79.4	692 -	704	0	---	K.NATTDALTSVLTK.I
1356.6021	1355.5948	1355.6140	-14.13	428 -	439	0	---	K.DCVSFTTNVSAR.F
1491.6775	1490.6702	1490.7360	-44.14	90 -	102	0	---	R.DSMMIEELLVPSK.E
1518.6587	1517.6514	1517.7667	-75.96	536 -	548	0	---	K.GGQQLVFNFYAFK.E
1523.7185	1522.7112	1522.7259	-9.61	90 -	102	0	---	R.DSMMIEELLVPSK.E + 2 Oxidation (M)
1559.7947	1558.7874	1558.8005	-8.37	2 -	15	1	---	M.TGGAVFSPDRHIFR.K
2001.9189	2000.9116	2000.8608	25.4	617 -	633	0	---	R.YSYLTEPGMSPQSPCER.T
2012.9465	2011.9392	2011.9157	11.7	966 -	982	1	---	R.LDDSPDQCRDSITSYLK.G
2012.9465	2011.9393	2011.9157	11.7	966 -	982	1	---	R.LDDSPDQCRDSITSYLK.G

No match to: 848.4879, 850.0154, 850.4973, 853.4743, 853.4743, 861.0195, 864.5118, 865.9869, 868.5078, 870.5034, 876.9944, 881.9677, 882.5228, 882.5228, 892.9686, 951.5814, 1017.6209, 1017.6209, 1019.6330, 1033.6514, 1045.5168, 1051.6565, 1051.6565, 1131.6523, 1136.6420, 1147.6807, 1151.6740, 1165.6849, 1165.6849, 1250.6191, 1250.6191, 1287.6302, 1300.7900, 1421.6768, 1421.6768, 1448.8531, 1454.7075, 1475.6875, 1479.7271, 1552.7145, 1567.6738, 1617.9915, 1617.9915, 1707.8094, 1732.0206, 1900.9595, 1900.9595, 1991.8297, 2015.1936, 2044.9634, 2073.0088, 2193.0256, 2211.0200, 2230.1453, 2233.0505, 2239.0674, 2283.0967, 2284.0908, 2305.0867, 2927.5508, 3337.7156

23. G8JKV3 **Mass:** 11943 **Score:** 32 **Expect:** 91 **Matches:** 4

Glutaredoxin domain-containing protein OS=Bos taurus OX=9913 PE=4 SV=1

Observed	Mr(expt)	Mr(calc)	ppm	Start	End	Miss	Ions	Peptide
864.5118	863.5045	863.4501	63.0	2 -	9	0	---	M.AQAFVNSK.I
870.5034	869.4961	869.5334	-42.91	99 -	106	1	---	R.LKQIGALQ.-
1518.6587	1517.6514	1517.8024	-99.48	1 -	14	1	---	-.MAQAFVNSKIQPGK.V
2305.0867	2304.0794	2304.0627	7.25	73 -	92	0	---	R.VFIGQECIGGCTDLVNTHER.G

No match to: 842.4690, 848.4879, 850.0154, 850.4973, 853.4743, 853.4743, 861.0195, 865.9869, 868.5078, 876.9944, 881.9677, 882.5228, 882.5228, 892.9686, 951.5814, 953.4899, 996.5488, 1017.6209, 1017.6209, 1019.6330, 1033.6514, 1045.5168, 1051.6565, 1051.6565, 1131.6523, 1136.6420, 1147.6807, 1151.6740, 1165.6849, 1165.6849, 1250.6191, 1250.6191, 1287.6302, 1300.7900, 1302.6307, 1316.8218, 1334.8221, 1334.8221, 1356.6021, 1421.6768, 1421.6768, 1448.8531, 1454.7075, 1475.6875, 1479.7271, 1491.6775, 1523.7185, 1552.7145, 1559.7947, 1567.6738, 1617.9915, 1617.9915, 1707.8094, 1732.0206, 1900.9595,

PDF-XChange Click to buy NOW! www.docu-track.com

1900.9595, 1991.8297, 2001.9189, 2012.9465, 2012.9465, 2015.1936, 2044.9634, 2073.0088, 2193.0256, 2211.0200, 2230.1453, 2233.0505, 2239.0674, 2283.0967, 2284.0908, 2927.5508, 3337.7156

24. A0A4W2C8T2 **Mass:** 11943 **Score:** 32 **Expect:** 91 **Matches:** 4
Glutaredoxin domain-containing protein OS=Bos indicus x Bos taurus OX=30522 PE=4 SV=1

Observed	Mr(expt)	Mr(calc)	ppm	Start	End	Miss	Ions	Peptide
864.5118	863.5045	863.4501	63.0	2 -	9	0	---	M.AQAFVNSK.I
870.5034	869.4961	869.5334	-42.91	99 -	106	1	---	R.LKQIGALQ.-
1518.6587	1517.6514	1517.8024	-99.48	1 -	14	1	---	-.MAQAFVNSKIQPGK.V
2305.0867	2304.0794	2304.0627	7.25	73 -	92	0	---	R.VFIGQECIGGCTDLVNTHER.G

No match to: 842.4690, 848.4879, 850.0154, 850.4973, 853.4743, 853.4743, 861.0195, 865.9869, 868.5078, 876.9944, 881.9677, 882.5228, 882.5228, 892.9686, 951.5814, 953.4899, 996.5488, 1017.6209, 1017.6209, 1019.6330, 1033.6514, 1045.5168, 1051.6565, 1051.6565, 1131.6523, 1136.6420, 1147.6807, 1151.6740, 1165.6849, 1165.6849, 1250.6191, 1250.6191, 1287.6302, 1300.7900, 1302.6307, 1316.8218, 1334.8221, 1334.8221, 1356.6021, 1421.6768, 1421.6768, 1448.8531, 1454.7075, 1475.6875, 1479.7271, 1491.6775, 1523.7185, 1552.7145, 1559.7947, 1567.6738, 1617.9915, 1617.9915, 1707.8094, 1732.0206, 1900.9595, 1900.9595, 1991.8297, 2001.9189, 2012.9465, 2012.9465, 2015.1936, 2044.9634, 2073.0088, 2193.0256, 2211.0200, 2230.1453, 2233.0505, 2239.0674, 2283.0967, 2284.0908, 2927.5508, 3337.7156

25. A0A3Q1MUB2 **Mass:** 54957 **Score:** 31 **Expect:** 1.1e+002 **Matches:** 10
Glucosaminyl (N-acetyl) transferase 4 OS=Bos taurus OX=9913 GN=GCNT4 PE=4 SV=1

Observed	Mr(expt)	Mr(calc)	ppm	Start	End	Miss	Ions	Peptide
996.5488	995.5415	995.5651	-23.72	359 -	368	0	---	R.VPGIPGEISK.T
1033.6514	1032.6441	1032.5968	45.9	448 -	456	1	---	R.KWITLSSAK.L
1131.6523	1130.6450	1130.5760	61.0	328 -	336	0	---	K.YIFNNSFVK.D
1165.6849	1164.6776	1164.6026	64.4	249 -	258	0	---	K.SNFELVSELK.K
1165.6849	1164.6777	1164.6026	64.4	249 -	258	0	---	K.SNFELVSELK.K
1316.8218	1315.8145	1315.6884	95.8	206 -	216	0	---	K.LETVQYAHISR.L
1448.8531	1447.8458	1447.7267	82.3	369 -	381	1	---	K.TAQDVSDLQSKTR.L
1732.0206	1731.0133	1730.8873	72.8	260 -	275	0	---	K.LNGSNMLETVKPPSTK.T + Oxidation (M)
2012.9465	2011.9392	2011.9832	-21.87	328 -	343	1	---	K.YIFNNSFVKDFFAWSK.D
2012.9465	2011.9393	2011.9832	-21.86	328 -	343	1	---	K.YIFNNSFVKDFFAWSK.D

No match to: 842.4690, 848.4879, 850.0154, 850.4973, 853.4743, 853.4743, 861.0195, 864.5118, 865.9869, 868.5078, 870.5034, 876.9944, 881.9677, 882.5228, 882.5228, 892.9686, 951.5814, 953.4899, 1017.6209, 1017.6209, 1019.6330, 1045.5168, 1051.6565, 1051.6565, 1136.6420, 1147.6807, 1151.6740, 1250.6191, 1250.6191, 1287.6302, 1300.7900, 1302.6307, 1334.8221, 1334.8221, 1356.6021, 1421.6768, 1421.6768, 1454.7075, 1475.6875, 1479.7271, 1491.6775, 1518.6587, 1523.7185, 1552.7145, 1559.7947, 1567.6738, 1617.9915, 1617.9915, 1707.8094, 1900.9595, 1900.9595, 1991.8297, 2001.9189, 2015.1936, 2044.9634, 2073.0088, 2193.0256, 2211.0200, 2230.1453, 2233.0505, 2239.0674, 2283.0967, 2284.0908, 2305.0867, 2927.5508, 3337.7156

26. A0A3Q1M1F6 **Mass:** 143195 **Score:** 30 **Expect:** 1.3e+002 **Matches:** 15
Myomesin 3 OS=Bos taurus OX=9913 GN=MYOM3 PE=4 SV=1

Observed	Mr(expt)	Mr(calc)	ppm	Start	End	Miss	Ions	Peptide
882.5228	881.5155	881.5083	8.20	998 -	1004	1	---	K.KLSHEIR.N
882.5228	881.5155	881.5083	8.23	998 -	1004	1	---	K.KLSHEIR.N
951.5814	950.5741	950.5338	42.5	198 -	205	1	---	R.LFPAGKYR.I
1045.5168	1044.5095	1044.5451	-34.06	947 -	955	1	---	R.VKVEEEANK.S
1165.6849	1164.6776	1164.5849	79.6	477 -	486	1	---	R.KTEIPFDMGK.S
1165.6849	1164.6777	1164.5849	79.7	477 -	486	1	---	R.KTEIPFDMGK.S
1250.6191	1249.6118	1249.6853	-58.76	328 -	338	1	---	R.QAVLKVACAYK.E
1250.6191	1249.6119	1249.6853	-58.73	328 -	338	1	---	R.QAVLKVACAYK.E
1302.6307	1301.6234	1301.6074	12.3	667 -	676	1	---	R.TGKEYEFCIR.S
1454.7075	1453.7002	1453.7864	-59.25	716 -	727	1	---	K.NEMVIGWKPPKR.R
1479.7271	1478.7198	1478.6606	40.1	37 -	48	1	---	R.QHSLQMDSSMRK.R + 2 Oxidation (M)
1491.6775	1490.6702	1490.7558	-57.40	1120 -	1131	0	---	K.QGPYFVEPLQWK.V
1707.8094	1706.8021	1706.9468	-84.75	598 -	613	0	---	R.GKPATLPPPTQVQAFR.D
2001.9189	2000.9116	2001.0378	-63.08	677 -	696	1	---	R.SVSEAGVGESSAVTEPIRVK.Q
2283.0967	2282.0894	2282.2246	-59.21	264 -	283	1	---	K.RLMFGPSVDFTSVLKPVFAR.E + Oxidation (M)

No match to: 842.4690, 848.4879, 850.0154, 850.4973, 853.4743, 853.4743, 861.0195, 864.5118, 865.9869, 868.5078, 870.5034, 876.9944, 881.9677, 892.9686, 953.4899, 996.5488, 1017.6209, 1017.6209, 1019.6330, 1033.6514, 1051.6565, 1051.6565, 1131.6523, 1136.6420, 1147.6807, 1151.6740, 1287.6302, 1300.7900, 1316.8218, 1334.8221, 1334.8221, 1356.6021, 1421.6768, 1421.6768, 1448.8531, 1475.6875, 1518.6587, 1523.7185, 1552.7145, 1559.7947, 1567.6738, 1617.9915, 1617.9915, 1732.0206, 1900.9595, 1900.9595, 1991.8297, 2012.9465, 2012.9465, 2015.1936, 2044.9634, 2073.0088, 2193.0256, 2211.0200, 2230.1453, 2233.0505, 2239.0674, 2284.0908, 2305.0867, 2927.5508, 3337.7156

27. Q9QAQ5 **Mass:** 12971 **Score:** 30 **Expect:** 1.4e+002 **Matches:** 4
Non-structural protein of 12.7 kDa OS=Bovine coronavirus (strain OK-0514) OX=231432 GN=5a PE=3 SV=1

Observed	Mr(expt)	Mr(calc)	ppm	Start	End	Miss	Ions	Peptide
1147.6807	1146.6734	1146.5743	86.4	1 -	9	0	---	-.MDIWKPETK.Y
1448.8531	1447.8458	1447.7823	43.9	2 -	12	1	---	M.DIWKPETKYLR.Y
1479.7271	1478.7198	1478.8286	-73.54	90 -	101	0	---	K.LVFLAVDFITWR.R
1559.7947	1558.7874	1558.7450	27.2	49 -	62	0	---	R.NQGSFCATLTLYGK.S

No match to: 842.4690, 848.4879, 850.0154, 850.4973, 853.4743, 853.4743, 861.0195, 864.5118, 865.9869, 868.5078, 870.5034, 876.9944, 881.9677, 882.5228, 882.5228, 892.9686, 951.5814, 953.4899, 996.5488, 1017.6209, 1017.6209, 1019.6330, 1033.6514, 1045.5168, 1051.6565, 1051.6565, 1131.6523, 1136.6420, 1151.6740, 1165.6849, 1165.6849, 1250.6191, 1250.6191, 1287.6302, 1300.7900, 1302.6307, 1316.8218, 1334.8221, 1334.8221, 1356.6021, 1421.6768, 1421.6768, 1454.7075, 1475.6875, 1491.6775, 1518.6587, 1523.7185, 1552.7145, 1567.6738, 1617.9915, 1617.9915, 1707.8094, 1732.0206, 1900.9595, 1900.9595, 1991.8297, 2001.9189, 2012.9465, 2012.9465, 2015.1936, 2044.9634, 2073.0088, 2193.0256, 2211.0200, 2230.1453, 2233.0505, 2239.0674, 2283.0967, 2284.0908, 2305.0867, 2927.5508, 3337.7156

28. Q6YPB4 **Mass:** 16612 **Score:** 30 **Expect:** 1.4e+002 **Matches:** 6
NOSIC domain-containing protein (Fragment) OS=Bos taurus OX=9913 PE=2 SV=1

Observed	Mr(expt)	Mr(calc)	ppm	Start	End	Miss	Ions	Peptide
1017.6209	1016.6136	1016.5403	72.1	95 -	102	0	---	R.QSLHTYLR.S
1017.6209	1016.6136	1016.5403	72.1	95 -	102	0	---	R.QSLHTYLR.S
1131.6523	1130.6450	1130.5819	55.9	38 -	46	1	---	K.ELNEEKLEK.L
1136.6420	1135.6347	1135.5431	80.7	47 -	56	0	---	K.LEELTMDAAK.A + Oxidation (M)
1567.6738	1566.6665	1566.7507	-53.73	7 -	18	0	---	R.EWYGYHFPELVK.I
2305.0867	2304.0794	2304.0613	7.85	66 -	86	0	---	R.SSMGMDISAIDLINIESFSSR.V + 2 Oxidation (M)

No match to: 842.4690, 848.4879, 850.0154, 850.4973, 853.4743, 853.4743, 861.0195, 864.5118, 865.9869, 868.5078, 870.5034, 876.9944, 881.9677, 882.5228, 882.5228, 892.9686, 951.5814, 953.4899, 996.5488, 1019.6330, 1033.6514, 1045.5168, 1051.6565, 1051.6565, 1147.6807, 1151.6740, 1165.6849, 1165.6849, 1250.6191, 1250.6191, 1287.6302, 1300.7900, 1302.6307, 1316.8218, 1334.8221, 1334.8221, 1356.6021, 1421.6768, 1421.6768, 1448.8531, 1454.7075, 1475.6875, 1479.7271, 1491.6775, 1518.6587, 1523.7185, 1552.7145, 1559.7947, 1617.9915, 1617.9915, 1707.8094, 1732.0206, 1900.9595, 1900.9595, 1991.8297, 2001.9189, 2012.9465, 2012.9465, 2015.1936, 2044.9634, 2073.0088, 2193.0256, 2211.0200, 2230.1453, 2233.0505, 2239.0674, 2283.0967, 2284.0908, 2927.5508, 3337.7156

PDF-XChange Click to buy NOW! www.docu-track.com
PDF-XChange Click to buy NOW! www.docu-track.com

[illegible]. D8V071 **Mass:** 35943 **Score:** 30 **Expect:** 1.5e+002 **Matches:** 7
Phosphoprotein OS=Tibrogargan virus (strain CS132) OX=1559361 GN=P PE=3 SV=1

Observed	Mr(expt)	Mr(calc)	ppm	Start	End	Miss	Ions	Peptide
951.5814	950.5741	950.4933	85.0	52 -	58	1	---	R.ELNYRTR.A
996.5488	995.5415	995.5222	19.4	295 -	303	1	---	K.ASGIYRAMK.L
1250.6191	1249.6118	1249.6302	-14.74	42 -	51	1	---	K.QDLKEFLDSR.E
1250.6191	1249.6119	1249.6302	-14.71	42 -	51	1	---	K.QDLKEFLDSR.E
1518.6587	1517.6514	1517.7031	-34.08	85 -	97	1	---	K.MEESSLHDDKAIK.Q + Oxidation (M)
1523.7185	1522.7112	1522.7092	1.31	185 -	196	1	---	K.DNQGYYFYPTKK.W
2044.9634	2043.9561	2044.0185	-30.51	111 -	130	1	---	R.SPNANSIGGQSVIKDVSSER.K

No match to: 842.4690, 848.4879, 850.0154, 850.4973, 853.4743, 853.4743, 861.0195, 864.5118, 865.9869, 868.5078, 870.5034, 876.9944, 881.9677, 882.5228, 882.5228, 892.9686, 953.4899, 1017.6209, 1017.6209, 1019.6330, 1033.6514, 1045.5168, 1051.6565, 1051.6565, 1131.6523, 1136.6420, 1147.6807, 1151.6740, 1165.6849, 1165.6849, 1287.6302, 1300.7900, 1302.6307, 1316.8218, 1334.8221, 1334.8221, 1356.6021, 1421.6768, 1421.6768, 1448.8531, 1454.7075, 1475.6875, 1479.7271, 1491.6775, 1552.7145, 1559.7947, 1567.6738, 1617.9915, 1617.9915, 1707.8094, 1732.0206, 1900.9595, 1900.9595, 1991.8297, 2001.9189, 2012.9465, 2012.9465, 2015.1936, 2073.0088, 2193.0256, 2211.0200, 2230.1453, 2233.0505, 2239.0674, 2283.0967, 2284.0908, 2305.0867, 2927.5508, 3337.7156

30. A0A4W2DGE8 **Mass:** 25670 **Score:** 29 **Expect:** 1.5e+002 **Matches:** 7
Peptidylprolyl isomerase OS=Bos indicus x Bos taurus OX=30522 GN=FKBP11 PE=4 SV=1

Observed	Mr(expt)	Mr(calc)	ppm	Start	End	Miss	Ions	Peptide
1165.6849	1164.6776	1164.7343	-48.62	2 -	11	0	---	M.TLRPSLLPLR.L
1165.6849	1164.6777	1164.7343	-48.59	2 -	11	0	1	M.TLRPSLLPLR.L
1250.6191	1249.6118	1249.6891	-61.83	172 -	182	0	---	R.LSTHALRPEAR.R
1250.6191	1249.6119	1249.6891	-61.79	172 -	182	0	---	R.LSTHALRPEAR.R
1300.7900	1299.7827	1299.6717	85.4	138 -	148	1	---	R.NLCQGSQRLPK.V
1518.6587	1517.6514	1517.7020	-33.33	214 -	224	1	---	K.VMQYCHPHFRK.T + Oxidation (M)
2044.9634	2043.9561	2044.1205	-80.39	74 -	91	1	---	R.IFDTSLTRDPLVIELGQK.Q

No match to: 842.4690, 848.4879, 850.0154, 850.4973, 853.4743, 853.4743, 861.0195, 864.5118, 865.9869, 868.5078, 870.5034, 876.9944, 881.9677, 882.5228, 882.5228, 892.9686, 951.5814, 953.4899, 996.5488, 1017.6209, 1017.6209, 1019.6330, 1033.6514, 1045.5168, 1051.6565, 1051.6565, 1131.6523, 1136.6420, 1147.6807, 1151.6740, 1287.6302, 1302.6307, 1316.8218, 1334.8221, 1334.8221, 1356.6021, 1421.6768, 1421.6768, 1448.8531, 1454.7075, 1475.6875, 1479.7271, 1491.6775, 1523.7185, 1552.7145, 1559.7947, 1567.6738, 1617.9915, 1617.9915, 1707.8094, 1732.0206, 1900.9595, 1900.9595, 1991.8297, 2001.9189, 2012.9465, 2012.9465, 2015.1936, 2073.0088, 2193.0256, 2211.0200, 2230.1453, 2233.0505, 2239.0674, 2283.0967, 2284.0908, 2305.0867, 2927.5508, 3337.7156

Search Parameters

Type of search : Sequence Query
Enzyme : Trypsin
Fixed modifications : Carbamidomethyl (C)
Variable modifications : Oxidation (M)
Mass values : Monoisotopic
Protein Mass : Unrestricted
Peptide Mass Tolerance : ± 100 ppm
Fragment Mass Tolerance: ± 0.3 Da
Max Missed Cleavages : 1
Instrument type : MALDI-TOF-TOF
Query1 (842.4690,1+): <no title>
Query2 (848.4879,1+): <no title>
Query3 (850.0154,1+): <no title>
Query4 (850.4973,1+): <no title>
Query5 (853.4743,1+): <no title>
Query6 (853.4743,1+): Locus:1..14.0.10
Query7 (861.0195,1+): <no title>
Query8 (864.5118,1+): <no title>
Query9 (865.9869,1+): <no title>
Query10 (868.5078,1+): <no title>
Query11 (870.5034,1+): <no title>
Query12 (876.9944,1+): <no title>
Query13 (881.9677,1+): <no title>
Query14 (882.5228,1+): Locus:1..14.0.7
Query15 (882.5228,1+): <no title>
Query16 (892.9686,1+): <no title>
Query17 (951.5814,1+): <no title>
Query18 (953.4899,1+): <no title>
Query19 (996.5488,1+): <no title>
Query20 (1017.6209,1+): <no title>
Query21 (1017.6209,1+): Locus:1..14.0.9
Query22 (1019.6330,1+): <no title>
Query23 (1033.6514,1+): <no title>
Query24 (1045.5168,1+): <no title>
Query25 (1051.6565,1+): Locus:1..14.0.1
Query26 (1051.6565,1+): <no title>
Query27 (1131.6523,1+): <no title>
Query28 (1136.6420,1+): <no title>
Query29 (1147.6807,1+): <no title>
Query30 (1151.6740,1+): <no title>
Query31 (1165.6849,1+): <no title>
Query32 (1165.6849,1+): Locus:1..14.0.8
Query33 (1250.6191,1+): <no title>
Query34 (1250.6191,1+): Locus:1..14.0.11
Query35 (1287.6302,1+): <no title>
Query36 (1300.7900,1+): <no title>
Query37 (1302.6307,1+): <no title>
Query38 (1316.8218,1+): <no title>
Query39 (1334.8221,1+): <no title>
Query40 (1334.8221,1+): Locus:1..14.0.2
Query41 (1356.6021,1+): <no title>
Query42 (1421.6768,1+): Locus:1..14.0.3
Query43 (1421.6768,1+): <no title>
Query44 (1448.8531,1+): <no title>
Query45 (1454.7075,1+): <no title>
Query46 (1475.6875,1+): <no title>
Query47 (1479.7271,1+): <no title>
Query48 (1491.6775,1+): <no title>
Query49 (1518.6587,1+): <no title>
Query50 (1523.7185,1+): <no title>
Query51 (1552.7145,1+): <no title>
Query52 (1559.7947,1+): <no title>

Query53 (1567.6738,1+): <no title>
Query54 (1617.9915,1+): Locus:1..14.0.6
Query55 (1617.9915,1+): <no title>
Query56 (1707.8094,1+): <no title>
Query57 (1732.0206,1+): <no title>
Query58 (1900.9595,1+): Locus:1..14.0.4
Query59 (1900.9595,1+): <no title>
Query60 (1991.8297,1+): <no title>
Query61 (2001.9189,1+): <no title>
Query62 (2012.9465,1+): <no title>
Query63 (2012.9465,1+): Locus:1..14.0.5
Query64 (2015.1936,1+): <no title>
Query65 (2044.9634,1+): <no title>
Query66 (2073.0088,1+): <no title>
Query67 (2193.0256,1+): <no title>
Query68 (2211.0200,1+): <no title>
Query69 (2230.1453,1+): <no title>
Query70 (2233.0505,1+): <no title>
Query71 (2239.0674,1+): <no title>
Query72 (2283.0967,1+): <no title>
Query73 (2284.0908,1+): <no title>
Query74 (2305.0867,1+): <no title>
Query75 (2927.5508,1+): <no title>
Query76 (3337.7156,1+): <no title>

Mascot: http://www.matrixscience.com/
