# Supplementary material for: Alpha-2-Macroglobulin as a New Promising Biomarker Improving the Diagnostic Sensitivity of Bovine Paratuberculosis
Source: Front Vet Sci. 2021 Mar 5;8:637716. doi: 10.3389/fvets.2021.637716 (PMC7973028; doi:10.3389/fvets.2021.637716)
Supplement: Supplementary Data 1 — Mascot search results and raw data files for LC-MS/MS analysis for the identification of specific blood proteins in cattle with Johne's disease. [file Data_Sheet_1.zip › mascot/4 Alpha-2-macroglobulin_2.pdf]

PDF-XChange
Click to buy NOW!
www.docu-track.com

PDF-XChange
Click to buy NOW!
www.docu-track.com

MATRIX SCIENCE MASCOT Search Results

Protein View: R9QSM8

Alpha-2-macroglobulin OS=Bos taurus OX=9913 GN=A2M PE=2 SV=1

Database:	Uniprot_bovine
Score:	90
Expect:	0.00014
Nominal mass (M_r):	134613
Calculated pI:	5.75

Sequence similarity is available as an NCBI BLAST search of R9QSM8 against nr.

Search parameters

Enzyme:	Trypsin: cuts C-term side of KR unless next residue is P.
Fixed modifications:	Carbamidomethyl (C)
Variable modifications:	Oxidation (M)

Protein sequence coverage: 19%

Matched peptides shown in ***bold red***.

1 MGKNKLLYPS LTLLLLLLLP TDASVSGKPQ YMVLVPSLLH TETPEKGCLL

51 LSHLNETVTV SASLESVREN RSLFTDVVAE K**DLFHCVSFT** **LPRSPTSQEV**

101 **MFLTIQVK**GP TQEFKKRTTV LVKNEESLVF VQTDKPIYKP EQTVKFRIVL

151 LDESFHPLNE LVPLVYVEDP KGNRIAQWQN LEVENGLQQL TFPLSSEPFQ

201 GSYKVVVQK**G** **SGGTAEHPFT** **VEEFVLPK**FE VQVRMPKIIT ILEEEVQVSV

251 CGLYTYGKPV PGRVTMNMCR KYRNPSNCYG EESNAVCEKF SGELNNEGCF

301 SQQVNTKIFQ LKRQEFEMKI EVEAKIQEEG TEVELTGKGA TEITTTITK**L**

351 **SFVTVDSNLR** RGIPFTGKVL LVDGKGVPMP NKVIFITANE ANHNSNTTTD

401 EHGLAQFSIT TTKIKGTSLS IRVKYKDHSP CYGYQWLSEE HQDAYHSANL

451 VFSR**SNSFVY** **LEPLPR**ELPC GKTQTVQAHY VLKGQVLKDL KELVFYYLIM

501 AKGGIVRSGT HTLPVEQGDM QGHFSMSVPV ESDIAPVAR**L** **LIYAILPDGE**

551 **VVGDSAR**YEI EHCLANK**VGL** **NFSPGQSFPA** **SQAHLRVTAS** **PQSLCALRAV**

601 **DQSVLLMRPE** **AELSAATVYN** **LLPVK**DLSSF PSSVNQQEED NEDCISHDNV

651 YINGIMYFPV SNTNEK**DMYS** **FLQDMGLK**AF TNSKIHKPKI CPQPEEHRIQ

701 HHTLLASPVR AEMGRNR**DFV** **HFDDTSEPPT** **ETVR**KYFPET WIWDLVVVSS

751 SGVHEVEVTV PDTITEWKAG ALCLSR**DTGL** **GLSPTASLRV** **FQPFFVELTM**

801 **PYSVIR**GEAF TLKATVLNYL PKCIRVSVQL EASPAFLAVP EKEQETYCIC

851 GNGRQTVSWA VTPKSLGNVN FTVSAEAVES QELCGSEVPV VPEHGRKDTI

901 IKPLLVEPEG LEKEVIFNSL LCPSVDFVFL GAEDGGQVLR HFPPAAATDT

951 AADAHDPARP GAKVSESLSL KLPPNVVEES AR**ASFSVLGD** **ILGSAMR**NTQ

1001 NLLQMPYGCG EQNMARFAPN IYVLDYLNET QQLTAELKSK AILYLNTGYQ

1051 RQLLYKHFDG SYSTFGEHRG NSEGNTWLTA FVLKSFAQAR GYIFIDEAHI

1101 TEALTWLAQK **QKSNGCFRST** **GTLLNNAIK**G GVDDEVTLSA YITIALLEMP

1151 LPVTHPVVRN ALFCLDSAWK CLKTMSMKTA LLDSLFHRLR SPWKKDNF

PDF-XChange Click to buy NOW! www.docu-track.com

PDF-XChange Click to buy NOW! www.docu-track.com

Unformatted sequence string: 1198 residues (for pasting into other applications).

Sort peptides by ◉ Residue Number ○ Increasing Mass ○ Decreasing Mass

Show predicted peptides also

Query	Start – End	Observed	Mr(expt)	Mr(calc)	ppm	M	Score	Peptic
48	82 – 93	1491.6775	1490.6702	1490.7340	-42.8	0		K.DLFH
56	94 – 108	1707.8094	1706.8021	1706.8913	-52.3	0		R.SPTS
61	210 – 228	2001.9189	2000.9116	2000.9844	-36.4	0		K.GSGG
33	350 – 360	1250.6191	1249.6118	1249.6667	-43.9	0		K.LSFV
34	350 – 360	1250.6191	1249.6119	1249.6667	-43.8	0		K.LSFV
42	455 – 466	1421.6768	1420.6695	1420.7350	-46.1	0	14	R.SNSF
43	455 – 466	1421.6768	1420.6695	1420.7350	-46.1	0		R.SNSF
58	540 – 557	1900.9595	1899.9522	1900.0306	-41.2	0	13	R.LLIY
59	540 – 557	1900.9595	1899.9522	1900.0306	-41.2	0		R.LLIY
62	568 – 586	2012.9465	2011.9392	2012.0228	-41.5	0		K.VGLN
63	568 – 586	2012.9465	2011.9393	2012.0228	-41.5	0		K.VGLN
37	587 – 598	1302.6307	1301.6234	1301.6762	-40.5	0		R.VTAS
75	599 – 625	2927.5508	2926.5435	2926.5837	-13.7	0		R.AVDQ
47	667 – 678	1479.7271	1478.7198	1478.6422	52.5	0		K.DMYS + 2 Oxidat (M)
60	718 – 734	1991.8297	1990.8224	1990.8909	-34.4	0		R.DFVH
35	777 – 789	1287.6302	1286.6229	1286.6830	-46.7	0		R.DTGL
66	790 – 806	2073.0088	2072.0015	2072.0805	-38.1	0		R.VFQF
50	983 – 997	1523.7185	1522.7112	1522.7814	-46.1	0		R.ASFS
19	1111 – 1118	996.5488	995.5415	995.4607	81.2	1		K.QKSN
27	1119 – 1129	1131.6523	1130.6450	1130.6295	13.7	0		R.STGT

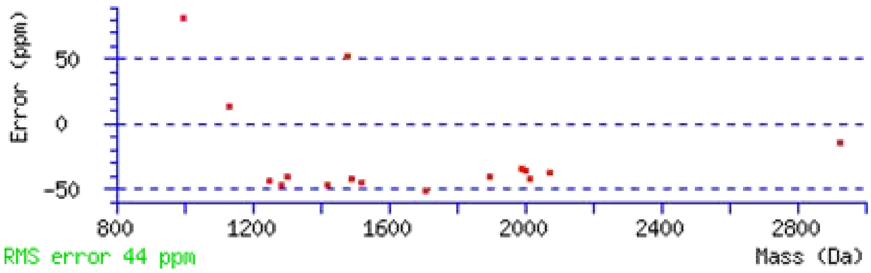


```
ID   R9QSM8_BOVIN            Unreviewed;      1198 AA.
AC   R9QSM8;
DT   18-SEP-2013, integrated into UniProtKB/TrEMBL.
DT   18-SEP-2013, sequence version 1.
DT   11-DEC-2019, entry version 23.
DE   SubName: Full=Alpha-2-macroglobulin {ECO:0000313|EMBL:AGC26508.1};
GN   Name=A2M {ECO:0000313|EMBL:AGC26508.1};
OS   Bos taurus (Bovine).
OC   Eukaryota; Metazoa; Chordata; Craniata; Vertebrata; Euteleostomi; Mammalia;
OC   Eutheria; Laurasiatheria; Artiodactyla; Ruminantia; Pecora; Bovidae;
OC   Bovinae; Bos.
OX   NCBI_TaxID=9913 {ECO:0000313|EMBL:AGC26508.1};
RN   [1] {ECO:0000313|EMBL:AGC26508.1}
RP   NUCLEOTIDE SEQUENCE.
RA   Wang X.;
RT   "Identification and characterization of novel splice variants and
RT   expression analysis of the A2M gene in healthy and mastitis-infected
RT   mammary tissues of Chinese Holstein dairy cow.";
```

PDF-XChange
Click to buy NOW!
www.docu-track.com

```
RL   Submitted (APR-2012) to the EMBL/GenBank/DDBJ databases.
CC   -----------------------------------------------------------------------
CC   Copyrighted by the UniProt Consortium, see https://www.uniprot.org/terms
CC   Distributed under the Creative Commons Attribution (CC BY 4.0) License
CC   -----------------------------------------------------------------------
DR   EMBL; JQ918150; AGC26508.1; -; mRNA.
DR   eggNOG; ENOG410KCRI; Eukaryota.
DR   eggNOG; ENOG410XQIV; LUCA.
DR   OrthoDB; 354230at2759; -.
DR   GO; GO:0005615; C:extracellular space; IEA:InterPro.
DR   GO; GO:0004866; F:endopeptidase inhibitor activity; IEA:InterPro.
DR   Gene3D; 2.60.40.10; -; 2.
DR   InterPro; IPR011625; A2M_N_BRD.
DR   InterPro; IPR011626; Alpha-macroglobulin_TED.
DR   InterPro; IPR013783; Ig-like_fold.
DR   InterPro; IPR014756; Ig_E-set.
DR   InterPro; IPR001599; Macroglobln_a2.
DR   InterPro; IPR019742; MacroglobnA2_CS.
DR   InterPro; IPR002890; MG2.
DR   InterPro; IPR041555; MG3.
DR   InterPro; IPR040839; MG4.
DR   InterPro; IPR008930; Terpenoid_cyclase/PrenylTrfase.
DR   InterPro; IPR010916; TonB_box_CS.
DR   Pfam; PF00207; A2M; 1.
DR   Pfam; PF07703; A2M_BRD; 1.
DR   Pfam; PF01835; MG2; 1.
DR   Pfam; PF17791; MG3; 1.
DR   Pfam; PF17789; MG4; 1.
DR   Pfam; PF07678; TED_complement; 1.
DR   SMART; SM01360; A2M; 1.
DR   SMART; SM01359; A2M_N_2; 1.
DR   SUPFAM; SSF48239; SSF48239; 1.
DR   SUPFAM; SSF81296; SSF81296; 1.
DR   PROSITE; PS00477; ALPHA_2_MACROGLOBULIN; 1.
DR   PROSITE; PS00430; TONB_DEPENDENT_REC_1; 1.
PE   2: Evidence at transcript level;
KW   Signal {ECO:0000256|SAM:SignalP}.
FT   SIGNAL          1..23
FT                   /evidence="ECO:0000256|SAM:SignalP"
FT   CHAIN           24..1198
FT                   /evidence="ECO:0000256|SAM:SignalP"
FT                   /id="PRO_5004488606"
FT   DOMAIN          459..607
FT                   /note="A2M_N_2"
FT                   /evidence="ECO:0000259|SMART:SM01359"
FT   DOMAIN          740..830
FT                   /note="A2M"
FT                   /evidence="ECO:0000259|SMART:SM01360"
SQ   SEQUENCE   1198 AA;  133386 MW;  9871AD7632A2EDBE CRC64;
     MGKNKLLYPS LTLLLLLLLP TDASVSGKPQ YMVLVPSLLH TETPEKGCLL LSHLNETVTV
     SASLESVREN RSLFTDVVAE KDLFHCVSFT LPRSPTSQEV MFLTIQVKGP TQEFKKRTTV
     LVKNEESLVF VQTDKPIYKP EQTVKFRIVL LDESFHPLNE LVPLVYVEDP KGNRIAQWQN
     LEVENGLQQL TFPLSSEPFQ GSYKVVVQKG SGGTAEHPFT VEEFVLPKFE VQVRMPKIIT
     ILEEEVQVSV CGLYTYGKPV PGRVTMNMCR KYRNPSNCYG EESNAVCEKF SGELNNEGCF
     SQQVNTKIFQ LKRQEFEMKI EVEAKIQEEG TEVELTGKGA TEITTTITKL SFVTVDSNLR
     RGIPFTGKVL LVDGKGVPMP NKVIFITANE ANHNSNTTTD EHGLAQFSIT TTKIKGTSLS
     IRVKYKDHSP CYGYQWLSEE HQDAYHSANL VFSRSNSFVY LEPLPRELPC GKTQTVQAHY
     VLKGQVLKDL KELVFYYLIM AKGGIVRSGT HTLPVEQGDM QGHFSMSVPV ESDIAPVARL
     LIYAILPDGE VVGDSARYEI EHCLANKVGL NFSPGQSFPA SQAHLRVTAS PQSLCALRAV
     DQSVLLMRPE AELSAATVYN LLPVKDLSSF PSSVNQQEED NEDCISHDNV YINGIMYFPV
     SNTNEKDMYS FLQDMGLKAF TNSKIHKPKI CPQPEEHRIQ HHTLLASPVR AEMGRNRDFV
     HFDDTSEPPT ETVRKYFPET WIWDLVVVSS SGVHEVEVTV PDTITEWKAG ALCLSRDTGL
     GLSPTASLRV FQPFFVELTM PYSVIRGEAF TLKATVLNYL PKCIRVSVQL EASPAFLAVP
     EKEQETYCIC GNGRQTVSWA VTPKSLGNVN FTVSAEAVES QELCGSEVPV VPEHGRKDTI
     IKPLLVEPEG LEKEVIFNSL LCPSVDFVFL GAEDGGQVLR HFPPAAATDT AADAHDPARP
```

PDF-XChange Click to buy NOW! www.docu-track.com

```
     GAKVSESLSL KLPPNVVEES ARASFSVLGD ILGSAMRNTQ NLLQMPYGCG EQNMARFAPN
     IYVLDYLNET QQLTAELKSK AILYLNTGYQ RQLLYKHFDG SYSTFGEHRG NSEGNTWLTA
     FVLKSFAQAR GYIFIDEAHI TEALTWLAQK QKSNGCFRST GTLLNNAIKG GVDDEVTLSA
     YITIALLEMP LPVTHPVVRN ALFCLDSAWK CLKTMSMKTA LLDSLFHRLR SPWKKDNF
//
```

Mascot: http://www.matrixscience.com/
