# Supplementary material for: Alpha-2-Macroglobulin as a New Promising Biomarker Improving the Diagnostic Sensitivity of Bovine Paratuberculosis
Source: Front Vet Sci. 2021 Mar 5;8:637716. doi: 10.3389/fvets.2021.637716 (PMC7973028; doi:10.3389/fvets.2021.637716)
Supplement: Supplementary Data 1 — Mascot search results and raw data files for LC-MS/MS analysis for the identification of specific blood proteins in cattle with Johne's disease. [file Data_Sheet_1.zip › mascot/5 IgM heavy chain constant region_1.pdf]

PDF-XChange Click to buy NOW! www.docu-track.com

MATRIX SCIENCE Mascot Search Results

```
User          :
Email         :
Search title  : BAEDW\2019_10_16\2019_10_16\MSMS 5\D1
Database      : NCBInr 20131104 (33815671 sequences; 11795550776 residues)
Timestamp     : 15 Oct 2019 at 10:55:09 GMT
Warning       : A Peptide summary report will usually give a much clearer picture of MS/MS search results.
Top Score     : 50 for gi|2232299, IgM heavy chain constant region [Bos taurus]
```

Mascot Score Histogram

Protein score is -10*Log(P), where P is the probability that the observed match is a random event.
Protein scores greater than 88 are significant (p<0.05).
Protein scores are derived from ions scores as a non-probabilistic basis for ranking protein hits.

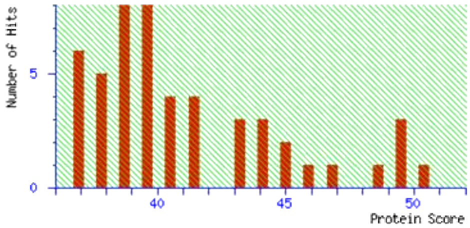

Protein Summary Report

Format As | Protein Summary (deprecated) | Help

Significance threshold p< 0.05 Max. number of hits 50

Re-Search All | Search Unmatched

Index

	Accession	Mass	Score	Description
1.	gi|2232299	48512	50	IgM heavy chain constant region [Bos taurus]
2.	gi|33413902	49848	50	immunoglobulin mu heavy chain constant region [Bos taurus]
3.	gi|206597414	28268	50	Major histocompatibility complex class II beta chain BMA2, (similar to HLA class II, D beta chain) pre
4.	gi|34538498	50536	50	immunoglobulin heavy chain constant region [Bos taurus]
5.	gi|518349625	5336	48	hypothetical protein [Moraxella boevrei]
6.	gi|494742220	17861	47	3-hydroxyacyl-ACP dehydratase [Bartonella melophagi]
7.	gi|518846823	5061	46	hypothetical protein [Mycoplasma hominis]
8.	gi|488818860	9345	45	hypothetical protein [Phaeospirillum molischianum]
9.	gi|446609084	31602	45	prephenate dehydratase [Streptococcus oralis]
10.	gi|518026209	30111	44	serine acetyltransferase [Afipia birgiae]
11.	gi|24496448	66533	44	immunoglobulin M heavy chain secretory form [Bos taurus]
12.	gi|547755423	19340	44	ribosome maturation factor RimM [Clostridium sp. CAG:221]
13.	gi|492049521	6980	43	hypothetical protein [Pseudomonas amygdali]
14.	gi|488127006	5046	43	hypothetical protein, partial [Helicobacter pylori]
15.	gi|68535459	8368	43	hypothetical protein jk0392 [Corynebacterium jeikeium K411]
16.	gi|3834667	48810	42	immunoglobulin heavy chain constant region [Bos taurus]
17.	gi|547238705	13800	42	ribosomal protein S13 [Firmicutes bacterium CAG:884]
18.	gi|345564418	28894	41	hypothetical protein AOL_s00083g474 [Arthrobotrys oligospora ATCC 24927]
19.	gi|517947175	20170	41	hypothetical protein [Anaerococcus sp. PH9]
20.	gi|522808524	25797	41	hypothetical protein [Mycobacterium marinum]
21.	gi|550715204	37257	41	transcriptional activator FtrA [Enterobacter sp. MGH 8]
22.	gi|516231730	9834	41	hypothetical protein [Vibrio sp. 712i1]
23.	gi|517496258	32907	40	hypothetical protein [Bacteroides gallinarum]
24.	gi|183984872	25857	40	hypothetical protein MMAR_4904 [Mycobacterium marinum M]
25.	gi|443492961	25831	40	putative deacetylase [Mycobacterium liflandii 128FXT]
26.	gi|118616343	25912	39	hypothetical protein MUL_0502 [Mycobacterium ulcerans Agy99]
27.	gi|491438800	10485	39	acetyltransferase [Corynebacterium jeikeium]
28.	gi|491891625	32908	39	hypothetical protein [Bacteroides stercoris]
29.	gi|547517138	32936	39	uncharacterized protein [Bacteroides stercoris CAG:120]
30.	gi|517368986	14244	39	30S ribosomal protein S13 [Streptomyces sp. LaPpAH-108]
31.	gi|516399639	5928	39	integrase [Vibrio vulnificus]
32.	gi|479181342	3438	39	Helix-turn-helix domain of resolvase. [Enterococcus sp. 7L76]
33.	gi|491349492	31061	39	hypothetical protein [Gordonia sputi]
34.	gi|493388713	17048	39	MarR family transcriptional regulator [Streptomyces tsukubaensis]
35.	gi|551036884	33460	39	hypothetical protein [Lachnospiraceae bacterium NK4A136]
36.	gi|320333797	39533	39	phospho-2-dehydro-3-deoxyheptonate aldolase [Deinococcus maricopensis DSM 21211]
37.	gi|409039618	29114	38	hypothetical protein PHACADRAFT_201965 [Phanerochaete carnosa HHB-10118-sp]
38.	gi|187251559	20917	38	crossover junction endodeoxyribonuclease RuvC [Elusimicrobium minutum Pei191]
39.	gi|261260695	10351	38	Yclm [Bacillus amyloliquefaciens]
40.	gi|517051098	27685	38	transcriptional regulator [Rhizobium sp. BR816]
41.	gi|444720581	100358	38	General transcription factor II-I [Tupaia chinensis]
42.	gi|71039991	32224	38	conserved ATPase domain protein [Macropis nuda]
43.	gi|490661404	4181	38	hypothetical protein [Burkholderia pseudomallei]
44.	gi|489236824	65949	37	multidrug ABC transporter ATP-binding protein [Gemella haemolysans]
45.	gi|547230974	17853	37	transposase [Bacteroides intestinalis CAG:564]
46.	gi|494229523	4528	37	hypothetical protein [Flavobacterium frigoris]
47.	gi|28493757	41909	37	pyruvate dehydrogenase E1 component alpha subunit [Tropheryma whipplei str. Twist]
48.	gi|517826373	12209	37	hypothetical protein [Hirschia maritima]
49.	gi|446609078	29288	37	prephenate dehydratase, partial [Streptococcus sp. GMD1S]
50.	gi|551565531	42563	37	hypothetical protein EMIHUDRAFT_210302 [Emiliania huxleyi CCMP1516]

Results List

1. gi|2232299 **Mass:** 48512 **Score:** 50 **Expect:** 3.1e+002 **Matches:** 9

IgM heavy chain constant region [Bos taurus]

Observed	Mr(expt)	Mr(calc)	ppm	Start		End	Miss	Ions	Peptide
1036.6012	1035.5939	1035.5502	42.3	148	-	155	0	---	K.QISLSWFR.D
1036.6012	1035.5939	1035.5502	42.3	148	-	155	0	---	K.QISLSWFR.D

PDF-XChange Click to buy NOW! www.docu-track.com

PDF-XChange Click to buy NOW! www.docu-track.com

1194.6759	1193.6686	1193.6233	37.9	54 -	62	0	---	R.FWTFPEVLR.D
1194.6759	1193.6686	1193.6233	37.9	54 -	62	0	18	R.FWTFPEVLR.D
1266.6686	1265.6613	1265.6040	45.3	269 -	278	0	---	K.ALETHTYFER.H
1595.9777	1594.9704	1594.9083	38.9	110 -	124	0	---	K.AEVLSPVVSVFVPPR.N
1595.9777	1594.9704	1594.9083	39.0	110 -	124	0	---	K.AEVLSPVVSVFVPPR.N
1633.9124	1632.9051	1632.8413	39.1	357 -	370	0	---	K.GFAPADVFVQWLQR.G
1784.0413	1783.0340	1782.9702	35.8	325 -	340	0	---	K.DVAMKPPSVYLLPPTR.E

No match to: 842.5546, 848.5696, 850.5803, 853.5549, 853.5549, 855.0955, 864.5864, 868.5867, 868.5867, 871.0599, 877.0814, 882.5994, 882.5994, 887.0316, 909.0287, 951.6699, 960.9595, 1017.7114, 1019.5801, 1019.7258, 1033.7395, 1051.7457, 1051.7457, 1060.1041, 1082.0940, 1098.0653, 1151.7755, 1151.7755, 1165.7815, 1165.7815, 1277.7600, 1296.7419, 1334.9314, 1334.9314, 1448.9645, 1448.9645, 1475.8114, 1487.8091, 1618.1177, 1618.1177, 1732.1512, 1902.3138, 1994.0560, 2221.2212, 2284.2476

2. gi|33413902 **Mass:** 49848 **Score:** 50 **Expect:** 3.5e+002 **Matches:** 9
immunoglobulin mu heavy chain constant region [Bos taurus]

Observed	Mr(expt)	Mr(calc)	ppm	Start	End	Miss	Ions	Peptide
1036.6012	1035.5939	1035.5502	42.3	142 -	149	0	---	K.QISLSWFR.D
1036.6012	1035.5939	1035.5502	42.3	142 -	149	0	---	K.QISLSWFR.D
1194.6759	1193.6686	1193.6233	37.9	48 -	56	0	---	R.FWTFPEVLR.D
1194.6759	1193.6686	1193.6233	37.9	48 -	56	0	18	R.FWTFPEVLR.D
1266.6686	1265.6613	1265.6040	45.3	263 -	272	0	---	K.ALETHTYFER.H
1595.9777	1594.9704	1594.9083	38.9	104 -	118	0	---	K.AEVLSPVVSVFVPPR.N
1595.9777	1594.9704	1594.9083	39.0	104 -	118	0	---	K.AEVLSPVVSVFVPPR.N
1633.9124	1632.9051	1632.8413	39.1	351 -	364	0	---	K.GFAPADVFVQWLQR.G
1784.0413	1783.0340	1782.9702	35.8	319 -	334	0	---	K.DVAMKPPSVYLLPPTR.E

No match to: 842.5546, 848.5696, 850.5803, 853.5549, 853.5549, 855.0955, 864.5864, 868.5867, 868.5867, 871.0599, 877.0814, 882.5994, 882.5994, 887.0316, 909.0287, 951.6699, 960.9595, 1017.7114, 1019.5801, 1019.7258, 1033.7395, 1051.7457, 1051.7457, 1060.1041, 1082.0940, 1098.0653, 1151.7755, 1151.7755, 1165.7815, 1165.7815, 1277.7600, 1296.7419, 1334.9314, 1334.9314, 1448.9645, 1448.9645, 1475.8114, 1487.8091, 1618.1177, 1618.1177, 1732.1512, 1902.3138, 1994.0560, 2221.2212, 2284.2476

3. gi|206597414 **Mass:** 28268 **Score:** 50 **Expect:** 3.6e+002 **Matches:** 5
Major histocompatibility complex class II beta chain BMA2, (similar to HLA class II, D beta chain) precursor [Gallus gallus]

Observed	Mr(expt)	Mr(calc)	ppm	Start	End	Miss	Ions	Peptide
1165.7815	1164.7742	1164.7958	-18.54	2 -	12	0	15	M.LVLLGLLLGVR.G
1165.7815	1164.7742	1164.7958	-18.53	2 -	12	0	---	M.LVLLGLLLGVR.G
1296.7419	1295.7346	1295.8363	-78.45	1 -	12	0	---	-.MLVLLGLLLGVR.G
1448.9645	1447.9572	1447.8147	98.4	115 -	128	0	18	R.IVPAQTGNPSVPIR.L
1448.9645	1447.9572	1447.8147	98.4	115 -	128	0	---	R.IVPAQTGNPSVPIR.L

No match to: 842.5546, 848.5696, 850.5803, 853.5549, 853.5549, 855.0955, 864.5864, 868.5867, 868.5867, 871.0599, 877.0814, 882.5994, 882.5994, 887.0316, 909.0287, 951.6699, 960.9595, 1017.7114, 1019.5801, 1019.7258, 1033.7395, 1036.6012, 1036.6012, 1051.7457, 1051.7457, 1060.1041, 1082.0940, 1098.0653, 1151.7755, 1151.7755, 1194.6759, 1194.6759, 1266.6686, 1277.7600, 1334.9314, 1334.9314, 1475.8114, 1487.8091, 1595.9777, 1595.9777, 1618.1177, 1618.1177, 1633.9124, 1732.1512, 1784.0413, 1902.3138, 1994.0560, 2221.2212, 2284.2476

4. gi|34538498 **Mass:** 50536 **Score:** 50 **Expect:** 3.7e+002 **Matches:** 9
immunoglobulin heavy chain constant region [Bos taurus]

Observed	Mr(expt)	Mr(calc)	ppm	Start	End	Miss	Ions	Peptide
1036.6012	1035.5939	1035.5502	42.3	142 -	149	0	---	K.QISLSWFR.D
1036.6012	1035.5939	1035.5502	42.3	142 -	149	0	---	K.QISLSWFR.D
1194.6759	1193.6686	1193.6233	37.9	48 -	56	0	---	R.FWTFPEVLR.D
1194.6759	1193.6686	1193.6233	37.9	48 -	56	0	18	R.FWTFPEVLR.D
1266.6686	1265.6613	1265.6040	45.3	263 -	272	0	---	K.ALETHTYFER.H
1595.9777	1594.9704	1594.9083	38.9	104 -	118	0	---	K.AEVLSPVVSVFVPPR.N
1595.9777	1594.9704	1594.9083	39.0	104 -	118	0	---	K.AEVLSPVVSVFVPPR.N
1633.9124	1632.9051	1632.8413	39.1	351 -	364	0	---	K.GFAPADVFVQWLQR.G
1784.0413	1783.0340	1782.9702	35.8	319 -	334	0	---	K.DVAMKPPSVYLLPPTR.E

No match to: 842.5546, 848.5696, 850.5803, 853.5549, 853.5549, 855.0955, 864.5864, 868.5867, 868.5867, 871.0599, 877.0814, 882.5994, 882.5994, 887.0316, 909.0287, 951.6699, 960.9595, 1017.7114, 1019.5801, 1019.7258, 1033.7395, 1051.7457, 1051.7457, 1060.1041, 1082.0940, 1098.0653, 1151.7755, 1151.7755, 1165.7815, 1165.7815, 1277.7600, 1296.7419, 1334.9314, 1334.9314, 1448.9645, 1448.9645, 1475.8114, 1487.8091, 1618.1177, 1618.1177, 1732.1512, 1902.3138, 1994.0560, 2221.2212, 2284.2476

5. gi|518349625 **Mass:** 5336 **Score:** 48 **Expect:** 4.9e+002 **Matches:** 7
hypothetical protein [Moraxella boevrei]

Observed	Mr(expt)	Mr(calc)	ppm	Start	End	Miss	Ions	Peptide
848.5696	847.5623	847.5068	65.5	23 -	28	1	---	K.WRFLVK.I
853.5549	852.5476	852.5109	43.1	12 -	18	0	---	K.IGYLFLK.I
853.5549	852.5477	852.5109	43.1	12 -	18	0	---	K.IGYLFLK.I
1165.7815	1164.7742	1164.7270	40.5	12 -	21	1	---	K.IGYLFLKIAK.K
1165.7815	1164.7742	1164.7270	40.5	12 -	21	1	---	K.IGYLFLKIAK.K
1277.7600	1276.7527	1276.6856	52.6	2 -	11	1	---	M.FGYLFDFIKK.I
1296.7419	1295.7346	1295.6260	83.8	1 -	10	0	---	-.MFGYLFDFIK.K + Oxidation (M)

No match to: 842.5546, 850.5803, 855.0955, 864.5864, 868.5867, 868.5867, 871.0599, 877.0814, 882.5994, 882.5994, 887.0316, 909.0287, 951.6699, 960.9595, 1017.7114, 1019.5801, 1019.7258, 1033.7395, 1036.6012, 1036.6012, 1051.7457, 1051.7457, 1060.1041, 1082.0940, 1098.0653, 1151.7755, 1151.7755, 1194.6759, 1194.6759, 1266.6686, 1334.9314, 1334.9314, 1448.9645, 1448.9645, 1475.8114, 1487.8091, 1595.9777, 1595.9777, 1618.1177, 1618.1177, 1633.9124, 1732.1512, 1784.0413, 1902.3138, 1994.0560, 2221.2212, 2284.2476

6. gi|494742220 **Mass:** 17861 **Score:** 47 **Expect:** 7.1e+002 **Matches:** 8
3-hydroxyacyl-ACP dehydratase [Bartonella melophagi]

Observed	Mr(expt)	Mr(calc)	ppm	Start	End	Miss	Ions	Peptide
850.5803	849.5730	849.5436	34.6	117 -	123	0	---	K.LHVQLLK.K
1019.5801	1018.5728	1018.5183	53.6	9 -	17	0	---	K.SLETVDIDK.L
1036.6012	1035.5939	1035.5753	18.0	26 -	33	0	---	R.YPFLLIDR.I
1036.6012	1035.5939	1035.5753	18.0	26 -	33	0	---	R.YPFLLIDR.I
1266.6686	1265.6613	1265.6074	42.6	131 -	141	0	---	R.FSCVAEVEGIR.V
1595.9777	1594.9704	1594.8752	59.7	91 -	104	1	---	K.KTSLIYLMTVNNAK.F
1595.9777	1594.9704	1594.8752	59.7	91 -	104	1	---	K.KTSLIYLMTVNNAK.F
1994.0560	1993.0487	1992.9747	37.1	142 -	159	0	---	R.VSEAEVSAMIVELEQTMK.-

No match to: 842.5546, 848.5696, 853.5549, 853.5549, 855.0955, 864.5864, 868.5867, 868.5867, 871.0599, 877.0814, 882.5994, 882.5994, 887.0316, 909.0287, 951.6699, 960.9595, 1017.7114, 1019.7258, 1033.7395, 1051.7457, 1051.7457, 1060.1041, 1082.0940, 1098.0653, 1151.7755, 1151.7755, 1165.7815, 1165.7815, 1194.6759, 1194.6759, 1277.7600, 1296.7419, 1334.9314, 1334.9314, 1448.9645, 1448.9645, 1475.8114, 1487.8091, 1618.1177, 1618.1177, 1633.9124, 1732.1512, 1784.0413, 1902.3138, 2221.2212, 2284.2476

7. gi|518846823 **Mass:** 5061 **Score:** 46 **Expect:** 7.9e+002 **Matches:** 6
hypothetical protein [Mycoplasma hominis]

PDF-XChange Click to buy NOW! www.docu-track.com

PDF-XChange Click to buy NOW! www.docu-track.com

Observed	Mr(expt)	Mr(calc)	ppm	Start	End	Miss	Ions	Peptide
1296.7419	1295.7346	1295.6948	30.8	33	42	1	---	K.IFLCNEKLLF.-
1448.9645	1447.9572	1447.9418	10.6	2	13	0	---	M.LVYITIILLIFK.G
1448.9645	1447.9572	1447.9418	10.7	2	13	0	---	M.LVYITIILLIFK.G
1595.9777	1594.9704	1594.9772	-4.27	1	13	0	---	-.MLVYITIILLIFK.G + Oxidation (M)
1595.9777	1594.9704	1594.9772	-4.24	1	13	0	---	-.MLVYITIILLIFK.G + Oxidation (M)
1633.9124	1632.9051	1633.0582	-93.74	2	15	1	---	M.LVYITIILLIFKGK.K

No match to: 842.5546, 848.5696, 850.5803, 853.5549, 853.5549, 855.0955, 864.5864, 868.5867, 868.5867, 871.0599, 877.0814, 882.5994, 882.5994, 887.0316, 909.0287, 951.6699, 960.9595, 1017.7114, 1019.5801, 1019.7258, 1033.7395, 1036.6012, 1036.6012, 1051.7457, 1051.7457, 1060.1041, 1082.0940, 1098.0653, 1151.7755, 1151.7755, 1165.7815, 1165.7815, 1194.6759, 1194.6759, 1266.6686, 1277.7600, 1334.9314, 1334.9314, 1475.8114, 1487.8091, 1618.1177, 1618.1177, 1732.1512, 1784.0413, 1902.3138, 1994.0560, 2221.2212, 2284.2476

8. gi|488818860 **Mass:** 9345 **Score:** 45 **Expect:** 1.1e+003 **Matches:** 6

hypothetical protein [Phaeospirillum molischianum]

Observed	Mr(expt)	Mr(calc)	ppm	Start	End	Miss	Ions	Peptide
1019.5801	1018.5728	1018.4985	73.0	47	54	0	---	R.ENFIHGFR.H
1036.6012	1035.5939	1035.6441	-48.44	4	13	0	---	K.LPTVVVGPVR.K
1036.6012	1035.5939	1035.6441	-48.44	4	13	0	---	K.LPTVVVGPVR.K
1266.6686	1265.6613	1265.6326	22.7	62	72	0	---	K.VLTPMSQSEFK.R
1277.7600	1276.7527	1276.8231	-55.12	2	13	1	---	M.IKLPTVVVGPVR.K
1784.0413	1783.0340	1782.8988	75.9	47	61	1	---	R.ENFIHGFRHPVTMAK.V

No match to: 842.5546, 848.5696, 850.5803, 853.5549, 853.5549, 855.0955, 864.5864, 868.5867, 868.5867, 871.0599, 877.0814, 882.5994, 882.5994, 887.0316, 909.0287, 951.6699, 960.9595, 1017.7114, 1019.7258, 1033.7395, 1051.7457, 1051.7457, 1060.1041, 1082.0940, 1098.0653, 1151.7755, 1151.7755, 1165.7815, 1165.7815, 1194.6759, 1194.6759, 1296.7419, 1334.9314, 1334.9314, 1448.9645, 1448.9645, 1475.8114, 1487.8091, 1595.9777, 1595.9777, 1618.1177, 1618.1177, 1633.9124, 1732.1512, 1902.3138, 1994.0560, 2221.2212, 2284.2476

9. gi|446609084 **Mass:** 31602 **Score:** 45 **Expect:** 1.1e+003 **Matches:** 7

prephenate dehydratase [Streptococcus oralis]

Observed	Mr(expt)	Mr(calc)	ppm	Start	End	Miss	Ions	Peptide
842.5546	841.5473	841.5021	53.7	223	229	0	---	K.IESRPLK.T
1036.6012	1035.5939	1035.5787	14.7	1	9	1	---	-.MKIAYLGPK.G + Oxidation (M)
1036.6012	1035.5939	1035.5787	14.7	1	9	1	3	-.MKIAYLGPK.G + Oxidation (M)
1266.6686	1265.6613	1265.5750	68.2	111	120	0	---	K.FIDMHYPEAK.I + Oxidation (M)
1296.7419	1295.7346	1295.6986	27.8	98	109	0	---	K.IFSHPQALAQGK.K
1784.0413	1783.0340	1782.8941	78.5	133	148	0	---	R.FISEHPDQPYAAIAPK.S
2284.2476	2283.2403	2283.2164	10.5	3	23	1	---	K.IAYLGPKGSFSHHVVQTAFPK.E

No match to: 848.5696, 850.5803, 853.5549, 853.5549, 855.0955, 864.5864, 868.5867, 868.5867, 871.0599, 877.0814, 882.5994, 882.5994, 887.0316, 909.0287, 951.6699, 960.9595, 1017.7114, 1019.5801, 1019.7258, 1033.7395, 1051.7457, 1051.7457, 1060.1041, 1082.0940, 1098.0653, 1151.7755, 1151.7755, 1165.7815, 1165.7815, 1194.6759, 1194.6759, 1277.7600, 1334.9314, 1334.9314, 1448.9645, 1448.9645, 1475.8114, 1487.8091, 1595.9777, 1595.9777, 1618.1177, 1618.1177, 1633.9124, 1732.1512, 1902.3138, 1994.0560, 2221.2212

10. gi|518026209 **Mass:** 30111 **Score:** 44 **Expect:** 1.3e+003 **Matches:** 7

serine acetyltransferase [Afipia birgiae]

Observed	Mr(expt)	Mr(calc)	ppm	Start	End	Miss	Ions	Peptide
1036.6012	1035.5939	1035.5362	55.7	116	124	0	---	K.GFHAIQAHR.L
1036.6012	1035.5939	1035.5362	55.7	116	124	0	---	K.GFHAIQAHR.L
1266.6686	1265.6613	1265.6418	15.5	125	134	0	---	R.LAHWLYHNGR.R
1296.7419	1295.7346	1295.6834	39.6	62	73	0	---	R.LDHSALSGDLIR.Q
1994.0560	1993.0487	1992.9177	65.7	74	91	0	---	R.QAYNDAIEAEPDLGNAFR.A
2221.2212	2220.2139	2220.2478	-15.26	229	251	1	---	R.VASGSVVLKDVPNNVTVAGVPAK.I
2284.2476	2283.2403	2283.1674	31.9	116	134	1	---	K.GFHAIQAHRLAHWLYHNGR.R

No match to: 842.5546, 848.5696, 850.5803, 853.5549, 853.5549, 855.0955, 864.5864, 868.5867, 868.5867, 871.0599, 877.0814, 882.5994, 882.5994, 887.0316, 909.0287, 951.6699, 960.9595, 1017.7114, 1019.5801, 1019.7258, 1033.7395, 1051.7457, 1051.7457, 1060.1041, 1082.0940, 1098.0653, 1151.7755, 1151.7755, 1165.7815, 1165.7815, 1194.6759, 1194.6759, 1277.7600, 1334.9314, 1334.9314, 1448.9645, 1448.9645, 1475.8114, 1487.8091, 1595.9777, 1595.9777, 1618.1177, 1618.1177, 1633.9124, 1732.1512, 1784.0413, 1902.3138

11. gi|24496448 **Mass:** 66533 **Score:** 44 **Expect:** 1.3e+003 **Matches:** 9

immunoglobulin M heavy chain secretory form [Bos taurus]

Observed	Mr(expt)	Mr(calc)	ppm	Start	End	Miss	Ions	Peptide
1036.6012	1035.5939	1035.5502	42.3	292	299	0	---	K.QISLSWFR.D
1036.6012	1035.5939	1035.5502	42.3	292	299	0	---	K.QISLSWFR.D
1194.6759	1193.6686	1193.6233	37.9	198	206	0	---	R.FWTFPEVLR.D
1194.6759	1193.6686	1193.6233	37.9	198	206	0	18	R.FWTFPEVLR.D
1266.6686	1265.6613	1265.6040	45.3	413	422	0	---	K.ALETHTYFER.H
1595.9777	1594.9704	1594.9083	38.9	254	268	0	---	K.AEVLSPVVSVFVPPR.N
1595.9777	1594.9704	1594.9083	39.0	254	268	0	---	K.AEVLSPVVSVFVPPR.N
1633.9124	1632.9051	1632.8413	39.1	501	514	0	---	K.GFAPADVFVQWLQR.G
1784.0413	1783.0340	1782.9702	35.8	469	484	0	---	K.DVAMKPPSVYLLPPTR.E

No match to: 842.5546, 848.5696, 850.5803, 853.5549, 853.5549, 855.0955, 864.5864, 868.5867, 868.5867, 871.0599, 877.0814, 882.5994, 882.5994, 887.0316, 909.0287, 951.6699, 960.9595, 1017.7114, 1019.5801, 1019.7258, 1033.7395, 1051.7457, 1051.7457, 1060.1041, 1082.0940, 1098.0653, 1151.7755, 1151.7755, 1165.7815, 1165.7815, 1277.7600, 1296.7419, 1334.9314, 1334.9314, 1448.9645, 1448.9645, 1475.8114, 1487.8091, 1618.1177, 1618.1177, 1732.1512, 1902.3138, 1994.0560, 2221.2212, 2284.2476

12. gi|547755423 **Mass:** 19340 **Score:** 44 **Expect:** 1.4e+003 **Matches:** 8

ribosome maturation factor RimM [Clostridium sp. CAG:221]

Observed	Mr(expt)	Mr(calc)	ppm	Start	End	Miss	Ions	Peptide
853.5549	852.5476	852.4817	77.3	10	17	0	---	K.IANTHGLK.G
853.5549	852.5477	852.4817	77.3	10	17	0	---	K.IANTHGLK.G
1019.5801	1018.5728	1018.5845	-11.45	1	9	1	---	-.MSQLLKVGK.I + Oxidation (M)
1266.6686	1265.6613	1265.7092	-37.80	10	21	1	---	K.IANTHGLKGEVK.V
1277.7600	1276.7527	1276.8482	-74.78	130	140	1	---	R.KPKELLIPVLK.S
1296.7419	1295.7346	1295.6544	61.9	44	54	1	---	K.TISVEGCKFQK.D
1595.9777	1594.9704	1594.8355	84.6	31	43	1	---	K.RFNDLEYVIIEGK.T
1595.9777	1594.9704	1594.8355	84.6	31	43	1	---	K.RFNDLEYVIIEGK.T

No match to: 842.5546, 848.5696, 850.5803, 855.0955, 864.5864, 868.5867, 871.0599, 877.0814, 882.5994, 882.5994, 887.0316, 909.0287, 951.6699, 960.9595, 1017.7114, 1019.7258, 1033.7395, 1036.6012, 1036.6012, 1051.7457, 1051.7457, 1060.1041, 1082.0940, 1098.0653, 1151.7755, 1151.7755, 1165.7815, 1165.7815, 1194.6759, 1194.6759, 1334.9314, 1334.9314, 1448.9645, 1448.9645, 1475.8114, 1487.8091, 1618.1177, 1618.1177, 1633.9124, 1732.1512, 1784.0413, 1902.3138, 1994.0560, 2221.2212, 2284.2476

13. gi|492049521 **Mass:** 6980 **Score:** 43 **Expect:** 1.5e+003 **Matches:** 6

hypothetical protein [Pseudomonas amygdali]

PDF-XChange Click to buy NOW! www.docu-track.com

PDF-XChange Click to buy NOW! www.docu-track.com

Observed	Mr(expt)	Mr(calc)	ppm	Start	End	Miss	Ions	Peptide
1036.6012	1035.5939	1035.5899	3.88	8 -	16	0	---	R.QVMLHIPAK.A
1036.6012	1035.5939	1035.5899	3.88	8 -	16	0	---	R.QVMLHIPAK.A
1618.1177	1617.1104	1616.9548	96.2	8 -	21	1	---	R.QVMLHIPAKALKPR.A + Oxidation (M)
1618.1177	1617.1104	1616.9548	96.2	8 -	21	1	---	R.QVMLHIPAKALKPR.A + Oxidation (M)
1784.0413	1783.0340	1783.0178	9.08	2 -	16	1	---	M.TILFTRQVMLHIPAK.A + Oxidation (M)
2221.2212	2220.2139	2220.1069	48.2	25 -	46	0	---	R.AVNIASVGGEQVMGNAVPAQHR.G + Oxidation (M)

No match to: 842.5546, 848.5696, 850.5803, 853.5549, 853.5549, 855.0955, 864.5864, 868.5867, 868.5867, 871.0599, 877.0814, 882.5994, 882.5994, 887.0316, 909.0287, 951.6699, 960.9595, 1017.7114, 1019.5801, 1019.7258, 1033.7395, 1051.7457, 1051.7457, 1060.1041, 1082.0940, 1098.0653, 1151.7755, 1151.7755, 1165.7815, 1165.7815, 1194.6759, 1194.6759, 1266.6686, 1277.7600, 1296.7419, 1334.9314, 1334.9314, 1448.9645, 1448.9645, 1475.8114, 1487.8091, 1595.9777, 1595.9777, 1633.9124, 1732.1512, 1902.3138, 1994.0560, 2284.2476

14. gi|488127006 **Mass:** 5046 **Score:** 43 **Expect:** 1.6e+003 **Matches:** 6
hypothetical protein, partial [Helicobacter pylori]

Observed	Mr(expt)	Mr(calc)	ppm	Start	End	Miss	Ions	Peptide
853.5549	852.5476	852.4779	81.8	34 -	40	0	---	R.MVIFISK.A + Oxidation (M)
853.5549	852.5477	852.4779	81.8	34 -	40	0	---	R.MVIFISK.A + Oxidation (M)
864.5864	863.5791	863.5116	78.2	19 -	25	1	---	K.EALKYLK.T
1165.7815	1164.7742	1164.6689	90.4	31 -	40	1	---	K.ATRMVIFISK.A
1165.7815	1164.7742	1164.6689	90.4	31 -	40	1	---	K.ATRMVIFISK.A
1266.6686	1265.6613	1265.6876	-20.75	34 -	44	1	---	R.MVIFISKACVV.-

No match to: 842.5546, 848.5696, 850.5803, 855.0955, 868.5867, 868.5867, 871.0599, 877.0814, 882.5994, 882.5994, 887.0316, 909.0287, 951.6699, 960.9595, 1017.7114, 1019.5801, 1019.7258, 1033.7395, 1036.6012, 1036.6012, 1051.7457, 1051.7457, 1060.1041, 1082.0940, 1098.0653, 1151.7755, 1151.7755, 1194.6759, 1194.6759, 1277.7600, 1296.7419, 1334.9314, 1334.9314, 1448.9645, 1448.9645, 1475.8114, 1487.8091, 1595.9777, 1595.9777, 1618.1177, 1618.1177, 1633.9124, 1732.1512, 1784.0413, 1902.3138, 1994.0560, 2221.2212, 2284.2476

15. gi|68535459 **Mass:** 8368 **Score:** 43 **Expect:** 1.7e+003 **Matches:** 5
hypothetical protein jk0392 [Corynebacterium jeikeium K411]

Observed	Mr(expt)	Mr(calc)	ppm	Start	End	Miss	Ions	Peptide
1194.6759	1193.6686	1193.6081	50.7	62 -	71	1	---	K.AFVEKNPDFK.E
1194.6759	1193.6686	1193.6081	50.7	62 -	71	1	---	K.AFVEKNPDFK.E
1296.7419	1295.7346	1295.6544	62.0	55 -	66	1	---	K.ASCSAVKAFVEK.N
1475.8114	1474.8041	1474.6841	81.4	19 -	30	0	---	R.DFNHTVVDPEFR.G
1784.0413	1783.0340	1783.0091	14.0	31 -	47	0	---	R.GQGLSKPLISEALLETK.E

No match to: 842.5546, 848.5696, 850.5803, 853.5549, 853.5549, 855.0955, 864.5864, 868.5867, 868.5867, 871.0599, 877.0814, 882.5994, 882.5994, 887.0316, 909.0287, 951.6699, 960.9595, 1017.7114, 1019.5801, 1019.7258, 1033.7395, 1036.6012, 1036.6012, 1051.7457, 1051.7457, 1060.1041, 1082.0940, 1098.0653, 1151.7755, 1151.7755, 1165.7815, 1165.7815, 1266.6686, 1277.7600, 1334.9314, 1334.9314, 1448.9645, 1448.9645, 1487.8091, 1595.9777, 1595.9777, 1618.1177, 1618.1177, 1633.9124, 1732.1512, 1902.3138, 1994.0560, 2221.2212, 2284.2476

16. gi|3834667 **Mass:** 48810 **Score:** 42 **Expect:** 2.2e+003 **Matches:** 7
immunoglobulin heavy chain constant region [Bos taurus]

Observed	Mr(expt)	Mr(calc)	ppm	Start	End	Miss	Ions	Peptide
1036.6012	1035.5939	1035.5502	42.3	151 -	158	0	---	K.QISLSWFR.D
1036.6012	1035.5939	1035.5502	42.3	151 -	158	0	---	K.QISLSWFR.D
1194.6759	1193.6686	1193.6233	37.9	54 -	62	0	---	R.FWTFPEVLR.D
1194.6759	1193.6686	1193.6233	37.9	54 -	62	0	18	R.FWTFPEVLR.D
1266.6686	1265.6613	1265.6040	45.3	272 -	281	0	---	K.ALETHTYFER.H
1633.9124	1632.9051	1632.8413	39.1	360 -	373	0	---	K.GFAPADVFVQWLQR.G
1784.0413	1783.0340	1782.9702	35.8	328 -	343	0	---	K.DVAMKPPSVYLLPPTR.E

No match to: 842.5546, 848.5696, 850.5803, 853.5549, 853.5549, 855.0955, 864.5864, 868.5867, 868.5867, 871.0599, 877.0814, 882.5994, 882.5994, 887.0316, 909.0287, 951.6699, 960.9595, 1017.7114, 1019.5801, 1019.7258, 1033.7395, 1051.7457, 1051.7457, 1060.1041, 1082.0940, 1098.0653, 1151.7755, 1151.7755, 1165.7815, 1165.7815, 1277.7600, 1296.7419, 1334.9314, 1334.9314, 1448.9645, 1448.9645, 1475.8114, 1487.8091, 1595.9777, 1595.9777, 1618.1177, 1618.1177, 1732.1512, 1902.3138, 1994.0560, 2221.2212, 2284.2476

17. gi|547238705 **Mass:** 13800 **Score:** 42 **Expect:** 2.4e+003 **Matches:** 7
ribosomal protein S13 [Firmicutes bacterium CAG:884]

Observed	Mr(expt)	Mr(calc)	ppm	Start	End	Miss	Ions	Peptide
882.5994	881.5921	881.5195	82.3	94 -	101	1	---	K.GLPVRGQR.T
882.5994	881.5922	881.5195	82.4	94 -	101	1	---	K.GLPVRGQR.T
1019.5801	1018.5728	1018.5229	49.0	71 -	78	1	---	R.REVNMNIK.T + Oxidation (M)
1475.8114	1474.8041	1474.7350	46.8	81 -	92	1	---	K.MEINSYQGIRHK.K
1595.9777	1594.9704	1594.9195	31.9	14 -	27	1	---	K.RTVIALTYIYGIGR.S
1595.9777	1594.9704	1594.9195	31.9	14 -	27	1	---	K.RTVIALTYIYGIGR.S
2284.2476	2283.2403	2283.2661	-11.27	15 -	35	1	---	R.TVIALTYIYGIGRSLAATICK.D

No match to: 842.5546, 848.5696, 850.5803, 853.5549, 853.5549, 855.0955, 864.5864, 868.5867, 868.5867, 871.0599, 877.0814, 887.0316, 909.0287, 951.6699, 960.9595, 1017.7114, 1019.7258, 1033.7395, 1036.6012, 1036.6012, 1051.7457, 1051.7457, 1060.1041, 1082.0940, 1098.0653, 1151.7755, 1151.7755, 1165.7815, 1165.7815, 1194.6759, 1194.6759, 1266.6686, 1277.7600, 1296.7419, 1334.9314, 1334.9314, 1448.9645, 1448.9645, 1487.8091, 1618.1177, 1618.1177, 1633.9124, 1732.1512, 1784.0413, 1902.3138, 1994.0560, 2221.2212

18. gi|345564418 **Mass:** 28894 **Score:** 41 **Expect:** 2.7e+003 **Matches:** 4
hypothetical protein AOL_s00083g474 [Arthrobotrys oligospora ATCC 24927]

Observed	Mr(expt)	Mr(calc)	ppm	Start	End	Miss	Ions	Peptide
1194.6759	1193.6686	1193.6445	20.2	176 -	185	0	---	R.FNVSVQYLPK.D
1194.6759	1193.6686	1193.6233	38.0	43 -	51	0	18	R.LPWYLSPYR.G
1633.9124	1632.9051	1632.7678	84.1	26 -	38	1	---	K.EITNDDRHFSMLR.Y
2221.2212	2220.2139	2220.0805	60.1	4 -	25	1	---	R.QRPTSSSSPAVASNGGVDTKMK.E + Oxidation (M)

No match to: 842.5546, 848.5696, 850.5803, 853.5549, 853.5549, 855.0955, 864.5864, 868.5867, 868.5867, 871.0599, 877.0814, 882.5994, 882.5994, 887.0316, 909.0287, 951.6699, 960.9595, 1017.7114, 1019.5801, 1019.7258, 1033.7395, 1036.6012, 1036.6012, 1051.7457, 1051.7457, 1060.1041, 1082.0940, 1098.0653, 1151.7755, 1151.7755, 1165.7815, 1165.7815, 1266.6686, 1277.7600, 1296.7419, 1334.9314, 1334.9314, 1448.9645, 1448.9645, 1475.8114, 1487.8091, 1595.9777, 1595.9777, 1618.1177, 1618.1177, 1732.1512, 1784.0413, 1902.3138, 1994.0560, 2284.2476

19. gi|517947175 **Mass:** 20170 **Score:** 41 **Expect:** 2.7e+003 **Matches:** 7
hypothetical protein [Anaerococcus sp. PH9]

Observed	Mr(expt)	Mr(calc)	ppm	Start	End	Miss	Ions	Peptide
1036.6012	1035.5939	1035.4985	92.1	12 -	21	0	---	R.DSGNANFALK.L
1036.6012	1035.5939	1035.4985	92.1	12 -	21	0	---	R.DSGNANFALK.L
1194.6759	1193.6686	1193.6153	44.7	22 -	31	1	---	K.LVGTQRYSDR.E
1194.6759	1193.6686	1193.6153	44.7	22 -	31	1	---	K.LVGTQRYSDR.E
1266.6686	1265.6613	1265.6438	13.9	1 -	11	0	---	-.MDLLQAVAYAR.D + Oxidation (M)
1487.8091	1486.8018	1486.6875	76.9	91 -	103	0	---	R.GCYVISYDAGNLR.Y

PDF-XChange Click to buy NOW! www.docu-track.com

2284.2476	2283.2403	2283.1317	47.6	1	21	1	---	-.MDLLQAVAYARDSGNANFALK.L + Oxidation (M)

No match to: 842.5546, 848.5696, 850.5803, 853.5549, 855.0955, 864.5864, 868.5867, 868.5867, 871.0599, 877.0814, 882.5994, 882.5994, 887.0316, 909.0287, 951.6699, 960.9595, 1017.7114, 1019.5801, 1019.7258, 1033.7395, 1051.7457, 1051.7457, 1060.1041, 1082.0940, 1098.0653, 1151.7755, 1151.7755, 1165.7815, 1165.7815, 1277.7600, 1296.7419, 1334.9314, 1334.9314, 1448.9645, 1448.9645, 1475.8114, 1595.9777, 1595.9777, 1618.1177, 1618.1177, 1633.9124, 1732.1512, 1784.0413, 1902.3138, 1994.0560, 2221.2212

20. gi|522808524 **Mass:** 25797 **Score:** 41 **Expect:** 2.7e+003 **Matches:** 8

hypothetical protein [Mycobacterium marinum]

Observed	Mr(expt)	Mr(calc)	ppm	Start	End	Miss	Ions	Peptide
951.6699	950.6626	950.5774	89.7	195	202	1	---	R.QLRKPGPR.Q
1151.7755	1150.7682	1150.7074	52.9	31	41	0	---	R.SVPLSLLVAPR.L
1151.7755	1150.7682	1150.7074	52.9	31	41	0	---	R.SVPLSLLVAPR.L
1266.6686	1265.6613	1265.6153	36.4	51	61	0	---	R.DPHTVDWLAGR.R
1595.9777	1594.9704	1594.8613	68.4	97	110	1	---	R.VMAADRVLEHLGLR.T + Oxidation (M)
1595.9777	1594.9704	1594.8613	68.4	97	110	1	---	R.VMAADRVLEHLGLR.T + Oxidation (M)
1784.0413	1783.0340	1783.0145	11.0	111	127	1	---	R.TRLFAAPGWVVSPGVVK.A
2284.2476	2283.2403	2283.2124	12.2	128	148	1	---	K.ALPGNGFRLLADYHGISDLVR.K

No match to: 842.5546, 848.5696, 850.5803, 853.5549, 853.5549, 855.0955, 864.5864, 868.5867, 868.5867, 871.0599, 877.0814, 882.5994, 882.5994, 887.0316, 909.0287, 960.9595, 1017.7114, 1019.5801, 1019.7258, 1033.7395, 1036.6012, 1036.6012, 1051.7457, 1051.7457, 1060.1041, 1082.0940, 1098.0653, 1165.7815, 1165.7815, 1194.6759, 1194.6759, 1277.7600, 1296.7419, 1334.9314, 1334.9314, 1448.9645, 1448.9645, 1475.8114, 1487.8091, 1618.1177, 1618.1177, 1633.9124, 1732.1512, 1902.3138, 1994.0560, 2221.2212

21. gi|550715204 **Mass:** 37257 **Score:** 41 **Expect:** 2.9e+003 **Matches:** 7

transcriptional activator FtrA [Enterobacter sp. MGH 8]

Observed	Mr(expt)	Mr(calc)	ppm	Start	End	Miss	Ions	Peptide
1165.7815	1164.7742	1164.6880	74.0	273	281	1	---	R.WLLTARLHR.A
1165.7815	1164.7742	1164.6880	74.1	273	281	1	---	R.WLLTARLHR.A
1194.6759	1193.6686	1193.6339	29.1	251	260	1	---	R.VGMSQRTFLR.R
1194.6759	1193.6686	1193.6339	29.1	251	260	1	11	R.VGMSQRTFLR.R
1475.8114	1474.8041	1474.7681	24.4	136	147	1	---	K.ATTHWRYTQALK.T
1784.0413	1783.0340	1782.9337	56.3	207	222	0	---	R.DGSQTQQLSRPVAQLR.E
2284.2476	2283.2403	2283.1680	31.7	207	226	1	---	R.DGSQTQQLSRPVAQLRESQR.L

No match to: 842.5546, 848.5696, 850.5803, 853.5549, 853.5549, 855.0955, 864.5864, 868.5867, 868.5867, 871.0599, 877.0814, 882.5994, 882.5994, 887.0316, 909.0287, 951.6699, 960.9595, 1017.7114, 1019.5801, 1019.7258, 1033.7395, 1036.6012, 1036.6012, 1051.7457, 1051.7457, 1060.1041, 1082.0940, 1098.0653, 1151.7755, 1151.7755, 1266.6686, 1277.7600, 1296.7419, 1334.9314, 1334.9314, 1448.9645, 1448.9645, 1487.8091, 1595.9777, 1595.9777, 1618.1177, 1618.1177, 1633.9124, 1732.1512, 1902.3138, 1994.0560, 2221.2212

22. gi|516231730 **Mass:** 9834 **Score:** 41 **Expect:** 2.9e+003 **Matches:** 6

hypothetical protein [Vibrio sp. 712i1]

Observed	Mr(expt)	Mr(calc)	ppm	Start	End	Miss	Ions	Peptide
853.5549	852.5476	852.5109	43.0	5	11	0	---	R.LFFIVSK.T
853.5549	852.5477	852.5109	43.1	5	11	0	---	R.LFFIVSK.T
1165.7815	1164.7742	1164.7019	62.1	2	11	1	---	M.VGRLFFIVSK.T
1165.7815	1164.7742	1164.7019	62.1	2	11	1	---	M.VGRLFFIVSK.T
1296.7419	1295.7346	1295.7424	-6.00	1	11	1	---	-.MVGRLFFIVSK.T
1994.0560	1993.0487	1993.0567	-4.01	67	83	1	---	R.LNHTMFSAIIEPIAKHR.- + Oxidation (M)

No match to: 842.5546, 848.5696, 850.5803, 855.0955, 864.5864, 868.5867, 868.5867, 871.0599, 877.0814, 882.5994, 882.5994, 887.0316, 909.0287, 951.6699, 960.9595, 1017.7114, 1019.5801, 1019.7258, 1033.7395, 1036.6012, 1036.6012, 1051.7457, 1051.7457, 1060.1041, 1082.0940, 1098.0653, 1151.7755, 1151.7755, 1194.6759, 1194.6759, 1266.6686, 1277.7600, 1334.9314, 1334.9314, 1448.9645, 1448.9645, 1475.8114, 1487.8091, 1595.9777, 1595.9777, 1618.1177, 1618.1177, 1633.9124, 1732.1512, 1784.0413, 1902.3138, 2221.2212, 2284.2476

23. gi|517496258 **Mass:** 32907 **Score:** 40 **Expect:** 3e+003 **Matches:** 6

hypothetical protein [Bacteroides gallinarum]

Observed	Mr(expt)	Mr(calc)	ppm	Start	End	Miss	Ions	Peptide
842.5546	841.5473	841.5749	-32.76	289	295	1	---	R.LLLLKSR.-
1194.6759	1193.6686	1193.5533	96.6	52	61	0	---	K.GNTKPMDMIR.I + 2 Oxidation (M)
1194.6759	1193.6686	1193.5533	96.6	52	61	0	11	K.GNTKPMDMIR.I + 2 Oxidation (M)
1266.6686	1265.6613	1265.6299	24.8	134	145	1	---	R.RAGGAGYSCVLR.L
1994.0560	1993.0487	1992.9873	30.8	52	68	1	---	K.GNTKPMDMIRIYEGNVR.A
2284.2476	2283.2403	2283.3065	-28.97	4	24	1	---	K.IFLLFVMITLALGAFAQSKGK.N + Oxidation (M)

No match to: 848.5696, 850.5803, 853.5549, 853.5549, 855.0955, 864.5864, 868.5867, 868.5867, 871.0599, 877.0814, 882.5994, 882.5994, 887.0316, 909.0287, 951.6699, 960.9595, 1017.7114, 1019.5801, 1019.7258, 1033.7395, 1036.6012, 1036.6012, 1051.7457, 1051.7457, 1060.1041, 1082.0940, 1098.0653, 1151.7755, 1151.7755, 1165.7815, 1165.7815, 1277.7600, 1296.7419, 1334.9314, 1334.9314, 1448.9645, 1448.9645, 1475.8114, 1487.8091, 1595.9777, 1595.9777, 1618.1177, 1618.1177, 1633.9124, 1732.1512, 1784.0413, 1902.3138, 2221.2212

24. gi|183984872 **Mass:** 25857 **Score:** 40 **Expect:** 3.4e+003 **Matches:** 8

hypothetical protein MMAR_4904 [Mycobacterium marinum M]

Observed	Mr(expt)	Mr(calc)	ppm	Start	End	Miss	Ions	Peptide
951.6699	950.6626	950.5774	89.7	195	202	1	---	R.QLRKPGPR.Q
1151.7755	1150.7682	1150.7074	52.9	31	41	0	---	R.SVPLSLLVAPR.L
1151.7755	1150.7682	1150.7074	52.9	31	41	0	---	R.SVPLSLLVAPR.L
1266.6686	1265.6613	1265.6153	36.4	51	61	0	---	R.DPHTVDWLAGR.R
1595.9777	1594.9704	1594.8613	68.4	97	110	1	---	R.VMAADRVLEHLGLR.T + Oxidation (M)
1595.9777	1594.9704	1594.8613	68.4	97	110	1	---	R.VMAADRVLEHLGLR.T + Oxidation (M)
1784.0413	1783.0340	1783.0145	11.0	111	127	1	---	R.TRLFAAPGWVVSPGVVK.A
2284.2476	2283.2403	2283.2124	12.2	128	148	1	---	K.ALPGNGFRLLADYHGISDLVR.K

No match to: 842.5546, 848.5696, 850.5803, 853.5549, 853.5549, 855.0955, 864.5864, 868.5867, 868.5867, 871.0599, 877.0814, 882.5994, 882.5994, 887.0316, 909.0287, 960.9595, 1017.7114, 1019.5801, 1019.7258, 1033.7395, 1036.6012, 1036.6012, 1051.7457, 1051.7457, 1060.1041, 1082.0940, 1098.0653, 1165.7815, 1165.7815, 1194.6759, 1194.6759, 1277.7600, 1296.7419, 1334.9314, 1334.9314, 1448.9645, 1448.9645, 1475.8114, 1487.8091, 1618.1177, 1618.1177, 1633.9124, 1732.1512, 1902.3138, 1994.0560, 2221.2212

25. gi|443492961 **Mass:** 25831 **Score:** 40 **Expect:** 3.4e+003 **Matches:** 8

putative deacetylase [Mycobacterium liflandii 128FXT]

Observed	Mr(expt)	Mr(calc)	ppm	Start	End	Miss	Ions	Peptide
951.6699	950.6626	950.5774	89.7	195	202	1	---	R.QLRKPGPR.Q
1151.7755	1150.7682	1150.7074	52.9	31	41	0	---	R.SVPLSLLVAPR.L
1151.7755	1150.7682	1150.7074	52.9	31	41	0	---	R.SVPLSLLVAPR.L
1266.6686	1265.6613	1265.6153	36.4	51	61	0	---	R.DPHTVDWLAGR.R
1595.9777	1594.9704	1594.8613	68.4	97	110	1	---	R.VMAADRVLEHLGLR.T + Oxidation (M)
1595.9777	1594.9704	1594.8613	68.4	97	110	1	---	R.VMAADRVLEHLGLR.T + Oxidation (M)

PDF-XChange Click to buy NOW! www.docu-track.com

1784.0413	1783.0340	1783.0145	11.0	111	127	1	---	R.TRLFAAPGWVVSPGVVK.A
2284.2476	2283.2403	2283.2124	12.2	128	148	1	---	K.ALPGNGFRLLADYHGISDLVR.K

No match to: 842.5546, 848.5696, 850.5803, 853.5549, 853.5549, 855.0955, 864.5864, 868.5867, 868.5867, 871.0599, 877.0814, 882.5994, 882.5994, 887.0316, 909.0287, 960.9595, 1017.7114, 1019.5801, 1019.7258, 1033.7395, 1036.6012, 1036.6012, 1051.7457, 1051.7457, 1060.1041, 1082.0940, 1098.0653, 1165.7815, 1165.7815, 1194.6759, 1194.6759, 1277.7600, 1296.7419, 1334.9314, 1334.9314, 1448.9645, 1448.9645, 1475.8114, 1487.8091, 1618.1177, 1618.1177, 1633.9124, 1732.1512, 1902.3138, 1994.0560, 2221.2212

26. gi|118616343 **Mass:** 25912 **Score:** 39 **Expect:** 3.9e+003 **Matches:** 8
hypothetical protein MUL_0502 [Mycobacterium ulcerans Agy99]

Observed	Mr(expt)	Mr(calc)	ppm	Start	End	Miss	Ions	Peptide
951.6699	950.6626	950.5774	89.7	195	202	1	---	R.QLRKPGPR.Q
1151.7755	1150.7682	1150.7074	52.9	31	41	0	---	R.SVPLSLLVAPR.L
1151.7755	1150.7682	1150.7074	52.9	31	41	0	---	R.SVPLSLLVAPR.L
1266.6686	1265.6613	1265.6153	36.4	51	61	0	---	R.DPHTVDWLAGR.R
1595.9777	1594.9704	1594.8613	68.4	97	110	1	---	R.VMAADRVLEHLGLR.T + Oxidation (M)
1595.9777	1594.9704	1594.8613	68.4	97	110	1	---	R.VMAADRVLEHLGLR.T + Oxidation (M)
1784.0413	1783.0340	1783.0145	11.0	111	127	1	---	R.TRLFAAPGWVVSPGVVK.A
2284.2476	2283.2403	2283.2124	12.2	128	148	1	---	K.ALPGNGFRLLADYHGISDLVR.K

No match to: 842.5546, 848.5696, 850.5803, 853.5549, 853.5549, 855.0955, 864.5864, 868.5867, 868.5867, 871.0599, 877.0814, 882.5994, 882.5994, 887.0316, 909.0287, 960.9595, 1017.7114, 1019.5801, 1019.7258, 1033.7395, 1036.6012, 1036.6012, 1051.7457, 1051.7457, 1060.1041, 1082.0940, 1098.0653, 1165.7815, 1165.7815, 1194.6759, 1194.6759, 1277.7600, 1296.7419, 1334.9314, 1334.9314, 1448.9645, 1448.9645, 1475.8114, 1487.8091, 1618.1177, 1618.1177, 1633.9124, 1732.1512, 1902.3138, 1994.0560, 2221.2212

27. gi|491438800 **Mass:** 10485 **Score:** 39 **Expect:** 3.9e+003 **Matches:** 5
acetyltransferase [Corynebacterium jeikeium]

Observed	Mr(expt)	Mr(calc)	ppm	Start	End	Miss	Ions	Peptide
1194.6759	1193.6686	1193.6081	50.7	81	90	1	---	K.AFVEKNPDFK.E
1194.6759	1193.6686	1193.6081	50.7	81	90	1	---	K.AFVEKNPDFK.E
1296.7419	1295.7346	1295.6544	62.0	74	85	1	---	K.ASCSAVKAFVEK.N
1475.8114	1474.8041	1474.6841	81.4	38	49	0	---	R.DFNHTVVDPEFR.G
1784.0413	1783.0340	1783.0091	14.0	50	66	0	---	R.GQGLSKPLISEALLETK.E

No match to: 842.5546, 848.5696, 850.5803, 853.5549, 853.5549, 855.0955, 864.5864, 868.5867, 868.5867, 871.0599, 877.0814, 882.5994, 882.5994, 887.0316, 909.0287, 951.6699, 960.9595, 1017.7114, 1019.5801, 1019.7258, 1033.7395, 1036.6012, 1036.6012, 1051.7457, 1051.7457, 1060.1041, 1082.0940, 1098.0653, 1151.7755, 1151.7755, 1165.7815, 1165.7815, 1266.6686, 1277.7600, 1334.9314, 1334.9314, 1448.9645, 1448.9645, 1487.8091, 1595.9777, 1595.9777, 1618.1177, 1618.1177, 1633.9124, 1732.1512, 1902.3138, 1994.0560, 2221.2212, 2284.2476

28. gi|491891625 **Mass:** 32908 **Score:** 39 **Expect:** 4e+003 **Matches:** 6
hypothetical protein [Bacteroides stercoris]

Observed	Mr(expt)	Mr(calc)	ppm	Start	End	Miss	Ions	Peptide
842.5546	841.5473	841.5749	-32.76	289	295	1	---	R.LLLLKSR.-
1194.6759	1193.6686	1193.5533	96.6	52	61	0	---	K.GNTKPMDMIR.I + 2 Oxidation (M)
1194.6759	1193.6686	1193.5533	96.6	52	61	0	11	K.GNTKPMDMIR.I + 2 Oxidation (M)
1266.6686	1265.6613	1265.6299	24.8	134	145	1	---	R.RAGGAGYSCVLR.L
1784.0413	1783.0340	1782.8901	80.7	228	243	1	---	K.GDQFQVSLIKYSGGER.Q
1994.0560	1993.0487	1992.9873	30.8	52	68	1	---	K.GNTKPMDMIRIYEGNVR.A

No match to: 848.5696, 850.5803, 853.5549, 853.5549, 855.0955, 864.5864, 868.5867, 871.0599, 877.0814, 882.5994, 882.5994, 887.0316, 909.0287, 951.6699, 960.9595, 1017.7114, 1019.5801, 1019.7258, 1033.7395, 1036.6012, 1036.6012, 1051.7457, 1051.7457, 1060.1041, 1082.0940, 1098.0653, 1151.7755, 1151.7755, 1165.7815, 1165.7815, 1277.7600, 1296.7419, 1334.9314, 1334.9314, 1448.9645, 1448.9645, 1475.8114, 1487.8091, 1595.9777, 1595.9777, 1618.1177, 1618.1177, 1633.9124, 1732.1512, 1902.3138, 2221.2212, 2284.2476

29. gi|547517138 **Mass:** 32936 **Score:** 39 **Expect:** 4e+003 **Matches:** 6
uncharacterized protein [Bacteroides stercoris CAG:120]

Observed	Mr(expt)	Mr(calc)	ppm	Start	End	Miss	Ions	Peptide
842.5546	841.5473	841.5749	-32.76	289	295	1	---	R.LLLLKSR.-
1194.6759	1193.6686	1193.5533	96.6	52	61	0	---	K.GNTKPMDMIR.I + 2 Oxidation (M)
1194.6759	1193.6686	1193.5533	96.6	52	61	0	11	K.GNTKPMDMIR.I + 2 Oxidation (M)
1266.6686	1265.6613	1265.6299	24.8	134	145	1	---	R.RAGGAGYSCVLR.L
1784.0413	1783.0340	1782.8901	80.7	228	243	1	---	K.GDQFQVSLIKYSGGER.Q
1994.0560	1993.0487	1992.9873	30.8	52	68	1	---	K.GNTKPMDMIRIYEGNVR.A

No match to: 848.5696, 850.5803, 853.5549, 853.5549, 855.0955, 864.5864, 868.5867, 871.0599, 877.0814, 882.5994, 882.5994, 887.0316, 909.0287, 951.6699, 960.9595, 1017.7114, 1019.5801, 1019.7258, 1033.7395, 1036.6012, 1036.6012, 1051.7457, 1051.7457, 1060.1041, 1082.0940, 1098.0653, 1151.7755, 1151.7755, 1165.7815, 1165.7815, 1277.7600, 1296.7419, 1334.9314, 1334.9314, 1448.9645, 1448.9645, 1475.8114, 1487.8091, 1595.9777, 1595.9777, 1618.1177, 1618.1177, 1633.9124, 1732.1512, 1902.3138, 2221.2212, 2284.2476

30. gi|517368986 **Mass:** 14244 **Score:** 39 **Expect:** 4.1e+003 **Matches:** 7
30S ribosomal protein S13 [Streptomyces sp. LaPpAH-108]

Observed	Mr(expt)	Mr(calc)	ppm	Start	End	Miss	Ions	Peptide
842.5546	841.5473	841.4658	96.9	4	11	0	---	R.VSGVDIPR.E
882.5994	881.5921	881.5195	82.3	95	102	1	---	R.GLPVRGQR.T
882.5994	881.5922	881.5195	82.4	95	102	1	---	R.GLPVRGQR.T
1194.6759	1193.6686	1193.5863	69.0	82	91	0	---	K.VEIGCYQGLR.H
1194.6759	1193.6686	1193.5863	69.0	82	91	0	---	K.VEIGCYQGLR.H
1487.8091	1486.8018	1486.7463	37.3	82	93	1	---	K.VEIGCYQGLRHR.R
2221.2212	2220.2139	2220.1274	39.0	47	65	1	---	R.DLSEEQLVAIREYVDSNIK.T

No match to: 848.5696, 850.5803, 853.5549, 853.5549, 855.0955, 864.5864, 868.5867, 871.0599, 877.0814, 887.0316, 909.0287, 951.6699, 960.9595, 1017.7114, 1019.5801, 1019.7258, 1033.7395, 1036.6012, 1036.6012, 1051.7457, 1051.7457, 1060.1041, 1082.0940, 1098.0653, 1151.7755, 1151.7755, 1165.7815, 1165.7815, 1266.6686, 1277.7600, 1296.7419, 1334.9314, 1334.9314, 1448.9645, 1448.9645, 1475.8114, 1595.9777, 1595.9777, 1618.1177, 1618.1177, 1633.9124, 1732.1512, 1784.0413, 1902.3138, 1994.0560, 2284.2476

31. gi|516399639 **Mass:** 5928 **Score:** 39 **Expect:** 4.1e+003 **Matches:** 5
integrase [Vibrio vulnificus]

Observed	Mr(expt)	Mr(calc)	ppm	Start	End	Miss	Ions	Peptide
1036.6012	1035.5939	1035.6077	-13.26	6	14	1	---	K.ATLIYARTK.N
1036.6012	1035.5939	1035.6077	-13.26	4	12	1	---	R.TKATLIYAR.T
1277.7600	1276.7527	1276.6663	67.7	34	44	0	---	R.YLGVELEDALR.L
1448.9645	1447.9572	1447.8623	65.5	15	27	1	---	K.NIRAVQILLGHSK.V
1448.9645	1447.9572	1447.8623	65.6	15	27	1	---	K.NIRAVQILLGHSK.V

No match to: 842.5546, 848.5696, 850.5803, 853.5549, 853.5549, 855.0955, 864.5864, 868.5867, 868.5867, 871.0599, 877.0814, 882.5994, 882.5994, 887.0316, 909.0287, 951.6699, 960.9595, 1017.7114, 1019.5801, 1019.7258, 1033.7395, 1051.7457, 1051.7457, 1060.1041, 1082.0940, 1098.0653, 1151.7755, 1151.7755, 1165.7815, 1165.7815, 1194.6759, 1194.6759, 1266.6686, 1296.7419,

PDF-XChange Click to buy NOW! www.docu-track.com

PDF-XChange Click to buy NOW! www.docu-track.com

1334.9314, 1334.9314, 1475.8114, 1487.8091, 1595.9777, 1595.9777, 1618.1177, 1618.1177, 1633.9124, 1732.1512, 1784.0413, 1902.3138, 1994.0560, 2221.2212, 2284.2476

32. gi|479181342 **Mass:** 3438 **Score:** 39 **Expect:** 4.2e+003 **Matches:** 3
Helix-turn-helix domain of resolvase. [Enterococcus sp. 7L76]

Observed	Mr(expt)	Mr(calc)	ppm	Start	End	Miss	Ions	Peptide
1266.6686	1265.6613	1265.7707	-86.40	2	13	1	---	M.LKQGIPVAEIAK.E
1277.7600	1276.7527	1276.6775	58.9	14	24	0	---	K.ENGISRPTIYK.I
2284.2476	2283.2403	2283.2586	-8.02	4	24	1	---	K.QGIPVAEIAKENGISRPTIYK.I

No match to: 842.5546, 848.5696, 850.5803, 853.5549, 853.5549, 855.0955, 864.5864, 868.5867, 868.5867, 871.0599, 877.0814, 882.5994, 882.5994, 887.0316, 909.0287, 951.6699, 960.9595, 1017.7114, 1019.5801, 1019.7258, 1033.7395, 1036.6012, 1036.6012, 1051.7457, 1051.7457, 1060.1041, 1082.0940, 1098.0653, 1151.7755, 1151.7755, 1165.7815, 1165.7815, 1194.6759, 1194.6759, 1296.7419, 1334.9314, 1334.9314, 1448.9645, 1448.9645, 1475.8114, 1487.8091, 1595.9777, 1595.9777, 1618.1177, 1618.1177, 1633.9124, 1732.1512, 1784.0413, 1902.3138, 1994.0560, 2221.2212

33. gi|491349492 **Mass:** 31061 **Score:** 39 **Expect:** 4.3e+003 **Matches:** 6
hypothetical protein [Gordonia sputi]

Observed	Mr(expt)	Mr(calc)	ppm	Start	End	Miss	Ions	Peptide
1036.6012	1035.5939	1035.5317	60.1	113	120	1	---	R.MLRVQMAR.T + 2 Oxidation (M)
1036.6012	1035.5939	1035.5317	60.1	113	120	1	---	R.MLRVQMAR.T + 2 Oxidation (M)
1194.6759	1193.6686	1193.6418	22.5	243	251	1	---	R.RIHQLWADR.Q
1194.6759	1193.6686	1193.6227	38.5	107	115	1	11	R.EFDLVRMLR.V + Oxidation (M)
1487.8091	1486.8018	1486.7449	38.3	211	222	1	---	R.EIEEMAEVRLPR.Q + Oxidation (M)
2284.2476	2283.2403	2283.0994	61.7	95	112	1	---	R.TQPYELVSWMDREFDLVR.M

No match to: 842.5546, 848.5696, 850.5803, 853.5549, 853.5549, 855.0955, 864.5864, 868.5867, 868.5867, 871.0599, 877.0814, 882.5994, 882.5994, 887.0316, 909.0287, 951.6699, 960.9595, 1017.7114, 1019.5801, 1019.7258, 1033.7395, 1051.7457, 1051.7457, 1060.1041, 1082.0940, 1098.0653, 1151.7755, 1151.7755, 1165.7815, 1165.7815, 1266.6686, 1277.7600, 1296.7419, 1334.9314, 1334.9314, 1448.9645, 1448.9645, 1475.8114, 1595.9777, 1595.9777, 1618.1177, 1618.1177, 1633.9124, 1732.1512, 1784.0413, 1902.3138, 1994.0560, 2221.2212

34. gi|493388713 **Mass:** 17048 **Score:** 39 **Expect:** 4.4e+003 **Matches:** 5
MarR family transcriptional regulator [Streptomyces tsukubaensis]

Observed	Mr(expt)	Mr(calc)	ppm	Start	End	Miss	Ions	Peptide
842.5546	841.5473	841.5134	40.4	141	148	1	---	K.KALGQVAR.A
848.5696	847.5623	847.4916	83.5	24	30	0	---	R.VLFSVQR.E
1296.7419	1295.7346	1295.6946	30.9	62	73	1	---	R.ASELARLSGQHK.Q
1475.8114	1474.8041	1474.8368	-22.17	48	61	0	---	R.SGAVLAHLRPEGIR.A
1994.0560	1993.0487	1993.0593	-5.29	68	85	1	---	R.LSGQHKQVVGTLVDDLER.L

No match to: 850.5803, 853.5549, 853.5549, 855.0955, 864.5864, 868.5867, 868.5867, 871.0599, 877.0814, 882.5994, 882.5994, 887.0316, 909.0287, 951.6699, 960.9595, 1017.7114, 1019.5801, 1019.7258, 1033.7395, 1036.6012, 1036.6012, 1051.7457, 1051.7457, 1060.1041, 1082.0940, 1098.0653, 1151.7755, 1151.7755, 1165.7815, 1165.7815, 1194.6759, 1194.6759, 1266.6686, 1277.7600, 1334.9314, 1334.9314, 1448.9645, 1448.9645, 1487.8091, 1595.9777, 1595.9777, 1618.1177, 1618.1177, 1633.9124, 1732.1512, 1784.0413, 1902.3138, 2221.2212, 2284.2476

35. gi|551036884 **Mass:** 33460 **Score:** 39 **Expect:** 4.5e+003 **Matches:** 8
hypothetical protein [Lachnospiraceae bacterium NK4A136]

Observed	Mr(expt)	Mr(calc)	ppm	Start	End	Miss	Ions	Peptide
864.5864	863.5791	863.5116	78.2	64	70	1	---	R.LIKYAEK.M
1036.6012	1035.5939	1035.5197	71.7	54	63	0	---	K.TISVTSSGER.L
1036.6012	1035.5939	1035.5197	71.7	54	63	0	---	K.TISVTSSGER.L
1151.7755	1150.7682	1150.6598	94.2	2	11	0	---	M.TITQLSTFLK.I
1151.7755	1150.7682	1150.6598	94.3	2	11	0	---	M.TITQLSTFLK.I
1266.6686	1265.6613	1265.6842	-18.07	105	114	1	---	R.FPKILMEFNK.D
1296.7419	1295.7346	1295.6543	62.0	67	76	1	---	K.YAEKMLQLER.E + Oxidation (M)
2284.2476	2283.2403	2283.1093	57.4	138	156	1	---	K.GELDMVYTLNPLFEDDRIK.I + Oxidation (M)

No match to: 842.5546, 848.5696, 850.5803, 853.5549, 853.5549, 855.0955, 868.5867, 868.5867, 871.0599, 877.0814, 882.5994, 882.5994, 887.0316, 909.0287, 951.6699, 960.9595, 1017.7114, 1019.5801, 1019.7258, 1033.7395, 1051.7457, 1051.7457, 1060.1041, 1082.0940, 1098.0653, 1165.7815, 1165.7815, 1194.6759, 1194.6759, 1277.7600, 1334.9314, 1334.9314, 1448.9645, 1448.9645, 1475.8114, 1487.8091, 1595.9777, 1595.9777, 1618.1177, 1618.1177, 1633.9124, 1732.1512, 1784.0413, 1902.3138, 1994.0560, 2221.2212

36. gi|320333797 **Mass:** 39533 **Score:** 39 **Expect:** 4.5e+003 **Matches:** 7
phospho-2-dehydro-3-deoxyheptonate aldolase [Deinococcus maricopensis DSM 21211]

Observed	Mr(expt)	Mr(calc)	ppm	Start	End	Miss	Ions	Peptide
882.5994	881.5921	881.5586	38.1	302	309	0	---	R.DLLIPLAK.A
882.5994	881.5922	881.5586	38.1	302	309	0	---	R.DLLIPLAK.A
1019.5801	1018.5728	1018.5229	49.0	1	8	1	---	-.MTRIDDLR.R
1194.6759	1193.6686	1193.5937	62.8	346	355	0	---	K.FMAALEPMLR.A + Oxidation (M)
1194.6759	1193.6686	1193.5937	62.8	346	355	0	13	K.FMAALEPMLR.A + Oxidation (M)
1266.6686	1265.6613	1265.7819	-95.29	220	231	1	---	R.EVGRAGKPVLLK.R
1633.9124	1632.9051	1632.8480	35.0	346	359	1	---	K.FMAALEPMLRAPVR.A + 2 Oxidation (M)

No match to: 842.5546, 848.5696, 850.5803, 853.5549, 853.5549, 855.0955, 864.5864, 868.5867, 868.5867, 871.0599, 877.0814, 887.0316, 909.0287, 951.6699, 960.9595, 1017.7114, 1019.7258, 1033.7395, 1036.6012, 1036.6012, 1051.7457, 1051.7457, 1060.1041, 1082.0940, 1098.0653, 1151.7755, 1151.7755, 1165.7815, 1165.7815, 1277.7600, 1296.7419, 1334.9314, 1334.9314, 1448.9645, 1448.9645, 1475.8114, 1487.8091, 1595.9777, 1595.9777, 1618.1177, 1618.1177, 1732.1512, 1784.0413, 1902.3138, 1994.0560, 2221.2212, 2284.2476

37. gi|409039618 **Mass:** 29114 **Score:** 38 **Expect:** 4.8e+003 **Matches:** 7
hypothetical protein PHACADRAFT_201965 [Phanerochaete carnosa HHB-10118-sp]

Observed	Mr(expt)	Mr(calc)	ppm	Start	End	Miss	Ions	Peptide
842.5546	841.5473	841.5385	10.5	75	81	1	---	R.LRLELAK.M
848.5696	847.5623	847.4837	92.8	77	83	1	---	R.LELAKMK.S + Oxidation (M)
1036.6012	1035.5939	1035.5138	77.4	195	202	0	---	R.NYFTNHIK.E
1036.6012	1035.5939	1035.5138	77.4	195	202	0	---	R.NYFTNHIK.E
1487.8091	1486.8018	1486.7416	40.5	203	214	1	---	K.ESLIKYTPDHER.I
1784.0413	1783.0340	1782.9992	19.5	160	175	1	---	R.TKVLVHASELFEVVGR.R
2284.2476	2283.2403	2283.0388	88.3	177	194	0	---	R.SPLFLYMGQWHVTCCTQR.N

No match to: 850.5803, 853.5549, 853.5549, 855.0955, 864.5864, 868.5867, 868.5867, 871.0599, 877.0814, 882.5994, 882.5994, 887.0316, 909.0287, 951.6699, 960.9595, 1017.7114, 1019.5801, 1019.7258, 1033.7395, 1051.7457, 1051.7457, 1060.1041, 1082.0940, 1098.0653, 1151.7755, 1151.7755, 1165.7815, 1165.7815, 1194.6759, 1194.6759, 1266.6686, 1277.7600, 1296.7419, 1334.9314, 1334.9314, 1448.9645, 1448.9645, 1475.8114, 1595.9777, 1595.9777, 1618.1177, 1618.1177, 1633.9124, 1732.1512, 1902.3138, 1994.0560, 2221.2212

38. gi|187251559 **Mass:** 20917 **Score:** 38 **Expect:** 4.8e+003 **Matches:** 6
crossover junction endodeoxyribonuclease RuvC [Elusimicrobium minutum Pei191]

Observed	Mr(expt)	Mr(calc)	ppm	Start	End	Miss	Ions	Peptide

PDF-XChange Click to buy NOW! www.docu-track.com

PDF-XChange Click to buy NOW! www.docu-track.com

848.5696	847.5623	847.4837	92.8	132 - 138	0	---	R.MVQLTLK.L + Oxidation (M)
1036.6012	1035.5939	1035.5019	88.9	116 - 126	0	---	K.SVICGSGTAGK.A
1036.6012	1035.5939	1035.5019	88.9	116 - 126	0	---	K.SVICGSGTAGK.A
1784.0413	1783.0340	1782.9112	68.9	2 - 18	0	---	M.DNNALTTVLGIDPGLDR.T
1994.0560	1993.0487	1993.0156	16.6	45 - 61	1	---	K.DSALPERLEYIFNEIGK.L
2284.2476	2283.2403	2283.1067	58.5	62 - 80	0	---	K.LAEAYKPNHVAMEEMFFLK.R + Oxidation (M)

No match to: 842.5546, 850.5803, 853.5549, 853.5549, 855.0955, 864.5864, 868.5867, 868.5867, 871.0599, 877.0814, 882.5994, 882.5994, 887.0316, 909.0287, 951.6699, 960.9595, 1017.7114, 1019.5801, 1019.7258, 1033.7395, 1051.7457, 1051.7457, 1060.1041, 1082.0940, 1098.0653, 1151.7755, 1151.7755, 1165.7815, 1165.7815, 1194.6759, 1194.6759, 1266.6686, 1277.7600, 1296.7419, 1334.9314, 1334.9314, 1448.9645, 1448.9645, 1475.8114, 1487.8091, 1595.9777, 1595.9777, 1618.1177, 1618.1177, 1633.9124, 1732.1512, 1902.3138, 2221.2212

39. gi|261260695 **Mass:** 10351 **Score:** 38 **Expect:** 5e+003 **Matches:** 5
Yclm [Bacillus amyloliquefaciens]

Observed	Mr(expt)	Mr(calc)	ppm	Start - End	Miss	Ions	Peptide
853.5549	852.5476	852.4857	72.6	51 - 57	0	---	K.LIAAYFR.R
853.5549	852.5477	852.4857	72.6	51 - 57	0	---	K.LIAAYFR.R
1194.6759	1193.6686	1193.6292	33.0	20 - 30	0	---	R.SDLFALLNSSK.A
1194.6759	1193.6686	1193.6292	33.0	20 - 30	0	11	R.SDLFALLNSSK.A
1994.0560	1993.0487	1993.0632	-7.27	1 - 17	1	---	-.YAAIARELNLDFSIIER.I

No match to: 842.5546, 848.5696, 850.5803, 855.0955, 864.5864, 868.5867, 868.5867, 871.0599, 877.0814, 882.5994, 882.5994, 887.0316, 909.0287, 951.6699, 960.9595, 1017.7114, 1019.5801, 1019.7258, 1033.7395, 1036.6012, 1036.6012, 1051.7457, 1051.7457, 1060.1041, 1082.0940, 1098.0653, 1151.7755, 1151.7755, 1165.7815, 1165.7815, 1266.6686, 1277.7600, 1296.7419, 1334.9314, 1334.9314, 1448.9645, 1448.9645, 1475.8114, 1487.8091, 1595.9777, 1595.9777, 1618.1177, 1618.1177, 1633.9124, 1732.1512, 1784.0413, 1902.3138, 2221.2212, 2284.2476

40. gi|517051098 **Mass:** 27685 **Score:** 38 **Expect:** 5.4e+003 **Matches:** 6
transcriptional regulator [Rhizobium sp. BR816]

Observed	Mr(expt)	Mr(calc)	ppm	Start - End	Miss	Ions	Peptide
1194.6759	1193.6686	1193.5539	96.1	98 - 106	0	---	R.FPECEPLFR.S
1194.6759	1193.6686	1193.5539	96.1	98 - 106	0	13	R.FPECEPLFR.S
1487.8091	1486.8018	1486.7139	59.1	96 - 106	1	---	R.HRFPECEPLFR.S
1595.9777	1594.9704	1594.8943	47.7	81 - 95	0	---	R.LALGGVRPVEWATAR.H
1595.9777	1594.9704	1594.8943	47.7	81 - 95	0	---	R.LALGGVRPVEWATAR.H
1994.0560	1993.0487	1993.0632	-7.29	198 - 214	1	---	K.SYWEIATILGISERTVR.F

No match to: 842.5546, 848.5696, 850.5803, 853.5549, 853.5549, 855.0955, 864.5864, 868.5867, 868.5867, 871.0599, 877.0814, 882.5994, 882.5994, 887.0316, 909.0287, 951.6699, 960.9595, 1017.7114, 1019.5801, 1019.7258, 1033.7395, 1036.6012, 1036.6012, 1051.7457, 1051.7457, 1060.1041, 1082.0940, 1098.0653, 1151.7755, 1151.7755, 1165.7815, 1165.7815, 1266.6686, 1277.7600, 1296.7419, 1334.9314, 1334.9314, 1448.9645, 1448.9645, 1475.8114, 1618.1177, 1618.1177, 1633.9124, 1732.1512, 1784.0413, 1902.3138, 2221.2212, 2284.2476

41. gi|444720581 **Mass:** 100358 **Score:** 38 **Expect:** 5.5e+003 **Matches:** 10
General transcription factor II-I [Tupaia chinensis]

Observed	Mr(expt)	Mr(calc)	ppm	Start - End	Miss	Ions	Peptide
842.5546	841.5473	841.5385	10.5	410 - 416	1	---	R.ILLAKER.I
848.5696	847.5623	847.5167	53.8	236 - 243	0	---	K.AGISFIIK.S
1036.6012	1035.5939	1035.5131	78.0	151 - 159	1	---	K.MKSTTQANR.M
1036.6012	1035.5939	1035.5131	78.0	151 - 159	1	---	K.MKSTTQANR.M
1266.6686	1265.6613	1265.6768	-12.22	358 - 368	1	---	R.EFNFAQAIKAK.G
1487.8091	1486.8018	1486.8395	-25.36	185 - 198	1	---	K.ALGKSTVVPVPYEK.M
1784.0413	1783.0340	1782.9127	68.0	810 - 825	1	---	K.FGEAIGMGFPVKVPYR.K + Oxidation (M)
1994.0560	1993.0487	1992.9561	46.5	252 - 270	0	---	K.TEPTEDSGISLEMAAVTVK.E + Oxidation (M)
2221.2212	2220.2139	2220.1195	42.5	250 - 270	1	---	K.VKTEPTEDSGISLEMAAVTVK.E + Oxidation (M)
2284.2476	2283.2403	2283.1998	17.7	645 - 664	1	---	K.VPEIEVTVEEAWNAKITDLK.Q

No match to: 850.5803, 853.5549, 853.5549, 855.0955, 864.5864, 868.5867, 868.5867, 871.0599, 877.0814, 882.5994, 882.5994, 887.0316, 909.0287, 951.6699, 960.9595, 1017.7114, 1019.5801, 1019.7258, 1033.7395, 1051.7457, 1051.7457, 1060.1041, 1082.0940, 1098.0653, 1151.7755, 1151.7755, 1165.7815, 1165.7815, 1194.6759, 1194.6759, 1277.7600, 1296.7419, 1334.9314, 1334.9314, 1448.9645, 1448.9645, 1475.8114, 1595.9777, 1595.9777, 1618.1177, 1618.1177, 1633.9124, 1732.1512, 1902.3138

42. gi|71039991 **Mass:** 32224 **Score:** 38 **Expect:** 6e+003 **Matches:** 9
conserved ATPase domain protein [Macropis nuda]

Observed	Mr(expt)	Mr(calc)	ppm	Start - End	Miss	Ions	Peptide
850.5803	849.5730	849.5072	77.4	143 - 149	1	---	K.FLRVSTK.I
1019.5801	1018.5728	1018.5771	-4.17	81 - 90	1	---	R.LSRSSALASK.A
1151.7755	1150.7682	1150.6532	99.9	204 - 213	1	---	K.RMFVLAASIK.A + Oxidation (M)
1151.7755	1150.7682	1150.6532	99.9	204 - 213	1	---	K.RMFVLAASIK.A + Oxidation (M)
1165.7815	1164.7742	1164.6979	65.6	253 - 262	1	---	K.LSHEVLLRAK.Q
1165.7815	1164.7742	1164.6979	65.6	253 - 262	1	---	K.LSHEVLLRAK.Q
1194.6759	1193.6686	1193.6404	23.6	109 - 119	0	---	R.LPDIHNTVTGK.T
1194.6759	1193.6686	1193.6404	23.6	109 - 119	0	---	R.LPDIHNTVTGK.T
1475.8114	1474.8041	1474.8144	-6.94	263 - 276	1	---	K.QIGFSDKQIAAAVK.S

No match to: 842.5546, 848.5696, 853.5549, 853.5549, 855.0955, 864.5864, 868.5867, 868.5867, 871.0599, 877.0814, 882.5994, 882.5994, 887.0316, 909.0287, 951.6699, 960.9595, 1017.7114, 1019.7258, 1033.7395, 1036.6012, 1036.6012, 1051.7457, 1051.7457, 1060.1041, 1082.0940, 1098.0653, 1266.6686, 1277.7600, 1296.7419, 1334.9314, 1334.9314, 1448.9645, 1448.9645, 1487.8091, 1595.9777, 1595.9777, 1618.1177, 1618.1177, 1633.9124, 1732.1512, 1784.0413, 1902.3138, 1994.0560, 2221.2212, 2284.2476

43. gi|490661404 **Mass:** 4181 **Score:** 38 **Expect:** 6e+003 **Matches:** 4
hypothetical protein [Burkholderia pseudomallei]

Observed	Mr(expt)	Mr(calc)	ppm	Start - End	Miss	Ions	Peptide
1448.9645	1447.9572	1447.8372	82.9	2 - 15	1	---	M.RGPLEARPVGLAGR.R
1448.9645	1447.9572	1447.8372	82.9	3 - 16	1	---	R.GPLEARPVGLAGRR.D
1595.9777	1594.9704	1594.8726	61.3	1 - 15	1	---	-.MRGPLEARPVGLAGR.R + Oxidation (M)
1595.9777	1594.9704	1594.8726	61.4	1 - 15	1	---	-.MRGPLEARPVGLAGR.R + Oxidation (M)

No match to: 842.5546, 848.5696, 850.5803, 853.5549, 853.5549, 855.0955, 864.5864, 868.5867, 868.5867, 871.0599, 877.0814, 882.5994, 882.5994, 887.0316, 909.0287, 951.6699, 960.9595, 1017.7114, 1019.5801, 1019.7258, 1033.7395, 1036.6012, 1036.6012, 1051.7457, 1051.7457, 1060.1041, 1082.0940, 1098.0653, 1151.7755, 1151.7755, 1165.7815, 1165.7815, 1194.6759, 1194.6759, 1266.6686, 1277.7600, 1296.7419, 1334.9314, 1334.9314, 1475.8114, 1487.8091, 1618.1177, 1618.1177, 1633.9124, 1732.1512, 1784.0413, 1902.3138, 1994.0560, 2221.2212, 2284.2476

44. gi|489236824 **Mass:** 65949 **Score:** 37 **Expect:** 6.2e+003 **Matches:** 11
multidrug ABC transporter ATP-binding protein [Gemella haemolysans]

Observed	Mr(expt)	Mr(calc)	ppm	Start - End	Miss	Ions	Peptide
848.5696	847.5623	847.4916	83.5	481 - 487	0	---	R.QLLTFAR.T
882.5994	881.5921	881.5083	95.1	525 - 532	0	---	R.TTIAIAHR.L
882.5994	881.5922	881.5083	95.1	525 - 532	0	---	R.TTIAIAHR.L

PDF-XChange Click to buy NOW! www.docu-track.com

1036.6012	1035.5939	1035.4985	92.1	96 - 104	0	---	R.NDSFANLQK.L
1036.6012	1035.5939	1035.4985	92.1	96 - 104	0	---	R.NDSFANLQK.L
1165.7815	1164.7742	1164.6727	87.1	86 - 95	1	---	K.VAHSIVRDLR.N
1165.7815	1164.7742	1164.6727	87.1	86 - 95	1	---	K.VAHSIVRDLR.N
1296.7419	1295.7346	1295.6366	75.7	569 - 579	0	---	K.MYQLQAGMLNK.-
1902.3138	1901.3065	1901.1172	99.6	238 - 254	1	---	K.SLKVDSLLLRPAMALFK.V
1994.0560	1993.0487	1992.9251	62.0	96 - 112	1	---	R.NDSFANLQKLGMSYFDK.T + Oxidation (M)
2221.2212	2220.2139	2220.1208	41.9	201 - 219	1	---	K.LSESIEGMKIIQAFNQEQR.L

No match to: 842.5546, 850.5803, 853.5549, 853.5549, 855.0955, 864.5864, 868.5867, 868.5867, 871.0599, 877.0814, 887.0316, 909.0287, 951.6699, 960.9595, 1017.7114, 1019.5801, 1019.7258, 1033.7395, 1051.7457, 1051.7457, 1060.1041, 1082.0940, 1098.0653, 1151.7755, 1151.7755, 1194.6759, 1194.6759, 1266.6686, 1277.7600, 1334.9314, 1334.9314, 1448.9645, 1448.9645, 1475.8114, 1487.8091, 1595.9777, 1595.9777, 1618.1177, 1618.1177, 1633.9124, 1732.1512, 1784.0413, 2284.2476

45. gi|547230974 **Mass:** 17853 **Score:** 37 **Expect:** 6.4e+003 **Matches:** 6
transposase [Bacteroides intestinalis CAG:564]

Observed	Mr(expt)	Mr(calc)	ppm	Start - End	Miss	Ions	Peptide
1019.5801	1018.5728	1018.4728	98.2	51 - 58	1	---	K.KGYYMAMR.L
1194.6759	1193.6686	1193.5676	84.6	113 - 124	0	---	R.ATNAAAESFNAK.I
1194.6759	1193.6686	1193.5676	84.6	113 - 124	0	---	R.ATNAAAESFNAK.I
1475.8114	1474.8041	1474.7317	49.1	2 - 14	1	---	M.YHAPVFANGDTRK.Q
1994.0560	1993.0487	1992.9008	74.2	52 - 67	1	---	K.GYYMAMRLGSIYHQCK.F + Oxidation (M)
2221.2212	2220.2139	2220.1803	15.1	94 - 111	1	---	R.VARSIQTHYLNIINFFER.R

No match to: 842.5546, 848.5696, 850.5803, 853.5549, 853.5549, 855.0955, 864.5864, 868.5867, 868.5867, 871.0599, 877.0814, 882.5994, 882.5994, 887.0316, 909.0287, 951.6699, 960.9595, 1017.7114, 1019.7258, 1033.7395, 1036.6012, 1036.6012, 1051.7457, 1051.7457, 1060.1041, 1082.0940, 1098.0653, 1151.7755, 1151.7755, 1165.7815, 1165.7815, 1266.6686, 1277.7600, 1296.7419, 1334.9314, 1334.9314, 1448.9645, 1448.9645, 1487.8091, 1595.9777, 1595.9777, 1618.1177, 1618.1177, 1633.9124, 1732.1512, 1784.0413, 1902.3138, 2284.2476

46. gi|494229523 **Mass:** 4528 **Score:** 37 **Expect:** 6.6e+003 **Matches:** 6
hypothetical protein [Flavobacterium frigoris]

Observed	Mr(expt)	Mr(calc)	ppm	Start - End	Miss	Ions	Peptide
1051.7457	1050.7384	1050.6437	90.1	2 - 12	0	---	M.PLPSGLGILGK.Q
1051.7457	1050.7385	1050.6437	90.2	2 - 12	0	---	M.PLPSGLGILGK.Q
1448.9645	1447.9572	1447.8875	48.2	2 - 15	1	---	M.PLPSGLGILGKQLR.F
1448.9645	1447.9572	1447.8875	48.2	2 - 15	1	---	M.PLPSGLGILGKQLR.F
1595.9777	1594.9704	1594.9229	29.8	1 - 15	1	---	-.MPLPSGLGILGKQLR.F + Oxidation (M)
1595.9777	1594.9704	1594.9229	29.8	1 - 15	1	---	-.MPLPSGLGILGKQLR.F + Oxidation (M)

No match to: 842.5546, 848.5696, 850.5803, 853.5549, 853.5549, 855.0955, 864.5864, 868.5867, 868.5867, 871.0599, 877.0814, 882.5994, 882.5994, 887.0316, 909.0287, 951.6699, 960.9595, 1017.7114, 1019.5801, 1019.7258, 1033.7395, 1036.6012, 1036.6012, 1060.1041, 1082.0940, 1098.0653, 1151.7755, 1151.7755, 1165.7815, 1165.7815, 1194.6759, 1194.6759, 1266.6686, 1277.7600, 1296.7419, 1334.9314, 1334.9314, 1475.8114, 1487.8091, 1618.1177, 1618.1177, 1633.9124, 1732.1512, 1784.0413, 1902.3138, 1994.0560, 2221.2212, 2284.2476

47. gi|28493757 **Mass:** 41909 **Score:** 37 **Expect:** 6.6e+003 **Matches:** 6
pyruvate dehydrogenase E1 component alpha subunit [Tropheryma whipplei str. Twist]

Observed	Mr(expt)	Mr(calc)	ppm	Start - End	Miss	Ions	Peptide
1036.6012	1035.5939	1035.5502	42.3	226 - 235	0	---	R.GYGFGIPGIR.I
1036.6012	1035.5939	1035.5502	42.3	226 - 235	0	---	R.GYGFGIPGIR.I
1194.6759	1193.6686	1193.6921	-19.63	42 - 50	1	---	R.FYRDIILVR.Q
1194.6759	1193.6686	1193.6921	-19.63	42 - 50	1	18	R.FYRDIILVR.Q
1475.8114	1474.8041	1474.7780	17.7	236 - 249	0	---	R.IDGNDVIAAYAVVR.E
1487.8091	1486.8018	1486.8620	-40.46	318 - 330	1	---	R.IRADIQLSVIGFR.N

No match to: 842.5546, 848.5696, 850.5803, 853.5549, 853.5549, 855.0955, 864.5864, 868.5867, 868.5867, 871.0599, 877.0814, 882.5994, 882.5994, 887.0316, 909.0287, 951.6699, 960.9595, 1017.7114, 1019.5801, 1019.7258, 1033.7395, 1051.7457, 1051.7457, 1060.1041, 1082.0940, 1098.0653, 1151.7755, 1151.7755, 1165.7815, 1165.7815, 1266.6686, 1277.7600, 1296.7419, 1334.9314, 1334.9314, 1448.9645, 1448.9645, 1595.9777, 1595.9777, 1618.1177, 1618.1177, 1633.9124, 1732.1512, 1784.0413, 1902.3138, 1994.0560, 2221.2212, 2284.2476

48. gi|517826373 **Mass:** 12209 **Score:** 37 **Expect:** 6.7e+003 **Matches:** 4
hypothetical protein [Hirschia maritima]

Observed	Mr(expt)	Mr(calc)	ppm	Start - End	Miss	Ions	Peptide
842.5546	841.5473	841.5385	10.5	46 - 53	1	---	R.AILQAKAK.T
1194.6759	1193.6686	1193.5652	86.7	26 - 34	0	---	K.ENWICGFIR.S
1194.6759	1193.6686	1193.5652	86.7	26 - 34	0	11	K.ENWICGFIR.S
2284.2476	2283.2403	2283.1980	18.6	82 - 100	1	---	R.LNLFEYIKSFGTGFIFYPK.F

No match to: 848.5696, 850.5803, 853.5549, 853.5549, 855.0955, 864.5864, 868.5867, 868.5867, 871.0599, 877.0814, 882.5994, 882.5994, 887.0316, 909.0287, 951.6699, 960.9595, 1017.7114, 1019.5801, 1019.7258, 1033.7395, 1036.6012, 1036.6012, 1051.7457, 1051.7457, 1060.1041, 1082.0940, 1098.0653, 1151.7755, 1151.7755, 1165.7815, 1165.7815, 1266.6686, 1277.7600, 1296.7419, 1334.9314, 1334.9314, 1448.9645, 1448.9645, 1475.8114, 1487.8091, 1595.9777, 1595.9777, 1618.1177, 1618.1177, 1633.9124, 1732.1512, 1784.0413, 1902.3138, 1994.0560, 2221.2212

49. gi|446609078 **Mass:** 29288 **Score:** 37 **Expect:** 6.7e+003 **Matches:** 6
prephenate dehydratase, partial [Streptococcus sp. GMD1S]

Observed	Mr(expt)	Mr(calc)	ppm	Start - End	Miss	Ions	Peptide
842.5546	841.5473	841.5021	53.7	223 - 229	0	---	K.IESRPLK.T
1036.6012	1035.5939	1035.5787	14.7	1 - 9	1	---	-.MKIAYLGPK.G + Oxidation (M)
1036.6012	1035.5939	1035.5787	14.7	1 - 9	1	3	-.MKIAYLGPK.G + Oxidation (M)
1296.7419	1295.7346	1295.6986	27.8	98 - 109	0	---	K.IFSHPQALAQGK.K
1784.0413	1783.0340	1782.8941	78.5	133 - 148	0	---	R.FISEHPDQPYAAIAPK.S
2284.2476	2283.2403	2283.2164	10.5	3 - 23	1	---	K.IAYLGPKGSFSHHVVQTAFPK.E

No match to: 848.5696, 850.5803, 853.5549, 853.5549, 855.0955, 864.5864, 868.5867, 868.5867, 871.0599, 877.0814, 882.5994, 882.5994, 887.0316, 909.0287, 951.6699, 960.9595, 1017.7114, 1019.5801, 1019.7258, 1033.7395, 1051.7457, 1051.7457, 1060.1041, 1082.0940, 1098.0653, 1151.7755, 1151.7755, 1165.7815, 1165.7815, 1194.6759, 1194.6759, 1266.6686, 1277.7600, 1334.9314, 1334.9314, 1448.9645, 1448.9645, 1475.8114, 1487.8091, 1595.9777, 1595.9777, 1618.1177, 1618.1177, 1633.9124, 1732.1512, 1902.3138, 1994.0560, 2221.2212

50. gi|551565531 **Mass:** 42563 **Score:** 37 **Expect:** 6.9e+003 **Matches:** 8
hypothetical protein EMIHUDRAFT_210302 [Emiliania huxleyi CCMP1516]

Observed	Mr(expt)	Mr(calc)	ppm	Start - End	Miss	Ions	Peptide
842.5546	841.5473	841.4658	96.9	191 - 198	0	---	R.VPLDGVSR.L
1036.6012	1035.5939	1035.5978	-3.74	336 - 343	0	---	K.QVLHVWVR.T
1036.6012	1035.5939	1035.5978	-3.74	336 - 343	0	---	K.QVLHVWVR.T
1194.6759	1193.6686	1193.6115	47.9	181 - 190	0	---	R.FLVGEDMVLR.V + Oxidation (M)
1194.6759	1193.6686	1193.6115	47.9	181 - 190	0	6	R.FLVGEDMVLR.V + Oxidation (M)
1475.8114	1474.8041	1474.8507	-31.61	54 - 67	1	---	K.LALGKLGSSIPTYR.L
1487.8091	1486.8018	1486.7715	20.4	179 - 190	1	---	R.HRFLVGEDMVLR.V + Oxidation (M)

PDF-XChange Click to buy NOW! www.docu-track.com

PDF-XChange Click to buy NOW! www.docu-track.com

1633.9124 1632.9051 1632.9100 -2.98 156 - 170 0 --- R.WPTQKPAVLPAAQAR.K

No match to: 848.5696, 850.5803, 853.5549, 853.5549, 855.0955, 864.5864, 868.5867, 868.5867, 871.0599, 877.0814, 882.5994, 882.5994, 887.0316, 909.0287, 951.6699, 960.9595, 1017.7114, 1019.5801, 1019.7258, 1033.7395, 1051.7457, 1051.7457, 1060.1041, 1082.0940, 1098.0653, 1151.7755, 1151.7755, 1165.7815, 1165.7815, 1266.6686, 1277.7600, 1296.7419, 1334.9314, 1334.9314, 1448.9645, 1448.9645, 1595.9777, 1595.9777, 1618.1177, 1618.1177, 1732.1512, 1784.0413, 1902.3138, 1994.0560, 2221.2212, 2284.2476

Search Parameters

**Type of search**	**: Sequence Query**
**Enzyme**	**: Trypsin**
**Fixed modifications**	**: Carbamidomethyl (C)**
**Variable modifications**	**: Oxidation (M)**
**Mass values**	**: Monoisotopic**
**Protein Mass**	**: Unrestricted**
**Peptide Mass Tolerance**	**: ± 100 ppm**
**Fragment Mass Tolerance**	**: ± 0.3 Da**
**Max Missed Cleavages**	**: 1**
**Instrument type**	**: MALDI-TOF-TOF**
**Query1 (842.5546,1+)**	**: <no title>**
**Query2 (848.5696,1+)**	**: <no title>**
**Query3 (850.5803,1+)**	**: <no title>**
**Query4 (853.5549,1+)**	**: <no title>**
**Query5 (853.5549,1+)**	**: Locus:1..5.0.8**
**Query6 (855.0955,1+)**	**: <no title>**
**Query7 (864.5864,1+)**	**: <no title>**
**Query8 (868.5867,1+)**	**: Locus:1..5.0.7**
**Query9 (868.5867,1+)**	**: <no title>**
**Query10 (871.0599,1+)**	**: <no title>**
**Query11 (877.0814,1+)**	**: <no title>**
**Query12 (882.5994,1+)**	**: <no title>**
**Query13 (882.5994,1+)**	**: Locus:1..5.0.4**
**Query14 (887.0316,1+)**	**: <no title>**
**Query15 (909.0287,1+)**	**: <no title>**
**Query16 (951.6699,1+)**	**: <no title>**
**Query17 (960.9595,1+)**	**: <no title>**
**Query18 (1017.7114,1+)**	**: <no title>**
**Query19 (1019.5801,1+)**	**: <no title>**
**Query20 (1019.7258,1+)**	**: <no title>**
**Query21 (1033.7395,1+)**	**: <no title>**
**Query22 (1036.6012,1+)**	**: <no title>**
**Query23 (1036.6012,1+)**	**: Locus:1..5.0.11**
**Query24 (1051.7457,1+)**	**: <no title>**
**Query25 (1051.7457,1+)**	**: Locus:1..5.0.1**
**Query26 (1060.1041,1+)**	**: <no title>**
**Query27 (1082.0940,1+)**	**: <no title>**
**Query28 (1098.0653,1+)**	**: <no title>**
**Query29 (1151.7755,1+)**	**: <no title>**
**Query30 (1151.7755,1+)**	**: Locus:1..5.0.12**
**Query31 (1165.7815,1+)**	**: Locus:1..5.0.6**
**Query32 (1165.7815,1+)**	**: <no title>**
**Query33 (1194.6759,1+)**	**: <no title>**
**Query34 (1194.6759,1+)**	**: Locus:1..5.0.2**
**Query35 (1266.6686,1+)**	**: <no title>**
**Query36 (1277.7600,1+)**	**: <no title>**
**Query37 (1296.7419,1+)**	**: <no title>**
**Query38 (1334.9314,1+)**	**: <no title>**
**Query39 (1334.9314,1+)**	**: Locus:1..5.0.3**
**Query40 (1448.9645,1+)**	**: Locus:1..5.0.10**
**Query41 (1448.9645,1+)**	**: <no title>**
**Query42 (1475.8114,1+)**	**: <no title>**
**Query43 (1487.8091,1+)**	**: <no title>**
**Query44 (1595.9777,1+)**	**: Locus:1..5.0.5**
**Query45 (1595.9777,1+)**	**: <no title>**
**Query46 (1618.1177,1+)**	**: Locus:1..5.0.9**
**Query47 (1618.1177,1+)**	**: <no title>**
**Query48 (1633.9124,1+)**	**: <no title>**
**Query49 (1732.1512,1+)**	**: <no title>**
**Query50 (1784.0413,1+)**	**: <no title>**
**Query51 (1902.3138,1+)**	**: <no title>**
**Query52 (1994.0560,1+)**	**: <no title>**
**Query53 (2221.2212,1+)**	**: <no title>**
**Query54 (2284.2476,1+)**	**: <no title>**

Mascot: http://www.matrixscience.com/
