# Supplementary material for: Alpha-2-Macroglobulin as a New Promising Biomarker Improving the Diagnostic Sensitivity of Bovine Paratuberculosis
Source: Front Vet Sci. 2021 Mar 5;8:637716. doi: 10.3389/fvets.2021.637716 (PMC7973028; doi:10.3389/fvets.2021.637716)
Supplement: Supplementary Data 1 — Mascot search results and raw data files for LC-MS/MS analysis for the identification of specific blood proteins in cattle with Johne's disease. [file Data_Sheet_1.zip › mascot/5 IgM heavy chain constant region_2.pdf]

PDF-XChange Click to buy NOW! www.docu-track.com

PDF-XChange Click to buy NOW! www.docu-track.com

MATRIX SCIENCE

Mascot Search Results

Protein View

Match to: **gi|2232299** Score: **50** Expect: **3.1e+002**
IgM heavy chain constant region [Bos taurus]

Nominal mass (M_r): **48512**; Calculated pI value: **5.68**
NCBI BLAST search of gi|2232299 against nr
Unformatted sequence string for pasting into other applications

Taxonomy: Bos taurus

Fixed modifications: Carbamidomethyl (C)
Variable modifications: Oxidation (M)
Cleavage by Trypsin: cuts C-term side of KR unless next residue is P
Sequence Coverage: **16%**

Matched peptides shown in **Bold Red**

**1**	EGESHPRVFP	LVSCVSSPSD	ESTVALGCLA	RDFVPNSVSF	SWKFNNSTVN
**51**	SER**FWTFPEV**	**LR**DGLWSASS	QVVLPSSSAF	QGPDDYLVCE	VQHPKGGKTV
**101**	GTVRVIATK**A**	**EVLSPVVSVF**	**VPPR**NSLSGD	GNSKSSLICQ	ATDFSPK**QIS**
**151**	**LSWFR**DGKRI	VSGISEGQVE	TVQSSPVTFR	AYSMLTITER	DWLSQNVYTC
**201**	QVEHNKETFQ	KNVSSSCDVA	PPSPIGVFTI	PPSFADIFLT	KSAKLSCLVT
**251**	NLASYDGLNI	SWSRQNGK**AL**	**ETHTYFER**HL	NDTFSARGEA	SVCSEDWESG
**301**	EEFTCTVAHS	DLPFPKKNSV	SKPK**DVAMKP**	**PSVYLLPPTR**	EQLSLRESAS
**351**	VTCLVK**GFAP**	**ADVFVQWLQR**	GEPVTKSKYV	TSSPAPEPQD	PSVYFVHSIP
**401**	TVAEEDWSKG	ETYTCVVGHE	ALPHMVTERT	VDKSTGK	

Show predicted peptides also

Sort Peptides By   ◉ Residue Number   ○ Increasing Mass   ○ Decreasing Mass

Start - End	Observed	Mr(expt)	Mr(calc)	ppm	Miss	Sequence	
54 - 62	1194.6759	1193.6686	1193.6233	38	0	R.FWTFPEVLR.D	(No match)
54 - 62	1194.6759	1193.6686	1193.6233	38	0	R.FWTFPEVLR.D	(Ions score 18)
110 - 124	1595.9777	1594.9704	1594.9083	39	0	K.AEVLSPVVSVFVPPR.N	(No match)
110 - 124	1595.9777	1594.9704	1594.9083	39	0	K.AEVLSPVVSVFVPPR.N	(No match)
148 - 155	1036.6012	1035.5939	1035.5502	42	0	K.QISLSWFR.D	(No match)
148 - 155	1036.6012	1035.5939	1035.5502	42	0	K.QISLSWFR.D	(No match)
269 - 278	1266.6686	1265.6613	1265.6040	45	0	K.ALETHTYFER.H	(No match)
325 - 340	1784.0413	1783.0340	1782.9702	36	0	K.DVAMKPPSVYLLPPTR.E	(No match)
357 - 370	1633.9124	1632.9051	1632.8413	39	0	K.GFAPADVFVQWLQR.G	(No match)

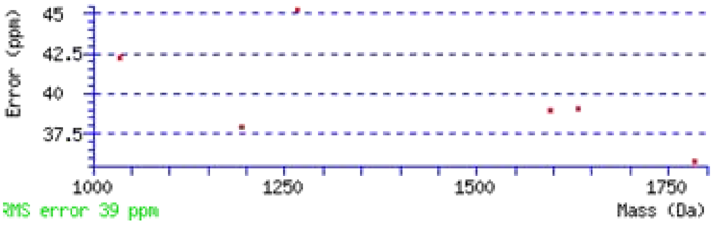


```
LOCUS       AAB62251                 437 aa            linear   MAM 24-JUL-2016
DEFINITION  IgM heavy chain constant region, partial [Bos taurus].
ACCESSION   AAB62251
VERSION     AAB62251.1
DBSOURCE    accession AF005274.1
KEYWORDS    .
SOURCE      Bos taurus (cattle)
  ORGANISM  Bos taurus
            Eukaryota; Metazoa; Chordata; Craniata; Vertebrata; Euteleostomi;
            Mammalia; Eutheria; Laurasiatheria; Cetartiodactyla; Ruminantia;
            Pecora; Bovidae; Bovinae; Bos.
REFERENCE   1  (residues 1 to 437)
  AUTHORS   Saini,S.S. and Kaushik,A.
  TITLE     Origin of bovine IgM structural variants
  JOURNAL   Mol. Immunol. 38 (5), 389-396 (2001)
   PUBMED   11684295
REFERENCE   2  (residues 1 to 437)
  AUTHORS   Saini,S.S. and Kaushik,A.
```

PDF-XChange Click to buy NOW! www.docu-track.com

```
  TITLE     Structural analysis of a bovine IgM
  JOURNAL   Unpublished
REFERENCE   3  (residues 1 to 437)
  AUTHORS   Saini,S.S. and Kaushik,A.
  TITLE     Direct Submission
  JOURNAL   Submitted (26-MAY-1997) Pathobiology, University of Guelph, Guelph,
            ON N1G 2W1, Canada
COMMENT     Method: conceptual translation.
FEATURES             Location/Qualifiers
     source          1..437
                     /organism="Bos taurus"
                     /strain="Holstein"
                     /db_xref="taxon:9913"
                     /cell_line="BL7G1"
                     /cell_type="mouse-bovine heterohybridoma"
                     /note="suffering from bovine leukosis"
     Protein         <1..>437
                     /product="IgM heavy chain constant region"
     Region          6..100
                     /region_name="Ig"
                     /note="Immunoglobulin domain; cl11960"
                     /db_xref="CDD:299845"
     Region          117..214
                     /region_name="IgC"
                     /note="Immunoglobulin Constant domain; cd00098"
                     /db_xref="CDD:143166"
     Site            order(118..121,136,138,140,142,179..182,184,186..187,
                     212..214)
                     /site_type="other"
                     /note="dimer interface [polypeptide binding]"
                     /db_xref="CDD:143166"
     Region          227..321
                     /region_name="Ig"
                     /note="Immunoglobulin domain; cl11960"
                     /db_xref="CDD:299845"
     Region          331..432
                     /region_name="Ig"
                     /note="Immunoglobulin domain; cl11960"
                     /db_xref="CDD:299845"
     CDS             1..437
                     /coded_by="AF005274.1:<1..>1311"
```

Mascot: http://www.matrixscience.com/
