# Supplementary material for: Alpha-2-Macroglobulin as a New Promising Biomarker Improving the Diagnostic Sensitivity of Bovine Paratuberculosis
Source: Front Vet Sci. 2021 Mar 5;8:637716. doi: 10.3389/fvets.2021.637716 (PMC7973028; doi:10.3389/fvets.2021.637716)
Supplement: Supplementary Data 1 — Mascot search results and raw data files for LC-MS/MS analysis for the identification of specific blood proteins in cattle with Johne's disease. [file Data_Sheet_1.zip › mascot/6 Complement component 3d_1.pdf]

PDF-XChange
Click to buy NOW!
www.docu-track.com

PDF-XChange
Click to buy NOW!
www.docu-track.com

MATRIX SCIENCE Mascot Search Results

```
User          :
Email         :
Search title  : BAEDW\2020_3_5\2020_3_5\MSMS 14\N16
Database      : Uniprot_bovine bovine_20200316 (134392 sequences; 76244912 residues)
Timestamp     : 31 Mar 2020 at 01:08:51 GMT
Warning       : A Peptide summary report will usually give a much clearer picture of MS/MS search results.
Top Score     : 251 for Q693V9, Complement component 3d (Fragment) OS=Bos taurus OX=9913 PE=2 SV=1
```

Mascot Score Histogram

Protein score is -10*Log(P), where P is the probability that the observed match is a random event.
Protein scores greater than 64 are significant (p<0.05).
Protein scores are derived from ions scores as a non-probabilistic basis for ranking protein hits.

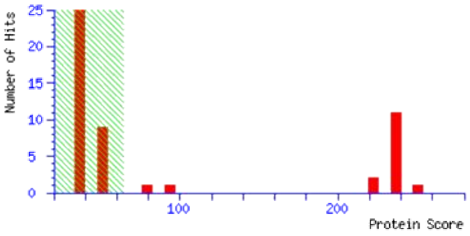

Protein Summary Report

Format As | Protein Summary (deprecated) | Help

Significance threshold p< 0.05 Max. number of hits 30

Preferred taxonomy All entries

Re-Search All | Search Unmatched

Index

	Accession	Mass	Score	Description
1.	Q693V9	34593	251	Complement component 3d (Fragment) OS=Bos taurus OX=9913 PE=2 SV=1
2.	A0A4W2D408	180682	237	Complement C3 OS=Bos indicus x Bos taurus OX=30522 GN=C3 PE=4 SV=1
3.	A0A3Q1MGT0	183008	236	Complement C3 OS=Bos taurus OX=9913 GN=C3 PE=1 SV=1
4.	A0A3Q1M2B2	185169	235	Complement C3 OS=Bos taurus OX=9913 GN=C3 PE=1 SV=1
5.	A0A4W2D441	186349	233	Complement C3 OS=Bos indicus x Bos taurus OX=30522 GN=C3 PE=4 SV=1
6.	Q2UVX4	188675	232	Complement C3 OS=Bos taurus OX=9913 GN=C3 PE=1 SV=2
7.	A0A0F6QNP7	188604	232	Complement component 3 OS=Bos taurus OX=9913 GN=C3 PE=2 SV=1
8.	A0A3Q1MHV6	181738	231	Complement C3 OS=Bos taurus OX=9913 GN=C3 PE=1 SV=1
9.	A0A4W2D3Y9	185555	231	Complement C3 OS=Bos indicus x Bos taurus OX=30522 GN=C3 PE=4 SV=1
10.	A0A4W2EPV7	179159	231	Complement C3 OS=Bos indicus x Bos taurus OX=30522 GN=C3 PE=4 SV=1
11.	A0A4W2D406	186684	231	Complement C3 OS=Bos indicus x Bos taurus OX=30522 GN=C3 PE=4 SV=1
12.	A0A4W2D411	190190	230	Complement C3 OS=Bos indicus x Bos taurus OX=30522 GN=C3 PE=4 SV=1
13.	A0A4W2CZN6	192486	229	Complement C3 OS=Bos indicus x Bos taurus OX=30522 GN=C3 PE=4 SV=1
14.	A0A4W2EPI5	182918	229	Complement C3 OS=Bos indicus x Bos taurus OX=30522 GN=C3 PE=4 SV=1
15.	A0A4W2HFJ5	188690	98	Complement C3 OS=Bos indicus x Bos taurus OX=30522 GN=C3 PE=4 SV=1
16.	A0A4W2CZQ8	170639	79	Complement C3 OS=Bos indicus x Bos taurus OX=30522 GN=C3 PE=4 SV=1
17.	A0A4W2DYD9	31930	54	Phosphatidylinositol transfer protein beta OS=Bos indicus x Bos taurus OX=30522 GN=PITPNB PE=4 SV=1
18.	A0A4W2C513	31804	53	Phosphatidylinositol transfer protein beta OS=Bos indicus x Bos taurus OX=30522 GN=PITPNB PE=4 SV=1
19.	Q9TR36	31804	53	Phosphatidylinositol transfer protein beta isoform OS=Bos taurus OX=9913 GN=PITPNB PE=1 SV=3
20.	A0A4W2FGB2	205135	53	Poly [ADP-ribose] polymerase OS=Bos indicus x Bos taurus OX=30522 GN=PARP14 PE=4 SV=1
21.	A0A4W2EP89	190854	50	Poly(ADP-ribose) polymerase family member 14 OS=Bos indicus x Bos taurus OX=30522 GN=PARP14 PE=4 SV=1
22.	F1ML90	204793	49	Poly [ADP-ribose] polymerase OS=Bos taurus OX=9913 GN=PARP14 PE=4 SV=3
23.	A0A4W2CYS7	190256	48	Poly(ADP-ribose) polymerase family member 14 OS=Bos indicus x Bos taurus OX=30522 GN=PARP14 PE=4 SV=1
24.	A0A3Q1MDH0	196044	44	Poly [ADP-ribose] polymerase OS=Bos taurus OX=9913 GN=PARP14 PE=4 SV=1
25.	A0A4W2E5T5	31943	44	Phosphatidylinositol transfer protein beta OS=Bos indicus x Bos taurus OX=30522 GN=PITPNB PE=4 SV=1
26.	A0A452DJF3	31845	43	Phosphatidylinositol transfer protein beta isoform OS=Bos taurus OX=9913 GN=PITPNB PE=4 SV=1
27.	A0A4W2DWE1	8549	41	CD3e molecule OS=Bos indicus x Bos taurus OX=30522 GN=CD3E PE=4 SV=1
28.	A0A3Q1MWW7	28595	40	Electron transfer flavoprotein subunit beta lysine methyltransferase OS=Bos taurus OX=9913 GN=ETFBKMT P[illegible]
29.	A0A3Q1M1B1	29199	40	Phosphatidylinositol transfer protein beta isoform OS=Bos taurus OX=9913 GN=PITPNB PE=4 SV=1
30.	Q8MII7	4845	40	Insulin receptor (Fragment) OS=Bos taurus OX=9913 GN=ir-A PE=2 SV=1

Results List

1. Q693V9 **Mass:** 34593 **Score:** 251 **Expect:** 1.1e-020 **Matches:** 20
Complement component 3d (Fragment) OS=Bos taurus OX=9913 PE=2 SV=1

Observed	Mr(expt)	Mr(calc)	ppm	Start	End	Miss	Ions	Peptide
845.4012	844.3939	844.4191	-29.85	254 -	259	0	---	R.WLNEQR.Y
987.5186	986.5113	986.5396	-28.69	42 -	49	0	---	R.QESLELIR.K
1083.5225	1082.5152	1082.5509	-32.98	51 -	59	0	14	K.GYTQQLAFR.Q
1083.5225	1082.5152	1082.5509	-32.94	51 -	59	0	---	K.GYTQQLAFR.Q
1143.6165	1142.6092	1142.6407	-27.57	41 -	49	1	---	K.RQESLELIR.K
1162.5488	1161.5415	1161.5666	-21.61	244 -	253	0	---	K.DYDTTPPVVR.W
1200.5436	1199.5363	1199.5683	-26.65	217 -	225	1	---	K.NRWEEPNQK.L
1211.6163	1210.6090	1210.6458	-30.41	50 -	59	1	---	R.KGYTQQLAFR.Q
1211.6163	1210.6091	1210.6458	-30.39	50 -	59	1	---	R.KGYTQQLAFR.Q
1221.5270	1220.5197	1220.5574	-30.90	171 -	180	0	40	K.AGDFLENHYR.E
1221.5270	1220.5197	1220.5574	-30.88	171 -	180	0	---	K.AGDFLENHYR.E
1290.6333	1289.6260	1289.6616	-27.57	243 -	253	1	---	R.KDYDTTPPVVR.W
1361.6049	1360.5976	1360.6405	-31.51	155 -	166	0	---	K.DICEAQVNSLGR.S
1790.9982	1789.9909	1790.0454	-30.45	184 -	200	0	---	R.RPYTVAIAAYALALLGK.L
1790.9982	1789.9909	1790.0454	-30.43	184 -	200	0	---	R.RPYTVAIAAYALALLGK.L
1880.9891	1879.9818	1880.0407	-31.31	226 -	242	0	---	K.LYNVEATSYALLALLAR.K

PDF-XChange Click to buy NOW! www.docu-track.com

PDF-XChange Click to buy NOW! www.docu-track.com

Observed	Mr(expt)	Mr(calc)	ppm	Start	End	Miss	Ions	Peptide
1880.9891	1879.9819	1880.0407	-31.29	226	242	0	80	K.LYNVEATSYALLALLAR.K
2506.2144	2505.2071	2505.2692	-24.80	62	83	0	---	K.SSAYAAFQYRPPSTWLTAYVVK.V
2506.2144	2505.2071	2505.2692	-24.79	62	83	0	---	K.SSAYAAFQYRPPSTWLTAYVVK.V
2598.2195	2597.2122	2597.2697	-22.12	111	133	0	---	K.QKPDGIFQEDGPVIHQEMIGGFR.D

No match to: 842.4847, 848.4952, 850.5135, 853.4918, 857.4026, 857.4026, 864.5186, 868.5277, 870.5200, 882.5434, 882.5434, 951.5951, 996.5623, 1017.6425, 1019.6487, 1033.6698, 1036.5013, 1036.5013, 1045.5382, 1051.6785, 1051.6785, 1090.4995, 1107.5156, 1124.5784, 1131.6732, 1136.6610, 1151.6952, 1165.5533, 1165.7053, 1179.5667, 1263.6616, 1277.6742, 1300.8134, 1334.8459, 1334.8459, 1379.6898, 1407.6674, 1434.8688, 1448.8787, 1475.7053, 1479.7522, 1482.7051, 1567.6991, 1618.0178, 1732.0514, 1808.9220, 1879.9926, 1890.9176, 1901.2008, 1993.9156, 2064.0874, 2098.0544, 2157.0273, 2157.0273, 2179.9844, 2211.0493, 2283.1272

2. A0A4W2D408 **Mass:** 180682 **Score:** 237 **Expect:** 2.7e-019 **Matches:** 28

Complement C3 OS=Bos indicus x Bos taurus OX=30522 GN=C3 PE=4 SV=1

Observed	Mr(expt)	Mr(calc)	ppm	Start	End	Miss	Ions	Peptide
845.4012	844.3939	844.3927	1.50	477	484	0	---	R.TDPGEQAK.I
848.4952	847.4879	847.4400	56.6	1368	1375	0	---	K.TLSTGVDR.Y
850.5135	849.5062	849.4266	93.8	639	645	0	---	R.SVQLMEK.R + Oxidation (M)
864.5186	863.5113	863.4939	20.2	758	764	0	---	K.LMNVFLK.D
987.5186	986.5113	986.5396	-28.69	1011	1018	0	---	R.QESLELIR.K
996.5623	995.5550	995.4672	88.2	650	658	0	---	K.AGQYSSDLR.K
1083.5225	1082.5152	1082.5509	-32.98	1020	1028	0	14	K.GYTQQLAFR.Q
1083.5225	1082.5152	1082.5509	-32.94	1020	1028	0	---	K.GYTQQLAFR.Q
1090.4995	1089.4922	1089.4873	4.50	590	600	0	---	K.ADIGCTPGSGR.N
1124.5784	1123.5711	1123.5622	7.97	650	659	1	---	K.AGQYSSDLRK.C
1143.6165	1142.6092	1142.6407	-27.57	1010	1018	1	---	K.RQESLELIR.K
1162.5488	1161.5415	1161.5666	-21.61	1213	1222	0	---	K.DYDTTPPVVR.W
1200.5436	1199.5363	1199.5683	-26.65	1186	1194	1	---	K.NRWEEPNQK.L
1211.6163	1210.6090	1210.6458	-30.41	1019	1028	1	---	R.KGYTQQLAFR.Q
1211.6163	1210.6091	1210.6458	-30.39	1019	1028	1	---	R.KGYTQQLAFR.Q
1221.5270	1220.5197	1220.5574	-30.90	1140	1149	0	40	K.AGDFLENHYR.E
1221.5270	1220.5197	1220.5574	-30.88	1140	1149	0	---	K.AGDFLENHYR.E
1290.6333	1289.6260	1289.6616	-27.57	1212	1222	1	---	R.KDYDTTPPVVR.W
1361.6049	1360.5976	1360.6405	-31.51	1124	1135	0	---	K.DICEAQVNSLGR.S
1790.9982	1789.9909	1790.0454	-30.45	1153	1169	0	---	R.RPYTVAIAAYALALLGK.L
1790.9982	1789.9909	1790.0454	-30.43	1153	1169	0	---	R.RPYTVAIAAYALALLGK.L
1880.9891	1879.9818	1880.0407	-31.31	1195	1211	0	---	K.LYNVEATSYALLALLAR.K
1880.9891	1879.9819	1880.0407	-31.29	1195	1211	0	80	K.LYNVEATSYALLALLAR.K
2157.0273	2156.0200	2156.0783	-27.04	948	967	0	---	K.ILLQGTPVAQMTEDAIDGER.L
2157.0273	2156.0201	2156.0783	-27.02	948	967	0	35	K.ILLQGTPVAQMTEDAIDGER.L
2506.2144	2505.2071	2505.2692	-24.80	1031	1052	0	---	K.SSAYAAFQYRPPSTWLTAYVVK.V
2506.2144	2505.2071	2505.2692	-24.79	1031	1052	0	---	K.SSAYAAFQYRPPSTWLTAYVVK.V
2598.2195	2597.2122	2597.2697	-22.12	1080	1102	0	---	K.QKPDGIFQEDGPVIHQEMIGGFR.D

No match to: 842.4847, 853.4918, 857.4026, 857.4026, 868.5277, 870.5200, 882.5434, 882.5434, 951.5951, 1017.6425, 1019.6487, 1033.6698, 1036.5013, 1036.5013, 1045.5382, 1051.6785, 1051.6785, 1107.5156, 1131.6732, 1136.6610, 1151.6952, 1165.5533, 1165.7053, 1179.5667, 1263.6616, 1277.6742, 1300.8134, 1334.8459, 1334.8459, 1379.6898, 1407.6674, 1434.8688, 1448.8787, 1475.7053, 1479.7522, 1482.7051, 1567.6991, 1618.0178, 1732.0514, 1808.9220, 1879.9926, 1890.9176, 1901.2008, 1993.9156, 2064.0874, 2098.0544, 2179.9844, 2211.0493, 2283.1272

3. A0A3Q1MGT0 **Mass:** 183008 **Score:** 236 **Expect:** 3.4e-019 **Matches:** 28

Complement C3 OS=Bos taurus OX=9913 GN=C3 PE=1 SV=1

Observed	Mr(expt)	Mr(calc)	ppm	Start	End	Miss	Ions	Peptide
845.4012	844.3939	844.3927	1.50	477	484	0	---	R.TDPGEQAK.I
848.4952	847.4879	847.4400	56.6	1368	1375	0	---	K.TLSTGVDR.Y
850.5135	849.5062	849.4266	93.8	639	645	0	---	R.SVQLMEK.R + Oxidation (M)
864.5186	863.5113	863.4939	20.2	758	764	0	---	K.LMNVFLK.D
987.5186	986.5113	986.5396	-28.69	1011	1018	0	---	R.QESLELIR.K
996.5623	995.5550	995.4672	88.2	650	658	0	---	K.AGQYSSDLR.K
1083.5225	1082.5152	1082.5509	-32.98	1020	1028	0	14	K.GYTQQLAFR.Q
1083.5225	1082.5152	1082.5509	-32.94	1020	1028	0	---	K.GYTQQLAFR.Q
1090.4995	1089.4922	1089.4873	4.50	590	600	0	---	K.ADIGCTPGSGR.N
1124.5784	1123.5711	1123.5622	7.97	650	659	1	---	K.AGQYSSDLRK.C
1143.6165	1142.6092	1142.6407	-27.57	1010	1018	1	---	K.RQESLELIR.K
1162.5488	1161.5415	1161.5666	-21.61	1213	1222	0	---	K.DYDTTPPVVR.W
1200.5436	1199.5363	1199.5683	-26.65	1186	1194	1	---	K.NRWEEPNQK.L
1211.6163	1210.6090	1210.6458	-30.41	1019	1028	1	---	R.KGYTQQLAFR.Q
1211.6163	1210.6091	1210.6458	-30.39	1019	1028	1	---	R.KGYTQQLAFR.Q
1221.5270	1220.5197	1220.5574	-30.90	1140	1149	0	40	K.AGDFLENHYR.E
1221.5270	1220.5197	1220.5574	-30.88	1140	1149	0	---	K.AGDFLENHYR.E
1290.6333	1289.6260	1289.6616	-27.57	1212	1222	1	---	R.KDYDTTPPVVR.W
1361.6049	1360.5976	1360.6405	-31.51	1124	1135	0	---	K.DICEAQVNSLGR.S
1790.9982	1789.9909	1790.0454	-30.45	1153	1169	0	---	R.RPYTVAIAAYALALLGK.L
1790.9982	1789.9909	1790.0454	-30.43	1153	1169	0	---	R.RPYTVAIAAYALALLGK.L
1880.9891	1879.9818	1880.0407	-31.31	1195	1211	0	---	K.LYNVEATSYALLALLAR.K
1880.9891	1879.9819	1880.0407	-31.29	1195	1211	0	80	K.LYNVEATSYALLALLAR.K
2157.0273	2156.0200	2156.0783	-27.04	948	967	0	---	K.ILLQGTPVAQMTEDAIDGER.L
2157.0273	2156.0201	2156.0783	-27.02	948	967	0	35	K.ILLQGTPVAQMTEDAIDGER.L
2506.2144	2505.2071	2505.2692	-24.80	1031	1052	0	---	K.SSAYAAFQYRPPSTWLTAYVVK.V
2506.2144	2505.2071	2505.2692	-24.79	1031	1052	0	---	K.SSAYAAFQYRPPSTWLTAYVVK.V
2598.2195	2597.2122	2597.2697	-22.12	1080	1102	0	---	K.QKPDGIFQEDGPVIHQEMIGGFR.D

No match to: 842.4847, 853.4918, 857.4026, 857.4026, 868.5277, 870.5200, 882.5434, 882.5434, 951.5951, 1017.6425, 1019.6487, 1033.6698, 1036.5013, 1036.5013, 1045.5382, 1051.6785, 1051.6785, 1107.5156, 1131.6732, 1136.6610, 1151.6952, 1165.5533, 1165.7053, 1179.5667, 1263.6616, 1277.6742, 1300.8134, 1334.8459, 1334.8459, 1379.6898, 1407.6674, 1434.8688, 1448.8787, 1475.7053, 1479.7522, 1482.7051, 1567.6991, 1618.0178, 1732.0514, 1808.9220, 1879.9926, 1890.9176, 1901.2008, 1993.9156, 2064.0874, 2098.0544, 2179.9844, 2211.0493, 2283.1272

4. A0A3Q1M2B2 **Mass:** 185169 **Score:** 235 **Expect:** 4.2e-019 **Matches:** 28

Complement C3 OS=Bos taurus OX=9913 GN=C3 PE=1 SV=1

Observed	Mr(expt)	Mr(calc)	ppm	Start	End	Miss	Ions	Peptide
845.4012	844.3939	844.3927	1.50	477	484	0	---	R.TDPGEQAK.I
848.4952	847.4879	847.4400	56.6	1388	1395	0	---	K.TLSTGVDR.Y
850.5135	849.5062	849.4266	93.8	639	645	0	---	R.SVQLMEK.R + Oxidation (M)
864.5186	863.5113	863.4939	20.2	758	764	0	---	K.LMNVFLK.D
987.5186	986.5113	986.5396	-28.69	1011	1018	0	---	R.QESLELIR.K
996.5623	995.5550	995.4672	88.2	650	658	0	---	K.AGQYSSDLR.K

PDF-XChange Click to buy NOW! www.docu-track.com
PDF-XChange Click to buy NOW! www.docu-track.com

1083.5225	1082.5152	1082.5509	-32.98	1020 -	1028	0	14	K.GYTQQLAFR.Q
1083.5225	1082.5152	1082.5509	-32.94	1020 -	1028	0	---	K.GYTQQLAFR.Q
1090.4995	1089.4922	1089.4873	4.50	590 -	600	0	---	K.ADIGCTPGSGR.N
1124.5784	1123.5711	1123.5622	7.97	650 -	659	1	---	K.AGQYSSDLRK.C
1143.6165	1142.6092	1142.6407	-27.57	1010 -	1018	1	---	K.RQESLELIR.K
1162.5488	1161.5415	1161.5666	-21.61	1213 -	1222	0	---	K.DYDTTPPVVR.W
1200.5436	1199.5363	1199.5683	-26.65	1186 -	1194	1	---	K.NRWEEPNQK.L
1211.6163	1210.6090	1210.6458	-30.41	1019 -	1028	1	---	R.KGYTQQLAFR.Q
1211.6163	1210.6091	1210.6458	-30.39	1019 -	1028	1	---	R.KGYTQQLAFR.Q
1221.5270	1220.5197	1220.5574	-30.90	1140 -	1149	0	40	K.AGDFLENHYR.E
1221.5270	1220.5197	1220.5574	-30.88	1140 -	1149	0	---	K.AGDFLENHYR.E
1290.6333	1289.6260	1289.6616	-27.57	1212 -	1222	1	---	R.KDYDTTPPVVR.W
1361.6049	1360.5976	1360.6405	-31.51	1124 -	1135	0	---	K.DICEAQVNSLGR.S
1790.9982	1789.9909	1790.0454	-30.45	1153 -	1169	0	---	R.RPYTVAIAAYALALLGK.L
1790.9982	1789.9909	1790.0454	-30.43	1153 -	1169	0	---	R.RPYTVAIAAYALALLGK.L
1880.9891	1879.9818	1880.0407	-31.31	1195 -	1211	0	---	K.LYNVEATSYALLALLAR.K
1880.9891	1879.9819	1880.0407	-31.29	1195 -	1211	0	80	K.LYNVEATSYALLALLAR.K
2157.0273	2156.0200	2156.0783	-27.04	948 -	967	0	---	K.ILLQGTPVAQMTEDAIDGER.L
2157.0273	2156.0201	2156.0783	-27.02	948 -	967	0	35	K.ILLQGTPVAQMTEDAIDGER.L
2506.2144	2505.2071	2505.2692	-24.80	1031 -	1052	0	---	K.SSAYAAFQYRPPSTWLTAYVVK.V
2506.2144	2505.2071	2505.2692	-24.79	1031 -	1052	0	---	K.SSAYAAFQYRPPSTWLTAYVVK.V
2598.2195	2597.2122	2597.2697	-22.12	1080 -	1102	0	---	K.QKPDGIFQEDGPVIHQEMIGGFR.D

No match to: 842.4847, 853.4918, 857.4026, 857.4026, 868.5277, 870.5200, 882.5434, 882.5434, 951.5951, 1017.6425, 1019.6487, 1033.6698, 1036.5013, 1036.5013, 1045.5382, 1051.6785, 1051.6785, 1107.5156, 1131.6732, 1136.6610, 1151.6952, 1165.5533, 1165.7053, 1179.5667, 1263.6616, 1277.6742, 1300.8134, 1334.8459, 1334.8459, 1379.6898, 1407.6674, 1434.8688, 1448.8787, 1475.7053, 1479.7522, 1482.7051, 1567.6991, 1618.0178, 1732.0514, 1808.9220, 1879.9926, 1890.9176, 1901.2008, 1993.9156, 2064.0874, 2098.0544, 2179.9844, 2211.0493, 2283.1272

5. A0A4W2D441 **Mass:** 186349 **Score:** 233 **Expect:** 6.7e-019 **Matches:** 28
Complement C3 OS=Bos indicus x Bos taurus OX=30522 GN=C3 PE=4 SV=1

Observed	Mr(expt)	Mr(calc)	ppm	Start	End	Miss	Ions	Peptide
845.4012	844.3939	844.3927	1.50	477 -	484	0	---	R.TDPGEQAK.I
848.4952	847.4879	847.4400	56.6	1419 -	1426	0	---	K.TLSTGVDR.Y
850.5135	849.5062	849.4266	93.8	670 -	676	0	---	R.SVQLMEK.R + Oxidation (M)
864.5186	863.5113	863.4939	20.2	789 -	795	0	---	K.LMNVFLK.D
987.5186	986.5113	986.5396	-28.69	1042 -	1049	0	---	R.QESLELIR.K
996.5623	995.5550	995.4672	88.2	681 -	689	0	---	K.AGQYSSDLR.K
1083.5225	1082.5152	1082.5509	-32.98	1051 -	1059	0	14	K.GYTQQLAFR.Q
1083.5225	1082.5152	1082.5509	-32.94	1051 -	1059	0	---	K.GYTQQLAFR.Q
1090.4995	1089.4922	1089.4873	4.50	621 -	631	0	---	K.ADIGCTPGSGR.N
1124.5784	1123.5711	1123.5622	7.97	681 -	690	1	---	K.AGQYSSDLRK.C
1143.6165	1142.6092	1142.6407	-27.57	1041 -	1049	1	---	K.RQESLELIR.K
1162.5488	1161.5415	1161.5666	-21.61	1244 -	1253	0	---	K.DYDTTPPVVR.W
1200.5436	1199.5363	1199.5683	-26.65	1217 -	1225	1	---	K.NRWEEPNQK.L
1211.6163	1210.6090	1210.6458	-30.41	1050 -	1059	1	---	R.KGYTQQLAFR.Q
1211.6163	1210.6091	1210.6458	-30.39	1050 -	1059	1	---	R.KGYTQQLAFR.Q
1221.5270	1220.5197	1220.5574	-30.90	1171 -	1180	0	40	K.AGDFLENHYR.E
1221.5270	1220.5197	1220.5574	-30.88	1171 -	1180	0	---	K.AGDFLENHYR.E
1290.6333	1289.6260	1289.6616	-27.57	1243 -	1253	1	---	R.KDYDTTPPVVR.W
1361.6049	1360.5976	1360.6405	-31.51	1155 -	1166	0	---	K.DICEAQVNSLGR.S
1790.9982	1789.9909	1790.0454	-30.45	1184 -	1200	0	---	R.RPYTVAIAAYALALLGK.L
1790.9982	1789.9909	1790.0454	-30.43	1184 -	1200	0	---	R.RPYTVAIAAYALALLGK.L
1880.9891	1879.9818	1880.0407	-31.31	1226 -	1242	0	---	K.LYNVEATSYALLALLAR.K
1880.9891	1879.9819	1880.0407	-31.29	1226 -	1242	0	80	K.LYNVEATSYALLALLAR.K
2157.0273	2156.0200	2156.0783	-27.04	979 -	998	0	---	K.ILLQGTPVAQMTEDAIDGER.L
2157.0273	2156.0201	2156.0783	-27.02	979 -	998	0	35	K.ILLQGTPVAQMTEDAIDGER.L
2506.2144	2505.2071	2505.2692	-24.80	1062 -	1083	0	---	K.SSAYAAFQYRPPSTWLTAYVVK.V
2506.2144	2505.2071	2505.2692	-24.79	1062 -	1083	0	---	K.SSAYAAFQYRPPSTWLTAYVVK.V
2598.2195	2597.2122	2597.2697	-22.12	1111 -	1133	0	---	K.QKPDGIFQEDGPVIHQEMIGGFR.D

No match to: 842.4847, 853.4918, 857.4026, 857.4026, 868.5277, 870.5200, 882.5434, 882.5434, 951.5951, 1017.6425, 1019.6487, 1033.6698, 1036.5013, 1036.5013, 1045.5382, 1051.6785, 1051.6785, 1107.5156, 1131.6732, 1136.6610, 1151.6952, 1165.5533, 1165.7053, 1179.5667, 1263.6616, 1277.6742, 1300.8134, 1334.8459, 1334.8459, 1379.6898, 1407.6674, 1434.8688, 1448.8787, 1475.7053, 1479.7522, 1482.7051, 1567.6991, 1618.0178, 1732.0514, 1808.9220, 1879.9926, 1890.9176, 1901.2008, 1993.9156, 2064.0874, 2098.0544, 2179.9844, 2211.0493, 2283.1272

6. Q2UVX4 **Mass:** 188675 **Score:** 232 **Expect:** 8.5e-019 **Matches:** 28
Complement C3 OS=Bos taurus OX=9913 GN=C3 PE=1 SV=2

Observed	Mr(expt)	Mr(calc)	ppm	Start	End	Miss	Ions	Peptide
845.4012	844.3939	844.3927	1.50	477 -	484	0	---	R.TDPGEQAK.I
848.4952	847.4879	847.4400	56.6	1419 -	1426	0	---	K.TLSTGVDR.Y
850.5135	849.5062	849.4266	93.8	670 -	676	0	---	R.SVQLMEK.R + Oxidation (M)
864.5186	863.5113	863.4939	20.2	789 -	795	0	---	K.LMNVFLK.D
987.5186	986.5113	986.5396	-28.69	1042 -	1049	0	---	R.QESLELIR.K
996.5623	995.5550	995.4672	88.2	681 -	689	0	---	K.AGQYSSDLR.K
1083.5225	1082.5152	1082.5509	-32.98	1051 -	1059	0	14	K.GYTQQLAFR.Q
1083.5225	1082.5152	1082.5509	-32.94	1051 -	1059	0	---	K.GYTQQLAFR.Q
1090.4995	1089.4922	1089.4873	4.50	621 -	631	0	---	K.ADIGCTPGSGR.N
1124.5784	1123.5711	1123.5622	7.97	681 -	690	1	---	K.AGQYSSDLRK.C
1143.6165	1142.6092	1142.6407	-27.57	1041 -	1049	1	---	K.RQESLELIR.K
1162.5488	1161.5415	1161.5666	-21.61	1244 -	1253	0	---	K.DYDTTPPVVR.W
1200.5436	1199.5363	1199.5683	-26.65	1217 -	1225	1	---	K.NRWEEPNQK.L
1211.6163	1210.6090	1210.6458	-30.41	1050 -	1059	1	---	R.KGYTQQLAFR.Q
1211.6163	1210.6091	1210.6458	-30.39	1050 -	1059	1	---	R.KGYTQQLAFR.Q
1221.5270	1220.5197	1220.5574	-30.90	1171 -	1180	0	40	K.AGDFLENHYR.E
1221.5270	1220.5197	1220.5574	-30.88	1171 -	1180	0	---	K.AGDFLENHYR.E
1290.6333	1289.6260	1289.6616	-27.57	1243 -	1253	1	---	R.KDYDTTPPVVR.W
1361.6049	1360.5976	1360.6405	-31.51	1155 -	1166	0	---	K.DICEAQVNSLGR.S
1790.9982	1789.9909	1790.0454	-30.45	1184 -	1200	0	---	R.RPYTVAIAAYALALLGK.L
1790.9982	1789.9909	1790.0454	-30.43	1184 -	1200	0	---	R.RPYTVAIAAYALALLGK.L
1880.9891	1879.9818	1880.0407	-31.31	1226 -	1242	0	---	K.LYNVEATSYALLALLAR.K
1880.9891	1879.9819	1880.0407	-31.29	1226 -	1242	0	80	K.LYNVEATSYALLALLAR.K
2157.0273	2156.0200	2156.0783	-27.04	979 -	998	0	---	K.ILLQGTPVAQMTEDAIDGER.L
2157.0273	2156.0201	2156.0783	-27.02	979 -	998	0	35	K.ILLQGTPVAQMTEDAIDGER.L

PDF-XChange Click to buy NOW! www.docu-track.com

PDF-XChange Click to buy NOW! www.docu-track.com

2506.2144	2505.2071	2505.2692	-24.80	1062 -	1083	0	---	K.SSAYAAFQYRPPSTWLTAYVVK.V
2506.2144	2505.2071	2505.2692	-24.79	1062 -	1083	0	---	K.SSAYAAFQYRPPSTWLTAYVVK.V
2598.2195	2597.2122	2597.2697	-22.12	1111 -	1133	0	---	K.QKPDGIFQEDGPVIHQEMIGGFR.D

No match to: 842.4847, 853.4918, 857.4026, 857.4026, 868.5277, 870.5200, 882.5434, 882.5434, 951.5951, 1017.6425, 1019.6487, 1033.6698, 1036.5013, 1036.5013, 1045.5382, 1051.6785, 1051.6785, 1107.5156, 1131.6732, 1136.6610, 1151.6952, 1165.5533, 1165.7053, 1179.5667, 1263.6616, 1277.6742, 1300.8134, 1334.8459, 1334.8459, 1379.6898, 1407.6674, 1434.8688, 1448.8787, 1475.7053, 1479.7522, 1482.7051, 1567.6991, 1618.0178, 1732.0514, 1808.9220, 1879.9926, 1890.9176, 1901.2008, 1993.9156, 2064.0874, 2098.0544, 2179.9844, 2211.0493, 2283.1272

7. A0A0F6QNP7 **Mass:** 188604 **Score:** 232 **Expect:** 8.5e-019 **Matches:** 28

Complement component 3 OS=Bos taurus OX=9913 GN=C3 PE=2 SV=1

Observed	Mr(expt)	Mr(calc)	ppm	Start	End	Miss	Ions	Peptide
845.4012	844.3939	844.3927	1.50	477 -	484	0	---	R.TDPGEQAK.I
848.4952	847.4879	847.4400	56.6	1419 -	1426	0	---	K.TLSTGVDR.Y
850.5135	849.5062	849.4266	93.8	670 -	676	0	---	R.SVQLMEK.R + Oxidation (M)
853.4918	852.4845	852.4779	7.76	789 -	795	0	---	K.LMSVFLK.D + Oxidation (M)
987.5186	986.5113	986.5396	-28.69	1042 -	1049	0	---	R.QESLELIR.K
996.5623	995.5550	995.4672	88.2	681 -	689	0	---	K.AGQYSSDLR.K
1083.5225	1082.5152	1082.5509	-32.98	1051 -	1059	0	14	K.GYTQQLAFR.Q
1083.5225	1082.5152	1082.5509	-32.94	1051 -	1059	0	---	K.GYTQQLAFR.Q
1090.4995	1089.4922	1089.4873	4.50	621 -	631	0	---	K.ADIGCTPGSGR.N
1124.5784	1123.5711	1123.5622	7.97	681 -	690	1	---	K.AGQYSSDLRK.C
1143.6165	1142.6092	1142.6407	-27.57	1041 -	1049	1	---	K.RQESLELIR.K
1162.5488	1161.5415	1161.5666	-21.61	1244 -	1253	0	---	K.DYDTTPPVVR.W
1200.5436	1199.5363	1199.5683	-26.65	1217 -	1225	1	---	K.NRWEEPNQK.L
1211.6163	1210.6090	1210.6458	-30.41	1050 -	1059	1	---	R.KGYTQQLAFR.Q
1211.6163	1210.6091	1210.6458	-30.39	1050 -	1059	1	---	R.KGYTQQLAFR.Q
1221.5270	1220.5197	1220.5574	-30.90	1171 -	1180	0	40	K.AGDFLENHYR.E
1221.5270	1220.5197	1220.5574	-30.88	1171 -	1180	0	---	K.AGDFLENHYR.E
1290.6333	1289.6260	1289.6616	-27.57	1243 -	1253	1	---	R.KDYDTTPPVVR.W
1361.6049	1360.5976	1360.6405	-31.51	1155 -	1166	0	---	K.DICEAQVNSLGR.S
1790.9982	1789.9909	1790.0454	-30.45	1184 -	1200	0	---	R.RPYTVAIAAYALALLGK.L
1790.9982	1789.9909	1790.0454	-30.43	1184 -	1200	0	---	R.RPYTVAIAAYALALLGK.L
1880.9891	1879.9818	1880.0407	-31.31	1226 -	1242	0	---	K.LYNVEATSYALLALLAR.K
1880.9891	1879.9819	1880.0407	-31.29	1226 -	1242	0	80	K.LYNVEATSYALLALLAR.K
2157.0273	2156.0200	2156.0783	-27.04	979 -	998	0	---	K.ILLQGTPVAQMTEDAIDGER.L
2157.0273	2156.0201	2156.0783	-27.02	979 -	998	0	35	K.ILLQGTPVAQMTEDAIDGER.L
2506.2144	2505.2071	2505.2692	-24.80	1062 -	1083	0	---	K.SSAYAAFQYRPPSTWLTAYVVK.V
2506.2144	2505.2071	2505.2692	-24.79	1062 -	1083	0	---	K.SSAYAAFQYRPPSTWLTAYVVK.V
2598.2195	2597.2122	2597.2697	-22.12	1111 -	1133	0	---	K.QKPDGIFQEDGPVIHQEMIGGFR.D

No match to: 842.4847, 857.4026, 857.4026, 864.5186, 868.5277, 870.5200, 882.5434, 882.5434, 951.5951, 1017.6425, 1019.6487, 1033.6698, 1036.5013, 1036.5013, 1045.5382, 1051.6785, 1051.6785, 1107.5156, 1131.6732, 1136.6610, 1151.6952, 1165.5533, 1165.7053, 1179.5667, 1263.6616, 1277.6742, 1300.8134, 1334.8459, 1334.8459, 1379.6898, 1407.6674, 1434.8688, 1448.8787, 1475.7053, 1479.7522, 1482.7051, 1567.6991, 1618.0178, 1732.0514, 1808.9220, 1879.9926, 1890.9176, 1901.2008, 1993.9156, 2064.0874, 2098.0544, 2179.9844, 2211.0493, 2283.1272

8. A0A3Q1MHV6 **Mass:** 181738 **Score:** 231 **Expect:** 1.1e-018 **Matches:** 27

Complement C3 OS=Bos taurus OX=9913 GN=C3 PE=1 SV=1

Observed	Mr(expt)	Mr(calc)	ppm	Start	End	Miss	Ions	Peptide
845.4012	844.3939	844.3927	1.50	477 -	484	0	---	R.TDPGEQAK.I
850.5135	849.5062	849.4266	93.8	639 -	645	0	---	R.SVQLMEK.R + Oxidation (M)
864.5186	863.5113	863.4939	20.2	758 -	764	0	---	K.LMNVFLK.D
987.5186	986.5113	986.5396	-28.69	1011 -	1018	0	---	R.QESLELIR.K
996.5623	995.5550	995.4672	88.2	650 -	658	0	---	K.AGQYSSDLR.K
1083.5225	1082.5152	1082.5509	-32.98	1020 -	1028	0	14	K.GYTQQLAFR.Q
1083.5225	1082.5152	1082.5509	-32.94	1020 -	1028	0	---	K.GYTQQLAFR.Q
1090.4995	1089.4922	1089.4873	4.50	590 -	600	0	---	K.ADIGCTPGSGR.N
1124.5784	1123.5711	1123.5622	7.97	650 -	659	1	---	K.AGQYSSDLRK.C
1143.6165	1142.6092	1142.6407	-27.57	1010 -	1018	1	---	K.RQESLELIR.K
1162.5488	1161.5415	1161.5666	-21.61	1213 -	1222	0	---	K.DYDTTPPVVR.W
1200.5436	1199.5363	1199.5683	-26.65	1186 -	1194	1	---	K.NRWEEPNQK.L
1211.6163	1210.6090	1210.6458	-30.41	1019 -	1028	1	---	R.KGYTQQLAFR.Q
1211.6163	1210.6091	1210.6458	-30.39	1019 -	1028	1	---	R.KGYTQQLAFR.Q
1221.5270	1220.5197	1220.5574	-30.90	1140 -	1149	0	40	K.AGDFLENHYR.E
1221.5270	1220.5197	1220.5574	-30.88	1140 -	1149	0	---	K.AGDFLENHYR.E
1290.6333	1289.6260	1289.6616	-27.57	1212 -	1222	1	---	R.KDYDTTPPVVR.W
1361.6049	1360.5976	1360.6405	-31.51	1124 -	1135	0	---	K.DICEAQVNSLGR.S
1790.9982	1789.9909	1790.0454	-30.45	1153 -	1169	0	---	R.RPYTVAIAAYALALLGK.L
1790.9982	1789.9909	1790.0454	-30.43	1153 -	1169	0	---	R.RPYTVAIAAYALALLGK.L
1880.9891	1879.9818	1880.0407	-31.31	1195 -	1211	0	---	K.LYNVEATSYALLALLAR.K
1880.9891	1879.9819	1880.0407	-31.29	1195 -	1211	0	80	K.LYNVEATSYALLALLAR.K
2157.0273	2156.0200	2156.0783	-27.04	948 -	967	0	---	K.ILLQGTPVAQMTEDAIDGER.L
2157.0273	2156.0201	2156.0783	-27.02	948 -	967	0	35	K.ILLQGTPVAQMTEDAIDGER.L
2506.2144	2505.2071	2505.2692	-24.80	1031 -	1052	0	---	K.SSAYAAFQYRPPSTWLTAYVVK.V
2506.2144	2505.2071	2505.2692	-24.79	1031 -	1052	0	---	K.SSAYAAFQYRPPSTWLTAYVVK.V
2598.2195	2597.2122	2597.2697	-22.12	1080 -	1102	0	---	K.QKPDGIFQEDGPVIHQEMIGGFR.D

No match to: 842.4847, 848.4952, 853.4918, 857.4026, 857.4026, 868.5277, 870.5200, 882.5434, 882.5434, 951.5951, 1017.6425, 1019.6487, 1033.6698, 1036.5013, 1036.5013, 1045.5382, 1051.6785, 1051.6785, 1107.5156, 1131.6732, 1136.6610, 1151.6952, 1165.5533, 1165.7053, 1179.5667, 1263.6616, 1277.6742, 1300.8134, 1334.8459, 1334.8459, 1379.6898, 1407.6674, 1434.8688, 1448.8787, 1475.7053, 1479.7522, 1482.7051, 1567.6991, 1618.0178, 1732.0514, 1808.9220, 1879.9926, 1890.9176, 1901.2008, 1993.9156, 2064.0874, 2098.0544, 2179.9844, 2211.0493, 2283.1272

9. A0A4W2D3Y9 **Mass:** 185555 **Score:** 231 **Expect:** 1.1e-018 **Matches:** 28

Complement C3 OS=Bos indicus x Bos taurus OX=30522 GN=C3 PE=4 SV=1

Observed	Mr(expt)	Mr(calc)	ppm	Start	End	Miss	Ions	Peptide
845.4012	844.3939	844.3927	1.50	511 -	518	0	---	R.TDPGEQAK.I
848.4952	847.4879	847.4400	56.6	1422 -	1429	0	---	K.TLSTGVDR.Y
850.5135	849.5062	849.4266	93.8	673 -	679	0	---	R.SVQLMEK.R + Oxidation (M)
864.5186	863.5113	863.4939	20.2	792 -	798	0	---	K.LMNVFLK.D
987.5186	986.5113	986.5396	-28.69	1045 -	1052	0	---	R.QESLELIR.K
996.5623	995.5550	995.4672	88.2	684 -	692	0	---	K.AGQYSSDLR.K
1083.5225	1082.5152	1082.5509	-32.98	1054 -	1062	0	14	K.GYTQQLAFR.Q
1083.5225	1082.5152	1082.5509	-32.94	1054 -	1062	0	---	K.GYTQQLAFR.Q
1090.4995	1089.4922	1089.4873	4.50	624 -	634	0	---	K.ADIGCTPGSGR.N

PDF-XChange Click to buy NOW! www.docu-track.com

PDF-XChange Click to buy NOW! www.docu-track.com

1124.5784	1123.5711	1123.5622	7.97	684	693	1	---	K.AGQYSSDLRK.C
1143.6165	1142.6092	1142.6407	-27.57	1044	1052	1	---	K.RQESLELIR.K
1162.5488	1161.5415	1161.5666	-21.61	1247	1256	0	---	K.DYDTTPPVVR.W
1200.5436	1199.5363	1199.5683	-26.65	1220	1228	1	---	K.NRWEEPNQK.L
1211.6163	1210.6090	1210.6458	-30.41	1053	1062	1	---	R.KGYTQQLAFR.Q
1211.6163	1210.6091	1210.6458	-30.39	1053	1062	1	---	R.KGYTQQLAFR.Q
1221.5270	1220.5197	1220.5574	-30.90	1174	1183	0	40	K.AGDFLENHYR.E
1221.5270	1220.5197	1220.5574	-30.88	1174	1183	0	---	K.AGDFLENHYR.E
1290.6333	1289.6260	1289.6616	-27.57	1246	1256	1	---	R.KDYDTTPPVVR.W
1361.6049	1360.5976	1360.6405	-31.51	1158	1169	0	---	K.DICEAQVNSLGR.S
1790.9982	1789.9909	1790.0454	-30.45	1187	1203	0	---	R.RPYTVAIAAYALALLGK.L
1790.9982	1789.9909	1790.0454	-30.43	1187	1203	0	---	R.RPYTVAIAAYALALLGK.L
1880.9891	1879.9818	1880.0407	-31.31	1229	1245	0	---	K.LYNVEATSYALLALLAR.K
1880.9891	1879.9819	1880.0407	-31.29	1229	1245	0	80	K.LYNVEATSYALLALLAR.K
2157.0273	2156.0200	2156.0783	-27.04	982	1001	0	---	K.ILLQGTPVAQMTEDAIDGER.L
2157.0273	2156.0201	2156.0783	-27.02	982	1001	0	35	K.ILLQGTPVAQMTEDAIDGER.L
2506.2144	2505.2071	2505.2692	-24.80	1065	1086	0	---	K.SSAYAAFQYRPPSTWLTAYVVK.V
2506.2144	2505.2071	2505.2692	-24.79	1065	1086	0	---	K.SSAYAAFQYRPPSTWLTAYVVK.V
2598.2195	2597.2122	2597.2697	-22.12	1114	1136	0	---	K.QKPDGIFQEDGPVIHQEMIGGFR.D

No match to: 842.4847, 853.4918, 857.4026, 857.4026, 868.5277, 870.5200, 882.5434, 951.5951, 1017.6425, 1019.6487, 1033.6698, 1036.5013, 1036.5013, 1045.5382, 1051.6785, 1051.6785, 1107.5156, 1131.6732, 1136.6610, 1151.6952, 1165.5533, 1165.7053, 1179.5667, 1263.6616, 1277.6742, 1300.8134, 1334.8459, 1334.8459, 1379.6898, 1407.6674, 1434.8688, 1448.8787, 1475.7053, 1479.7522, 1482.7051, 1567.6991, 1618.0178, 1732.0514, 1808.9220, 1879.9926, 1890.9176, 1901.2008, 1993.9156, 2064.0874, 2098.0544, 2179.9844, 2211.0493, 2283.1272

10. A0A4W2EPV7 **Mass:** 179159 **Score:** 231 **Expect:** 1.1e-018 **Matches:** 27

Complement C3 OS=Bos indicus x Bos taurus OX=30522 GN=C3 PE=4 SV=1

Observed	Mr(expt)	Mr(calc)	ppm	Start	End	Miss	Ions	Peptide
845.4012	844.3939	844.3927	1.50	477	484	0	---	R.TDPGEQAK.I
848.4952	847.4879	847.4400	56.6	1355	1362	0	---	K.TLSTGVDR.Y
850.5135	849.5062	849.4266	93.8	639	645	0	---	R.SVQLMEK.R + Oxidation (M)
864.5186	863.5113	863.4939	20.2	758	764	0	---	K.LMNVFLK.D
987.5186	986.5113	986.5396	-28.69	1011	1018	0	---	R.QESLELIR.K
996.5623	995.5550	995.4672	88.2	650	658	0	---	K.AGQYSSDLR.K
1083.5225	1082.5152	1082.5509	-32.98	1020	1028	0	14	K.GYTQQLAFR.Q
1083.5225	1082.5152	1082.5509	-32.94	1020	1028	0	---	K.GYTQQLAFR.Q
1090.4995	1089.4922	1089.4873	4.50	590	600	0	---	K.ADIGCTPGSGR.N
1124.5784	1123.5711	1123.5622	7.97	650	659	1	---	K.AGQYSSDLRK.C
1143.6165	1142.6092	1142.6407	-27.57	1010	1018	1	---	K.RQESLELIR.K
1162.5488	1161.5415	1161.5666	-21.61	1180	1189	0	---	K.DYDTTPPVVR.W
1200.5436	1199.5363	1199.5683	-26.65	1153	1161	1	---	K.NRWEEPNQK.L
1211.6163	1210.6090	1210.6458	-30.41	1019	1028	1	---	R.KGYTQQLAFR.Q
1211.6163	1210.6091	1210.6458	-30.39	1019	1028	1	---	R.KGYTQQLAFR.Q
1221.5270	1220.5197	1220.5574	-30.90	1107	1116	0	40	K.AGDFLENHYR.E
1221.5270	1220.5197	1220.5574	-30.88	1107	1116	0	---	K.AGDFLENHYR.E
1290.6333	1289.6260	1289.6616	-27.57	1179	1189	1	---	R.KDYDTTPPVVR.W
1790.9982	1789.9909	1790.0454	-30.45	1120	1136	0	---	R.RPYTVAIAAYALALLGK.L
1790.9982	1789.9909	1790.0454	-30.43	1120	1136	0	---	R.RPYTVAIAAYALALLGK.L
1880.9891	1879.9818	1880.0407	-31.31	1162	1178	0	---	K.LYNVEATSYALLALLAR.K
1880.9891	1879.9819	1880.0407	-31.29	1162	1178	0	80	K.LYNVEATSYALLALLAR.K
2157.0273	2156.0200	2156.0783	-27.04	948	967	0	---	K.ILLQGTPVAQMTEDAIDGER.L
2157.0273	2156.0201	2156.0783	-27.02	948	967	0	35	K.ILLQGTPVAQMTEDAIDGER.L
2506.2144	2505.2071	2505.2692	-24.80	1031	1052	0	---	K.SSAYAAFQYRPPSTWLTAYVVK.V
2506.2144	2505.2071	2505.2692	-24.79	1031	1052	0	---	K.SSAYAAFQYRPPSTWLTAYVVK.V
2598.2195	2597.2122	2597.3085	-37.09	601	624	1	---	R.NYAGVFTDAGLTLKTSQGLETQQR.A

No match to: 842.4847, 853.4918, 857.4026, 857.4026, 868.5277, 870.5200, 882.5434, 882.5434, 951.5951, 1017.6425, 1019.6487, 1033.6698, 1036.5013, 1036.5013, 1045.5382, 1051.6785, 1051.6785, 1107.5156, 1131.6732, 1136.6610, 1151.6952, 1165.5533, 1165.7053, 1179.5667, 1263.6616, 1277.6742, 1300.8134, 1334.8459, 1334.8459, 1361.6049, 1379.6898, 1407.6674, 1434.8688, 1448.8787, 1475.7053, 1479.7522, 1482.7051, 1567.6991, 1618.0178, 1732.0514, 1808.9220, 1879.9926, 1890.9176, 1901.2008, 1993.9156, 2064.0874, 2098.0544, 2179.9844, 2211.0493, 2283.1272

11. A0A4W2D406 **Mass:** 186684 **Score:** 231 **Expect:** 1.1e-018 **Matches:** 28

Complement C3 OS=Bos indicus x Bos taurus OX=30522 GN=C3 PE=4 SV=1

Observed	Mr(expt)	Mr(calc)	ppm	Start	End	Miss	Ions	Peptide
845.4012	844.3939	844.3927	1.50	511	518	0	---	R.TDPGEQAK.I
848.4952	847.4879	847.4400	56.6	1422	1429	0	---	K.TLSTGVDR.Y
850.5135	849.5062	849.4266	93.8	673	679	0	---	R.SVQLMEK.R + Oxidation (M)
864.5186	863.5113	863.4939	20.2	792	798	0	---	K.LMNVFLK.D
987.5186	986.5113	986.5396	-28.69	1045	1052	0	---	R.QESLELIR.K
996.5623	995.5550	995.4672	88.2	684	692	0	---	K.AGQYSSDLR.K
1083.5225	1082.5152	1082.5509	-32.98	1054	1062	0	14	K.GYTQQLAFR.Q
1083.5225	1082.5152	1082.5509	-32.94	1054	1062	0	---	K.GYTQQLAFR.Q
1090.4995	1089.4922	1089.4873	4.50	624	634	0	---	K.ADIGCTPGSGR.N
1124.5784	1123.5711	1123.5622	7.97	684	693	1	---	K.AGQYSSDLRK.C
1143.6165	1142.6092	1142.6407	-27.57	1044	1052	1	---	K.RQESLELIR.K
1162.5488	1161.5415	1161.5666	-21.61	1247	1256	0	---	K.DYDTTPPVVR.W
1200.5436	1199.5363	1199.5683	-26.65	1220	1228	1	---	K.NRWEEPNQK.L
1211.6163	1210.6090	1210.6458	-30.41	1053	1062	1	---	R.KGYTQQLAFR.Q
1211.6163	1210.6091	1210.6458	-30.39	1053	1062	1	---	R.KGYTQQLAFR.Q
1221.5270	1220.5197	1220.5574	-30.90	1174	1183	0	40	K.AGDFLENHYR.E
1221.5270	1220.5197	1220.5574	-30.88	1174	1183	0	---	K.AGDFLENHYR.E
1290.6333	1289.6260	1289.6616	-27.57	1246	1256	1	---	R.KDYDTTPPVVR.W
1361.6049	1360.5976	1360.6405	-31.51	1158	1169	0	---	K.DICEAQVNSLGR.S
1790.9982	1789.9909	1790.0454	-30.45	1187	1203	0	---	R.RPYTVAIAAYALALLGK.L
1790.9982	1789.9909	1790.0454	-30.43	1187	1203	0	---	R.RPYTVAIAAYALALLGK.L
1880.9891	1879.9818	1880.0407	-31.31	1229	1245	0	---	K.LYNVEATSYALLALLAR.K
1880.9891	1879.9819	1880.0407	-31.29	1229	1245	0	80	K.LYNVEATSYALLALLAR.K
2157.0273	2156.0200	2156.0783	-27.04	982	1001	0	---	K.ILLQGTPVAQMTEDAIDGER.L
2157.0273	2156.0201	2156.0783	-27.02	982	1001	0	35	K.ILLQGTPVAQMTEDAIDGER.L
2506.2144	2505.2071	2505.2692	-24.80	1065	1086	0	---	K.SSAYAAFQYRPPSTWLTAYVVK.V
2506.2144	2505.2071	2505.2692	-24.79	1065	1086	0	---	K.SSAYAAFQYRPPSTWLTAYVVK.V
2598.2195	2597.2122	2597.2697	-22.12	1114	1136	0	---	K.QKPDGIFQEDGPVIHQEMIGGFR.D

PDF-XChange Click to buy NOW! www.docu-track.com

PDF-XChange Click to buy NOW! www.docu-track.com

No match to: 842.4847, 853.4918, 857.4026, 857.4026, 868.5277, 870.5200, 882.5434, 882.5434, 951.5951, 1017.6425, 1019.6487, 1033.6698, 1036.5013, 1036.5013, 1045.5382, 1051.6785, 1051.6785, 1107.5156, 1131.6732, 1136.6610, 1151.6952, 1165.5533, 1165.7053, 1179.5667, 1263.6616, 1277.6742, 1300.8134, 1334.8459, 1334.8459, 1379.6898, 1407.6674, 1434.8688, 1448.8787, 1475.7053, 1479.7522, 1482.7051, 1567.6991, 1618.0178, 1732.0514, 1808.9220, 1879.9926, 1890.9176, 1901.2008, 1993.9156, 2064.0874, 2098.0544, 2179.9844, 2211.0493, 2283.1272

12. A0A4W2D411 **Mass:** 190190 **Score:** 230 **Expect:** 1.3e-018 **Matches:** 28

Complement C3 OS=Bos indicus x Bos taurus OX=30522 GN=C3 PE=4 SV=1

Observed	Mr(expt)	Mr(calc)	ppm	Start	End	Miss	Ions	Peptide
845.4012	844.3939	844.3927	1.50	511 -	518	0	---	R.TDPGEQAK.I
848.4952	847.4879	847.4400	56.6	1453 -	1460	0	---	K.TLSTGVDR.Y
850.5135	849.5062	849.4266	93.8	704 -	710	0	---	R.SVQLMEK.R + Oxidation (M)
864.5186	863.5113	863.4939	20.2	823 -	829	0	---	K.LMNVFLK.D
987.5186	986.5113	986.5396	-28.69	1076 -	1083	0	---	R.QESLELIR.K
996.5623	995.5550	995.4672	88.2	715 -	723	0	---	K.AGQYSSDLR.K
1083.5225	1082.5152	1082.5509	-32.98	1085 -	1093	0	14	K.GYTQQLAFR.Q
1083.5225	1082.5152	1082.5509	-32.94	1085 -	1093	0	---	K.GYTQQLAFR.Q
1090.4995	1089.4922	1089.4873	4.50	655 -	665	0	---	K.ADIGCTPGSGR.N
1124.5784	1123.5711	1123.5622	7.97	715 -	724	1	---	K.AGQYSSDLRK.C
1143.6165	1142.6092	1142.6407	-27.57	1075 -	1083	1	---	K.RQESLELIR.K
1162.5488	1161.5415	1161.5666	-21.61	1278 -	1287	0	---	K.DYDTTPPVVR.W
1200.5436	1199.5363	1199.5683	-26.65	1251 -	1259	1	---	K.NRWEEPNQK.L
1211.6163	1210.6090	1210.6458	-30.41	1084 -	1093	1	---	R.KGYTQQLAFR.Q
1211.6163	1210.6091	1210.6458	-30.39	1084 -	1093	1	---	R.KGYTQQLAFR.Q
1221.5270	1220.5197	1220.5574	-30.90	1205 -	1214	0	40	K.AGDFLENHYR.E
1221.5270	1220.5197	1220.5574	-30.88	1205 -	1214	0	---	K.AGDFLENHYR.E
1290.6333	1289.6260	1289.6616	-27.57	1277 -	1287	1	---	R.KDYDTTPPVVR.W
1361.6049	1360.5976	1360.6405	-31.51	1189 -	1200	0	---	K.DICEAQVNSLGR.S
1790.9982	1789.9909	1790.0454	-30.45	1218 -	1234	0	---	R.RPYTVAIAAYALALLGK.L
1790.9982	1789.9909	1790.0454	-30.43	1218 -	1234	0	---	R.RPYTVAIAAYALALLGK.L
1880.9891	1879.9818	1880.0407	-31.31	1260 -	1276	0	---	K.LYNVEATSYALLALLAR.K
1880.9891	1879.9819	1880.0407	-31.29	1260 -	1276	0	80	K.LYNVEATSYALLALLAR.K
2157.0273	2156.0200	2156.0783	-27.04	1013 -	1032	0	---	K.ILLQGTPVAQMTEDAIDGER.L
2157.0273	2156.0201	2156.0783	-27.02	1013 -	1032	0	35	K.ILLQGTPVAQMTEDAIDGER.L
2506.2144	2505.2071	2505.2692	-24.80	1096 -	1117	0	---	K.SSAYAAFQYRPPSTWLTAYVVK.V
2506.2144	2505.2071	2505.2692	-24.79	1096 -	1117	0	---	K.SSAYAAFQYRPPSTWLTAYVVK.V
2598.2195	2597.2122	2597.2697	-22.12	1145 -	1167	0	---	K.QKPDGIFQEDGPVIHQEMIGGFR.D

No match to: 842.4847, 853.4918, 857.4026, 857.4026, 868.5277, 870.5200, 882.5434, 882.5434, 951.5951, 1017.6425, 1019.6487, 1033.6698, 1036.5013, 1036.5013, 1045.5382, 1051.6785, 1051.6785, 1107.5156, 1131.6732, 1136.6610, 1151.6952, 1165.5533, 1165.7053, 1179.5667, 1263.6616, 1277.6742, 1300.8134, 1334.8459, 1334.8459, 1379.6898, 1407.6674, 1434.8688, 1448.8787, 1475.7053, 1479.7522, 1482.7051, 1567.6991, 1618.0178, 1732.0514, 1808.9220, 1879.9926, 1890.9176, 1901.2008, 1993.9156, 2064.0874, 2098.0544, 2179.9844, 2211.0493, 2283.1272

13. A0A4W2CZN6 **Mass:** 192486 **Score:** 229 **Expect:** 1.7e-018 **Matches:** 28

Complement C3 OS=Bos indicus x Bos taurus OX=30522 GN=C3 PE=4 SV=1

Observed	Mr(expt)	Mr(calc)	ppm	Start	End	Miss	Ions	Peptide
845.4012	844.3939	844.3927	1.50	511 -	518	0	---	R.TDPGEQAK.I
848.4952	847.4879	847.4400	56.6	1453 -	1460	0	---	K.TLSTGVDR.Y
850.5135	849.5062	849.4266	93.8	704 -	710	0	---	R.SVQLMEK.R + Oxidation (M)
864.5186	863.5113	863.4939	20.2	823 -	829	0	---	K.LMNVFLK.D
987.5186	986.5113	986.5396	-28.69	1076 -	1083	0	---	R.QESLELIR.K
996.5623	995.5550	995.4672	88.2	715 -	723	0	---	K.AGQYSSDLR.K
1083.5225	1082.5152	1082.5509	-32.98	1085 -	1093	0	14	K.GYTQQLAFR.Q
1083.5225	1082.5152	1082.5509	-32.94	1085 -	1093	0	---	K.GYTQQLAFR.Q
1090.4995	1089.4922	1089.4873	4.50	655 -	665	0	---	K.ADIGCTPGSGR.N
1124.5784	1123.5711	1123.5622	7.97	715 -	724	1	---	K.AGQYSSDLRK.C
1143.6165	1142.6092	1142.6407	-27.57	1075 -	1083	1	---	K.RQESLELIR.K
1162.5488	1161.5415	1161.5666	-21.61	1278 -	1287	0	---	K.DYDTTPPVVR.W
1200.5436	1199.5363	1199.5683	-26.65	1251 -	1259	1	---	K.NRWEEPNQK.L
1211.6163	1210.6090	1210.6458	-30.41	1084 -	1093	1	---	R.KGYTQQLAFR.Q
1211.6163	1210.6091	1210.6458	-30.39	1084 -	1093	1	---	R.KGYTQQLAFR.Q
1221.5270	1220.5197	1220.5574	-30.90	1205 -	1214	0	40	K.AGDFLENHYR.E
1221.5270	1220.5197	1220.5574	-30.88	1205 -	1214	0	---	K.AGDFLENHYR.E
1290.6333	1289.6260	1289.6616	-27.57	1277 -	1287	1	---	R.KDYDTTPPVVR.W
1361.6049	1360.5976	1360.6405	-31.51	1189 -	1200	0	---	K.DICEAQVNSLGR.S
1790.9982	1789.9909	1790.0454	-30.45	1218 -	1234	0	---	R.RPYTVAIAAYALALLGK.L
1790.9982	1789.9909	1790.0454	-30.43	1218 -	1234	0	---	R.RPYTVAIAAYALALLGK.L
1880.9891	1879.9818	1880.0407	-31.31	1260 -	1276	0	---	K.LYNVEATSYALLALLAR.K
1880.9891	1879.9819	1880.0407	-31.29	1260 -	1276	0	80	K.LYNVEATSYALLALLAR.K
2157.0273	2156.0200	2156.0783	-27.04	1013 -	1032	0	---	K.ILLQGTPVAQMTEDAIDGER.L
2157.0273	2156.0201	2156.0783	-27.02	1013 -	1032	0	35	K.ILLQGTPVAQMTEDAIDGER.L
2506.2144	2505.2071	2505.2692	-24.80	1096 -	1117	0	---	K.SSAYAAFQYRPPSTWLTAYVVK.V
2506.2144	2505.2071	2505.2692	-24.79	1096 -	1117	0	---	K.SSAYAAFQYRPPSTWLTAYVVK.V
2598.2195	2597.2122	2597.2697	-22.12	1145 -	1167	0	---	K.QKPDGIFQEDGPVIHQEMIGGFR.D

No match to: 842.4847, 853.4918, 857.4026, 857.4026, 868.5277, 870.5200, 882.5434, 882.5434, 951.5951, 1017.6425, 1019.6487, 1033.6698, 1036.5013, 1036.5013, 1045.5382, 1051.6785, 1051.6785, 1107.5156, 1131.6732, 1136.6610, 1151.6952, 1165.5533, 1165.7053, 1179.5667, 1263.6616, 1277.6742, 1300.8134, 1334.8459, 1334.8459, 1379.6898, 1407.6674, 1434.8688, 1448.8787, 1475.7053, 1479.7522, 1482.7051, 1567.6991, 1618.0178, 1732.0514, 1808.9220, 1879.9926, 1890.9176, 1901.2008, 1993.9156, 2064.0874, 2098.0544, 2179.9844, 2211.0493, 2283.1272

14. A0A4W2EPI5 **Mass:** 182918 **Score:** 229 **Expect:** 1.7e-018 **Matches:** 27

Complement C3 OS=Bos indicus x Bos taurus OX=30522 GN=C3 PE=4 SV=1

Observed	Mr(expt)	Mr(calc)	ppm	Start	End	Miss	Ions	Peptide
845.4012	844.3939	844.3927	1.50	477 -	484	0	---	R.TDPGEQAK.I
850.5135	849.5062	849.4266	93.8	670 -	676	0	---	R.SVQLMEK.R + Oxidation (M)
864.5186	863.5113	863.4939	20.2	789 -	795	0	---	K.LMNVFLK.D
987.5186	986.5113	986.5396	-28.69	1042 -	1049	0	---	R.QESLELIR.K
996.5623	995.5550	995.4672	88.2	681 -	689	0	---	K.AGQYSSDLR.K
1083.5225	1082.5152	1082.5509	-32.98	1051 -	1059	0	14	K.GYTQQLAFR.Q
1083.5225	1082.5152	1082.5509	-32.94	1051 -	1059	0	---	K.GYTQQLAFR.Q
1090.4995	1089.4922	1089.4873	4.50	621 -	631	0	---	K.ADIGCTPGSGR.N
1124.5784	1123.5711	1123.5622	7.97	681 -	690	1	---	K.AGQYSSDLRK.C
1143.6165	1142.6092	1142.6407	-27.57	1041 -	1049	1	---	K.RQESLELIR.K
1162.5488	1161.5415	1161.5666	-21.61	1244 -	1253	0	---	K.DYDTTPPVVR.W

PDF-XChange Click to buy NOW! www.docu-track.com

1200.5436	1199.5363	1199.5683	-26.65	1217	1225	1	---	K.NRWEEPNQK.L
1211.6163	1210.6090	1210.6458	-30.41	1050	1059	1	---	R.KGYTQQLAFR.Q
1211.6163	1210.6091	1210.6458	-30.39	1050	1059	1	---	R.KGYTQQLAFR.Q
1221.5270	1220.5197	1220.5574	-30.90	1171	1180	0	40	K.AGDFLENHYR.E
1221.5270	1220.5197	1220.5574	-30.88	1171	1180	0	---	K.AGDFLENHYR.E
1290.6333	1289.6260	1289.6616	-27.57	1243	1253	1	---	R.KDYDTTPPVVR.W
1361.6049	1360.5976	1360.6405	-31.51	1155	1166	0	---	K.DICEAQVNSLGR.S
1790.9982	1789.9909	1790.0454	-30.45	1184	1200	0	---	R.RPYTVAIAAYALALLGK.L
1790.9982	1789.9909	1790.0454	-30.43	1184	1200	0	---	R.RPYTVAIAAYALALLGK.L
1880.9891	1879.9818	1880.0407	-31.31	1226	1242	0	---	K.LYNVEATSYALLALLAR.K
1880.9891	1879.9819	1880.0407	-31.29	1226	1242	0	80	K.LYNVEATSYALLALLAR.K
2157.0273	2156.0200	2156.0783	-27.04	979	998	0	---	K.ILLQGTPVAQMTEDAIDGER.L
2157.0273	2156.0201	2156.0783	-27.02	979	998	0	35	K.ILLQGTPVAQMTEDAIDGER.L
2506.2144	2505.2071	2505.2692	-24.80	1062	1083	0	---	K.SSAYAAFQYRPPSTWLTAYVVK.V
2506.2144	2505.2071	2505.2692	-24.79	1062	1083	0	---	K.SSAYAAFQYRPPSTWLTAYVVK.V
2598.2195	2597.2122	2597.2697	-22.12	1111	1133	0	---	K.QKPDGIFQEDGPVIHQEMIGGFR.D

No match to: 842.4847, 848.4952, 853.4918, 857.4026, 857.4026, 868.5277, 870.5200, 882.5434, 882.5434, 951.5951, 1017.6425, 1019.6487, 1033.6698, 1036.5013, 1036.5013, 1045.5382, 1051.6785, 1051.6785, 1107.5156, 1131.6732, 1136.6610, 1151.6952, 1165.5533, 1165.7053, 1179.5667, 1263.6616, 1277.6742, 1300.8134, 1334.8459, 1334.8459, 1379.6898, 1407.6674, 1434.8688, 1448.8787, 1475.7053, 1479.7522, 1482.7051, 1567.6991, 1618.0178, 1732.0514, 1808.9220, 1879.9926, 1890.9176, 1901.2008, 1993.9156, 2064.0874, 2098.0544, 2179.9844, 2211.0493, 2283.1272

15. A0A4W2HFJ5 **Mass:** 188690 **Score:** 98 **Expect:** 2.3e-005 **Matches:** 24

Complement C3 OS=Bos indicus x Bos taurus OX=30522 GN=C3 PE=4 SV=1

Observed	Mr(expt)	Mr(calc)	ppm	Start	End	Miss	Ions	Peptide
845.4012	844.3939	844.3927	1.50	477	484	0	---	R.TDPGEQAK.I
848.4952	847.4879	847.4400	56.6	1419	1426	0	---	K.TLSTGVDR.Y
850.5135	849.5062	849.4266	93.8	670	676	0	---	R.SVQLMEK.R + Oxidation (M)
864.5186	863.5113	863.4939	20.2	789	795	0	---	K.LMNVFLK.D
987.5186	986.5113	986.5396	-28.69	1042	1049	0	---	R.QESLELIR.K
996.5623	995.5550	995.4672	88.2	681	689	0	---	K.AGQYSSDLR.K
1083.5225	1082.5152	1082.5509	-32.98	1051	1059	0	14	K.GYTQQLAFR.Q
1083.5225	1082.5152	1082.5509	-32.94	1051	1059	0	---	K.GYTQQLAFR.Q
1090.4995	1089.4922	1089.4873	4.50	621	631	0	---	K.ADIGCTPGSGR.N
1124.5784	1123.5711	1123.5622	7.97	681	690	1	---	K.AGQYSSDLRK.C
1143.6165	1142.6092	1142.6407	-27.57	1041	1049	1	---	K.RQESLELIR.K
1162.5488	1161.5415	1161.5666	-21.61	1244	1253	0	---	K.DYDTTPPVVR.W
1200.5436	1199.5363	1199.5683	-26.65	1217	1225	1	---	K.NRWEEPNQK.L
1211.6163	1210.6090	1210.6458	-30.41	1050	1059	1	---	R.KGYTQQLAFR.Q
1211.6163	1210.6091	1210.6458	-30.39	1050	1059	1	---	R.KGYTQQLAFR.Q
1290.6333	1289.6260	1289.6616	-27.57	1243	1253	1	---	R.KDYDTTPPVVR.W
1361.6049	1360.5976	1360.6405	-31.51	1155	1166	0	---	K.DICEAQVNSLGR.S
1790.9982	1789.9909	1790.0454	-30.45	1184	1200	0	---	R.RPYTVAIAAYALALLGK.L
1790.9982	1789.9909	1790.0454	-30.43	1184	1200	0	---	R.RPYTVAIAAYALALLGK.L
2157.0273	2156.0200	2156.0783	-27.04	979	998	0	---	K.ILLQGTPVAQMTEDAIDGER.L
2157.0273	2156.0201	2156.0783	-27.02	979	998	0	35	K.ILLQGTPVAQMTEDAIDGER.L
2506.2144	2505.2071	2505.2692	-24.80	1062	1083	0	---	K.SSAYAAFQYRPPSTWLTAYVVK.V
2506.2144	2505.2071	2505.2692	-24.79	1062	1083	0	---	K.SSAYAAFQYRPPSTWLTAYVVK.V
2598.2195	2597.2122	2597.2697	-22.12	1111	1133	0	---	K.QKPDGIFQEDGPVIHQEMIGGFR.D

No match to: 842.4847, 853.4918, 857.4026, 857.4026, 868.5277, 870.5200, 882.5434, 882.5434, 951.5951, 1017.6425, 1019.6487, 1033.6698, 1036.5013, 1036.5013, 1045.5382, 1051.6785, 1051.6785, 1107.5156, 1131.6732, 1136.6610, 1151.6952, 1165.5533, 1165.7053, 1179.5667, 1221.5270, 1221.5270, 1263.6616, 1277.6742, 1300.8134, 1334.8459, 1334.8459, 1379.6898, 1407.6674, 1434.8688, 1448.8787, 1475.7053, 1479.7522, 1482.7051, 1567.6991, 1618.0178, 1732.0514, 1808.9220, 1879.9926, 1880.9891, 1880.9891, 1890.9176, 1901.2008, 1993.9156, 2064.0874, 2098.0544, 2179.9844, 2211.0493, 2283.1272

16. A0A4W2CZQ8 **Mass:** 170639 **Score:** 79 **Expect:** 0.0019 **Matches:** 18

Complement C3 OS=Bos indicus x Bos taurus OX=30522 GN=C3 PE=4 SV=1

Observed	Mr(expt)	Mr(calc)	ppm	Start	End	Miss	Ions	Peptide
845.4012	844.3939	844.3927	1.50	477	484	0	---	R.TDPGEQAK.I
848.4952	847.4879	847.4400	56.6	1281	1288	0	---	K.TLSTGVDR.Y
850.5135	849.5062	849.4266	93.8	639	645	0	---	R.SVQLMEK.R + Oxidation (M)
864.5186	863.5113	863.4939	20.2	758	764	0	---	K.LMNVFLK.D
987.5186	986.5113	986.5396	-28.69	1011	1018	0	---	R.QESLELIR.K
996.5623	995.5550	995.4672	88.2	650	658	0	---	K.AGQYSSDLR.K
1083.5225	1082.5152	1082.5509	-32.98	1020	1028	0	14	K.GYTQQLAFR.Q
1083.5225	1082.5152	1082.5509	-32.94	1020	1028	0	---	K.GYTQQLAFR.Q
1090.4995	1089.4922	1089.4873	4.50	590	600	0	---	K.ADIGCTPGSGR.N
1124.5784	1123.5711	1123.5622	7.97	650	659	1	---	K.AGQYSSDLRK.C
1143.6165	1142.6092	1142.6407	-27.57	1010	1018	1	---	K.RQESLELIR.K
1211.6163	1210.6090	1210.6458	-30.41	1019	1028	1	---	R.KGYTQQLAFR.Q
1211.6163	1210.6091	1210.6458	-30.39	1019	1028	1	---	R.KGYTQQLAFR.Q
2157.0273	2156.0200	2156.0783	-27.04	948	967	0	---	K.ILLQGTPVAQMTEDAIDGER.L
2157.0273	2156.0201	2156.0783	-27.02	948	967	0	35	K.ILLQGTPVAQMTEDAIDGER.L
2506.2144	2505.2071	2505.2692	-24.80	1031	1052	0	---	K.SSAYAAFQYRPPSTWLTAYVVK.V
2506.2144	2505.2071	2505.2692	-24.79	1031	1052	0	---	K.SSAYAAFQYRPPSTWLTAYVVK.V
2598.2195	2597.2122	2597.2697	-22.12	1080	1102	0	---	K.QKPDGIFQEDGPVIHQEMIGGFR.D

No match to: 842.4847, 853.4918, 857.4026, 857.4026, 868.5277, 870.5200, 882.5434, 882.5434, 951.5951, 1017.6425, 1019.6487, 1033.6698, 1036.5013, 1036.5013, 1045.5382, 1051.6785, 1051.6785, 1107.5156, 1131.6732, 1136.6610, 1151.6952, 1162.5488, 1165.5533, 1165.7053, 1179.5667, 1200.5436, 1221.5270, 1221.5270, 1263.6616, 1277.6742, 1290.6333, 1300.8134, 1334.8459, 1334.8459, 1361.6049, 1379.6898, 1407.6674, 1434.8688, 1448.8787, 1475.7053, 1479.7522, 1482.7051, 1567.6991, 1618.0178, 1732.0514, 1790.9982, 1790.9982, 1808.9220, 1879.9926, 1880.9891, 1880.9891, 1890.9176, 1901.2008, 1993.9156, 2064.0874, 2098.0544, 2179.9844, 2211.0493, 2283.1272

17. A0A4W2DYD9 **Mass:** 31930 **Score:** 54 **Expect:** 0.56 **Matches:** 12

Phosphatidylinositol transfer protein beta OS=Bos indicus x Bos taurus OX=30522 GN=PITPNB PE=4 SV=1

Observed	Mr(expt)	Mr(calc)	ppm	Start	End	Miss	Ions	Peptide
848.4952	847.4879	847.5531	-76.92	195	201	1	---	K.LVTIKFK.W
868.5277	867.5204	867.4603	69.3	171	178	0	---	R.GPLGPNWK.K
996.5623	995.5550	995.5552	-0.21	171	179	1	---	R.GPLGPNWKK.E
1036.5013	1035.4940	1035.4873	6.52	147	155	0	---	R.SQVEPADYK.A
1036.5013	1035.4941	1035.4873	6.56	147	155	0	---	R.SQVEPADYK.A
1051.6785	1050.6712	1050.5896	77.7	1	8	1	---	-.MVLIKEFR.V + Oxidation (M)
1051.6785	1050.6712	1050.5896	77.7	1	8	1	---	-.MVLIKEFR.V + Oxidation (M)
1090.4995	1089.4922	1089.5832	-83.49	219	226	1	---	K.RIFTNFHR.Q

PDF-XChange Click to buy NOW! www.docu-track.com

1162.5488	1161.5415	1161.5414	0.07	51	60	1	---	K.DGEKGQYTHK.I
1179.5667	1178.5594	1178.6237	-54.50	200	208	1	---	K.FKWWGLQSK.V
1200.5436	1199.5363	1199.5182	15.1	87	95	0	---	K.AWNAYPYCR.T
1879.9926	1878.9853	1878.9073	41.5	96	110	1	---	R.TIVTNEYMKDDFFIK.I + Oxidation (M)

No match to: 842.4847, 845.4012, 850.5135, 853.4918, 857.4026, 857.4026, 864.5186, 870.5200, 882.5434, 882.5434, 951.5951, 987.5186, 1017.6425, 1019.6487, 1033.6698, 1045.5382, 1083.5225, 1083.5225, 1107.5156, 1124.5784, 1131.6732, 1136.6610, 1143.6165, 1151.6952, 1165.5533, 1165.7053, 1211.6163, 1211.6163, 1221.5270, 1221.5270, 1263.6616, 1277.6742, 1290.6333, 1300.8134, 1334.8459, 1334.8459, 1361.6049, 1379.6898, 1407.6674, 1434.8688, 1448.8787, 1475.7053, 1479.7522, 1482.7051, 1567.6991, 1618.0178, 1732.0514, 1790.9982, 1790.9982, 1808.9220, 1880.9891, 1880.9891, 1890.9176, 1901.2008, 1993.9156, 2064.0874, 2098.0544, 2157.0273, 2157.0273, 2179.9844, 2211.0493, 2283.1272, 2506.2144, 2506.2144, 2598.2195

18. A0A4W2C513 **Mass:** 31804 **Score:** 53 **Expect:** 0.64 **Matches:** 12

Phosphatidylinositol transfer protein beta OS=Bos indicus x Bos taurus OX=30522 GN=PITPNB PE=4 SV=1

Observed	Mr(expt)	Mr(calc)	ppm	Start	End	Miss	Ions	Peptide
848.4952	847.4879	847.5531	-76.92	195	201	1	---	K.LVTIKFK.W
868.5277	867.5204	867.4603	69.3	171	178	0	---	R.GPLGPNWK.K
996.5623	995.5550	995.5552	-0.21	171	179	1	---	R.GPLGPNWKK.E
1036.5013	1035.4940	1035.4873	6.52	147	155	0	---	R.SQVEPADYK.A
1036.5013	1035.4941	1035.4873	6.56	147	155	0	---	R.SQVEPADYK.A
1051.6785	1050.6712	1050.5896	77.7	1	8	1	---	-.MVLIKEFR.V + Oxidation (M)
1051.6785	1050.6712	1050.5896	77.7	1	8	1	---	-.MVLIKEFR.V + Oxidation (M)
1090.4995	1089.4922	1089.5832	-83.49	219	226	1	---	K.RIFTNFHR.Q
1162.5488	1161.5415	1161.5414	0.07	51	60	1	---	K.DGEKGQYTHK.I
1179.5667	1178.5594	1178.6237	-54.50	200	208	1	---	K.FKWWGLQSK.V
1200.5436	1199.5363	1199.5182	15.1	87	95	0	---	K.AWNAYPYCR.T
1879.9926	1878.9853	1878.9073	41.5	96	110	1	---	R.TIVTNEYMKDDFFIK.I + Oxidation (M)

No match to: 842.4847, 845.4012, 850.5135, 853.4918, 857.4026, 857.4026, 864.5186, 870.5200, 882.5434, 882.5434, 951.5951, 987.5186, 1017.6425, 1019.6487, 1033.6698, 1045.5382, 1083.5225, 1083.5225, 1107.5156, 1124.5784, 1131.6732, 1136.6610, 1143.6165, 1151.6952, 1165.5533, 1165.7053, 1211.6163, 1211.6163, 1221.5270, 1221.5270, 1263.6616, 1277.6742, 1290.6333, 1300.8134, 1334.8459, 1334.8459, 1361.6049, 1379.6898, 1407.6674, 1434.8688, 1448.8787, 1475.7053, 1479.7522, 1482.7051, 1567.6991, 1618.0178, 1732.0514, 1790.9982, 1790.9982, 1808.9220, 1880.9891, 1880.9891, 1890.9176, 1901.2008, 1993.9156, 2064.0874, 2098.0544, 2157.0273, 2157.0273, 2179.9844, 2211.0493, 2283.1272, 2506.2144, 2506.2144, 2598.2195

19. Q9TR36 **Mass:** 31804 **Score:** 53 **Expect:** 0.64 **Matches:** 12

Phosphatidylinositol transfer protein beta isoform OS=Bos taurus OX=9913 GN=PITPNB PE=1 SV=3

Observed	Mr(expt)	Mr(calc)	ppm	Start	End	Miss	Ions	Peptide
848.4952	847.4879	847.5531	-76.92	195	201	1	---	K.LVTIKFK.W
868.5277	867.5204	867.4603	69.3	171	178	0	---	R.GPLGPNWK.K
996.5623	995.5550	995.5552	-0.21	171	179	1	---	R.GPLGPNWKK.E
1036.5013	1035.4940	1035.4873	6.52	147	155	0	---	R.SQVEPADYK.A
1036.5013	1035.4941	1035.4873	6.56	147	155	0	---	R.SQVEPADYK.A
1051.6785	1050.6712	1050.5896	77.7	1	8	1	---	-.MVLIKEFR.V + Oxidation (M)
1051.6785	1050.6712	1050.5896	77.7	1	8	1	---	-.MVLIKEFR.V + Oxidation (M)
1090.4995	1089.4922	1089.5832	-83.49	219	226	1	---	K.RIFTNFHR.Q
1162.5488	1161.5415	1161.5414	0.07	51	60	1	---	K.DGEKGQYTHK.I
1179.5667	1178.5594	1178.6237	-54.50	200	208	1	---	K.FKWWGLQSK.V
1200.5436	1199.5363	1199.5182	15.1	87	95	0	---	K.AWNAYPYCR.T
1879.9926	1878.9853	1878.9073	41.5	96	110	1	---	R.TIVTNEYMKDDFFIK.I + Oxidation (M)

No match to: 842.4847, 845.4012, 850.5135, 853.4918, 857.4026, 857.4026, 864.5186, 870.5200, 882.5434, 882.5434, 951.5951, 987.5186, 1017.6425, 1019.6487, 1033.6698, 1045.5382, 1083.5225, 1083.5225, 1107.5156, 1124.5784, 1131.6732, 1136.6610, 1143.6165, 1151.6952, 1165.5533, 1165.7053, 1211.6163, 1211.6163, 1221.5270, 1221.5270, 1263.6616, 1277.6742, 1290.6333, 1300.8134, 1334.8459, 1334.8459, 1361.6049, 1379.6898, 1407.6674, 1434.8688, 1448.8787, 1475.7053, 1479.7522, 1482.7051, 1567.6991, 1618.0178, 1732.0514, 1790.9982, 1790.9982, 1808.9220, 1880.9891, 1880.9891, 1890.9176, 1901.2008, 1993.9156, 2064.0874, 2098.0544, 2157.0273, 2157.0273, 2179.9844, 2211.0493, 2283.1272, 2506.2144, 2506.2144, 2598.2195

20. A0A4W2FGB2 **Mass:** 205135 **Score:** 53 **Expect:** 0.67 **Matches:** 18

Poly [ADP-ribose] polymerase OS=Bos indicus x Bos taurus OX=30522 GN=PARP14 PE=4 SV=1

Observed	Mr(expt)	Mr(calc)	ppm	Start	End	Miss	Ions	Peptide
850.5135	849.5062	849.5324	-30.79	1752	1759	0	---	R.SLIVPPPK.D
882.5434	881.5361	881.4494	98.4	1580	1587	0	---	K.TYIATDAK.G
882.5434	881.5361	881.4494	98.4	1580	1587	0	---	K.TYIATDAK.G
996.5623	995.5550	995.5011	54.2	1734	1740	1	---	K.KHMYYVR.V
1124.5784	1123.5711	1123.5509	18.0	385	394	0	---	K.DASTLFSDIR.S
1143.6165	1142.6092	1142.6295	-17.75	1052	1061	1	---	K.AGQKLQEELK.T
1162.5488	1161.5415	1161.5699	-24.47	1549	1558	0	---	K.ITNMQLEDAK.K
1200.5436	1199.5363	1199.6121	-63.16	29	37	1	---	K.LQMYFQSRK.R
1211.6163	1210.6090	1210.5618	39.0	73	82	0	---	R.ESHELEWPGK.G
1211.6163	1210.6091	1210.5618	39.0	73	82	0	---	R.ESHELEWPGK.G
1290.6333	1289.6260	1289.6146	8.85	1264	1274	1	---	R.AGRNVEIECSR.Q
1407.6674	1406.6601	1406.7340	-52.53	1420	1431	1	---	K.KTELAVFQVCGR.N
1434.8688	1433.8615	1433.8103	35.7	1295	1307	1	---	K.NIIHVIGGNDVKR.S
1880.9891	1879.9818	1879.9527	15.5	1237	1253	1	---	K.EEADVIVNSTSKSFNLK.A
1880.9891	1879.9819	1879.9527	15.5	1237	1253	1	---	K.EEADVIVNSTSKSFNLK.A
2157.0273	2156.0200	2156.1478	-59.24	83	101	1	---	K.GTFKLTVQIPTVPDEVQER.K
2157.0273	2156.0201	2156.1478	-59.22	83	101	1	26	K.GTFKLTVQIPTVPDEVQER.K
2211.0493	2210.0420	2210.2271	-83.72	1056	1076	1	---	K.LQEELKTAGQGVVVDVGTVLR.T

No match to: 842.4847, 845.4012, 848.4952, 853.4918, 857.4026, 857.4026, 864.5186, 868.5277, 870.5200, 951.5951, 987.5186, 1017.6425, 1019.6487, 1033.6698, 1036.5013, 1036.5013, 1045.5382, 1051.6785, 1051.6785, 1083.5225, 1083.5225, 1090.4995, 1107.5156, 1131.6732, 1136.6610, 1151.6952, 1165.5533, 1165.7053, 1179.5667, 1221.5270, 1221.5270, 1263.6616, 1277.6742, 1300.8134, 1334.8459, 1334.8459, 1361.6049, 1379.6898, 1448.8787, 1475.7053, 1479.7522, 1482.7051, 1567.6991, 1618.0178, 1732.0514, 1790.9982, 1790.9982, 1808.9220, 1879.9926, 1890.9176, 1901.2008, 1993.9156, 2064.0874, 2098.0544, 2179.9844, 2283.1272, 2506.2144, 2506.2144, 2598.2195

21. A0A4W2EP89 **Mass:** 190854 **Score:** 50 **Expect:** 1.4 **Matches:** 17

Poly(ADP-ribose) polymerase family member 14 OS=Bos indicus x Bos taurus OX=30522 GN=PARP14 PE=4 SV=1

Observed	Mr(expt)	Mr(calc)	ppm	Start	End	Miss	Ions	Peptide
882.5434	881.5361	881.4494	98.4	1580	1587	0	---	K.TYIATDAK.G
882.5434	881.5361	881.4494	98.4	1580	1587	0	---	K.TYIATDAK.G
1124.5784	1123.5711	1123.5509	18.0	385	394	0	---	K.DASTLFSDIR.S
1143.6165	1142.6092	1142.6295	-17.75	1052	1061	1	---	K.AGQKLQEELK.T
1162.5488	1161.5415	1161.5699	-24.47	1549	1558	0	---	K.ITNMQLEDAK.K
1179.5667	1178.5594	1178.5689	-8.01	1663	1671	1	---	K.TVWMPRMAR.- + 2 Oxidation (M)
1200.5436	1199.5363	1199.6121	-63.16	29	37	1	---	K.LQMYFQSRK.R
1211.6163	1210.6090	1210.5618	39.0	73	82	0	---	R.ESHELEWPGK.G
1211.6163	1210.6091	1210.5618	39.0	73	82	0	---	R.ESHELEWPGK.G

PDF-XChange Click to buy NOW! www.docu-track.com

PDF-XChange Click to buy NOW! www.docu-track.com

1290.6333	1289.6260	1289.6146	8.85	1264	1274	1	---	R.AGRNVEIECSR.Q
1407.6674	1406.6601	1406.7340	-52.53	1420	1431	1	---	K.KTELAVFQVCGR.N
1434.8688	1433.8615	1433.8103	35.7	1295	1307	1	---	K.NIIHVIGGNDVKR.S
1880.9891	1879.9818	1879.9527	15.5	1237	1253	1	---	K.EEADVIVNSTSKSFNLK.A
1880.9891	1879.9819	1879.9527	15.5	1237	1253	1	---	K.EEADVIVNSTSKSFNLK.A
2157.0273	2156.0200	2156.1478	-59.24	83	101	1	---	K.GTFKLTVQIPTVPDEVQER.K
2157.0273	2156.0201	2156.1478	-59.22	83	101	1	26	K.GTFKLTVQIPTVPDEVQER.K
2211.0493	2210.0420	2210.2271	-83.72	1056	1076	1	---	K.LQEELKTAGQGVVVDVGTVLR.T

No match to: 842.4847, 845.4012, 848.4952, 850.5135, 853.4918, 857.4026, 857.4026, 864.5186, 868.5277, 870.5200, 951.5951, 987.5186, 996.5623, 1017.6425, 1019.6487, 1033.6698, 1036.5013, 1036.5013, 1045.5382, 1051.6785, 1051.6785, 1083.5225, 1083.5225, 1090.4995, 1107.5156, 1131.6732, 1136.6610, 1151.6952, 1165.5533, 1165.7053, 1221.5270, 1221.5270, 1263.6616, 1277.6742, 1300.8134, 1334.8459, 1334.8459, 1361.6049, 1379.6898, 1448.8787, 1475.7053, 1479.7522, 1482.7051, 1567.6991, 1618.0178, 1732.0514, 1790.9982, 1790.9982, 1808.9220, 1879.9926, 1890.9176, 1901.2008, 1993.9156, 2064.0874, 2098.0544, 2179.9844, 2283.1272, 2506.2144, 2506.2144, 2598.2195

22. F1ML90 **Mass:** 204793 **Score:** 49 **Expect:** 1.5 **Matches:** 17

Poly [ADP-ribose] polymerase OS=Bos taurus OX=9913 GN=PARP14 PE=4 SV=3

Observed	Mr(expt)	Mr(calc)	ppm	Start	End	Miss	Ions	Peptide
882.5434	881.5361	881.4494	98.4	1578	1585	0	---	K.TYIATDAK.G
882.5434	881.5361	881.4494	98.4	1578	1585	0	---	K.TYIATDAK.G
996.5623	995.5550	995.5011	54.2	1732	1738	1	---	K.KHMYYVR.V
1124.5784	1123.5711	1123.5509	18.0	383	392	0	---	K.DASTLFSDIR.S
1143.6165	1142.6092	1142.6295	-17.75	1050	1059	1	---	K.AGQKLQEELK.T
1162.5488	1161.5415	1161.5699	-24.47	1547	1556	0	---	K.ITNMQLEDAK.K
1200.5436	1199.5363	1199.6121	-63.16	27	35	1	---	K.LQMYFQSRK.R
1211.6163	1210.6090	1210.5618	39.0	71	80	0	---	R.ESHELEWPGK.G
1211.6163	1210.6091	1210.5618	39.0	71	80	0	---	R.ESHELEWPGK.G
1290.6333	1289.6260	1289.6146	8.85	1262	1272	1	---	R.AGRNVEIECSR.Q
1407.6674	1406.6601	1406.7340	-52.53	1418	1429	1	---	K.KTELAVFQVCGR.N
1434.8688	1433.8615	1433.8103	35.7	1293	1305	1	---	K.NIIHVIGGNDVKR.S
1880.9891	1879.9818	1879.9527	15.5	1235	1251	1	---	K.EEADVIVNSTSKSFNLK.A
1880.9891	1879.9819	1879.9527	15.5	1235	1251	1	---	K.EEADVIVNSTSKSFNLK.A
2157.0273	2156.0200	2156.1478	-59.24	81	99	1	---	K.GTFKLTVQIPTVPDEVQER.K
2157.0273	2156.0201	2156.1478	-59.22	81	99	1	26	K.GTFKLTVQIPTVPDEVQER.K
2211.0493	2210.0420	2210.2271	-83.72	1054	1074	1	---	K.LQEELKTAGQGVVVDVGTVLR.T

No match to: 842.4847, 845.4012, 848.4952, 850.5135, 853.4918, 857.4026, 857.4026, 864.5186, 868.5277, 870.5200, 951.5951, 987.5186, 1017.6425, 1019.6487, 1033.6698, 1036.5013, 1036.5013, 1045.5382, 1051.6785, 1051.6785, 1083.5225, 1083.5225, 1090.4995, 1107.5156, 1131.6732, 1136.6610, 1151.6952, 1165.5533, 1165.7053, 1179.5667, 1221.5270, 1221.5270, 1263.6616, 1277.6742, 1300.8134, 1334.8459, 1334.8459, 1361.6049, 1379.6898, 1448.8787, 1475.7053, 1479.7522, 1482.7051, 1567.6991, 1618.0178, 1732.0514, 1790.9982, 1790.9982, 1808.9220, 1879.9926, 1890.9176, 1901.2008, 1993.9156, 2064.0874, 2098.0544, 2179.9844, 2283.1272, 2506.2144, 2506.2144, 2598.2195

23. A0A4W2CYS7 **Mass:** 190256 **Score:** 48 **Expect:** 2 **Matches:** 16

Poly(ADP-ribose) polymerase family member 14 OS=Bos indicus x Bos taurus OX=30522 GN=PARP14 PE=4 SV=1

Observed	Mr(expt)	Mr(calc)	ppm	Start	End	Miss	Ions	Peptide
882.5434	881.5361	881.4494	98.4	1580	1587	0	---	K.TYIATDAK.G
882.5434	881.5361	881.4494	98.4	1580	1587	0	---	K.TYIATDAK.G
1124.5784	1123.5711	1123.5509	18.0	385	394	0	---	K.DASTLFSDIR.S
1143.6165	1142.6092	1142.6295	-17.75	1052	1061	1	---	K.AGQKLQEELK.T
1162.5488	1161.5415	1161.5699	-24.47	1549	1558	0	---	K.ITNMQLEDAK.K
1200.5436	1199.5363	1199.6121	-63.16	29	37	1	---	K.LQMYFQSRK.R
1211.6163	1210.6090	1210.5618	39.0	73	82	0	---	R.ESHELEWPGK.G
1211.6163	1210.6091	1210.5618	39.0	73	82	0	---	R.ESHELEWPGK.G
1290.6333	1289.6260	1289.6146	8.85	1264	1274	1	---	R.AGRNVEIECSR.Q
1407.6674	1406.6601	1406.7340	-52.53	1420	1431	1	---	K.KTELAVFQVCGR.N
1434.8688	1433.8615	1433.8103	35.7	1295	1307	1	---	K.NIIHVIGGNDVKR.S
1880.9891	1879.9818	1879.9527	15.5	1237	1253	1	---	K.EEADVIVNSTSKSFNLK.A
1880.9891	1879.9819	1879.9527	15.5	1237	1253	1	---	K.EEADVIVNSTSKSFNLK.A
2157.0273	2156.0200	2156.1478	-59.24	83	101	1	---	K.GTFKLTVQIPTVPDEVQER.K
2157.0273	2156.0201	2156.1478	-59.22	83	101	1	26	K.GTFKLTVQIPTVPDEVQER.K
2211.0493	2210.0420	2210.2271	-83.72	1056	1076	1	---	K.LQEELKTAGQGVVVDVGTVLR.T

No match to: 842.4847, 845.4012, 848.4952, 850.5135, 853.4918, 857.4026, 857.4026, 864.5186, 868.5277, 870.5200, 951.5951, 987.5186, 996.5623, 1017.6425, 1019.6487, 1033.6698, 1036.5013, 1036.5013, 1045.5382, 1051.6785, 1051.6785, 1083.5225, 1083.5225, 1090.4995, 1107.5156, 1131.6732, 1136.6610, 1151.6952, 1165.5533, 1165.7053, 1179.5667, 1221.5270, 1221.5270, 1263.6616, 1277.6742, 1300.8134, 1334.8459, 1334.8459, 1361.6049, 1379.6898, 1448.8787, 1475.7053, 1479.7522, 1482.7051, 1567.6991, 1618.0178, 1732.0514, 1790.9982, 1790.9982, 1808.9220, 1879.9926, 1890.9176, 1901.2008, 1993.9156, 2064.0874, 2098.0544, 2179.9844, 2283.1272, 2506.2144, 2506.2144, 2598.2195

24. A0A3Q1MDH0 **Mass:** 196044 **Score:** 44 **Expect:** 5.2 **Matches:** 17

Poly [ADP-ribose] polymerase OS=Bos taurus OX=9913 GN=PARP14 PE=4 SV=1

Observed	Mr(expt)	Mr(calc)	ppm	Start	End	Miss	Ions	Peptide
882.5434	881.5361	881.4494	98.4	1501	1508	0	---	K.TYIATDAK.G
882.5434	881.5361	881.4494	98.4	1501	1508	0	---	K.TYIATDAK.G
996.5623	995.5550	995.5011	54.2	1655	1661	1	---	K.KHMYYVR.V
1124.5784	1123.5711	1123.5509	18.0	306	315	0	---	K.DASTLFSDIR.S
1143.6165	1142.6092	1142.6295	-17.75	973	982	1	---	K.AGQKLQEELK.T
1162.5488	1161.5415	1161.5699	-24.47	1470	1479	0	---	K.ITNMQLEDAK.K
1200.5436	1199.5363	1199.6121	-63.16	29	37	1	---	K.LQMYFQSRK.R
1211.6163	1210.6090	1210.5618	39.0	73	82	0	---	R.ESHELEWPGK.G
1211.6163	1210.6091	1210.5618	39.0	73	82	0	---	R.ESHELEWPGK.G
1290.6333	1289.6260	1289.6146	8.85	1185	1195	1	---	R.AGRNVEIECSR.Q
1407.6674	1406.6601	1406.7340	-52.53	1341	1352	1	---	K.KTELAVFQVCGR.N
1434.8688	1433.8615	1433.8103	35.7	1216	1228	1	---	K.NIIHVIGGNDVKR.S
1880.9891	1879.9818	1879.9527	15.5	1158	1174	1	---	K.EEADVIVNSTSKSFNLK.A
1880.9891	1879.9819	1879.9527	15.5	1158	1174	1	---	K.EEADVIVNSTSKSFNLK.A
2157.0273	2156.0200	2156.1478	-59.24	83	101	1	---	K.GTFKLTVQIPTVPDEVQER.K
2157.0273	2156.0201	2156.1478	-59.22	83	101	1	26	K.GTFKLTVQIPTVPDEVQER.K
2211.0493	2210.0420	2210.2271	-83.72	977	997	1	---	K.LQEELKTAGQGVVVDVGTVLR.T

No match to: 842.4847, 845.4012, 848.4952, 850.5135, 853.4918, 857.4026, 857.4026, 864.5186, 868.5277, 870.5200, 951.5951, 987.5186, 1017.6425, 1019.6487, 1033.6698, 1036.5013, 1036.5013, 1045.5382, 1051.6785, 1051.6785, 1083.5225, 1083.5225, 1090.4995, 1107.5156, 1131.6732, 1136.6610, 1151.6952, 1165.5533, 1165.7053, 1179.5667, 1221.5270, 1221.5270, 1263.6616, 1277.6742, 1300.8134, 1334.8459, 1334.8459, 1361.6049, 1379.6898, 1448.8787, 1475.7053, 1479.7522, 1482.7051, 1567.6991, 1618.0178, 1732.0514, 1790.9982, 1790.9982, 1808.9220, 1879.9926, 1890.9176, 1901.2008, 1993.9156, 2064.0874, 2098.0544, 2179.9844, 2283.1272, 2506.2144, 2506.2144, 2598.2195

PDF-XChange Click to buy NOW! www.docu-track.com

PDF-XChange Click to buy NOW! www.docu-track.com

[illegible]. A0A4W2E5T5 **Mass:** 31943 **Score:** 44 **Expect:** 5.6 **Matches:** 10
Phosphatidylinositol transfer protein beta OS=Bos indicus x Bos taurus OX=30522 GN=PITPNB PE=4 SV=1

Observed	Mr(expt)	Mr(calc)	ppm	Start	End	Miss	Ions	Peptide
848.4952	847.4879	847.5531	-76.92	197	203	1	---	K.LVTIKFK.W
868.5277	867.5204	867.4603	69.3	173	180	0	---	R.GPLGPNWK.K
996.5623	995.5550	995.5552	-0.21	173	181	1	---	R.GPLGPNWKK.E
1036.5013	1035.4940	1035.4873	6.52	149	157	0	---	R.SQVEPADYK.A
1036.5013	1035.4941	1035.4873	6.56	149	157	0	---	R.SQVEPADYK.A
1090.4995	1089.4922	1089.5832	-83.49	221	228	1	---	K.RIFTNFHR.Q
1162.5488	1161.5415	1161.5414	0.07	53	62	1	---	K.DGEKGQYTHK.I
1179.5667	1178.5594	1178.6237	-54.50	202	210	1	---	K.FKWWGLQSK.V
1200.5436	1199.5363	1199.5182	15.1	89	97	0	---	K.AWNAYPYCR.T
1879.9926	1878.9853	1878.9073	41.5	98	112	1	---	R.TIVTNEYMKDDFFIK.I + Oxidation (M)

No match to: 842.4847, 845.4012, 850.5135, 853.4918, 857.4026, 857.4026, 864.5186, 870.5200, 882.5434, 882.5434, 951.5951, 987.5186, 1017.6425, 1019.6487, 1033.6698, 1045.5382, 1051.6785, 1051.6785, 1083.5225, 1083.5225, 1107.5156, 1124.5784, 1131.6732, 1136.6610, 1143.6165, 1151.6952, 1165.5533, 1165.7053, 1211.6163, 1211.6163, 1221.5270, 1221.5270, 1263.6616, 1277.6742, 1290.6333, 1300.8134, 1334.8459, 1334.8459, 1361.6049, 1379.6898, 1407.6674, 1434.8688, 1448.8787, 1475.7053, 1479.7522, 1482.7051, 1567.6991, 1618.0178, 1732.0514, 1790.9982, 1790.9982, 1808.9220, 1880.9891, 1880.9891, 1890.9176, 1901.2008, 1993.9156, 2064.0874, 2098.0544, 2157.0273, 2157.0273, 2179.9844, 2211.0493, 2283.1272, 2506.2144, 2506.2144, 2598.2195

26. A0A452DJF3 **Mass:** 31845 **Score:** 43 **Expect:** 7.1 **Matches:** 11
Phosphatidylinositol transfer protein beta isoform OS=Bos taurus OX=9913 GN=PITPNB PE=4 SV=1

Observed	Mr(expt)	Mr(calc)	ppm	Start	End	Miss	Ions	Peptide
848.4952	847.4879	847.5531	-76.92	195	201	1	---	K.LVTIKFK.W
868.5277	867.5204	867.4603	69.3	171	178	0	---	R.GPLGPNWK.K
996.5623	995.5550	995.5552	-0.21	171	179	1	---	R.GPLGPNWKK.E
1036.5013	1035.4940	1035.4873	6.52	147	155	0	---	R.SQVEPADYK.A
1036.5013	1035.4941	1035.4873	6.56	147	155	0	---	R.SQVEPADYK.A
1051.6785	1050.6712	1050.5896	77.7	1	8	1	---	-.MVLIKEFR.V + Oxidation (M)
1051.6785	1050.6712	1050.5896	77.7	1	8	1	---	-.MVLIKEFR.V + Oxidation (M)
1090.4995	1089.4922	1089.5832	-83.49	219	226	1	---	K.RIFTNFHR.Q
1162.5488	1161.5415	1161.5414	0.07	51	60	1	---	K.DGEKGQYTHK.I
1179.5667	1178.5594	1178.6237	-54.50	200	208	1	---	K.FKWWGLQSK.V
1200.5436	1199.5363	1199.5182	15.1	87	95	0	---	K.AWNAYPYCR.T

No match to: 842.4847, 845.4012, 850.5135, 853.4918, 857.4026, 857.4026, 864.5186, 870.5200, 882.5434, 882.5434, 951.5951, 987.5186, 1017.6425, 1019.6487, 1033.6698, 1045.5382, 1083.5225, 1083.5225, 1107.5156, 1124.5784, 1131.6732, 1136.6610, 1143.6165, 1151.6952, 1165.5533, 1165.7053, 1211.6163, 1211.6163, 1221.5270, 1221.5270, 1263.6616, 1277.6742, 1290.6333, 1300.8134, 1334.8459, 1334.8459, 1361.6049, 1379.6898, 1407.6674, 1434.8688, 1448.8787, 1475.7053, 1479.7522, 1482.7051, 1567.6991, 1618.0178, 1732.0514, 1790.9982, 1790.9982, 1808.9220, 1879.9926, 1880.9891, 1880.9891, 1890.9176, 1901.2008, 1993.9156, 2064.0874, 2098.0544, 2157.0273, 2157.0273, 2179.9844, 2211.0493, 2283.1272, 2506.2144, 2506.2144, 2598.2195

27. A0A4W2DWE1 **Mass:** 8549 **Score:** 41 **Expect:** 10 **Matches:** 5
CD3e molecule OS=Bos indicus x Bos taurus OX=30522 GN=CD3E PE=4 SV=1

Observed	Mr(expt)	Mr(calc)	ppm	Start	End	Miss	Ions	Peptide
848.4952	847.4879	847.4552	38.6	20	26	1	---	K.DPRGIYK.C
1107.5156	1106.5083	1106.5356	-24.68	10	19	0	---	K.NTWNLGSSTK.D
1162.5488	1161.5415	1161.5051	31.4	63	71	0	---	R.EDDQYSHLR.K
1290.6333	1289.6260	1289.6000	20.2	63	72	1	---	R.EDDQYSHLRK.N
1475.7053	1474.6980	1474.7164	-12.49	10	22	1	---	K.NTWNLGSSTKDPR.G

No match to: 842.4847, 845.4012, 850.5135, 853.4918, 857.4026, 857.4026, 864.5186, 868.5277, 870.5200, 882.5434, 882.5434, 951.5951, 987.5186, 996.5623, 1017.6425, 1019.6487, 1033.6698, 1036.5013, 1036.5013, 1045.5382, 1051.6785, 1051.6785, 1083.5225, 1083.5225, 1090.4995, 1124.5784, 1131.6732, 1136.6610, 1143.6165, 1151.6952, 1165.5533, 1165.7053, 1179.5667, 1200.5436, 1211.6163, 1211.6163, 1221.5270, 1221.5270, 1263.6616, 1277.6742, 1300.8134, 1334.8459, 1334.8459, 1361.6049, 1379.6898, 1407.6674, 1434.8688, 1448.8787, 1479.7522, 1482.7051, 1567.6991, 1618.0178, 1732.0514, 1790.9982, 1790.9982, 1808.9220, 1879.9926, 1880.9891, 1880.9891, 1890.9176, 1901.2008, 1993.9156, 2064.0874, 2098.0544, 2157.0273, 2157.0273, 2179.9844, 2211.0493, 2283.1272, 2506.2144, 2506.2144, 2598.2195

28. A0A3Q1MWW7 **Mass:** 28595 **Score:** 40 **Expect:** 13 **Matches:** 7
Electron transfer flavoprotein subunit beta lysine methyltransferase OS=Bos taurus OX=9913 GN=ETFBKMT PE=4 SV=1

Observed	Mr(expt)	Mr(calc)	ppm	Start	End	Miss	Ions	Peptide
868.5277	867.5204	867.5654	-51.84	58	64	1	---	R.LRLLTPR.C
870.5200	869.5127	869.4759	42.3	72	78	0	---	R.ADLWPLR.D
1162.5488	1161.5415	1161.6064	-55.81	27	37	1	---	R.GTGSLLDPKMK.A + Oxidation (M)
1879.9926	1878.9853	1878.9053	42.6	79	94	0	---	R.DPYWAIYWPGGQALSR.Y
2157.0273	2156.0200	2156.0527	-15.15	9	26	1	---	R.AVRSSGFPLFPWGHCPWR.G
2157.0273	2156.0201	2156.0527	-15.13	9	26	1	7	R.AVRSSGFPLFPWGHCPWR.G
2179.9844	2178.9771	2179.0644	-40.07	38	57	0	---	K.AFLEENTEVTSSGSLTPEIR.L

No match to: 842.4847, 845.4012, 848.4952, 850.5135, 853.4918, 857.4026, 857.4026, 864.5186, 882.5434, 882.5434, 951.5951, 987.5186, 996.5623, 1017.6425, 1019.6487, 1033.6698, 1036.5013, 1036.5013, 1045.5382, 1051.6785, 1051.6785, 1083.5225, 1083.5225, 1090.4995, 1107.5156, 1124.5784, 1131.6732, 1136.6610, 1143.6165, 1151.6952, 1165.5533, 1165.7053, 1179.5667, 1200.5436, 1211.6163, 1211.6163, 1221.5270, 1221.5270, 1263.6616, 1277.6742, 1290.6333, 1300.8134, 1334.8459, 1334.8459, 1361.6049, 1379.6898, 1407.6674, 1434.8688, 1448.8787, 1475.7053, 1479.7522, 1482.7051, 1567.6991, 1618.0178, 1732.0514, 1790.9982, 1790.9982, 1808.9220, 1880.9891, 1880.9891, 1890.9176, 1901.2008, 1993.9156, 2064.0874, 2098.0544, 2211.0493, 2283.1272, 2506.2144, 2506.2144, 2598.2195

29. A0A3Q1M1B1 **Mass:** 29199 **Score:** 40 **Expect:** 14 **Matches:** 9
Phosphatidylinositol transfer protein beta isoform OS=Bos taurus OX=9913 GN=PITPNB PE=4 SV=1

Observed	Mr(expt)	Mr(calc)	ppm	Start	End	Miss	Ions	Peptide
848.4952	847.4879	847.5531	-76.92	174	180	1	---	K.LVTIKFK.W
868.5277	867.5204	867.4603	69.3	150	157	0	---	R.GPLGPNWK.K
996.5623	995.5550	995.5552	-0.21	150	158	1	---	R.GPLGPNWKK.E
1036.5013	1035.4940	1035.4873	6.52	126	134	0	---	R.SQVEPADYK.A
1036.5013	1035.4941	1035.4873	6.56	126	134	0	---	R.SQVEPADYK.A
1090.4995	1089.4922	1089.5832	-83.49	198	205	1	---	K.RIFTNFHR.Q
1162.5488	1161.5415	1161.5414	0.07	35	44	1	---	K.DGEKGQYTHK.I
1179.5667	1178.5594	1178.6237	-54.50	179	187	1	---	K.FKWWGLQSK.V
1200.5436	1199.5363	1199.5182	15.1	71	79	0	---	K.AWNAYPYCR.T

No match to: 842.4847, 845.4012, 850.5135, 853.4918, 857.4026, 857.4026, 864.5186, 870.5200, 882.5434, 882.5434, 951.5951, 987.5186, 1017.6425, 1019.6487, 1033.6698, 1045.5382, 1051.6785, 1051.6785, 1083.5225, 1083.5225, 1107.5156, 1124.5784, 1131.6732, 1136.6610, 1143.6165, 1151.6952, 1165.5533, 1165.7053, 1211.6163, 1211.6163, 1221.5270, 1221.5270, 1263.6616, 1277.6742, 1290.6333, 1300.8134, 1334.8459, 1334.8459, 1361.6049, 1379.6898, 1407.6674, 1434.8688, 1448.8787, 1475.7053, 1479.7522, 1482.7051, 1567.6991, 1618.0178, 1732.0514, 1790.9982, 1790.9982, 1808.9220, 1879.9926, 1880.9891, 1880.9891, 1890.9176, 1901.2008, 1993.9156, 2064.0874, 2098.0544, 2157.0273, 2157.0273, 2179.9844, 2211.0493, 2283.1272, 2506.2144, 2506.2144, 2598.2195

PDF-XChange Click to buy NOW! www.docu-track.com

[illegible]. Q8MII7 **Mass:** 4845 **Score:** 40 **Expect:** 15 **Matches:** 4

Insulin receptor (Fragment) OS=Bos taurus OX=9913 GN=ir-A PE=2 SV=1

Observed	Mr(expt)	Mr(calc)	ppm	Start	End	Miss	Ions	Peptide
987.5186	986.5113	986.5033	8.15	32 -	42	0	---	R.ALGDVGNVTAA.-
996.5623	995.5550	995.4560	99.5	3 -	10	0	---	K.ELEESSFR.K
1124.5784	1123.5711	1123.5509	18.0	3 -	11	1	---	K.ELEESSFRK.T
1143.6165	1142.6092	1142.6044	4.24	31 -	42	1	---	R.RALGDVGNVTAA.-

No match to: 842.4847, 845.4012, 848.4952, 850.5135, 853.4918, 857.4026, 857.4026, 864.5186, 868.5277, 870.5200, 882.5434, 882.5434, 951.5951, 1017.6425, 1019.6487, 1033.6698, 1036.5013, 1036.5013, 1045.5382, 1051.6785, 1051.6785, 1083.5225, 1083.5225, 1090.4995, 1107.5156, 1131.6732, 1136.6610, 1151.6952, 1162.5488, 1165.5533, 1165.7053, 1179.5667, 1200.5436, 1211.6163, 1211.6163, 1221.5270, 1221.5270, 1263.6616, 1277.6742, 1290.6333, 1300.8134, 1334.8459, 1334.8459, 1361.6049, 1379.6898, 1407.6674, 1434.8688, 1448.8787, 1475.7053, 1479.7522, 1482.7051, 1567.6991, 1618.0178, 1732.0514, 1790.9982, 1790.9982, 1808.9220, 1879.9926, 1880.9891, 1880.9891, 1890.9176, 1901.2008, 1993.9156, 2064.0874, 2098.0544, 2157.0273, 2157.0273, 2179.9844, 2211.0493, 2283.1272, 2506.2144, 2506.2144, 2598.2195

Search Parameters

Type of search	: Sequence Query
Enzyme	: Trypsin
Fixed modifications	: Carbamidomethyl (C)
Variable modifications	: Oxidation (M)
Mass values	: Monoisotopic
Protein Mass	: Unrestricted
Peptide Mass Tolerance	: ± 100 ppm
Fragment Mass Tolerance	: ± 0.3 Da
Max Missed Cleavages	: 1
Instrument type	: MALDI-TOF-TOF
Query1 (842.4847,1+)	: <no title>
Query2 (845.4012,1+)	: <no title>
Query3 (848.4952,1+)	: <no title>
Query4 (850.5135,1+)	: <no title>
Query5 (853.4918,1+)	: <no title>
Query6 (857.4026,1+)	: Locus:1..14.0.12
Query7 (857.4026,1+)	: <no title>
Query8 (864.5186,1+)	: <no title>
Query9 (868.5277,1+)	: <no title>
Query10 (870.5200,1+)	: <no title>
Query11 (882.5434,1+)	: <no title>
Query12 (882.5434,1+)	: Locus:1..14.0.9
Query13 (951.5951,1+)	: <no title>
Query14 (987.5186,1+)	: <no title>
Query15 (996.5623,1+)	: <no title>
Query16 (1017.6425,1+)	: <no title>
Query17 (1019.6487,1+)	: <no title>
Query18 (1033.6698,1+)	: <no title>
Query19 (1036.5013,1+)	: <no title>
Query20 (1036.5013,1+)	: Locus:1..14.0.10
Query21 (1045.5382,1+)	: <no title>
Query22 (1051.6785,1+)	: Locus:1..14.0.5
Query23 (1051.6785,1+)	: <no title>
Query24 (1083.5225,1+)	: Locus:1..14.0.3
Query25 (1083.5225,1+)	: <no title>
Query26 (1090.4995,1+)	: <no title>
Query27 (1107.5156,1+)	: <no title>
Query28 (1124.5784,1+)	: <no title>
Query29 (1131.6732,1+)	: <no title>
Query30 (1136.6610,1+)	: <no title>
Query31 (1143.6165,1+)	: <no title>
Query32 (1151.6952,1+)	: <no title>
Query33 (1162.5488,1+)	: <no title>
Query34 (1165.5533,1+)	: <no title>
Query35 (1165.7053,1+)	: <no title>
Query36 (1179.5667,1+)	: <no title>
Query37 (1200.5436,1+)	: <no title>
Query38 (1211.6163,1+)	: <no title>
Query39 (1211.6163,1+)	: Locus:1..14.0.11
Query40 (1221.5270,1+)	: Locus:1..14.0.4
Query41 (1221.5270,1+)	: <no title>
Query42 (1263.6616,1+)	: <no title>
Query43 (1277.6742,1+)	: <no title>
Query44 (1290.6333,1+)	: <no title>
Query45 (1300.8134,1+)	: <no title>
Query46 (1334.8459,1+)	: <no title>
Query47 (1334.8459,1+)	: Locus:1..14.0.8
Query48 (1361.6049,1+)	: <no title>
Query49 (1379.6898,1+)	: <no title>
Query50 (1407.6674,1+)	: <no title>
Query51 (1434.8688,1+)	: <no title>
Query52 (1448.8787,1+)	: <no title>
Query53 (1475.7053,1+)	: <no title>
Query54 (1479.7522,1+)	: <no title>
Query55 (1482.7051,1+)	: <no title>
Query56 (1567.6991,1+)	: <no title>
Query57 (1618.0178,1+)	: <no title>
Query58 (1732.0514,1+)	: <no title>
Query59 (1790.9982,1+)	: Locus:1..14.0.2
Query60 (1790.9982,1+)	: <no title>
Query61 (1808.9220,1+)	: <no title>
Query62 (1879.9926,1+)	: <no title>
Query63 (1880.9891,1+)	: <no title>
Query64 (1880.9891,1+)	: Locus:1..14.0.1
Query65 (1890.9176,1+)	: <no title>
Query66 (1901.2008,1+)	: <no title>
Query67 (1993.9156,1+)	: <no title>
Query68 (2064.0874,1+)	: <no title>
Query69 (2098.0544,1+)	: <no title>
Query70 (2157.0273,1+)	: <no title>
Query71 (2157.0273,1+)	: Locus:1..14.0.6
Query72 (2179.9844,1+)	: <no title>
Query73 (2211.0493,1+)	: <no title>
Query74 (2283.1272,1+)	: <no title>
Query75 (2506.2144,1+)	: Locus:1..14.0.7
Query76 (2506.2144,1+)	: <no title>
Query77 (2598.2195,1+)	: <no title>

Mascot: http://www.matrixscience.com/
