# Supplementary material for: Alpha-2-Macroglobulin as a New Promising Biomarker Improving the Diagnostic Sensitivity of Bovine Paratuberculosis
Source: Front Vet Sci. 2021 Mar 5;8:637716. doi: 10.3389/fvets.2021.637716 (PMC7973028; doi:10.3389/fvets.2021.637716)
Supplement: Supplementary Data 1 — Mascot search results and raw data files for LC-MS/MS analysis for the identification of specific blood proteins in cattle with Johne's disease. [file Data_Sheet_1.zip › mascot/6 Complement component 3d_2.pdf]

PDF-XChange Click to buy NOW! www.docu-track.com

PDF-XChange Click to buy NOW! www.docu-track.com

MATRIX SCIENCE MASCOT Search Results

Protein View: Q693V9

Complement component 3d (Fragment) OS=Bos taurus OX=9913 PE=2 SV=1

Database:	Uniprot_bovine
Score:	251
Expect:	1.1e-020
Nominal mass (M_r):	34593
Calculated pI:	6.68

Sequence similarity is available as an NCBI BLAST search of Q693V9 against nr.

Search parameters

Enzyme:	Trypsin: cuts C-term side of KR unless next residue is P.
Fixed modifications:	Carbamidomethyl (C)
Variable modifications:	Oxidation (M)

Protein sequence coverage: 48%

Matched peptides shown in *bold red*.

```
  1 HLIQTPSGCG EQNMIGMTPT VIAVHYLDST DQWEKFGLEK RQESLELIRK
 51 GYTQQLAFRQ KSSAYAAFQY RPPSTWLTAY VVKVFALAAN LIAIDSKDLC
101 ETVKWLILEK QKPDGIFQED GPVIHQEMIG GFRDTREKDV SLTAFVLIAL
151 HEAKDICEAQ VNSLGRSIAK AGDFLENHYR ELRRPYTVAI AAYALALLGK
201 LEGDRLTKFL NTAKEKNRWE EPNQKLYNVE ATSYALLALL ARKDYDTTPP
251 VVRWLNEQRY YGGGYGSTQA TFMVFQALAQ YQKDVPDHKE LNLDVSIQLP
301 SRN
```

Unformatted sequence string: 303 residues (for pasting into other applications).

Sort peptides by Residue Number Increasing Mass Decreasing Mass

Show predicted peptides also

Query	Start – End	Observed	Mr(expt)	Mr(calc)	ppm	M	Score	Peptide
31	41 – 49	1143.6165	1142.6092	1142.6407	-27.6	1		K.RQESLELIR.K
14	42 – 49	987.5186	986.5113	986.5396	-28.7	0		R.QESLELIR.K
38	50 – 59	1211.6163	1210.6090	1210.6458	-30.4	1		R.KGYTQQLAFR.Q
39	50 – 59	1211.6163	1210.6091	1210.6458	-30.4	1		R.KGYTQQLAFR.Q
24	51 – 59	1083.5225	1082.5152	1082.5509	-33.0	0	14	K.GYTQQLAFR.Q
25	51 – 59	1083.5225	1082.5152	1082.5509	-32.9	0		K.GYTQQLAFR.Q
75	62 – 83	2506.2144	2505.2071	2505.2692	-24.8	0		K.SSAYAAFQYRPPSTWLTAYVVK.V
76	62 – 83	2506.2144	2505.2071	2505.2692	-24.8	0		K.SSAYAAFQYRPPSTWLTAYVVK.V
77	111 – 133	2598.2195	2597.2122	2597.2697	-22.1	0		K.QKPDGIFQEDGPVIHQEMIGGFR.D
48	155 – 166	1361.6049	1360.5976	1360.6405	-31.5	0		K.DICEAQVNSLGR.S
40	171 – 180	1221.5270	1220.5197	1220.5574	-30.9	0	40	K.AGDFLENHYR.E
41	171 – 180	1221.5270	1220.5197	1220.5574	-30.9	0		K.AGDFLENHYR.E
59	184 – 200	1790.9982	1789.9909	1790.0454	-30.4	0		R.RPYTVAIAAYALALLGK.L
60	184 – 200	1790.9982	1789.9909	1790.0454	-30.4	0		R.RPYTVAIAAYALALLGK.L
37	217 – 225	1200.5436	1199.5363	1199.5683	-26.7	1		K.NRWEEPNQK.L
63	226 – 242	1880.9891	1879.9818	1880.0407	-31.3	0		K.LYNVEATSYALLALLAR.K
64	226 – 242	1880.9891	1879.9819	1880.0407	-31.3	0	80	K.LYNVEATSYALLALLAR.K
44	243 – 253	1290.6333	1289.6260	1289.6616	-27.6	1		R.KDYDTTPPVVR.W
33	244 – 253	1162.5488	1161.5415	1161.5666	-21.6	0		K.DYDTTPPVVR.W
2	254 – 259	845.4012	844.3939	844.4191	-29.8	0		R.WLNEQR.Y

PDF-XChange Click to buy NOW! www.docu-track.com

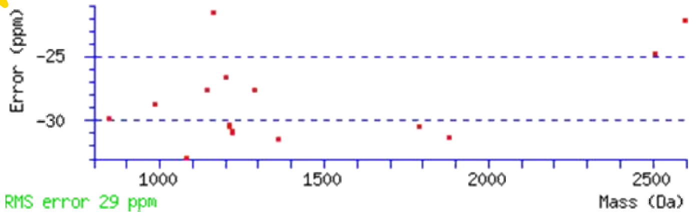

```
ID   Q693V9_BOVIN            Unreviewed;       303 AA.
AC   Q693V9;
DT   13-SEP-2004, integrated into UniProtKB/TrEMBL.
DT   13-SEP-2004, sequence version 1.
DT   26-FEB-2020, entry version 71.
DE   SubName: Full=Complement component 3d {ECO:0000313|EMBL:ACG50173.1};
DE   SubName: Full=Complement component C3d {ECO:0000313|EMBL:AAT76518.1};
DE   Flags: Fragment;
OS   Bos taurus (Bovine).
OC   Eukaryota; Metazoa; Chordata; Craniata; Vertebrata; Euteleostomi; Mammalia;
OC   Eutheria; Laurasiatheria; Artiodactyla; Ruminantia; Pecora; Bovidae;
OC   Bovinae; Bos.
OX   NCBI_TaxID=9913 {ECO:0000313|EMBL:AAT76518.1};
RN   [1] {ECO:0000313|EMBL:AAT76518.1}
RP   NUCLEOTIDE SEQUENCE.
RA   Collada C., Casado R., Gomez L., Allona I., Aragoncillo C.;
RL   Submitted (MAY-2004) to the EMBL/GenBank/DDBJ databases.
RN   [2] {ECO:0000313|EMBL:AAT76518.1}
RP   NUCLEOTIDE SEQUENCE.
RX   PubMed=16904753; DOI=10.1016/j.vetimm.2006.07.005;
RA   Firth M.A., Moore D.P., Pei Y., Shewen P.E., Lo R.Y.C., Yoo D.,
RA   Hodgins D.C.;
RT   "Cloning of a gene fragment encoding bovine complement component C3d with
RT   expression and characterization of derived fusion proteins.";
RL   Vet. Immunol. Immunopathol. 114:61-71(2006).
RN   [3] {ECO:0000313|EMBL:ACG50173.1}
RP   NUCLEOTIDE SEQUENCE.
RC   TISSUE=Liver {ECO:0000313|EMBL:ACG50173.1};
RA   Liu D., Niu Z.X.;
RT   "Cloning and structural analysis of simmental cattle complement component
RT   3d gene.";
RL   Submitted (JUL-2008) to the EMBL/GenBank/DDBJ databases.
CC   -----------------------------------------------------------------------
CC   Copyrighted by the UniProt Consortium, see https://www.uniprot.org/terms
CC   Distributed under the Creative Commons Attribution (CC BY 4.0) License
CC   -----------------------------------------------------------------------
DR   EMBL; AY630404; AAT76518.1; -; mRNA.
DR   EMBL; EU868825; ACG50173.1; -; mRNA.
DR   SMR; Q693V9; -.
DR   eggNOG; KOG1366; Eukaryota.
DR   eggNOG; ENOG410XRED; LUCA.
DR   HOGENOM; CLU_001634_4_0_1; -.
DR   GO; GO:0005615; C:extracellular space; IEA:InterPro.
DR   GO; GO:0006956; P:complement activation; IEA:InterPro.
DR   InterPro; IPR011626; Alpha-macroglobulin_TED.
DR   InterPro; IPR035711; Complement_C3-like.
DR   InterPro; IPR019742; MacrogloblnA2_CS.
DR   InterPro; IPR008930; Terpenoid_cyclase/PrenylTrfase.
DR   PANTHER; PTHR11412:SF81; PTHR11412:SF81; 1.
DR   Pfam; PF07678; TED_complement; 1.
DR   SUPFAM; SSF48239; SSF48239; 1.
DR   PROSITE; PS00477; ALPHA_2_MACROGLOBULIN; 1.
PE   2: Evidence at transcript level;
FT   DOMAIN          2..281
FT                   /note="TED_complement"
FT                   /evidence="ECO:0000259|Pfam:PF07678"
FT   NON_TER         1
FT                   /evidence="ECO:0000313|EMBL:AAT76518.1"
FT   NON_TER         303
FT                   /evidence="ECO:0000313|EMBL:AAT76518.1"
SQ   SEQUENCE   303 AA;  34443 MW;  2F3A15020CEA3797 CRC64;
     HLIQTPSGCG EQNMIGMTPT VIAVHYLDST DQWEKFGLEK RQESLELIRK GYTQQLAFRQ
     KSSAYAAFQY RPPSTWLTAY VVKVFALAAN LIAIDSKDLC ETVKWLILEK QKPDGIFQED
     GPVIHQEMIG GFRDTREKDV SLTAFVLIAL HEAKDICEAQ VNSLGRSIAK AGDFLENHYR
     ELRRPYTVAI AAYALALLGK LEGDRLTKFL NTAKEKNRWE EPNQKLYNVE ATSYALLALL
     ARKDYDTTPP VVRWLNEQRY YGGGYGSTQA TFMVFQALAQ YQKDVPDHKE LNLDVSIQLP
     SRN
//
```

Mascot: http://www.matrixscience.com/
