# Supplementary material for: Alpha-2-Macroglobulin as a New Promising Biomarker Improving the Diagnostic Sensitivity of Bovine Paratuberculosis
Source: Front Vet Sci. 2021 Mar 5;8:637716. doi: 10.3389/fvets.2021.637716 (PMC7973028; doi:10.3389/fvets.2021.637716)
Supplement: Supplementary Data 1 — Mascot search results and raw data files for LC-MS/MS analysis for the identification of specific blood proteins in cattle with Johne's disease. [file Data_Sheet_1.zip › mascot/7 alpha-1B-glycoprotein precursor_1.pdf]

PDF-XChange Click to buy NOW! www.docu-track.com

MATRIX SCIENCE Mascot Search Results

```
User         :
Email        :
Search title : BAEDW\2020_3_5\2020_3_5\MSMS 14\N19
Database     : Uniprot_bovine bovine_20200316 (134392 sequences; 76244912 residues)
Timestamp    : 31 Mar 2020 at 01:08:58 GMT
Warning      : A Peptide summary report will usually give a much clearer picture of MS/MS search results.
Top Score    : 98 for Q2KJF1, Alpha-1B-glycoprotein OS=Bos taurus OX=9913 GN=A1BG PE=1 SV=1
```

Mascot Score Histogram

Protein score is -10*Log(P), where P is the probability that the observed match is a random event.
Protein scores greater than 64 are significant (p<0.05).
Protein scores are derived from ions scores as a non-probabilistic basis for ranking protein hits.

Error: try setting browser cache to automatic.

Protein Summary Report

Format As | Protein Summary (deprecated) | Help

Significance threshold p< 0.05 Max. number of hits 30

Preferred taxonomy All entries

Re-Search All | Search Unmatched

Index

	Accession	Mass	Score	Description
1.	Q2KJF1	54091	98	Alpha-1B-glycoprotein OS=Bos taurus OX=9913 GN=A1BG PE=1 SV=1
2.	A0A4W2GYB4	54132	97	Alpha-1-B glycoprotein OS=Bos indicus x Bos taurus OX=30522 GN=A1BG PE=4 SV=1
3.	A0A4W2E798	54147	97	Alpha-1-B glycoprotein OS=Bos indicus x Bos taurus OX=30522 GN=A1BG PE=4 SV=1
4.	A0A4W2IFR5	54987	97	Alpha-1-B glycoprotein OS=Bos indicus x Bos taurus OX=30522 GN=A1BG PE=4 SV=1
5.	A0A4W2E8B7	55003	97	Alpha-1-B glycoprotein OS=Bos indicus x Bos taurus OX=30522 GN=A1BG PE=4 SV=1
6.	A0A4W2H1C4	60879	94	Alpha-1-B glycoprotein OS=Bos indicus x Bos taurus OX=30522 GN=A1BG PE=4 SV=1
7.	A0A3Q1MJT2	62948	93	Alpha-1B-glycoprotein OS=Bos taurus OX=9913 GN=A1BG PE=1 SV=1
8.	A0A4W2EKQ3	63006	93	Alpha-1-B glycoprotein OS=Bos indicus x Bos taurus OX=30522 GN=A1BG PE=4 SV=1
9.	A0A4W2IGA3	80257	50	Kinesin-like protein OS=Bos indicus x Bos taurus OX=30522 GN=KIF3B PE=3 SV=1
10.	A0A3Q1MG81	80257	50	Kinesin-like protein OS=Bos taurus OX=9913 GN=KIF3B PE=3 SV=1
11.	A0A4W2H2X1	84971	49	Kinesin-like protein OS=Bos indicus x Bos taurus OX=30522 GN=KIF3B PE=3 SV=1
12.	F1N020	84971	49	Kinesin-like protein OS=Bos taurus OX=9913 GN=KIF3B PE=3 SV=2
13.	A0A4W2F5Q7	26142	41	Peroxiredoxin like 2A OS=Bos indicus x Bos taurus OX=30522 GN=PRXL2A PE=4 SV=1
14.	A0A3Q1LNN7	68022	41	Serum albumin OS=Bos taurus OX=9913 GN=ALB PE=4 SV=1
15.	A0A452DI98	15508	41	U6 snRNA-associated Sm-like protein LSm4 OS=Bos taurus OX=9913 GN=LSM4 PE=3 SV=1
16.	A0A4W2FGN9	123016	40	Coiled-coil domain containing 150 OS=Bos indicus x Bos taurus OX=30522 GN=CCDC150 PE=4 SV=1
17.	A0A4W2I014	105104	40	Protein phosphatase 1 regulatory subunit 21 OS=Bos indicus x Bos taurus OX=30522 GN=PPP1R21 PE=4 SV=1
18.	A0A4W2CGR1	105032	40	Protein phosphatase 1 regulatory subunit 21 OS=Bos indicus x Bos taurus OX=30522 GN=PPP1R21 PE=4 SV=1
19.	A0A4W2DDL5	70207	40	Albumin OS=Bos indicus x Bos taurus OX=30522 GN=ALB PE=4 SV=1
20.	A0A140T897	71274	40	Serum albumin OS=Bos taurus OX=9913 GN=ALB PE=4 SV=1
21.	A0A4W2EVR9	71453	40	Albumin OS=Bos indicus x Bos taurus OX=30522 GN=ALB PE=4 SV=1
22.	A0A4W2DGX3	13967	40	U6 snRNA-associated Sm-like protein LSm4 OS=Bos indicus x Bos taurus OX=30522 GN=LSM4 PE=3 SV=1
23.	A0A4W2HT62	15511	39	U6 snRNA-associated Sm-like protein LSm4 OS=Bos indicus x Bos taurus OX=30522 GN=LSM4 PE=3 SV=1
24.	Q3ZBK6	15511	39	U6 snRNA-associated Sm-like protein LSm4 OS=Bos taurus OX=9913 GN=LSM4 PE=2 SV=1
25.	A0A4W2EW60	76020	39	Albumin OS=Bos indicus x Bos taurus OX=30522 GN=ALB PE=4 SV=1
26.	A0A4W2D8T3	76089	39	Albumin OS=Bos indicus x Bos taurus OX=30522 GN=ALB PE=4 SV=1
27.	A0A4W2HAU5	24454	38	Peroxiredoxin like 2A OS=Bos indicus x Bos taurus OX=30522 GN=PRXL2A PE=4 SV=1
28.	Q3ZBK2	24454	38	Peroxiredoxin like 2A OS=Bos taurus OX=9913 GN=PRXL2A PE=2 SV=1
29.	A0A4W2H5Z2	33370	37	Ketohexokinase OS=Bos indicus x Bos taurus OX=30522 GN=KHK PE=4 SV=1
30.	P02769	71244	36	Serum albumin OS=Bos taurus OX=9913 GN=ALB PE=1 SV=4

Results List

1. Q2KJF1 **Mass:** 54091 **Score:** 98 **Expect:** 2.4e-005 **Matches:** 12
Alpha-1B-glycoprotein OS=Bos taurus OX=9913 GN=A1BG PE=1 SV=1

Observed	Mr(expt)	Mr(calc)	ppm	Start		End	Miss	Ions	Peptide
805.4537	804.4464	804.4858	-48.92	148	-	154	0	---	R.GVTFLLR.L
919.4136	918.4063	918.4559	-53.98	288	-	295	0	---	R.SELAAWSR.D
1142.5725	1141.5652	1141.6244	-51.83	415	-	425	0	---	R.ALWTGALTPGR.D
1142.5725	1141.5652	1141.6244	-51.82	415	-	425	0	---	R.ALWTGALTPGR.D
1175.5778	1174.5705	1174.6346	-54.59	81	-	91	0	---	R.FPLGPVTSTTR.G
1457.7133	1456.7060	1456.7773	-48.93	487	-	499	0	---	R.SLLSELSDPVELR.V
1498.6241	1497.6168	1497.6882	-47.65	269	-	283	0	---	R.LSALAAGDGSGYTCR.Y
1513.7238	1512.7165	1512.7936	-51.00	357	-	370	0	27	R.VLSPAGPEAQFELR.G
1513.7238	1512.7165	1512.7936	-50.97	357	-	370	0	---	R.VLSPAGPEAQFELR.G
1647.7585	1646.7512	1646.8338	-50.13	431	-	444	0	---	R.CEAEVPDVSFLLLR.A
1647.7585	1646.7513	1646.8338	-50.11	431	-	444	0	27	R.CEAEVPDVSFLLLR.A
2530.2642	2529.2569	2529.3374	-31.80	224	-	247	0	---	K.VLRPGSSASLTCVAPLSGVDFQLR.R

No match to: 842.4627, 848.4779, 850.0055, 850.4871, 853.4691, 864.5048, 865.9789, 868.4995, 870.5005, 876.9857, 882.5121, 882.5121, 892.9567, 927.4397, 951.5660, 982.5261, 996.5410, 1017.6141, 1017.6141, 1019.6234, 1033.6417, 1045.5149, 1051.6498, 1051.6498, 1065.5970, 1131.6476, 1133.6560, 1136.6389, 1147.6736, 1151.6641, 1165.6772, 1165.6773, 1194.5631,

PDF-XChange Click to buy NOW! www.docu-track.com

PDF-XChange Click to buy NOW! www.docu-track.com

1300.7844, 1302.7943, 1316.8119, 1334.8163, 1334.8163, 1351.5889, 1414.8141, 1434.8339, 1448.8483, 1448.8483, 1475.6820,
1479.7203, 1479.7203, 1557.7402, 1567.6654, 1567.6654, 1583.9542, 1615.7332, 1617.9894, 1617.9894, 1630.7391, 1633.7523,
1697.8313, 1724.7576, 1732.0159, 1901.1652, 2015.1964, 2044.9374, 2184.3481, 2211.0227, 2283.0957, 2305.0806

2. A0A4W2GYB4 **Mass:** 54132 **Score:** 97 **Expect:** 2.4e-005 **Matches:** 12

Alpha-1-B glycoprotein OS=Bos indicus x Bos taurus OX=30522 GN=A1BG PE=4 SV=1

Observed	Mr(expt)	Mr(calc)	ppm	Start	End	Miss	Ions	Peptide
805.4537	804.4464	804.4858	-48.92	142 -	148	0	---	R.GVTFLLR.L
919.4136	918.4063	918.4559	-53.98	282 -	289	0	---	R.SELAAWSR.D
1142.5725	1141.5652	1141.6244	-51.83	417 -	427	0	---	R.ALWTGALTPGR.D
1142.5725	1141.5652	1141.6244	-51.82	417 -	427	0	---	R.ALWTGALTPGR.D
1175.5778	1174.5705	1174.6346	-54.59	75 -	85	0	---	R.FPLGPVTSTTR.G
1457.7133	1456.7060	1456.7773	-48.93	489 -	501	0	---	R.SLLSELSDPVELR.V
1498.6241	1497.6168	1497.6882	-47.65	263 -	277	0	---	R.LSALAAGDGSGYTCR.Y
1513.7238	1512.7165	1512.7936	-51.00	351 -	364	0	27	R.VLSPAGPEAQFELR.G
1513.7238	1512.7165	1512.7936	-50.97	351 -	364	0	---	R.VLSPAGPEAQFELR.G
1647.7585	1646.7512	1646.8338	-50.13	433 -	446	0	---	R.CEAEVPDVSFLLLR.A
1647.7585	1646.7513	1646.8338	-50.11	433 -	446	0	27	R.CEAEVPDVSFLLLR.A
2530.2642	2529.2569	2529.3374	-31.80	218 -	241	0	---	K.VLRPGSSASLTCVAPLSGVDFQLR.R

No match to: 842.4627, 848.4779, 850.0055, 850.4871, 853.4691, 864.5048, 865.9789, 868.4995, 870.5005, 876.9857, 882.5121,
882.5121, 892.9567, 927.4397, 951.5660, 982.5261, 996.5410, 1017.6141, 1017.6141, 1019.6234, 1033.6417, 1045.5149,
1051.6498, 1051.6498, 1065.5970, 1131.6476, 1133.6560, 1136.6389, 1147.6736, 1151.6641, 1165.6772, 1165.6773, 1194.5631,
1300.7844, 1302.7943, 1316.8119, 1334.8163, 1334.8163, 1351.5889, 1414.8141, 1434.8339, 1448.8483, 1448.8483, 1475.6820,
1479.7203, 1479.7203, 1557.7402, 1567.6654, 1567.6654, 1583.9542, 1615.7332, 1617.9894, 1617.9894, 1630.7391, 1633.7523,
1697.8313, 1724.7576, 1732.0159, 1901.1652, 2015.1964, 2044.9374, 2184.3481, 2211.0227, 2283.0957, 2305.0806

3. A0A4W2E798 **Mass:** 54147 **Score:** 97 **Expect:** 2.4e-005 **Matches:** 12

Alpha-1-B glycoprotein OS=Bos indicus x Bos taurus OX=30522 GN=A1BG PE=4 SV=1

Observed	Mr(expt)	Mr(calc)	ppm	Start	End	Miss	Ions	Peptide
805.4537	804.4464	804.4858	-48.92	142 -	148	0	---	R.GVTFLLR.L
919.4136	918.4063	918.4559	-53.98	282 -	289	0	---	R.SELAAWSR.D
1142.5725	1141.5652	1141.6244	-51.83	417 -	427	0	---	R.ALWTGALTPGR.D
1142.5725	1141.5652	1141.6244	-51.82	417 -	427	0	---	R.ALWTGALTPGR.D
1175.5778	1174.5705	1174.6346	-54.59	75 -	85	0	---	R.FPLGPVTSTTR.G
1457.7133	1456.7060	1456.7773	-48.93	489 -	501	0	---	R.SLLSELSDPVELR.V
1498.6241	1497.6168	1497.6882	-47.65	263 -	277	0	---	R.LSALAAGDGSGYTCR.Y
1513.7238	1512.7165	1512.7936	-51.00	351 -	364	0	27	R.VLSPAGPEAQFELR.G
1513.7238	1512.7165	1512.7936	-50.97	351 -	364	0	---	R.VLSPAGPEAQFELR.G
1647.7585	1646.7512	1646.8338	-50.13	433 -	446	0	---	R.CEAEVPDVSFLLLR.A
1647.7585	1646.7513	1646.8338	-50.11	433 -	446	0	27	R.CEAEVPDVSFLLLR.A
2530.2642	2529.2569	2529.3374	-31.80	218 -	241	0	---	K.VLRPGSSASLTCVAPLSGVDFQLR.R

No match to: 842.4627, 848.4779, 850.0055, 850.4871, 853.4691, 864.5048, 865.9789, 868.4995, 870.5005, 876.9857, 882.5121,
882.5121, 892.9567, 927.4397, 951.5660, 982.5261, 996.5410, 1017.6141, 1017.6141, 1019.6234, 1033.6417, 1045.5149,
1051.6498, 1051.6498, 1065.5970, 1131.6476, 1133.6560, 1136.6389, 1147.6736, 1151.6641, 1165.6772, 1165.6773, 1194.5631,
1300.7844, 1302.7943, 1316.8119, 1334.8163, 1334.8163, 1351.5889, 1414.8141, 1434.8339, 1448.8483, 1448.8483, 1475.6820,
1479.7203, 1479.7203, 1557.7402, 1567.6654, 1567.6654, 1583.9542, 1615.7332, 1617.9894, 1617.9894, 1630.7391, 1633.7523,
1697.8313, 1724.7576, 1732.0159, 1901.1652, 2015.1964, 2044.9374, 2184.3481, 2211.0227, 2283.0957, 2305.0806

4. A0A4W2IFR5 **Mass:** 54987 **Score:** 97 **Expect:** 2.6e-005 **Matches:** 12

Alpha-1-B glycoprotein OS=Bos indicus x Bos taurus OX=30522 GN=A1BG PE=4 SV=1

Observed	Mr(expt)	Mr(calc)	ppm	Start	End	Miss	Ions	Peptide
805.4537	804.4464	804.4858	-48.92	148 -	154	0	---	R.GVTFLLR.L
919.4136	918.4063	918.4559	-53.98	288 -	295	0	---	R.SELAAWSR.D
1142.5725	1141.5652	1141.6244	-51.83	423 -	433	0	---	R.ALWTGALTPGR.D
1142.5725	1141.5652	1141.6244	-51.82	423 -	433	0	---	R.ALWTGALTPGR.D
1175.5778	1174.5705	1174.6346	-54.59	81 -	91	0	---	R.FPLGPVTSTTR.G
1457.7133	1456.7060	1456.7773	-48.93	495 -	507	0	---	R.SLLSELSDPVELR.V
1498.6241	1497.6168	1497.6882	-47.65	269 -	283	0	---	R.LSALAAGDGSGYTCR.Y
1513.7238	1512.7165	1512.7936	-51.00	357 -	370	0	27	R.VLSPAGPEAQFELR.G
1513.7238	1512.7165	1512.7936	-50.97	357 -	370	0	---	R.VLSPAGPEAQFELR.G
1647.7585	1646.7512	1646.8338	-50.13	439 -	452	0	---	R.CEAEVPDVSFLLLR.A
1647.7585	1646.7513	1646.8338	-50.11	439 -	452	0	27	R.CEAEVPDVSFLLLR.A
2530.2642	2529.2569	2529.3374	-31.80	224 -	247	0	---	K.VLRPGSSASLTCVAPLSGVDFQLR.R

No match to: 842.4627, 848.4779, 850.0055, 850.4871, 853.4691, 864.5048, 865.9789, 868.4995, 870.5005, 876.9857, 882.5121,
882.5121, 892.9567, 927.4397, 951.5660, 982.5261, 996.5410, 1017.6141, 1017.6141, 1019.6234, 1033.6417, 1045.5149,
1051.6498, 1051.6498, 1065.5970, 1131.6476, 1133.6560, 1136.6389, 1147.6736, 1151.6641, 1165.6772, 1165.6773, 1194.5631,
1300.7844, 1302.7943, 1316.8119, 1334.8163, 1334.8163, 1351.5889, 1414.8141, 1434.8339, 1448.8483, 1448.8483, 1475.6820,
1479.7203, 1479.7203, 1557.7402, 1567.6654, 1567.6654, 1583.9542, 1615.7332, 1617.9894, 1617.9894, 1630.7391, 1633.7523,
1697.8313, 1724.7576, 1732.0159, 1901.1652, 2015.1964, 2044.9374, 2184.3481, 2211.0227, 2283.0957, 2305.0806

5. A0A4W2E8B7 **Mass:** 55003 **Score:** 97 **Expect:** 2.6e-005 **Matches:** 12

Alpha-1-B glycoprotein OS=Bos indicus x Bos taurus OX=30522 GN=A1BG PE=4 SV=1

Observed	Mr(expt)	Mr(calc)	ppm	Start	End	Miss	Ions	Peptide
805.4537	804.4464	804.4858	-48.92	148 -	154	0	---	R.GVTFLLR.L
919.4136	918.4063	918.4559	-53.98	288 -	295	0	---	R.SELAAWSR.D
1142.5725	1141.5652	1141.6244	-51.83	423 -	433	0	---	R.ALWTGALTPGR.D
1142.5725	1141.5652	1141.6244	-51.82	423 -	433	0	---	R.ALWTGALTPGR.D
1175.5778	1174.5705	1174.6346	-54.59	81 -	91	0	---	R.FPLGPVTSTTR.G
1457.7133	1456.7060	1456.7773	-48.93	495 -	507	0	---	R.SLLSELSDPVELR.V
1498.6241	1497.6168	1497.6882	-47.65	269 -	283	0	---	R.LSALAAGDGSGYTCR.Y
1513.7238	1512.7165	1512.7936	-51.00	357 -	370	0	27	R.VLSPAGPEAQFELR.G
1513.7238	1512.7165	1512.7936	-50.97	357 -	370	0	---	R.VLSPAGPEAQFELR.G
1647.7585	1646.7512	1646.8338	-50.13	439 -	452	0	---	R.CEAEVPDVSFLLLR.A
1647.7585	1646.7513	1646.8338	-50.11	439 -	452	0	27	R.CEAEVPDVSFLLLR.A
2530.2642	2529.2569	2529.3374	-31.80	224 -	247	0	---	K.VLRPGSSASLTCVAPLSGVDFQLR.R

No match to: 842.4627, 848.4779, 850.0055, 850.4871, 853.4691, 864.5048, 865.9789, 868.4995, 870.5005, 876.9857, 882.5121,
882.5121, 892.9567, 927.4397, 951.5660, 982.5261, 996.5410, 1017.6141, 1017.6141, 1019.6234, 1033.6417, 1045.5149,
1051.6498, 1051.6498, 1065.5970, 1131.6476, 1133.6560, 1136.6389, 1147.6736, 1151.6641, 1165.6772, 1165.6773, 1194.5631,
1300.7844, 1302.7943, 1316.8119, 1334.8163, 1334.8163, 1351.5889, 1414.8141, 1434.8339, 1448.8483, 1448.8483, 1475.6820,
1479.7203, 1479.7203, 1557.7402, 1567.6654, 1567.6654, 1583.9542, 1615.7332, 1617.9894, 1617.9894, 1630.7391, 1633.7523,
1697.8313, 1724.7576, 1732.0159, 1901.1652, 2015.1964, 2044.9374, 2184.3481, 2211.0227, 2283.0957, 2305.0806

6. A0A4W2H1C4 **Mass:** 60879 **Score:** 94 **Expect:** 5.6e-005 **Matches:** 12

Alpha-1-B glycoprotein OS=Bos indicus x Bos taurus OX=30522 GN=A1BG PE=4 SV=1

PDF-XChange Click to buy NOW! www.docu-track.com

PDF-XChange Click to buy NOW! www.docu-track.com

Observed	Mr(expt)	Mr(calc)	ppm	Start	End	Miss	Ions	Peptide
805.4537	804.4464	804.4858	-48.92	148 -	154	0	---	R.GVTFLLR.L
919.4136	918.4063	918.4559	-53.98	288 -	295	0	---	R.SELAAWSR.D
1142.5725	1141.5652	1141.6244	-51.83	415 -	425	0	---	R.ALWTGALTPGR.D
1142.5725	1141.5652	1141.6244	-51.82	415 -	425	0	---	R.ALWTGALTPGR.D
1175.5778	1174.5705	1174.6346	-54.59	81 -	91	0	---	R.FPLGPVTSTTR.G
1457.7133	1456.7060	1456.7773	-48.93	487 -	499	0	---	R.SLLSELSDPVELR.V
1498.6241	1497.6168	1497.6882	-47.65	269 -	283	0	---	R.LSALAAGDGSGYTCR.Y
1513.7238	1512.7165	1512.7936	-51.00	357 -	370	0	27	R.VLSPAGPEAQFELR.G
1513.7238	1512.7165	1512.7936	-50.97	357 -	370	0	---	R.VLSPAGPEAQFELR.G
1647.7585	1646.7512	1646.8338	-50.13	431 -	444	0	---	R.CEAEVPDVSFLLLR.A
1647.7585	1646.7513	1646.8338	-50.11	431 -	444	0	27	R.CEAEVPDVSFLLLR.A
2530.2642	2529.2569	2529.3374	-31.80	224 -	247	0	---	K.VLRPGSSASLTCVAPLSGVDFQLR.R

No match to: 842.4627, 848.4779, 850.0055, 850.4871, 853.4691, 864.5048, 865.9789, 868.4995, 870.5005, 876.9857, 882.5121, 882.5121, 892.9567, 927.4397, 951.5660, 982.5261, 996.5410, 1017.6141, 1017.6141, 1019.6234, 1033.6417, 1045.5149, 1051.6498, 1051.6498, 1065.5970, 1131.6476, 1133.6560, 1136.6389, 1147.6736, 1151.6641, 1165.6772, 1165.6773, 1194.5631, 1300.7844, 1302.7943, 1316.8119, 1334.8163, 1334.8163, 1351.5889, 1414.8141, 1434.8339, 1448.8483, 1448.8483, 1475.6820, 1479.7203, 1479.7203, 1557.7402, 1567.6654, 1567.6654, 1583.9542, 1615.7332, 1617.9894, 1617.9894, 1630.7391, 1633.7523, 1697.8313, 1724.7576, 1732.0159, 1901.1652, 2015.1964, 2044.9374, 2184.3481, 2211.0227, 2283.0957, 2305.0806

7. A0A3Q1MJT2 **Mass:** 62948 **Score:** 93 **Expect:** 7.1e-005 **Matches:** 12

Alpha-1B-glycoprotein OS=Bos taurus OX=9913 GN=A1BG PE=1 SV=1

Observed	Mr(expt)	Mr(calc)	ppm	Start	End	Miss	Ions	Peptide
805.4537	804.4464	804.4858	-48.92	148 -	154	0	---	R.GVTFLLR.L
919.4136	918.4063	918.4559	-53.98	288 -	295	0	---	R.SELAAWSR.D
1142.5725	1141.5652	1141.6244	-51.83	415 -	425	0	---	R.ALWTGALTPGR.D
1142.5725	1141.5652	1141.6244	-51.82	415 -	425	0	---	R.ALWTGALTPGR.D
1175.5778	1174.5705	1174.6346	-54.59	81 -	91	0	---	R.FPLGPVTSTTR.G
1457.7133	1456.7060	1456.7773	-48.93	487 -	499	0	---	R.SLLSELSDPVELR.V
1498.6241	1497.6168	1497.6882	-47.65	269 -	283	0	---	R.LSALAAGDGSGYTCR.Y
1513.7238	1512.7165	1512.7936	-51.00	357 -	370	0	27	R.VLSPAGPEAQFELR.G
1513.7238	1512.7165	1512.7936	-50.97	357 -	370	0	---	R.VLSPAGPEAQFELR.G
1647.7585	1646.7512	1646.8338	-50.13	431 -	444	0	---	R.CEAEVPDVSFLLLR.A
1647.7585	1646.7513	1646.8338	-50.11	431 -	444	0	27	R.CEAEVPDVSFLLLR.A
2530.2642	2529.2569	2529.3374	-31.80	224 -	247	0	---	K.VLRPGSSASLTCVAPLSGVDFQLR.R

No match to: 842.4627, 848.4779, 850.0055, 850.4871, 853.4691, 864.5048, 865.9789, 868.4995, 870.5005, 876.9857, 882.5121, 882.5121, 892.9567, 927.4397, 951.5660, 982.5261, 996.5410, 1017.6141, 1017.6141, 1019.6234, 1033.6417, 1045.5149, 1051.6498, 1051.6498, 1065.5970, 1131.6476, 1133.6560, 1136.6389, 1147.6736, 1151.6641, 1165.6772, 1165.6773, 1194.5631, 1300.7844, 1302.7943, 1316.8119, 1334.8163, 1334.8163, 1351.5889, 1414.8141, 1434.8339, 1448.8483, 1448.8483, 1475.6820, 1479.7203, 1479.7203, 1557.7402, 1567.6654, 1567.6654, 1583.9542, 1615.7332, 1617.9894, 1617.9894, 1630.7391, 1633.7523, 1697.8313, 1724.7576, 1732.0159, 1901.1652, 2015.1964, 2044.9374, 2184.3481, 2211.0227, 2283.0957, 2305.0806

8. A0A4W2EKQ3 **Mass:** 63006 **Score:** 93 **Expect:** 7.1e-005 **Matches:** 12

Alpha-1-B glycoprotein OS=Bos indicus x Bos taurus OX=30522 GN=A1BG PE=4 SV=1

Observed	Mr(expt)	Mr(calc)	ppm	Start	End	Miss	Ions	Peptide
805.4537	804.4464	804.4858	-48.92	148 -	154	0	---	R.GVTFLLR.L
919.4136	918.4063	918.4559	-53.98	288 -	295	0	---	R.SELAAWSR.D
1142.5725	1141.5652	1141.6244	-51.83	415 -	425	0	---	R.ALWTGALTPGR.D
1142.5725	1141.5652	1141.6244	-51.82	415 -	425	0	---	R.ALWTGALTPGR.D
1175.5778	1174.5705	1174.6346	-54.59	81 -	91	0	---	R.FPLGPVTSTTR.G
1457.7133	1456.7060	1456.7773	-48.93	487 -	499	0	---	R.SLLSELSDPVELR.V
1498.6241	1497.6168	1497.6882	-47.65	269 -	283	0	---	R.LSALAAGDGSGYTCR.Y
1513.7238	1512.7165	1512.7936	-51.00	357 -	370	0	27	R.VLSPAGPEAQFELR.G
1513.7238	1512.7165	1512.7936	-50.97	357 -	370	0	---	R.VLSPAGPEAQFELR.G
1647.7585	1646.7512	1646.8338	-50.13	431 -	444	0	---	R.CEAEVPDVSFLLLR.A
1647.7585	1646.7513	1646.8338	-50.11	431 -	444	0	27	R.CEAEVPDVSFLLLR.A
2530.2642	2529.2569	2529.3374	-31.80	224 -	247	0	---	K.VLRPGSSASLTCVAPLSGVDFQLR.R

No match to: 842.4627, 848.4779, 850.0055, 850.4871, 853.4691, 864.5048, 865.9789, 868.4995, 870.5005, 876.9857, 882.5121, 882.5121, 892.9567, 927.4397, 951.5660, 982.5261, 996.5410, 1017.6141, 1017.6141, 1019.6234, 1033.6417, 1045.5149, 1051.6498, 1051.6498, 1065.5970, 1131.6476, 1133.6560, 1136.6389, 1147.6736, 1151.6641, 1165.6772, 1165.6773, 1194.5631, 1300.7844, 1302.7943, 1316.8119, 1334.8163, 1334.8163, 1351.5889, 1414.8141, 1434.8339, 1448.8483, 1448.8483, 1475.6820, 1479.7203, 1479.7203, 1557.7402, 1567.6654, 1567.6654, 1583.9542, 1615.7332, 1617.9894, 1617.9894, 1630.7391, 1633.7523, 1697.8313, 1724.7576, 1732.0159, 1901.1652, 2015.1964, 2044.9374, 2184.3481, 2211.0227, 2283.0957, 2305.0806

9. A0A4W2IGA3 **Mass:** 80257 **Score:** 50 **Expect:** 1.4 **Matches:** 8

Kinesin-like protein OS=Bos indicus x Bos taurus OX=30522 GN=KIF3B PE=3 SV=1

Observed	Mr(expt)	Mr(calc)	ppm	Start	End	Miss	Ions	Peptide
868.4995	867.4922	867.4273	74.9	48 -	55	0	---	K.GVAHEMPK.T
1033.6417	1032.6344	1032.6331	1.24	540 -	548	1	---	K.LFSKLQAVK.A
1045.5149	1044.5076	1044.5312	-22.57	252 -	261	1	---	R.QAKTGAQGER.L
1498.6241	1497.6168	1497.6770	-40.15	103 -	115	1	---	K.TYTMEGVRGDPEK.R + Oxidation (M)
1513.7238	1512.7165	1512.7460	-19.50	142 -	153	0	9	R.ASYLEIYQEEIR.D
1513.7238	1512.7165	1512.7460	-19.47	142 -	153	0	---	R.ASYLEIYQEEIR.D
1647.7585	1646.7512	1646.8007	-30.06	615 -	628	0	---	R.AENIMLLELDMPSR.T + Oxidation (M)
1647.7585	1646.7513	1646.8007	-30.04	615 -	628	0	27	R.AENIMLLELDMPSR.T + Oxidation (M)

No match to: 805.4537, 842.4627, 848.4779, 850.0055, 850.4871, 853.4691, 864.5048, 865.9789, 870.5005, 876.9857, 882.5121, 882.5121, 892.9567, 919.4136, 927.4397, 951.5660, 982.5261, 996.5410, 1017.6141, 1017.6141, 1019.6234, 1051.6498, 1051.6498, 1065.5970, 1131.6476, 1133.6560, 1136.6389, 1142.5725, 1142.5725, 1147.6736, 1151.6641, 1165.6772, 1165.6773, 1175.5778, 1194.5631, 1300.7844, 1302.7943, 1316.8119, 1334.8163, 1334.8163, 1351.5889, 1414.8141, 1434.8339, 1448.8483, 1448.8483, 1457.7133, 1475.6820, 1479.7203, 1479.7203, 1557.7402, 1567.6654, 1567.6654, 1583.9542, 1615.7332, 1617.9894, 1617.9894, 1630.7391, 1633.7523, 1697.8313, 1724.7576, 1732.0159, 1901.1652, 2015.1964, 2044.9374, 2184.3481, 2211.0227, 2283.0957, 2305.0806, 2530.2642

10. A0A3Q1MG81 **Mass:** 80257 **Score:** 50 **Expect:** 1.4 **Matches:** 8

Kinesin-like protein OS=Bos taurus OX=9913 GN=KIF3B PE=3 SV=1

Observed	Mr(expt)	Mr(calc)	ppm	Start	End	Miss	Ions	Peptide
868.4995	867.4922	867.4273	74.9	48 -	55	0	---	K.GVAHEMPK.T
1033.6417	1032.6344	1032.6331	1.24	540 -	548	1	---	K.LFSKLQAVK.A
1045.5149	1044.5076	1044.5312	-22.57	252 -	261	1	---	R.QAKTGAQGER.L
1498.6241	1497.6168	1497.6770	-40.15	103 -	115	1	---	K.TYTMEGVRGDPEK.R + Oxidation (M)
1513.7238	1512.7165	1512.7460	-19.50	142 -	153	0	9	R.ASYLEIYQEEIR.D
1513.7238	1512.7165	1512.7460	-19.47	142 -	153	0	---	R.ASYLEIYQEEIR.D
1647.7585	1646.7512	1646.8007	-30.06	615 -	628	0	---	R.AENIMLLELDMPSR.T + Oxidation (M)
1647.7585	1646.7513	1646.8007	-30.04	615 -	628	0	27	R.AENIMLLELDMPSR.T + Oxidation (M)

PDF-XChange Click to buy NOW! www.docu-track.com

PDF-XChange Click to buy NOW! www.docu-track.com

No match to: 805.4537, 842.4627, 848.4779, 850.0055, 850.4871, 853.4691, 864.5048, 865.9789, 870.5005, 876.9857, 882.5121, 882.5121, 892.9567, 919.4136, 927.4397, 951.5660, 982.5261, 996.5410, 1017.6141, 1017.6141, 1019.6234, 1051.6498, 1051.6498, 1065.5970, 1131.6476, 1133.6560, 1136.6389, 1142.5725, 1142.5725, 1147.6736, 1151.6641, 1165.6772, 1165.6773, 1175.5778, 1194.5631, 1300.7844, 1302.7943, 1316.8119, 1334.8163, 1334.8163, 1351.5889, 1414.8141, 1434.8339, 1448.8483, 1448.8483, 1457.7133, 1475.6820, 1479.7203, 1479.7203, 1557.7402, 1567.6654, 1567.6654, 1583.9542, 1615.7332, 1617.9894, 1617.9894, 1630.7391, 1633.7523, 1697.8313, 1724.7576, 1732.0159, 1901.1652, 2015.1964, 2044.9374, 2184.3481, 2211.0227, 2283.0957, 2305.0806, 2530.2642

11. A0A4W2H2X1 **Mass:** 84971 **Score:** 49 **Expect:** 1.7 **Matches:** 8

Kinesin-like protein OS=Bos indicus x Bos taurus OX=30522 GN=KIF3B PE=3 SV=1

Observed	Mr(expt)	Mr(calc)	ppm	Start	End	Miss	Ions	Peptide
868.4995	867.4922	867.4273	74.9	48	55	0	---	K.GVAHEMPK.T
1033.6417	1032.6344	1032.6331	1.24	540	548	1	---	K.LFSKLQAVK.A
1045.5149	1044.5076	1044.5312	-22.57	252	261	1	---	R.QAKTGAQGER.L
1498.6241	1497.6168	1497.6770	-40.15	103	115	1	---	K.TYTMEGVRGDPEK.R + Oxidation (M)
1513.7238	1512.7165	1512.7460	-19.50	142	153	0	9	R.ASYLEIYQEEIR.D
1513.7238	1512.7165	1512.7460	-19.47	142	153	0	---	R.ASYLEIYQEEIR.D
1647.7585	1646.7512	1646.8007	-30.06	653	666	0	---	R.AENIMLLELDMPSR.T + Oxidation (M)
1647.7585	1646.7513	1646.8007	-30.04	653	666	0	27	R.AENIMLLELDMPSR.T + Oxidation (M)

No match to: 805.4537, 842.4627, 848.4779, 850.0055, 850.4871, 853.4691, 864.5048, 865.9789, 870.5005, 876.9857, 882.5121, 882.5121, 892.9567, 919.4136, 927.4397, 951.5660, 982.5261, 996.5410, 1017.6141, 1017.6141, 1019.6234, 1051.6498, 1051.6498, 1065.5970, 1131.6476, 1133.6560, 1136.6389, 1142.5725, 1142.5725, 1147.6736, 1151.6641, 1165.6772, 1165.6773, 1175.5778, 1194.5631, 1300.7844, 1302.7943, 1316.8119, 1334.8163, 1334.8163, 1351.5889, 1414.8141, 1434.8339, 1448.8483, 1448.8483, 1457.7133, 1475.6820, 1479.7203, 1479.7203, 1557.7402, 1567.6654, 1567.6654, 1583.9542, 1615.7332, 1617.9894, 1617.9894, 1630.7391, 1633.7523, 1697.8313, 1724.7576, 1732.0159, 1901.1652, 2015.1964, 2044.9374, 2184.3481, 2211.0227, 2283.0957, 2305.0806, 2530.2642

12. F1N020 **Mass:** 84971 **Score:** 49 **Expect:** 1.7 **Matches:** 8

Kinesin-like protein OS=Bos taurus OX=9913 GN=KIF3B PE=3 SV=2

Observed	Mr(expt)	Mr(calc)	ppm	Start	End	Miss	Ions	Peptide
868.4995	867.4922	867.4273	74.9	48	55	0	---	K.GVAHEMPK.T
1033.6417	1032.6344	1032.6331	1.24	540	548	1	---	K.LFSKLQAVK.A
1045.5149	1044.5076	1044.5312	-22.57	252	261	1	---	R.QAKTGAQGER.L
1498.6241	1497.6168	1497.6770	-40.15	103	115	1	---	K.TYTMEGVRGDPEK.R + Oxidation (M)
1513.7238	1512.7165	1512.7460	-19.50	142	153	0	9	R.ASYLEIYQEEIR.D
1513.7238	1512.7165	1512.7460	-19.47	142	153	0	---	R.ASYLEIYQEEIR.D
1647.7585	1646.7512	1646.8007	-30.06	653	666	0	---	R.AENIMLLELDMPSR.T + Oxidation (M)
1647.7585	1646.7513	1646.8007	-30.04	653	666	0	27	R.AENIMLLELDMPSR.T + Oxidation (M)

No match to: 805.4537, 842.4627, 848.4779, 850.0055, 850.4871, 853.4691, 864.5048, 865.9789, 870.5005, 876.9857, 882.5121, 882.5121, 892.9567, 919.4136, 927.4397, 951.5660, 982.5261, 996.5410, 1017.6141, 1017.6141, 1019.6234, 1051.6498, 1051.6498, 1065.5970, 1131.6476, 1133.6560, 1136.6389, 1142.5725, 1142.5725, 1147.6736, 1151.6641, 1165.6772, 1165.6773, 1175.5778, 1194.5631, 1300.7844, 1302.7943, 1316.8119, 1334.8163, 1334.8163, 1351.5889, 1414.8141, 1434.8339, 1448.8483, 1448.8483, 1457.7133, 1475.6820, 1479.7203, 1479.7203, 1557.7402, 1567.6654, 1567.6654, 1583.9542, 1615.7332, 1617.9894, 1617.9894, 1630.7391, 1633.7523, 1697.8313, 1724.7576, 1732.0159, 1901.1652, 2015.1964, 2044.9374, 2184.3481, 2211.0227, 2283.0957, 2305.0806, 2530.2642

13. A0A4W2F5Q7 **Mass:** 26142 **Score:** 41 **Expect:** 10 **Matches:** 7

Peroxiredoxin like 2A OS=Bos indicus x Bos taurus OX=30522 GN=PRXL2A PE=4 SV=1

Observed	Mr(expt)	Mr(calc)	ppm	Start	End	Miss	Ions	Peptide
996.5410	995.5337	995.5400	-6.27	119	126	1	---	K.EHIKNEVK.D
1065.5970	1064.5897	1064.5008	83.5	86	93	0	---	R.RPGCFLCR.E
1151.6641	1150.6568	1150.6234	29.1	60	69	1	---	K.TLEKDAVTFK.A
1194.5631	1193.5558	1193.5396	13.6	151	159	1	---	R.KMMFMGFVR.L + 3 Oxidation (M)
1300.7844	1299.7771	1299.7510	20.1	211	222	1	---	K.VNLTSVLEAARK.I
1414.8141	1413.8068	1413.6929	80.6	123	133	1	---	K.NEVKDFQPYFK.G
1557.7402	1556.7329	1556.8497	-75.00	72	85	1	---	K.ALWEKNGAVIMAVR.R

No match to: 805.4537, 842.4627, 848.4779, 850.0055, 850.4871, 853.4691, 864.5048, 865.9789, 868.4995, 870.5005, 876.9857, 882.5121, 882.5121, 892.9567, 919.4136, 927.4397, 951.5660, 982.5261, 1017.6141, 1017.6141, 1019.6234, 1033.6417, 1045.5149, 1051.6498, 1051.6498, 1131.6476, 1133.6560, 1136.6389, 1142.5725, 1142.5725, 1147.6736, 1165.6772, 1165.6773, 1175.5778, 1302.7943, 1316.8119, 1334.8163, 1334.8163, 1351.5889, 1434.8339, 1448.8483, 1448.8483, 1457.7133, 1475.6820, 1479.7203, 1479.7203, 1498.6241, 1513.7238, 1513.7238, 1567.6654, 1567.6654, 1583.9542, 1615.7332, 1617.9894, 1617.9894, 1630.7391, 1633.7523, 1647.7585, 1647.7585, 1697.8313, 1724.7576, 1732.0159, 1901.1652, 2015.1964, 2044.9374, 2184.3481, 2211.0227, 2283.0957, 2305.0806, 2530.2642

14. A0A3Q1LNN7 **Mass:** 68022 **Score:** 41 **Expect:** 11 **Matches:** 9

Serum albumin OS=Bos taurus OX=9913 GN=ALB PE=4 SV=1

Observed	Mr(expt)	Mr(calc)	ppm	Start	End	Miss	Ions	Peptide
927.4397	926.4324	926.4861	-57.97	161	167	0	---	K.YLYEIAR.R
1017.6141	1016.6068	1016.5727	33.6	212	220	1	---	K.VLTSSARQR.L
1017.6141	1016.6068	1016.5727	33.6	212	220	1	---	K.VLTSSARQR.L
1479.7203	1478.7130	1478.7881	-50.80	421	433	0	---	K.LGEYGFQNALIVR.Y
1479.7203	1478.7131	1478.7881	-50.77	421	433	0	---	K.LGEYGFQNALIVR.Y
1567.6654	1566.6581	1566.7354	-49.35	347	359	0	---	K.DAFLGSFLYEYSR.R
1567.6654	1566.6581	1566.7354	-49.34	347	359	0	22	K.DAFLGSFLYEYSR.R
1724.7576	1723.7503	1723.8273	-44.65	469	482	0	---	R.MPCTEDYLSLILNR.L
2044.9374	2043.9301	2044.0206	-44.28	168	183	1	---	R.RHPYFYAPELLYYANK.Y

No match to: 805.4537, 842.4627, 848.4779, 850.0055, 850.4871, 853.4691, 864.5048, 865.9789, 868.4995, 870.5005, 876.9857, 882.5121, 882.5121, 892.9567, 919.4136, 951.5660, 982.5261, 996.5410, 1019.6234, 1033.6417, 1045.5149, 1051.6498, 1051.6498, 1065.5970, 1131.6476, 1133.6560, 1136.6389, 1142.5725, 1142.5725, 1147.6736, 1151.6641, 1165.6772, 1165.6773, 1175.5778, 1194.5631, 1300.7844, 1302.7943, 1316.8119, 1334.8163, 1334.8163, 1351.5889, 1414.8141, 1434.8339, 1448.8483, 1448.8483, 1457.7133, 1475.6820, 1498.6241, 1513.7238, 1513.7238, 1557.7402, 1583.9542, 1615.7332, 1617.9894, 1630.7391, 1633.7523, 1647.7585, 1647.7585, 1697.8313, 1732.0159, 1901.1652, 2015.1964, 2184.3481, 2211.0227, 2283.0957, 2305.0806, 2530.2642

15. A0A452DI98 **Mass:** 15508 **Score:** 41 **Expect:** 12 **Matches:** 8

U6 snRNA-associated Sm-like protein LSm4 OS=Bos taurus OX=9913 GN=LSM4 PE=3 SV=1

Observed	Mr(expt)	Mr(calc)	ppm	Start	End	Miss	Ions	Peptide
805.4537	804.4464	804.4355	13.6	110	117	1	---	R.GVFGGRGR.G
864.5048	863.4975	863.4171	93.1	42	48	0	---	R.EVICTSR.D
927.4397	926.4324	926.5046	-77.93	116	125	1	---	R.GRGGIPGTGR.G
996.5410	995.5337	995.5512	-17.56	126	134	1	---	R.GQPEKKPGR.Q
1142.5725	1141.5652	1141.6204	-48.30	91	100	1	---	R.GGLQQQKQQK.G
1142.5725	1141.5652	1141.6204	-48.29	91	100	1	---	R.GGLQQQKQQK.G
1194.5631	1193.5558	1193.5724	-13.89	103	115	1	---	R.GMGGAGRGVFGGR.G + Oxidation (M)
1457.7133	1456.7060	1456.6744	21.7	53	62	1	---	K.FWRMPECYIR.G

PDF-XChange Click to buy NOW! www.docu-track.com

PDF-XChange Click to buy NOW! www.docu-track.com

No match to: 842.4627, 848.4779, 850.0055, 850.4871, 853.4691, 865.9789, 868.4995, 870.5005, 876.9857, 882.5121, 882.5121, 892.9567, 919.4136, 951.5660, 982.5261, 1017.6141, 1017.6141, 1019.6234, 1033.6417, 1045.5149, 1051.6498, 1051.6498, 1065.5970, 1131.6476, 1133.6560, 1136.6389, 1147.6736, 1151.6641, 1165.6772, 1165.6773, 1175.5778, 1300.7844, 1302.7943, 1316.8119, 1334.8163, 1334.8163, 1351.5889, 1414.8141, 1434.8339, 1448.8483, 1448.8483, 1475.6820, 1479.7203, 1479.7203, 1498.6241, 1513.7238, 1513.7238, 1557.7402, 1567.6654, 1567.6654, 1583.9542, 1615.7332, 1617.9894, 1617.9894, 1630.7391, 1633.7523, 1647.7585, 1647.7585, 1697.8313, 1724.7576, 1732.0159, 1901.1652, 2015.1964, 2044.9374, 2184.3481, 2211.0227, 2283.0957, 2305.0806, 2530.2642

16. A0A4W2FGN9 **Mass:** 123016 **Score:** 40 **Expect:** 12 **Matches:** 19

Coiled-coil domain containing 150 OS=Bos indicus x Bos taurus OX=30522 GN=CCDC150 PE=4 SV=1

Observed	Mr(expt)	Mr(calc)	ppm	Start	End	Miss	Ions	Peptide
805.4537	804.4464	804.4341	15.3	548 -	554	1	---	K.ITDSKNK.L
848.4779	847.4706	847.4664	4.95	642 -	648	1	---	K.KVGNFQR.Q
864.5048	863.4975	863.4422	64.0	826 -	832	1	---	R.EVTEMKK.A
868.4995	867.4922	867.4450	54.4	766 -	772	0	---	K.LDHVQEK.L
919.4136	918.4063	918.4043	2.24	1010 -	1016	1	---	R.EEDQDKR.H
927.4397	926.4324	926.4094	24.9	941 -	948	0	---	R.SQYDTSAR.N
1147.6736	1146.6663	1146.6067	52.0	893 -	901	1	---	R.MKQIETELR.Q
1300.7844	1299.7771	1299.7510	20.1	441 -	451	1	---	K.ELLESTIARLR.G
1448.8483	1447.8410	1447.7453	66.1	30 -	41	1	---	R.MRIVEEQTSSLR.D
1448.8483	1447.8410	1447.7453	66.1	30 -	41	1	---	R.MRIVEEQTSSLR.D
1475.6820	1474.6747	1474.6535	14.4	163 -	174	1	---	K.EEEDKAQDEVQR.L
1479.7203	1478.7130	1478.7551	-28.48	985 -	996	1	---	K.CRFDGLQLELTK.N
1479.7203	1478.7131	1478.7551	-28.46	985 -	996	1	---	K.CRFDGLQLELTK.N
1498.6241	1497.6168	1497.6639	-31.43	90 -	100	1	---	K.NCEFLVNRMCR.L
1557.7402	1556.7329	1556.8232	-58.00	660 -	673	0	---	K.VTIMLENVLASHSK.M + Oxidation (M)
1630.7391	1629.7318	1629.8759	-88.43	895 -	907	1	---	K.QIETELRQMELIK.D
2184.3481	2183.3408	2183.1368	93.4	747 -	765	1	---	R.KLAMSLEQALQTNNHLQTK.L + Oxidation (M)
2211.0227	2210.0154	2210.0498	-15.54	964 -	981	1	---	R.QELENRCQITANLEEAHR.W
2283.0957	2282.0884	2282.1324	-19.29	359 -	377	1	---	K.MENTRIIADHQAILQEEQK.M + Oxidation (M)

No match to: 842.4627, 850.0055, 850.4871, 853.4691, 865.9789, 870.5005, 876.9857, 882.5121, 882.5121, 892.9567, 951.5660, 982.5261, 996.5410, 1017.6141, 1017.6141, 1019.6234, 1033.6417, 1045.5149, 1051.6498, 1051.6498, 1065.5970, 1131.6476, 1133.6560, 1136.6389, 1142.5725, 1142.5725, 1151.6641, 1165.6772, 1165.6773, 1175.5778, 1194.5631, 1302.7943, 1316.8119, 1334.8163, 1334.8163, 1351.5889, 1414.8141, 1434.8339, 1457.7133, 1513.7238, 1513.7238, 1567.6654, 1567.6654, 1583.9542, 1615.7332, 1617.9894, 1617.9894, 1633.7523, 1647.7585, 1647.7585, 1697.8313, 1724.7576, 1732.0159, 1901.1652, 2015.1964, 2044.9374, 2305.0806, 2530.2642

17. A0A4W2I014 **Mass:** 105104 **Score:** 40 **Expect:** 12 **Matches:** 16

Protein phosphatase 1 regulatory subunit 21 OS=Bos indicus x Bos taurus OX=30522 GN=PPP1R21 PE=4 SV=1

Observed	Mr(expt)	Mr(calc)	ppm	Start	End	Miss	Ions	Peptide
842.4627	841.4554	841.4480	8.84	49 -	56	0	---	K.MAPLAPSR.G
853.4691	852.4618	852.4453	19.3	286 -	292	0	---	K.HVEAELR.S
1033.6417	1032.6344	1032.5451	86.5	334 -	342	1	---	K.SQTLEKEAK.E
1131.6476	1130.6403	1130.5931	41.7	910 -	918	1	---	K.QREEIDTLK.M
1147.6736	1146.6663	1146.6357	26.7	700 -	709	1	---	R.RVLLSSTESR.E
1165.6772	1164.6699	1164.5696	86.2	860 -	869	1	---	K.SKEALTEEMK.V
1165.6773	1164.6700	1164.5696	86.2	860 -	869	1	---	K.SKEALTEEMK.V
1457.7133	1456.7060	1456.7522	-31.66	710 -	722	0	---	R.EGLAQQVQQSLEK.I
1567.6654	1566.6581	1566.7613	-65.84	840 -	852	1	---	K.SVHFYAECRALSK.R
1567.6654	1566.6581	1566.7613	-65.83	840 -	852	1	---	K.SVHFYAECRALSK.R
1617.9894	1616.9821	1616.8734	67.3	755 -	770	1	---	K.LKSTSSGQVVGVAQEK.A
1617.9894	1616.9821	1616.8734	67.3	755 -	770	1	---	K.LKSTSSGQVVGVAQEK.A
1633.7523	1632.7450	1632.8592	-69.94	42 -	56	1	---	R.HLGPCVKMAPLAPSR.G
1697.8313	1696.8240	1696.8090	8.86	148 -	163	1	---	R.AGEAMASAELQGKYQK.L + Oxidation (M)
2015.1964	2014.1891	2014.0346	76.7	490 -	505	1	---	K.LKTFSEHLTSYICFLR.K
2184.3481	2183.3408	2183.2201	55.3	549 -	568	0	---	K.LQTYIALLALPSTEPDGLLR.T

No match to: 805.4537, 848.4779, 850.0055, 850.4871, 864.5048, 865.9789, 868.4995, 870.5005, 876.9857, 882.5121, 882.5121, 892.9567, 919.4136, 927.4397, 951.5660, 982.5261, 996.5410, 1017.6141, 1017.6141, 1019.6234, 1045.5149, 1051.6498, 1051.6498, 1065.5970, 1133.6560, 1136.6389, 1142.5725, 1142.5725, 1151.6641, 1175.5778, 1194.5631, 1300.7844, 1302.7943, 1316.8119, 1334.8163, 1334.8163, 1351.5889, 1414.8141, 1434.8339, 1448.8483, 1448.8483, 1475.6820, 1479.7203, 1479.7203, 1498.6241, 1513.7238, 1513.7238, 1557.7402, 1583.9542, 1615.7332, 1630.7391, 1647.7585, 1647.7585, 1724.7576, 1732.0159, 1901.1652, 2044.9374, 2211.0227, 2283.0957, 2305.0806, 2530.2642

18. A0A4W2CGR1 **Mass:** 105032 **Score:** 40 **Expect:** 12 **Matches:** 16

Protein phosphatase 1 regulatory subunit 21 OS=Bos indicus x Bos taurus OX=30522 GN=PPP1R21 PE=4 SV=1

Observed	Mr(expt)	Mr(calc)	ppm	Start	End	Miss	Ions	Peptide
842.4627	841.4554	841.4480	8.84	49 -	56	0	---	K.MAPLAPSR.G
853.4691	852.4618	852.4453	19.3	286 -	292	0	---	K.HVEAELR.S
1033.6417	1032.6344	1032.5451	86.5	334 -	342	1	---	K.SQTLEKEAK.E
1131.6476	1130.6403	1130.5931	41.7	910 -	918	1	---	K.QREEIDTLK.M
1147.6736	1146.6663	1146.6357	26.7	700 -	709	1	---	R.RVLLSSTESR.E
1165.6772	1164.6699	1164.5696	86.2	860 -	869	1	---	K.SKEALTEEMK.V
1165.6773	1164.6700	1164.5696	86.2	860 -	869	1	---	K.SKEALTEEMK.V
1457.7133	1456.7060	1456.7522	-31.66	710 -	722	0	---	R.EGLAQQVQQSLEK.I
1567.6654	1566.6581	1566.7613	-65.84	840 -	852	1	---	K.SVHFYAECRALSK.R
1567.6654	1566.6581	1566.7613	-65.83	840 -	852	1	---	K.SVHFYAECRALSK.R
1617.9894	1616.9821	1616.8734	67.3	755 -	770	1	---	K.LKSTSSGQVVGVAQEK.A
1617.9894	1616.9821	1616.8734	67.3	755 -	770	1	---	K.LKSTSSGQVVGVAQEK.A
1633.7523	1632.7450	1632.8592	-69.94	42 -	56	1	---	R.HLGPCVKMAPLAPSR.G
1697.8313	1696.8240	1696.8090	8.86	148 -	163	1	---	R.AGEAMASAELQGKYQK.L + Oxidation (M)
2015.1964	2014.1891	2014.0346	76.7	490 -	505	1	---	K.LKTFSEHLTSYICFLR.K
2184.3481	2183.3408	2183.2201	55.3	549 -	568	0	---	K.LQTYIALLALPSTEPDGLLR.T

No match to: 805.4537, 848.4779, 850.0055, 850.4871, 864.5048, 865.9789, 868.4995, 870.5005, 876.9857, 882.5121, 882.5121, 892.9567, 919.4136, 927.4397, 951.5660, 982.5261, 996.5410, 1017.6141, 1017.6141, 1019.6234, 1045.5149, 1051.6498, 1051.6498, 1065.5970, 1133.6560, 1136.6389, 1142.5725, 1142.5725, 1151.6641, 1175.5778, 1194.5631, 1300.7844, 1302.7943, 1316.8119, 1334.8163, 1334.8163, 1351.5889, 1414.8141, 1434.8339, 1448.8483, 1448.8483, 1475.6820, 1479.7203, 1479.7203, 1498.6241, 1513.7238, 1513.7238, 1557.7402, 1583.9542, 1615.7332, 1630.7391, 1647.7585, 1647.7585, 1724.7576, 1732.0159, 1901.1652, 2044.9374, 2211.0227, 2283.0957, 2305.0806, 2530.2642

19. A0A4W2DDL5 **Mass:** 70207 **Score:** 40 **Expect:** 13 **Matches:** 9

Albumin OS=Bos indicus x Bos taurus OX=30522 GN=ALB PE=4 SV=1

Observed	Mr(expt)	Mr(calc)	ppm	Start	End	Miss	Ions	Peptide
927.4397	926.4324	926.4861	-57.97	152 -	158	0	---	K.YLYEIAR.R
1017.6141	1016.6068	1016.5727	33.6	203 -	211	1	---	K.VLTSSARQR.L

PDF-XChange Click to buy NOW! www.docu-track.com

1017.6141	1016.6068	1016.5727	33.6	203	211	1	---	K.VLTSSARQR.L
1479.7203	1478.7130	1478.7881	-50.80	412	424	0	---	K.LGEYGFQNALIVR.Y
1479.7203	1478.7131	1478.7881	-50.77	412	424	0	---	K.LGEYGFQNALIVR.Y
1567.6654	1566.6581	1566.7354	-49.35	338	350	0	---	K.DAFLGSFLYEYSR.R
1567.6654	1566.6581	1566.7354	-49.34	338	350	0	22	K.DAFLGSFLYEYSR.R
1724.7576	1723.7503	1723.8273	-44.65	460	473	0	---	R.MPCTEDYLSLILNR.L
2044.9374	2043.9301	2044.0206	-44.28	159	174	1	---	R.RHPYFYAPELLYYANK.Y

No match to: 805.4537, 842.4627, 848.4779, 850.0055, 850.4871, 853.4691, 864.5048, 865.9789, 868.4995, 870.5005, 876.9857, 882.5121, 882.5121, 892.9567, 919.4136, 951.5660, 982.5261, 996.5410, 1019.6234, 1033.6417, 1045.5149, 1051.6498, 1051.6498, 1065.5970, 1131.6476, 1133.6560, 1136.6389, 1142.5725, 1142.5725, 1147.6736, 1151.6641, 1165.6772, 1165.6773, 1175.5778, 1194.5631, 1300.7844, 1302.7943, 1316.8119, 1334.8163, 1334.8163, 1351.5889, 1414.8141, 1434.8339, 1448.8483, 1448.8483, 1457.7133, 1475.6820, 1498.6241, 1513.7238, 1513.7238, 1557.7402, 1583.9542, 1615.7332, 1617.9894, 1617.9894, 1630.7391, 1633.7523, 1647.7585, 1647.7585, 1697.8313, 1732.0159, 1901.1652, 2015.1964, 2184.3481, 2211.0227, 2283.0957, 2305.0806, 2530.2642

20. A0A140T897 **Mass:** 71274 **Score:** 40 **Expect:** 13 **Matches:** 9
Serum albumin OS=Bos taurus OX=9913 GN=ALB PE=4 SV=1

Observed	Mr(expt)	Mr(calc)	ppm	Start	End	Miss	Ions	Peptide
927.4397	926.4324	926.4861	-57.97	161	167	0	---	K.YLYEIAR.R
1017.6141	1016.6068	1016.5727	33.6	212	220	1	---	K.VLTSSARQR.L
1017.6141	1016.6068	1016.5727	33.6	212	220	1	---	K.VLTSSARQR.L
1479.7203	1478.7130	1478.7881	-50.80	421	433	0	---	K.LGEYGFQNALIVR.Y
1479.7203	1478.7131	1478.7881	-50.77	421	433	0	---	K.LGEYGFQNALIVR.Y
1567.6654	1566.6581	1566.7354	-49.35	347	359	0	---	K.DAFLGSFLYEYSR.R
1567.6654	1566.6581	1566.7354	-49.34	347	359	0	22	K.DAFLGSFLYEYSR.R
1724.7576	1723.7503	1723.8273	-44.65	469	482	0	---	R.MPCTEDYLSLILNR.L
2044.9374	2043.9301	2044.0206	-44.28	168	183	1	---	R.RHPYFYAPELLYYANK.Y

No match to: 805.4537, 842.4627, 848.4779, 850.0055, 850.4871, 853.4691, 864.5048, 865.9789, 868.4995, 870.5005, 876.9857, 882.5121, 882.5121, 892.9567, 919.4136, 951.5660, 982.5261, 996.5410, 1019.6234, 1033.6417, 1045.5149, 1051.6498, 1051.6498, 1065.5970, 1131.6476, 1133.6560, 1136.6389, 1142.5725, 1142.5725, 1147.6736, 1151.6641, 1165.6772, 1165.6773, 1175.5778, 1194.5631, 1300.7844, 1302.7943, 1316.8119, 1334.8163, 1334.8163, 1351.5889, 1414.8141, 1434.8339, 1448.8483, 1448.8483, 1457.7133, 1475.6820, 1498.6241, 1513.7238, 1513.7238, 1557.7402, 1583.9542, 1615.7332, 1617.9894, 1617.9894, 1630.7391, 1633.7523, 1647.7585, 1647.7585, 1697.8313, 1732.0159, 1901.1652, 2015.1964, 2184.3481, 2211.0227, 2283.0957, 2305.0806, 2530.2642

21. A0A4W2EVR9 **Mass:** 71453 **Score:** 40 **Expect:** 13 **Matches:** 9
Albumin OS=Bos indicus x Bos taurus OX=30522 GN=ALB PE=4 SV=1

Observed	Mr(expt)	Mr(calc)	ppm	Start	End	Miss	Ions	Peptide
927.4397	926.4324	926.4861	-57.97	161	167	0	---	K.YLYEIAR.R
1017.6141	1016.6068	1016.5727	33.6	212	220	1	---	K.VLTSSARQR.L
1017.6141	1016.6068	1016.5727	33.6	212	220	1	---	K.VLTSSARQR.L
1479.7203	1478.7130	1478.7881	-50.80	421	433	0	---	K.LGEYGFQNALIVR.Y
1479.7203	1478.7131	1478.7881	-50.77	421	433	0	---	K.LGEYGFQNALIVR.Y
1567.6654	1566.6581	1566.7354	-49.35	347	359	0	---	K.DAFLGSFLYEYSR.R
1567.6654	1566.6581	1566.7354	-49.34	347	359	0	22	K.DAFLGSFLYEYSR.R
1724.7576	1723.7503	1723.8273	-44.65	469	482	0	---	R.MPCTEDYLSLILNR.L
2044.9374	2043.9301	2044.0206	-44.28	168	183	1	---	R.RHPYFYAPELLYYANK.Y

No match to: 805.4537, 842.4627, 848.4779, 850.0055, 850.4871, 853.4691, 864.5048, 865.9789, 868.4995, 870.5005, 876.9857, 882.5121, 882.5121, 892.9567, 919.4136, 951.5660, 982.5261, 996.5410, 1019.6234, 1033.6417, 1045.5149, 1051.6498, 1051.6498, 1065.5970, 1131.6476, 1133.6560, 1136.6389, 1142.5725, 1142.5725, 1147.6736, 1151.6641, 1165.6772, 1165.6773, 1175.5778, 1194.5631, 1300.7844, 1302.7943, 1316.8119, 1334.8163, 1334.8163, 1351.5889, 1414.8141, 1434.8339, 1448.8483, 1448.8483, 1457.7133, 1475.6820, 1498.6241, 1513.7238, 1513.7238, 1557.7402, 1583.9542, 1615.7332, 1617.9894, 1617.9894, 1630.7391, 1633.7523, 1647.7585, 1647.7585, 1697.8313, 1732.0159, 1901.1652, 2015.1964, 2184.3481, 2211.0227, 2283.0957, 2305.0806, 2530.2642

22. A0A4W2DGX3 **Mass:** 13967 **Score:** 40 **Expect:** 14 **Matches:** 8
U6 snRNA-associated Sm-like protein LSm4 OS=Bos indicus x Bos taurus OX=30522 GN=LSM4 PE=3 SV=1

Observed	Mr(expt)	Mr(calc)	ppm	Start	End	Miss	Ions	Peptide
805.4537	804.4464	804.4355	13.6	96	103	1	---	R.GVFGGRGR.G
864.5048	863.4975	863.4171	93.1	28	34	0	---	R.EVICTSR.D
927.4397	926.4324	926.5046	-77.93	102	111	1	---	R.GRGGIPGTGR.G
996.5410	995.5337	995.5512	-17.56	112	120	1	---	R.GQPEKKPGR.Q
1142.5725	1141.5652	1141.6204	-48.30	77	86	1	---	R.GGLQQQKQQK.G
1142.5725	1141.5652	1141.6204	-48.29	77	86	1	---	R.GGLQQQKQQK.G
1194.5631	1193.5558	1193.5724	-13.89	89	101	1	---	R.GMGGAGRGVFGGR.G + Oxidation (M)
1457.7133	1456.7060	1456.6744	21.7	39	48	1	---	K.FWRMPECYIR.G

No match to: 842.4627, 848.4779, 850.0055, 850.4871, 853.4691, 865.9789, 868.4995, 870.5005, 876.9857, 882.5121, 882.5121, 892.9567, 919.4136, 951.5660, 982.5261, 1017.6141, 1017.6141, 1019.6234, 1033.6417, 1045.5149, 1051.6498, 1051.6498, 1065.5970, 1131.6476, 1133.6560, 1136.6389, 1147.6736, 1151.6641, 1165.6772, 1165.6773, 1175.5778, 1300.7844, 1302.7943, 1316.8119, 1334.8163, 1334.8163, 1351.5889, 1414.8141, 1434.8339, 1448.8483, 1448.8483, 1475.6820, 1479.7203, 1479.7203, 1498.6241, 1513.7238, 1513.7238, 1557.7402, 1567.6654, 1567.6654, 1583.9542, 1615.7332, 1617.9894, 1617.9894, 1630.7391, 1633.7523, 1647.7585, 1647.7585, 1697.8313, 1724.7576, 1732.0159, 1901.1652, 2015.1964, 2044.9374, 2184.3481, 2211.0227, 2283.0957, 2305.0806, 2530.2642

23. A0A4W2HT62 **Mass:** 15511 **Score:** 39 **Expect:** 16 **Matches:** 8
U6 snRNA-associated Sm-like protein LSm4 OS=Bos indicus x Bos taurus OX=30522 GN=LSM4 PE=3 SV=1

Observed	Mr(expt)	Mr(calc)	ppm	Start	End	Miss	Ions	Peptide
805.4537	804.4464	804.4355	13.6	110	117	1	---	R.GVFGGRGR.G
864.5048	863.4975	863.4171	93.1	42	48	0	---	R.EVICTSR.D
927.4397	926.4324	926.5046	-77.93	116	125	1	---	R.GRGGIPGTGR.G
996.5410	995.5337	995.5512	-17.56	126	134	1	---	R.GQPEKKPGR.Q
1142.5725	1141.5652	1141.6204	-48.30	91	100	1	---	R.GGLQQQKQQK.G
1142.5725	1141.5652	1141.6204	-48.29	91	100	1	---	R.GGLQQQKQQK.G
1194.5631	1193.5558	1193.5724	-13.89	103	115	1	---	R.GMGGAGRGVFGGR.G + Oxidation (M)
1457.7133	1456.7060	1456.6744	21.7	53	62	1	---	K.FWRMPECYIR.G

No match to: 842.4627, 848.4779, 850.0055, 850.4871, 853.4691, 865.9789, 868.4995, 870.5005, 876.9857, 882.5121, 882.5121, 892.9567, 919.4136, 951.5660, 982.5261, 1017.6141, 1017.6141, 1019.6234, 1033.6417, 1045.5149, 1051.6498, 1051.6498, 1065.5970, 1131.6476, 1133.6560, 1136.6389, 1147.6736, 1151.6641, 1165.6772, 1165.6773, 1175.5778, 1300.7844, 1302.7943, 1316.8119, 1334.8163, 1334.8163, 1351.5889, 1414.8141, 1434.8339, 1448.8483, 1448.8483, 1475.6820, 1479.7203, 1479.7203, 1498.6241, 1513.7238, 1513.7238, 1557.7402, 1567.6654, 1567.6654, 1583.9542, 1615.7332, 1617.9894, 1617.9894, 1630.7391, 1633.7523, 1647.7585, 1647.7585, 1697.8313, 1724.7576, 1732.0159, 1901.1652, 2015.1964, 2044.9374, 2184.3481, 2211.0227, 2283.0957, 2305.0806, 2530.2642

24. Q3ZBK6 **Mass:** 15511 **Score:** 39 **Expect:** 16 **Matches:** 8
U6 snRNA-associated Sm-like protein LSm4 OS=Bos taurus OX=9913 GN=LSM4 PE=2 SV=1

PDF-XChange Click to buy NOW! www.docu-track.com

Observed	Mr(expt)	Mr(calc)	ppm	Start	End	Miss	Ions	Peptide
805.4537	804.4464	804.4355	13.6	110	117	1	---	R.GVFGGRGR.G
864.5048	863.4975	863.4171	93.1	42	48	0	---	R.EVICTSR.D
927.4397	926.4324	926.5046	-77.93	116	125	1	---	R.GRGGIPGTGR.G
996.5410	995.5337	995.5512	-17.56	126	134	1	---	R.GQPEKKPGR.Q
1142.5725	1141.5652	1141.6204	-48.30	91	100	1	---	R.GGLQQQKQQK.G
1142.5725	1141.5652	1141.6204	-48.29	91	100	1	---	R.GGLQQQKQQK.G
1194.5631	1193.5558	1193.5724	-13.89	103	115	1	---	R.GMGGAGRGVFGGR.G + Oxidation (M)
1457.7133	1456.7060	1456.6744	21.7	53	62	1	---	K.FWRMPECYIR.G

No match to: 842.4627, 848.4779, 850.0055, 850.4871, 853.4691, 865.9789, 868.4995, 870.5005, 876.9857, 882.5121, 882.5121, 892.9567, 919.4136, 951.5660, 982.5261, 1017.6141, 1017.6141, 1019.6234, 1033.6417, 1045.5149, 1051.6498, 1051.6498, 1065.5970, 1131.6476, 1133.6560, 1136.6389, 1147.6736, 1151.6641, 1165.6772, 1165.6773, 1175.5778, 1300.7844, 1302.7943, 1316.8119, 1334.8163, 1334.8163, 1351.5889, 1414.8141, 1434.8339, 1448.8483, 1448.8483, 1475.6820, 1479.7203, 1479.7203, 1498.6241, 1513.7238, 1513.7238, 1557.7402, 1567.6654, 1567.6654, 1583.9542, 1615.7332, 1617.9894, 1617.9894, 1630.7391, 1633.7523, 1647.7585, 1647.7585, 1697.8313, 1724.7576, 1732.0159, 1901.1652, 2015.1964, 2044.9374, 2184.3481, 2211.0227, 2283.0957, 2305.0806, 2530.2642

25. A0A4W2EW60 **Mass:** 76020 **Score:** 39 **Expect:** 17 **Matches:** 9
Albumin OS=Bos indicus x Bos taurus OX=30522 GN=ALB PE=4 SV=1

Observed	Mr(expt)	Mr(calc)	ppm	Start	End	Miss	Ions	Peptide
927.4397	926.4324	926.4861	-57.97	161	167	0	---	K.YLYEIAR.R
1017.6141	1016.6068	1016.5727	33.6	212	220	1	---	K.VLTSSARQR.L
1017.6141	1016.6068	1016.5727	33.6	212	220	1	---	K.VLTSSARQR.L
1479.7203	1478.7130	1478.7881	-50.80	421	433	0	---	K.LGEYGFQNALIVR.Y
1479.7203	1478.7131	1478.7881	-50.77	421	433	0	---	K.LGEYGFQNALIVR.Y
1567.6654	1566.6581	1566.7354	-49.35	347	359	0	---	K.DAFLGSFLYEYSR.R
1567.6654	1566.6581	1566.7354	-49.34	347	359	0	22	K.DAFLGSFLYEYSR.R
1724.7576	1723.7503	1723.8273	-44.65	469	482	0	---	R.MPCTEDYLSLILNR.L
2044.9374	2043.9301	2044.0206	-44.28	168	183	1	---	R.RHPYFYAPELLYYANK.Y

No match to: 805.4537, 842.4627, 848.4779, 850.0055, 850.4871, 853.4691, 864.5048, 865.9789, 868.4995, 870.5005, 876.9857, 882.5121, 882.5121, 892.9567, 919.4136, 951.5660, 982.5261, 996.5410, 1019.6234, 1033.6417, 1045.5149, 1051.6498, 1051.6498, 1065.5970, 1131.6476, 1133.6560, 1136.6389, 1142.5725, 1142.5725, 1147.6736, 1151.6641, 1165.6772, 1165.6773, 1175.5778, 1194.5631, 1300.7844, 1302.7943, 1316.8119, 1334.8163, 1334.8163, 1351.5889, 1414.8141, 1434.8339, 1448.8483, 1448.8483, 1457.7133, 1475.6820, 1498.6241, 1513.7238, 1513.7238, 1557.7402, 1583.9542, 1615.7332, 1617.9894, 1617.9894, 1630.7391, 1633.7523, 1647.7585, 1647.7585, 1697.8313, 1732.0159, 1901.1652, 2015.1964, 2184.3481, 2211.0227, 2283.0957, 2305.0806, 2530.2642

26. A0A4W2D8T3 **Mass:** 76089 **Score:** 39 **Expect:** 18 **Matches:** 9
Albumin OS=Bos indicus x Bos taurus OX=30522 GN=ALB PE=4 SV=1

Observed	Mr(expt)	Mr(calc)	ppm	Start	End	Miss	Ions	Peptide
927.4397	926.4324	926.4861	-57.97	209	215	0	---	K.YLYEIAR.R
1017.6141	1016.6068	1016.5727	33.6	260	268	1	---	K.VLTSSARQR.L
1017.6141	1016.6068	1016.5727	33.6	260	268	1	---	K.VLTSSARQR.L
1479.7203	1478.7130	1478.7881	-50.80	469	481	0	---	K.LGEYGFQNALIVR.Y
1479.7203	1478.7131	1478.7881	-50.77	469	481	0	---	K.LGEYGFQNALIVR.Y
1567.6654	1566.6581	1566.7354	-49.35	395	407	0	---	K.DAFLGSFLYEYSR.R
1567.6654	1566.6581	1566.7354	-49.34	395	407	0	22	K.DAFLGSFLYEYSR.R
1724.7576	1723.7503	1723.8273	-44.65	517	530	0	---	R.MPCTEDYLSLILNR.L
2044.9374	2043.9301	2044.0206	-44.28	216	231	1	---	R.RHPYFYAPELLYYANK.Y

No match to: 805.4537, 842.4627, 848.4779, 850.0055, 850.4871, 853.4691, 864.5048, 865.9789, 868.4995, 870.5005, 876.9857, 882.5121, 882.5121, 892.9567, 919.4136, 951.5660, 982.5261, 996.5410, 1019.6234, 1033.6417, 1045.5149, 1051.6498, 1051.6498, 1065.5970, 1131.6476, 1133.6560, 1136.6389, 1142.5725, 1142.5725, 1147.6736, 1151.6641, 1165.6772, 1165.6773, 1175.5778, 1194.5631, 1300.7844, 1302.7943, 1316.8119, 1334.8163, 1334.8163, 1351.5889, 1414.8141, 1434.8339, 1448.8483, 1448.8483, 1457.7133, 1475.6820, 1498.6241, 1513.7238, 1513.7238, 1557.7402, 1583.9542, 1615.7332, 1617.9894, 1617.9894, 1630.7391, 1633.7523, 1647.7585, 1647.7585, 1697.8313, 1732.0159, 1901.1652, 2015.1964, 2184.3481, 2211.0227, 2283.0957, 2305.0806, 2530.2642

27. A0A4W2HAU5 **Mass:** 24454 **Score:** 38 **Expect:** 21 **Matches:** 7
Peroxiredoxin like 2A OS=Bos indicus x Bos taurus OX=30522 GN=PRXL2A PE=4 SV=1

Observed	Mr(expt)	Mr(calc)	ppm	Start	End	Miss	Ions	Peptide
996.5410	995.5337	995.5400	-6.27	104	111	1	---	K.EHIKNEVK.D
1065.5970	1064.5897	1064.5008	83.5	71	78	0	---	R.RPGCFLCR.E
1151.6641	1150.6568	1150.6234	29.1	45	54	1	---	K.TLEKDAVTFK.A
1194.5631	1193.5558	1193.5396	13.6	136	144	1	---	R.KMMFMGFVR.L + 3 Oxidation (M)
1300.7844	1299.7771	1299.7510	20.1	196	207	1	---	K.VNLTSVLEAARK.I
1414.8141	1413.8068	1413.6929	80.6	108	118	1	---	K.NEVKDFQPYFK.G
1557.7402	1556.7329	1556.8497	-75.00	57	70	1	---	K.ALWEKNGAVIMAVR.R

No match to: 805.4537, 842.4627, 848.4779, 850.0055, 850.4871, 853.4691, 864.5048, 865.9789, 868.4995, 870.5005, 876.9857, 882.5121, 882.5121, 892.9567, 919.4136, 927.4397, 951.5660, 982.5261, 1017.6141, 1017.6141, 1019.6234, 1033.6417, 1045.5149, 1051.6498, 1051.6498, 1131.6476, 1133.6560, 1136.6389, 1142.5725, 1142.5725, 1147.6736, 1165.6772, 1165.6773, 1175.5778, 1302.7943, 1316.8119, 1334.8163, 1334.8163, 1351.5889, 1434.8339, 1448.8483, 1448.8483, 1457.7133, 1475.6820, 1479.7203, 1479.7203, 1498.6241, 1513.7238, 1513.7238, 1567.6654, 1567.6654, 1583.9542, 1615.7332, 1617.9894, 1617.9894, 1630.7391, 1633.7523, 1647.7585, 1647.7585, 1697.8313, 1724.7576, 1732.0159, 1901.1652, 2015.1964, 2044.9374, 2184.3481, 2211.0227, 2283.0957, 2305.0806, 2530.2642

28. Q3ZBK2 **Mass:** 24454 **Score:** 38 **Expect:** 21 **Matches:** 7
Peroxiredoxin-like 2A OS=Bos taurus OX=9913 GN=PRXL2A PE=2 SV=1

Observed	Mr(expt)	Mr(calc)	ppm	Start	End	Miss	Ions	Peptide
996.5410	995.5337	995.5400	-6.27	104	111	1	---	K.EHIKNEVK.D
1065.5970	1064.5897	1064.5008	83.5	71	78	0	---	R.RPGCFLCR.E
1151.6641	1150.6568	1150.6234	29.1	45	54	1	---	K.TLEKDAVTFK.A
1194.5631	1193.5558	1193.5396	13.6	136	144	1	---	R.KMMFMGFVR.L + 3 Oxidation (M)
1300.7844	1299.7771	1299.7510	20.1	196	207	1	---	K.VNLTSVLEAARK.I
1414.8141	1413.8068	1413.6929	80.6	108	118	1	---	K.NEVKDFQPYFK.G
1557.7402	1556.7329	1556.8497	-75.00	57	70	1	---	K.ALWEKNGAVIMAVR.R

No match to: 805.4537, 842.4627, 848.4779, 850.0055, 850.4871, 853.4691, 864.5048, 865.9789, 868.4995, 870.5005, 876.9857, 882.5121, 882.5121, 892.9567, 919.4136, 927.4397, 951.5660, 982.5261, 1017.6141, 1017.6141, 1019.6234, 1033.6417, 1045.5149, 1051.6498, 1051.6498, 1131.6476, 1133.6560, 1136.6389, 1142.5725, 1142.5725, 1147.6736, 1165.6772, 1165.6773, 1175.5778, 1302.7943, 1316.8119, 1334.8163, 1334.8163, 1351.5889, 1434.8339, 1448.8483, 1448.8483, 1457.7133, 1475.6820, 1479.7203, 1479.7203, 1498.6241, 1513.7238, 1513.7238, 1567.6654, 1567.6654, 1583.9542, 1615.7332, 1617.9894, 1617.9894, 1630.7391, 1633.7523, 1647.7585, 1647.7585, 1697.8313, 1724.7576, 1732.0159, 1901.1652, 2015.1964, 2044.9374, 2184.3481, 2211.0227, 2283.0957, 2305.0806, 2530.2642

29. A0A4W2H5Z2 **Mass:** 33370 **Score:** 37 **Expect:** 26 **Matches:** 7
Ketohexokinase OS=Bos indicus x Bos taurus OX=30522 GN=KHK PE=4 SV=1

PDF-XChange Click to buy NOW! www.docu-track.com

PDF-XChange Click to buy NOW! www.docu-track.com

PDF-XChange Click to buy NOW! www.docu-track.com

Observed	Mr(expt)	Mr(calc)	ppm	Start	End	Miss	Ions	Peptide
850.4871	849.4798	849.4821	-2.65	210	- 216	1	---	R.GLYSRVR.K
1065.5970	1064.5897	1064.4961	87.9	289	- 298	0	---	K.CGLQGFDGIV.-
1133.6560	1132.6487	1132.5560	81.9	32	- 39	1	---	R.CLSQRWQR.G
1136.6389	1135.6316	1135.5873	39.0	124	- 132	1	---	K.DFEKVELTR.F
1615.7332	1614.7259	1614.8227	-59.91	78	- 91	1	---	R.QRGVDVSQVAWQSR.G
1630.7391	1629.7318	1629.8376	-64.89	66	- 79	1	---	R.GHVADFLVADFRQR.G
1697.8313	1696.8240	1696.8025	12.7	273	- 287	1	---	K.SMQEALRFGCQVAGK.K + Oxidation (M)

No match to: 805.4537, 842.4627, 848.4779, 850.0055, 853.4691, 864.5048, 865.9789, 868.4995, 870.5005, 876.9857, 882.5121, 882.5121, 892.9567, 919.4136, 927.4397, 951.5660, 982.5261, 996.5410, 1017.6141, 1017.6141, 1019.6234, 1033.6417, 1045.5149, 1051.6498, 1051.6498, 1131.6476, 1142.5725, 1142.5725, 1147.6736, 1151.6641, 1165.6772, 1165.6773, 1175.5778, 1194.5631, 1300.7844, 1302.7943, 1316.8119, 1334.8163, 1334.8163, 1351.5889, 1414.8141, 1434.8339, 1448.8483, 1448.8483, 1457.7133, 1475.6820, 1479.7203, 1479.7203, 1498.6241, 1513.7238, 1513.7238, 1557.7402, 1567.6654, 1567.6654, 1583.9542, 1617.9894, 1617.9894, 1633.7523, 1647.7585, 1647.7585, 1724.7576, 1732.0159, 1901.1652, 2015.1964, 2044.9374, 2184.3481, 2211.0227, 2283.0957, 2305.0806, 2530.2642

30. P02769 **Mass:** 71244 **Score:** 36 **Expect:** 37 **Matches:** 7

Serum albumin OS=Bos taurus OX=9913 GN=ALB PE=1 SV=4

Observed	Mr(expt)	Mr(calc)	ppm	Start	End	Miss	Ions	Peptide
927.4397	926.4324	926.4861	-57.97	161	- 167	0	---	K.YLYEIAR.R
1479.7203	1478.7130	1478.7881	-50.80	421	- 433	0	---	K.LGEYGFQNALIVR.Y
1479.7203	1478.7131	1478.7881	-50.77	421	- 433	0	---	K.LGEYGFQNALIVR.Y
1567.6654	1566.6581	1566.7354	-49.35	347	- 359	0	---	K.DAFLGSFLYEYSR.R
1567.6654	1566.6581	1566.7354	-49.34	347	- 359	0	22	K.DAFLGSFLYEYSR.R
1724.7576	1723.7503	1723.8273	-44.65	469	- 482	0	---	R.MPCTEDYLSLILNR.L
2044.9374	2043.9301	2044.0206	-44.28	168	- 183	1	---	R.RHPYFYAPELLYYANK.Y

No match to: 805.4537, 842.4627, 848.4779, 850.0055, 850.4871, 853.4691, 864.5048, 865.9789, 868.4995, 870.5005, 876.9857, 882.5121, 882.5121, 892.9567, 919.4136, 951.5660, 982.5261, 996.5410, 1017.6141, 1017.6141, 1019.6234, 1033.6417, 1045.5149, 1051.6498, 1051.6498, 1065.5970, 1131.6476, 1133.6560, 1136.6389, 1142.5725, 1142.5725, 1147.6736, 1151.6641, 1165.6772, 1165.6773, 1175.5778, 1194.5631, 1300.7844, 1302.7943, 1316.8119, 1334.8163, 1334.8163, 1351.5889, 1414.8141, 1434.8339, 1448.8483, 1448.8483, 1457.7133, 1475.6820, 1498.6241, 1513.7238, 1513.7238, 1557.7402, 1583.9542, 1615.7332, 1617.9894, 1617.9894, 1630.7391, 1633.7523, 1647.7585, 1647.7585, 1697.8313, 1732.0159, 1901.1652, 2015.1964, 2184.3481, 2211.0227, 2283.0957, 2305.0806, 2530.2642

Search Parameters

Type of search : Sequence Query
Enzyme : Trypsin
Fixed modifications : Carbamidomethyl (C)
Variable modifications : Oxidation (M)
Mass values : Monoisotopic
Protein Mass : Unrestricted
Peptide Mass Tolerance : ± 100 ppm
Fragment Mass Tolerance: ± 0.3 Da
Max Missed Cleavages : 1
Instrument type : MALDI-TOF-TOF
Query1 (805.4537,1+): <no title>
Query2 (842.4627,1+): <no title>
Query3 (848.4779,1+): <no title>
Query4 (850.0055,1+): <no title>
Query5 (850.4871,1+): <no title>
Query6 (853.4691,1+): <no title>
Query7 (864.5048,1+): <no title>
Query8 (865.9789,1+): <no title>
Query9 (868.4995,1+): <no title>
Query10 (870.5005,1+): <no title>
Query11 (876.9857,1+): <no title>
Query12 (882.5121,1+): Locus:1..14.0.7
Query13 (882.5121,1+): <no title>
Query14 (892.9567,1+): <no title>
Query15 (919.4136,1+): <no title>
Query16 (927.4397,1+): <no title>
Query17 (951.5660,1+): <no title>
Query18 (982.5261,1+): <no title>
Query19 (996.5410,1+): <no title>
Query20 (1017.6141,1+): Locus:1..14.0.10
Query21 (1017.6141,1+): <no title>
Query22 (1019.6234,1+): <no title>
Query23 (1033.6417,1+): <no title>
Query24 (1045.5149,1+): <no title>
Query25 (1051.6498,1+): Locus:1..14.0.3
Query26 (1051.6498,1+): <no title>
Query27 (1065.5970,1+): <no title>
Query28 (1131.6476,1+): <no title>
Query29 (1133.6560,1+): <no title>
Query30 (1136.6389,1+): <no title>
Query31 (1142.5725,1+): <no title>
Query32 (1142.5725,1+): Locus:1..14.0.11
Query33 (1147.6736,1+): <no title>
Query34 (1151.6641,1+): <no title>
Query35 (1165.6772,1+): <no title>
Query36 (1165.6773,1+): Locus:1..14.0.8
Query37 (1175.5778,1+): <no title>
Query38 (1194.5631,1+): <no title>
Query39 (1300.7844,1+): <no title>
Query40 (1302.7943,1+): <no title>
Query41 (1316.8119,1+): <no title>
Query42 (1334.8163,1+): Locus:1..14.0.6
Query43 (1334.8163,1+): <no title>
Query44 (1351.5889,1+): <no title>
Query45 (1414.8141,1+): <no title>
Query46 (1434.8339,1+): <no title>
Query47 (1448.8483,1+): Locus:1..14.0.12
Query48 (1448.8483,1+): <no title>
Query49 (1457.7133,1+): <no title>
Query50 (1475.6820,1+): <no title>
Query51 (1479.7203,1+): <no title>
Query52 (1479.7203,1+): Locus:1..14.0.2
Query53 (1498.6241,1+): <no title>
Query54 (1513.7238,1+): Locus:1..14.0.1

PDF-XChange Click to buy NOW! www.docu-track.com
PDF-XChange Click to buy NOW! www.docu-track.com

Query55 (1513.7238,1+): <no title>
Query56 (1557.7402,1+): <no title>
Query57 (1567.6654,1+): <no title>
Query58 (1567.6654,1+): Locus:1..14.0.4
Query59 (1583.9542,1+): <no title>
Query60 (1615.7332,1+): <no title>
Query61 (1617.9894,1+): Locus:1..14.0.9
Query62 (1617.9894,1+): <no title>
Query63 (1630.7391,1+): <no title>
Query64 (1633.7523,1+): <no title>
Query65 (1647.7585,1+): <no title>
Query66 (1647.7585,1+): Locus:1..14.0.5
Query67 (1697.8313,1+): <no title>
Query68 (1724.7576,1+): <no title>
Query69 (1732.0159,1+): <no title>
Query70 (1901.1652,1+): <no title>
Query71 (2015.1964,1+): <no title>
Query72 (2044.9374,1+): <no title>
Query73 (2184.3481,1+): <no title>
Query74 (2211.0227,1+): <no title>
Query75 (2283.0957,1+): <no title>
Query76 (2305.0806,1+): <no title>
Query77 (2530.2642,1+): <no title>

Mascot: http://www.matrixscience.com/
