# Supplementary material for: Alpha-2-Macroglobulin as a New Promising Biomarker Improving the Diagnostic Sensitivity of Bovine Paratuberculosis
Source: Front Vet Sci. 2021 Mar 5;8:637716. doi: 10.3389/fvets.2021.637716 (PMC7973028; doi:10.3389/fvets.2021.637716)
Supplement: Supplementary Data 1 — Mascot search results and raw data files for LC-MS/MS analysis for the identification of specific blood proteins in cattle with Johne's disease. [file Data_Sheet_1.zip › mascot/7 alpha-1B-glycoprotein precursor_2.pdf]

PDF-XChange Click to buy NOW! www.docu-track.com
PDF-XChange Click to buy NOW! www.docu-track.com

MATRIX SCIENCE MASCOT Search Results

Protein View: Q2KJF1

Alpha-1B-glycoprotein OS=Bos taurus OX=9913 GN=A1BG PE=1 SV=1

Database:	Uniprot_bovine
Score:	98
Expect:	2.4e-005
Nominal mass (M_r):	54091
Calculated pI:	5.29

Sequence similarity is available as an NCBI BLAST search of Q2KJF1 against nr.

Search parameters

Enzyme:	Trypsin: cuts C-term side of KR unless next residue is P.
Fixed modifications:	Carbamidomethyl (C)
Variable modifications:	Oxidation (M)

Protein sequence coverage: 23%

Matched peptides shown in ***bold red***.

1 MSAWAALLLL WGLSLSPVTE QATFFDPRPS LWAEAGSPLA PWADVTLTCQ
51 SPLPTQEFQL LKDGVGQEPV HLESPAHEHR **FPLGPVTSTT** **R**GLYRCSYKG
101 NNDWISPSNL VEVTGAEPLP APSISTSPVS WITPGLNTTL LCLSGLR**GVT**
151 **FLLR**LEGEDQ FLEVAEAPEA TQATFPVHRA GNYSCSYRTH AAGTPSEPSA
201 TVTIEELDPP PAPTLTVDRE SAK**VLRPGSS** **ASLTCVAPLS** **GVDFQLR**RGA
251 EEQLVPRAST SPDRVFFR**LS** **ALAAGDGSGY** **TCR**YRLR**SEL** **AAWSR**DSAPA
301 ELVLSDGTLP APELSAEPAI LSPTPGALVQ LRCRAPRAGV RFALVRKDAG
351 GRQVQR**VLSP** **AGPEAQFELR** GVSAVDSGNY SCVYVDTSPP FAGSKPSATL
401 ELRVDGPLPR PQLR**ALWTGA** **LTPGR**DAVLR **CEAEVPDVSF** **LLLR**AGEEEP
451 LAVAWSTHGP ADLVLTSVGP QHAGTYSCRY RTGGPR**SLLS** **ELSDPVELR**V
501 AGS

Unformatted sequence string: 503 residues (for pasting into other applications).

Sort peptides by Residue Number Increasing Mass Decreasing Mass

Show predicted peptides also

Query	Start – End	Observed	Mr(expt)	Mr(calc)	ppm	M	Score	Peptide
37	81 – 91	1175.5778	1174.5705	1174.6346	-54.6	0		R.FPLGPVTSTTR.G
1	148 – 154	805.4537	804.4464	804.4858	-48.9	0		R.GVTFLLR.L
77	224 – 247	2530.2642	2529.2569	2529.3374	-31.8	0		K.VLRPGSSASLTCVAPLSGVDFQLR.R
53	269 – 283	1498.6241	1497.6168	1497.6882	-47.6	0		R.LSALAAGDGSGYTCR.Y
15	288 – 295	919.4136	918.4063	918.4559	-54.0	0		R.SELAAWSR.D
54	357 – 370	1513.7238	1512.7165	1512.7936	-51.0	0	27	R.VLSPAGPEAQFELR.G
55	357 – 370	1513.7238	1512.7165	1512.7936	-51.0	0		R.VLSPAGPEAQFELR.G
31	415 – 425	1142.5725	1141.5652	1141.6244	-51.8	0		R.ALWTGALTPGR.D
32	415 – 425	1142.5725	1141.5652	1141.6244	-51.8	0		R.ALWTGALTPGR.D
65	431 – 444	1647.7585	1646.7512	1646.8338	-50.1	0		R.CEAEVPDVSFLLLR.A
66	431 – 444	1647.7585	1646.7513	1646.8338	-50.1	0	27	R.CEAEVPDVSFLLLR.A
49	487 – 499	1457.7133	1456.7060	1456.7773	-48.9	0		R.SLLSELSDPVELR.V

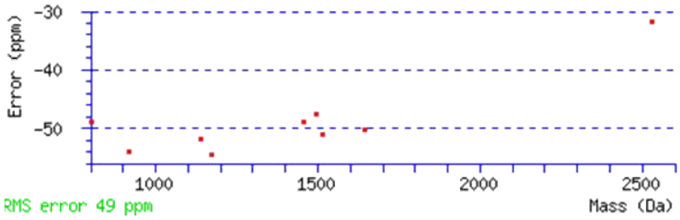

ID A1BG_BOVIN Reviewed; 503 AA.
AC Q2KJF1;

PDF-XChange Click to buy NOW! www.docu-track.com

```
DT   02-SEP-2008, integrated into UniProtKB/Swiss-Prot.
DT   07-MAR-2006, sequence version 1.
DT   12-AUG-2020, entry version 92.
DE   RecName: Full=Alpha-1B-glycoprotein {ECO:0000250|UniProtKB:P04217};
DE   AltName: Full=Alpha-1-B glycoprotein {ECO:0000250|UniProtKB:P04217, ECO:0000312|EMBL:AAI05375.1};
DE   Flags: Precursor;
GN   Name=A1BG {ECO:0000312|EMBL:AAI05375.1};
OS   Bos taurus (Bovine).
OC   Eukaryota; Metazoa; Chordata; Craniata; Vertebrata; Euteleostomi; Mammalia;
OC   Eutheria; Laurasiatheria; Artiodactyla; Ruminantia; Pecora; Bovidae;
OC   Bovinae; Bos.
OX   NCBI_TaxID=9913;
RN   [1] {ECO:0000312|EMBL:AAI05375.1}
RP   NUCLEOTIDE SEQUENCE [LARGE SCALE MRNA].
RC   STRAIN=Hereford {ECO:0000312|EMBL:AAI05375.1};
RC   TISSUE=Testis {ECO:0000312|EMBL:AAI05375.1};
RG   NIH - Mammalian Gene Collection (MGC) project;
RL   Submitted (SEP-2005) to the EMBL/GenBank/DDBJ databases.
RN   [2] {ECO:0000305}
RP   PROTEIN SEQUENCE OF 22-36, INTERACTION WITH CRISP3, AND GLYCOSYLATION.
RC   TISSUE=Serum {ECO:0000269|PubMed:20116414};
RX   PubMed=20116414; DOI=10.1016/j.bbagen.2010.01.011;
RA   Udby L., Johnsen A.H., Borregaard N.;
RT   "Human CRISP-3 binds serum alpha1B-glycoprotein across species.";
RL   Biochim. Biophys. Acta 1800:481-485(2010).
CC   -!- SUBUNIT: Interacts with CRISP3. {ECO:0000269|PubMed:20116414}.
CC   -!- SUBCELLULAR LOCATION: Secreted {ECO:0000305}.
CC   -!- TISSUE SPECIFICITY: Plasma. {ECO:0000305}.
CC   ---------------------------------------------------------------------------
CC   Copyrighted by the UniProt Consortium, see https://www.uniprot.org/terms
CC   Distributed under the Creative Commons Attribution (CC BY 4.0) License
CC   ---------------------------------------------------------------------------
DR   EMBL; BC105374; AAI05375.1; -; mRNA.
DR   RefSeq; NP_001039708.1; NM_001046243.2.
DR   STRING; 9913.ENSBTAP00000012837; -.
DR   MEROPS; I43.950; -.
DR   PaxDb; Q2KJF1; -.
DR   PeptideAtlas; Q2KJF1; -.
DR   PRIDE; Q2KJF1; -.
DR   Ensembl; ENSBTAT00000012837; ENSBTAP00000012837; ENSBTAG00000009735.
DR   GeneID; 518955; -.
DR   KEGG; bta:518955; -.
DR   CTD; 1; -.
DR   VGNC; VGNC:25433; A1BG.
DR   eggNOG; ENOG502RYEX; Eukaryota.
DR   GeneTree; ENSGT00980000198504; -.
DR   HOGENOM; CLU_042929_1_0_1; -.
DR   InParanoid; Q2KJF1; -.
DR   OMA; IKHQFLL; -.
DR   OrthoDB; 1327293at2759; -.
DR   TreeFam; TF336644; -.
DR   Reactome; R-BTA-114608; Platelet degranulation.
DR   Reactome; R-BTA-6798695; Neutrophil degranulation.
DR   Proteomes; UP000009136; Chromosome 18.
DR   Bgee; ENSBTAG00000009735; Expressed in liver and 4 other tissues.
DR   ExpressionAtlas; Q2KJF1; baseline and differential.
DR   GO; GO:0005576; C:extracellular region; IEA:UniProtKB-SubCell.
DR   Gene3D; 2.60.40.10; -; 5.
DR   InterPro; IPR016332; A1B_glyco/leuk_Ig-like_rcpt.
DR   InterPro; IPR007110; Ig-like_dom.
DR   InterPro; IPR036179; Ig-like_dom_sf.
DR   InterPro; IPR013783; Ig-like_fold.
DR   InterPro; IPR003599; Ig_sub.
DR   InterPro; IPR003598; Ig_sub2.
DR   Pfam; PF13895; Ig_2; 1.
DR   PIRSF; PIRSF001979; Alpha_1B_glycoprot_prd; 1.
DR   SMART; SM00409; IG; 4.
DR   SMART; SM00408; IGc2; 4.
DR   SUPFAM; SSF48726; SSF48726; 5.
DR   PROSITE; PS50835; IG_LIKE; 2.
PE   1: Evidence at protein level;
KW   Direct protein sequencing; Disulfide bond; Glycoprotein;
KW   Immunoglobulin domain; Reference proteome; Repeat; Secreted; Signal.
FT   SIGNAL          1..21
FT                   /evidence="ECO:0000269|PubMed:20116414"
FT   CHAIN           22..503
FT                   /note="Alpha-1B-glycoprotein"
FT                   /evidence="ECO:0000269|PubMed:20116414"
FT                   /id="PRO_0000348606"
FT   DOMAIN          27..115
FT                   /note="Ig-like V-type 1"
FT                   /evidence="ECO:0000255"
FT   DOMAIN          117..204
FT                   /note="Ig-like V-type 2"
```

PDF-XChange Click to buy NOW! www.docu-track.com

PDF-XChange Click to buy NOW! www.docu-track.com

```
FT                   /evidence="ECO:0000255"
FT   DOMAIN          208..305
FT                   /note="Ig-like V-type 3"
FT                   /evidence="ECO:0000255"
FT   DOMAIN          307..405
FT                   /note="Ig-like V-type 4"
FT                   /evidence="ECO:0000255"
FT   DOMAIN          406..501
FT                   /note="Ig-like V-type 5"
FT                   /evidence="ECO:0000255"
FT   CARBOHYD        137
FT                   /note="N-linked (GlcNAc...) asparagine"
FT                   /evidence="ECO:0000255"
FT   CARBOHYD        182
FT                   /note="N-linked (GlcNAc...) asparagine"
FT                   /evidence="ECO:0000255"
FT   CARBOHYD        379
FT                   /note="N-linked (GlcNAc...) asparagine"
FT                   /evidence="ECO:0000255"
FT   DISULFID        49..96
FT                   /evidence="ECO:0000250|UniProtKB:P04217,
FT                   ECO:0000255|PROSITE-ProRule:PRU00114"
FT   DISULFID        142..185
FT                   /evidence="ECO:0000250|UniProtKB:P04217,
FT                   ECO:0000255|PROSITE-ProRule:PRU00114"
FT   DISULFID        235..282
FT                   /evidence="ECO:0000250|UniProtKB:P04217,
FT                   ECO:0000255|PROSITE-ProRule:PRU00114"
FT   DISULFID        333..382
FT                   /evidence="ECO:0000250|UniProtKB:P04217,
FT                   ECO:0000255|PROSITE-ProRule:PRU00114"
FT   DISULFID        431..478
FT                   /evidence="ECO:0000250|UniProtKB:P04217,
FT                   ECO:0000255|PROSITE-ProRule:PRU00114"
SQ   SEQUENCE   503 AA;  53554 MW;  22643DBA75DD4435 CRC64;
     MSAWAALLLL WGLSLSPVTE QATFFDPRPS LWAEAGSPLA PWADVTLTCQ SPLPTQEFQL
     LKDGVGQEPV HLESPAHEHR FPLGPVTSTT RGLYRCSYKG NNDWISPSNL VEVTGAEPLP
     APSISTSPVS WITPGLNTTL LCLSGLRGVT FLLRLEGEDQ FLEVAEAPEA TQATFPVHRA
     GNYSCSYRTH AAGTPSEPSA TVTIEELDPP PAPTLTVDRE SAKVLRPGSS ASLTCVAPLS
     GVDFQLRRGA EEQLVPRAST SPDRVFFRLS ALAAGDGSGY TCRYRLRSEL AAWSRDSAPA
     ELVLSDGTLP APELSAEPAI LSPTPGALVQ LRCRAPRAGV RFALVRKDAG GRQVQRVLSP
     AGPEAQFELR GVSAVDSGNY SCVYVDTSPP FAGSKPSATL ELRVDGPLPR PQLRALWTGA
     LTPGRDAVLR CEAEVPDVSF LLLRAGEEEP LAVAWSTHGP ADLVLTSVGP QHAGTYSCRY
     RTGGPRSLLS ELSDPVELRV AGS
//
```

Mascot: http://www.matrixscience.com/
