# Supplementary material for: Alpha-2-Macroglobulin as a New Promising Biomarker Improving the Diagnostic Sensitivity of Bovine Paratuberculosis
Source: Front Vet Sci. 2021 Mar 5;8:637716. doi: 10.3389/fvets.2021.637716 (PMC7973028; doi:10.3389/fvets.2021.637716)
Supplement: Supplementary Data 1 — Mascot search results and raw data files for LC-MS/MS analysis for the identification of specific blood proteins in cattle with Johne's disease. [file Data_Sheet_1.zip › mascot/8 Kallikrein G_1.pdf]

PDF-XChange Click to buy NOW! www.docu-track.com

PDF-XChange Click to buy NOW! www.docu-track.com

MATRIX SCIENCE Mascot Search Results

User	:
Email	:
Search title	: BAEDW\2020_3_5\2020_3_5\MSMS 14\N22
Database	: Uniprot_bovine bovine_20200316 (134392 sequences; 76244912 residues)
Timestamp	: 31 Mar 2020 at 01:09:04 GMT
Warning	: A Peptide summary report will usually give a much clearer picture of MS/MS search results.
Top Score	: 66 for A0A1R3UGP4, Kallikrein G OS=Bos taurus OX=9913 GN=KLNG PE=3 SV=1

Mascot Score Histogram

Protein score is -10*Log(P), where P is the probability that the observed match is a random event.
Protein scores greater than 64 are significant (p<0.05).
Protein scores are derived from ions scores as a non-probabilistic basis for ranking protein hits.

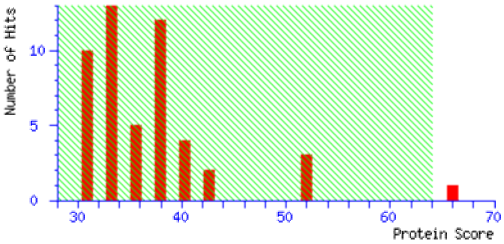

Protein Summary Report

Format As | Protein Summary (deprecated) | Help

Significance threshold p< 0.05 | Max. number of hits 30

Preferred taxonomy | All entries

Re-Search All | Search Unmatched

Index

	Accession	Mass	Score	Description
1.	A0A1R3UGP4	28249	66	Kallikrein G OS=Bos taurus OX=9913 GN=KLNG PE=3 SV=1
2.	F1N376	69563	53	Tyrosine-protein phosphatase non-receptor type OS=Bos taurus OX=9913 GN=LOC526769 PE=3 SV=3
3.	A0A4W2FSQ5	66334	52	Uncharacterized protein OS=Bos indicus x Bos taurus OX=30522 GN=LOC113906723 PE=4 SV=1
4.	A0A4W2CKS5	66352	52	Uncharacterized protein OS=Bos indicus x Bos taurus OX=30522 PE=4 SV=1
5.	A0A4W2F3Q5	49802	43	Dihydrolipoamide dehydrogenase OS=Bos indicus x Bos taurus OX=30522 GN=DLD PE=3 SV=1
6.	A0A4W2F4W9	52206	42	Dihydrolipoyl dehydrogenase OS=Bos indicus x Bos taurus OX=30522 GN=DLD PE=3 SV=1
7.	A0A4W2G1C7	31863	41	Kallikrein related peptidase 11 OS=Bos indicus x Bos taurus OX=30522 GN=KLK11 PE=3 SV=1
8.	A0A4W2G501	23018	40	TMEM9 domain family member B OS=Bos indicus x Bos taurus OX=30522 GN=TMEM9B PE=4 SV=1
9.	Q3ZCD6	23018	40	TMEM9 domain family member B OS=Bos taurus OX=9913 GN=TMEM9B PE=2 SV=1
10.	A0A4W2DBF5	25092	39	Uncharacterized protein OS=Bos indicus x Bos taurus OX=30522 PE=4 SV=1
11.	F1N206	54723	39	Dihydrolipoyl dehydrogenase OS=Bos taurus OX=9913 GN=DLD PE=1 SV=2
12.	A0A4W2H686	54723	39	Dihydrolipoyl dehydrogenase OS=Bos indicus x Bos taurus OX=30522 GN=DLD PE=3 SV=1
13.	A0A4W2H5Z2	33370	38	Ketohexokinase OS=Bos indicus x Bos taurus OX=30522 GN=KHK PE=4 SV=1
14.	A0A3Q1N4Y2	25122	38	Uncharacterized protein OS=Bos taurus OX=9913 PE=4 SV=1
15.	F6PSE2	92262	38	Nibrin OS=Bos taurus OX=9913 GN=NBN PE=4 SV=2
16.	A0A4W2CAE8	92262	38	Nibrin OS=Bos indicus x Bos taurus OX=30522 GN=NBN PE=4 SV=1
17.	A0A4W2BNU6	41726	38	Dehydrogenase/reductase 12 OS=Bos indicus x Bos taurus OX=30522 GN=DHRS12 PE=4 SV=1
18.	A0A3Q1M3R6	41726	38	Dehydrogenase/reductase SDR family member 12 OS=Bos taurus OX=9913 GN=DHRS12 PE=4 SV=1
19.	A0A4W2E9U7	76824	38	Nibrin OS=Bos indicus x Bos taurus OX=30522 GN=NBN PE=4 SV=1
20.	A0A4W2E4S5	35275	38	Kallikrein related peptidase 11 OS=Bos indicus x Bos taurus OX=30522 GN=KLK11 PE=3 SV=1
21.	Q5EA35	33326	37	Ketohexokinase OS=Bos taurus OX=9913 GN=KHK PE=2 SV=1
22.	Q0II75	38414	37	Ketohexokinase OS=Bos taurus OX=9913 GN=KHK PE=1 SV=1
23.	A0A4W2EMU6	31365	36	Ketohexokinase OS=Bos indicus x Bos taurus OX=30522 GN=KHK PE=4 SV=1
24.	A0A4W2G3Z2	37905	36	Kallikrein related peptidase 11 OS=Bos indicus x Bos taurus OX=30522 GN=KLK11 PE=3 SV=1
25.	F1N4M1	37905	36	Kallikrein related peptidase 11 OS=Bos taurus OX=9913 GN=KLK11 PE=3 SV=3
26.	A0A4W2H4X6	31395	35	Ketohexokinase OS=Bos indicus x Bos taurus OX=30522 GN=KHK PE=4 SV=1
27.	A0A4W2C9V0	38733	35	Uncharacterized protein OS=Bos indicus x Bos taurus OX=30522 GN=MFGE8 PE=4 SV=1
28.	A0A4W2F3R1	41965	34	Reverse transcriptase domain-containing protein OS=Bos indicus x Bos taurus OX=30522 PE=4 SV=1
29.	A0A4W2IP26	92361	34	Nibrin OS=Bos indicus x Bos taurus OX=30522 GN=NBN PE=4 SV=1
30.	A0A1C9HI59	67468	34	LS037 OS=Lumpy skin disease virus OX=59509 GN=LW037 PE=4 SV=1

Results List

1. A0A1R3UGP4 **Mass:** 28249 **Score:** 66 **Expect:** 0.034 **Matches:** 10

Kallikrein G OS=Bos taurus OX=9913 GN=KLNG PE=3 SV=1

Observed	Mr(expt)	Mr(calc)	ppm	Start	End	Miss	Ions	Peptide
848.4780	847.4707	847.4473	27.6	109 -	115	0	---	R.NDIMLVK.M + Oxidation (M)
951.5720	950.5647	950.4862	82.7	239 -	245	0	---	K.YVDWIQK.T
1179.5499	1178.5426	1178.6482	-89.54	229 -	238	1	---	K.KPGVYTKVCK.Y
1184.6520	1183.6447	1183.6747	-25.32	45 -	55	0	---	R.LLCGATLIAPR.W
1211.6572	1210.6499	1210.6604	-8.69	163 -	172	1	---	R.CANVTIIKHR.E
2015.1949	2014.1876	2013.9908	97.7	88 -	105	1	---	R.TATKSFPHPDFNNSLPNK.D
2283.0847	2282.0774	2282.0975	-8.78	68 -	87	1	---	R.YVVHLGAHSLGRQDGCEQTR.T
2305.0740	2304.0667	2304.1793	-48.84	1 -	21	0	---	-.MMTLQLIMFALVTGHVGGETR.I
2321.0552	2320.0479	2320.1742	-54.42	1 -	21	0	---	-.MMTLQLIMFALVTGHVGGETR.I + Oxidation (M)
2528.2617	2527.2544	2527.4019	-58.33	2 -	24	1	---	M.MTLQLIMFALVTGHVGGETRIIK.G

PDF-XChange Click to buy NOW! www.docu-track.com

PDF-XChange Click to buy NOW! www.docu-track.com

No match to: 813.4393, 834.4629, 838.4906, 842.4641, 846.4475, 850.0118, 850.4896, 850.4896, 853.4691, 856.4822, 864.4857, 865.9818, 866.4929, 868.5015, 868.5015, 870.4977, 876.9884, 882.5173, 882.5173, 892.9574, 892.9574, 896.3969, 908.9280, 996.5430, 1017.6157, 1017.6157, 1019.6299, 1033.6429, 1045.5127, 1045.5127, 1051.6522, 1051.6522, 1065.6028, 1131.6493, 1133.6587, 1147.6777, 1151.6696, 1165.6809, 1165.6809, 1169.5313, 1171.6676, 1185.6776, 1185.6776, 1189.6735, 1193.6417, 1203.6858, 1207.6569, 1225.6708, 1279.7007, 1300.7860, 1316.8134, 1334.8168, 1334.8168, 1440.5756, 1448.8479, 1448.8479, 1475.6846, 1522.7052, 1533.7407, 1538.7010, 1554.6993, 1581.7728, 1617.9867, 1617.9867, 1732.0165, 1901.1586, 2211.0193

2. F1N376 **Mass:** 69563 **Score:** 53 **Expect:** 0.74 **Matches:** 12

Tyrosine-protein phosphatase non-receptor type OS=Bos taurus OX=9913 GN=LOC526769 PE=3 SV=3

Observed	Mr(expt)	Mr(calc)	ppm	Start	End	Miss	Ions	Peptide
813.4393	812.4320	812.4868	-67.44	214	220	1	---	R.QPLKATR.I
838.4906	837.4833	837.4821	1.48	538	546	0	---	R.KPVGAGPGR.G
846.4475	845.4402	845.3991	48.6	565	570	1	---	R.ERQEER.G
866.4929	865.4856	865.5134	-32.09	206	213	0	---	R.SGVVVHLR.Q
1133.6587	1132.6514	1132.6101	36.5	47	55	1	---	R.RHNEVTHIK.I
1179.5499	1178.5426	1178.5536	-9.34	547	558	0	---	R.GGACAGVTMVTR.G
1522.7052	1521.6979	1521.7762	-51.46	166	178	0	---	R.VTHIMIHFQPDGK.Y
1538.7010	1537.6937	1537.7711	-50.35	166	178	0	---	R.VTHIMIHFQPDGK.Y + Oxidation (M)
2015.1949	2014.1876	2014.0201	83.2	538	558	1	---	R.KPVGAGPGRGGACAGVTMVTR.G + Oxidation (M)
2211.0193	2210.0120	2210.1596	-66.79	71	90	0	---	K.FATLAELVQHYTGQHGGLLR.E
2321.0552	2320.0479	2320.1394	-39.41	221	242	1	---	R.ISAASIESRVQELSEATDASEK.A
2528.2617	2527.2544	2527.2819	-10.88	290	311	1	---	R.VILHDVDDRVPGADYINANYIR.Q

No match to: 834.4629, 842.4641, 848.4780, 850.0118, 850.4896, 850.4896, 853.4691, 856.4822, 864.4857, 865.9818, 868.5015, 868.5015, 870.4977, 876.9884, 882.5173, 882.5173, 892.9574, 892.9574, 896.3969, 908.9280, 951.5720, 996.5430, 1017.6157, 1017.6157, 1019.6299, 1033.6429, 1045.5127, 1045.5127, 1051.6522, 1051.6522, 1065.6028, 1131.6493, 1147.6777, 1151.6696, 1165.6809, 1165.6809, 1169.5313, 1171.6676, 1184.6520, 1185.6776, 1185.6776, 1189.6735, 1193.6417, 1203.6858, 1207.6569, 1211.6572, 1225.6708, 1279.7007, 1300.7860, 1316.8134, 1334.8168, 1334.8168, 1440.5756, 1448.8479, 1448.8479, 1475.6846, 1533.7407, 1554.6993, 1581.7728, 1617.9867, 1617.9867, 1732.0165, 1901.1586, 2283.0847, 2305.0740

3. A0A4W2FSQ5 **Mass:** 66334 **Score:** 52 **Expect:** 0.76 **Matches:** 12

Uncharacterized protein OS=Bos indicus x Bos taurus OX=30522 GN=LOC113906723 PE=4 SV=1

Observed	Mr(expt)	Mr(calc)	ppm	Start	End	Miss	Ions	Peptide
813.4393	812.4320	812.4868	-67.44	194	200	1	---	R.QPLKATR.I
838.4906	837.4833	837.4821	1.48	510	518	0	---	R.KPVGAGPGR.G
846.4475	845.4402	845.3991	48.6	537	542	1	---	R.ERQEER.G
866.4929	865.4856	865.5134	-32.09	186	193	0	---	R.SGVVVHLR.Q
1133.6587	1132.6514	1132.6101	36.5	27	35	1	---	R.RHNEVTHIK.I
1179.5499	1178.5426	1178.5536	-9.34	519	530	0	---	R.GGACAGVTMVTR.G
1522.7052	1521.6979	1521.7762	-51.46	146	158	0	---	R.VTHIMIHFQPDGK.Y
1538.7010	1537.6937	1537.7711	-50.35	146	158	0	---	R.VTHIMIHFQPDGK.Y + Oxidation (M)
2015.1949	2014.1876	2014.0201	83.2	510	530	1	---	R.KPVGAGPGRGGACAGVTMVTR.G + Oxidation (M)
2211.0193	2210.0120	2210.1596	-66.79	51	70	0	---	K.FATLAELVQHYTGQHGGLLR.E
2321.0552	2320.0479	2320.1394	-39.41	201	222	1	---	R.ISAASIESRVQELSEATDASEK.A
2528.2617	2527.2544	2527.2819	-10.88	270	291	1	---	R.VILHDVDDRVPGADYINANYIR.S

No match to: 834.4629, 842.4641, 848.4780, 850.0118, 850.4896, 850.4896, 853.4691, 856.4822, 864.4857, 865.9818, 868.5015, 868.5015, 870.4977, 876.9884, 882.5173, 882.5173, 892.9574, 892.9574, 896.3969, 908.9280, 951.5720, 996.5430, 1017.6157, 1017.6157, 1019.6299, 1033.6429, 1045.5127, 1045.5127, 1051.6522, 1051.6522, 1065.6028, 1131.6493, 1147.6777, 1151.6696, 1165.6809, 1165.6809, 1169.5313, 1171.6676, 1184.6520, 1185.6776, 1185.6776, 1189.6735, 1193.6417, 1203.6858, 1207.6569, 1211.6572, 1225.6708, 1279.7007, 1300.7860, 1316.8134, 1334.8168, 1334.8168, 1440.5756, 1448.8479, 1448.8479, 1475.6846, 1533.7407, 1554.6993, 1581.7728, 1617.9867, 1617.9867, 1732.0165, 1901.1586, 2283.0847, 2305.0740

4. A0A4W2CKS5 **Mass:** 66352 **Score:** 52 **Expect:** 0.89 **Matches:** 12

Uncharacterized protein OS=Bos indicus x Bos taurus OX=30522 PE=4 SV=1

Observed	Mr(expt)	Mr(calc)	ppm	Start	End	Miss	Ions	Peptide
813.4393	812.4320	812.4868	-67.44	194	200	1	---	R.QPLKATR.I
838.4906	837.4833	837.4821	1.48	510	518	0	---	R.KPVGAGPGR.G
846.4475	845.4402	845.3991	48.6	537	542	1	---	R.ERQEER.G
866.4929	865.4856	865.5134	-32.09	186	193	0	---	R.SGVVVHLR.Q
1133.6587	1132.6514	1132.6101	36.5	27	35	1	---	R.RHNEVTHIK.I
1179.5499	1178.5426	1178.5536	-9.34	519	530	0	---	R.GGACAGVTMVTR.G
1522.7052	1521.6979	1521.7762	-51.46	146	158	0	---	R.VTHIMIHFQPDGK.Y
1538.7010	1537.6937	1537.7711	-50.35	146	158	0	---	R.VTHIMIHFQPDGK.Y + Oxidation (M)
2015.1949	2014.1876	2014.0201	83.2	510	530	1	---	R.KPVGAGPGRGGACAGVTMVTR.G + Oxidation (M)
2211.0193	2210.0120	2210.1596	-66.79	51	70	0	---	K.FATLAELVQHYTGQHGGLLR.E
2321.0552	2320.0479	2320.1394	-39.41	201	222	1	---	R.ISAASIESRVQELSEATDASEK.A
2528.2617	2527.2544	2527.2819	-10.88	270	291	1	---	R.VILHDVDDRVPGADYINANYIR.S

No match to: 834.4629, 842.4641, 848.4780, 850.0118, 850.4896, 850.4896, 853.4691, 856.4822, 864.4857, 865.9818, 868.5015, 868.5015, 870.4977, 876.9884, 882.5173, 882.5173, 892.9574, 892.9574, 896.3969, 908.9280, 951.5720, 996.5430, 1017.6157, 1017.6157, 1019.6299, 1033.6429, 1045.5127, 1045.5127, 1051.6522, 1051.6522, 1065.6028, 1131.6493, 1147.6777, 1151.6696, 1165.6809, 1165.6809, 1169.5313, 1171.6676, 1184.6520, 1185.6776, 1185.6776, 1189.6735, 1193.6417, 1203.6858, 1207.6569, 1211.6572, 1225.6708, 1279.7007, 1300.7860, 1316.8134, 1334.8168, 1334.8168, 1440.5756, 1448.8479, 1448.8479, 1475.6846, 1533.7407, 1554.6993, 1581.7728, 1617.9867, 1617.9867, 1732.0165, 1901.1586, 2283.0847, 2305.0740

5. A0A4W2F3Q5 **Mass:** 49802 **Score:** 43 **Expect:** 6.3 **Matches:** 12

Dihydrolipoamide dehydrogenase OS=Bos indicus x Bos taurus OX=30522 GN=DLD PE=3 SV=1

Observed	Mr(expt)	Mr(calc)	ppm	Start	End	Miss	Ions	Peptide
868.5015	867.4942	867.4524	48.2	7	13	0	---	R.VYCSLVK.R
868.5015	867.4943	867.4524	48.2	7	13	0	---	R.VYCSLVK.R
1017.6157	1016.6084	1016.5614	46.2	393	401	1	---	K.ILGQKSTDR.V
1017.6157	1016.6084	1016.5614	46.2	393	401	1	---	K.ILGQKSTDR.V
1151.6696	1150.6623	1150.5870	65.5	363	372	1	---	K.EEGIEYKVGK.F
1165.6809	1164.6736	1164.5631	94.9	123	132	1	---	K.MMEQKSNAVK.A
1165.6809	1164.6736	1164.5631	94.9	123	132	1	---	K.MMEQKSNAVK.A
1193.6417	1192.6344	1192.6353	-0.72	370	380	1	---	K.VGKFPFAANSR.A
1203.6858	1202.6785	1202.6772	1.14	289	298	1	---	R.IPVNTRFQTK.I
1279.7007	1278.6934	1278.6311	48.7	118	127	1	---	R.LNLEKMMEQK.S + Oxidation (M)

PDF-XChange Click to buy NOW! www.docu-track.com

PDF-XChange Click to buy NOW! www.docu-track.com

1533.7407	1532.7334	1532.7682	-22.69	237	252	1	---	K.SDGKIDVSIEAASGGK.A
1581.7728	1580.7655	1580.7569	5.44	357	369	1	---	K.SEEQLKEEGIEYK.V

No match to: 813.4393, 834.4629, 838.4906, 842.4641, 846.4475, 848.4780, 850.0118, 850.4896, 850.4896, 853.4691, 856.4822, 864.4857, 865.9818, 866.4929, 870.4977, 876.9884, 882.5173, 882.5173, 892.9574, 892.9574, 896.3969, 908.9280, 951.5720, 996.5430, 1019.6299, 1033.6429, 1045.5127, 1045.5127, 1051.6522, 1051.6522, 1065.6028, 1131.6493, 1133.6587, 1147.6777, 1169.5313, 1171.6676, 1179.5499, 1184.6520, 1185.6776, 1185.6776, 1189.6735, 1207.6569, 1211.6572, 1225.6708, 1300.7860, 1316.8134, 1334.8168, 1334.8168, 1440.5756, 1448.8479, 1448.8479, 1475.6846, 1522.7052, 1538.7010, 1554.6993, 1617.9867, 1617.9867, 1732.0165, 1901.1586, 2015.1949, 2211.0193, 2283.0847, 2305.0740, 2321.0552, 2528.2617

6. A0A4W2F4W9 **Mass:** 52206 **Score:** 42 **Expect:** 9.3 **Matches:** 12

Dihydrolipoyl dehydrogenase OS=Bos indicus x Bos taurus OX=30522 GN=DLD PE=3 SV=1

Observed	Mr(expt)	Mr(calc)	ppm	Start	End	Miss	Ions	Peptide
868.5015	867.4942	867.4524	48.2	7	13	0	---	R.VYCSLVK.R
868.5015	867.4943	867.4524	48.2	7	13	0	---	R.VYCSLVK.R
1017.6157	1016.6084	1016.5614	46.2	418	426	1	---	K.ILGQKSTDR.V
1017.6157	1016.6084	1016.5614	46.2	418	426	1	---	K.ILGQKSTDR.V
1151.6696	1150.6623	1150.5870	65.5	388	397	1	---	K.EEGIEYKVGK.F
1165.6809	1164.6736	1164.5631	94.9	100	109	1	---	K.MMEQKSNAVK.A
1165.6809	1164.6736	1164.5631	94.9	100	109	1	---	K.MMEQKSNAVK.A
1193.6417	1192.6344	1192.6353	-0.72	395	405	1	---	K.VGKFPFAANSR.A
1203.6858	1202.6785	1202.6772	1.14	314	323	1	---	R.IPVNTRFQTK.I
1279.7007	1278.6934	1278.6311	48.7	95	104	1	---	R.LNLEKMMEQK.S + Oxidation (M)
1533.7407	1532.7334	1532.7682	-22.69	262	277	1	---	K.SDGKIDVSIEAASGGK.A
1581.7728	1580.7655	1580.7569	5.44	382	394	1	---	K.SEEQLKEEGIEYK.V

No match to: 813.4393, 834.4629, 838.4906, 842.4641, 846.4475, 848.4780, 850.0118, 850.4896, 850.4896, 853.4691, 856.4822, 864.4857, 865.9818, 866.4929, 870.4977, 876.9884, 882.5173, 882.5173, 892.9574, 892.9574, 896.3969, 908.9280, 951.5720, 996.5430, 1019.6299, 1033.6429, 1045.5127, 1045.5127, 1051.6522, 1051.6522, 1065.6028, 1131.6493, 1133.6587, 1147.6777, 1169.5313, 1171.6676, 1179.5499, 1184.6520, 1185.6776, 1185.6776, 1189.6735, 1207.6569, 1211.6572, 1225.6708, 1300.7860, 1316.8134, 1334.8168, 1334.8168, 1440.5756, 1448.8479, 1448.8479, 1475.6846, 1522.7052, 1538.7010, 1554.6993, 1617.9867, 1617.9867, 1732.0165, 1901.1586, 2015.1949, 2211.0193, 2283.0847, 2305.0740, 2321.0552, 2528.2617

7. A0A4W2G1C7 **Mass:** 31863 **Score:** 41 **Expect:** 11 **Matches:** 7

Kallikrein related peptidase 11 OS=Bos indicus x Bos taurus OX=30522 GN=KLK11 PE=3 SV=1

Observed	Mr(expt)	Mr(calc)	ppm	Start	End	Miss	Ions	Peptide
848.4780	847.4707	847.4473	27.6	140	146	0	---	R.NDIMLVK.M + Oxidation (M)
951.5720	950.5647	950.4862	82.7	270	276	0	---	K.YVDWIQK.T
1179.5499	1178.5426	1178.6482	-89.54	260	269	1	---	K.KPGVYTKVCK.Y
1184.6520	1183.6447	1183.6747	-25.32	76	86	0	---	R.LLCGATLIAPR.W
1211.6572	1210.6499	1210.6604	-8.69	194	203	1	---	R.CANVTIIKHR.E
2015.1949	2014.1876	2013.9908	97.7	119	136	1	---	R.TATKSFPHPDFNNSLPNK.D
2283.0847	2282.0774	2282.0975	-8.78	99	118	1	---	R.YVVHLGAHSLGRQDGCEQTR.T

No match to: 813.4393, 834.4629, 838.4906, 842.4641, 846.4475, 850.0118, 850.4896, 850.4896, 853.4691, 856.4822, 864.4857, 865.9818, 866.4929, 868.5015, 868.5015, 870.4977, 876.9884, 882.5173, 882.5173, 892.9574, 892.9574, 896.3969, 908.9280, 996.5430, 1017.6157, 1017.6157, 1019.6299, 1033.6429, 1045.5127, 1045.5127, 1051.6522, 1051.6522, 1065.6028, 1131.6493, 1133.6587, 1147.6777, 1151.6696, 1165.6809, 1165.6809, 1169.5313, 1171.6676, 1185.6776, 1185.6776, 1189.6735, 1193.6417, 1203.6858, 1207.6569, 1225.6708, 1279.7007, 1300.7860, 1316.8134, 1334.8168, 1334.8168, 1440.5756, 1448.8479, 1448.8479, 1475.6846, 1522.7052, 1533.7407, 1538.7010, 1554.6993, 1581.7728, 1617.9867, 1617.9867, 1732.0165, 1901.1586, 2211.0193, 2305.0740, 2321.0552, 2528.2617

8. A0A4W2G501 **Mass:** 23018 **Score:** 40 **Expect:** 15 **Matches:** 7

TMEM9 domain family member B OS=Bos indicus x Bos taurus OX=30522 GN=TMEM9B PE=4 SV=1

Observed	Mr(expt)	Mr(calc)	ppm	Start	End	Miss	Ions	Peptide
1131.6493	1130.6420	1130.6270	13.3	1	10	0	---	-.MATLWAGLLR.L
1147.6777	1146.6704	1146.6219	42.3	1	10	0	---	-.MATLWAGLLR.L + Oxidation (M)
1179.5499	1178.5426	1178.5390	3.07	79	88	0	---	R.GPDVEAYCLR.C
1207.6569	1206.6496	1206.6145	29.1	172	180	1	---	K.VEYAQQRWK.L
1211.6572	1210.6499	1210.6193	25.3	93	102	1	---	K.YEERSSVTIK.V
1225.6708	1224.6635	1224.5453	96.5	42	50	1	---	R.CKCICPPYK.D
2283.0847	2282.0774	2282.0606	7.37	60	78	1	---	K.NISQKDCDCLHVVDPMPVR.G

No match to: 813.4393, 834.4629, 838.4906, 842.4641, 846.4475, 848.4780, 850.0118, 850.4896, 850.4896, 853.4691, 856.4822, 864.4857, 865.9818, 866.4929, 868.5015, 868.5015, 870.4977, 876.9884, 882.5173, 882.5173, 892.9574, 892.9574, 896.3969, 908.9280, 951.5720, 996.5430, 1017.6157, 1017.6157, 1019.6299, 1033.6429, 1045.5127, 1045.5127, 1051.6522, 1051.6522, 1065.6028, 1133.6587, 1151.6696, 1165.6809, 1165.6809, 1169.5313, 1171.6676, 1184.6520, 1185.6776, 1185.6776, 1189.6735, 1193.6417, 1203.6858, 1279.7007, 1300.7860, 1316.8134, 1334.8168, 1334.8168, 1440.5756, 1448.8479, 1448.8479, 1475.6846, 1522.7052, 1533.7407, 1538.7010, 1554.6993, 1581.7728, 1617.9867, 1617.9867, 1732.0165, 1901.1586, 2015.1949, 2211.0193, 2305.0740, 2321.0552, 2528.2617

9. Q3ZCD6 **Mass:** 23018 **Score:** 40 **Expect:** 15 **Matches:** 7

TMEM9 domain family member B OS=Bos taurus OX=9913 GN=TMEM9B PE=2 SV=1

Observed	Mr(expt)	Mr(calc)	ppm	Start	End	Miss	Ions	Peptide
1131.6493	1130.6420	1130.6270	13.3	1	10	0	---	-.MATLWAGLLR.L
1147.6777	1146.6704	1146.6219	42.3	1	10	0	---	-.MATLWAGLLR.L + Oxidation (M)
1179.5499	1178.5426	1178.5390	3.07	79	88	0	---	R.GPDVEAYCLR.C
1207.6569	1206.6496	1206.6145	29.1	172	180	1	---	K.VEYAQQRWK.L
1211.6572	1210.6499	1210.6193	25.3	93	102	1	---	K.YEERSSVTIK.V
1225.6708	1224.6635	1224.5453	96.5	42	50	1	---	R.CKCICPPYK.D
2283.0847	2282.0774	2282.0606	7.37	60	78	1	---	K.NISQKDCDCLHVVDPMPVR.G

No match to: 813.4393, 834.4629, 838.4906, 842.4641, 846.4475, 848.4780, 850.0118, 850.4896, 850.4896, 853.4691, 856.4822, 864.4857, 865.9818, 866.4929, 868.5015, 868.5015, 870.4977, 876.9884, 882.5173, 882.5173, 892.9574, 892.9574, 896.3969, 908.9280, 951.5720, 996.5430, 1017.6157, 1017.6157, 1019.6299, 1033.6429, 1045.5127, 1045.5127, 1051.6522, 1051.6522, 1065.6028, 1133.6587, 1151.6696, 1165.6809, 1165.6809, 1169.5313, 1171.6676, 1184.6520, 1185.6776, 1185.6776, 1189.6735, 1193.6417, 1203.6858, 1279.7007, 1300.7860, 1316.8134, 1334.8168, 1334.8168, 1440.5756, 1448.8479, 1448.8479, 1475.6846, 1522.7052, 1533.7407, 1538.7010, 1554.6993, 1581.7728, 1617.9867, 1617.9867, 1732.0165, 1901.1586, 2015.1949, 2211.0193, 2305.0740, 2321.0552, 2528.2617

10. A0A4W2DBF5 **Mass:** 25092 **Score:** 39 **Expect:** 16 **Matches:** 13

Uncharacterized protein OS=Bos indicus x Bos taurus OX=30522 PE=4 SV=1

Observed	Mr(expt)	Mr(calc)	ppm	Start	End	Miss	Ions	Peptide
838.4906	837.4833	837.5072	-28.53	49	55	0	---	K.DPKPIIR.D

PDF-XChange Click to buy NOW! www.docu-track.com

866.4929	865.4856	865.4215	74.1	175 -	181	1	---	R.ETTMKEK.S
882.5173	881.5100	881.4454	73.3	5 -	13	1	---	R.STSSTKSGK.F
882.5173	881.5100	881.4454	73.3	5 -	13	1	---	R.STSSTKSGK.F
1165.6809	1164.6736	1164.6026	61.0	98 -	106	1	---	R.KLEVEYEQK.R
1165.6809	1164.6736	1164.6026	61.0	98 -	106	1	---	R.KLEVEYEQK.R
1193.6417	1192.6344	1192.6088	21.5	99 -	107	1	---	K.LEVEYEQKR.A
1207.6569	1206.6496	1206.5815	56.4	14 -	23	1	---	K.FMNPTDQARK.E
1522.7052	1521.6979	1521.7830	-55.88	162 -	174	1	---	K.VKGMNLCTVMTLR.E
1538.7010	1537.6937	1537.7779	-54.72	162 -	174	1	---	K.VKGMNLCTVMTLR.E + Oxidation (M)
1554.6993	1553.6920	1553.7728	-51.99	162 -	174	1	---	K.VKGMNLCTVMTLR.E + 2 Oxidation (M)
2211.0193	2210.0120	2210.1538	-64.16	144 -	163	1	---	R.LSMTTVLMTATVTDQVEKVK.G + Oxidation (M)
2528.2617	2527.2544	2527.2662	-4.67	140 -	161	1	---	K.TWTRLSMTTVLMTATVTDQVEK.V + Oxidation (M)

No match to: 813.4393, 834.4629, 842.4641, 846.4475, 848.4780, 850.0118, 850.4896, 850.4896, 853.4691, 856.4822, 864.4857, 865.9818, 868.5015, 868.5015, 870.4977, 876.9884, 892.9574, 892.9574, 896.3969, 908.9280, 951.5720, 996.5430, 1017.6157, 1017.6157, 1019.6299, 1033.6429, 1045.5127, 1045.5127, 1051.6522, 1051.6522, 1065.6028, 1131.6493, 1133.6587, 1147.6777, 1151.6696, 1169.5313, 1171.6676, 1179.5499, 1184.6520, 1185.6776, 1185.6776, 1189.6735, 1203.6858, 1211.6572, 1225.6708, 1279.7007, 1300.7860, 1316.8134, 1334.8168, 1334.8168, 1440.5756, 1448.8479, 1448.8479, 1475.6846, 1533.7407, 1581.7728, 1617.9867, 1617.9867, 1732.0165, 1901.1586, 2015.1949, 2283.0847, 2305.0740, 2321.0552

11. F1N206 **Mass:** 54723 **Score:** 39 **Expect:** 19 **Matches:** 12

Dihydrolipoyl dehydrogenase OS=Bos taurus OX=9913 GN=DLD PE=1 SV=2

Observed	Mr(expt)	Mr(calc)	ppm	Start	End	Miss	Ions	Peptide
868.5015	867.4942	867.4524	48.2	7 -	13	0	---	R.VYCSLVK.R
868.5015	867.4943	867.4524	48.2	7 -	13	0	---	R.VYCSLVK.R
1017.6157	1016.6084	1016.5614	46.2	441 -	449	1	---	K.ILGQKSTDR.V
1017.6157	1016.6084	1016.5614	46.2	441 -	449	1	---	K.ILGQKSTDR.V
1151.6696	1150.6623	1150.5870	65.5	411 -	420	1	---	K.EEGIEYKVGK.F
1165.6809	1164.6736	1164.5631	94.9	123 -	132	1	---	K.MMEQKSNAVK.A
1165.6809	1164.6736	1164.5631	94.9	123 -	132	1	---	K.MMEQKSNAVK.A
1193.6417	1192.6344	1192.6353	-0.72	418 -	428	1	---	K.VGKFPFAANSR.A
1203.6858	1202.6785	1202.6772	1.14	337 -	346	1	---	R.IPVNTRFQTK.I
1279.7007	1278.6934	1278.6311	48.7	118 -	127	1	---	R.LNLEKMMEQK.S + Oxidation (M)
1533.7407	1532.7334	1532.7682	-22.69	285 -	300	1	---	K.SDGKIDVSIEAASGGK.A
1581.7728	1580.7655	1580.7569	5.44	405 -	417	1	---	K.SEEQLKEEGIEYK.V

No match to: 813.4393, 834.4629, 838.4906, 842.4641, 846.4475, 848.4780, 850.0118, 850.4896, 850.4896, 853.4691, 856.4822, 864.4857, 865.9818, 866.4929, 870.4977, 876.9884, 882.5173, 882.5173, 892.9574, 892.9574, 896.3969, 908.9280, 951.5720, 996.5430, 1019.6299, 1033.6429, 1045.5127, 1045.5127, 1051.6522, 1051.6522, 1065.6028, 1131.6493, 1133.6587, 1147.6777, 1169.5313, 1171.6676, 1179.5499, 1184.6520, 1185.6776, 1185.6776, 1189.6735, 1207.6569, 1211.6572, 1225.6708, 1300.7860, 1316.8134, 1334.8168, 1334.8168, 1440.5756, 1448.8479, 1448.8479, 1475.6846, 1522.7052, 1538.7010, 1554.6993, 1617.9867, 1617.9867, 1732.0165, 1901.1586, 2015.1949, 2211.0193, 2283.0847, 2305.0740, 2321.0552, 2528.2617

12. A0A4W2H686 **Mass:** 54723 **Score:** 39 **Expect:** 19 **Matches:** 12

Dihydrolipoyl dehydrogenase OS=Bos indicus x Bos taurus OX=30522 GN=DLD PE=3 SV=1

Observed	Mr(expt)	Mr(calc)	ppm	Start	End	Miss	Ions	Peptide
868.5015	867.4942	867.4524	48.2	7 -	13	0	---	R.VYCSLVK.R
868.5015	867.4943	867.4524	48.2	7 -	13	0	---	R.VYCSLVK.R
1017.6157	1016.6084	1016.5614	46.2	441 -	449	1	---	K.ILGQKSTDR.V
1017.6157	1016.6084	1016.5614	46.2	441 -	449	1	---	K.ILGQKSTDR.V
1151.6696	1150.6623	1150.5870	65.5	411 -	420	1	---	K.EEGIEYKVGK.F
1165.6809	1164.6736	1164.5631	94.9	123 -	132	1	---	K.MMEQKSNAVK.A
1165.6809	1164.6736	1164.5631	94.9	123 -	132	1	---	K.MMEQKSNAVK.A
1193.6417	1192.6344	1192.6353	-0.72	418 -	428	1	---	K.VGKFPFAANSR.A
1203.6858	1202.6785	1202.6772	1.14	337 -	346	1	---	R.IPVNTRFQTK.I
1279.7007	1278.6934	1278.6311	48.7	118 -	127	1	---	R.LNLEKMMEQK.S + Oxidation (M)
1533.7407	1532.7334	1532.7682	-22.69	285 -	300	1	---	K.SDGKIDVSIEAASGGK.A
1581.7728	1580.7655	1580.7569	5.44	405 -	417	1	---	K.SEEQLKEEGIEYK.V

No match to: 813.4393, 834.4629, 838.4906, 842.4641, 846.4475, 848.4780, 850.0118, 850.4896, 850.4896, 853.4691, 856.4822, 864.4857, 865.9818, 866.4929, 870.4977, 876.9884, 882.5173, 882.5173, 892.9574, 892.9574, 896.3969, 908.9280, 951.5720, 996.5430, 1019.6299, 1033.6429, 1045.5127, 1045.5127, 1051.6522, 1051.6522, 1065.6028, 1131.6493, 1133.6587, 1147.6777, 1169.5313, 1171.6676, 1179.5499, 1184.6520, 1185.6776, 1185.6776, 1189.6735, 1207.6569, 1211.6572, 1225.6708, 1300.7860, 1316.8134, 1334.8168, 1334.8168, 1440.5756, 1448.8479, 1448.8479, 1475.6846, 1522.7052, 1538.7010, 1554.6993, 1617.9867, 1617.9867, 1732.0165, 1901.1586, 2015.1949, 2211.0193, 2283.0847, 2305.0740, 2321.0552, 2528.2617

13. A0A4W2H5Z2 **Mass:** 33370 **Score:** 38 **Expect:** 19 **Matches:** 10

Ketohexokinase OS=Bos indicus x Bos taurus OX=30522 GN=KHK PE=4 SV=1

Observed	Mr(expt)	Mr(calc)	ppm	Start	End	Miss	Ions	Peptide
834.4629	833.4556	833.4065	58.9	273 -	279	0	---	K.SMQEALR.F
850.4896	849.4823	849.4821	0.25	210 -	216	1	---	R.GLYSRVR.K
850.4896	849.4823	849.4821	0.30	210 -	216	1	---	R.GLYSRVR.K
866.4929	865.4856	865.4116	85.5	280 -	287	0	---	R.FGCQVAGK.K
1065.6028	1064.5955	1064.4961	93.4	289 -	298	0	---	K.CGLQGFDGIV.-
1133.6587	1132.6514	1132.5560	84.3	32 -	39	1	---	R.CLSQRWQR.G
1185.6776	1184.6703	1184.6454	21.0	133 -	141	1	---	R.FKWIHIEGR.N
1185.6776	1184.6703	1184.6454	21.0	133 -	141	1	---	R.FKWIHIEGR.N
1193.6417	1192.6344	1192.5911	36.4	288 -	298	1	---	K.KCGLQGFDGIV.-
1533.7407	1532.7334	1532.7695	-23.55	153 -	165	1	---	R.IEQHNARQPPESK.I

No match to: 813.4393, 838.4906, 842.4641, 846.4475, 848.4780, 850.0118, 853.4691, 856.4822, 864.4857, 865.9818, 868.5015, 868.5015, 870.4977, 876.9884, 882.5173, 882.5173, 892.9574, 892.9574, 896.3969, 908.9280, 951.5720, 996.5430, 1017.6157, 1017.6157, 1019.6299, 1033.6429, 1045.5127, 1045.5127, 1051.6522, 1051.6522, 1131.6493, 1147.6777, 1151.6696, 1165.6809, 1165.6809, 1169.5313, 1171.6676, 1179.5499, 1184.6520, 1189.6735, 1203.6858, 1207.6569, 1211.6572, 1225.6708, 1279.7007, 1300.7860, 1316.8134, 1334.8168, 1334.8168, 1440.5756, 1448.8479, 1448.8479, 1475.6846, 1522.7052, 1538.7010, 1554.6993, 1581.7728, 1617.9867, 1617.9867, 1732.0165, 1901.1586, 2015.1949, 2211.0193, 2283.0847, 2305.0740, 2321.0552, 2528.2617

14. A0A3Q1N4Y2 **Mass:** 25122 **Score:** 38 **Expect:** 20 **Matches:** 13

Uncharacterized protein OS=Bos taurus OX=9913 PE=4 SV=1

Observed	Mr(expt)	Mr(calc)	ppm	Start	End	Miss	Ions	Peptide

PDF-XChange Click to buy NOW! www.docu-track.com
PDF-XChange Click to buy NOW! www.docu-track.com

838.4906	837.4833	837.5072	-28.53	49	55	0	---	K.DPKPIIR.D
866.4929	865.4856	865.4215	74.1	175	181	1	---	R.ETTMKEK.S
882.5173	881.5100	881.4454	73.3	5	13	1	---	R.STSSTKSGK.F
882.5173	881.5100	881.4454	73.3	5	13	1	---	R.STSSTKSGK.F
1165.6809	1164.6736	1164.6026	61.0	98	106	1	---	R.KLEVEYEQK.R
1165.6809	1164.6736	1164.6026	61.0	98	106	1	---	R.KLEVEYEQK.R
1193.6417	1192.6344	1192.6088	21.5	99	107	1	---	K.LEVEYEQKR.A
1207.6569	1206.6496	1206.5815	56.4	14	23	1	---	K.FMNPTDQARK.E
1522.7052	1521.6979	1521.7830	-55.88	162	174	1	---	K.VKGMNLCTVMTLR.E
1538.7010	1537.6937	1537.7779	-54.72	162	174	1	---	K.VKGMNLCTVMTLR.E + Oxidation (M)
1554.6993	1553.6920	1553.7728	-51.99	162	174	1	---	K.VKGMNLCTVMTLR.E + 2 Oxidation (M)
2211.0193	2210.0120	2210.1538	-64.16	144	163	1	---	R.LSMTTVLMTATVTDQVEKVK.G + Oxidation (M)
2528.2617	2527.2544	2527.2662	-4.67	140	161	1	---	K.TWTRLSMTTVLMTATVTDQVEK.V + Oxidation (M)

No match to: 813.4393, 834.4629, 842.4641, 846.4475, 848.4780, 850.0118, 850.4896, 850.4896, 853.4691, 856.4822, 864.4857, 865.9818, 868.5015, 868.5015, 870.4977, 876.9884, 892.9574, 892.9574, 896.3969, 908.9280, 951.5720, 996.5430, 1017.6157, 1017.6157, 1019.6299, 1033.6429, 1045.5127, 1045.5127, 1051.6522, 1051.6522, 1065.6028, 1131.6493, 1133.6587, 1147.6777, 1151.6696, 1169.5313, 1171.6676, 1179.5499, 1184.6520, 1185.6776, 1185.6776, 1189.6735, 1203.6858, 1211.6572, 1225.6708, 1279.7007, 1300.7860, 1316.8134, 1334.8168, 1334.8168, 1440.5756, 1448.8479, 1448.8479, 1475.6846, 1533.7407, 1581.7728, 1617.9867, 1617.9867, 1732.0165, 1901.1586, 2015.1949, 2283.0847, 2305.0740, 2321.0552

15. F6PSE2 **Mass:** 92262 **Score:** 38 **Expect:** 21 **Matches:** 13

Nibrin OS=Bos taurus OX=9913 GN=NBN PE=4 SV=2

Observed	Mr(expt)	Mr(calc)	ppm	Start	End	Miss	Ions	Peptide
846.4475	845.4402	845.4494	-10.87	305	311	0	---	R.LITEENK.E
850.4896	849.4823	849.4232	69.5	684	690	0	---	K.ESLWSTK.E
850.4896	849.4823	849.4232	69.6	684	690	0	---	K.ESLWSTK.E
951.5720	950.5647	950.5073	60.4	594	601	0	---	K.SLFTENLK.S
1131.6493	1130.6420	1130.6118	26.8	137	146	0	---	K.MQNGLSQILK.S
1147.6777	1146.6704	1146.6067	55.6	137	146	0	---	K.MQNGLSQILK.S + Oxidation (M)
1189.6735	1188.6662	1188.5775	74.7	771	779	0	---	K.NTELEEWLR.Q
1207.6569	1206.6496	1206.6318	14.8	438	447	1	---	K.EIKIFGMEPK.C + Oxidation (M)
1316.8134	1315.8061	1315.6925	86.4	151	161	1	---	R.VTFGVFESKFR.V
1522.7052	1521.6979	1521.8416	-94.39	16	29	1	---	R.LPGLWASGLPERAR.C
1538.7010	1537.6937	1537.7386	-29.17	498	510	0	---	R.GWPSQHQQTNSIR.D
2211.0193	2210.0120	2210.0559	-19.84	539	557	0	---	R.METSCSLLEQTQPTTSLIR.K + Oxidation (M)
2528.2617	2527.2544	2527.1761	31.0	771	790	1	---	K.NTELEEWLRQEMEVQNQHAK.E + Oxidation (M)

No match to: 813.4393, 834.4629, 838.4906, 842.4641, 848.4780, 850.0118, 853.4691, 856.4822, 864.4857, 865.9818, 866.4929, 868.5015, 868.5015, 870.4977, 876.9884, 882.5173, 882.5173, 892.9574, 892.9574, 896.3969, 908.9280, 996.5430, 1017.6157, 1017.6157, 1019.6299, 1033.6429, 1045.5127, 1045.5127, 1051.6522, 1051.6522, 1065.6028, 1133.6587, 1151.6696, 1165.6809, 1165.6809, 1169.5313, 1171.6676, 1179.5499, 1184.6520, 1185.6776, 1185.6776, 1193.6417, 1203.6858, 1211.6572, 1225.6708, 1279.7007, 1300.7860, 1334.8168, 1334.8168, 1440.5756, 1448.8479, 1448.8479, 1475.6846, 1533.7407, 1554.6993, 1581.7728, 1617.9867, 1617.9867, 1732.0165, 1901.1586, 2015.1949, 2283.0847, 2305.0740, 2321.0552

16. A0A4W2CAE8 **Mass:** 92262 **Score:** 38 **Expect:** 21 **Matches:** 13

Nibrin OS=Bos indicus x Bos taurus OX=30522 GN=NBN PE=4 SV=1

Observed	Mr(expt)	Mr(calc)	ppm	Start	End	Miss	Ions	Peptide
846.4475	845.4402	845.4494	-10.87	305	311	0	---	R.LITEENK.E
850.4896	849.4823	849.4232	69.5	684	690	0	---	K.ESLWSTK.E
850.4896	849.4823	849.4232	69.6	684	690	0	---	K.ESLWSTK.E
951.5720	950.5647	950.5073	60.4	594	601	0	---	K.SLFTENLK.S
1131.6493	1130.6420	1130.6118	26.8	137	146	0	---	K.MQNGLSQILK.S
1147.6777	1146.6704	1146.6067	55.6	137	146	0	---	K.MQNGLSQILK.S + Oxidation (M)
1189.6735	1188.6662	1188.5775	74.7	771	779	0	---	K.NTELEEWLR.Q
1207.6569	1206.6496	1206.6318	14.8	438	447	1	---	K.EIKIFGMEPK.C + Oxidation (M)
1316.8134	1315.8061	1315.6925	86.4	151	161	1	---	R.VTFGVFESKFR.V
1522.7052	1521.6979	1521.8416	-94.39	16	29	1	---	R.LPGLWASGLPERAR.C
1538.7010	1537.6937	1537.7386	-29.17	498	510	0	---	R.GWPSQHQQTNSIR.D
2211.0193	2210.0120	2210.0559	-19.84	539	557	0	---	R.METSCSLLEQTQPTTSLIR.K + Oxidation (M)
2528.2617	2527.2544	2527.1761	31.0	771	790	1	---	K.NTELEEWLRQEMEVQNQHAK.E + Oxidation (M)

No match to: 813.4393, 834.4629, 838.4906, 842.4641, 848.4780, 850.0118, 853.4691, 856.4822, 864.4857, 865.9818, 866.4929, 868.5015, 868.5015, 870.4977, 876.9884, 882.5173, 882.5173, 892.9574, 892.9574, 896.3969, 908.9280, 996.5430, 1017.6157, 1017.6157, 1019.6299, 1033.6429, 1045.5127, 1045.5127, 1051.6522, 1051.6522, 1065.6028, 1133.6587, 1151.6696, 1165.6809, 1165.6809, 1169.5313, 1171.6676, 1179.5499, 1184.6520, 1185.6776, 1185.6776, 1193.6417, 1203.6858, 1211.6572, 1225.6708, 1279.7007, 1300.7860, 1334.8168, 1334.8168, 1440.5756, 1448.8479, 1448.8479, 1475.6846, 1533.7407, 1554.6993, 1581.7728, 1617.9867, 1617.9867, 1732.0165, 1901.1586, 2015.1949, 2283.0847, 2305.0740, 2321.0552

17. A0A4W2BNU6 **Mass:** 41726 **Score:** 38 **Expect:** 21 **Matches:** 11

Dehydrogenase/reductase 12 OS=Bos indicus x Bos taurus OX=30522 GN=DHRS12 PE=4 SV=1

Observed	Mr(expt)	Mr(calc)	ppm	Start	End	Miss	Ions	Peptide
834.4629	833.4556	833.4317	28.8	55	62	0	---	K.ATAMEIAK.R
850.4896	849.4823	849.4266	65.6	55	62	0	---	K.ATAMEIAK.R + Oxidation (M)
850.4896	849.4823	849.4266	65.6	55	62	0	---	K.ATAMEIAK.R + Oxidation (M)
866.4929	865.4856	865.4657	23.0	13	19	1	---	K.GLREYTK.S
1133.6587	1132.6514	1132.5989	46.4	224	233	1	---	R.VEVRGFSSPR.G
1189.6735	1188.6662	1188.5986	56.9	133	142	1	---	K.RELTEDGLEK.N
1334.8168	1333.8095	1333.7276	61.4	170	182	0	---	R.VITVSSGGMLVQK.L + Oxidation (M)
1334.8168	1333.8095	1333.7276	61.5	170	182	0	---	R.VITVSSGGMLVQK.L + Oxidation (M)
1533.7407	1532.7334	1532.6936	26.0	359	371	1	---	R.NRTCVSHVSCTGR.Q
1901.1586	1900.1513	1900.0101	74.3	342	358	1	---	R.ILGWVVMPSSRGSSRPR.N + Oxidation (M)
2211.0193	2210.0120	2210.1048	-41.98	115	133	1	---	K.QEHTLNVLINNAGCMVNKR.E

No match to: 813.4393, 838.4906, 842.4641, 846.4475, 848.4780, 850.0118, 853.4691, 856.4822, 864.4857, 865.9818, 868.5015, 868.5015, 870.4977, 876.9884, 882.5173, 882.5173, 892.9574, 892.9574, 896.3969, 908.9280, 951.5720, 996.5430, 1017.6157, 1017.6157, 1019.6299, 1033.6429, 1045.5127, 1045.5127, 1051.6522, 1051.6522, 1065.6028, 1131.6493, 1147.6777, 1151.6696, 1165.6809, 1165.6809, 1169.5313, 1171.6676, 1179.5499, 1184.6520, 1185.6776, 1185.6776, 1193.6417, 1203.6858, 1207.6569, 1211.6572, 1225.6708, 1279.7007, 1300.7860, 1316.8134, 1440.5756, 1448.8479, 1448.8479, 1475.6846, 1522.7052, 1538.7010, 1554.6993, 1581.7728, 1617.9867, 1617.9867, 1732.0165, 2015.1949, 2283.0847, 2305.0740, 2321.0552, 2528.2617

PDF-XChange Click to buy NOW! www.docu-track.com

18. A0A3Q1M3R6 **Mass:** 41726 **Score:** 38 **Expect:** 21 **Matches:** 11
Dehydrogenase/reductase SDR family member 12 OS=Bos taurus OX=9913 GN=DHRS12 PE=4 SV=1

Observed	Mr(expt)	Mr(calc)	ppm	Start	End	Miss	Ions	Peptide
834.4629	833.4556	833.4317	28.8	55	62	0	---	K.ATAMEIAK.R
850.4896	849.4823	849.4266	65.6	55	62	0	---	K.ATAMEIAK.R + Oxidation (M)
850.4896	849.4823	849.4266	65.6	55	62	0	---	K.ATAMEIAK.R + Oxidation (M)
866.4929	865.4856	865.4657	23.0	13	19	1	---	K.GLREYTK.S
1133.6587	1132.6514	1132.5989	46.4	224	233	1	---	R.VEVRGFSSPR.G
1189.6735	1188.6662	1188.5986	56.9	133	142	1	---	K.RELTEDGLEK.N
1334.8168	1333.8095	1333.7276	61.4	170	182	0	---	R.VITVSSGGMLVQK.L + Oxidation (M)
1334.8168	1333.8095	1333.7276	61.5	170	182	0	---	R.VITVSSGGMLVQK.L + Oxidation (M)
1533.7407	1532.7334	1532.6936	26.0	359	371	1	---	R.NRTCVSHVSCTGR.Q
1901.1586	1900.1513	1900.0101	74.3	342	358	1	---	R.ILGWVVMPSSRGSSRPR.N + Oxidation (M)
2211.0193	2210.0120	2210.1048	-41.98	115	133	1	---	K.QEHTLNVLINNAGCMVNKR.E

No match to: 813.4393, 838.4906, 842.4641, 846.4475, 848.4780, 850.0118, 853.4691, 856.4822, 864.4857, 865.9818, 868.5015, 868.5015, 870.4977, 876.9884, 882.5173, 882.5173, 892.9574, 892.9574, 896.3969, 908.9280, 951.5720, 996.5430, 1017.6157, 1017.6157, 1019.6299, 1033.6429, 1045.5127, 1045.5127, 1051.6522, 1051.6522, 1065.6028, 1131.6493, 1147.6777, 1151.6696, 1165.6809, 1165.6809, 1169.5313, 1171.6676, 1179.5499, 1184.6520, 1185.6776, 1185.6776, 1193.6417, 1203.6858, 1207.6569, 1211.6572, 1225.6708, 1279.7007, 1300.7860, 1316.8134, 1440.5756, 1448.8479, 1448.8479, 1475.6846, 1522.7052, 1538.7010, 1554.6993, 1581.7728, 1617.9867, 1617.9867, 1732.0165, 2015.1949, 2283.0847, 2305.0740, 2321.0552, 2528.2617

19. A0A4W2E9U7 **Mass:** 76824 **Score:** 38 **Expect:** 21 **Matches:** 12
Nibrin OS=Bos indicus x Bos taurus OX=30522 GN=NBN PE=4 SV=1

Observed	Mr(expt)	Mr(calc)	ppm	Start	End	Miss	Ions	Peptide
846.4475	845.4402	845.4494	-10.87	169	175	0	---	R.LITEENK.E
850.4896	849.4823	849.4232	69.5	547	553	0	---	K.ESLWSTK.E
850.4896	849.4823	849.4232	69.6	547	553	0	---	K.ESLWSTK.E
951.5720	950.5647	950.5073	60.4	457	464	0	---	K.SLFTENLK.S
1131.6493	1130.6420	1130.6118	26.8	1	10	0	---	-.MQNGLSQILK.S
1147.6777	1146.6704	1146.6067	55.6	1	10	0	---	-.MQNGLSQILK.S + Oxidation (M)
1189.6735	1188.6662	1188.5775	74.7	634	642	0	---	K.NTELEEWLR.Q
1207.6569	1206.6496	1206.6318	14.8	301	310	1	---	K.EIKIFGMEPK.C + Oxidation (M)
1316.8134	1315.8061	1315.6925	86.4	15	25	1	---	R.VTFGVFESKFR.V
1538.7010	1537.6937	1537.7386	-29.17	361	373	0	---	R.GWPSQHQQTNSIR.D
2211.0193	2210.0120	2210.0559	-19.84	402	420	0	---	R.METSCSLLEQTQPTTSLIR.K + Oxidation (M)
2528.2617	2527.2544	2527.1761	31.0	634	653	1	---	K.NTELEEWLRQEMEVQNQHAK.E + Oxidation (M)

No match to: 813.4393, 834.4629, 838.4906, 842.4641, 848.4780, 850.0118, 853.4691, 856.4822, 864.4857, 865.9818, 866.4929, 868.5015, 868.5015, 870.4977, 876.9884, 882.5173, 882.5173, 892.9574, 892.9574, 896.3969, 908.9280, 996.5430, 1017.6157, 1017.6157, 1019.6299, 1033.6429, 1045.5127, 1045.5127, 1051.6522, 1051.6522, 1065.6028, 1133.6587, 1151.6696, 1165.6809, 1165.6809, 1169.5313, 1171.6676, 1179.5499, 1184.6520, 1185.6776, 1185.6776, 1193.6417, 1203.6858, 1211.6572, 1225.6708, 1279.7007, 1300.7860, 1334.8168, 1334.8168, 1440.5756, 1448.8479, 1448.8479, 1475.6846, 1522.7052, 1533.7407, 1554.6993, 1581.7728, 1617.9867, 1617.9867, 1732.0165, 1901.1586, 2015.1949, 2283.0847, 2305.0740, 2321.0552

20. A0A4W2E4S5 **Mass:** 35275 **Score:** 38 **Expect:** 23 **Matches:** 7
Kallikrein related peptidase 11 OS=Bos indicus x Bos taurus OX=30522 GN=KLK11 PE=3 SV=1

Observed	Mr(expt)	Mr(calc)	ppm	Start	End	Miss	Ions	Peptide
848.4780	847.4707	847.4473	27.6	177	183	0	---	R.NDIMLVK.M + Oxidation (M)
951.5720	950.5647	950.4862	82.7	307	313	0	---	K.YVDWIQK.T
1179.5499	1178.5426	1178.6482	-89.54	297	306	1	---	K.KPGVYTKVCK.Y
1184.6520	1183.6447	1183.6747	-25.32	113	123	0	---	R.LLCGATLIAPR.W
1211.6572	1210.6499	1210.6604	-8.69	231	240	1	---	R.CANVTIIKHR.E
2015.1949	2014.1876	2013.9908	97.7	156	173	1	---	R.TATKSFPHPDFNNSLPNK.D
2283.0847	2282.0774	2282.0975	-8.78	136	155	1	---	R.YVVHLGAHSLGRQDGCEQTR.T

No match to: 813.4393, 834.4629, 838.4906, 842.4641, 846.4475, 850.0118, 850.4896, 850.4896, 853.4691, 856.4822, 864.4857, 865.9818, 866.4929, 868.5015, 868.5015, 870.4977, 876.9884, 882.5173, 882.5173, 892.9574, 892.9574, 896.3969, 908.9280, 996.5430, 1017.6157, 1017.6157, 1019.6299, 1033.6429, 1045.5127, 1045.5127, 1051.6522, 1051.6522, 1065.6028, 1131.6493, 1133.6587, 1147.6777, 1151.6696, 1165.6809, 1165.6809, 1169.5313, 1171.6676, 1185.6776, 1185.6776, 1189.6735, 1193.6417, 1203.6858, 1207.6569, 1225.6708, 1279.7007, 1300.7860, 1316.8134, 1334.8168, 1334.8168, 1440.5756, 1448.8479, 1448.8479, 1475.6846, 1522.7052, 1533.7407, 1538.7010, 1554.6993, 1581.7728, 1617.9867, 1617.9867, 1732.0165, 1901.1586, 2211.0193, 2305.0740, 2321.0552, 2528.2617

21. Q5EA35 **Mass:** 33326 **Score:** 37 **Expect:** 24 **Matches:** 10
Ketohexokinase OS=Bos taurus OX=9913 GN=KHK PE=2 SV=1

Observed	Mr(expt)	Mr(calc)	ppm	Start	End	Miss	Ions	Peptide
834.4629	833.4556	833.4065	58.9	273	279	0	---	K.SMQEALR.F
850.4896	849.4823	849.4821	0.25	210	216	1	---	R.GLYSRVR.K
850.4896	849.4823	849.4821	0.30	210	216	1	---	R.GLYSRVR.K
866.4929	865.4856	865.4116	85.5	280	287	0	---	R.FGCQVAGK.K
1065.6028	1064.5955	1064.4961	93.4	289	298	0	---	K.CGLQGFDGIV.-
1133.6587	1132.6514	1132.5560	84.3	32	39	1	---	R.CLSQRWQR.G
1185.6776	1184.6703	1184.6454	21.0	133	141	1	---	R.FKWIHIEGR.N
1185.6776	1184.6703	1184.6454	21.0	133	141	1	---	R.FKWIHIEGR.N
1193.6417	1192.6344	1192.5911	36.4	288	298	1	---	K.KCGLQGFDGIV.-
1533.7407	1532.7334	1532.7695	-23.55	153	165	1	---	R.IEQHNARQPPESK.I

No match to: 813.4393, 838.4906, 842.4641, 846.4475, 848.4780, 850.0118, 853.4691, 856.4822, 864.4857, 865.9818, 868.5015, 868.5015, 870.4977, 876.9884, 882.5173, 882.5173, 892.9574, 892.9574, 896.3969, 908.9280, 951.5720, 996.5430, 1017.6157, 1017.6157, 1019.6299, 1033.6429, 1045.5127, 1045.5127, 1051.6522, 1051.6522, 1131.6493, 1147.6777, 1151.6696, 1165.6809, 1165.6809, 1169.5313, 1171.6676, 1179.5499, 1184.6520, 1189.6735, 1203.6858, 1207.6569, 1211.6572, 1225.6708, 1279.7007, 1300.7860, 1316.8134, 1334.8168, 1334.8168, 1440.5756, 1448.8479, 1448.8479, 1475.6846, 1522.7052, 1538.7010, 1554.6993, 1581.7728, 1617.9867, 1617.9867, 1732.0165, 1901.1586, 2015.1949, 2211.0193, 2283.0847, 2305.0740, 2321.0552, 2528.2617

22. Q0II75 **Mass:** 38414 **Score:** 37 **Expect:** 24 **Matches:** 10
Ketohexokinase OS=Bos taurus OX=9913 GN=KHK PE=1 SV=1

Observed	Mr(expt)	Mr(calc)	ppm	Start	End	Miss	Ions	Peptide
834.4629	833.4556	833.4065	58.9	318	324	0	---	K.SMQEALR.F
850.4896	849.4823	849.4821	0.25	255	261	1	---	R.GLYSRVR.K

PDF-XChange Click to buy NOW! www.docu-track.com

PDF-XChange Click to buy NOW! www.docu-track.com

850.4896	849.4823	849.4821	0.30	255	261	1	---	R.GLYSRVR.K
866.4929	865.4856	865.4116	85.5	325	332	0	---	R.FGCQVAGK.K
1065.6028	1064.5955	1064.4961	93.4	334	343	0	---	K.CGLQGFDGIV.-
1133.6587	1132.6514	1132.5560	84.3	32	39	1	---	R.CLSQRWQR.G
1185.6776	1184.6703	1184.6454	21.0	178	186	1	---	R.FKWIHIEGR.N
1185.6776	1184.6703	1184.6454	21.0	178	186	1	---	R.FKWIHIEGR.N
1193.6417	1192.6344	1192.5911	36.4	333	343	1	---	K.KCGLQGFDGIV.-
1533.7407	1532.7334	1532.7695	-23.55	198	210	1	---	R.IEQHNARQPPESK.I

No match to: 813.4393, 838.4906, 842.4641, 846.4475, 848.4780, 850.0118, 853.4691, 856.4822, 864.4857, 865.9818, 868.5015, 868.5015, 870.4977, 876.9884, 882.5173, 882.5173, 892.9574, 892.9574, 896.3969, 908.9280, 951.5720, 996.5430, 1017.6157, 1017.6157, 1019.6299, 1033.6429, 1045.5127, 1045.5127, 1051.6522, 1051.6522, 1131.6493, 1147.6777, 1151.6696, 1165.6809, 1165.6809, 1169.5313, 1171.6676, 1179.5499, 1184.6520, 1189.6735, 1203.6858, 1207.6569, 1211.6572, 1225.6708, 1279.7007, 1300.7860, 1316.8134, 1334.8168, 1334.8168, 1440.5756, 1448.8479, 1448.8479, 1475.6846, 1522.7052, 1538.7010, 1554.6993, 1581.7728, 1617.9867, 1617.9867, 1732.0165, 1901.1586, 2015.1949, 2211.0193, 2283.0847, 2305.0740, 2321.0552, 2528.2617

23. A0A4W2EMU6 **Mass:** 31365 **Score:** 36 **Expect:** 33 **Matches:** 9

Ketohexokinase OS=Bos indicus x Bos taurus OX=30522 GN=KHK PE=4 SV=1

Observed	Mr(expt)	Mr(calc)	ppm	Start	End	Miss	Ions	Peptide
834.4629	833.4556	833.4065	58.9	257	263	0	---	K.SMQEALR.F
850.4896	849.4823	849.4821	0.25	194	200	1	---	R.GLYSRVR.K
850.4896	849.4823	849.4821	0.30	194	200	1	---	R.GLYSRVR.K
866.4929	865.4856	865.4116	85.5	264	271	0	---	R.FGCQVAGK.K
1065.6028	1064.5955	1064.4961	93.4	273	282	0	---	K.CGLQGFDGIV.-
1185.6776	1184.6703	1184.6454	21.0	117	125	1	---	R.FKWIHIEGR.N
1185.6776	1184.6703	1184.6454	21.0	117	125	1	---	R.FKWIHIEGR.N
1193.6417	1192.6344	1192.5911	36.4	272	282	1	---	K.KCGLQGFDGIV.-
1533.7407	1532.7334	1532.7695	-23.55	137	149	1	---	R.IEQHNARQPPESK.I

No match to: 813.4393, 838.4906, 842.4641, 846.4475, 848.4780, 850.0118, 853.4691, 856.4822, 864.4857, 865.9818, 868.5015, 868.5015, 870.4977, 876.9884, 882.5173, 882.5173, 892.9574, 892.9574, 896.3969, 908.9280, 951.5720, 996.5430, 1017.6157, 1017.6157, 1019.6299, 1033.6429, 1045.5127, 1045.5127, 1051.6522, 1051.6522, 1131.6493, 1133.6587, 1147.6777, 1151.6696, 1165.6809, 1165.6809, 1169.5313, 1171.6676, 1179.5499, 1184.6520, 1189.6735, 1203.6858, 1207.6569, 1211.6572, 1225.6708, 1279.7007, 1300.7860, 1316.8134, 1334.8168, 1334.8168, 1440.5756, 1448.8479, 1448.8479, 1475.6846, 1522.7052, 1538.7010, 1554.6993, 1581.7728, 1617.9867, 1617.9867, 1732.0165, 1901.1586, 2015.1949, 2211.0193, 2283.0847, 2305.0740, 2321.0552, 2528.2617

24. A0A4W2G3Z2 **Mass:** 37905 **Score:** 36 **Expect:** 35 **Matches:** 7

Kallikrein related peptidase 11 OS=Bos indicus x Bos taurus OX=30522 GN=KLK11 PE=3 SV=1

Observed	Mr(expt)	Mr(calc)	ppm	Start	End	Miss	Ions	Peptide
848.4780	847.4707	847.4473	27.6	202	208	0	---	R.NDIMLVK.M + Oxidation (M)
951.5720	950.5647	950.4862	82.7	332	338	0	---	K.YVDWIQK.T
1179.5499	1178.5426	1178.6482	-89.54	322	331	1	---	K.KPGVYTKVCK.Y
1184.6520	1183.6447	1183.6747	-25.32	138	148	0	---	R.LLCGATLIAPR.W
1211.6572	1210.6499	1210.6604	-8.69	256	265	1	---	R.CANVTIIKHR.E
2015.1949	2014.1876	2013.9908	97.7	181	198	1	---	R.TATKSFPHPDFNNSLPNK.D
2283.0847	2282.0774	2282.0975	-8.78	161	180	1	---	R.YVVHLGAHSLGRQDGCEQTR.T

No match to: 813.4393, 834.4629, 838.4906, 842.4641, 846.4475, 850.0118, 850.4896, 850.4896, 853.4691, 856.4822, 864.4857, 865.9818, 866.4929, 868.5015, 868.5015, 870.4977, 876.9884, 882.5173, 882.5173, 892.9574, 892.9574, 896.3969, 908.9280, 996.5430, 1017.6157, 1017.6157, 1019.6299, 1033.6429, 1045.5127, 1045.5127, 1051.6522, 1051.6522, 1065.6028, 1131.6493, 1133.6587, 1147.6777, 1151.6696, 1165.6809, 1165.6809, 1169.5313, 1171.6676, 1185.6776, 1185.6776, 1189.6735, 1193.6417, 1203.6858, 1207.6569, 1225.6708, 1279.7007, 1300.7860, 1316.8134, 1334.8168, 1334.8168, 1440.5756, 1448.8479, 1448.8479, 1475.6846, 1522.7052, 1533.7407, 1538.7010, 1554.6993, 1581.7728, 1617.9867, 1617.9867, 1732.0165, 1901.1586, 2211.0193, 2305.0740, 2321.0552, 2528.2617

25. F1N4M1 **Mass:** 37905 **Score:** 36 **Expect:** 35 **Matches:** 7

Kallikrein related peptidase 11 OS=Bos taurus OX=9913 GN=KLK11 PE=3 SV=3

Observed	Mr(expt)	Mr(calc)	ppm	Start	End	Miss	Ions	Peptide
848.4780	847.4707	847.4473	27.6	202	208	0	---	R.NDIMLVK.M + Oxidation (M)
951.5720	950.5647	950.4862	82.7	332	338	0	---	K.YVDWIQK.T
1179.5499	1178.5426	1178.6482	-89.54	322	331	1	---	K.KPGVYTKVCK.Y
1184.6520	1183.6447	1183.6747	-25.32	138	148	0	---	R.LLCGATLIAPR.W
1211.6572	1210.6499	1210.6604	-8.69	256	265	1	---	R.CANVTIIKHR.E
2015.1949	2014.1876	2013.9908	97.7	181	198	1	---	R.TATKSFPHPDFNNSLPNK.D
2283.0847	2282.0774	2282.0975	-8.78	161	180	1	---	R.YVVHLGAHSLGRQDGCEQTR.T

No match to: 813.4393, 834.4629, 838.4906, 842.4641, 846.4475, 850.0118, 850.4896, 850.4896, 853.4691, 856.4822, 864.4857, 865.9818, 866.4929, 868.5015, 868.5015, 870.4977, 876.9884, 882.5173, 882.5173, 892.9574, 892.9574, 896.3969, 908.9280, 996.5430, 1017.6157, 1017.6157, 1019.6299, 1033.6429, 1045.5127, 1045.5127, 1051.6522, 1051.6522, 1065.6028, 1131.6493, 1133.6587, 1147.6777, 1151.6696, 1165.6809, 1165.6809, 1169.5313, 1171.6676, 1185.6776, 1185.6776, 1189.6735, 1193.6417, 1203.6858, 1207.6569, 1225.6708, 1279.7007, 1300.7860, 1316.8134, 1334.8168, 1334.8168, 1440.5756, 1448.8479, 1448.8479, 1475.6846, 1522.7052, 1533.7407, 1538.7010, 1554.6993, 1581.7728, 1617.9867, 1617.9867, 1732.0165, 1901.1586, 2211.0193, 2305.0740, 2321.0552, 2528.2617

26. A0A4W2H4X6 **Mass:** 31395 **Score:** 35 **Expect:** 39 **Matches:** 9

Ketohexokinase OS=Bos indicus x Bos taurus OX=30522 GN=KHK PE=4 SV=1

Observed	Mr(expt)	Mr(calc)	ppm	Start	End	Miss	Ions	Peptide
834.4629	833.4556	833.4065	58.9	257	263	0	---	K.SMQEALR.F
850.4896	849.4823	849.4821	0.25	194	200	1	---	R.GLYSRVR.K
850.4896	849.4823	849.4821	0.30	194	200	1	---	R.GLYSRVR.K
866.4929	865.4856	865.4116	85.5	264	271	0	---	R.FGCQVAGK.K
1065.6028	1064.5955	1064.4961	93.4	273	282	0	---	K.CGLQGFDGIV.-
1185.6776	1184.6703	1184.6454	21.0	117	125	1	---	R.FKWIHIEGR.N
1185.6776	1184.6703	1184.6454	21.0	117	125	1	---	R.FKWIHIEGR.N
1193.6417	1192.6344	1192.5911	36.4	272	282	1	---	K.KCGLQGFDGIV.-
1533.7407	1532.7334	1532.7695	-23.55	137	149	1	---	R.IEQHNARQPPESK.I

No match to: 813.4393, 838.4906, 842.4641, 846.4475, 848.4780, 850.0118, 853.4691, 856.4822, 864.4857, 865.9818, 868.5015, 868.5015, 870.4977, 876.9884, 882.5173, 882.5173, 892.9574, 892.9574, 896.3969, 908.9280, 951.5720, 996.5430, 1017.6157, 1017.6157, 1019.6299, 1033.6429, 1045.5127, 1045.5127, 1051.6522, 1051.6522, 1131.6493, 1133.6587, 1147.6777, 1151.6696, 1165.6809, 1165.6809, 1169.5313, 1171.6676, 1179.5499, 1184.6520, 1189.6735, 1203.6858, 1207.6569, 1211.6572, 1225.6708, 1279.7007, 1300.7860, 1316.8134, 1334.8168, 1334.8168, 1440.5756, 1448.8479, 1448.8479, 1475.6846, 1522.7052, 1538.7010, 1554.6993, 1581.7728, 1617.9867, 1617.9867, 1732.0165, 1901.1586, 2015.1949, 2211.0193, 2283.0847, 2305.0740, 2321.0552, 2528.2617

PDF-XChange Click to buy NOW! www.docu-track.com

PDF-XChange Click to buy NOW! www.docu-track.com

27. A0A4W2C9V0 **Mass:** 38733 **Score:** 35 **Expect:** 40 **Matches:** 8
Uncharacterized protein OS=Bos indicus x Bos taurus OX=30522 GN=MFGE8 PE=4 SV=1

Observed	Mr(expt)	Mr(calc)	ppm	Start	End	Miss	Ions	Peptide
838.4906	837.4833	837.4232	71.8	105	112	0	---	R.AGSAEYLK.T
842.4641	841.4568	841.4446	14.5	57	63	0	---	R.WAPELAR.L
868.5015	867.4942	867.4086	98.7	116	123	0	---	K.VAYSTDGR.Q
868.5015	867.4943	867.4086	98.7	116	123	0	---	K.VAYSTDGR.Q
1171.6676	1170.6603	1170.6833	-19.63	255	265	1	---	R.RVTGIITQGAR.D
1193.6417	1192.6344	1192.6353	-0.73	124	133	0	---	R.QFQFIQVAGR.S
1522.7052	1521.6979	1521.8317	-87.90	323	334	1	---	R.FVRIQPVAWHNR.I
2211.0193	2210.0120	2210.1154	-46.77	92	112	1	---	K.MWVTGVVTQGASRAGSAEYLK.T

No match to: 813.4393, 834.4629, 846.4475, 848.4780, 850.0118, 850.4896, 850.4896, 853.4691, 856.4822, 864.4857, 865.9818, 866.4929, 870.4977, 876.9884, 882.5173, 882.5173, 892.9574, 892.9574, 896.3969, 908.9280, 951.5720, 996.5430, 1017.6157, 1017.6157, 1019.6299, 1033.6429, 1045.5127, 1045.5127, 1051.6522, 1051.6522, 1065.6028, 1131.6493, 1133.6587, 1147.6777, 1151.6696, 1165.6809, 1165.6809, 1169.5313, 1179.5499, 1184.6520, 1185.6776, 1185.6776, 1189.6735, 1203.6858, 1207.6569, 1211.6572, 1225.6708, 1279.7007, 1300.7860, 1316.8134, 1334.8168, 1334.8168, 1440.5756, 1448.8479, 1448.8479, 1475.6846, 1533.7407, 1538.7010, 1554.6993, 1581.7728, 1617.9867, 1617.9867, 1732.0165, 1901.1586, 2015.1949, 2283.0847, 2305.0740, 2321.0552, 2528.2617

28. A0A4W2F3R1 **Mass:** 41965 **Score:** 34 **Expect:** 51 **Matches:** 9
Reverse transcriptase domain-containing protein OS=Bos indicus x Bos taurus OX=30522 PE=4 SV=1

Observed	Mr(expt)	Mr(calc)	ppm	Start	End	Miss	Ions	Peptide
834.4629	833.4556	833.5044	-58.57	173	179	1	---	K.SLLMKVK.E + Oxidation (M)
848.4780	847.4707	847.4287	49.6	178	184	1	---	K.VKEESEK.V
1131.6493	1130.6420	1130.5720	61.9	16	25	0	---	R.ELPDVQAGFR.K
1133.6587	1132.6514	1132.6162	31.1	242	251	0	---	K.VMTNLDSILK.S
1179.5499	1178.5426	1178.5655	-19.40	334	342	0	---	R.QYFGHLMQR.V
1193.6417	1192.6344	1192.5836	42.6	89	99	0	---	R.NLYAGQEATAR.T
1533.7407	1532.7334	1532.7578	-15.92	317	330	0	---	K.EISPGCSLEGLMLK.L
1554.6993	1553.6920	1553.7694	-49.80	76	88	0	---	K.EMGIPDHLTCLLR.N
1581.7728	1580.7655	1580.7697	-2.65	49	60	1	---	R.KNIYFCFIDYAK.A

No match to: 813.4393, 838.4906, 842.4641, 846.4475, 850.0118, 850.4896, 850.4896, 853.4691, 856.4822, 864.4857, 865.9818, 866.4929, 868.5015, 868.5015, 870.4977, 876.9884, 882.5173, 882.5173, 892.9574, 892.9574, 896.3969, 908.9280, 951.5720, 996.5430, 1017.6157, 1017.6157, 1019.6299, 1033.6429, 1045.5127, 1045.5127, 1051.6522, 1051.6522, 1065.6028, 1147.6777, 1151.6696, 1165.6809, 1165.6809, 1169.5313, 1171.6676, 1184.6520, 1185.6776, 1185.6776, 1189.6735, 1203.6858, 1207.6569, 1211.6572, 1225.6708, 1279.7007, 1300.7860, 1316.8134, 1334.8168, 1334.8168, 1440.5756, 1448.8479, 1448.8479, 1475.6846, 1522.7052, 1538.7010, 1617.9867, 1617.9867, 1732.0165, 1901.1586, 2015.1949, 2211.0193, 2283.0847, 2305.0740, 2321.0552, 2528.2617

29. A0A4W2IP26 **Mass:** 92361 **Score:** 34 **Expect:** 60 **Matches:** 11
Nibrin OS=Bos indicus x Bos taurus OX=30522 GN=NBN PE=4 SV=1

Observed	Mr(expt)	Mr(calc)	ppm	Start	End	Miss	Ions	Peptide
846.4475	845.4402	845.4494	-10.87	305	311	0	---	R.LITEENK.E
951.5720	950.5647	950.5073	60.4	594	601	0	---	K.SLFTENLK.S
1131.6493	1130.6420	1130.6118	26.8	137	146	0	---	K.MQNGLSQILK.S
1147.6777	1146.6704	1146.6067	55.6	137	146	0	---	K.MQNGLSQILK.S + Oxidation (M)
1189.6735	1188.6662	1188.5775	74.7	771	779	0	---	K.NTELEEWLR.Q
1207.6569	1206.6496	1206.6318	14.8	438	447	1	---	K.EIKIFGMEPK.C + Oxidation (M)
1316.8134	1315.8061	1315.6925	86.4	151	161	1	---	R.VTFGVFESKFR.V
1522.7052	1521.6979	1521.8416	-94.39	16	29	1	---	R.LPGLWASGLPERAR.C
1538.7010	1537.6937	1537.7386	-29.17	498	510	0	---	R.GWPSQHQQTNSIR.D
2211.0193	2210.0120	2210.0559	-19.84	539	557	0	---	R.METSCSLLEQTQPTTSLIR.K + Oxidation (M)
2528.2617	2527.2544	2527.1761	31.0	771	790	1	---	K.NTELEEWLRQEMEVQNQHAK.E + Oxidation (M)

No match to: 813.4393, 834.4629, 838.4906, 842.4641, 848.4780, 850.0118, 850.4896, 850.4896, 853.4691, 856.4822, 864.4857, 865.9818, 866.4929, 868.5015, 868.5015, 870.4977, 876.9884, 882.5173, 882.5173, 892.9574, 892.9574, 896.3969, 908.9280, 996.5430, 1017.6157, 1017.6157, 1019.6299, 1033.6429, 1045.5127, 1045.5127, 1051.6522, 1051.6522, 1065.6028, 1133.6587, 1151.6696, 1165.6809, 1165.6809, 1169.5313, 1171.6676, 1179.5499, 1184.6520, 1185.6776, 1185.6776, 1193.6417, 1203.6858, 1211.6572, 1225.6708, 1279.7007, 1300.7860, 1334.8168, 1334.8168, 1440.5756, 1448.8479, 1448.8479, 1475.6846, 1533.7407, 1554.6993, 1581.7728, 1617.9867, 1617.9867, 1732.0165, 1901.1586, 2015.1949, 2283.0847, 2305.0740, 2321.0552

30. A0A1C9HI59 **Mass:** 67468 **Score:** 34 **Expect:** 60 **Matches:** 9
LS037 OS=Lumpy skin disease virus OX=59509 GN=LW037 PE=4 SV=1

Observed	Mr(expt)	Mr(calc)	ppm	Start	End	Miss	Ions	Peptide
846.4475	845.4402	845.4793	-46.19	272	278	1	---	R.VNKNMLK.L
853.4691	852.4618	852.4276	40.2	1	6	1	---	-.MDFIRR.K + Oxidation (M)
856.4822	855.4749	855.4854	-12.28	449	455	1	---	K.KFSLFSK.N
1065.6028	1064.5955	1064.5695	24.5	317	324	1	---	K.KYFIGFYK.E
1184.6520	1183.6447	1183.5945	42.4	546	555	1	---	K.TNHLTNNDKK.Y
1279.7007	1278.6934	1278.6707	17.7	108	118	0	---	K.TLVSELFLDDK.F
1901.1586	1900.1513	1900.0492	53.7	484	499	1	---	K.ILSHLKCVEDVTVFIK.F
2015.1949	2014.1876	2014.0081	89.1	427	443	1	---	K.EKDPYILYNGMLTNISK.V + Oxidation (M)
2283.0847	2282.0774	2282.1616	-36.90	429	448	1	---	K.DPYILYNGMLTNISKVPSNK.K + Oxidation (M)

No match to: 813.4393, 834.4629, 838.4906, 842.4641, 848.4780, 850.0118, 850.4896, 850.4896, 864.4857, 865.9818, 866.4929, 868.5015, 868.5015, 870.4977, 876.9884, 882.5173, 882.5173, 892.9574, 892.9574, 896.3969, 908.9280, 951.5720, 996.5430, 1017.6157, 1017.6157, 1019.6299, 1033.6429, 1045.5127, 1045.5127, 1051.6522, 1051.6522, 1131.6493, 1133.6587, 1147.6777, 1151.6696, 1165.6809, 1165.6809, 1169.5313, 1171.6676, 1179.5499, 1185.6776, 1185.6776, 1189.6735, 1193.6417, 1203.6858, 1207.6569, 1211.6572, 1225.6708, 1300.7860, 1316.8134, 1334.8168, 1334.8168, 1440.5756, 1448.8479, 1448.8479, 1475.6846, 1522.7052, 1533.7407, 1538.7010, 1554.6993, 1581.7728, 1617.9867, 1617.9867, 1732.0165, 2211.0193, 2305.0740, 2321.0552, 2528.2617

Search Parameters

Type of search : **Sequence Query**
Enzyme : **Trypsin**
Fixed modifications : **Carbamidomethyl (C)**
Variable modifications : **Oxidation (M)**
Mass values : **Monoisotopic**

PDF-XChange Click to buy NOW! www.docu-track.com

PDF-XChange Click to buy NOW! www.docu-track.com

```
[illegible]otein Mass           : Unrestricted
[illegible]eptide Mass Tolerance : ± 100 ppm
Fragment Mass Tolerance: ± 0.3 Da
Max Missed Cleavages   : 1
Instrument type        : MALDI-TOF-TOF
   Query1 (813.4393,1+): <no title>
   Query2 (834.4629,1+): <no title>
   Query3 (838.4906,1+): <no title>
   Query4 (842.4641,1+): <no title>
   Query5 (846.4475,1+): <no title>
   Query6 (848.4780,1+): <no title>
   Query7 (850.0118,1+): <no title>
   Query8 (850.4896,1+): Locus:1..14.0.6
   Query9 (850.4896,1+): <no title>
  Query10 (853.4691,1+): <no title>
  Query11 (856.4822,1+): <no title>
  Query12 (864.4857,1+): <no title>
  Query13 (865.9818,1+): <no title>
  Query14 (866.4929,1+): <no title>
  Query15 (868.5015,1+): <no title>
  Query16 (868.5015,1+): Locus:1..14.0.11
  Query17 (870.4977,1+): <no title>
  Query18 (876.9884,1+): <no title>
  Query19 (882.5173,1+): Locus:1..14.0.1
  Query20 (882.5173,1+): <no title>
  Query21 (892.9574,1+): <no title>
  Query22 (892.9574,1+): Locus:1..14.0.10
  Query23 (896.3969,1+): <no title>
  Query24 (908.9280,1+): <no title>
  Query25 (951.5720,1+): <no title>
  Query26 (996.5430,1+): <no title>
 Query27 (1017.6157,1+): <no title>
 Query28 (1017.6157,1+): Locus:1..14.0.12
 Query29 (1019.6299,1+): <no title>
 Query30 (1033.6429,1+): <no title>
 Query31 (1045.5127,1+): <no title>
 Query32 (1045.5127,1+): Locus:1..14.0.8
 Query33 (1051.6522,1+): <no title>
 Query34 (1051.6522,1+): Locus:1..14.0.2
 Query35 (1065.6028,1+): <no title>
 Query36 (1131.6493,1+): <no title>
 Query37 (1133.6587,1+): <no title>
 Query38 (1147.6777,1+): <no title>
 Query39 (1151.6696,1+): <no title>
 Query40 (1165.6809,1+): <no title>
 Query41 (1165.6809,1+): Locus:1..14.0.3
 Query42 (1169.5313,1+): <no title>
 Query43 (1171.6676,1+): <no title>
 Query44 (1179.5499,1+): <no title>
 Query45 (1184.6520,1+): <no title>
 Query46 (1185.6776,1+): <no title>
 Query47 (1185.6776,1+): Locus:1..14.0.7
 Query48 (1189.6735,1+): <no title>
 Query49 (1193.6417,1+): <no title>
 Query50 (1203.6858,1+): <no title>
 Query51 (1207.6569,1+): <no title>
 Query52 (1211.6572,1+): <no title>
 Query53 (1225.6708,1+): <no title>
 Query54 (1279.7007,1+): <no title>
 Query55 (1300.7860,1+): <no title>
 Query56 (1316.8134,1+): <no title>
 Query57 (1334.8168,1+): Locus:1..14.0.4
 Query58 (1334.8168,1+): <no title>
 Query59 (1440.5756,1+): <no title>
 Query60 (1448.8479,1+): <no title>
 Query61 (1448.8479,1+): Locus:1..14.0.9
 Query62 (1475.6846,1+): <no title>
 Query63 (1522.7052,1+): <no title>
 Query64 (1533.7407,1+): <no title>
 Query65 (1538.7010,1+): <no title>
 Query66 (1554.6993,1+): <no title>
 Query67 (1581.7728,1+): <no title>
 Query68 (1617.9867,1+): Locus:1..14.0.5
 Query69 (1617.9867,1+): <no title>
 Query70 (1732.0165,1+): <no title>
 Query71 (1901.1586,1+): <no title>
 Query72 (2015.1949,1+): <no title>
 Query73 (2211.0193,1+): <no title>
 Query74 (2283.0847,1+): <no title>
 Query75 (2305.0740,1+): <no title>
 Query76 (2321.0552,1+): <no title>
 Query77 (2528.2617,1+): <no title>
```

Mascot: http://www.matrixscience.com/
