# Supplementary material for: Alpha-2-Macroglobulin as a New Promising Biomarker Improving the Diagnostic Sensitivity of Bovine Paratuberculosis
Source: Front Vet Sci. 2021 Mar 5;8:637716. doi: 10.3389/fvets.2021.637716 (PMC7973028; doi:10.3389/fvets.2021.637716)
Supplement: Supplementary Data 1 — Mascot search results and raw data files for LC-MS/MS analysis for the identification of specific blood proteins in cattle with Johne's disease. [file Data_Sheet_1.zip › mascot/8 Kallikrein G_2.pdf]

PDF-XChange Click to buy NOW! www.docu-track.com

MATRIX SCIENCE MASCOT Search Results

Protein View: A0A1R3UGP4

Kallikrein G OS=Bos taurus OX=9913 GN=KLNG PE=3 SV=1

Database:	Uniprot_bovine
Score:	66
Expect:	0.034
Nominal mass (M_r):	28249
Calculated pI:	9.07

Sequence similarity is available as an NCBI BLAST search of A0A1R3UGP4 against nr.

Search parameters

Enzyme:	Trypsin: cuts C-term side of KR unless next residue is P.
Fixed modifications:	Carbamidomethyl (C)
Variable modifications:	Oxidation (M)

Protein sequence coverage: 42%

Matched peptides shown in ***bold red***.

1 **MMTLQLIMFA LVTGHVGGET RIIK**GYECPP HSQPWQAALF QKTR**LLCGAT**
51 **LIAPR**WLLTA AHCRKPR**YVV HLGAHSLGRQ DGCEQTRTAT KSFPHPDFNN**
101 **SLPNK**DHR**ND IMLVK**MVTPA HLTWAVRPLT VSPRCVPAGA NCLISGWGTM
151 SSPQLHLPHT LR**CANVTIIK HR**ECEDAYPG NITDTMVCAS VRKEGKDSCQ
201 GDSGGPLVCN GSLQGIISWG QDPCAVSK**KP GVYTKVCKYV DWIQK**TMENN

Unformatted sequence string: 250 residues (for pasting into other applications).

Sort peptides by Residue Number Increasing Mass Decreasing Mass

Show predicted peptides also

Query	Start – End	Observed	Mr(expt)	Mr(calc)	ppm	M	Score	Peptide
75	1 – 21	2305.0740	2304.0667	2304.1793	-48.8	0		-.MMTLQLIMFALVTGHVGGETR.I
76	1 – 21	2321.0552	2320.0479	2320.1742	-54.4	0		-.MMTLQLIMFALVTGHVGGETR.I + Oxidation (M)
77	2 – 24	2528.2617	2527.2544	2527.4019	-58.3	1		M.MTLQLIMFALVTGHVGGETRIIK.G
45	45 – 55	1184.6520	1183.6447	1183.6747	-25.3	0		R.LLCGATLIAPR.W
74	68 – 87	2283.0847	2282.0774	2282.0975	-8.78	1		R.YVVHLGAHSLGRQDGCEQTR.T
72	88 – 105	2015.1949	2014.1876	2013.9908	97.7	1		R.TATKSFPHPDFNNSLPNK.D
6	109 – 115	848.4780	847.4707	847.4473	27.6	0		R.NDIMLVK.M + Oxidation (M)
52	163 – 172	1211.6572	1210.6499	1210.6604	-8.69	1		R.CANVTIIKHR.E
44	229 – 238	1179.5499	1178.5426	1178.6482	-89.5	1		K.KPGVYTKVCK.Y
25	239 – 245	951.5720	950.5647	950.4862	82.7	0		K.YVDWIQK.T

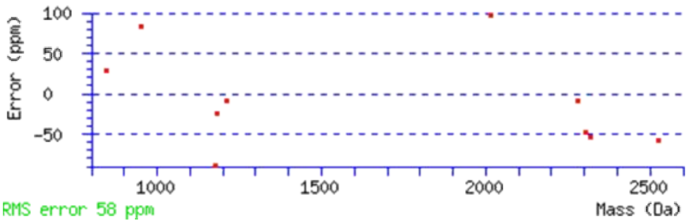


```
ID   A0A1R3UGP4_BOVIN        Unreviewed;        250 AA.
AC   A0A1R3UGP4;
DT   12-APR-2017, integrated into UniProtKB/TrEMBL.
DT   12-APR-2017, sequence version 1.
DT   11-DEC-2019, entry version 7.
DE   SubName: Full=Kallikrein G {ECO:0000313|EMBL:SFW93292.1};
GN   Name=KLNG {ECO:0000313|EMBL:SFW93292.1};
OS   Bos taurus (Bovine).
OC   Eukaryota; Metazoa; Chordata; Craniata; Vertebrata; Euteleostomi; Mammalia;
OC   Eutheria; Laurasiatheria; Artiodactyla; Ruminantia; Pecora; Bovidae;
```

PDF-XChange Click to buy NOW! www.docu-track.com

```
OC   Bovinae; Bos.
OX   NCBI_TaxID=9913 {ECO:0000313|EMBL:SFW93292.1};
RN   [1] {ECO:0000313|EMBL:SFW93292.1}
RP   NUCLEOTIDE SEQUENCE.
RX   PubMed=28224083; DOI=10.1016/j.ymgmr.2017.01.009;
RA   Premzl M.;
RT   "Comparative genomic analysis of eutherian kallikrein genes.";
RL   Mol. Genet. Metab. Rep. 10:96-99(2017).
RN   [2] {ECO:0000313|EMBL:SFW93292.1}
RP   NUCLEOTIDE SEQUENCE.
RX   DOI=10.1016/j.genrep.2019.100414;
RA   Premzl M.;
RT   "Eutherian third-party data gene collections.";
RL   Gene Rep 16:0-0(2019).
CC   -!- SIMILARITY: Belongs to the peptidase S1 family.
CC       {ECO:0000256|SAAS:SAAS00559343}.
CC   ---------------------------------------------------------------------------
CC   Copyrighted by the UniProt Consortium, see https://www.uniprot.org/terms
CC   Distributed under the Creative Commons Attribution (CC BY 4.0) License
CC   ---------------------------------------------------------------------------
DR   EMBL; LT631645; SFW93292.1; -; Genomic_DNA.
DR   RefSeq; XP_010813547.1; XM_010815245.2.
DR   RefSeq; XP_010822145.1; XM_010823843.2.
DR   GeneID; 526737; -.
DR   CTD; 11012; -.
DR   GO; GO:0004252; F:serine-type endopeptidase activity; IEA:InterPro.
DR   CDD; cd00190; Tryp_SPc; 1.
DR   InterPro; IPR009003; Peptidase_S1_PA.
DR   InterPro; IPR001314; Peptidase_S1A.
DR   InterPro; IPR001254; Trypsin_dom.
DR   InterPro; IPR018114; TRYPSIN_HIS.
DR   InterPro; IPR033116; TRYPSIN_SER.
DR   Pfam; PF00089; Trypsin; 1.
DR   PRINTS; PR00722; CHYMOTRYPSIN.
DR   SMART; SM00020; Tryp_SPc; 1.
DR   SUPFAM; SSF50494; SSF50494; 1.
DR   PROSITE; PS50240; TRYPSIN_DOM; 1.
DR   PROSITE; PS00134; TRYPSIN_HIS; 1.
DR   PROSITE; PS00135; TRYPSIN_SER; 1.
PE   3: Inferred from homology;
KW   Disulfide bond {ECO:0000256|SAAS:SAAS00037407};
KW   Hydrolase {ECO:0000256|RuleBase:RU363034};
KW   Protease {ECO:0000256|RuleBase:RU363034};
KW   Serine protease {ECO:0000256|RuleBase:RU363034};
KW   Signal {ECO:0000256|SAM:SignalP}.
FT   SIGNAL          1..18
FT                   /evidence="ECO:0000256|SAM:SignalP"
FT   CHAIN           19..250
FT                   /evidence="ECO:0000256|SAM:SignalP"
FT                   /id="PRO_5010232748"
FT   DOMAIN          22..248
FT                   /note="Peptidase S1"
FT                   /evidence="ECO:0000259|PROSITE:PS50240"
SQ   SEQUENCE   250 AA;  27526 MW;  FC0A1E17DA0325D1 CRC64;
     MMTLQLIMFA LVTGHVGGET RIIKGYECPP HSQPWQAALF QKTRLLCGAT LIAPRWLLTA
     AHCRKPRYVV HLGAHSLGRQ DGCEQTRTAT KSFPHPDFNN SLPNKDHRND IMLVKMVTPA
     HLTWAVRPLT VSPRCVPAGA NCLISGWGTM SSPQLHLPHT LRCANVTIIK HRECEDAYPG
     NITDTMVCAS VRKEGKDSCQ GDSGGPLVCN GSLQGIISWG QDPCAVSKKP GVYTKVCKYV
     DWIQKTMENN
//
```

Mascot: http://www.matrixscience.com/

PDF-XChange Click to buy NOW! www.docu-track.com
