# Supplementary material for: Alpha-2-Macroglobulin as a New Promising Biomarker Improving the Diagnostic Sensitivity of Bovine Paratuberculosis
Source: Front Vet Sci. 2021 Mar 5;8:637716. doi: 10.3389/fvets.2021.637716 (PMC7973028; doi:10.3389/fvets.2021.637716)
Supplement: Supplementary Data 1 — Mascot search results and raw data files for LC-MS/MS analysis for the identification of specific blood proteins in cattle with Johne's disease. [file Data_Sheet_1.zip › mascot/9 complement component C9 precursor_1.pdf]

PDF-XChange Click to buy NOW! www.docu-track.com
PDF-XChange Click to buy NOW! www.docu-track.com

MATRIX SCIENCE Mascot Search Results

User :
Email :
Search title : BAEDW\2020_3_5\2020_3_5\MSMS 14\N24
Database : Uniprot_bovine bovine_20200316 (134392 sequences; 76244912 residues)
Timestamp : 31 Mar 2020 at 01:09:09 GMT
Warning : A Peptide summary report will usually give a much clearer picture of MS/MS search results.
Top Score : 131 for A0A3Q1MU98, Complement component C9 OS=Bos taurus OX=9913 GN=C9 PE=1 SV=1

Mascot Score Histogram

Protein score is -10*Log(P), where P is the probability that the observed match is a random event.
Protein scores greater than 64 are significant (p<0.05).
Protein scores are derived from ions scores as a non-probabilistic basis for ranking protein hits.

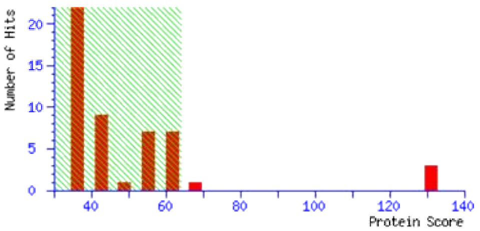

Protein Summary Report

Format As | Protein Summary (deprecated) | Help

Significance threshold p< 0.05 Max. number of hits 30

Preferred taxonomy All entries

Re-Search All | Search Unmatched

Index

	Accession	Mass	Score	Description
1.	A0A3Q1MU98	58618	131	Complement component C9 OS=Bos taurus OX=9913 GN=C9 PE=1 SV=1
2.	A0A4W2FBY7	63327	128	Complement C9 OS=Bos indicus x Bos taurus OX=30522 GN=C9 PE=4 SV=1
3.	Q3MHN2	63327	128	Complement component C9 OS=Bos taurus OX=9913 GN=C9 PE=2 SV=1
4.	Q2KJF1	54091	68	Alpha-1B-glycoprotein OS=Bos taurus OX=9913 GN=A1BG PE=1 SV=1
5.	A0A4W2H1C4	60879	64	Alpha-1-B glycoprotein OS=Bos indicus x Bos taurus OX=30522 GN=A1BG PE=4 SV=1
6.	A0A3Q1MJT2	62948	63	Alpha-1B-glycoprotein OS=Bos taurus OX=9913 GN=A1BG PE=1 SV=1
7.	A0A4W2EKQ3	63006	63	Alpha-1-B glycoprotein OS=Bos indicus x Bos taurus OX=30522 GN=A1BG PE=4 SV=1
8.	A0A4W2GYB4	54132	61	Alpha-1-B glycoprotein OS=Bos indicus x Bos taurus OX=30522 GN=A1BG PE=4 SV=1
9.	A0A4W2E798	54147	61	Alpha-1-B glycoprotein OS=Bos indicus x Bos taurus OX=30522 GN=A1BG PE=4 SV=1
10.	A0A4W2IFR5	54987	60	Alpha-1-B glycoprotein OS=Bos indicus x Bos taurus OX=30522 GN=A1BG PE=4 SV=1
11.	A0A4W2E8B7	55003	60	Alpha-1-B glycoprotein OS=Bos indicus x Bos taurus OX=30522 GN=A1BG PE=4 SV=1
12.	A0A3Q1LNN7	68022	57	Serum albumin OS=Bos taurus OX=9913 GN=ALB PE=4 SV=1
13.	A0A4W2DDL5	70207	56	Albumin OS=Bos indicus x Bos taurus OX=30522 GN=ALB PE=4 SV=1
14.	A0A4W2D8T3	76089	56	Albumin OS=Bos indicus x Bos taurus OX=30522 GN=ALB PE=4 SV=1
15.	A0A140T897	71274	56	Serum albumin OS=Bos taurus OX=9913 GN=ALB PE=4 SV=1
16.	A0A4W2EVR9	71453	56	Albumin OS=Bos indicus x Bos taurus OX=30522 GN=ALB PE=4 SV=1
17.	A0A4W2EW60	76020	55	Albumin OS=Bos indicus x Bos taurus OX=30522 GN=ALB PE=4 SV=1
18.	P02769	71244	55	Serum albumin OS=Bos taurus OX=9913 GN=ALB PE=1 SV=4
19.	C1J945	56189	48	Nonstructural protein 5A (Fragment) OS=Bovine viral diarrhea virus OX=11099 PE=4 SV=1
20.	A0A4W2EGF3	97654	46	Dynamin 3 OS=Bos indicus x Bos taurus OX=30522 GN=DNM3 PE=3 SV=1
21.	A0A3Q1MDN4	96034	45	Uncharacterized protein OS=Bos taurus OX=9913 GN=DNM3 PE=3 SV=1
22.	A0A4W2EI53	63308	44	Dynamin 3 OS=Bos indicus x Bos taurus OX=30522 GN=DNM3 PE=3 SV=1
23.	A0A4W2IJA6	101091	43	Family with sequence similarity 111 member B OS=Bos indicus x Bos taurus OX=30522 GN=FAM111B PE=4 SV=1
24.	A0A4W2ED96	52578	42	6-phosphofructo-2-kinase/fructose-2,6-biphosphatase 1 OS=Bos indicus x Bos taurus OX=30522 GN=PFKFB1 PE
25.	A0A3Q1LXR0	52578	42	6-phosphofructo-2-kinase/fructose-2,6-bisphosphatase 1 OS=Bos taurus OX=9913 GN=PFKFB1 PE=4 SV=1
26.	A0A4W2EAJ9	72734	40	Dynamin 3 OS=Bos indicus x Bos taurus OX=30522 GN=DNM3 PE=3 SV=1
27.	A0A4W2GW83	70119	40	Albumin OS=Bos indicus x Bos taurus OX=30522 GN=ALB PE=4 SV=1
28.	B0JYQ0	71244	40	ALB protein OS=Bos taurus OX=9913 GN=ALB PE=2 SV=1
29.	A0A4W2D9L2	98304	39	Dynamin 3 OS=Bos indicus x Bos taurus OX=30522 GN=DNM3 PE=3 SV=1
30.	A0A4W2IKT9	75992	39	Albumin OS=Bos indicus x Bos taurus OX=30522 GN=ALB PE=4 SV=1

Results List

1. A0A3Q1MU98 **Mass:** 58618 **Score:** 131 **Expect:** 1.1e-008 **Matches:** 12
Complement component C9 OS=Bos taurus OX=9913 GN=C9 PE=1 SV=1

Observed	Mr(expt)	Mr(calc)	ppm	Start	End	Miss	Ions	Peptide
842.4471	841.4398	841.4480	-9.70	447 -	453	1	---	K.MKDAHLK.K
868.4844	867.4771	867.4702	8.03	287 -	294	0	---	R.LLSSYSAK.Q
1156.5005	1155.4932	1155.5706	-67.00	33 -	42	0	---	R.QGTPLPIDCR.M
1175.5602	1174.5529	1174.6169	-54.42	295 -	303	1	---	K.QEKMFLHVK.G + Oxidation (M)
1267.5582	1266.5509	1266.6357	-66.92	64 -	74	0	---	R.SIEIFGQFNGR.K
1267.5582	1266.5510	1266.6357	-66.89	64 -	74	0	29	R.SIEIFGQFNGR.K
1387.5682	1386.5609	1386.6568	-69.15	182 -	192	0	---	R.VWDGNTLTYYR.R
1387.5682	1386.5610	1386.6568	-69.12	182 -	192	0	18	R.VWDGNTLTYYR.R
1455.6167	1454.6094	1454.7041	-65.10	460 -	471	0	---	R.AIEDYINEFSVR.K
1455.6167	1454.6094	1454.7041	-65.10	460 -	471	0	57	R.AIEDYINEFSVR.K
1550.6960	1549.6887	1549.7889	-64.62	193 -	205	0	---	R.RPWNVASLTYDTK.A
1550.6960	1549.6888	1549.7889	-64.60	193 -	205	0	---	R.RPWNVASLTYDTK.A

No match to: 805.4406, 848.4654, 849.9925, 850.4734, 853.4557, 864.4832, 865.9639, 870.4808, 876.9719, 882.5003, 882.5003, 892.9417, 908.9123, 919.3989, 927.4218, 951.5530, 953.4604, 996.5237, 998.4718, 1017.5948, 1019.6071, 1033.6263, 1045.4912, 1051.6328, 1051.6328, 1065.5367, 1131.6298, 1133.6357, 1136.6206, 1139.4833, 1142.5555, 1142.5555, 1151.6481, 1165.6569, 1165.6569, 1184.6132, 1189.4962, 1247.6283, 1268.5472, 1287.5924, 1300.7646, 1305.5656, 1311.6147, 1334.7938, 1334.7938,

PDF-XChange Click to buy NOW! www.docu-track.com
PDF-XChange Click to buy NOW! www.docu-track.com

1396.6301, 1439.7159, 1448.8251, 1475.6594, 1479.6964, 1479.6964, 1498.6063, 1513.6995, 1513.6995, 1557.7151, 1567.6414, 1567.6414, 1617.9590, 1647.7349, 1724.7261, 1731.9867, 1901.1331, 2044.9095, 2210.9834, 2283.0552

2. A0A4W2FBY7 **Mass:** 63327 **Score:** 128 **Expect:** 2.1e-008 **Matches:** 12

Complement C9 OS=Bos indicus x Bos taurus OX=30522 GN=C9 PE=4 SV=1

Observed	Mr(expt)	Mr(calc)	ppm	Start	End	Miss	Ions	Peptide
842.4471	841.4398	841.4480	-9.70	489	495	1	---	K.MKDAHLK.K
868.4844	867.4771	867.4702	8.03	287	294	0	---	R.LLSSYSAK.Q
1156.5005	1155.4932	1155.5706	-67.00	33	42	0	---	R.QGTPLPIDCR.M
1175.5602	1174.5529	1174.6169	-54.42	295	303	1	---	K.QEKMFLHVK.G + Oxidation (M)
1267.5582	1266.5509	1266.6357	-66.92	64	74	0	---	R.SIEIFGQFNGR.K
1267.5582	1266.5510	1266.6357	-66.89	64	74	0	29	R.SIEIFGQFNGR.K
1387.5682	1386.5609	1386.6568	-69.15	182	192	0	---	R.VWDGNTLTYYR.R
1387.5682	1386.5610	1386.6568	-69.12	182	192	0	18	R.VWDGNTLTYYR.R
1455.6167	1454.6094	1454.7041	-65.10	502	513	0	---	R.AIEDYINEFSVR.K
1455.6167	1454.6094	1454.7041	-65.10	502	513	0	57	R.AIEDYINEFSVR.K
1550.6960	1549.6887	1549.7889	-64.62	193	205	0	---	R.RPWNVASLTYDTK.A
1550.6960	1549.6888	1549.7889	-64.60	193	205	0	---	R.RPWNVASLTYDTK.A

No match to: 805.4406, 848.4654, 849.9925, 850.4734, 853.4557, 864.4832, 865.9639, 870.4808, 876.9719, 882.5003, 882.5003, 892.9417, 908.9123, 919.3989, 927.4218, 951.5530, 953.4604, 996.5237, 998.4718, 1017.5948, 1019.6071, 1033.6263, 1045.4912, 1051.6328, 1051.6328, 1065.5367, 1131.6298, 1133.6357, 1136.6206, 1139.4833, 1142.5555, 1142.5555, 1151.6481, 1165.6569, 1165.6569, 1184.6132, 1189.4962, 1247.6283, 1268.5472, 1287.5924, 1300.7646, 1305.5656, 1311.6147, 1334.7938, 1334.7938, 1396.6301, 1439.7159, 1448.8251, 1475.6594, 1479.6964, 1479.6964, 1498.6063, 1513.6995, 1513.6995, 1557.7151, 1567.6414, 1567.6414, 1617.9590, 1647.7349, 1724.7261, 1731.9867, 1901.1331, 2044.9095, 2210.9834, 2283.0552

3. Q3MHN2 **Mass:** 63327 **Score:** 128 **Expect:** 2.1e-008 **Matches:** 12

Complement component C9 OS=Bos taurus OX=9913 GN=C9 PE=2 SV=1

Observed	Mr(expt)	Mr(calc)	ppm	Start	End	Miss	Ions	Peptide
842.4471	841.4398	841.4480	-9.70	489	495	1	---	K.MKDAHLK.K
868.4844	867.4771	867.4702	8.03	287	294	0	---	R.LLSSYSAK.Q
1156.5005	1155.4932	1155.5706	-67.00	33	42	0	---	R.QGTPLPIDCR.M
1175.5602	1174.5529	1174.6169	-54.42	295	303	1	---	K.QEKMFLHVK.G + Oxidation (M)
1267.5582	1266.5509	1266.6357	-66.92	64	74	0	---	R.SIEIFGQFNGR.K
1267.5582	1266.5510	1266.6357	-66.89	64	74	0	29	R.SIEIFGQFNGR.K
1387.5682	1386.5609	1386.6568	-69.15	182	192	0	---	R.VWDGNTLTYYR.R
1387.5682	1386.5610	1386.6568	-69.12	182	192	0	18	R.VWDGNTLTYYR.R
1455.6167	1454.6094	1454.7041	-65.10	502	513	0	---	R.AIEDYINEFSVR.K
1455.6167	1454.6094	1454.7041	-65.10	502	513	0	57	R.AIEDYINEFSVR.K
1550.6960	1549.6887	1549.7889	-64.62	193	205	0	---	R.RPWNVASLTYDTK.A
1550.6960	1549.6888	1549.7889	-64.60	193	205	0	---	R.RPWNVASLTYDTK.A

No match to: 805.4406, 848.4654, 849.9925, 850.4734, 853.4557, 864.4832, 865.9639, 870.4808, 876.9719, 882.5003, 882.5003, 892.9417, 908.9123, 919.3989, 927.4218, 951.5530, 953.4604, 996.5237, 998.4718, 1017.5948, 1019.6071, 1033.6263, 1045.4912, 1051.6328, 1051.6328, 1065.5367, 1131.6298, 1133.6357, 1136.6206, 1139.4833, 1142.5555, 1142.5555, 1151.6481, 1165.6569, 1165.6569, 1184.6132, 1189.4962, 1247.6283, 1268.5472, 1287.5924, 1300.7646, 1305.5656, 1311.6147, 1334.7938, 1334.7938, 1396.6301, 1439.7159, 1448.8251, 1475.6594, 1479.6964, 1479.6964, 1498.6063, 1513.6995, 1513.6995, 1557.7151, 1567.6414, 1567.6414, 1617.9590, 1647.7349, 1724.7261, 1731.9867, 1901.1331, 2044.9095, 2210.9834, 2283.0552

4. Q2KJF1 **Mass:** 54091 **Score:** 68 **Expect:** 0.022 **Matches:** 11

Alpha-1B-glycoprotein OS=Bos taurus OX=9913 GN=A1BG PE=1 SV=1

Observed	Mr(expt)	Mr(calc)	ppm	Start	End	Miss	Ions	Peptide
805.4406	804.4333	804.4858	-65.20	148	154	0	---	R.GVTFLLR.L
919.3989	918.3916	918.4559	-69.98	288	295	0	---	R.SELAAWSR.D
998.4718	997.4645	997.5192	-54.85	249	257	0	---	R.GAEEQLVPR.A
1142.5555	1141.5482	1141.6244	-66.72	415	425	0	---	R.ALWTGALTPGR.D
1142.5555	1141.5483	1141.6244	-66.68	415	425	0	---	R.ALWTGALTPGR.D
1175.5602	1174.5529	1174.6346	-69.57	81	91	0	---	R.FPLGPVTSTTR.G
1247.6283	1246.6210	1246.7146	-75.06	404	414	0	---	R.VDGPLPRPQLR.A
1498.6063	1497.5990	1497.6882	-59.53	269	283	0	---	R.LSALAAGDGSGYTCR.Y
1513.6995	1512.6922	1512.7936	-67.06	357	370	0	27	R.VLSPAGPEAQFELR.G
1513.6995	1512.6922	1512.7936	-67.03	357	370	0	---	R.VLSPAGPEAQFELR.G
1647.7349	1646.7276	1646.8338	-64.46	431	444	0	---	R.CEAEVPDVSFLLLR.A

No match to: 842.4471, 848.4654, 849.9925, 850.4734, 853.4557, 864.4832, 865.9639, 868.4844, 870.4808, 876.9719, 882.5003, 882.5003, 892.9417, 908.9123, 927.4218, 951.5530, 953.4604, 996.5237, 1017.5948, 1019.6071, 1033.6263, 1045.4912, 1051.6328, 1051.6328, 1065.5367, 1131.6298, 1133.6357, 1136.6206, 1139.4833, 1151.6481, 1156.5005, 1165.6569, 1165.6569, 1184.6132, 1189.4962, 1267.5582, 1267.5582, 1268.5472, 1287.5924, 1300.7646, 1305.5656, 1311.6147, 1334.7938, 1334.7938, 1387.5682, 1387.5682, 1396.6301, 1439.7159, 1448.8251, 1455.6167, 1455.6167, 1475.6594, 1479.6964, 1479.6964, 1550.6960, 1550.6960, 1557.7151, 1567.6414, 1567.6414, 1617.9590, 1724.7261, 1731.9867, 1901.1331, 2044.9095, 2210.9834, 2283.0552

5. A0A4W2H1C4 **Mass:** 60879 **Score:** 64 **Expect:** 0.05 **Matches:** 11

Alpha-1-B glycoprotein OS=Bos indicus x Bos taurus OX=30522 GN=A1BG PE=4 SV=1

Observed	Mr(expt)	Mr(calc)	ppm	Start	End	Miss	Ions	Peptide
805.4406	804.4333	804.4858	-65.20	148	154	0	---	R.GVTFLLR.L
919.3989	918.3916	918.4559	-69.98	288	295	0	---	R.SELAAWSR.D
998.4718	997.4645	997.5192	-54.85	249	257	0	---	R.GAEEQLVPR.A
1142.5555	1141.5482	1141.6244	-66.72	415	425	0	---	R.ALWTGALTPGR.D
1142.5555	1141.5483	1141.6244	-66.68	415	425	0	---	R.ALWTGALTPGR.D
1175.5602	1174.5529	1174.6346	-69.57	81	91	0	---	R.FPLGPVTSTTR.G
1247.6283	1246.6210	1246.7146	-75.06	404	414	0	---	R.VDGPLPRPQLR.A
1498.6063	1497.5990	1497.6882	-59.53	269	283	0	---	R.LSALAAGDGSGYTCR.Y
1513.6995	1512.6922	1512.7936	-67.06	357	370	0	27	R.VLSPAGPEAQFELR.G
1513.6995	1512.6922	1512.7936	-67.03	357	370	0	---	R.VLSPAGPEAQFELR.G
1647.7349	1646.7276	1646.8338	-64.46	431	444	0	---	R.CEAEVPDVSFLLLR.A

No match to: 842.4471, 848.4654, 849.9925, 850.4734, 853.4557, 864.4832, 865.9639, 868.4844, 870.4808, 876.9719, 882.5003, 882.5003, 892.9417, 908.9123, 927.4218, 951.5530, 953.4604, 996.5237, 1017.5948, 1019.6071, 1033.6263, 1045.4912, 1051.6328, 1051.6328, 1065.5367, 1131.6298, 1133.6357, 1136.6206, 1139.4833, 1151.6481, 1156.5005, 1165.6569, 1165.6569, 1184.6132, 1189.4962, 1267.5582, 1267.5582, 1268.5472, 1287.5924, 1300.7646, 1305.5656, 1311.6147, 1334.7938, 1334.7938, 1387.5682, 1387.5682, 1396.6301, 1439.7159, 1448.8251, 1455.6167, 1455.6167, 1475.6594, 1479.6964, 1479.6964, 1550.6960, 1550.6960, 1557.7151, 1567.6414, 1567.6414, 1617.9590, 1724.7261, 1731.9867, 1901.1331, 2044.9095, 2210.9834, 2283.0552

6. A0A3Q1MJT2 **Mass:** 62948 **Score:** 63 **Expect:** 0.063 **Matches:** 11

Alpha-1B-glycoprotein OS=Bos taurus OX=9913 GN=A1BG PE=1 SV=1

Observed	Mr(expt)	Mr(calc)	ppm	Start	End	Miss	Ions	Peptide
805.4406	804.4333	804.4858	-65.20	148	154	0	---	R.GVTFLLR.L
919.3989	918.3916	918.4559	-69.98	288	295	0	---	R.SELAAWSR.D
998.4718	997.4645	997.5192	-54.85	249	257	0	---	R.GAEEQLVPR.A

PDF-XChange Click to buy NOW! www.docu-track.com

1142.5555	1141.5482	1141.6244	-66.72	415	425	0	---	R.ALWTGALTPGR.D
1142.5555	1141.5483	1141.6244	-66.68	415	425	0	---	R.ALWTGALTPGR.D
1175.5602	1174.5529	1174.6346	-69.57	81	91	0	---	R.FPLGPVTSTTR.G
1247.6283	1246.6210	1246.7146	-75.06	404	414	0	---	R.VDGPLPRPQLR.A
1498.6063	1497.5990	1497.6882	-59.53	269	283	0	---	R.LSALAAGDGSGYTCR.Y
1513.6995	1512.6922	1512.7936	-67.06	357	370	0	27	R.VLSPAGPEAQFELR.G
1513.6995	1512.6922	1512.7936	-67.03	357	370	0	---	R.VLSPAGPEAQFELR.G
1647.7349	1646.7276	1646.8338	-64.46	431	444	0	---	R.CEAEVPDVSFLLLR.A

No match to: 842.4471, 848.4654, 849.9925, 850.4734, 853.4557, 864.4832, 865.9639, 868.4844, 870.4808, 876.9719, 882.5003, 882.5003, 892.9417, 908.9123, 927.4218, 951.5530, 953.4604, 996.5237, 1017.5948, 1019.6071, 1033.6263, 1045.4912, 1051.6328, 1051.6328, 1065.5367, 1131.6298, 1133.6357, 1136.6206, 1151.6481, 1156.5005, 1165.6569, 1165.6569, 1184.6132, 1189.4962, 1267.5582, 1267.5582, 1268.5472, 1287.5924, 1300.7646, 1305.5656, 1311.6147, 1334.7938, 1334.7938, 1387.5682, 1387.5682, 1396.6301, 1439.7159, 1448.8251, 1455.6167, 1455.6167, 1475.6594, 1479.6964, 1479.6964, 1550.6960, 1550.6960, 1557.7151, 1567.6414, 1567.6414, 1617.9590, 1724.7261, 1731.9867, 1901.1331, 2044.9095, 2210.9834, 2283.0552

7. A0A4W2EKQ3 **Mass:** 63006 **Score:** 63 **Expect:** 0.063 **Matches:** 11

Alpha-1-B glycoprotein OS=Bos indicus x Bos taurus OX=30522 GN=A1BG PE=4 SV=1

Observed	Mr(expt)	Mr(calc)	ppm	Start	End	Miss	Ions	Peptide
805.4406	804.4333	804.4858	-65.20	148	154	0	---	R.GVTFLLR.L
919.3989	918.3916	918.4559	-69.98	288	295	0	---	R.SELAAWSR.D
998.4718	997.4645	997.5192	-54.85	249	257	0	---	R.GAEEQLVPR.A
1142.5555	1141.5482	1141.6244	-66.72	415	425	0	---	R.ALWTGALTPGR.D
1142.5555	1141.5483	1141.6244	-66.68	415	425	0	---	R.ALWTGALTPGR.D
1175.5602	1174.5529	1174.6346	-69.57	81	91	0	---	R.FPLGPVTSTTR.G
1247.6283	1246.6210	1246.7146	-75.06	404	414	0	---	R.VDGPLPRPQLR.A
1498.6063	1497.5990	1497.6882	-59.53	269	283	0	---	R.LSALAAGDGSGYTCR.Y
1513.6995	1512.6922	1512.7936	-67.06	357	370	0	27	R.VLSPAGPEAQFELR.G
1513.6995	1512.6922	1512.7936	-67.03	357	370	0	---	R.VLSPAGPEAQFELR.G
1647.7349	1646.7276	1646.8338	-64.46	431	444	0	---	R.CEAEVPDVSFLLLR.A

No match to: 842.4471, 848.4654, 849.9925, 850.4734, 853.4557, 864.4832, 865.9639, 868.4844, 870.4808, 876.9719, 882.5003, 882.5003, 892.9417, 908.9123, 927.4218, 951.5530, 953.4604, 996.5237, 1017.5948, 1019.6071, 1033.6263, 1045.4912, 1051.6328, 1051.6328, 1065.5367, 1131.6298, 1133.6357, 1136.6206, 1139.4833, 1151.6481, 1156.5005, 1165.6569, 1165.6569, 1184.6132, 1189.4962, 1267.5582, 1267.5582, 1268.5472, 1287.5924, 1300.7646, 1305.5656, 1311.6147, 1334.7938, 1334.7938, 1387.5682, 1387.5682, 1396.6301, 1439.7159, 1448.8251, 1455.6167, 1455.6167, 1475.6594, 1479.6964, 1479.6964, 1550.6960, 1550.6960, 1557.7151, 1567.6414, 1567.6414, 1617.9590, 1724.7261, 1731.9867, 1901.1331, 2044.9095, 2210.9834, 2283.0552

8. A0A4W2GYB4 **Mass:** 54132 **Score:** 61 **Expect:** 0.11 **Matches:** 10

Alpha-1-B glycoprotein OS=Bos indicus x Bos taurus OX=30522 GN=A1BG PE=4 SV=1

Observed	Mr(expt)	Mr(calc)	ppm	Start	End	Miss	Ions	Peptide
805.4406	804.4333	804.4858	-65.20	142	148	0	---	R.GVTFLLR.L
919.3989	918.3916	918.4559	-69.98	282	289	0	---	R.SELAAWSR.D
998.4718	997.4645	997.5192	-54.85	243	251	0	---	R.GAEEQLVPR.A
1142.5555	1141.5482	1141.6244	-66.72	417	427	0	---	R.ALWTGALTPGR.D
1142.5555	1141.5483	1141.6244	-66.68	417	427	0	---	R.ALWTGALTPGR.D
1175.5602	1174.5529	1174.6346	-69.57	75	85	0	---	R.FPLGPVTSTTR.G
1498.6063	1497.5990	1497.6882	-59.53	263	277	0	---	R.LSALAAGDGSGYTCR.Y
1513.6995	1512.6922	1512.7936	-67.06	351	364	0	27	R.VLSPAGPEAQFELR.G
1513.6995	1512.6922	1512.7936	-67.03	351	364	0	---	R.VLSPAGPEAQFELR.G
1647.7349	1646.7276	1646.8338	-64.46	433	446	0	---	R.CEAEVPDVSFLLLR.A

No match to: 842.4471, 848.4654, 849.9925, 850.4734, 853.4557, 864.4832, 865.9639, 868.4844, 870.4808, 876.9719, 882.5003, 882.5003, 892.9417, 908.9123, 927.4218, 951.5530, 953.4604, 996.5237, 1017.5948, 1019.6071, 1033.6263, 1045.4912, 1051.6328, 1051.6328, 1065.5367, 1131.6298, 1133.6357, 1136.6206, 1139.4833, 1151.6481, 1156.5005, 1165.6569, 1165.6569, 1184.6132, 1189.4962, 1247.6283, 1267.5582, 1267.5582, 1268.5472, 1287.5924, 1300.7646, 1305.5656, 1311.6147, 1334.7938, 1334.7938, 1387.5682, 1387.5682, 1396.6301, 1439.7159, 1448.8251, 1455.6167, 1455.6167, 1475.6594, 1479.6964, 1479.6964, 1550.6960, 1550.6960, 1557.7151, 1567.6414, 1567.6414, 1617.9590, 1724.7261, 1731.9867, 1901.1331, 2044.9095, 2210.9834, 2283.0552

9. A0A4W2E798 **Mass:** 54147 **Score:** 61 **Expect:** 0.11 **Matches:** 10

Alpha-1-B glycoprotein OS=Bos indicus x Bos taurus OX=30522 GN=A1BG PE=4 SV=1

Observed	Mr(expt)	Mr(calc)	ppm	Start	End	Miss	Ions	Peptide
805.4406	804.4333	804.4858	-65.20	142	148	0	---	R.GVTFLLR.L
919.3989	918.3916	918.4559	-69.98	282	289	0	---	R.SELAAWSR.D
998.4718	997.4645	997.5192	-54.85	243	251	0	---	R.GAEEQLVPR.A
1142.5555	1141.5482	1141.6244	-66.72	417	427	0	---	R.ALWTGALTPGR.D
1142.5555	1141.5483	1141.6244	-66.68	417	427	0	---	R.ALWTGALTPGR.D
1175.5602	1174.5529	1174.6346	-69.57	75	85	0	---	R.FPLGPVTSTTR.G
1498.6063	1497.5990	1497.6882	-59.53	263	277	0	---	R.LSALAAGDGSGYTCR.Y
1513.6995	1512.6922	1512.7936	-67.06	351	364	0	27	R.VLSPAGPEAQFELR.G
1513.6995	1512.6922	1512.7936	-67.03	351	364	0	---	R.VLSPAGPEAQFELR.G
1647.7349	1646.7276	1646.8338	-64.46	433	446	0	---	R.CEAEVPDVSFLLLR.A

No match to: 842.4471, 848.4654, 849.9925, 850.4734, 853.4557, 864.4832, 865.9639, 868.4844, 870.4808, 876.9719, 882.5003, 882.5003, 892.9417, 908.9123, 927.4218, 951.5530, 953.4604, 996.5237, 1017.5948, 1019.6071, 1033.6263, 1045.4912, 1051.6328, 1051.6328, 1065.5367, 1131.6298, 1133.6357, 1136.6206, 1139.4833, 1151.6481, 1156.5005, 1165.6569, 1165.6569, 1184.6132, 1189.4962, 1247.6283, 1267.5582, 1267.5582, 1268.5472, 1287.5924, 1300.7646, 1305.5656, 1311.6147, 1334.7938, 1334.7938, 1387.5682, 1387.5682, 1396.6301, 1439.7159, 1448.8251, 1455.6167, 1455.6167, 1475.6594, 1479.6964, 1479.6964, 1550.6960, 1550.6960, 1557.7151, 1567.6414, 1567.6414, 1617.9590, 1724.7261, 1731.9867, 1901.1331, 2044.9095, 2210.9834, 2283.0552

10. A0A4W2IFR5 **Mass:** 54987 **Score:** 60 **Expect:** 0.12 **Matches:** 10

Alpha-1-B glycoprotein OS=Bos indicus x Bos taurus OX=30522 GN=A1BG PE=4 SV=1

Observed	Mr(expt)	Mr(calc)	ppm	Start	End	Miss	Ions	Peptide
805.4406	804.4333	804.4858	-65.20	148	154	0	---	R.GVTFLLR.L
919.3989	918.3916	918.4559	-69.98	288	295	0	---	R.SELAAWSR.D
998.4718	997.4645	997.5192	-54.85	249	257	0	---	R.GAEEQLVPR.A
1142.5555	1141.5482	1141.6244	-66.72	423	433	0	---	R.ALWTGALTPGR.D
1142.5555	1141.5483	1141.6244	-66.68	423	433	0	---	R.ALWTGALTPGR.D
1175.5602	1174.5529	1174.6346	-69.57	81	91	0	---	R.FPLGPVTSTTR.G
1498.6063	1497.5990	1497.6882	-59.53	269	283	0	---	R.LSALAAGDGSGYTCR.Y
1513.6995	1512.6922	1512.7936	-67.06	357	370	0	27	R.VLSPAGPEAQFELR.G
1513.6995	1512.6922	1512.7936	-67.03	357	370	0	---	R.VLSPAGPEAQFELR.G
1647.7349	1646.7276	1646.8338	-64.46	439	452	0	---	R.CEAEVPDVSFLLLR.A

No match to: 842.4471, 848.4654, 849.9925, 850.4734, 853.4557, 864.4832, 865.9639, 868.4844, 870.4808, 876.9719, 882.5003, 882.5003, 892.9417, 908.9123, 927.4218, 951.5530, 953.4604, 996.5237, 1017.5948, 1019.6071, 1033.6263, 1045.4912, 1051.6328, 1051.6328, 1065.5367, 1131.6298, 1133.6357, 1136.6206, 1139.4833, 1151.6481, 1156.5005, 1165.6569, 1165.6569, 1184.6132, 1189.4962, 1247.6283, 1267.5582, 1267.5582, 1268.5472, 1287.5924, 1300.7646, 1305.5656, 1311.6147, 1334.7938, 1334.7938, 1387.5682, 1387.5682, 1396.6301, 1439.7159, 1448.8251, 1455.6167, 1455.6167, 1475.6594, 1479.6964, 1479.6964, 1550.6960, 1550.6960, 1557.7151, 1567.6414, 1567.6414, 1617.9590, 1724.7261, 1731.9867, 1901.1331, 2044.9095, 2210.9834, 2283.0552

PDF-XChange Click to buy NOW! www.docu-track.com

[illegible]1. A0A4W2E8B7 **Mass:** 55003 **Score:** 60 **Expect:** 0.12 **Matches:** 10
Alpha-1-B glycoprotein OS=Bos indicus x Bos taurus OX=30522 GN=A1BG PE=4 SV=1

Observed	Mr(expt)	Mr(calc)	ppm	Start	End	Miss	Ions	Peptide
805.4406	804.4333	804.4858	-65.20	148	154	0	---	R.GVTFLLR.L
919.3989	918.3916	918.4559	-69.98	288	295	0	---	R.SELAAWSR.D
998.4718	997.4645	997.5192	-54.85	249	257	0	---	R.GAEEQLVPR.A
1142.5555	1141.5482	1141.6244	-66.72	423	433	0	---	R.ALWTGALTPGR.D
1142.5555	1141.5483	1141.6244	-66.68	423	433	0	---	R.ALWTGALTPGR.D
1175.5602	1174.5529	1174.6346	-69.57	81	91	0	---	R.FPLGPVTSTTR.G
1498.6063	1497.5990	1497.6882	-59.53	269	283	0	---	R.LSALAAGDGSGYTCR.Y
1513.6995	1512.6922	1512.7936	-67.06	357	370	0	27	R.VLSPAGPEAQFELR.G
1513.6995	1512.6922	1512.7936	-67.03	357	370	0	---	R.VLSPAGPEAQFELR.G
1647.7349	1646.7276	1646.8338	-64.46	439	452	0	---	R.CEAEVPDVSFLLLR.A

No match to: 842.4471, 848.4654, 849.9925, 850.4734, 853.4557, 864.4832, 865.9639, 868.4844, 870.4808, 876.9719, 882.5003, 882.5003, 892.9417, 908.9123, 927.4218, 951.5530, 953.4604, 996.5237, 1017.5948, 1019.6071, 1033.6263, 1045.4912, 1051.6328, 1051.6328, 1065.5367, 1131.6298, 1133.6357, 1136.6206, 1139.4833, 1151.6481, 1156.5005, 1165.6569, 1165.6569, 1184.6132, 1189.4962, 1247.6283, 1267.5582, 1267.5582, 1268.5472, 1287.5924, 1300.7646, 1305.5656, 1311.6147, 1334.7938, 1334.7938, 1387.5682, 1387.5682, 1396.6301, 1439.7159, 1448.8251, 1455.6167, 1455.6167, 1475.6594, 1479.6964, 1479.6964, 1550.6960, 1550.6960, 1557.7151, 1567.6414, 1567.6414, 1617.9590, 1724.7261, 1731.9867, 1901.1331, 2044.9095, 2210.9834, 2283.0552

12. A0A3Q1LNN7 **Mass:** 68022 **Score:** 57 **Expect:** 0.25 **Matches:** 9
Serum albumin OS=Bos taurus OX=9913 GN=ALB PE=4 SV=1

Observed	Mr(expt)	Mr(calc)	ppm	Start	End	Miss	Ions	Peptide
927.4218	926.4145	926.4861	-77.29	161	167	0	---	K.YLYEIAR.R
1017.5948	1016.5875	1016.5727	14.6	212	220	1	---	K.VLTSSARQR.L
1439.7159	1438.7086	1438.8045	-66.61	360	371	1	---	R.RHPEYAVSVLLR.L
1479.6964	1478.6891	1478.7881	-66.96	421	433	0	---	K.LGEYGFQNALIVR.Y
1479.6964	1478.6891	1478.7881	-66.96	421	433	0	10	K.LGEYGFQNALIVR.Y
1567.6414	1566.6341	1566.7354	-64.69	347	359	0	27	K.DAFLGSFLYEYSR.R
1567.6414	1566.6341	1566.7354	-64.67	347	359	0	---	K.DAFLGSFLYEYSR.R
1724.7261	1723.7188	1723.8273	-62.93	469	482	0	---	R.MPCTEDYLSLILNR.L
2044.9095	2043.9022	2044.0206	-57.93	168	183	1	---	R.RHPYFYAPELLYYANK.Y

No match to: 805.4406, 842.4471, 848.4654, 849.9925, 850.4734, 853.4557, 864.4832, 865.9639, 868.4844, 870.4808, 876.9719, 882.5003, 882.5003, 892.9417, 908.9123, 919.3989, 951.5530, 953.4604, 996.5237, 998.4718, 1019.6071, 1033.6263, 1045.4912, 1051.6328, 1051.6328, 1065.5367, 1131.6298, 1133.6357, 1136.6206, 1139.4833, 1142.5555, 1142.5555, 1151.6481, 1156.5005, 1165.6569, 1165.6569, 1175.5602, 1184.6132, 1189.4962, 1247.6283, 1267.5582, 1267.5582, 1268.5472, 1287.5924, 1300.7646, 1305.5656, 1311.6147, 1334.7938, 1334.7938, 1387.5682, 1387.5682, 1396.6301, 1448.8251, 1455.6167, 1455.6167, 1475.6594, 1498.6063, 1513.6995, 1513.6995, 1550.6960, 1550.6960, 1557.7151, 1617.9590, 1647.7349, 1731.9867, 1901.1331, 2210.9834, 2283.0552

13. A0A4W2DDL5 **Mass:** 70207 **Score:** 56 **Expect:** 0.31 **Matches:** 9
Albumin OS=Bos indicus x Bos taurus OX=30522 GN=ALB PE=4 SV=1

Observed	Mr(expt)	Mr(calc)	ppm	Start	End	Miss	Ions	Peptide
927.4218	926.4145	926.4861	-77.29	152	158	0	---	K.YLYEIAR.R
1017.5948	1016.5875	1016.5727	14.6	203	211	1	---	K.VLTSSARQR.L
1439.7159	1438.7086	1438.8045	-66.61	351	362	1	---	R.RHPEYAVSVLLR.L
1479.6964	1478.6891	1478.7881	-66.96	412	424	0	---	K.LGEYGFQNALIVR.Y
1479.6964	1478.6891	1478.7881	-66.96	412	424	0	10	K.LGEYGFQNALIVR.Y
1567.6414	1566.6341	1566.7354	-64.69	338	350	0	27	K.DAFLGSFLYEYSR.R
1567.6414	1566.6341	1566.7354	-64.67	338	350	0	---	K.DAFLGSFLYEYSR.R
1724.7261	1723.7188	1723.8273	-62.93	460	473	0	---	R.MPCTEDYLSLILNR.L
2044.9095	2043.9022	2044.0206	-57.93	159	174	1	---	R.RHPYFYAPELLYYANK.Y

No match to: 805.4406, 842.4471, 848.4654, 849.9925, 850.4734, 853.4557, 864.4832, 865.9639, 868.4844, 870.4808, 876.9719, 882.5003, 882.5003, 892.9417, 908.9123, 919.3989, 951.5530, 953.4604, 996.5237, 998.4718, 1019.6071, 1033.6263, 1045.4912, 1051.6328, 1051.6328, 1065.5367, 1131.6298, 1133.6357, 1136.6206, 1139.4833, 1142.5555, 1142.5555, 1151.6481, 1156.5005, 1165.6569, 1165.6569, 1175.5602, 1184.6132, 1189.4962, 1247.6283, 1267.5582, 1267.5582, 1268.5472, 1287.5924, 1300.7646, 1305.5656, 1311.6147, 1334.7938, 1334.7938, 1387.5682, 1387.5682, 1396.6301, 1448.8251, 1455.6167, 1455.6167, 1475.6594, 1498.6063, 1513.6995, 1513.6995, 1550.6960, 1550.6960, 1557.7151, 1617.9590, 1647.7349, 1731.9867, 1901.1331, 2210.9834, 2283.0552

14. A0A4W2D8T3 **Mass:** 76089 **Score:** 56 **Expect:** 0.33 **Matches:** 9
Albumin OS=Bos indicus x Bos taurus OX=30522 GN=ALB PE=4 SV=1

Observed	Mr(expt)	Mr(calc)	ppm	Start	End	Miss	Ions	Peptide
927.4218	926.4145	926.4861	-77.29	209	215	0	---	K.YLYEIAR.R
1017.5948	1016.5875	1016.5727	14.6	260	268	1	---	K.VLTSSARQR.L
1439.7159	1438.7086	1438.8045	-66.61	408	419	1	---	R.RHPEYAVSVLLR.L
1479.6964	1478.6891	1478.7881	-66.96	469	481	0	---	K.LGEYGFQNALIVR.Y
1479.6964	1478.6891	1478.7881	-66.96	469	481	0	10	K.LGEYGFQNALIVR.Y
1567.6414	1566.6341	1566.7354	-64.69	395	407	0	27	K.DAFLGSFLYEYSR.R
1567.6414	1566.6341	1566.7354	-64.67	395	407	0	---	K.DAFLGSFLYEYSR.R
1724.7261	1723.7188	1723.8273	-62.93	517	530	0	---	R.MPCTEDYLSLILNR.L
2044.9095	2043.9022	2044.0206	-57.93	216	231	1	---	R.RHPYFYAPELLYYANK.Y

No match to: 805.4406, 842.4471, 848.4654, 849.9925, 850.4734, 853.4557, 864.4832, 865.9639, 868.4844, 870.4808, 876.9719, 882.5003, 882.5003, 892.9417, 908.9123, 919.3989, 951.5530, 953.4604, 996.5237, 998.4718, 1019.6071, 1033.6263, 1045.4912, 1051.6328, 1051.6328, 1065.5367, 1131.6298, 1133.6357, 1136.6206, 1139.4833, 1142.5555, 1142.5555, 1151.6481, 1156.5005, 1165.6569, 1165.6569, 1175.5602, 1184.6132, 1189.4962, 1247.6283, 1267.5582, 1267.5582, 1268.5472, 1287.5924, 1300.7646, 1305.5656, 1311.6147, 1334.7938, 1334.7938, 1387.5682, 1387.5682, 1396.6301, 1448.8251, 1455.6167, 1455.6167, 1475.6594, 1498.6063, 1513.6995, 1513.6995, 1550.6960, 1550.6960, 1557.7151, 1617.9590, 1647.7349, 1731.9867, 1901.1331, 2210.9834, 2283.0552

15. A0A140T897 **Mass:** 71274 **Score:** 56 **Expect:** 0.37 **Matches:** 9
Serum albumin OS=Bos taurus OX=9913 GN=ALB PE=4 SV=1

Observed	Mr(expt)	Mr(calc)	ppm	Start	End	Miss	Ions	Peptide
927.4218	926.4145	926.4861	-77.29	161	167	0	---	K.YLYEIAR.R
1017.5948	1016.5875	1016.5727	14.6	212	220	1	---	K.VLTSSARQR.L
1439.7159	1438.7086	1438.8045	-66.61	360	371	1	---	R.RHPEYAVSVLLR.L
1479.6964	1478.6891	1478.7881	-66.96	421	433	0	---	K.LGEYGFQNALIVR.Y
1479.6964	1478.6891	1478.7881	-66.96	421	433	0	10	K.LGEYGFQNALIVR.Y
1567.6414	1566.6341	1566.7354	-64.69	347	359	0	27	K.DAFLGSFLYEYSR.R
1567.6414	1566.6341	1566.7354	-64.67	347	359	0	---	K.DAFLGSFLYEYSR.R
1724.7261	1723.7188	1723.8273	-62.93	469	482	0	---	R.MPCTEDYLSLILNR.L
2044.9095	2043.9022	2044.0206	-57.93	168	183	1	---	R.RHPYFYAPELLYYANK.Y

No match to: 805.4406, 842.4471, 848.4654, 849.9925, 850.4734, 853.4557, 864.4832, 865.9639, 868.4844, 870.4808, 876.9719, 882.5003, 882.5003, 892.9417, 908.9123, 919.3989, 951.5530, 953.4604, 996.5237, 998.4718, 1019.6071, 1033.6263, 1045.4912, 1051.6328, 1051.6328, 1065.5367, 1131.6298, 1133.6357, 1136.6206, 1139.4833, 1142.5555, 1142.5555, 1151.6481, 1156.5005, 1165.6569, 1165.6569, 1175.5602, 1184.6132, 1189.4962, 1247.6283, 1267.5582, 1267.5582, 1268.5472, 1287.5924, 1300.7646,

PDF-XChange Click to buy NOW! www.docu-track.com

PDF-XChange Click to buy NOW! www.docu-track.com

1305.5656, 1311.6147, 1334.7938, 1334.7938, 1387.5682, 1387.5682, 1396.6301, 1448.8251, 1455.6167, 1455.6167, 1475.6594, 1498.6063, 1513.6995, 1513.6995, 1550.6960, 1550.6960, 1557.7151, 1617.9590, 1647.7349, 1731.9867, 1901.1331, 2210.9834, 2283.0552

16. A0A4W2EVR9 **Mass:** 71453 **Score:** 56 **Expect:** 0.37 **Matches:** 9

Albumin OS=Bos indicus x Bos taurus OX=30522 GN=ALB PE=4 SV=1

Observed	Mr(expt)	Mr(calc)	ppm	Start	End	Miss	Ions	Peptide
927.4218	926.4145	926.4861	-77.29	161	167	0	---	K.YLYEIAR.R
1017.5948	1016.5875	1016.5727	14.6	212	220	1	---	K.VLTSSARQR.L
1439.7159	1438.7086	1438.8045	-66.61	360	371	1	---	R.RHPEYAVSVLLR.L
1479.6964	1478.6891	1478.7881	-66.96	421	433	0	---	K.LGEYGFQNALIVR.Y
1479.6964	1478.6891	1478.7881	-66.96	421	433	0	10	K.LGEYGFQNALIVR.Y
1567.6414	1566.6341	1566.7354	-64.69	347	359	0	27	K.DAFLGSFLYEYSR.R
1567.6414	1566.6341	1566.7354	-64.67	347	359	0	---	K.DAFLGSFLYEYSR.R
1724.7261	1723.7188	1723.8273	-62.93	469	482	0	---	R.MPCTEDYLSLILNR.L
2044.9095	2043.9022	2044.0206	-57.93	168	183	1	---	R.RHPYFYAPELLYYANK.Y

No match to: 805.4406, 842.4471, 848.4654, 849.9925, 850.4734, 853.4557, 864.4832, 865.9639, 868.4844, 870.4808, 876.9719, 882.5003, 882.5003, 892.9417, 908.9123, 919.3989, 951.5530, 953.4604, 996.5237, 998.4718, 1019.6071, 1033.6263, 1045.4912, 1051.6328, 1051.6328, 1065.5367, 1131.6298, 1133.6357, 1136.6206, 1139.4833, 1142.5555, 1142.5555, 1151.6481, 1156.5005, 1165.6569, 1165.6569, 1175.5602, 1184.6132, 1189.4962, 1247.6283, 1267.5582, 1267.5582, 1268.5472, 1287.5924, 1300.7646, 1305.5656, 1311.6147, 1334.7938, 1334.7938, 1387.5682, 1387.5682, 1396.6301, 1448.8251, 1455.6167, 1455.6167, 1475.6594, 1498.6063, 1513.6995, 1513.6995, 1550.6960, 1550.6960, 1557.7151, 1617.9590, 1647.7349, 1731.9867, 1901.1331, 2210.9834, 2283.0552

17. A0A4W2EW60 **Mass:** 76020 **Score:** 55 **Expect:** 0.43 **Matches:** 9

Albumin OS=Bos indicus x Bos taurus OX=30522 GN=ALB PE=4 SV=1

Observed	Mr(expt)	Mr(calc)	ppm	Start	End	Miss	Ions	Peptide
927.4218	926.4145	926.4861	-77.29	161	167	0	---	K.YLYEIAR.R
1017.5948	1016.5875	1016.5727	14.6	212	220	1	---	K.VLTSSARQR.L
1439.7159	1438.7086	1438.8045	-66.61	360	371	1	---	R.RHPEYAVSVLLR.L
1479.6964	1478.6891	1478.7881	-66.96	421	433	0	---	K.LGEYGFQNALIVR.Y
1479.6964	1478.6891	1478.7881	-66.96	421	433	0	10	K.LGEYGFQNALIVR.Y
1567.6414	1566.6341	1566.7354	-64.69	347	359	0	27	K.DAFLGSFLYEYSR.R
1567.6414	1566.6341	1566.7354	-64.67	347	359	0	---	K.DAFLGSFLYEYSR.R
1724.7261	1723.7188	1723.8273	-62.93	469	482	0	---	R.MPCTEDYLSLILNR.L
2044.9095	2043.9022	2044.0206	-57.93	168	183	1	---	R.RHPYFYAPELLYYANK.Y

No match to: 805.4406, 842.4471, 848.4654, 849.9925, 850.4734, 853.4557, 864.4832, 865.9639, 868.4844, 870.4808, 876.9719, 882.5003, 882.5003, 892.9417, 908.9123, 919.3989, 951.5530, 953.4604, 996.5237, 998.4718, 1019.6071, 1033.6263, 1045.4912, 1051.6328, 1051.6328, 1065.5367, 1131.6298, 1133.6357, 1136.6206, 1139.4833, 1142.5555, 1142.5555, 1151.6481, 1156.5005, 1165.6569, 1165.6569, 1175.5602, 1184.6132, 1189.4962, 1247.6283, 1267.5582, 1267.5582, 1268.5472, 1287.5924, 1300.7646, 1305.5656, 1311.6147, 1334.7938, 1334.7938, 1387.5682, 1387.5682, 1396.6301, 1448.8251, 1455.6167, 1455.6167, 1475.6594, 1498.6063, 1513.6995, 1513.6995, 1550.6960, 1550.6960, 1557.7151, 1617.9590, 1647.7349, 1731.9867, 1901.1331, 2210.9834, 2283.0552

18. P02769 **Mass:** 71244 **Score:** 55 **Expect:** 0.46 **Matches:** 8

Serum albumin OS=Bos taurus OX=9913 GN=ALB PE=1 SV=4

Observed	Mr(expt)	Mr(calc)	ppm	Start	End	Miss	Ions	Peptide
927.4218	926.4145	926.4861	-77.29	161	167	0	---	K.YLYEIAR.R
1439.7159	1438.7086	1438.8045	-66.61	360	371	1	---	R.RHPEYAVSVLLR.L
1479.6964	1478.6891	1478.7881	-66.96	421	433	0	---	K.LGEYGFQNALIVR.Y
1479.6964	1478.6891	1478.7881	-66.96	421	433	0	10	K.LGEYGFQNALIVR.Y
1567.6414	1566.6341	1566.7354	-64.69	347	359	0	27	K.DAFLGSFLYEYSR.R
1567.6414	1566.6341	1566.7354	-64.67	347	359	0	---	K.DAFLGSFLYEYSR.R
1724.7261	1723.7188	1723.8273	-62.93	469	482	0	---	R.MPCTEDYLSLILNR.L
2044.9095	2043.9022	2044.0206	-57.93	168	183	1	---	R.RHPYFYAPELLYYANK.Y

No match to: 805.4406, 842.4471, 848.4654, 849.9925, 850.4734, 853.4557, 864.4832, 865.9639, 868.4844, 870.4808, 876.9719, 882.5003, 882.5003, 892.9417, 908.9123, 919.3989, 951.5530, 953.4604, 996.5237, 998.4718, 1017.5948, 1019.6071, 1033.6263, 1045.4912, 1051.6328, 1051.6328, 1065.5367, 1131.6298, 1133.6357, 1136.6206, 1139.4833, 1142.5555, 1142.5555, 1151.6481, 1156.5005, 1165.6569, 1165.6569, 1175.5602, 1184.6132, 1189.4962, 1247.6283, 1267.5582, 1267.5582, 1268.5472, 1287.5924, 1300.7646, 1305.5656, 1311.6147, 1334.7938, 1334.7938, 1387.5682, 1387.5682, 1396.6301, 1448.8251, 1455.6167, 1455.6167, 1475.6594, 1498.6063, 1513.6995, 1513.6995, 1550.6960, 1550.6960, 1557.7151, 1617.9590, 1647.7349, 1731.9867, 1901.1331, 2210.9834, 2283.0552

19. C1J945 **Mass:** 56189 **Score:** 48 **Expect:** 2.1 **Matches:** 12

Nonstructrual protein 5A (Fragment) OS=Bovine viral diarrhea virus OX=11099 PE=4 SV=1

Observed	Mr(expt)	Mr(calc)	ppm	Start	End	Miss	Ions	Peptide
805.4406	804.4333	804.4341	-1.00	68	75	0	---	R.NVSGSLTK.V
927.4218	926.4145	926.4974	-89.46	156	163	1	---	K.RYTGVGFK.G
1033.6263	1032.6190	1032.5716	45.9	91	99	1	---	R.GPVNYRVTK.Y
1133.6357	1132.6284	1132.6427	-12.59	338	347	0	---	R.RPFVMVLGSK.T
1189.4962	1188.4889	1188.5921	-86.78	370	379	1	---	R.EIRDLMAEGR.M
1287.5924	1286.5851	1286.6507	-50.94	459	469	0	---	K.YHLVGDVEEVK.R
1557.7151	1556.7078	1556.7769	-44.40	143	155	1	---	K.WEVEHGVVTRMAK.R + Oxidation (M)
1567.6414	1566.6341	1566.7453	-71.01	387	399	0	---	R.EFDPELSELVDFK.G
1567.6414	1566.6341	1566.7453	-70.99	387	399	0	---	R.EFDPELSELVDFK.G
1724.7261	1723.7188	1723.8781	-92.37	100	113	1	---	K.YYDDDLKEIKPVAR.L
1731.9867	1730.9794	1730.8298	86.5	197	211	0	---	K.GCAFTYDLTLSNLTR.L
2044.9095	2043.9022	2044.0741	-84.11	107	123	1	---	K.EIKPVARLEGQVDHYYK.G

No match to: 842.4471, 848.4654, 849.9925, 850.4734, 853.4557, 864.4832, 865.9639, 868.4844, 870.4808, 876.9719, 882.5003, 882.5003, 892.9417, 908.9123, 919.3989, 951.5530, 953.4604, 996.5237, 998.4718, 1017.5948, 1019.6071, 1045.4912, 1051.6328, 1051.6328, 1065.5367, 1131.6298, 1136.6206, 1139.4833, 1142.5555, 1142.5555, 1151.6481, 1156.5005, 1165.6569, 1165.6569, 1175.5602, 1184.6132, 1247.6283, 1267.5582, 1267.5582, 1268.5472, 1300.7646, 1305.5656, 1311.6147, 1334.7938, 1334.7938, 1387.5682, 1387.5682, 1396.6301, 1439.7159, 1448.8251, 1455.6167, 1455.6167, 1475.6594, 1479.6964, 1479.6964, 1498.6063, 1513.6995, 1513.6995, 1550.6960, 1550.6960, 1617.9590, 1647.7349, 1901.1331, 2210.9834, 2283.0552

20. A0A4W2EGF3 **Mass:** 97654 **Score:** 46 **Expect:** 3.8 **Matches:** 13

Dynamin 3 OS=Bos indicus x Bos taurus OX=30522 GN=DNM3 PE=3 SV=1

Observed	Mr(expt)	Mr(calc)	ppm	Start	End	Miss	Ions	Peptide
927.4218	926.4145	926.4644	-53.82	272	279	0	---	R.MGTPHLQK.V + Oxidation (M)
1019.6071	1018.5998	1018.5672	32.0	229	237	1	---	R.RGYVGVVNR.S
1133.6357	1132.6284	1132.5910	33.0	247	256	1	---	K.DIKAAMLAER.K + Oxidation (M)
1136.6206	1135.6133	1135.6098	3.10	762	771	0	---	R.RPPPSPTTQR.R
1142.5555	1141.5482	1141.6567	-95.05	510	519	1	---	K.NTIGNQVIRK.G
1142.5555	1141.5483	1141.6567	-95.01	509	518	1	---	K.KNTIGNQVIR.K
1156.5005	1155.4932	1155.5560	-54.37	90	98	1	---	K.KFTDFDEVR.H
1184.6132	1183.6059	1183.5729	27.9	158	166	0	---	R.EMIMQFITR.E + Oxidation (M)

PDF-XChange Click to buy NOW! www.docu-track.com
PDF-XChange Click to buy NOW! www.docu-track.com

1267.5582	1266.5509	1266.5703	-15.28	78	87	0	---	K.AEYAEFLHCK.G
1267.5582	1266.5510	1266.5703	-15.26	78	87	0	---	K.AEYAEFLHCK.G
1396.6301	1395.6228	1395.7068	-60.15	658	669	0	---	R.NLVDSYMSIINK.C
1513.6995	1512.6922	1512.8412	-98.51	387	399	1	18	R.EISYAIKNIHGIR.T
1513.6995	1512.6922	1512.8412	-98.49	387	399	1	---	R.EISYAIKNIHGIR.T

No match to: 805.4406, 842.4471, 848.4654, 849.9925, 850.4734, 853.4557, 864.4832, 865.9639, 868.4844, 870.4808, 876.9719, 882.5003, 882.5003, 892.9417, 908.9123, 919.3989, 951.5530, 953.4604, 996.5237, 998.4718, 1017.5948, 1033.6263, 1045.4912, 1051.6328, 1051.6328, 1065.5367, 1131.6298, 1139.4833, 1151.6481, 1165.6569, 1165.6569, 1175.5602, 1189.4962, 1247.6283, 1268.5472, 1287.5924, 1300.7646, 1305.5656, 1311.6147, 1334.7938, 1334.7938, 1387.5682, 1387.5682, 1439.7159, 1448.8251, 1455.6167, 1455.6167, 1475.6594, 1479.6964, 1479.6964, 1498.6063, 1550.6960, 1550.6960, 1557.7151, 1567.6414, 1567.6414, 1617.9590, 1647.7349, 1724.7261, 1731.9867, 1901.1331, 2044.9095, 2210.9834, 2283.0552

21. A0A3Q1MDN4 **Mass:** 96034 **Score:** 45 **Expect:** 4.1 **Matches:** 13

Uncharacterized protein OS=Bos taurus OX=9913 GN=DNM3 PE=3 SV=1

Observed	Mr(expt)	Mr(calc)	ppm	Start	End	Miss	Ions	Peptide
927.4218	926.4145	926.4644	-53.82	272	279	0	---	R.MGTPHLQK.V + Oxidation (M)
1019.6071	1018.5998	1018.5672	32.0	229	237	1	---	R.RGYVGVVNR.S
1131.6298	1130.6225	1130.7063	-74.10	839	849	0	---	R.LPQGLPLALLP.-
1133.6357	1132.6284	1132.5910	33.0	247	256	1	---	K.DIKAAMLAER.K + Oxidation (M)
1142.5555	1141.5482	1141.6567	-95.05	510	519	1	---	K.NTIGNQVIRK.G
1142.5555	1141.5483	1141.6567	-95.01	509	518	1	---	K.KNTIGNQVIR.K
1156.5005	1155.4932	1155.5560	-54.37	90	98	1	---	K.KFTDFDEVR.H
1184.6132	1183.6059	1183.5729	27.9	158	166	0	---	R.EMIMQFITR.E + Oxidation (M)
1267.5582	1266.5509	1266.5703	-15.28	78	87	0	---	K.AEYAEFLHCK.G
1267.5582	1266.5510	1266.5703	-15.26	78	87	0	---	K.AEYAEFLHCK.G
1396.6301	1395.6228	1395.7068	-60.15	654	665	0	---	R.NLVDSYMSIINK.C
1513.6995	1512.6922	1512.8412	-98.51	387	399	1	18	R.EISYAIKNIHGIR.T
1513.6995	1512.6922	1512.8412	-98.49	387	399	1	---	R.EISYAIKNIHGIR.T

No match to: 805.4406, 842.4471, 848.4654, 849.9925, 850.4734, 853.4557, 864.4832, 865.9639, 868.4844, 870.4808, 876.9719, 882.5003, 882.5003, 892.9417, 908.9123, 919.3989, 951.5530, 953.4604, 996.5237, 998.4718, 1017.5948, 1033.6263, 1045.4912, 1051.6328, 1051.6328, 1065.5367, 1136.6206, 1139.4833, 1151.6481, 1165.6569, 1165.6569, 1175.5602, 1189.4962, 1247.6283, 1268.5472, 1287.5924, 1300.7646, 1305.5656, 1311.6147, 1334.7938, 1334.7938, 1387.5682, 1387.5682, 1439.7159, 1448.8251, 1455.6167, 1455.6167, 1475.6594, 1479.6964, 1479.6964, 1498.6063, 1550.6960, 1550.6960, 1557.7151, 1567.6414, 1567.6414, 1617.9590, 1647.7349, 1724.7261, 1731.9867, 1901.1331, 2044.9095, 2210.9834, 2283.0552

22. A0A4W2EI53 **Mass:** 63308 **Score:** 44 **Expect:** 5.7 **Matches:** 11

Dynamin 3 OS=Bos indicus x Bos taurus OX=30522 GN=DNM3 PE=3 SV=1

Observed	Mr(expt)	Mr(calc)	ppm	Start	End	Miss	Ions	Peptide
927.4218	926.4145	926.4644	-53.82	272	279	0	---	R.MGTPHLQK.V + Oxidation (M)
1019.6071	1018.5998	1018.5672	32.0	229	237	1	---	R.RGYVGVVNR.S
1133.6357	1132.6284	1132.5910	33.0	247	256	1	---	K.DIKAAMLAER.K + Oxidation (M)
1142.5555	1141.5482	1141.6567	-95.05	510	519	1	---	K.NTIGNQVIRK.G
1142.5555	1141.5483	1141.6567	-95.01	509	518	1	---	K.KNTIGNQVIR.K
1156.5005	1155.4932	1155.5560	-54.37	90	98	1	---	K.KFTDFDEVR.H
1184.6132	1183.6059	1183.5729	27.9	158	166	0	---	R.EMIMQFITR.E + Oxidation (M)
1267.5582	1266.5509	1266.5703	-15.28	78	87	0	---	K.AEYAEFLHCK.G
1267.5582	1266.5510	1266.5703	-15.26	78	87	0	---	K.AEYAEFLHCK.G
1513.6995	1512.6922	1512.8412	-98.51	387	399	1	18	R.EISYAIKNIHGIR.T
1513.6995	1512.6922	1512.8412	-98.49	387	399	1	---	R.EISYAIKNIHGIR.T

No match to: 805.4406, 842.4471, 848.4654, 849.9925, 850.4734, 853.4557, 864.4832, 865.9639, 868.4844, 870.4808, 876.9719, 882.5003, 882.5003, 892.9417, 908.9123, 919.3989, 951.5530, 953.4604, 996.5237, 998.4718, 1017.5948, 1033.6263, 1045.4912, 1051.6328, 1051.6328, 1065.5367, 1131.6298, 1136.6206, 1139.4833, 1151.6481, 1165.6569, 1165.6569, 1175.5602, 1189.4962, 1247.6283, 1268.5472, 1287.5924, 1300.7646, 1305.5656, 1311.6147, 1334.7938, 1334.7938, 1387.5682, 1387.5682, 1396.6301, 1439.7159, 1448.8251, 1455.6167, 1455.6167, 1475.6594, 1479.6964, 1479.6964, 1498.6063, 1550.6960, 1550.6960, 1557.7151, 1567.6414, 1567.6414, 1617.9590, 1647.7349, 1724.7261, 1731.9867, 1901.1331, 2044.9095, 2210.9834, 2283.0552

23. A0A4W2IJA6 **Mass:** 101091 **Score:** 43 **Expect:** 7.1 **Matches:** 14

Family with sequence similarity 111 member B OS=Bos indicus x Bos taurus OX=30522 GN=FAM111B PE=4 SV=1

Observed	Mr(expt)	Mr(calc)	ppm	Start	End	Miss	Ions	Peptide
842.4471	841.4398	841.5021	-74.03	528	534	0	---	K.NLQILNK.V
998.4718	997.4645	997.5015	-37.06	289	297	0	---	R.CLPSGSHLK.I
1017.5948	1016.5875	1016.5363	50.4	88	96	1	---	R.NLDRGSLSR.H
1045.4912	1044.4839	1044.5815	-93.44	391	400	0	---	K.QSIVDIVSGK.V
1136.6206	1135.6133	1135.5305	72.9	32	41	1	---	R.RCPHDAPGAR.L
1156.5005	1155.4932	1155.5309	-32.56	553	560	1	---	K.YFQEERER.M
1247.6283	1246.6210	1246.6094	9.31	486	494	1	---	R.YYNNNLYKR.L
1305.5656	1304.5583	1304.6435	-65.27	642	652	0	---	K.SWPDIISTCVK.V
1396.6301	1395.6228	1395.6743	-36.85	3	15	0	---	K.GQDGLKPDPEQGR.G
1475.6594	1474.6521	1474.7892	-92.97	628	640	0	---	R.HVLHLIVGESTDR.K
1557.7151	1556.7078	1556.6703	24.1	449	461	1	---	K.DGETEDVEHNREK.I
1567.6414	1566.6341	1566.7402	-67.71	800	812	0	7	K.LVAMHSFGHFYSR.V + Oxidation (M)
1567.6414	1566.6341	1566.7402	-67.69	800	812	0	---	K.LVAMHSFGHFYSR.V + Oxidation (M)
2044.9095	2043.9022	2044.0340	-64.45	642	658	1	---	K.SWPDIISTCVKVTFTYK.E

No match to: 805.4406, 848.4654, 849.9925, 850.4734, 853.4557, 864.4832, 865.9639, 868.4844, 870.4808, 876.9719, 882.5003, 882.5003, 892.9417, 908.9123, 919.3989, 927.4218, 951.5530, 953.4604, 996.5237, 1019.6071, 1033.6263, 1051.6328, 1051.6328, 1065.5367, 1131.6298, 1133.6357, 1139.4833, 1142.5555, 1142.5555, 1151.6481, 1165.6569, 1165.6569, 1175.5602, 1184.6132, 1189.4962, 1267.5582, 1267.5582, 1268.5472, 1287.5924, 1300.7646, 1311.6147, 1334.7938, 1334.7938, 1387.5682, 1387.5682, 1439.7159, 1448.8251, 1455.6167, 1455.6167, 1479.6964, 1479.6964, 1498.6063, 1513.6995, 1513.6995, 1550.6960, 1550.6960, 1617.9590, 1647.7349, 1724.7261, 1731.9867, 1901.1331, 2210.9834, 2283.0552

24. A0A4W2ED96 **Mass:** 52578 **Score:** 42 **Expect:** 7.7 **Matches:** 13

6-phosphofructo-2-kinase/fructose-2,6-biphosphatase 1 OS=Bos indicus x Bos taurus OX=30522 GN=PFKFB1 PE=4 SV=1

Observed	Mr(expt)	Mr(calc)	ppm	Start	End	Miss	Ions	Peptide
850.4734	849.4661	849.4014	76.2	1	7	1	---	-.MEEKASR.R
996.5237	995.5164	995.5189	-2.45	52	59	0	---	K.VFNLGQYR.R
1019.6071	1018.5998	1018.5811	18.4	166	173	1	---	K.VLEDFLKR.I
1051.6328	1050.6255	1050.5233	97.3	385	393	0	---	K.SSDELPYLK.C
1051.6328	1050.6255	1050.5233	97.3	385	393	0	---	K.SSDELPYLK.C
1142.5555	1141.5482	1141.5842	-31.47	376	384	0	---	R.CLLAYFLDK.S
1142.5555	1141.5483	1141.5842	-31.44	376	384	0	---	R.CLLAYFLDK.S
1267.5582	1266.5509	1266.6429	-72.59	236	246	1	---	R.HGESELNLRGR.I
1267.5582	1266.5510	1266.6429	-72.57	236	246	1	13	R.HGESELNLRGR.I
1479.6964	1478.6891	1478.7478	-39.65	104	116	1	---	R.VAVFDATNTTRER.R
1479.6964	1478.6891	1478.7478	-39.64	104	116	1	---	R.VAVFDATNTTRER.R
1567.6414	1566.6341	1566.6984	-41.06	153	165	1	---	K.LGSPDYIDCDREK.V

PDF-XChange Click to buy NOW! www.docu-track.com

PDF-XChange Click to buy NOW! www.docu-track.com

Observed	Mr(expt)	Mr(calc)	ppm	Start	End	Miss	Ions	Peptide
1567.6414	1566.6341	1566.6984	-41.03	153	165	1	---	K.LGSPDYIDCDREK.V

No match to: 805.4406, 842.4471, 848.4654, 849.9925, 853.4557, 864.4832, 865.9639, 868.4844, 870.4808, 876.9719, 882.5003, 882.5003, 892.9417, 908.9123, 919.3989, 927.4218, 951.5530, 953.4604, 998.4718, 1017.5948, 1033.6263, 1045.4912, 1065.5367, 1131.6298, 1133.6357, 1136.6206, 1139.4833, 1151.6481, 1156.5005, 1165.6569, 1165.6569, 1175.5602, 1184.6132, 1189.4962, 1247.6283, 1268.5472, 1287.5924, 1300.7646, 1305.5656, 1311.6147, 1334.7938, 1334.7938, 1387.5682, 1387.5682, 1396.6301, 1439.7159, 1448.8251, 1455.6167, 1455.6167, 1475.6594, 1498.6063, 1513.6995, 1513.6995, 1550.6960, 1550.6960, 1557.7151, 1617.9590, 1647.7349, 1724.7261, 1731.9867, 1901.1331, 2044.9095, 2210.9834, 2283.0552

25. A0A3Q1LXR0 **Mass:** 52578 **Score:** 42 **Expect:** 7.7 **Matches:** 13

6-phosphofructo-2-kinase/fructose-2,6-bisphosphatase 1 OS=Bos taurus OX=9913 GN=PFKFB1 PE=4 SV=1

Observed	Mr(expt)	Mr(calc)	ppm	Start	End	Miss	Ions	Peptide
850.4734	849.4661	849.4014	76.2	1	7	1	---	-.MEEKASR.R
996.5237	995.5164	995.5189	-2.45	52	59	0	---	K.VFNLGQYR.R
1019.6071	1018.5998	1018.5811	18.4	166	173	1	---	K.VLEDFLKR.I
1051.6328	1050.6255	1050.5233	97.3	385	393	0	---	K.SSDELPYLK.C
1051.6328	1050.6255	1050.5233	97.3	385	393	0	---	K.SSDELPYLK.C
1142.5555	1141.5482	1141.5842	-31.47	376	384	0	---	R.CLLAYFLDK.S
1142.5555	1141.5483	1141.5842	-31.44	376	384	0	---	R.CLLAYFLDK.S
1267.5582	1266.5509	1266.6429	-72.59	236	246	1	---	R.HGESELNLRGR.I
1267.5582	1266.5510	1266.6429	-72.57	236	246	1	13	R.HGESELNLRGR.I
1479.6964	1478.6891	1478.7478	-39.65	104	116	1	---	R.VAVFDATNTTRER.R
1479.6964	1478.6891	1478.7478	-39.64	104	116	1	---	R.VAVFDATNTTRER.R
1567.6414	1566.6341	1566.6984	-41.06	153	165	1	---	K.LGSPDYIDCDREK.V
1567.6414	1566.6341	1566.6984	-41.03	153	165	1	---	K.LGSPDYIDCDREK.V

No match to: 805.4406, 842.4471, 848.4654, 849.9925, 853.4557, 864.4832, 865.9639, 868.4844, 870.4808, 876.9719, 882.5003, 882.5003, 892.9417, 908.9123, 919.3989, 927.4218, 951.5530, 953.4604, 998.4718, 1017.5948, 1033.6263, 1045.4912, 1065.5367, 1131.6298, 1133.6357, 1136.6206, 1139.4833, 1151.6481, 1156.5005, 1165.6569, 1165.6569, 1175.5602, 1184.6132, 1189.4962, 1247.6283, 1268.5472, 1287.5924, 1300.7646, 1305.5656, 1311.6147, 1334.7938, 1334.7938, 1387.5682, 1387.5682, 1396.6301, 1439.7159, 1448.8251, 1455.6167, 1455.6167, 1475.6594, 1498.6063, 1513.6995, 1513.6995, 1550.6960, 1550.6960, 1557.7151, 1617.9590, 1647.7349, 1724.7261, 1731.9867, 1901.1331, 2044.9095, 2210.9834, 2283.0552

26. A0A4W2EAJ9 **Mass:** 72734 **Score:** 40 **Expect:** 13 **Matches:** 11

Dynamin 3 OS=Bos indicus x Bos taurus OX=30522 GN=DNM3 PE=3 SV=1

Observed	Mr(expt)	Mr(calc)	ppm	Start	End	Miss	Ions	Peptide
927.4218	926.4145	926.4644	-53.82	272	279	0	---	R.MGTPHLQK.V + Oxidation (M)
1019.6071	1018.5998	1018.5672	32.0	229	237	1	---	R.RGYVGVVNR.S
1133.6357	1132.6284	1132.5910	33.0	247	256	1	---	K.DIKAAMLAER.K + Oxidation (M)
1142.5555	1141.5482	1141.6567	-95.05	510	519	1	---	K.NTIGNQVIRK.G
1142.5555	1141.5483	1141.6567	-95.01	509	518	1	---	K.KNTIGNQVIR.K
1156.5005	1155.4932	1155.5560	-54.37	90	98	1	---	K.KFTDFDEVR.H
1184.6132	1183.6059	1183.5729	27.9	158	166	0	---	R.EMIMQFITR.E + Oxidation (M)
1267.5582	1266.5509	1266.5703	-15.28	78	87	0	---	K.AEYAEFLHCK.G
1267.5582	1266.5510	1266.5703	-15.26	78	87	0	---	K.AEYAEFLHCK.G
1513.6995	1512.6922	1512.8412	-98.51	387	399	1	18	R.EISYAIKNIHGIR.T
1513.6995	1512.6922	1512.8412	-98.49	387	399	1	---	R.EISYAIKNIHGIR.T

No match to: 805.4406, 842.4471, 848.4654, 849.9925, 850.4734, 853.4557, 864.4832, 865.9639, 868.4844, 870.4808, 876.9719, 882.5003, 882.5003, 892.9417, 908.9123, 919.3989, 951.5530, 953.4604, 996.5237, 998.4718, 1017.5948, 1033.6263, 1045.4912, 1051.6328, 1051.6328, 1065.5367, 1131.6298, 1136.6206, 1139.4833, 1151.6481, 1165.6569, 1165.6569, 1175.5602, 1189.4962, 1247.6283, 1268.5472, 1287.5924, 1300.7646, 1305.5656, 1311.6147, 1334.7938, 1334.7938, 1387.5682, 1387.5682, 1396.6301, 1439.7159, 1448.8251, 1455.6167, 1455.6167, 1475.6594, 1479.6964, 1479.6964, 1498.6063, 1550.6960, 1550.6960, 1557.7151, 1567.6414, 1567.6414, 1617.9590, 1647.7349, 1724.7261, 1731.9867, 1901.1331, 2044.9095, 2210.9834, 2283.0552

27. A0A4W2GW83 **Mass:** 70119 **Score:** 40 **Expect:** 13 **Matches:** 6

Albumin OS=Bos indicus x Bos taurus OX=30522 GN=ALB PE=4 SV=1

Observed	Mr(expt)	Mr(calc)	ppm	Start	End	Miss	Ions	Peptide
927.4218	926.4145	926.4861	-77.29	152	158	0	---	K.YLYEIAR.R
1017.5948	1016.5875	1016.5727	14.6	203	211	1	---	K.VLTSSARQR.L
1439.7159	1438.7086	1438.8045	-66.61	351	362	1	---	R.RHPEYAVSVLLR.L
1567.6414	1566.6341	1566.7354	-64.69	338	350	0	27	K.DAFLGSFLYEYSR.R
1567.6414	1566.6341	1566.7354	-64.67	338	350	0	---	K.DAFLGSFLYEYSR.R
2044.9095	2043.9022	2044.0206	-57.93	159	174	1	---	R.RHPYFYAPELLYYANK.Y

No match to: 805.4406, 842.4471, 848.4654, 849.9925, 850.4734, 853.4557, 864.4832, 865.9639, 868.4844, 870.4808, 876.9719, 882.5003, 882.5003, 892.9417, 908.9123, 919.3989, 951.5530, 953.4604, 996.5237, 998.4718, 1019.6071, 1033.6263, 1045.4912, 1051.6328, 1051.6328, 1065.5367, 1131.6298, 1133.6357, 1136.6206, 1139.4833, 1142.5555, 1142.5555, 1151.6481, 1156.5005, 1165.6569, 1165.6569, 1175.5602, 1184.6132, 1189.4962, 1247.6283, 1267.5582, 1267.5582, 1268.5472, 1287.5924, 1300.7646, 1305.5656, 1311.6147, 1334.7938, 1334.7938, 1387.5682, 1387.5682, 1396.6301, 1448.8251, 1455.6167, 1455.6167, 1475.6594, 1479.6964, 1479.6964, 1498.6063, 1513.6995, 1513.6995, 1550.6960, 1550.6960, 1557.7151, 1617.9590, 1647.7349, 1724.7261, 1731.9867, 1901.1331, 2210.9834, 2283.0552

28. B0JYQ0 **Mass:** 71244 **Score:** 40 **Expect:** 14 **Matches:** 6

ALB protein OS=Bos taurus OX=9913 GN=ALB PE=2 SV=1

Observed	Mr(expt)	Mr(calc)	ppm	Start	End	Miss	Ions	Peptide
927.4218	926.4145	926.4861	-77.29	161	167	0	---	K.YLYEIAR.R
1017.5948	1016.5875	1016.5727	14.6	212	220	1	---	K.VLTSSARQR.L
1439.7159	1438.7086	1438.8045	-66.61	360	371	1	---	R.RHPEYAVSVLLR.L
1567.6414	1566.6341	1566.7354	-64.69	347	359	0	27	K.DAFLGSFLYEYSR.R
1567.6414	1566.6341	1566.7354	-64.67	347	359	0	---	K.DAFLGSFLYEYSR.R
2044.9095	2043.9022	2044.0206	-57.93	168	183	1	---	R.RHPYFYAPELLYYANK.Y

No match to: 805.4406, 842.4471, 848.4654, 849.9925, 850.4734, 853.4557, 864.4832, 865.9639, 868.4844, 870.4808, 876.9719, 882.5003, 882.5003, 892.9417, 908.9123, 919.3989, 951.5530, 953.4604, 996.5237, 998.4718, 1019.6071, 1033.6263, 1045.4912, 1051.6328, 1051.6328, 1065.5367, 1131.6298, 1133.6357, 1136.6206, 1139.4833, 1142.5555, 1142.5555, 1151.6481, 1156.5005, 1165.6569, 1165.6569, 1175.5602, 1184.6132, 1189.4962, 1247.6283, 1267.5582, 1267.5582, 1268.5472, 1287.5924, 1300.7646, 1305.5656, 1311.6147, 1334.7938, 1334.7938, 1387.5682, 1387.5682, 1396.6301, 1448.8251, 1455.6167, 1455.6167, 1475.6594, 1479.6964, 1479.6964, 1498.6063, 1513.6995, 1513.6995, 1550.6960, 1550.6960, 1557.7151, 1617.9590, 1647.7349, 1724.7261, 1731.9867, 1901.1331, 2210.9834, 2283.0552

29. A0A4W2D9L2 **Mass:** 98304 **Score:** 39 **Expect:** 16 **Matches:** 11

Dynamin 3 OS=Bos indicus x Bos taurus OX=30522 GN=DNM3 PE=3 SV=1

Observed	Mr(expt)	Mr(calc)	ppm	Start	End	Miss	Ions	Peptide
927.4218	926.4145	926.4644	-53.82	272	279	0	---	R.MGTPHLQK.V + Oxidation (M)
1019.6071	1018.5998	1018.5672	32.0	229	237	1	---	R.RGYVGVVNR.S
1133.6357	1132.6284	1132.5910	33.0	247	256	1	---	K.DIKAAMLAER.K + Oxidation (M)
1136.6206	1135.6133	1135.6098	3.10	768	777	0	---	R.RPPPSPTTQR.R
1156.5005	1155.4932	1155.5560	-54.37	90	98	1	---	K.KFTDFDEVR.H
1184.6132	1183.6059	1183.5729	27.9	158	166	0	---	R.EMIMQFITR.E + Oxidation (M)
1267.5582	1266.5509	1266.5703	-15.28	78	87	0	---	K.AEYAEFLHCK.G

PDF-XChange Click to buy NOW! www.docu-track.com

PDF-XChange Click to buy NOW! www.docu-track.com

1267.5582	1266.5510	1266.5703	-15.26	78 - 87	0	---	K.AEYAEFLHCK.G
1396.6301	1395.6228	1395.7068	-60.15	664 - 675	0	---	R.NLVDSYMSIINK.C
1513.6995	1512.6922	1512.8412	-98.51	387 - 399	1	18	R.EISYAIKNIHGIR.T
1513.6995	1512.6922	1512.8412	-98.49	387 - 399	1	---	R.EISYAIKNIHGIR.T

No match to: 805.4406, 842.4471, 848.4654, 849.9925, 850.4734, 853.4557, 864.4832, 865.9639, 868.4844, 870.4808, 876.9719, 882.5003, 882.5003, 892.9417, 908.9123, 919.3989, 951.5530, 953.4604, 996.5237, 998.4718, 1017.5948, 1033.6263, 1045.4912, 1051.6328, 1051.6328, 1065.5367, 1131.6298, 1139.4833, 1142.5555, 1142.5555, 1151.6481, 1165.6569, 1165.6569, 1175.5602, 1189.4962, 1247.6283, 1268.5472, 1287.5924, 1300.7646, 1305.5656, 1311.6147, 1334.7938, 1334.7938, 1387.5682, 1387.5682, 1439.7159, 1448.8251, 1455.6167, 1455.6167, 1475.6594, 1479.6964, 1479.6964, 1498.6063, 1550.6960, 1550.6960, 1557.7151, 1567.6414, 1567.6414, 1617.9590, 1647.7349, 1724.7261, 1731.9867, 1901.1331, 2044.9095, 2210.9834, 2283.0552

30. A0A4W2IKT9 **Mass:** 75992 **Score:** 39 **Expect:** 17 **Matches:** 6

Albumin OS=Bos indicus x Bos taurus OX=30522 GN=ALB PE=4 SV=1

Observed	Mr(expt)	Mr(calc)	ppm	Start - End	Miss	Ions	Peptide
927.4218	926.4145	926.4861	-77.29	161 - 167	0	---	K.YLYEIAR.R
1017.5948	1016.5875	1016.5727	14.6	212 - 220	1	---	K.VLTSSARQR.L
1439.7159	1438.7086	1438.8045	-66.61	360 - 371	1	---	R.RHPEYAVSVLLR.L
1567.6414	1566.6341	1566.7354	-64.69	347 - 359	0	27	K.DAFLGSFLYEYSR.R
1567.6414	1566.6341	1566.7354	-64.67	347 - 359	0	---	K.DAFLGSFLYEYSR.R
2044.9095	2043.9022	2044.0206	-57.93	168 - 183	1	---	R.RHPYFYAPELLYYANK.Y

No match to: 805.4406, 842.4471, 848.4654, 849.9925, 850.4734, 853.4557, 864.4832, 865.9639, 868.4844, 870.4808, 876.9719, 882.5003, 882.5003, 892.9417, 908.9123, 919.3989, 951.5530, 953.4604, 996.5237, 998.4718, 1019.6071, 1033.6263, 1045.4912, 1051.6328, 1051.6328, 1065.5367, 1131.6298, 1133.6357, 1136.6206, 1139.4833, 1142.5555, 1142.5555, 1151.6481, 1156.5005, 1165.6569, 1165.6569, 1175.5602, 1184.6132, 1189.4962, 1247.6283, 1267.5582, 1267.5582, 1268.5472, 1287.5924, 1300.7646, 1305.5656, 1311.6147, 1334.7938, 1334.7938, 1387.5682, 1387.5682, 1396.6301, 1448.8251, 1455.6167, 1455.6167, 1475.6594, 1479.6964, 1479.6964, 1498.6063, 1513.6995, 1513.6995, 1550.6960, 1550.6960, 1557.7151, 1617.9590, 1647.7349, 1724.7261, 1731.9867, 1901.1331, 2210.9834, 2283.0552

Search Parameters

Type of search : Sequence Query
Enzyme : Trypsin
Fixed modifications : Carbamidomethyl (C)
Variable modifications : Oxidation (M)
Mass values : Monoisotopic
Protein Mass : Unrestricted
Peptide Mass Tolerance : ± 100 ppm
Fragment Mass Tolerance: ± 0.3 Da
Max Missed Cleavages : 1
Instrument type : MALDI-TOF-TOF
Query1 (805.4406,1+): <no title>
Query2 (842.4471,1+): <no title>
Query3 (848.4654,1+): <no title>
Query4 (849.9925,1+): <no title>
Query5 (850.4734,1+): <no title>
Query6 (853.4557,1+): <no title>
Query7 (864.4832,1+): <no title>
Query8 (865.9639,1+): <no title>
Query9 (868.4844,1+): <no title>
Query10 (870.4808,1+): <no title>
Query11 (876.9719,1+): <no title>
Query12 (882.5003,1+): <no title>
Query13 (882.5003,1+): Locus:1..14.0.7
Query14 (892.9417,1+): <no title>
Query15 (908.9123,1+): <no title>
Query16 (919.3989,1+): <no title>
Query17 (927.4218,1+): <no title>
Query18 (951.5530,1+): <no title>
Query19 (953.4604,1+): <no title>
Query20 (996.5237,1+): <no title>
Query21 (998.4718,1+): <no title>
Query22 (1017.5948,1+): <no title>
Query23 (1019.6071,1+): <no title>
Query24 (1033.6263,1+): <no title>
Query25 (1045.4912,1+): <no title>
Query26 (1051.6328,1+): <no title>
Query27 (1051.6328,1+): Locus:1..14.0.6
Query28 (1065.5367,1+): <no title>
Query29 (1131.6298,1+): <no title>
Query30 (1133.6357,1+): <no title>
Query31 (1136.6206,1+): <no title>
Query32 (1139.4833,1+): <no title>
Query33 (1142.5555,1+): <no title>
Query34 (1142.5555,1+): Locus:1..14.0.12
Query35 (1151.6481,1+): <no title>
Query36 (1156.5005,1+): <no title>
Query37 (1165.6569,1+): Locus:1..14.0.10
Query38 (1165.6569,1+): <no title>
Query39 (1175.5602,1+): <no title>
Query40 (1184.6132,1+): <no title>
Query41 (1189.4962,1+): <no title>
Query42 (1247.6283,1+): <no title>
Query43 (1267.5582,1+): <no title>
Query44 (1267.5582,1+): Locus:1..14.0.2
Query45 (1268.5472,1+): <no title>
Query46 (1287.5924,1+): <no title>
Query47 (1300.7646,1+): <no title>
Query48 (1305.5656,1+): <no title>
Query49 (1311.6147,1+): <no title>
Query50 (1334.7938,1+): <no title>
Query51 (1334.7938,1+): Locus:1..14.0.9
Query52 (1387.5682,1+): <no title>
Query53 (1387.5682,1+): Locus:1..14.0.3
Query54 (1396.6301,1+): <no title>
Query55 (1439.7159,1+): <no title>
Query56 (1448.8251,1+): <no title>
Query57 (1455.6167,1+): <no title>
Query58 (1455.6167,1+): Locus:1..14.0.1
Query59 (1475.6594,1+): <no title>
Query60 (1479.6964,1+): <no title>
Query61 (1479.6964,1+): Locus:1..14.0.5
Query62 (1498.6063,1+): <no title>
Query63 (1513.6995,1+): Locus:1..14.0.4

PDF-XChange
Click to buy NOW!
www.docu-track.com

PDF-XChange
Click to buy NOW!
www.docu-track.com

```
Query64 (1513.6995,1+): <no title>
Query65 (1550.6960,1+): <no title>
Query66 (1550.6960,1+): Locus:1..14.0.11
Query67 (1557.7151,1+): <no title>
Query68 (1567.6414,1+): Locus:1..14.0.8
Query69 (1567.6414,1+): <no title>
Query70 (1617.9590,1+): <no title>
Query71 (1647.7349,1+): <no title>
Query72 (1724.7261,1+): <no title>
Query73 (1731.9867,1+): <no title>
Query74 (1901.1331,1+): <no title>
Query75 (2044.9095,1+): <no title>
Query76 (2210.9834,1+): <no title>
Query77 (2283.0552,1+): <no title>
```

Mascot: http://www.matrixscience.com/
