# Supplementary material for: Alpha-2-Macroglobulin as a New Promising Biomarker Improving the Diagnostic Sensitivity of Bovine Paratuberculosis
Source: Front Vet Sci. 2021 Mar 5;8:637716. doi: 10.3389/fvets.2021.637716 (PMC7973028; doi:10.3389/fvets.2021.637716)
Supplement: Supplementary Data 1 — Mascot search results and raw data files for LC-MS/MS analysis for the identification of specific blood proteins in cattle with Johne's disease. [file Data_Sheet_1.zip › mascot/9 complement component C9 precursor_2.pdf]

PDF-XChange Click to buy NOW! www.docu-track.com

# MATRIX SCIENCE MASCOT Search Results

## Protein View: A0A3Q1MU98

### Complement component C9 OS=Bos taurus OX=9913 GN=C9 PE=1 SV=1

| | |
|---|---|
| Database: | Uniprot_bovine |
| Score: | 131 |
| Expect: | 1.1e-008 |
| Nominal mass ($M_r$): | 58618 |
| Calculated pI: | 5.74 |

Sequence similarity is available as an NCBI BLAST search of A0A3Q1MU98 against nr.

### Search parameters

| | |
|---|---|
| Enzyme: | Trypsin: cuts C-term side of KR unless next residue is P. |
| Fixed modifications: | Carbamidomethyl (C) |
| Variable modifications: | Oxidation (M) |

### Protein sequence coverage: 16%

Matched peptides shown in *bold red*.

```
  1 MSAGQRFAFA ICILEISLLR AGPTPSYDPA ERQGTPLPID CRMSSWSEWS
 51 KCDPCLKQMF RSRSIEIFGQ FNGRKCVDAV GDRQQCVPTE ACEDPEEGCG
101 NDFQCGTGRC IKNRLLCNED NDCGDYSDED NCEQDPRPPC RNRVVEESEL
151 ARTAGFGINI LGMDPLSTPF DNQYYNGLCD RVWDGNTLTY YRRPWNVASL
201 TYDTKADKNF RTENHEESIQ ILRTIIEEKK LNFNAGLSVK YTPVEAIEKN
251 KCVDLEHSDK GSTSSPSKLA AEAKFRFTYS KDDIYRLLSS YSAKQEKMFL
301 HVKGKVHLGR FVMRSRDVML QTTFLDSINT LPTTYEKGEY FAFLETYGTH
351 YSSSGSLGGL YELIYVLDKK SMEQKETNPE ADLFDDVITF IRGGTRKYAT
401 ELKEKLLRGA RMINVTDFVN WAASLNHAPV LISQKLVPIY DLIPVKMKDA
451 HLKKQNLERA IEDYINEFSV RKCQPCQNGG TVVLLDGECV CSCPKEFKGV
501 ACEIKK
```

Unformatted sequence string: 506 residues (for pasting into other applications).

Sort peptides by ◉ Residue Number ○ Increasing Mass ○ Decreasing Mass

Show predicted peptides also

| Query | Start – End | Observed | Mr(expt) | Mr(calc) | ppm | M | Score | Peptide |
|---|---|---|---|---|---|---|---|---|
| 36 | 33 – 42 | 1156.5005 | 1155.4932 | 1155.5706 | -67.0 | 0 | | R.QGTPLPIDCR.M |
| 43 | 64 – 74 | 1267.5582 | 1266.5509 | 1266.6357 | -66.9 | 0 | | R.SIEIFGQFNGR.K |
| 44 | 64 – 74 | 1267.5582 | 1266.5510 | 1266.6357 | -66.9 | 0 | 29 | R.SIEIFGQFNGR.K |
| 52 | 182 – 192 | 1387.5682 | 1386.5609 | 1386.6568 | -69.2 | 0 | | R.VWDGNTLTYYR.R |
| 53 | 182 – 192 | 1387.5682 | 1386.5610 | 1386.6568 | -69.1 | 0 | 18 | R.VWDGNTLTYYR.R |
| 65 | 193 – 205 | 1550.6960 | 1549.6887 | 1549.7889 | -64.6 | 0 | | R.RPWNVASLTYDTK.A |
| 66 | 193 – 205 | 1550.6960 | 1549.6888 | 1549.7889 | -64.6 | 0 | | R.RPWNVASLTYDTK.A |
| 9 | 287 – 294 | 868.4844 | 867.4771 | 867.4702 | 8.03 | 0 | | R.LLSSYSAK.Q |
| 39 | 295 – 303 | 1175.5602 | 1174.5529 | 1174.6169 | -54.4 | 1 | | K.QEKMFLHVK.G + Oxidation (M) |
| 2 | 447 – 453 | 842.4471 | 841.4398 | 841.4480 | -9.70 | 1 | | K.MKDAHLK.K |
| 57 | 460 – 471 | 1455.6167 | 1454.6094 | 1454.7041 | -65.1 | 0 | | R.AIEDYINEFSVR.K |
| 58 | 460 – 471 | 1455.6167 | 1454.6094 | 1454.7041 | -65.1 | 0 | 57 | R.AIEDYINEFSVR.K |

PDF-XChange Click to buy NOW! www.docu-track.com

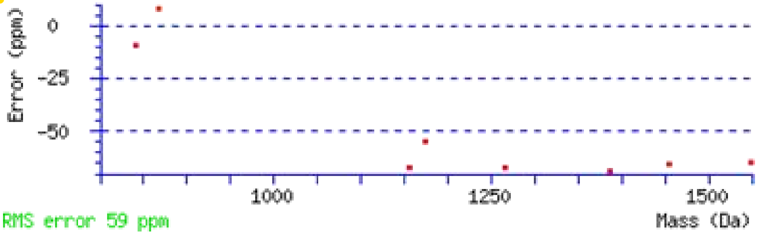


```
ID   A0A3Q1MU98_BOVIN        Unreviewed;        506 AA.
AC   A0A3Q1MU98;
DT   10-APR-2019, integrated into UniProtKB/TrEMBL.
DT   10-APR-2019, sequence version 1.
DT   07-OCT-2020, entry version 9.
DE   RecName: Full=Complement component C9 {ECO:0000256|ARBA:ARBA00018261};
GN   Name=C9 {ECO:0000313|Ensembl:ENSBTAP00000074107,
GN   ECO:0000313|VGNC:VGNC:26647};
OS   Bos taurus (Bovine).
OC   Eukaryota; Metazoa; Chordata; Craniata; Vertebrata; Euteleostomi; Mammalia;
OC   Eutheria; Laurasiatheria; Artiodactyla; Ruminantia; Pecora; Bovidae;
OC   Bovinae; Bos.
OX   NCBI_TaxID=9913 {ECO:0000313|Ensembl:ENSBTAP00000074107, ECO:0000313|Proteomes:UP000009[illegible]
RN   [1] {ECO:0000313|Ensembl:ENSBTAP00000074107, ECO:0000313|Proteomes:UP000009136}
RP   NUCLEOTIDE SEQUENCE [LARGE SCALE GENOMIC DNA].
RC   STRAIN=Hereford {ECO:0000313|Ensembl:ENSBTAP00000074107,
RC   ECO:0000313|Proteomes:UP000009136};
RA   Rosen B.D., Bickhart D.M., Koren S., Schnabel R.D., Hall R., Zimin A.,
RA   Dreischer C., Schultheiss S., Schroeder S.G., Elsik C.G., Couldrey C.,
RA   Liu G.E., Van Tassell C.P., Phillippy A.M., Smith T.P.L., Medrano J.F.;
RT   "ARS-UCD1.2.";
RL   Submitted (MAR-2018) to the EMBL/GenBank/DDBJ databases.
RN   [2] {ECO:0000313|Ensembl:ENSBTAP00000074107}
RP   IDENTIFICATION.
RC   STRAIN=Hereford {ECO:0000313|Ensembl:ENSBTAP00000074107};
RG   Ensembl;
RL   Submitted (JAN-2019) to UniProtKB.
CC   -!- SUBCELLULAR LOCATION: Cell membrane {ECO:0000256|ARBA:ARBA00004651};
CC       Multi-pass membrane protein {ECO:0000256|ARBA:ARBA00004651}. Membrane
CC       {ECO:0000256|ARBA:ARBA00004141}; Multi-pass membrane protein
CC       {ECO:0000256|ARBA:ARBA00004141}. Secreted
CC       {ECO:0000256|ARBA:ARBA00004613}. Target cell membrane
CC       {ECO:0000256|ARBA:ARBA00004276}; Multi-pass membrane protein
CC       {ECO:0000256|ARBA:ARBA00004276}.
CC   -!- SIMILARITY: Belongs to the complement C6/C7/C8/C9 family.
CC       {ECO:0000256|ARBA:ARBA00009214}.
CC   -!- CAUTION: Lacks conserved residue(s) required for the propagation of
CC       feature annotation. {ECO:0000256|PROSITE-ProRule:PRU00124}.
CC   ---------------------------------------------------------------------------
CC   Copyrighted by the UniProt Consortium, see https://www.uniprot.org/terms
CC   Distributed under the Creative Commons Attribution (CC BY 4.0) License
CC   ---------------------------------------------------------------------------
DR   Ensembl; ENSBTAT00000083951; ENSBTAP00000074107; ENSBTAG00000016149.
DR   VGNC; VGNC:26647; C9.
DR   GeneTree; ENSGT00940000159777; -.
DR   Proteomes; UP000009136; Chromosome 20.
DR   ExpressionAtlas; A0A3Q1MU98; baseline.
DR   GO; GO:0005576; C:extracellular region; IEA:UniProtKB-SubCell.
DR   GO; GO:0005579; C:membrane attack complex; IEA:UniProtKB-KW.
DR   GO; GO:0044218; C:other organism cell membrane; IEA:UniProtKB-SubCell.
DR   GO; GO:0006957; P:complement activation, alternative pathway; IEA:UniProtKB-KW.
DR   GO; GO:0006958; P:complement activation, classical pathway; IEA:UniProtKB-KW.
DR   GO; GO:0019835; P:cytolysis; IEA:UniProtKB-KW.
DR   CDD; cd00112; LDLa; 1.
DR   Gene3D; 2.20.100.10; -; 1.
DR   Gene3D; 4.10.400.10; -; 1.
DR   InterPro; IPR037567; Complement_C9.
DR   InterPro; IPR000742; EGF-like_dom.
DR   InterPro; IPR009030; Growth_fac_rcpt_cys_sf.
DR   InterPro; IPR036055; LDL_receptor-like_sf.
DR   InterPro; IPR023415; LDLR_class-A_CS.
DR   InterPro; IPR002172; LDrepeatLR_classA_rpt.
DR   InterPro; IPR001862; MAC_perforin.
```

PDF-XChange Click to buy NOW! www.docu-track.com

```
DR   InterPro; IPR020864; MACPF.
DR   InterPro; IPR020863; MACPF_CS.
DR   InterPro; IPR000884; TSP1_rpt.
DR   InterPro; IPR036383; TSP1_rpt_sf.
DR   PANTHER; PTHR45742:SF3; PTHR45742:SF3; 2.
DR   Pfam; PF00057; Ldl_recept_a; 1.
DR   Pfam; PF01823; MACPF; 1.
DR   PRINTS; PR00764; COMPLEMENTC9.
DR   SMART; SM00192; LDLa; 1.
DR   SMART; SM00457; MACPF; 1.
DR   SMART; SM00209; TSP1; 1.
DR   SUPFAM; SSF57184; SSF57184; 1.
DR   PROSITE; PS00022; EGF_1; 1.
DR   PROSITE; PS01209; LDLRA_1; 1.
DR   PROSITE; PS50068; LDLRA_2; 1.
DR   PROSITE; PS00279; MACPF_1; 1.
DR   PROSITE; PS51412; MACPF_2; 1.
DR   PROSITE; PS50092; TSP1; 1.
PE   1: Evidence at protein level;
KW   Coiled coil {ECO:0000256|SAM:Coils};
KW   Complement alternate pathway {ECO:0000256|ARBA:ARBA00023162};
KW   Complement pathway {ECO:0000256|ARBA:ARBA00023058};
KW   Cytolysis {ECO:0000256|ARBA:ARBA00023058};
KW   Disulfide bond {ECO:0000256|ARBA:ARBA00023157, ECO:0000256|PROSITE-
KW   ProRule:PRU00124}; Glycoprotein {ECO:0000256|ARBA:ARBA00023180};
KW   Immunity {ECO:0000256|ARBA:ARBA00023058, ECO:0000256|ARBA:ARBA00023162};
KW   Innate immunity {ECO:0000256|ARBA:ARBA00023058,
KW   ECO:0000256|ARBA:ARBA00023162};
KW   Membrane {ECO:0000256|ARBA:ARBA00022452, ECO:0000256|ARBA:ARBA00022537};
KW   Membrane attack complex {ECO:0000256|ARBA:ARBA00023058};
KW   Proteomics identification {ECO:0000213|PeptideAtlas:A0A3Q1MU98};
KW   Reference proteome {ECO:0000313|Proteomes:UP000009136};
KW   Secreted {ECO:0000256|ARBA:ARBA00022525}; Signal {ECO:0000256|SAM:SignalP};
KW   Target cell membrane {ECO:0000256|ARBA:ARBA00022537};
KW   Target membrane {ECO:0000256|ARBA:ARBA00022537};
KW   Transmembrane {ECO:0000256|ARBA:ARBA00022452};
KW   Transmembrane beta strand {ECO:0000256|ARBA:ARBA00022452}.
FT   SIGNAL          1..21
FT                   /evidence="ECO:0000256|SAM:SignalP"
FT   CHAIN           22..506
FT                   /note="Complement component C9"
FT                   /evidence="ECO:0000256|SAM:SignalP"
FT                   /id="PRO_5018711762"
FT   DOMAIN          136..472
FT                   /note="MACPF"
FT                   /evidence="ECO:0000259|PROSITE:PS51412"
FT   COILED          447..467
FT                   /evidence="ECO:0000256|SAM:Coils"
FT   DISULFID        105..123
FT                   /evidence="ECO:0000256|PROSITE-ProRule:PRU00124"
FT   DISULFID        117..132
FT                   /evidence="ECO:0000256|PROSITE-ProRule:PRU00124"
SQ   SEQUENCE   506 AA;  57400 MW;  96AF4A8F30D3EB39 CRC64;
     MSAGQRFAFA ICILEISLLR AGPTPSYDPA ERQGTPLPID CRMSSWSEWS KCDPCLKQMF
     RSRSIEIFGQ FNGRKCVDAV GDRQQCVPTE ACEDPEEGCG NDFQCGTGRC IKNRLLCNED
     NDCGDYSDED NCEQDPRPPC RNRVVEESEL ARTAGFGINI LGMDPLSTPF DNQYYNGLCD
     RVWDGNTLTY YRRPWNVASL TYDTKADKNF RTENHEESIQ ILRTIIEEKK LNFNAGLSVK
     YTPVEAIEKN KCVDLEHSDK GSTSSPSKLA AEAKFRFTYS KDDIYRLLSS YSAKQEKMFL
     HVKGKVHLGR FVMRSRDVML QTTFLDSINT LPTTYEKGEY FAFLETYGTH YSSSGSLGGL
     YELIYVLDKK SMEQKETNPE ADLFDDVITF IRGGTRKYAT ELKEKLLRGA RMINVTDFVN
     WAASLNHAPV LISQKLVPIY DLIPVKMKDA HLKKQNLERA IEDYINEFSV RKCQPCQNGG
     TVVLLDGECV CSCPKEFKGV ACEIKK
//
```

Mascot: http://www.matrixscience.com/
